# Antecedent presentation of neurological phenotypes in the Collaborative Cross reveals four classes with complex sex-dependencies

**DOI:** 10.1038/s41598-020-64862-z

**Published:** 2020-05-13

**Authors:** Raena Eldridge, Daniel Osorio, Katia Amstalden, Caitlin Edwards, Colin R. Young, James J. Cai, Kranti Konganti, Andrew Hillhouse, David W. Threadgill, C. Jane Welsh, Candice Brinkmeyer-Langford

**Affiliations:** 1 Texas A&M University, Department of Veterinary Integrative Biosciences, College Station, USA; 2 University of North Carolina, Department of Epidemiology, Chapel Hill, USA; 3 Texas A&M Institute for Genome Sciences and Society, College Station, USA; 4 Texas A&M University, Department of Molecular and Cellular Medicine, College Station, USA; 5 Texas A&M University, Department of Veterinary Pathobiology, College Station, USA

**Keywords:** Behavioural methods, Central nervous system infections

## Abstract

Antecedent viral infection may contribute to increased susceptibility to several neurological diseases, such as multiple sclerosis and Parkinson’s disease. Variation in clinical presentations of these diseases is often associated with gender, genetic background, or a combination of these and other factors. The complicated etiologies of these virally influenced diseases are difficult to study in conventional laboratory mouse models, which display a very limited number of phenotypes. We have used the genetically and phenotypically diverse Collaborative Cross mouse panel to examine complex neurological phenotypes after viral infection. Female and male mice from 18 CC strains were evaluated using a multifaceted phenotyping pipeline to define their unique disease profiles following infection with Theiler’s Murine Encephalomyelitis Virus, a neurotropic virus. We identified 4 distinct disease progression profiles based on limb-specific paresis and paralysis, tremors and seizures, and other clinical signs, along with separate gait profiles. We found that mice of the same strain had more similar profiles compared to those of different strains, and also identified strains and phenotypic parameters in which sex played a significant role in profile differences. These results demonstrate the value of using CC mice for studying complex disease subtypes influenced by sex and genetic background. Our findings will be useful for developing novel mouse models of virally induced neurological diseases with heterogenous presentation, an important step for designing personalized, precise treatments.

## Introduction

Prior viral infection is known or suspected to contribute to several neurological conditions, including multiple sclerosis (MS)^1–9^, amyotrophic lateral sclerosis (ALS)^10–12^, Parkinson’s disease (PD)^13–20^, and epilepsy^21–23^. In each of these diseases, the neurological effects of viral infection are variable among individuals, suggesting genetic background of the host influences the neurological sequelae. Precisely defined neurological phenotypes are necessary for genetic association studies to identify genetic effects and to reveal mechanisms.

Human studies have revealed different phenotypic subtypes in each of several neurological diseases. For example, PD subtypes can be tremor-dominant, akinetic-rigid, or mixed; each subtype is associated with different rates of disease progression and long-term prognoses^24–26^. MS can be categorized as relapsing-remitting or progressive^27^, with additional subtypes defined by clinical symptoms including clinically or radiologically isolated syndromes and secondary progressive MS^28^. Such classifications are important to determine the most effective treatment regimen, and in some cases to identify prognostic biomarkers e.g.,^24^. Disease subtypes are likely to arise from different contributing factors, including type of viral infection, sex of the affected individual, and interactions with genetic background.

Mice can be effective models for human diseases because genetic and environmental factors, biochemical mechanisms, and disease phenotypes are often similar in both species. Mice have contributed significantly to studies of human neurological diseases, via experiments using conventional inbred or genetically modified mouse strains (such as knock-out/knock-in models). Furthermore, numerous phenotyping methods are available for assessing the neurological functions of mice, longitudinal testing in mice is feasible and economical, and multiple states of disease progression can be readily tracked over time. However, limitations and caveats for using mice as models of human disease are a concern. Laboratory strains of mice used for research are frequently inbred, producing experimental reproducibility at the expense of reduced genetic and phenotypic heterogeneity. The Collaborative Cross (CC) mouse resource was developed to address this limitation. The different strains of the CC were derived from intercrosses of 8 founder strains, including 5 conventional inbred and 3 wild-derived strains, crossbred such that each resulting CC strain is genetically and phenotypically distinct, is reproducible, and contains a level of genetic diversity more typical of an individual human. CC panels of mouse strains feature the reproducibility of inbred strains and the genetic diversity of an outbred population^29,30^.

The CC is useful for modeling neurological conditions caused by Theiler’s murine encephalomyelitis virus (TMEV)^31^. TMEV is a neurotropic virus that may cause seizures and epilepsy, demyelinating disease similar to MS, or other phenotypes, depending on the genetic background of the host mouse strain^32–35^. In this study, we test the hypothesis that the CC strains could be used to develop precise neurological profiles of TMEV-infected mice – similar to subtypes observed in human neurological diseases.

We used a battery of neurological phenotyping protocols to define the comprehensive phenotypic profiles of TMEV-infected CC mice of different genetic backgrounds. We found that mice of the same strain have very similar profiles, compared to mice from different strains. We identified 4 distinct profiles of disease progression among 18 CC or CC-RIX (recombinant inbred intercrosses)^36^ backgrounds tested based on 5 neurological profiles, and 3 separate gait profiles. Not only did we confirm that animals of each strain displayed a unique phenotypic response to TMEV infection, we also discovered that sex influenced some phenotypes but not all strains exhibited sex differences for these phenotypes. These profiles can be used for future association studies to identify genetic factors and mechanisms that modify disease and add further dimensions to our understanding subtypes of neurological diseases.

## Materials and Methods

### Ethics statement

All procedures were approved by the Institutional Animal Care and Use Committee at Texas A&M University and performed under animal use protocol number 2017-0082. All experiments were performed in accordance with relevant guidelines and regulations. Mice were group-housed and all testing performed during the light phase.

### Mice

The mice used were from the CC resource and represent 18 genetically distinct strains^37^. Age- and sex-matched control mice of the same strain were used for all phenotyping activities described here. Often, mouse strains differ from each other in ways which may resemble abnormal neurological behaviors for one strain but not another. Also, age and/or sex can influence neurological phenotypes to different extents in different strains^31,38^. Therefore, we only considered comparisons to be relevant if proper controls were used.

### Infection

At 4 weeks of age, female (*n* = 50) and male (*n* = 50) mice were anesthetized by isoflurane inhalation (MWI, Meridian, ID) and injected intracerebrally with 5.0 × 10^4^ plaque-forming units (PFU) of the BeAn strain of TMEV (American Type Culture Collection [ATCC] VR 995, Manassas, VA) in 20 µl of PBS placed into the fenestra at a depth of approximately 1.5 mm^39,40^. Sham-infected mice (*n* = 25 females and 27 males) were anesthetized and injected with PBS only. Numbers of mice per strain are listed in Table 1.

**Table 1:** Strains used for these experiments included 14 CC strains and 4 recombinant inbred crosses (RIX) of CC strains. H2 haplotypes, deduced by the founder strain(s) from which the H2 region was inherited^30^, are shown in the second column. Numbers of uninfected mice in each category are shown in parentheses.

Strain	H2 haplotype	Number of infected mice (Number of uninfected mice)		
		Female	Male	Total
CC002	*b*	2 (2)	2 (2)	**4 (4)**
CC005	*z*	2 (2)	2 (2)	**4 (4)**
CC006	*het*	2 (2)	2 (2)	**4 (4)**
CC011	*g7*	2 (2)	2 (2)	**4 (4)**
CC012	*WSB/EiJ*	5 (1)	5 (1)	**10 (2)**
CC012xCC032	*WSB/EiJ × b*	2 (1)	4 (1)	**6 (2)**
CC013xCC041	*a × b*	3 (1)	2 (1)	**5 (2)**
CC015	*WSB/EiJ × a*	2 (1)	2 (1)	**4 (2)**
CC017	*CAST/EiJ*	2 (2)	2 (2)	**4 (4)**
CC023	*b*	2 (2)	2 (2)	**4 (4)**
CC025	*PWK/PhJ*	3 (1)	2 (0)	**5 (1)**
CC027	*het*	2 (2)	2 (2)	**4 (4)**
CC032xCC013	*b × a*	4 (1)	4 (1)	**8 (2)**
CC037	*b*	1 (1)	4 (2)	**5 (3)**
CC041xCC012	*b × WSB/EiJ*	8 (1)	6 (1)	**14 (2)**
CC051	*b × a*	3 (1)	2 (2)	**5 (3)**
CC057	*z*	3 (1)	3 (1)	**6 (2)**
CC078	*z*	3 (1)	2 (1)	**5 (2)**
**TOTAL**		50 (25)	50 (27)	**100 (52)**

### Weight

The mice were weighed at least once prior to infection, then twice daily for the first 14 days post-infection (the acute phase of TMEV infection^32,41^) to capture the initial weight changes as an indicator of response to infection. The mice were then weighed daily to monitor their weights during the chronic phase of TMEV infection, defined as 35–90 days post-infection (dpi), as well as during the transitional period between acute and chronic phases (15–34 dpi).

### Qualitative neurological phenotyping

Mice were evaluated twice daily for the first two weeks post-infection to capture the rapidly changing phenotypic landscape of the acute phase of TMEV infection. Thereafter, the mice were phenotyped once weekly to continue to monitor changing phenotypes without adding undue stress. To maintain consistency with previous studies we evaluated neurological phenotypes using methods frequently employed by others^32,33,42^. These are referred to here as “qualitative phenotypes” because their measurements are less objective than those phenotypes measured via specialized equipment, here referred to as “quantitative phenotyping”.

#### Seizure scoring

Inflammation within the CNS can induce seizures of varying severity. During the phenotyping process, the mice were carefully observed for seizures (which tended to be more prevalent during handling due to increased stress/excitement). The seizures were measured on the Racine scale ranging from 1–5 as follows: stage 1 – mouth and facial movement; stage 2 – head nodding; stage 3 – forelimb clonus; stage 4 – rearing with forelimb clonus; stage 5 – rearing and falling with forelimb clonus^43^.

#### Encephalitis

Behavioral symptoms of encephalitis or swelling of the brain can include a swollen head (compared to uninfected control mice of the same strain), squinting eyes (ptosis), ruffled fur, hunched posture, lethargy, and ataxia. Previous studies have used a combination of these symptoms to assign an overall score for encephalitis (e.g.^44^); however, this method of scoring requires that the severities of these symptoms progress in unison. We chose to evaluate piloerection, hunch, and ptosis separately because we have found, for CC strains, these symptoms appear with different severities^45^. Therefore, the score assigned to encephalitis as measured in this category was based solely on the appearance of the head and face of the mouse. A score of 1 was given if the eyes appeared sunken or squinty (ptosis), with a swollen head, compared to uninfected control mice from the same strain. A score of 0 was given if the eyes and head appeared normal.

#### Hunch

Hunched posture is a clinical sign of encephalitis^38,39,42^. During the phenotyping process, mice were evaluated for signs of hunched posture in 3 different ways. First, the mice were observed before being removed from the cage (while they were most at ease). Second, their stance and walking behavior were observed when they had been removed from the cage and released to walk around freely. Third, the mice were placed on a grate and gently pulled backwards to elongate their spine which was then palpated to feel for curvature. The mice were given a score of 0 if there was no hunch, and a score of 1 if hunching was observed.

#### Piloerection

Fur ruffling (piloerection) is an indicator of encephalitis if observed in an infected mouse but not uninfected mice of the same strain and sex^38,39,46,47^. Ruffling was scored as 1 if present, and 0 if not.

#### Righting reflex

The righting reflex is a simple test of motor neuron function used in rodent models. Each mouse was flipped onto its back on a flat surface and the time required to right itself (to a prone position, with all four paws underneath it) was measured for at least 2 consecutive trials. Longer times, up to total loss of righting reflex, were seen with decreased motor neuron functioning, such as limb paresis or paralysis or loss of proprioception. Each mouse was scored based on how many seconds it took to right itself (0–5 seconds).

#### Clonus

The mice were scored for clonus based on the method described previously^48^. Each mouse was assigned the following score: 0 – no clonus; 1 – clonus. Those with clonus were further graded based on the type of clonus present: A – forelimb clonus as indicated by clasping of the forelimbs together or repeated clenching forepaws into fists; B – hindlimb clonus as indicated by clasping of the hindlimbs together or splaying and curling of the hind toes; C – both forelimb clonus (A) and hindlimb clonus (B); D – clonus in combination with the spastic twisting of the body.

#### Paralysis and paresis

Mice were observed for signs of paralysis or paresis during the inverted grate test and when walking on a flat surface. Each individual paw, as well as each limb, was evaluated separately to define the location of the affected area. Each mouse was placed on a grate which was then turned over. The mouse was then allowed to navigate hanging from the grate, and its movements were evaluated based on the following scale: 0 – mouse was active and able to walk with no signs of weakness; 1 – limb dangled from the grate for several seconds before recovery; 2 – limb dangled off of grate more than 50% of the time; 3 – limb continuously dangled from grate (due to paralysis or paresis); 4 – mouse was unable to stay on grate due to whole body weakness (in this case, every limb would receive a score of 4). A limb retaining limited movement was labeled as showing paresis. Whole leg paralysis was defined as paralysis of the entire leg (including the paw), while paw paralysis was defined as paralysis of only the paw (but retention of leg function). If a mouse’s paw was completely unable to grip the grate, the paw was classified as paralyzed. If the same limb was then observed to not move when walking on a flat surface, the whole limb was classified as paralyzed.

Additionally, paralysis was classified as either spastic or flaccid. In cases of spastic whole limb paralysis, the entire limb was immobile and tucked into the body of the mouse. Spastic paw paralysis resulted in a mouse continuously contracting (making a fist) or splaying its toes, either of which resulted in inability to grip the grate. Flaccid whole limb paralysis resulted in the entire limb dragging while walking and pendulant while on the grate. Similarly, flaccid paw paralysis resulted in a dragging/hanging paw.

### Quantitative neurological phenotyping

#### DigiGait

DigiGait (Mouse Specifics, Boston, MA, USA) was used for quantitative analysis of 32–34 different gait parameters per limb. Measurements were taken 1 day prior to infection (T0, baseline), 21dpi (T1, mid-infection), and 89dpi (T2, one day prior to euthanization). The 21dpi time point was selected based on previous work showing that, while some strains are more susceptible to the adverse effects of the infection during one phase vs. the other, the “transitional” period between acute and chronic phases seems to be a time of stability^31^. 21dpi was therefore selected to capture a snapshot of mid-infection gait profiles while avoiding undue stress to any strain.

Gait abnormalities (including spasticity and paralysis) have been previously shown to correlate to disease progression during TMEV infection in mice^49^. DigiGait measurements provided objective and quantifiable data including different aspects of motor impairment. Critically, the DigiGait technology detected abnormalities in gait and limb function not perceivable by pure observation. Parameters were scored for each individual limb; therefore, most parameters had 4 values per mouse.

### RNA isolation and sequencing

RNA samples isolated from hippocampus and thoracic spinal cords of 90 mice, representing 13 CC mouse strains (see Table S1 for details), were quantified with the Qubit Fluorometer (Life Technologies) with a broad range RNA assay and concentrations were normalized for library preparation. RNA quality was verified on the Agilent TapeStation with a RNA ScreenTape. Unfortunately, high-quality RNA was not available for the 90dpi time point for both infected and uninfected mice from strains CC012, CC012xCC032, CC013xCC041, CC057 and CC078. Messenger RNA sequencing libraries were prepared using the Illumina TruSeq Stranded mRNA preparation kit. Barcoded libraries were pooled at equimolar concentrations and sequenced on an Illumina NovaSeq 6000 S4 150 cycle paired-end sequencing kit. Sequencing FASTQ files were generated by Illumnia BaseSpace using the bcltofastq program.

A total of approximately 6.7 billion paired-end reads were checked to trim any adapter sequences and low quality bases using Trimmomatic^50^ resulting in approximately 6.0 billion filtered reads (89.3%) out of which a total of 5.6 billion filtered reads (approximately 92.3%) mapped to the combined reference sequences of *Mus musculus* (mm10 ENSEMBL release 99) genome assembly and the TMEV virus reference sequence from NCBI (https://www.ncbi.nlm.nih.gov/nuccore/M16020.1/). Read mapping was performed using STAR^51^. Transcript wise counts were generated using featureCounts tool from the SUBREAD package^52^. Differential gene expression tests were then performed used DESeq2^53^ following recommended guidelines by the authors. Expression (Fold Change) of the polyprotein AAA47930.1 of the TMEV virus was measured after DEG (Differentially Expressed Genes) test was calculated using DESeq2 based on infection state (i.e. infection is present) for each strain.

### Statistical analysis

#### Qualitative data

Qualitative data for each mouse was modeled as censored data and fitted using Cox proportional hazard regression. Differences between mice stratified for each covariate (including infection status, sex, and strain) were quantified. For each covariate, Kaplan-Meyer curves were used to show the cumulative probability of phenotype onset of mice during the first 70 days post-infection.

#### Quantitative data

Within-group mean differences between infected and uninfected mice were performed for each strain and sex independently. To identify patterns between strains in response to the virus infection, the differences were standardized (by subtracting the mean and dividing by the standard deviation, Table S6), and clustered with the Pearson correlation coefficient as a measurement of distance, using the ‘single’ method of agglomeration. To define the strength of the similarity between strains, 1000 pseudo replicates of bootstrap were performed using the ‘pvclust’ R package^54^, and the identified groups with an approximately unbiased p-value (AU) lower or equal to 0.05 were defined as significant. Phenotypic differences between the identified groups was performed using ANOVA. Statistics for all DigiGait parameters are provided in File S2; raw data is presented in Table S3.

## Results

### CC strains distinguish four categories of progression following TMEV-infection

**Figure 1:**
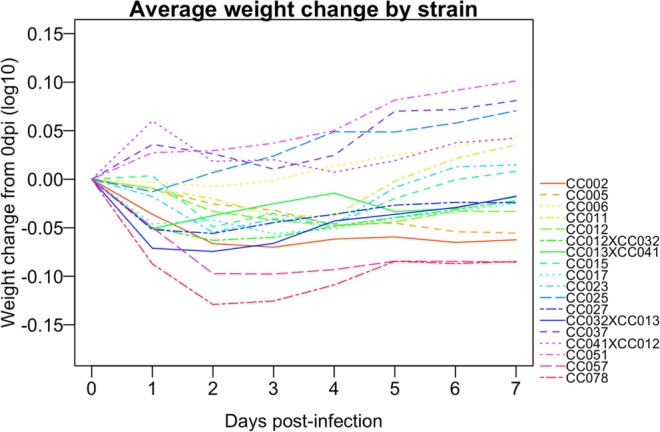
Weight changes during first week post-infection. Average post-infection weight changes during the first 7 dpi demonstrated varied levels of susceptibility to TMEV-induced weight loss.

The amount of weight lost post-TMEV infection varies among standard inbred strains and does not always correlate with long-term pathology. In previous studies, SJL/J mice, considered highly susceptible to TMEV-induced demyelinating disease, lost weight^38^ while resistant C57BL/6 lost little to no weight depending on seizure activity^55^. CBA mice, considered to have intermediate level of susceptibility to TMEV-induced demyelination, lost more weight than SJL^39^. Therefore, we anticipated a range of weight loss following TMEV infection in the more-diverse CC strains. We evaluated post-infection mortality and weight loss as a preliminary assessment of response to TMEV infection in 14 CC strains and 4 CC-RIX. Seven infected mice (out of 100 total) died over the course of the experiment: CC002 female (at 58 dpi), CC017 female (at 5 dpi), CC023 female (at 13 dpi), CC023 male (at 46 dpi), CC057 male (at 37 dpi), CC078 female (at 20 dpi), and CC078 male (at 11 dpi) (Fig. S1). Mice of these strains were among those that lost the most weight post-infection and struggled to regain pre-infection weights during the acute phase. Most strains (83%) initially lost some weight after infection, but by 7 dpi 6 strains (out of 18) had weight gains that surpassed their pre-infection weights (Fig. 1). CC023 mice experienced a second episode of weight loss over the course of 7–10 dpi, such that they again weighed less than their pre-infection weights. The greatest weight lost by any strain immediately following infection (1 dpi) was ~25% for CC078 mice; CC057 lost about 20%. By comparison, sham-infected mice of all strains had little to no weight loss. Weight loss stabilized by the end of the acute phase for all strains (Fig. S2).

**Figure 2:**
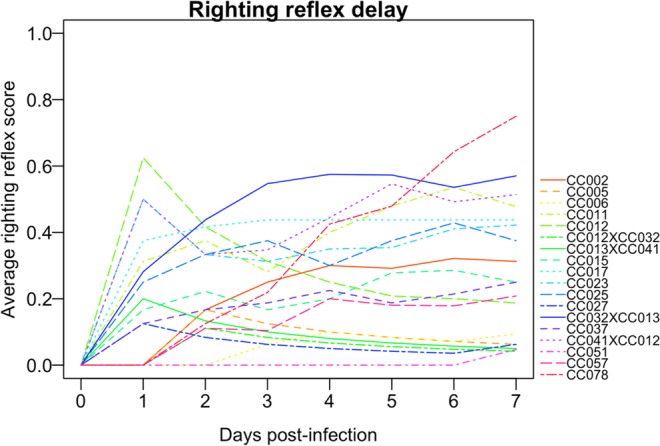
Onset of delayed righting reflex. Strains differed by date of first observation of delayed righting reflex, indicating a delayed motor response.

We evaluated TMEV-infected and sham-infected mice based on frequency of observed phenotypes known to be associated with TMEV pathology, including delayed righting reflex, paresis and/or paralysis of each limb, dystonia phenotypes (frequency of clonus and seizure observations), and sickness/pain phenotypes (frequency of hunching, ruffling/piloerection, and “encephalitis” as defined by ptosis). Details and statistics for each phenotype are provided in File S1. All strains exhibited delays in righting reflex after infection, though mice from some strains maintained the ability to immediately right themselves starting from 2 dpi or later, while others struggled to right themselves as soon as 1 dpi (Fig. 2). As a delayed righting reflex indicates attenuated motor coordination, these findings distinguish strains with early versus late onset of motor neuron deficits.

Paresis (weakness) and paralysis are well-known features of TMEV infection in susceptible strains such as SJL/J and CBA^39,42^. We observed, consistent with prior studies^42^, that hind limbs of infected mice tended to exhibit paresis earlier in the acute phase, and slightly more frequently overall, compared to fore limbs. Two strains had no significant paresis in *any* limb.

Paralysis is typically considered a long-term outcome of limb weakness caused by TMEV infection of SJL/J mice^32,42^. For this study, however, paresis was not highly predictive of paralysis: many strains that were physically weak during the acute phase did not “progress” to full paralysis. Furthermore, paralysis did not affect all limbs, or all strains, equally. The right hind limb was paralyzed most often (7 strains), and the left fore limb was paralyzed in mice from 4 strains (3 of which had right hind limb paralysis). Types of paralysis also varied. Two strains presented spastic paw or whole limb paralysis, with affected paws and limbs tightly clenched; while 3 other strains had flaccid whole limb paralysis, with affected limbs dragging limply. Furthermore, one strain had flaccid paw paralysis, which resolved by 50 dpi, and 3 other strains exhibited spastic paw paralysis with trembling. The trembling could be a symptom of dystonia.

Dystonia can manifest at several levels post-TMEV infection. The most pronounced is epileptic seizures, observed previously in certain mouse strains deemed “resistant” to TMEV-induced demyelination, e.g. C57BL/6^55–57^ and certain CC strains^45^. In the present study, seizures (Racine scale 4–5) were observed in 6 strains during the acute phase, all prior to 7 dpi. Thereafter seizure activity ceased, except for single events in each of 2 of these strains during the chronic phase. However, we noted tremors throughout the chronic phase (for example, batting the paw while trying to grip the grate) in 4 strains; tremors are a focal manifestation of dystonia. Clonus, at the milder end of the dystonic phenotype spectrum (a level 3 on the Racine seizure scale), was observed at least once in most (94%) strains but significantly more often in 10 strains. When held by the tail, forelimb clasping or hindlimb twisting were prevalent in 5 of these strains. Additionally, 2 more of strains with clonus displayed full-body twisting, and mice of the remaining 3 strains exhibited a variety of types of clonus.

Regardless of whether mice demonstrate any neurological phenotype after TMEV infection, they often present behaviors indicative of sickness or pain. Of the strains studied here, 2 in particular showed significantly more pain-like behavior, as evidenced by ruffled fur, hunched back, and encephalitis (or ptosis); 4 other strains also were significantly affected with at least 2 of these 3 pain phenotypes. Though more subjective, these pain-related signs provide useful insight into the variable impacts of TMEV infection on the physical well-being of the mice: some strains experienced profound weakness or paralysis but did not show signs of pain.

**Figure 3:**
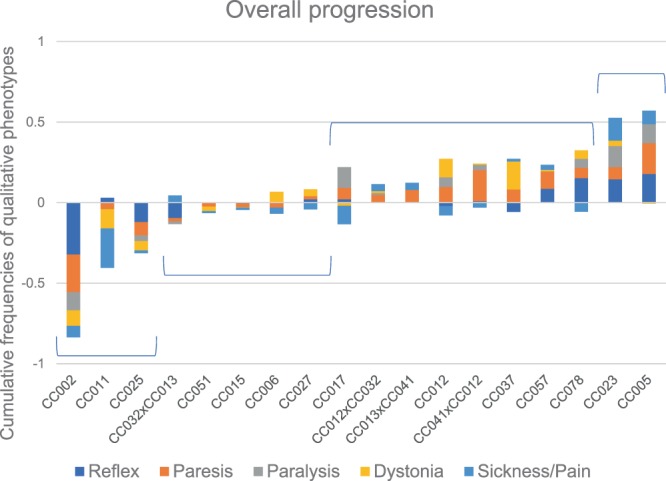
Progression of neurological phenotypes varied by strain. Observation frequencies for qualitative phenotypes (righting reflex, paresis, paralysis, dystonia, and sickness/pain) were compiled together for 14 dpi and 90 dpi and the differences from 14–90 dpi compared to visualize how each strain fared throughout the chronic phase of TMEV infection (“progression score”). Strains included in each successive progression score are delineated by brackets.

Using observed frequencies of each of the aforementioned phenotypes, we assigned each strain to one of four categories of disease progression, based on rate of progression, trajectory, and phenotypic subtypes. Overall progression scores were determined based on cumulative frequencies of delayed righting reflex, paresis, paralysis, dystonia, and pain behaviors observed at 14 dpi compared to 90 dpi (Fig. 3). The frequency of each phenotype was calculated as the number of observations of that phenotype for a given strain by a given date (e.g. 14 dpi or 90 dpi) divided by the number of total measurements by that date. Thus, those strains with an increase in the frequency of observed phenotypes from 14 to 90 dpi were deemed to have a more pronounced progression than those strains for which the frequency of observed phenotypes declined in that same time period. The four categories of disease progression were:

- Remitting: defined as having a progression score >1 standard deviation *below* the average progression score for all strains.
- Non-progressive: defined as having a *negative* progression score within 1 standard deviation of the average progression score for all strains (indicating a neutral to improved disease trajectory from 14–90 dpi).
- Moderately progressive: defined as having a *positive* progression score within 1 standard deviation of the average progression score for all strains (indicating overall progression from 14–90 dpi).
- Most progressive: defined as having a progression score >1 standard deviation *above* the average progression score for all strains.

### Distinct impacts of TMEV infection on gait was not predictive by pre-infection gait

DigiGait measurements taken pre-infection (T0) revealed gait differences characteristic of each strain. Post-infection measurements, taken at the juncture between acute and chronic phases (T1, at 21 dpi) and just prior to sacrifice (T2, at 89 dpi), revealed how TMEV infection affected the gaits of each strain, and whether those differences persisted long-term. For some strains, gait changes also varied by sex and/or limb. The strains that appeared to be similarly affected when evaluated by qualitative measures did not have similar DigiGait profiles. We propose that DigiGait measurements provided a more nuanced and complex assessment of TMEV-induced neurological sequelae.

**Figure 4:**
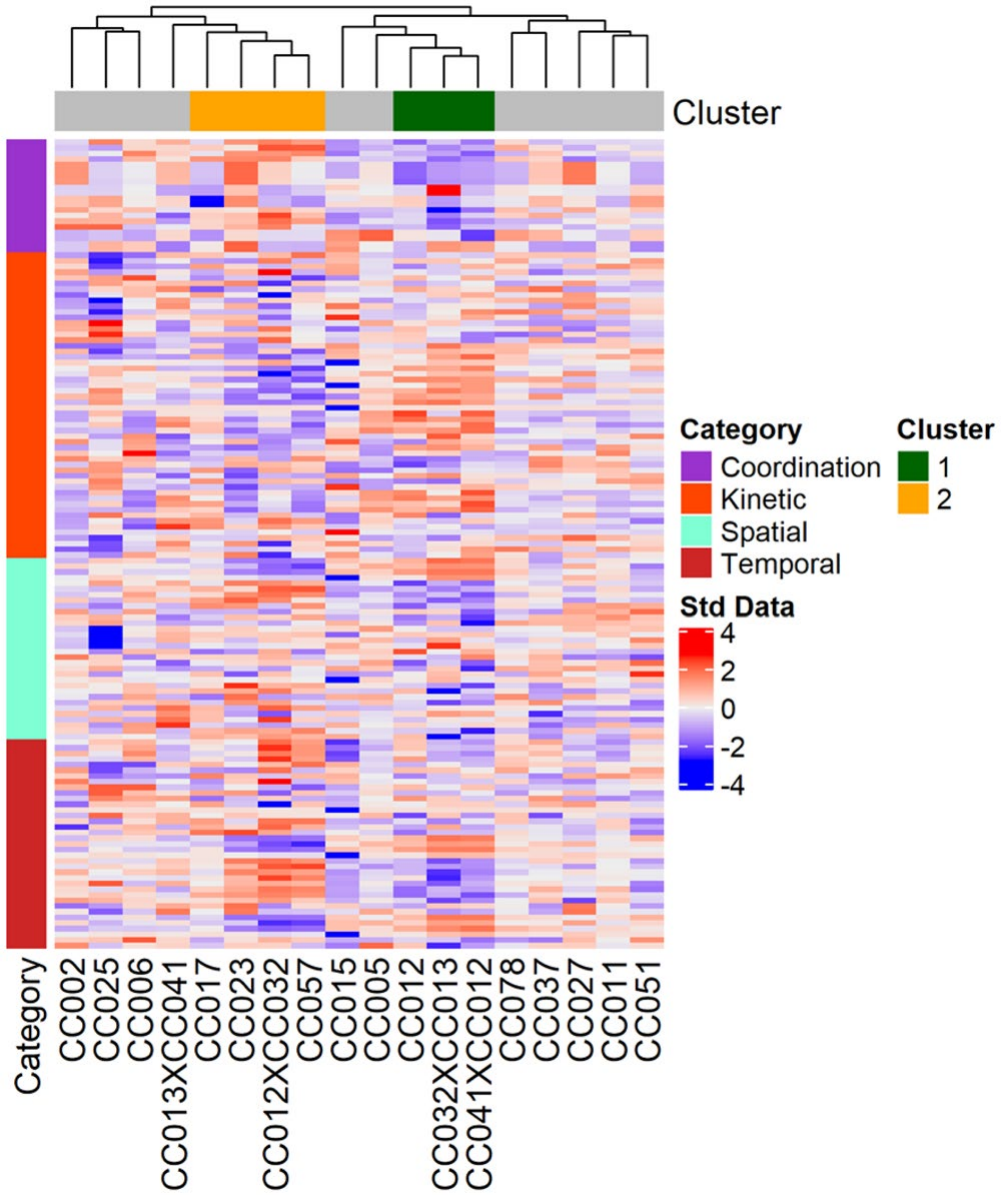
Gait parameters were affected differently depending on mouse strain. Pearson correlation clustering revealed two major clusters, 1 (green bar) and 2 (orange bar), which distinguish between strains with significantly different gait parameter measurements. Each individual parameter was categorized based on the primary gait characteristic it measured^58–60^; categories for each parameter are listed in Table S5. The two clusters do not include those strains for which sex was a major influence on gait parameters. The results shown here include data from infected mice at all time points; additional granularity (e.g. separate time points and sexes) is described in the text and shown in figures included in Figs. S4 and S5.

Hierarchical clustering of all DigiGait measurements from all infected mice grouped mouse strains with similar phenotypes/progression, as measured by significantly different DigiGait parameters, into two significant clusters (Fig. 4). These clusters did not differentiate between “severely” affected and “less-severely” affected strains; rather, these clusters distinguished mouse strains with relatively faster, longer strides (Category 1) from those with a distinctively bradykinetic gait, with short stride lengths and greater ataxia (Category 2) (see Figure S3 for comparisons of all significant DigiGait parameters differentiating the two categories, Table S2 for a complete list of parameters with p-values, and Table S3 for complete raw and statistical data for DigiGait experiments). The 11 strains excluded from these two clusters (marked in grey) had less well-defined gait profiles, and/or were affected in ways not readily measured with gait parameters. Some of the un-clustered strains showed sex differences, as described below.

Clustering was repeated for each of 3 time points (pre-infection, mid-infection, and just prior to sacrifice) to better define changes in DigiGait profiles over time (Figure S4). We used differences in measurement compared to pre-infection for the clustering analyses of the 2 post-infection time points. One caveat of these analyses is that measurements were not available for all strains at all time points, because we excluded unreliable data when the performance of some individuals in a strain could not be measured accurately, often because paralysis or other behavioral issues rendered the mice unable to complete the task. All but 1 strain clustered together for the pre-infection baseline time point, reflecting that inherent gait differences between CC strains did not significantly contribute to post-TMEV infection gait profiles. Strains included in the 2 clusters shown in Fig. 4 were not located near each other in the pre-infection tree, suggesting that the post-infection clusters were more strongly influenced by infection than by pre-existing differences. Two significant clusters at T1 (mid-infection, 21 dpi) revealed that the post-infection gaits of the different strains were distinguishable by spatial/temporal ambulation metrics such as stride lengths (Category 1-like), and ataxia and other postural or balance metrics (Category 2-like). By T2, or 89 dpi, the Category 1 strains remained within the same group, while Category 2 strains were more dispersed. This suggests that spatial and temporal gait parameters were more prognostic overall, while the metrics pertaining to coordination and balance were less consistent.

Complete strain profiles, including qualitative phenotype summaries, are available in Table S4.

### Sex differences in TMEV-induced phenotypes varied across strains

**Figure 5:**
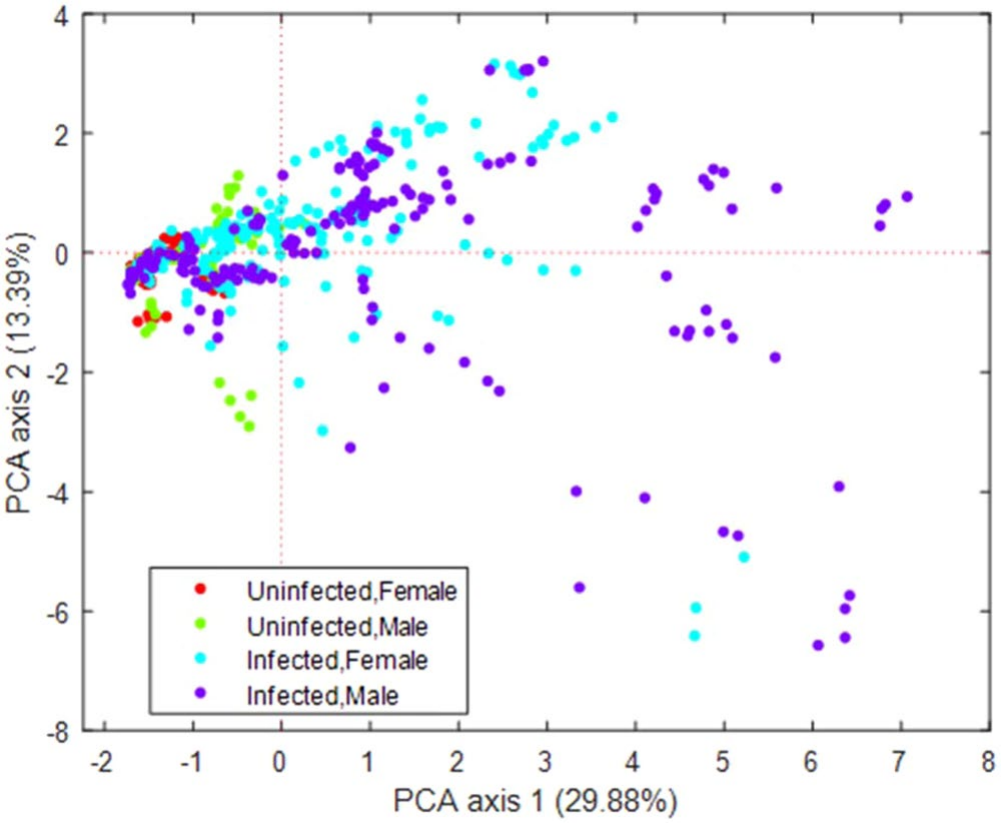
Principal component analysis showed that both infection and sex influenced phenotypes. Principal component analysis (PCA) based on 538 qualitative measurements (phenotypic traits) in mice from 18 strains revealed greater dispersion of qualitative phenotypes in infected vs. uninfected mice. Greater phenotypic dispersion was also seen in males compared to females in each treatment group.

Principal components analysis of qualitative phenotype measurements revealed differences based on infection status and sex (Fig. 5). Therefore, we evaluated TMEV-induced phenotypes for sex differences at multiple time points throughout the experiment (File S1). Paresis, for example, was observed earlier and more frequently in male mice: as early as 1 dpi, male mice of 6 strains (but females from only 2 of these strains) demonstrated weakened grips when walking on an inverted grate. Females and males initially exhibited paralysis at about the same time and frequency (by 7 dpi, females from 4 strains, and males from 3 strains, showed paralysis), but overall, males developed hindlimb paralysis more frequently than females. Sex influenced dystonia phenotypes as well, though not substantially. In one strain, clonus increased in male mice compared to females during the chronic phase. In another strain, females displayed clonus more frequently over time compared to males.

Overall progression rates also varied enough for females and males to be classified with different disease progression scores for 8 strains (Figure S6). For example, CC025 females showed substantial improvement from 14–90pi, such that the cumulative frequencies of observed qualitative phenotypes decreased between those dates (“remitting” progression category). CC025 males did not demonstrate as many of these phenotypes from the beginning, and because there was little room for improvement their disease progression course was classified as “moderately progressive.” CC037 and CC078 males, meanwhile, had higher incidences of the qualitative phenotypes from the beginning, but the frequency with which these phenotypes were observed leveled off such that the disease course of the males was categorized as “non-progressive.” On the other hand, while females from these strains initially displayed fewer qualitative phenotypes, the frequency with which the phenotypes were observed increased substantially over time, resulting in a category of “most progressive” for CC037 and CC078 females. Similar categorical differences distinguished the disease progression courses for CC005 (females were moderately progressive; males were most progressive), CC011 (females were non-progressive while males were remitting), CC017 (females were non-progressive while males were moderately progressive), CC023 (females were most progressive, males were moderately progressive), CC027 (males had a non-progressive disease course while females were moderately progressive), and CC057 (females were non-progressive; males were most progressive).

We used gait parameters to further investigate sex differences between strains. Quantitative DigiGait measurements analyzed by hierarchical clustering (Fig. 4) revealed that 11 strains did not group into the 2 major clusters of the overall heatmap. Therefore, we built sex-specific clusters to identify significant differences between sexes (Figure S5). We considered sex differences to be in response to TMEV infection because DigiGait software takes body size and sex into consideration when analyzing data. Females and males of strains included in the original major clusters of the overall heatmap – Clusters 1 and 2 (Fig. 4) – generally remained located near each other. However, for females, fewer strains were included in clusters (5 vs. 7 when both sexes were considered together). Strains CC002 and CC013xCC041 clustered with CC012, which had previously clustered with CC041xCC012 and CC032xCC013; the latter two strains remained in a separate cluster together. The 2 female clusters were not as distinct as those of the overall heatmap, and when only those DigiGait parameters representing principal components were included, there was no longer any statistical significance. This indicates a greater diversity in the DigiGait measurements for the female mice, and suggests that TMEV infection affected the gaits of females more subtly compared to males. By comparison, the DigiGait parameters of male mice clustered into more distinct profiles which were very different in composition than those of females. Parameters related to coordination and kinetic aspects of gait were distinguishing factors between the 2 clusters, even when only principal components were considered, indicating that TMEV infection played a bigger role in the gait dynamics of male mice. Strain CC025 did not appear in the male-specific tree due to insufficient data.

In only 2 strains, CC041xCC012 and CC032xCC013, was sex not an influence on gait measurements. The distinct gait differences between the strains of Clusters 1 and 2 of the overall heatmap shown in Fig. 4, along with their appreciable relevance to human disease, disappeared when each sex was evaluated separately leading us to conclude that both genetic background and sex substantially influenced the many diverse phenotypic produced by TMEV infection.

### Phenotypes of RIX strains were distinct to those of parental strains

The 4 recombinant-inbred backcross strains presented an opportunity to identify relationships between phenotypes in cases where parental phenotypes were known. For example, we have previously profiled TMEV-induced phenotypes for both CC013 and CC041^45^. Each of these strains demonstrated very strong but different responses to TMEV, with CC013 mice exhibiting seizures while CC041 mice experienced profound hind limb paralysis. However, none of these parental phenotypes were observed for CC013xCC041 mice. Similarly, CC032xCC013 and CC041xCC012 presented with milder phenotypes than the parental strains. These 3 RIX strains may exemplify epistasis and are likely tolerant, if not resistant, to TMEV infection unlike the parental strains. Although CC032 mice were not available for comparison, CC012xCC032 was the only RIX strain to demonstrate a TMEV response with similarities to a human condition, as described in Discussion.

### Levels of TMEV RNA at 90dpi indicate that viral clearance from the CNS is not correlative with H2 haplotype, disease progression, or phenotypes

During the chronic phase of infection (>35 dpi), TMEV RNA can still be detected in the CNS tissues of some strains of mice, with the highest levels measured in “susceptible” mouse strains such as SJL/J^61–64^. Susceptibility and resistance to TMEV have been attributed in part to certain haplotypes of a region of the genome containing many genes involved in immune response; this region is termed major histocompatibility complex (MHC; known as H2 in mice)^34^. Though not all H2 haplotypes have been classified in relation to TMEV response, some have: for example, H2^s^ has been associated with susceptibility to TMEV-induced demyelinating disease, while H2^b^ is associated with TMEV resistance^65^. We have previously demonstrated that H2 haplotype is not predictive of TMEV clearance or phenotypes in CC mice^31^. As shown in Table 1, many strains evaluated in this study did not share the same H2. However, the H2^z^ haplotype found in CC005, CC057, and CC078 was associated with high progression scores and increased mortality: 5 of the 7 mice that died during the experiment were from these strains. These findings suggest H2^z^ as a susceptibility locus for certain TMEV-induced phenotypes. Additional non-H2 loci are also likely to influence the trajectory of neurological sequelae attributed to TMEV infection.

Like the neurological phenotypes described above, levels of TMEV RNA in infected vs. uninfected mice varied between different CC strains. Expression of TMEV RNA did not differ significantly between sexes or tissues. TMEV RNA measurements tended to fall into one of two distinct groups: values of zero or less, implying viral clearance; or ≥20-fold increase compared to uninfected mice of the same strain, implying persistent infection. At 90 dpi, strains with higher progression scores – those falling into the categories “moderately progressive” or “most progressive” – tended to have higher levels of TMEV RNA, and some considered “non-progressive” or “remitting” had virtually no detectable TMEV RNA. However, CC011 (a remitting strain) and CC006, CC015, and CC027 (all non-progressive strains) still showed evidence of persistent infection. Strains CC002, CC023, and CC037 all carried the TMEV resistance haplotype H2^b^, but of these, only CC002 showed evidence suggesting viral clearance. CC023, considered “most progressive,” had measurable levels of TMEV RNA, but while these measurements suggested viral persistence, this strain did not have the highest levels as one might expect based on disease phenotypes. These findings suggest the clearance/persistence of TMEV is not a directly correlative measure for disease severity.

RNA sequencing comparisons of TMEV levels are summarized in Table S7.

## Discussion

In humans, the spectrum of neurological phenotypes that can follow viral infection may range from a life without neurological dysfunction to one of dramatic disability, or death. Differences such as sex or ethnicity influence post-infection disease patterns, posing a challenge to understand disease process using humans or even conventional mouse strains that typically focus on specific disease states with limited genetic and phenotypic diversity. In this study we have demonstrated that mice can, in fact, model the phenotypic diversity observed in humans. Genetically different CC strains present with strikingly different phenotypes and disease progression after infection with the same neurotropic virus. The extensive phenotyping we describe captures several disease subtypes and will support future studies to identify the mechanisms behind each category.

Neurological diseases in humans, including those known or suspected to follow viral infection, often vary in presentation depending on sex or race. For example, MS occurs 3 times as often in women as in men^66^, and although MS has long been considered as affecting people of predominantly northern European ancestry, African Americans have a higher incidence of MS and exhibit different disease phenotypes compared to Caucasians^67–69^; non-white Hispanics may have a lower incidence of MS^68^. PD, meanwhile, affects men twice as often as women^70^, and some studies report race differences in PD diagnosis; due to sampling differences, these studies disagree on which race is most frequently affected^71–73^. Furthermore, females and males experience different PD symptoms and disease phenotypes^74^. ALS can also present with different disease phenotypes, although neuronal degeneration and weakness are hallmarks of ALS. Some ALS patients also experience additional symptoms such as tremors or cognitive deficits^10^. Males, and non-Hispanic Caucasians, have an increased incidence of ALS, according to annual reports of the National ALS Registry e.g.,^75^.

Another cause for diverse presentations of neurological diseases is differences in response to antecedent viral infections. Females and males differ in immunological responses to viruses, which contribute to sex differences in disease prevalence e.g.,^76,77^. For example, the skewed sex ratio for MS incidence has been attributed in part to sex differences in those infected with the Epstein-Barr virus during adolescence^78^. Sex differences in mice in response to TMEV infection have also been described, in context of hormonal regulation of immune response^79–81^. We observed sex differences in this study, but not in all strains or for all phenotypes. Furthermore, we did not find a significant overall effect of sex on levels of TMEV RNA, though RNA sequencing of additional mice from each sex and strain will be necessary to properly evaluate sex effects.

In our study viral clearance and persistence do not reliably correlate with disease severity. Immune responses can prove both helpful and harmful. A strong response can clear the infection but cause irreparable damage to the CNS such as seen in TMEV-induced epilepsy fujinami^55,82,83^, or lead to autoimmunity, if not properly regulated^84,85^. In other cases, such as with enteroviruses, the virus itself is cytopathic^86^. Our findings showed that strains with little to no measurable viral RNA at 90 dpi continued to showed reduced evidence of disease progression. For these mice, it appears the appropriate immune response that cleared the virus was regulated properly, reducing or preventing progressive neurological disease phenotypes. Others with relatively high levels of TMEV RNA (CC006, CC011, CC015, and CC027) demonstrated reduced phenotypic signs of disease. These strains appear to be neither susceptible nor resistant to TMEV infection: they may be considered *tolerant* of the infection.

Of 18 strains studied, no 2 strains exhibited the exact same neurological sequelae following infection with TMEV. Furthermore, no 2 strains showed the same phenotypic responses as previously reported for strains of mice, such as SJL/J and C57BL/6. In fact, clinical signs of demyelination, as seen in TMEV-infected SJL/J mice^87^, have not been observed in any CC strains to date^45^. Several CC strains did exhibit seizure activity, typical for TMEV-infected C57BL/6 mice^35,88^, but the seizures were not recurrent or as severe. Instead, we identified previously unseen progression patterns and phenotypic subtypes for neurological outcomes of TMEV infection. For example, CC023 and CC057 mice demonstrated disease progression profiles and gait characteristics similar to PD, and, as in humans, these phenotypes were more significant in males than females. As gait abnormalities are among the first signs of PD^89^, these two strains are targets for future investigations into how viral infection can contribute to the onset of PD. DigiGait measurements for CC012xCC032 indicated a gait profile similar to that observed for PD and normal pressure hydrocephalus (NPH), two conditions which feature similar clinical signs in humans but can be distinguished from each other based on gait kinetics^90^. Several gait parameter measurements for CC012xCC032 mice, such as Paw Angle Variability, suggested a profile more similar to NPH than Parkinson’s. Importantly, TMEV can cause meningitis^38^, which can eventually lead to the onset of hydrocephalus and which is considered to be a contributing factor for NPH in humans^91^.

A unique and valuable contribution of this study is the detailed, multifaceted profiling of neurological diseases in mice of different sexes and genetic backgrounds. The integration of quantitative DigiGait analysis with other standard neurological phenotyping methods enabled us to identify subtle changes and differences between strains over time. Ultimately, some TMEV-infected CC strains (e.g. CC023, CC057, and CC012xCC032) may be valuable as novel models of neurological diseases (e.g. PD and NPH), giving researchers new tools for exploring gene-environment interactions underlying different disease subtypes.

## Supplementary information

Supplementary information.
