# Supplementary material for: Antecedent presentation of neurological phenotypes in the Collaborative Cross reveals four classes with complex sex-dependencies
Source: Sci Rep. 2020 May 13;10:7918. doi: 10.1038/s41598-020-64862-z (PMC7220920; doi:10.1038/s41598-020-64862-z)
Supplement: Supplementary file 1 — Supplementary information. [file 41598_2020_64862_MOESM1_ESM.pdf]

Supplementary information

Antecedent presentation of neurological phenotypes in the Collaborative Cross reveals four classes with complex sex-dependencies

Raena Eldridge[1#], Daniel Osorio[1#], Katia Amstalden[1], Caitlin Edwards[2], Colin R. Young[1], James J. Cai[1], Kranti Konganti[3], Andrew Hillhouse[3], David W. Threadgill[4], C. Jane Welsh[1,5], Candice Brinkmeyer-Langford[1*]

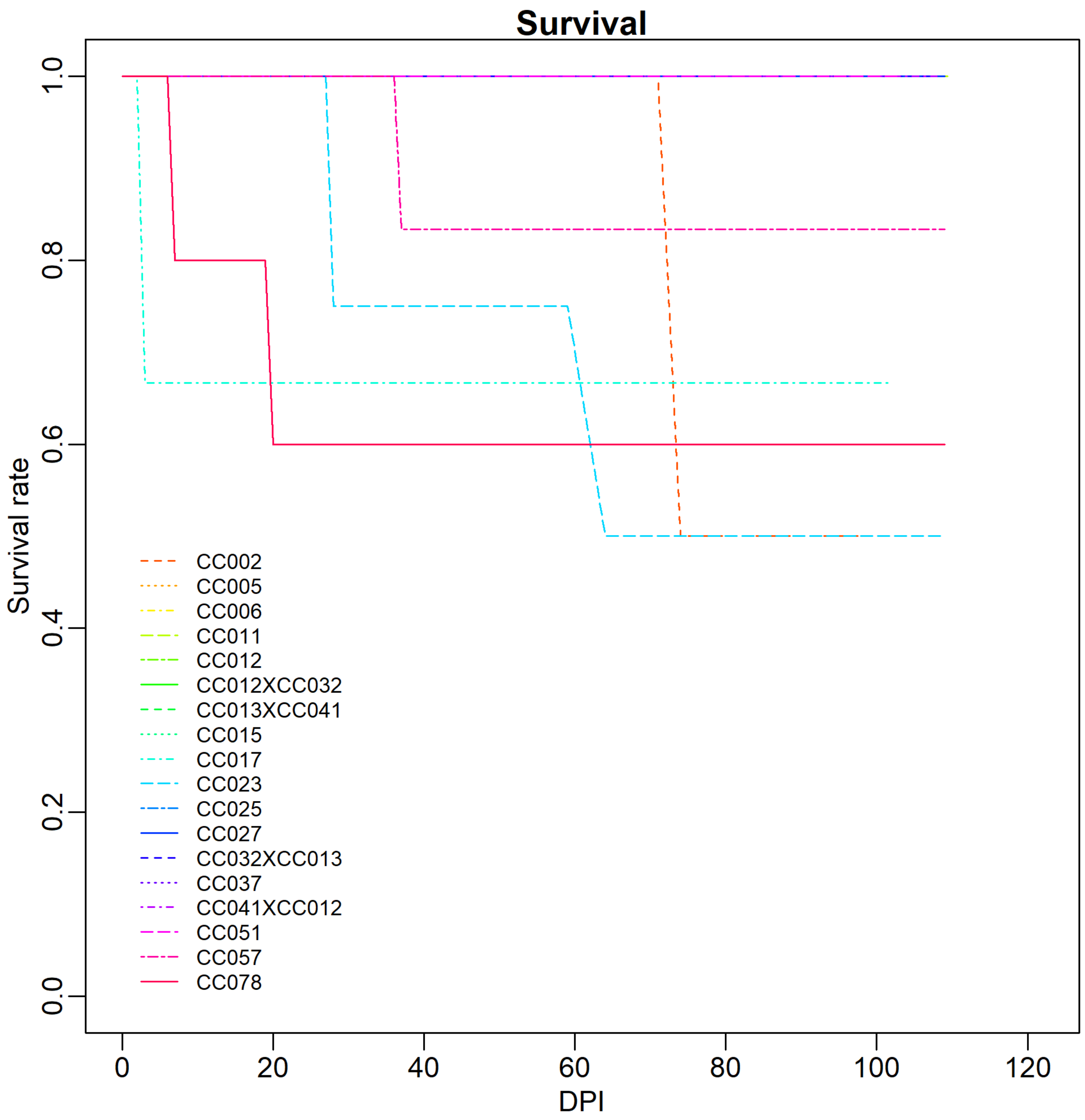

Figure S1. Survival rates varied by strain, and in comparison with the average of all strains, neither sex nor time point affected mortality. Deaths included 2 mice each from strains CC023 and CC078; a single mouse each died from strains CC002, CC017, and CC057. Refer to text for additional information about the sex and date of death for each mortality.

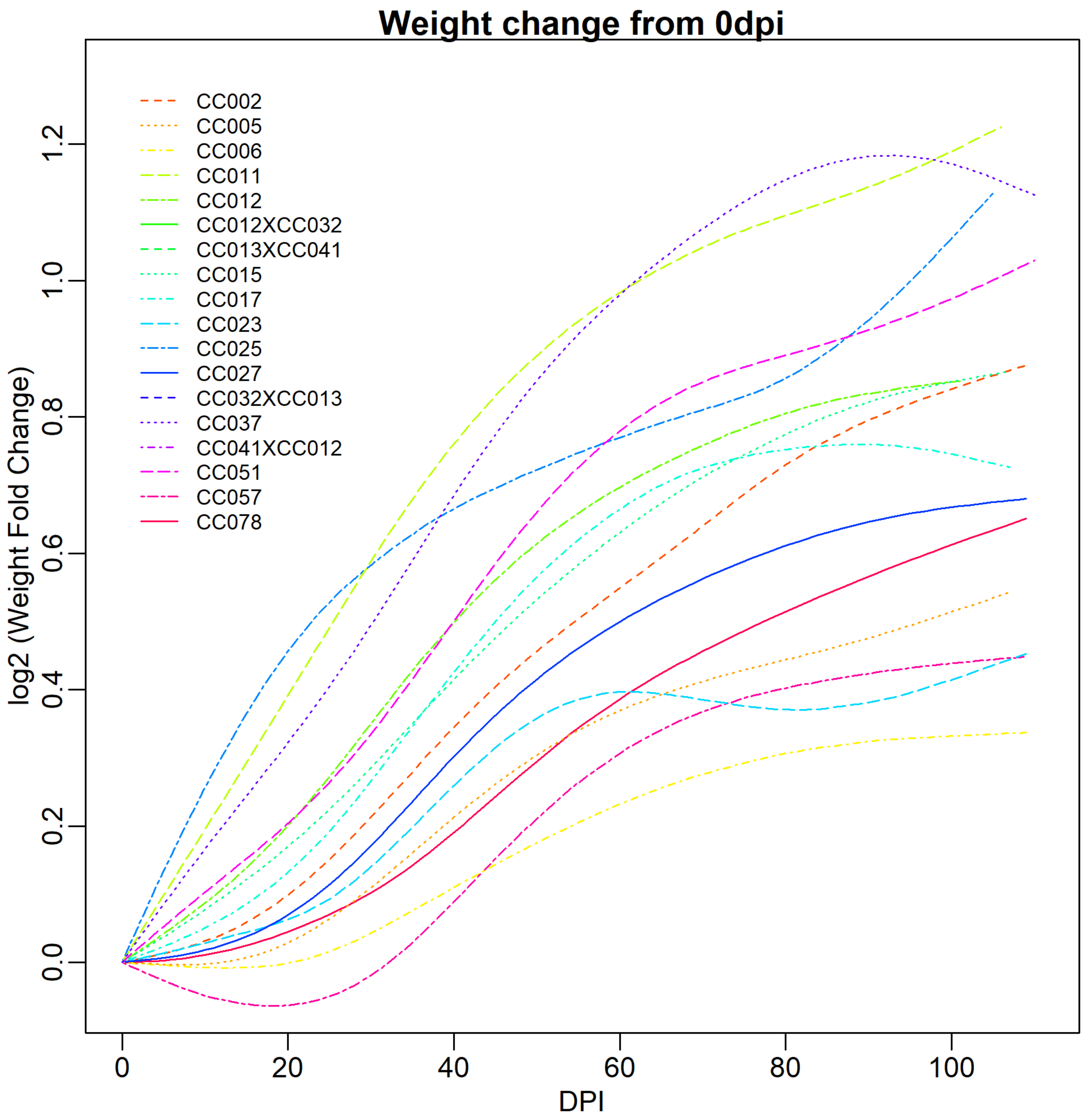

Figure S2. Average post-infection weight loss trends during the chronic phase of infection (35dpi and later).

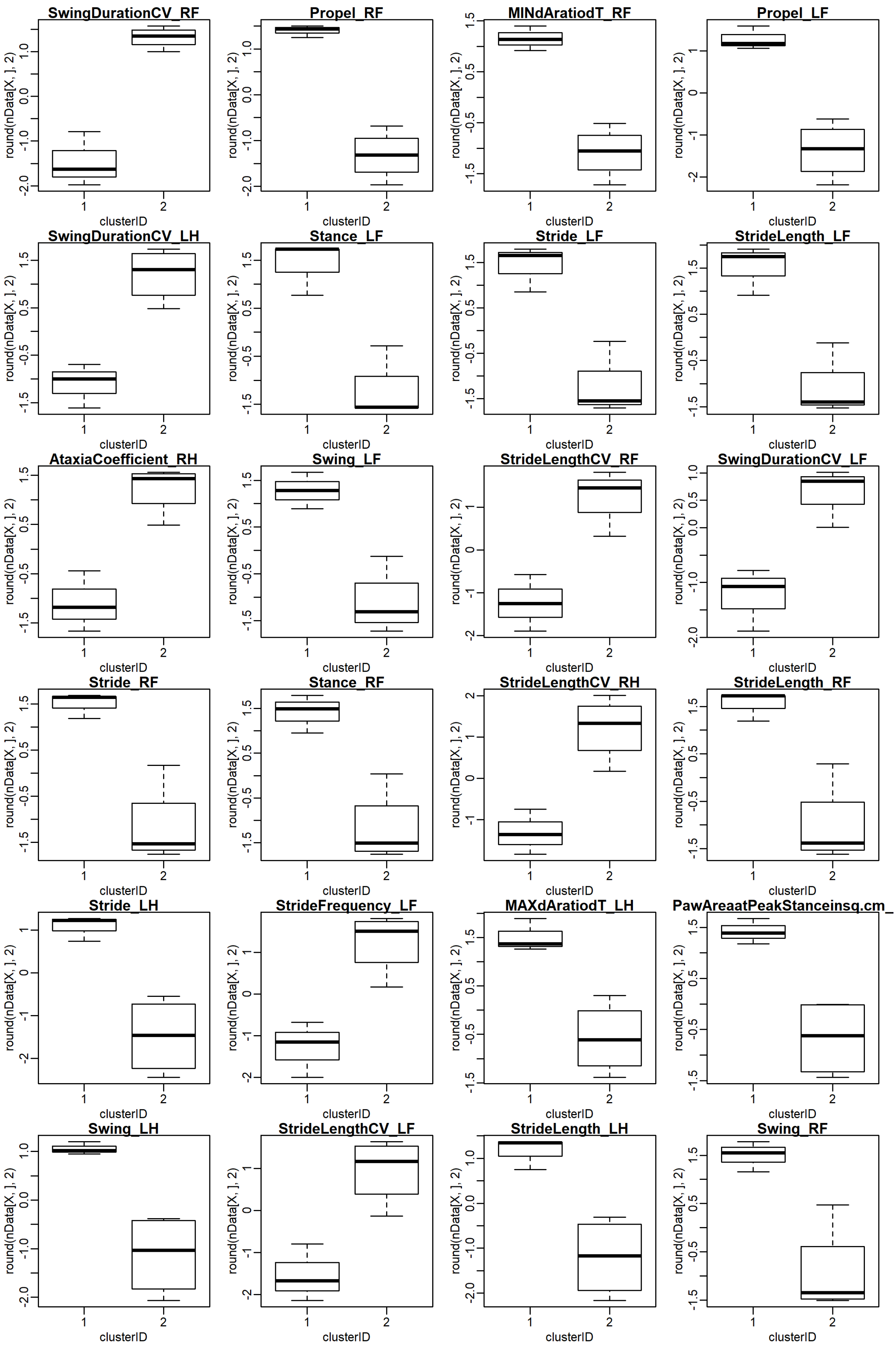

Figure S3. DigiGait parameters with significant differences between Cluster 1 and Cluster 2.

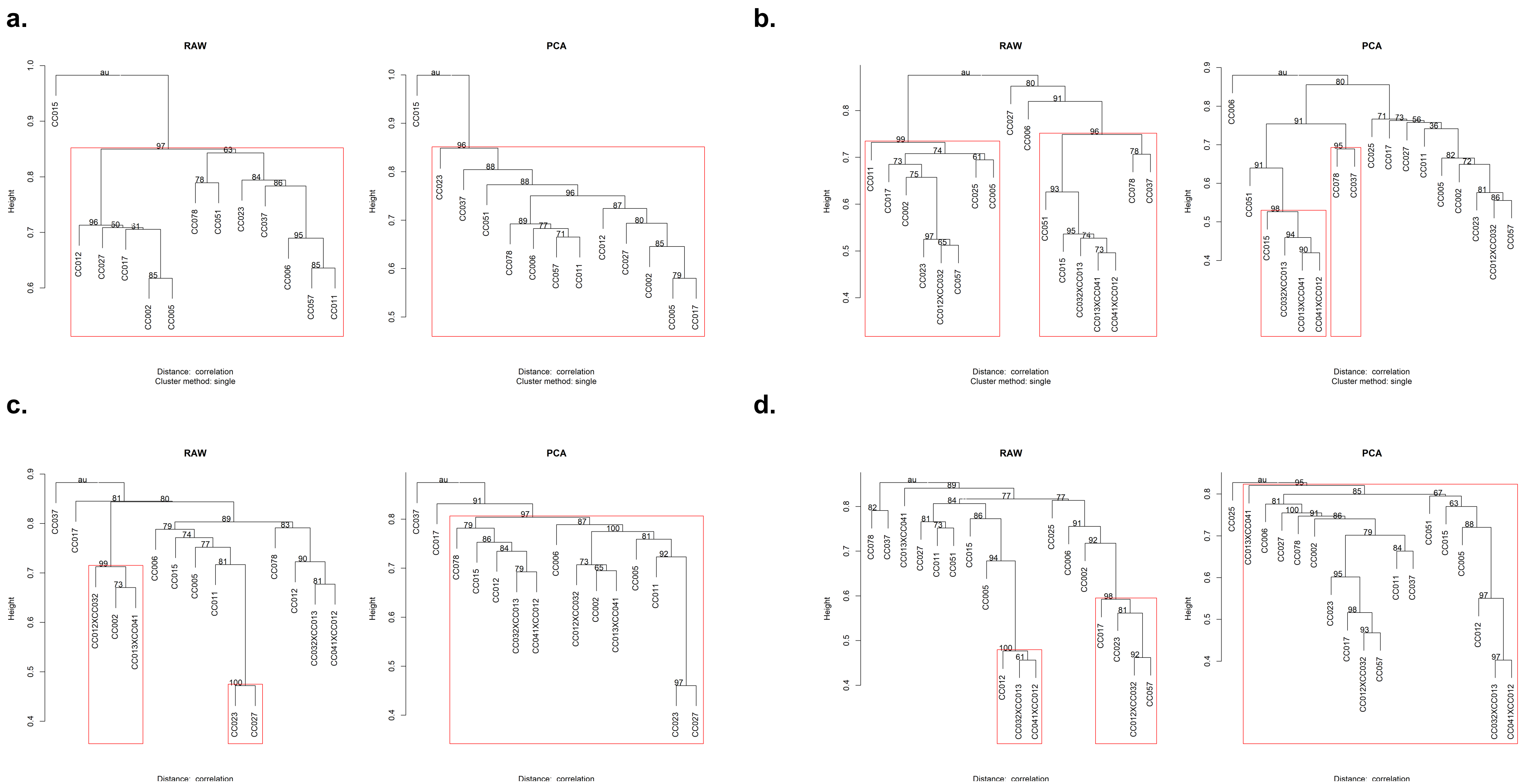

Figure S4. Hierarchical clustering analyses of DigiGait measurements, using all DigiGait parameters (RAW) and principal components only (PCA), illustrated differences in strain gait profiles over time. a: pre-infection; b: mid-infection (21dpi); c: late infection (89-90dpi); d: overall, including all time points together to summarize gait evolution throughout the experiment.

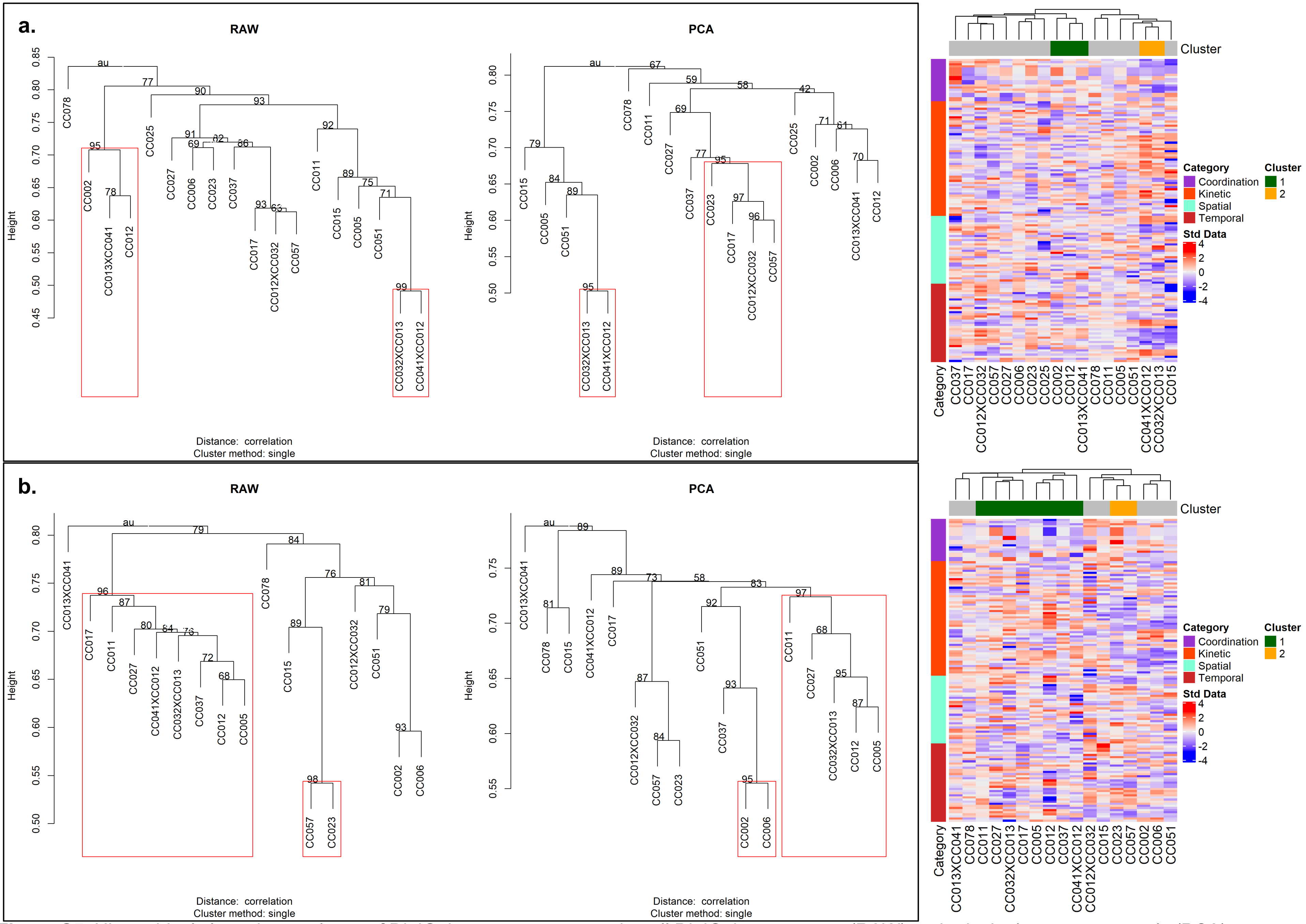

Figure S5. Hierarchical clustering analyses of DigiGait measurements, using all DigiGait parameters (RAW) and principal components only (PCA), illustrated differences in overall strain gait profiles between sexes. Heatmaps to the right of each also show categories for gait parameters as described in the text. a: female; b: male.

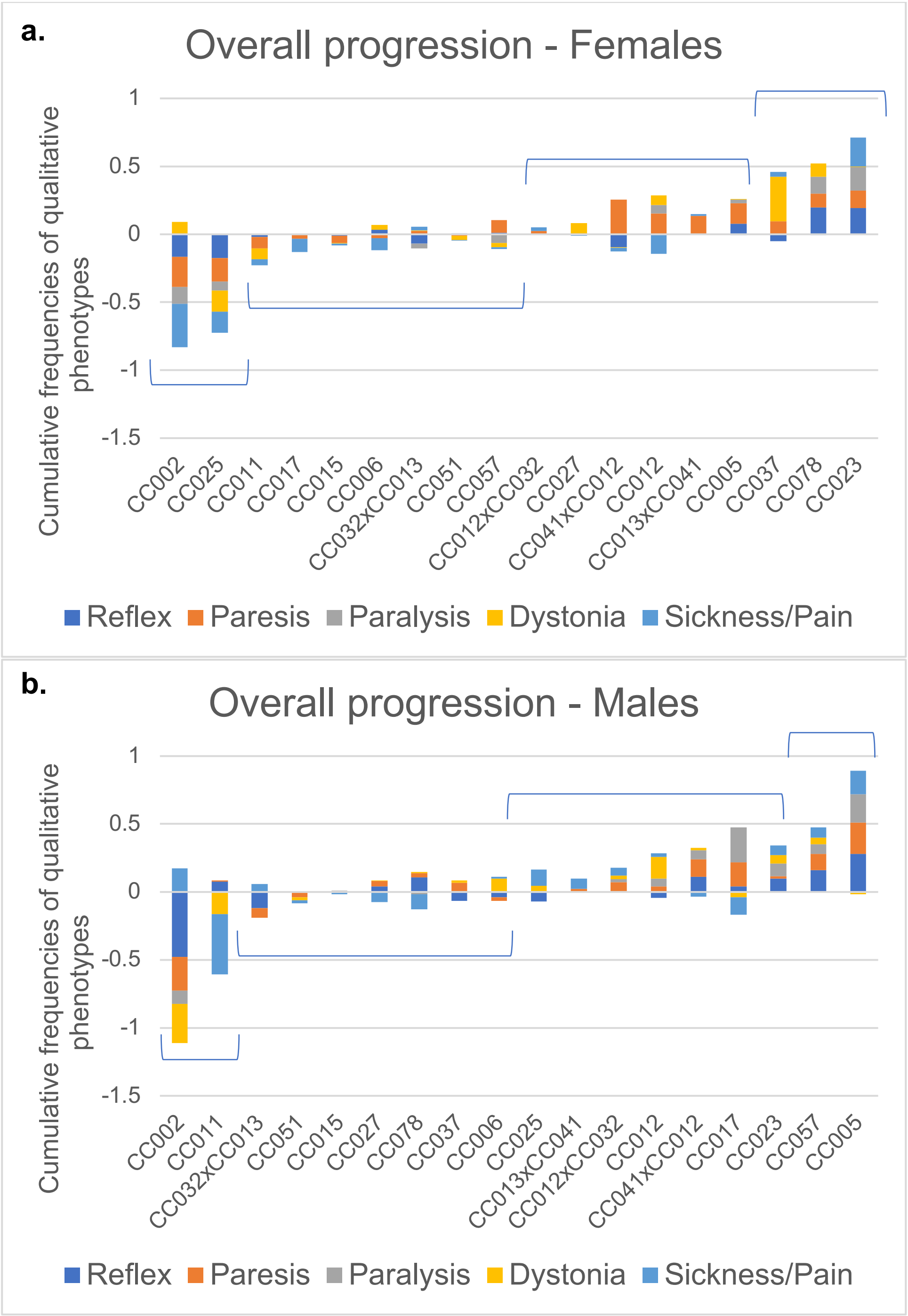

Figure S6. Cumulative frequencies of qualitative phenotypes varied by sex, resulting in different disease progression profiles for 8 strains. Strains included in each successive progression score are delineated by brackets.

Table S1

Strain	Sex	Infected	Hippocampus	Spinal cord
CC002	F	N		x
CC002	F	Y		x
CC002	M	N		x
CC002	M	Y	x	
CC002	M	Y		x
CC005	F	N	x	x
CC005	F	N	x	x
CC005	F	Y	x	x
CC005	F	Y	x	x
CC005	M	N	x	x
CC005	M	N	x	x
CC005	M	Y	x	x
CC005	M	Y	x	x
CC006	F	N		x
CC006	F	Y		x
CC006	F	Y	x	x
CC006	M	N	x	x
CC006	M	Y		x
CC006	M	Y	x	x
CC006	N	F	x	x
CC011	F	N	x	x
CC011	F	N	x	x
CC011	F	Y	x	x
CC011	F	Y	x	x
CC011	M	N	x	x
CC011	M	N	x	
CC011	M	Y	x	x
CC011	M	Y	x	x
CC015	F	N	x	x
CC015	F	Y	x	x
CC015	M	N	x	x
CC015	M	N	x	x
CC015	M	Y	x	x
CC015	M	Y	x	x
CC017	F	N	x	x
CC017	F	N	x	x
CC017	F	Y	x	x
CC017	M	N	x	x
CC017	M	N	x	x
CC017	M	Y	x	x
CC023	F	N		x
CC023	F	N	x	x
CC023	F	Y		x

Strain	Sex	Infected	Hippocampus	Spinal cord
CC023	F	Y	x	x
CC023	M	N		x
CC023	M	N	x	x
CC023	M	Y		x
CC023	M	Y	x	
CC025	F	N	x	
CC025	F	Y	x	x
CC025	M	Y	x	
CC027	F	N		x
CC027	F	N	x	x
CC027	F	Y		x
CC027	F	Y	x	x
CC027	M	N	x	x
CC027	M	N	x	x
CC027	M	Y		x
CC027	M	Y	x	x
CC032xCC013	F	N	x	
CC032xCC013	F	Y	x	
CC032xCC013	F	Y	x	
CC032xCC013	M	N	x	
CC032xCC013	M	Y	x	
CC032xCC013	M	Y	x	
CC032xCC013	M	Y	x	
CC037	F	N	x	x
CC037	F	Y	x	x
CC037	M	N	x	x
CC037	M	N	x	x
CC037	M	Y	x	x
CC037	M	Y	x	x
CC037	M	Y	x	x
CC037	M	Y	x	x
CC041xCC012	F	N	x	
CC041xCC012	F	Y	x	
CC041xCC012	F	Y	x	
CC041xCC012	F	Y	x	
CC041xCC012	M	N	x	
CC041xCC012	F	Y	x	
CC041xCC012	F	Y	x	
CC041xCC012	M	Y	x	
CC041xCC012	M	Y	x	
CC041xCC012	M	Y	x	
CC051	F	N	x	x
CC051	F	Y	x	x
CC051	F	Y	x	x

Strain	Sex	Infected	Hippocampus	Spinal cord
CC051	F	Y	x	x
CC051	M	N	x	x
CC051	M	Y	x	x

Table S1. To compare viral clearance across different CC strains, RNA sequencing was used to evaluate TMEV transcript levels in the hippocampus and/or spinal cord at 90dpi. "x" indicates that RNAseq data is available for these mice/tissues.

Table S2

Parameter	p.adj
SwingDurationCV_RF	***0.027082715***
Propel_RF	***0.027082715***
MINdAratiodT_RF	***0.041896791***
Propel_LF	***0.041896791***
SwingDurationCV_LH	***0.041896791***
Stance_LF	***0.041896791***
Stride_LF	***0.041896791***
StrideLength_LF	***0.041896791***
AtaxiaCoefficient_RH	***0.041896791***
Swing_LF	***0.044358044***
StrideLengthCV_RF	***0.045233112***
SwingDurationCV_LF	***0.045233112***
Stride_RF	***0.045233112***
Stance_RF	***0.045233112***
StrideLengthCV_RH	***0.045233112***
StrideLength_RF	***0.045233112***
Stride_LH	***0.045233112***
StrideFrequency_LF	***0.045233112***
MAXdAratiodT_LH	***0.045863973***
PawAreaatPeakStanceinsq.cm_LH	***0.045863973***
Swing_LH	***0.046920565***
StrideLengthCV_LF	***0.046920565***
StrideLength_LH	***0.047363274***
Swing_RF	***0.049881785***
StrideLengthCV_LH	0.053458453
AtaxiaCoefficient_RF	0.053458453
PawAngle_RF	0.053656036
PawAngleVariability_RF	0.05518387
SWVar_LF	0.05518387
StrideFrequency_RF	0.05518387
AbsolutePawAngle_RF	0.055487562
SwingDurationCV_RH	0.055487562
PawAreaVariabilityatPeakStanceinsq.cm_RF	0.055487562
AtaxiaCoefficient_LF	0.055487562
StrideFrequency_LH	0.059452936
StepAngleVar_LH	0.064599806
Stance_LH	0.067847979
MAXdAratiodT_RH	0.071323411
StanceWidthCV_LF	0.071323411
MidlineDistance_LH	0.071323411
pPropelStride_LF	0.071323411

Parameter	p.adj
PawAreaatPeakStanceinsq.cm_RH	0.072279995
Stride_RH	0.074137841
PawAngleVariability_LF	0.074137841
AtaxiaCoefficient_LH	0.078378389
MINdAratiodT_LF	0.081235815
StrideLength_RH	0.081235815
MidlineDistance_RH	0.082346556
PawAngleVariability_LH	0.084032685
PawAngleVariability_RH	0.084032685
AbsolutePawAngle_LF	0.089824634
AxisDistance_LF	0.102087721
PawDrag_RH	0.108366882
StrideFrequency_RH	0.111407031
pPropelStance_LF	0.112055995
pBrakeStance_LF	0.112055995
Brake_LH	0.114793944
SLVar_RH	0.114793944
pPropelStride_RF	0.114793944
GaitSymmetry_RH	0.114793944
GaitSymmetry_LH	0.114793944
GaitSymmetry_RF	0.114793944
GaitSymmetry_LF	0.114965569
SLVar_LH	0.117461484
pBrakeStride_LF	0.11896994
Swing_RH	0.124024597
Stance_RH	0.129286874
StepAngle_LH	0.13380209
PawAreaatPeakStanceinsq.cm_LF	0.166127171
StepAngleCV_LH	0.17902756
SWVar_LH	0.182308737
Brake_RF	0.183633431
PawAngle_LF	0.185701321
StanceWidthCV_LH	0.185701321
PawDrag_LH	0.185701321
nSteps_RF	0.187254469
MAXdAratiodT_LF	0.191104468
SLVar_RF	0.195701883
MAXdAratiodT_RF	0.206355255
nSteps_LF	0.217259324
PawAreaatPeakStanceinsq.cm_RF	0.22483255
Propel_RH	0.22483255
StanceFactor_LF	0.236881369

Parameter	p.adj
PawAreaVariabilityatPeakStanceinsq.cm_LH	0.239180927
SLVar_LF	0.252715892
HindLimbSharedStanceTime_LH	0.266308686
MidlineDistance_RF	0.266308686
pBrakeStance_RF	0.266308686
pPropelStance_RF	0.266308686
StepAngleVar_LF	0.271407717
pStanceStride_RF	0.304590874
pSwingStride_RF	0.304590874
Brake_LF	0.307962443
Propel_LH	0.307962443
AxisDistance_RH	0.339904057
nSteps_LH	0.361175968
StanceratioSwing_LF	0.388193875
StepAngleCV_LF	0.423396983
StanceFactor_LH	0.446331787
StanceratioSwing_RF	0.460537231
OverlapDistance_LF	0.465778083
OverlapDistance_LH	0.472138971
nSteps_RH	0.473527554
pBrakeStride_RF	0.508701472
pSharedStance_RH	0.533613907
pBrakeStride_LH	0.533613907
pBrakeStance_LH	0.533613907
pPropelStance_LH	0.533613907
MINdAratiodT_RH	0.60356501
pBrakeStride_RH	0.646161598
MidlineDistance_LF	0.646161598
StanceWidth_LF	0.652130465
pPropelStride_RH	0.667780905
pPropelStance_RH	0.667780905
pBrakeStance_RH	0.667780905
StanceWidth_LH	0.672426525
Brake_RH	0.683858812
pPropelStride_LH	0.701556432
AbsolutePawAngle_RH	0.701556432
PawPlacementPositioning[PPP]_LH	0.730767397
PawPlacementPositioning[PPP]_LF	0.730767397
PawAngle_RH	0.742406236
OverlapDistance_RH	0.744064991
OverlapDistance_RF	0.744064991
pStanceStride_RH	0.744064991

Parameter	p.adj
pSwingStride_RH	0.744064991
PawPlacementPositioning[PPP]_RF	0.744064991
PawPlacementPositioning[PPP]_RH	0.744064991
AbsolutePawAngle_LH	0.755941839
pSharedStance_LH	0.766863879
PawAreaVariabilityatPeakStanceinsq.cm_LF	0.805367164
MINdAratiodT_LH	0.806462903
StanceratioSwing_RH	0.890741107
StanceratioSwing_LH	0.892710696
PawAreaVariabilityatPeakStanceinsq.cm_RH	0.91158214
AxisDistance_LH	0.91158214
PawAngle_LH	0.968914647
StepAngle_LF	0.994014323
pStanceStride_LH	0.994014323
pSwingStride_LH	0.994014323
pSwingStride_LF	0.994014323
pStanceStride_LF	0.994014323
AxisDistance_RF	0.997961722

Table S2. P-adj values are listed for all DigiGait parameters for all limbs. LF indicates left fore limb, LH = left hind limb, RF = right fore limb, RH = right hind limb. Statistically significant values are shown in bold, italicized font.

Table S3

Trial#	Strain	Sex	Inf?	ID	#Steps_LF	#Steps_LH	#Steps_RF	#Steps_RH	%BrakeStance_LF	%BrakeStance_LH
1	CC002	F	N	1	35	31	28.5	29	30.8	45.6
1	CC025	F	N	2	19.5	19.5	21	22.5	51.3	46.4
1	CC012XCC032	F	N	10	21	14.5	19	16.5	43.3	17.7
2	CC012XCC032	F	N	10	6.5	5.5	5.5	5.5	37.3	42.8
1	CC012XCC032	M	N	11	12.5	8	10	9.5	40.4	5.1
2	CC012XCC032	M	N	11	5.5	6	5	6.5	61.3	9.7
1	CC013xCC041	F	N	12	44.5	54.5	54	54.5	42.5	38.8
2	CC013XCC041	F	N	12	6	6	5.5	5.5	55.1	17.6
1	CC013xCC041	M	N	13	10	6	6.5	12	46.8	26.8
2	CC013XCC041	M	N	13	7.5	6.5	7	6.5	44.6	24.3
1	CC032XCC013	F	N	25	14	10	19	15.5	54.7	22.2
2	CC032XCC013	F	N	25	13	12.5	13	10	49.7	30.4
1	CC041XCC012	F	N	26	15	16	10	14.5	50.6	25.5
1	CC041XCC012	F	N	26	23	23	20.5	16.5	50.7	29.7
2	CC041XCC012	F	N	26	10.5	6.5	12.5	9	38.6	39.3
1	CC032XCC013	M	N	27	16	14	15	17.5	64.7	25.3
2	CC032XCC013	M	N	27	7	6	8.5	7	33.7	40.9
1	CC041XCC012	M	N	28	15.5	14	23	18.5	65.1	32.1
2	CC041XCC012	M	N	28	9	8	9.5	7	68.9	18.6
0	CC012	F	N	51	9.5	7.5	8	7	28.2	14.6
2	CC012	F	N	51	7	6	10.5	7.5	58.8	25.7
0	CC012	M	N	52	10	12.5	9	8.5	46.5	9.2
2	CC012	M	N	52	12.5	7	16.5	4	47	23.7
0	CC057	F	N	63	6	5.5	6.5	8.5	43.1	37.7
1	CC057	F	N	63	6.5	6.5	6	6.5	35.3	27.8
0	CC057	M	N	64	10	7	6	7	42.1	47
1	CC057	M	N	64	6.5	6.5	6.5	6.5	31	25.6
0	CC078	F	N	65	7	7	7	7.5	27	9.6
1	CC078	F	N	65	7	7	7.5	6.5	52.7	9.2
2	CC078	F	N	65	22.5	16.5	27	19.5	45.1	9.6
0	CC078	M	N	66	8	7.5	7.5	7.5	18.5	9.8
2	CC078	M	N	66	23.5	21	25	22.5	31.2	31.6
0	CC002	F	N	78	13.5	22.5	17.5	29	43.4	28.6
1	CC002	F	N	78	35	31	28.5	29	30.8	45.6
0	CC002	M	N	80	14.5	11.5	11	18	46.8	22.6
1	CC002	M	N	80	17	15	11	10	53.6	49.2
2	CC002	M	N	80	7	5.5	8	9	72.9	15
0	CC006	F	N	82	7.5	7.5	7.5	7.5	82.1	3.8
1	CC006	F	N	82	12	8.5	9	6	64	23.5
2	CC006	F	N	82	13.5	9.5	8	11.5	57.3	41.5
0	CC006	M	N	84	13	14.5	10	26	53.3	43.4
0	CC023	F	N	86	9	12.5	9	9	45.4	48.5
1	CC023	F	N	86	41	33.5	47.5	35	41.9	16.1
2	CC023	F	N	86	6	6	10	6.5	45.5	37.3
0	CC023	M	N	88	6	6.5	6.5	6.5	24.5	12.2
1	CC023	M	N	88	11	10	10	10.5	31.3	22.3
0	CC027	F	N	90	11	12.5	20	17	40	36.8

Trial#	Strain	Sex	Inf?	ID	#Steps_LF	#Steps_LH	#Steps_RF	#Steps_RH	%BrakeStance_LF	%BrakeStance_LH
1	CC027	F	N	90	9	11.5	11.5	9	47.1	66.7
2	CC027	F	N	90	10	8	9.5	11.5	25.8	30.5
0	CC027	M	N	92	12	24	12.5	12	30.1	35.3
1	CC027	M	N	92	9	8.5	9	11.5	34.7	24.1
1	CC027	M	N	92	6.5	12	10	14	54.4	62.1
2	CC027	M	N	92	3.5	3.5	4	4	47	23.3
0	CC005	F	N	94	9.5	6.5	6.5	7	43.1	19.9
1	CC005	F	N	94	10.5	9	9	10	52.3	74.5
2	CC005	F	N	94	23	28.5	21	28	63.9	53.4
0	CC011	F	N	95	10.5	8	10	8	46.9	44.2
1	CC011	F	N	95	36.5	24	41.5	27.5	32.9	5.2
2	CC011	F	N	95	11.5	10.5	12	11	35.6	12.7
1	CC017	F	N	96	16	11.5	12	12.5	47.3	25.1
0	CC005	M	N	99	5	6	5.5	5.5	55.7	17.7
1	CC005	M	N	99	14.5	17.5	12	9.5	57.1	22.8
2	CC005	M	N	99	54.5	52	51.5	42.5	49.1	21
0	CC011	M	N	100	6.5	6	5.5	5.5	35.6	20.3
1	CC011	M	N	100	7	6.5	7	7	46.2	29.7
2	CC011	M	N	100	38	34.5	47	39.5	51.3	17
0	CC017	M	N	101	19	31	26.5	27.5	32.8	46.1
1	CC017	M	N	101	8	5	4	7	42.4	18.6
1	CC006	F	N	114	24	30.5	27.5	23	70.5	49.1
0	CC037	F	N	115	16	15.5	17.5	15.5	43.3	27.4
0	CC051	F	N	116	24	15	23	16	31.5	20.1
1	CC051	F	N	116	9	9	6	6.5	32.2	27.1
1	CC006	M	N	117	12	10	10	9.5	48.5	24.3
0	CC037	M	N	118	20	15.5	16	15.5	45.3	42.2
1	CC037	M	N	118	4.5	6	6	6.5	50.1	19
0	CC005	F	N	126	11	9	10	9	52.5	21.6
1	CC005	F	N	126	8	7	7.5	6.5	41.5	27.2
2	CC005	F	N	126	6.5	6.5	12.5	6.5	52.2	26.8
0	CC011	F	N	127	11	10.5	11	11.5	34.8	13.4
1	CC011	F	N	127	7.5	7.5	7.5	7.5	31.8	28.2
2	CC011	F	N	127	6	6.5	6.5	6.5	48	50.4
0	CC011	M	N	128	7.5	5.5	7.5	7.5	52.2	28.4
1	CC011	M	N	128	6	6	5.5	6	46.3	37.8
2	CC011	M	N	128	6.5	6.5	6	6.5	58	39.6
1	CC037	M	N	129	7	7.5	11.5	8	54.2	31.8
2	CC037	M	N	129	10.5	9.5	12	8	28.5	30.4
1	CC051	M	N	130	5.5	6	6	5.5	33.1	34.5
2	CC051	M	N	130	7.5	6.5	7	6	48.1	44.9
0	CC027	F	N	137	6.5	6.5	6.5	7	35.8	17.8
1	CC027	F	N	137	7	8.5	8.5	9	44.1	35.9
2	CC027	F	N	137	50	45.5	45.5	42.5	57.6	32.3
0	CC015	M	N	142	6.5	6	6.5	6	31.5	32
1	CC015	M	N	142	9	8.5	11	12.5	49	30.9
2	CC015	M	N	142	7.5	7	8.5	7.5	40.3	34.8
0	CC027	M	N	144	7.5	7	6	6	47.3	21.6

Trial#	Strain	Sex	Inf?	ID	#Steps_LF	#Steps_LH	#Steps_RF	#Steps_RH	%BrakeStance_LF	%BrakeStance_LH
1	CC027	M	N	144	9.5	8	7.5	10	45.7	25.8
2	CC027	M	N	144	9	11	6.5	8.5	44	24.3
0	CC015	F	N	160	7.5	7.5	7.5	7	29.7	43.5
1	CC015	F	N	160	75	71	75	79.5	56	44
0	CC017	F	N	161	11.5	10.5	11.5	10	53.1	30.8
0	CC023	F	N	162	8	8	8	8	38.9	58.4
1	CC023	F	N	162	8.5	9	10.5	8.5	46.6	34.1
0	CC005	M	N	163	6	8.5	7	6.5	44.2	15.4
1	CC005	M	N	163	6	5.5	5.5	5.5	74.8	10.2
0	CC015	M	N	164	9.5	9.5	10	10	44.8	21.8
1	CC015	M	N	164	10.5	8	17	21.5	40.4	39.5
0	CC017	M	N	165	8.5	9.5	10	9	36.8	50.5
1	CC017	M	N	165	19.5	21.5	21.5	16.5	60.9	27.5
0	CC023	M	N	166	8.5	8.5	8	8	48.4	22.7
1	CC023	M	N	166	11	8.5	8.5	9	58.5	19
0	CC051	M	N	168	9	8	9	9	66.9	14.6
0	CC002	F	Y	79	14	13.5	15	16.5	52.9	52.2
0	CC002	M	Y	81	12.5	6.5	12.5	9	50.5	37.1
1	CC002	M	Y	5	18	13.5	23	24.5	38.5	52.5
1	CC002	F	Y	79	34	5	25.5	17	36.1	71
1	CC002	F	Y	79	34	22.5	28.5	26	45.6	37.7
1	CC002	M	Y	81	18	13.5	23	24.5	38.5	52.5
1	CC002	M	Y	81	17	18	17.5	23.5	45.8	43.7
1	CC002	F	Y	10000	34	5	25.5	17	36.1	71
1	CC002	F	Y	10000	32	65.5	45	63.5	32.9	39.8
2	CC002	M	Y	5	9.5	5.5	16.5	7	36.2	27.1
2	CC002	F	Y	79	8.5	9	8.5	8.5	40.9	30.7
2	CC002	M	Y	81	11	6.5	14.5	11.5	29.1	14.2
0	CC005	F	Y	104	8.5	7	8	7	42.3	28.7
0	CC005	M	Y	109	12	8.5	9.5	9	37.4	10.5
0	CC005	M	Y	170	8	7	8	7.5	73.6	21.1
1	CC005	F	Y	104	21.5	10.5	15.5	12	51.4	14.3
1	CC005	M	Y	109	7	10	12	16.5	13.8	25.4
1	CC005	F	Y	131	5.5	5.5	6	6	55.5	25.2
1	CC005	M	Y	170	9.5	10.5	5.5	7.5	60.7	31.1
2	CC005	F	Y	104	16.5	12.5	15.5	14.5	70.1	33.5
2	CC005	M	Y	109	12	8.5	8.5	8.5	53.4	43.9
2	CC005	M	Y	109	10	10.5	13	15	60.9	18.4
2	CC005	M	Y	109	12	8.5	8.5	8.5	53.4	43.9
2	CC005	F	Y	131	7.5	5.5	6.5	9	72.5	21.1
0	CC006	F	Y	83	10	8.5	10.5	8.5	54.3	22.3
0	CC006	M	Y	85	11.5	14	18	22	39.8	28.3
0	CC006	F	Y	119	53.5	51	55.5	50.5	42.9	24.3
1	CC006	F	Y	83	21	20.5	20	17.5	52.6	37.2
1	CC006	M	Y	85	21	25	19.5	19.5	49.4	41.5
1	CC006	F	Y	119	8.5	8.5	8.5	9	55.4	10
1	CC006	M	Y	124	8.5	7.5	8.5	8	33.7	29
2	CC006	F	Y	83	9.5	9	8.5	9.5	48.7	12.2

Trial#	Strain	Sex	Inf?	ID	#Steps_LF	#Steps_LH	#Steps_RF	#Steps_RH	%BrakeStance_LF	%BrakeStance_LH
2	CC006	M	Y	85	19	20.5	12	15.5	63.9	33.8
0	CC011	F	Y	105	14	10	16.5	7.5	28.8	5.2
0	CC011	M	Y	110	10	10.5	11	11	30.8	25
0	CC011	F	Y	132	7.5	6	6	6.5	35.1	20.1
0	CC011	F	Y	132	9	10.5	9	14.5	52.5	44.7
1	CC011	F	Y	105	9.5	8	9.5	8	43.3	30.5
1	CC011	M	Y	110	6	5.5	5.5	5	57.2	31.5
1	CC011	F	Y	132	7.5	7	7	7.5	56.1	39.6
1	CC011	M	Y	133	7	7	7.5	6	60.4	41.3
2	CC011	F	Y	105	7	6.5	6.5	7	23.6	29.1
2	CC011	M	Y	110	10.5	7.5	13.5	6.5	36	16.5
2	CC011	F	Y	132	6	6.5	6	6.5	48.3	33.1
2	CC011	M	Y	133	6.5	6	5.5	6	65.1	34.6
0	CC012	M	Y	53	7.5	6.5	7	8.5	30.9	17.7
0	CC012	M	Y	54	5.5	7.5	7.5	7.5	40	39.1
0	CC012	M	Y	55	7.5	6.5	6.5	7.5	33.7	16.2
0	CC012	M	Y	56	31	29.5	33.5	34.5	26.3	13.6
0	CC012	M	Y	57	7	7	7.5	9	41.8	20.7
0	CC012	F	Y	58	11	8	9.5	6.5	37.6	25.3
0	CC012	F	Y	59	7.5	7	7.5	7.5	28.2	26.1
0	CC012	F	Y	60	47	37	28.5	32.5	45.1	29.6
0	CC012	F	Y	61	34	38	41	35.5	58.1	27.3
0	CC012	F	Y	62	5.5	4.5	5	5.5	31.4	15
2	CC012	M	Y	53	6.5	6.5	7.5	6	60.3	7.1
2	CC012	M	Y	54	4.5	5.5	5	5.5	46.2	17.7
2	CC012	M	Y	55	4.5	6	6	6.5	21.7	12.4
2	CC012	M	Y	56	7	11	6	11.5	31.5	17.8
2	CC012	M	Y	57	7	7	7.5	7	55.6	27.6
2	CC012	F	Y	58	6.5	5	4.5	4.5	35.1	48.6
2	CC012	F	Y	59	7.5	4.5	7	6.5	67.8	35.6
2	CC012	F	Y	60	27	7	28.5	27	30.1	39.3
2	CC012	F	Y	61	5.5	4.5	7.5	4.5	31.9	21.1
2	CC012	F	Y	62	9.5	7.5	6	8	36.9	39.2
1	CC012XCC032	F	Y	14	46	60.5	50	68.5	32.3	44.8
1	CC012xCC032	F	Y	15	36	38	25	36.5	52.3	44
1	CC012XCC032	M	Y	16	23	19.5	20	19	53.2	32
1	CC012xCC032	M	Y	17	22.5	17.5	17.5	17.5	48	17.8
1	CC012XCC032	M	Y	18	13.5	7	9	15	55	12.2
1	CC012xCC032	M	Y	19	10.5	10.5	13	18	37	54.3
2	CC012XCC032	F	Y	14	9.5	7.5	11	6.5	66.4	41.8
2	CC012XCC032	F	Y	15	7	7	6	6.5	54.2	35.2
2	CC012XCC032	M	Y	16	10.5	6	14	9.5	40.6	29.9
2	CC012XCC032	M	Y	17	13.5	8	9	6.5	52	27.4
2	CC012XCC032	M	Y	18	6	7.5	6	7	62.4	35.3
2	CC012XCC032	M	Y	19	7.5	5.5	8	5	64.4	7.4
1	CC013xCC041	F	Y	20	26	23	16.5	28.5	49.6	18.3
1	CC013xCC041	F	Y	21	41	54.5	39.5	46	52.9	35.1
1	CC013xCC041	F	Y	22	40	30	38.5	32.5	40.4	31.2

Trial#	Strain	Sex	Inf?	ID	#Steps_LF	#Steps_LH	#Steps_RF	#Steps_RH	%BrakeStance_LF	%BrakeStance_LH
1	CC013xCC041	M	Y	23	8.5	8	9.5	8	52.3	11.4
1	CC013xCC041	M	Y	24	8	5	10.5	12.5	38.8	28.5
2	CC013XCC041	F	Y	20	6.5	6	6.5	6.5	44.5	36.6
2	CC013XCC041	F	Y	21	8	7.5	8	5	52.8	44.3
2	CC013XCC041	F	Y	22	7.5	7	6.5	6.5	72.7	14.7
2	CC013XCC041	M	Y	23	14	8.5	11.5	10.5	64.1	32.2
2	CC013XCC041	M	Y	24	18	10.5	18.5	18.5	45.1	12.7
0	CC015	F	Y	145	6	7.5	6.5	7	56.4	35
0	CC015	M	Y	171	7.5	7.5	7.5	8	52.4	29.6
0	CC015	M	Y	172	7.5	8	9	9	49.6	44.6
1	CC015	F	Y	145	6.5	7	7	8	41.6	33.9
1	CC015	M	Y	171	27	30.5	23.5	26	48.7	45.1
1	CC015	M	Y	172	15.5	20.5	13	18.5	51.3	54.6
1	CC015	M	Y	175	64.5	52.5	76.5	65	62.6	33.9
2	CC015	F	Y	145	8	6.5	6.5	7	53.3	51.2
0	CC017	F	Y	106	8	8	9	9.5	31.7	25.7
0	CC017	M	Y	111	8.5	12	8	10	39.2	11.3
0	CC017	F	Y	169	12.5	8.5	14	10	52.3	26.3
0	CC017	M	Y	173	12	9	17.5	11	66.4	35.7
1	CC017	M	Y	111	9	8	6.5	7	56.4	27
1	CC017	F	Y	169	11.5	11	9.5	12	50.6	29.6
0	CC023	M	Y	87	5.5	6	5.5	5.5	14.4	12.4
0	CC023	F	Y	89	8	6.5	8	14	31	16.4
0	CC023	F	Y	146	9	10.5	11.5	9	44.7	41.6
0	CC023	M	Y	152	8.5	4.5	9	4.5	56.4	12.3
1	CC023	M	Y	87	31.5	20.5	28.5	15.5	38.6	22
1	CC023	F	Y	89	26.5	20	29.5	15.5	46.8	18.6
1	CC023	M	Y	152	9.5	6	12	7.5	50.8	29.5
1	CC023	M	Y	152	4	4	4	3.5	45.7	32.2
2	CC023	M	Y	87	5	5	4.5	4.5	45.4	13.6
2	CC023	F	Y	89	5.5	5	6.5	4.5	60.8	16.7
2	CC023	M	Y	152	16.5	7	13	11.5	41.8	11.9
0	CC025	M	Y	7	29	27.5	28.5	28.5	28	36.9
0	CC025	F	Y	148	11.5	9.5	9.5	10	37	35.4
0	CC025	M	Y	154	3.5	4	5	3.5	46.5	7.5
1	CC025	M	Y	7	29	27.5	29	28	26.3	32.1
1	CC025	F	Y	8	33	37	34.5	36.5	38.9	24.4
1	CC025	F	Y	9	22.5	17	15.5	14.5	42.1	39.2
1	CC025	F	Y	148	11	8.5	11.5	11	27	26.9
2	CC025	F	Y	148	9	8.5	8.5	8.5	59	27.8
2	CC025	M	Y	154	8	7	7.5	7.5	40.9	35.7
0	CC027	F	Y	149	6.5	7.5	8.5	6.5	53.1	17.7
0	CC027	M	Y	155	13	8.5	15	6	36.9	11.4
1	CC027	M	Y	91	2.5	2	3	3.5	28.2	53.3
1	CC027	M	Y	91	2.5	2	3	3.5	28.2	53.3
1	CC027	F	Y	93	19.5	18	20	27	39.7	24.6
1	CC027	F	Y	149	17	15.5	21.5	26	49.3	43.8
1	CC027	M	Y	155	4.5	3	2.5	2.5	49.4	50.3

Trial#	Strain	Sex	Inf?	ID	#Steps_LF	#Steps_LH	#Steps_RF	#Steps_RH	%BrakeStance_LF	%BrakeStance_LH
1	CC027	M	Y	155	5.5	5.5	5.5	5	55.9	24.5
2	CC027	F	Y	89	5.5	5	6.5	4.5	60.8	16.7
2	CC027	M	Y	91	8	10	6	5.5	39.8	32.9
2	CC027	F	Y	149	18.5	14.5	23.5	18	47	42
2	CC027	M	Y	155	10.5	8	8	8.5	56	21.2
1	CC032XCC013	M	Y	30	9.5	8.5	12	8	70.7	37.4
1	CC032XCC013	M	Y	31	13.5	13	13.5	13	52.2	29.9
1	CC032XCC013	M	Y	32	15.5	11.5	11.5	11.5	52.5	31.8
1	CC032XCC013	F	Y	33	18	13	6.5	12.5	40.7	37.8
1	CC032XCC013	F	Y	34	11	7	9.5	7.5	49.6	39.4
1	CC032XCC013	F	Y	35	9	6.5	7.5	6.5	63.1	34.3
1	CC032XCC013	F	Y	35	5.5	5.5	6.5	5.5	53.5	33.5
1	CC032XCC013	F	Y	36	5.5	5.5	5	5.5	39	52.1
2	CC032XCC013	M	Y	29	5	4.5	4.5	4.5	34.4	37.1
2	CC032XCC013	M	Y	30	6.5	5	9.5	7.5	59.6	14.7
2	CC032XCC013	M	Y	31	15	8.5	18	11	38.7	29
2	CC032XCC013	M	Y	32	7.5	6.5	14.5	9.5	52.1	42.6
2	CC032XCC013	F	Y	33	7.5	7.5	8	7	48.2	43.1
2	CC032XCC013	F	Y	34	11	8	9	8	62.7	45.5
2	CC032XCC013	F	Y	35	8.5	7.5	10.5	7	48.3	42.6
2	CC032XCC013	F	Y	36	9	7.5	9	7.5	45.4	29.3
0	CC037	F	Y	120	32	24.5	30	20	43.4	24.3
0	CC037	M	Y	125	43	29	40.5	23	39.7	26.6
0	CC037	M	Y	157	11.5	6	9	5.5	43.1	24.2
0	CC037	M	Y	158	5.5	6.5	10.5	10.5	47.7	25.7
1	CC037	F	Y	120	11.5	9.5	10	9	53.4	49.6
1	CC037	M	Y	125	11	10.5	10	10	40.5	24.5
1	CC037	M	Y	134	10.5	7.5	10.5	8	57.6	39.2
1	CC037	M	Y	158	7	7	7.5	9	38.3	42.2
2	CC037	M	Y	134	8	6	9.5	6	68.8	50
2	CC037	M	Y	158	8.5	8.5	9	9	30.9	34.5
1	CC041XCC012	M	Y	37	11.5	16	14.5	11	59.9	17.2
1	CC041XCC012	M	Y	38	17	7	7.5	7	58.9	24
1	CC041XCC012	M	Y	39	7.5	19	17.5	22.5	55	29.9
1	CC041XCC012	M	Y	40	22.5	14.5	12.5	15.5	61.2	32.4
1	CC041XCC012	M	Y	41	11	7	7.5	7	50.4	34.4
1	CC041XCC012	F	Y	43	12	8.5	12.5	10	55.2	45.7
1	CC041XCC012	F	Y	44	6	5.5	10.5	7	29.8	30.3
1	CC041XCC012	F	Y	45	7	6	8	6.5	46.2	21.9
1	CC041XCC012	F	Y	46	7.5	6	7	6	67.3	55.2
1	CC041XCC012	F	Y	47	7	8.5	12	8.5	48.9	23
1	CC041XCC012	F	Y	48	9	6	7	6	44.5	52.7
1	CC041XCC012	F	Y	50	5.5	5	5	4.5	53.8	35.3
1	CC041XCC012	F	Y	50	5	9	14	10.5	41.3	22.4
2	CC041XCC012	M	Y	37	8	5.5	6	9.5	50.5	34.8
2	CC041XCC012	M	Y	38	8	7.5	9.5	8	57.8	31.8
2	CC041XCC012	F	Y	39	6.5	7.5	8.5	7	45.5	27.1
2	CC041XCC012	M	Y	40	10	8.5	7.5	6.5	58.1	30.8

Trial#	Strain	Sex	Inf?	ID	#Steps_LF	#Steps_LH	#Steps_RF	#Steps_RH	%BrakeStance_LF	%BrakeStance_LH
2	CC041XCC012	M	Y	41	7.5	7.5	8	8	45.6	29.5
2	CC041XCC012	M	Y	42	11	10	10	8.5	42.1	10.1
2	CC041XCC012	F	Y	43	7	7	6.5	6.5	54	41.8
2	CC041XCC012	F	Y	44	9	8.5	8.5	8.5	54.6	23
2	CC041XCC012	F	Y	45	7.5	7.5	8.5	7	45.6	42.6
2	CC041XCC012	F	Y	46	8.5	8.5	8	8.5	43.2	35.2
2	CC041XCC012	F	Y	47	7	6	6.5	6	42.4	40.3
2	CC041XCC012	F	Y	48	6.5	6.5	6.5	7	52.9	27.9
2	CC041XCC012	F	Y	50	10.5	10.5	11.5	10.5	44.7	14.5
0	CC051	F	Y	121	23	20.5	24.5	21	44.7	36.9
0	CC051	F	Y	122	22	16	24	17.5	33.4	22.4
0	CC051	F	Y	123	16	15.5	15	14.5	38.2	21.7
0	CC051	M	Y	175	8	8.5	9	8	47.5	27.8
1	CC051	F	Y	121	12.5	10.5	10.5	10.5	42.9	35.6
1	CC051	F	Y	122	8	8	9.5	8.5	40.5	37.7
1	CC051	F	Y	123	9	7	7.5	8	45.8	32.8
0	CC057	F	Y	67	8	7.5	7.5	7.5	46	36.9
0	CC057	F	Y	68	11.5	10.5	10	10.5	47.5	22.3
0	CC057	F	Y	69	12.5	12	13	15	51.6	40.2
0	CC057	M	Y	70	19.5	9.5	8	8.5	46.1	45.8
0	CC057	M	Y	71	9	19	12	20.5	55.9	57.6
0	CC057	M	Y	72	14	15	19.5	18	62.3	28.8
1	CC057	F	Y	67	6	6	6.5	6.5	45.9	10.1
1	CC057	F	Y	68	14.5	14	14.5	14	42.7	29.5
1	CC057	F	Y	69	17.5	17	21	18	43.7	20.7
1	CC057	F	Y	69	28.5	29.5	30.5	28.5	58.8	26.6
1	CC057	M	Y	70	8.5	7.5	8	6.5	58.2	6.4
1	CC057	M	Y	72	6.5	6.5	7	6	51.4	35.1
2	CC057	F	Y	69	28.5	29.5	30.5	28.5	58.8	26.6
0	CC078	F	Y	73	7	7	8	9	36.3	8.2
0	CC078	F	Y	74	7.5	9.5	8	9	29.6	37.4
0	CC078	F	Y	75	6.5	8	8	6.5	38.5	17.5
0	CC078	M	Y	76	7.5	7.5	7.5	7.5	38.9	22.3
1	CC078	F	Y	74	9	8	7.5	7.5	34.2	24.8
1	CC078	F	Y	75	10	9	9.5	9.5	47.4	10.5
1	CC078	M	Y	76	7	7	7.5	7	54.3	28.5
2	CC078	F	Y	74	20.5	22	26.5	24.5	21.4	33.8
2	CC078	M	Y	76	31.5	26	30.5	27	61.8	21.8
2	CC005	M	N	163	26	22	16.5	27	61.5	20
2	CC005	M	Y	170	79.5	69.5	88.5	58	46	22.2
2	CC015	F	N	160	24.5	18.5	27.5	18	53.3	38.9
2	CC015	M	N	164	9	7.5	8	8.5	61.8	35.1
2	CC015	M	Y	171	12	10.5	11.5	11.5	62.8	42.3
2	CC015	M	Y	172	21	17	22	20	51	53.2
2	CC017	F	N	161	10.5	8.5	16	9.5	57.8	37.4
2	CC017	M	N	165	7.5	5.5	9	4.5	42	31.2
2	CC017	F	Y	169	32.5	28.5	23.5	26.5	73.6	17
2	CC023	F	N	162	14	13.5	11	12	69.1	36.7

Trial#	Strain	Sex	Inf?	ID	#Steps_LF	#Steps_LH	#Steps_RF	#Steps_RH	%BrakeStance_LF	%BrakeStance_LH

Table S3. Raw DigiGait measurement data. Far left column indicates time point at which data was measured: T0 = pre-infection, T1 = 21dpi, and T3 = 89dpi. DigiGait parameters listed across the top indicate which limb is associated with the data, where appropriate: FL for left fore limb, FR for right fore limb, HL for left hind limb, and HR for right hind limb.

Table S3

Trial#	Strain	Sex	Inf?	%BrakeStance_RF	%BrakeStance_RH	%BrakeStride_LF	%BrakeStride_LH	%BrakeStride_RF
1	CC002	F	N	28.5	22.9	21.5	32.8	18.4
1	CC025	F	N	50.8	35.5	35.3	36.1	35.4
1	CC012XCC032	F	N	28.9	18.9	28.6	12.8	21.8
2	CC012XCC032	F	N	42.2	35.8	24.2	28.4	27.8
1	CC012XCC032	M	N	43.9	4.5	28.8	4.3	32.8
2	CC012XCC032	M	N	68.3	8.8	41.1	8	45.3
1	CC013xCC041	F	N	54.2	29.7	30	25.6	37.5
2	CC013XCC041	F	N	73.5	32.4	42.3	13.1	55.4
1	CC013xCC041	M	N	52.5	49.5	22.6	17.5	35.2
2	CC013XCC041	M	N	47.1	32.3	31	18.5	33
1	CC032XCC013	F	N	52.4	42.2	41.5	15.1	34.7
2	CC032XCC013	F	N	46.2	28.7	35.5	23.9	30.6
1	CC041XCC012	F	N	51.6	32.6	39.1	17.1	38.3
1	CC041XCC012	F	N	39.1	32.5	36.9	20.2	25.8
2	CC041XCC012	F	N	42	31.7	23.8	29.9	21.9
1	CC032XCC013	M	N	46.7	41.7	50.4	16.6	33.8
2	CC032XCC013	M	N	61.2	31.1	16.9	31.9	36
1	CC041XCC012	M	N	61.3	20.8	42.5	22.9	40
2	CC041XCC012	M	N	63	16.3	49.6	13.9	44.7
0	CC012	F	N	41.7	31.4	18.6	10	29
2	CC012	F	N	64.1	15.3	40.2	18.3	43.3
0	CC012	M	N	57.2	17.8	26.2	6.5	33.3
2	CC012	M	N	50.5	28.9	34.5	18.3	35.6
0	CC057	F	N	47.2	36.3	30.4	27.9	33
1	CC057	F	N	32.5	33.6	25.5	20.3	23.5
0	CC057	M	N	47.2	30.6	31.5	34.6	35.7
1	CC057	M	N	27.1	30.9	21.5	18.3	20.4
0	CC078	F	N	27.8	16.1	17.4	7	18.3
1	CC078	F	N	50.9	12.7	34.9	6.3	32.8
2	CC078	F	N	50.5	43.8	29	7.7	30.1
0	CC078	M	N	38.4	20.4	10.9	6	23.8
2	CC078	M	N	48.1	36.6	18.9	22	30.2
0	CC002	F	N	55.8	45.4	30.5	19.2	37.6
1	CC002	F	N	28.5	22.9	21.5	32.8	18.4
0	CC002	M	N	49.6	30.2	29.2	16.3	34.4
1	CC002	M	N	41.2	22.2	39.1	37.1	35.7
2	CC002	M	N	67.6	24.6	47.9	11.5	44.6
0	CC006	F	N	47.3	13.6	49.4	2.7	29.9
1	CC006	F	N	67.5	15.1	42.4	17.1	48.2
2	CC006	F	N	43.5	28.9	39.7	30.2	29.6
0	CC006	M	N	66	34.6	39.8	33.3	46
0	CC023	F	N	37	29.4	31	34.2	24.5
1	CC023	F	N	50.7	29.1	27.3	10.8	26.3
2	CC023	F	N	29	28.1	29.1	27.1	18.5
0	CC023	M	N	24.2	10.7	16.2	9	15.6
1	CC023	M	N	34.7	30.7	19.6	16.4	23.5
0	CC027	F	N	41.5	34.9	16.3	10.9	21.8

Trial#	Strain	Sex	Inf?	%BrakeStance_RF	%BrakeStance_RH	%BrakeStride_LF	%BrakeStride_LH	%BrakeStride_RF
1	CC027	F	N	36.2	51	33.2	48	26.6
2	CC027	F	N	43	17.1	17	22	30.6
0	CC027	M	N	24.4	64.6	15.4	15.3	15.9
1	CC027	M	N	36	39.6	21	17.7	16.5
1	CC027	M	N	76.1	49	45	53.5	55
2	CC027	M	N	57	39.6	29.7	15	34.8
0	CC005	F	N	45.9	12.7	27.5	14.6	28
1	CC005	F	N	59.9	37.5	35.2	56	41.1
2	CC005	F	N	55.2	47.3	42.8	31	43.6
0	CC011	F	N	32	7.2	26.8	31.2	19.6
1	CC011	F	N	37.6	19.2	19.2	4.2	21.3
2	CC011	F	N	38.4	17.2	23.9	9.4	25.1
1	CC017	F	N	45.2	23.9	34.2	18.9	33.8
0	CC005	M	N	47.9	17.4	37	13	31.6
1	CC005	M	N	66.4	14.5	38.2	14.9	42.5
2	CC005	M	N	51.2	19.3	34.5	16.2	32.2
0	CC011	M	N	44	14.6	23.1	15.1	29.9
1	CC011	M	N	35	19.5	28.1	21.6	25.1
2	CC011	M	N	55.5	19.7	35.4	13.4	37.9
0	CC017	M	N	35.1	47.1	18.5	20.8	21.8
1	CC017	M	N	43.2	17.7	26	13.8	33.2
1	CC006	F	N	64.2	61.1	41.1	18.2	33.1
0	CC037	F	N	37.2	46.6	25.4	19.9	23
0	CC051	F	N	45	35.2	20	14.7	27.8
1	CC051	F	N	35	37.6	20.7	20.3	24.6
1	CC006	M	N	59.1	34.3	30.1	15.8	36
0	CC037	M	N	45.1	22.1	27.1	29.7	28.1
1	CC037	M	N	66.5	33	31.4	12.5	46.7
0	CC005	F	N	49.6	12.6	33.8	16.6	34.8
1	CC005	F	N	47.1	17.6	31.3	20.9	35.4
2	CC005	F	N	46.9	24.4	33.4	17.4	21.4
0	CC011	F	N	46.1	23.1	23.8	10.1	32.7
1	CC011	F	N	27.3	31.8	21.1	21.5	16.4
2	CC011	F	N	50.7	45.5	36.8	38.6	37.8
0	CC011	M	N	37.7	38.7	33.2	20	24.5
1	CC011	M	N	24.5	43	29	26.8	14.8
2	CC011	M	N	31.4	42.3	36	29.4	20
1	CC037	M	N	49.8	36.7	38	24.2	32.5
2	CC037	M	N	51.1	59	16.2	23.8	24.1
1	CC051	M	N	40.9	26.8	21.6	25.4	27.5
2	CC051	M	N	46.6	26.6	30.1	29	33.5
0	CC027	F	N	58.5	33.6	20.3	13.8	39.5
1	CC027	F	N	38.4	32.8	29.1	25.4	23.4
2	CC027	F	N	56.9	33.2	41.2	24.8	40.8
0	CC015	M	N	25.5	35.4	23.6	21.3	17.4
1	CC015	M	N	54.9	45.5	26.6	15.7	38
2	CC015	M	N	34.9	16.7	27.7	23.8	23.9
0	CC027	M	N	20.8	38.5	32.7	15.8	14.8

Trial#	Strain	Sex	Inf?	%BrakeStance_RF	%BrakeStance_RH	%BrakeStride_LF	%BrakeStride_LH	%BrakeStride_RF
1	CC027	M	N	44.1	33	26.7	18.5	28.4
2	CC027	M	N	39.4	19.2	26.9	15.1	25.3
0	CC015	F	N	28.6	47	22.6	31.2	18.4
1	CC015	F	N	53.2	37.7	40.9	29.7	38.1
0	CC017	F	N	43.3	36.9	31.7	19.8	28.6
0	CC023	F	N	60.2	55.7	25.2	42	41.7
1	CC023	F	N	36.3	32.6	30.9	24.5	24.5
0	CC005	M	N	74.7	49.5	29.1	10.9	47.6
1	CC005	M	N	74.1	28.9	53.1	7.9	56.9
0	CC015	M	N	50.5	41.6	29.5	16.3	34.1
1	CC015	M	N	66	33.6	29.4	29	48.2
0	CC017	M	N	40.1	43.5	26.6	40.9	28.1
1	CC017	M	N	61.1	44	41.6	18.8	41.4
0	CC023	M	N	33.5	37.9	29.1	16.1	21.4
1	CC023	M	N	42.6	17.1	36.3	13.4	28.7
0	CC051	M	N	49	37.7	46	11.2	31.7
0	CC002	F	Y	67.7	48.8	30.7	42	45
0	CC002	M	Y	53	41.5	33.8	28.3	38.2
1	CC002	M	Y	34.1	43.8	23.5	27	22.5
1	CC002	F	Y	63.3	51.2	23.6	34.6	45.4
1	CC002	F	Y	37.3	28.5	30.8	27.5	25.9
1	CC002	M	Y	34.1	43.8	23.5	27	22.5
1	CC002	M	Y	50.3	38.3	34.2	32	37.1
1	CC002	F	Y	63.3	51.2	23.6	34.6	45.4
1	CC002	F	Y	39.4	32.4	24.3	18.8	27.1
2	CC002	M	Y	42.5	34.8	23.3	17.6	26.9
2	CC002	F	Y	41.3	33.4	27.2	22.8	29.1
2	CC002	M	Y	52.8	53.6	21.4	10.6	38.3
0	CC005	F	Y	46.9	16.5	24.6	21.8	27.4
0	CC005	M	Y	36.3	18.9	24.6	7.6	23.4
0	CC005	M	Y	70.4	30.8	53.9	16	47.4
1	CC005	F	Y	54.4	21.3	31.9	10.7	33.5
1	CC005	M	Y	26	42.2	10	16.2	19.7
1	CC005	F	Y	71.7	17.2	35.6	16.4	49.6
1	CC005	M	Y	56.5	19.6	40.2	21.6	36.1
2	CC005	F	Y	77.7	24.9	40.6	25.9	52.2
2	CC005	M	Y	59.5	24.4	36.3	33.9	44
2	CC005	M	Y	59	46.6	38.9	14.7	38.5
2	CC005	M	Y	59.5	24.4	36.3	33.9	44
2	CC005	F	Y	71.7	36.6	49.3	16.5	54.9
0	CC006	F	Y	37	24.2	35.1	15.8	23.6
0	CC006	M	Y	40.7	36.8	27.7	16.6	23
0	CC006	F	Y	45.7	26.9	28.1	18	27.7
1	CC006	F	Y	51.1	43.6	33.7	27.4	29.5
1	CC006	M	Y	52.2	44.4	39.1	29.1	35.9
1	CC006	F	Y	48.8	10.3	36	6.9	29.2
1	CC006	M	Y	68.1	24.5	21.4	19.9	46.7
2	CC006	F	Y	54	11.8	28.9	7.7	36

Trial#	Strain	Sex	Inf?	%BrakeStance_RF	%BrakeStance_RH	%BrakeStride_LF	%BrakeStride_LH	%BrakeStride_RF
2	CC006	M	Y	74.3	26.3	46.7	23	56.1
0	CC011	F	Y	33.6	3.4	14.8	4.1	19.4
0	CC011	M	Y	43.2	25	21.7	19	30
0	CC011	F	Y	33.8	16.7	21.6	13.5	21.9
0	CC011	F	Y	49.7	38.6	35.8	10.4	28.5
1	CC011	F	Y	60.7	28.8	29.1	22.4	41.3
1	CC011	M	Y	29.9	31.4	41.2	23.8	23.5
1	CC011	F	Y	35.3	30.9	39.4	27.8	25.5
1	CC011	M	Y	71.1	33.7	38.7	29.7	50.8
2	CC011	F	Y	48.4	20.9	16.2	21	32
2	CC011	M	Y	57	18.8	27.5	13.1	43
2	CC011	F	Y	45.2	21.5	35.7	25.8	33.4
2	CC011	M	Y	50.6	40.1	41.1	24.6	32.5
0	CC012	M	Y	27.6	21.1	19.6	12.6	17.6
0	CC012	M	Y	53.2	25.3	26.9	24.6	34.3
0	CC012	M	Y	38.3	22.6	22	11	23.6
0	CC012	M	Y	39.1	22.3	17.3	9.1	23.7
0	CC012	M	Y	33.8	7.9	28.6	13.8	22.4
0	CC012	F	Y	48.9	26.5	23.4	18.3	26.8
0	CC012	F	Y	51.1	13.7	19.3	19.2	33.8
0	CC012	F	Y	44.8	33	33.2	18.9	29.9
0	CC012	F	Y	43.6	19	33.1	16.5	26.1
0	CC012	F	Y	46.1	19.1	19.7	8.1	28.9
2	CC012	M	Y	40	17.9	40.4	5.1	25.1
2	CC012	M	Y	34.4	14.3	31.7	11.3	21.3
2	CC012	M	Y	56.7	26.6	13.9	8.6	35.9
2	CC012	M	Y	57	31.7	22.5	12.9	38.4
2	CC012	M	Y	53	18.6	35	19.1	34.4
2	CC012	F	Y	37.8	31.5	23.1	37	23
2	CC012	F	Y	58.4	42	40.9	23.5	40.3
2	CC012	F	Y	47	33.3	20.1	27.7	30.7
2	CC012	F	Y	37.2	29.5	20.9	14.8	27.3
2	CC012	F	Y	41.1	50.3	25.9	28	30.1
1	CC012XCC032	F	Y	25.4	47.7	25.2	29.2	18.7
1	CC012xCC032	F	Y	47.6	53.8	36.9	26.6	31.5
1	CC012XCC032	M	Y	37.1	22.6	37.2	23.7	25.8
1	CC012xCC032	M	Y	49.3	20.8	29.5	12.1	31.7
1	CC012XCC032	M	Y	49.7	28.1	32	8.5	35.6
1	CC012xCC032	M	Y	39.9	33.2	29.1	39.5	32.7
2	CC012XCC032	F	Y	49.6	40.9	48.1	29.2	31.1
2	CC012XCC032	F	Y	28.9	54.9	38.4	24.3	21.5
2	CC012XCC032	M	Y	53	37.7	27.4	22.1	30.4
2	CC012XCC032	M	Y	40.3	35.6	29.3	18.3	26.5
2	CC012XCC032	M	Y	42.4	34.6	42.2	27.1	25.4
2	CC012XCC032	M	Y	62.8	43.7	44.5	4.6	43.1
1	CC013xCC041	F	Y	60.7	32.1	34.3	12.1	42.5
1	CC013xCC041	F	Y	52.4	43.3	36.7	23.2	35.8
1	CC013xCC041	F	Y	30.7	23.8	27.6	24.2	20.7

Trial#	Strain	Sex	Inf?	%BrakeStance_RF	%BrakeStance_RH	%BrakeStride_LF	%BrakeStride_LH	%BrakeStride_RF
1	CC013xCC041	M	Y	53	43.9	38.1	8.6	41.5
1	CC013xCC041	M	Y	37.9	38.3	28.1	22.2	26.3
2	CC013XCC041	F	Y	48.3	14	30.9	26.5	33.2
2	CC013XCC041	F	Y	45.3	36.5	37.6	33.2	34.7
2	CC013XCC041	F	Y	71.4	14	48	10.9	50.2
2	CC013XCC041	M	Y	49.6	70.5	46.9	25.9	37.6
2	CC013XCC041	M	Y	45.1	39.5	32.4	9.9	33.1
0	CC015	F	Y	31.3	58.5	38	23.8	21.9
0	CC015	M	Y	43.8	32.3	35.2	22.7	30.8
0	CC015	M	Y	51.4	48.6	34.2	33.7	35.6
1	CC015	F	Y	43.9	45.2	28.5	23.1	30.1
1	CC015	M	Y	57.7	37.8	35.5	32.3	43.5
1	CC015	M	Y	54.4	45.4	38.9	36.8	37.7
1	CC015	M	Y	66.9	32	43	25.9	46.8
2	CC015	F	Y	58.6	48.5	38.3	37.5	44.7
0	CC017	F	Y	40.3	29.5	18.3	16.1	24.2
0	CC017	M	Y	48.9	24.1	27.5	7.6	28.8
0	CC017	F	Y	49.5	47.3	34.8	20.6	34.7
0	CC017	M	Y	44.9	28.9	42.7	26.3	26.1
1	CC017	M	Y	62.3	26.5	32.7	20.3	35.6
1	CC017	F	Y	60.8	24.3	32.8	18.1	47.3
0	CC023	M	Y	13.9	13.4	8.4	8.8	8.5
0	CC023	F	Y	46.4	48.7	19.2	11.7	29.9
0	CC023	F	Y	40.7	29.2	26.9	25.7	24.6
0	CC023	M	Y	70	37.6	19.1	10.3	45.2
1	CC023	M	Y	51.9	17.5	27.2	18.2	32.9
1	CC023	F	Y	44.3	29.9	28.3	13.4	24.6
1	CC023	M	Y	70.5	40.7	37.6	24.3	48.6
1	CC023	M	Y	53.3	40.7	30.5	22.5	35.7
2	CC023	M	Y	23.8	23.6	37.3	7.2	18.1
2	CC023	F	Y	37.8	51	43.4	12.6	24.6
2	CC023	M	Y	45.7	17.9	25.9	7.8	31.3
0	CC025	M	Y	41.8	35.5	18.8	28.5	29.3
0	CC025	F	Y	55.6	32.1	22.4	28.7	38.2
0	CC025	M	Y	41.1	18.7	32.2	5.7	27.9
1	CC025	M	Y	40.2	35.9	17.7	25.1	29.1
1	CC025	F	Y	39.7	27.3	20.1	16.7	20.6
1	CC025	F	Y	48.2	38.4	26.9	28.4	31.8
1	CC025	F	Y	32.5	32.1	17.8	18.4	20.6
2	CC025	F	Y	32.7	29	41.7	19.9	23
2	CC025	M	Y	49.8	14.2	27.1	27.2	35
0	CC027	F	Y	55.7	21.1	35.1	13	33.7
0	CC027	M	Y	55.8	12.8	23	8.1	32.6
1	CC027	M	Y	23.7	38	25.4	37.5	17.6
1	CC027	M	Y	23.7	38	25.4	37.5	17.6
1	CC027	F	Y	51.5	32.7	21.1	14.2	28.5
1	CC027	F	Y	46.7	33.4	29.8	30.8	26.9
1	CC027	M	Y	77.6	19.6	35.9	40.8	60.1

Trial#	Strain	Sex	Inf?	%BrakeStance_RF	%BrakeStance_RH	%BrakeStride_LF	%BrakeStride_LH	%BrakeStride_RF
1	CC027	M	Y	46.6	26.6	39.9	18.6	32.5
2	CC027	F	Y	37.8	51	43.4	12.6	24.6
2	CC027	M	Y	36.5	28.4	25.6	23	24.6
2	CC027	F	Y	56.5	45.8	32.7	29.3	36.9
2	CC027	M	Y	55.3	30.5	36	15.9	37.2
1	CC032XCC013	M	Y	55.7	19	48.1	27.2	38.6
1	CC032XCC013	M	Y	36.3	20.3	37.8	24.3	25.7
1	CC032XCC013	M	Y	49	33.8	28.7	19.7	28.3
1	CC032XCC013	F	Y	42.8	25.5	26.2	25.2	33
1	CC032XCC013	F	Y	46	40.3	35.6	24	27.9
1	CC032XCC013	F	Y	46.9	36.5	43.8	24.5	32.7
1	CC032XCC013	F	Y	46.6	37.6	39.7	24.1	31.7
1	CC032XCC013	F	Y	14.5	26.4	27	39.6	8.2
2	CC032XCC013	M	Y	77.7	51.3	25.7	28.5	56.1
2	CC032XCC013	M	Y	70.6	37.2	39.6	10.1	40.1
2	CC032XCC013	M	Y	52.5	25.3	19.2	21.4	40.9
2	CC032XCC013	M	Y	65.3	37.7	35.2	27.6	36.2
2	CC032XCC013	F	Y	43.1	47.1	34	33.8	28.1
2	CC032XCC013	F	Y	44.2	41.2	42.5	33.8	31.4
2	CC032XCC013	F	Y	41.3	43	36.1	31	24.9
2	CC032XCC013	F	Y	28.3	27.7	31.1	23	18.7
0	CC037	F	Y	44	23.3	28.2	18.2	28.8
0	CC037	M	Y	39.1	29.5	22.4	17.2	23.9
0	CC037	M	Y	44.3	17.8	27.8	16.3	27.5
0	CC037	M	Y	25.4	50.4	27.3	9.8	15.2
1	CC037	F	Y	64.2	20.9	36.4	37.4	43.8
1	CC037	M	Y	28	36.5	26.2	15.2	17.1
1	CC037	M	Y	56.7	27.1	40.1	27.6	38.4
1	CC037	M	Y	37.6	28.4	25.5	30.5	24.1
2	CC037	M	Y	33.6	62.6	48.1	38	22.9
2	CC037	M	Y	35.4	48.3	20.5	24.1	23.2
1	CC041XCC012	M	Y	55.6	10.1	45.8	13.8	34
1	CC041XCC012	M	Y	66	26.1	34.4	18.6	49.4
1	CC041XCC012	M	Y	53.1	20.4	41.2	19.4	39.1
1	CC041XCC012	M	Y	45.6	18.6	45.6	23.5	33.7
1	CC041XCC012	M	Y	49	13.5	35.5	25.6	33.1
1	CC041XCC012	F	Y	52.1	39.1	40	35.1	35.7
1	CC041XCC012	F	Y	50.7	22.1	20	22.3	33.8
1	CC041XCC012	F	Y	41.3	22.2	33.2	15.8	29.6
1	CC041XCC012	F	Y	61.5	17.3	48.7	43.1	45.7
1	CC041XCC012	F	Y	59.1	18.2	36.4	16.7	38.5
1	CC041XCC012	F	Y	51.8	31.3	29.8	38	34
1	CC041XCC012	F	Y	46.1	38	32.5	25.9	33.2
1	CC041XCC012	F	Y	62.1	22.7	32.3	17.7	40.8
2	CC041XCC012	M	Y	55.8	33.9	36.3	26	40.1
2	CC041XCC012	M	Y	71.4	10.5	37.3	22.9	46.5
2	CC041XCC012	F	Y	46.5	13.8	28.7	19.5	29.5
2	CC041XCC012	M	Y	71.1	25.9	44.3	16.8	50.1

Trial#	Strain	Sex	Inf?	%BrakeStance_RF	%BrakeStance_RH	%BrakeStride_LF	%BrakeStride_LH	%BrakeStride_RF
2	CC041XCC012	M	Y	51.9	22.5	30.8	20.7	38
2	CC041XCC012	M	Y	65.2	16.3	29.8	4.9	45.3
2	CC041XCC012	F	Y	57.7	23	37.3	30.2	40.1
2	CC041XCC012	F	Y	48.7	28.2	39.8	18.3	35.5
2	CC041XCC012	F	Y	67.6	42.5	32.1	31.9	48.3
2	CC041XCC012	F	Y	48.1	31.6	29.3	24	32.5
2	CC041XCC012	F	Y	48.9	36.9	29.8	30.7	34.9
2	CC041XCC012	F	Y	55.7	31.8	37.8	21.7	42
2	CC041XCC012	F	Y	56.3	15.2	29.3	10.8	38.7
0	CC051	F	Y	57	34.8	29.7	28	38.4
0	CC051	F	Y	37	42.1	23	17.4	20.7
0	CC051	F	Y	41.4	34.7	24.7	16.2	28.7
0	CC051	M	Y	45	48.7	31.6	20.9	32.3
1	CC051	F	Y	65.3	39	28.5	26.2	43.1
1	CC051	F	Y	54	43.2	29.2	28.2	36.1
1	CC051	F	Y	41	26.9	30.3	25.4	28
0	CC057	F	Y	31.6	22	32.6	27.3	22.8
0	CC057	F	Y	44.1	24.9	31.2	15.7	31.6
0	CC057	F	Y	32.7	31.8	38.3	31.2	25.5
0	CC057	M	Y	40.9	34.2	34.3	36	27.5
0	CC057	M	Y	53.9	31.8	43.3	34.9	42.1
0	CC057	M	Y	60.4	32.7	47	21.1	41.1
1	CC057	F	Y	34.7	21.6	35.7	6.9	25.9
1	CC057	F	Y	34.4	20.3	29.8	23	26.8
1	CC057	F	Y	61.1	34.1	31.9	16.8	49.5
1	CC057	F	Y	60.6	28	44.2	18.9	41.1
1	CC057	M	Y	38.4	25.5	41.5	4.4	25.1
1	CC057	M	Y	40.8	40.7	39.5	25.8	32.3
2	CC057	F	Y	60.6	28	44.2	18.9	41.1
0	CC078	F	Y	48.9	27.4	21.6	5.3	31.2
0	CC078	F	Y	27.1	21	19.5	25.8	17.4
0	CC078	F	Y	31.2	16.3	24.7	12.2	19.8
0	CC078	M	Y	31	38.7	26.5	13.7	20.8
1	CC078	F	Y	25.3	22.5	22.6	18.4	19.3
1	CC078	F	Y	28.6	12.2	34.5	7.8	19.3
1	CC078	M	Y	58.2	42.3	34.1	17.3	39.9
2	CC078	F	Y	51.8	31.4	14.5	25.3	36.8
2	CC078	M	Y	57.2	44.9	38.3	14.4	40.8
2	CC005	M	N	50.4	24.2	38.6	16.1	42.9
2	CC005	M	Y	39.1	46.4	22.8	14.7	22.5
2	CC015	F	N	29.3	37.3	37	25.2	17.6
2	CC015	M	N	54.5	45.1	41.8	25.5	37.7
2	CC015	M	Y	54.5	42.6	47	33.1	36.2
2	CC015	M	Y	42.4	43.8	38.7	40.9	29.6
2	CC017	F	N	38.9	26.6	40.1	26.6	24.4
2	CC017	M	N	42.6	32.3	22.7	20.9	21.7
2	CC017	F	Y	54.3	12.4	55.2	10.9	39.1
2	CC023	F	N	63.9	28.6	46.8	25.4	43

Trial#	Strain	Sex	Inf?	%BrakeStance_RF	%BrakeStance_RH	%BrakeStride_LF	%BrakeStride_LH	%BrakeStride_RF

Table S3. Raw DigiGait measurement data. Far left column indicates time point at which data was measured: T0 = pre-infection, T1 = 21dpi, and T3 = 89dpi. DigiGait parameters listed across the top indicate which limb is associated with the data, where appropriate: FL for left fore limb, FR for right fore limb, HL for left hind limb, and HR for right hind limb.

Table S3

Trial#	Strain	Sex	Inf?	%BrakeStride_RH	%PropelStance_LF	%PropelStance_LH	%PropelStance_RF
1	CC002	F	N	16.9	69.2	54.4	71.5
1	CC025	F	N	26.4	48.7	53.6	49.2
1	CC012XCC032	F	N	13.2	56.7	82.3	71.1
2	CC012XCC032	F	N	26.3	62.7	57.2	57.8
1	CC012XCC032	M	N	3.7	59.6	94.9	56.1
2	CC012XCC032	M	N	7.5	38.7	90.3	31.7
1	CC013xCC041	F	N	22.2	57.5	61.2	45.8
2	CC013XCC041	F	N	25.2	44.9	82.4	26.5
1	CC013xCC041	M	N	31.9	53.2	73.2	47.5
2	CC013XCC041	M	N	24.6	55.4	75.7	52.9
1	CC032XCC013	F	N	32	45.3	77.8	47.6
2	CC032XCC013	F	N	22.8	50.3	69.6	53.8
1	CC041XCC012	F	N	22.3	49.4	74.5	48.4
1	CC041XCC012	F	N	22.3	49.3	70.3	60.9
2	CC041XCC012	F	N	22.8	61.4	60.7	58
1	CC032XCC013	M	N	26.3	35.3	74.7	53.3
2	CC032XCC013	M	N	18.4	66.3	59.1	38.8
1	CC041XCC012	M	N	14.7	34.9	67.9	38.7
2	CC041XCC012	M	N	10.8	31.1	81.4	37
0	CC012	F	N	22.6	71.8	85.4	58.3
2	CC012	F	N	10	41.2	74.3	35.9
0	CC012	M	N	13	53.5	90.8	42.8
2	CC012	M	N	23.9	53	76.3	49.5
0	CC057	F	N	26.2	56.9	62.3	52.8
1	CC057	F	N	24.7	64.7	72.2	67.5
0	CC057	M	N	23.4	57.9	53	52.8
1	CC057	M	N	22.8	69	74.4	72.9
0	CC078	F	N	11.7	73	90.4	72.2
1	CC078	F	N	8.9	47.3	90.8	49.1
2	CC078	F	N	27.8	54.9	90.4	49.5
0	CC078	M	N	13.2	81.5	90.2	61.6
2	CC078	M	N	24.9	68.8	68.4	51.9
0	CC002	F	N	28.9	56.6	71.4	44.2
1	CC002	F	N	16.9	69.2	54.4	71.5
0	CC002	M	N	18.4	53.2	77.4	50.4
1	CC002	M	N	15.8	46.4	50.8	58.8
2	CC002	M	N	18.7	27.1	85	32.4
0	CC006	F	N	9.1	17.9	96.2	52.7
1	CC006	F	N	10.5	36	76.5	32.5
2	CC006	F	N	18.4	42.7	58.5	56.5
0	CC006	M	N	18.5	46.7	56.6	34
0	CC023	F	N	21.7	54.6	51.5	63
1	CC023	F	N	18.9	58.1	83.9	49.3
2	CC023	F	N	18.3	54.5	62.7	71
0	CC023	M	N	7.1	75.5	87.8	75.8
1	CC023	M	N	21.9	68.7	77.7	65.3
0	CC027	F	N	15.5	60	63.2	58.5

Trial#	Strain	Sex	Inf?	%BrakeStride_RH	%PropelStance_LF	%PropelStance_LH	%PropelStance_RF
1	CC027	F	N	35.1	52.9	33.3	63.8
2	CC027	F	N	12	74.2	69.5	57
0	CC027	M	N	26.8	69.9	64.7	75.6
1	CC027	M	N	29.2	65.3	75.9	64
1	CC027	M	N	36.7	45.6	37.9	23.9
2	CC027	M	N	24.7	53	76.7	43
0	CC005	F	N	9.4	56.9	80.1	54.1
1	CC005	F	N	28.7	47.7	25.5	40.1
2	CC005	F	N	29.1	36.1	46.6	44.8
0	CC011	F	N	5.5	53.1	55.8	68
1	CC011	F	N	15.1	67.1	94.8	62.4
2	CC011	F	N	13	64.4	87.3	61.6
1	CC017	F	N	19	52.7	74.9	54.8
0	CC005	M	N	12.8	44.3	82.3	52.1
1	CC005	M	N	10.9	42.9	77.2	33.6
2	CC005	M	N	16.4	50.9	79	48.8
0	CC011	M	N	10.4	64.4	79.7	56
1	CC011	M	N	14.1	53.8	70.3	65
2	CC011	M	N	15.7	48.7	83	44.5
0	CC017	M	N	19.6	67.2	53.9	64.9
1	CC017	M	N	12.3	57.6	81.4	56.8
1	CC006	F	N	32.8	29.5	50.9	35.8
0	CC037	F	N	28	56.7	72.6	62.8
0	CC051	F	N	27.1	68.5	79.9	55
1	CC051	F	N	28.9	67.8	72.9	65
1	CC006	M	N	23.2	51.5	75.7	40.9
0	CC037	M	N	15.7	54.7	57.8	54.9
1	CC037	M	N	25.7	49.9	81	33.5
0	CC005	F	N	9.1	47.5	78.4	50.4
1	CC005	F	N	13.1	58.5	72.8	52.9
2	CC005	F	N	12.1	47.8	73.2	53.1
0	CC011	F	N	17.2	65.2	86.6	53.9
1	CC011	F	N	23.9	68.2	71.8	72.7
2	CC011	F	N	36	52	49.6	49.3
0	CC011	M	N	28.2	47.8	71.6	62.3
1	CC011	M	N	30.3	53.7	62.2	75.5
2	CC011	M	N	29.1	42	60.4	68.6
1	CC037	M	N	28.5	45.8	68.2	50.2
2	CC037	M	N	48.6	71.5	69.6	48.9
1	CC051	M	N	20.6	66.9	65.5	59.1
2	CC051	M	N	20.4	51.9	55.1	53.4
0	CC027	F	N	23.1	64.2	82.2	41.5
1	CC027	F	N	19.5	55.9	64.1	61.6
2	CC027	F	N	18.8	42.4	67.7	43.1
0	CC015	M	N	24.2	68.5	68	74.5
1	CC015	M	N	19.4	51	69.1	45.1
2	CC015	M	N	9.9	59.7	65.2	65.1
0	CC027	M	N	26.4	52.7	78.4	79.2

Trial#	Strain	Sex	Inf?	%BrakeStride_RH	%PropelStance_LF	%PropelStance_LH	%PropelStance_RF
1	CC027	M	N	23.1	54.3	74.2	55.9
2	CC027	M	N	13.8	56	75.7	60.6
0	CC015	F	N	36.8	70.3	56.5	71.4
1	CC015	F	N	24	44	56	46.8
0	CC017	F	N	22.6	46.9	69.2	56.7
0	CC023	F	N	41.3	61.1	41.6	39.8
1	CC023	F	N	21.6	53.4	65.9	63.7
0	CC005	M	N	25.3	55.8	84.6	25.3
1	CC005	M	N	13.3	25.2	89.8	25.9
0	CC015	M	N	31.1	55.2	78.2	49.5
1	CC015	M	N	21.2	59.6	60.5	34
0	CC017	M	N	33.5	63.2	49.5	59.9
1	CC017	M	N	32	39.1	72.5	38.9
0	CC023	M	N	27	51.6	77.3	66.5
1	CC023	M	N	13	41.5	81	57.4
0	CC051	M	N	28.2	33.1	85.4	51
0	CC002	F	Y	35.1	47.1	47.8	32.3
0	CC002	M	Y	28.5	49.5	62.9	47
1	CC002	M	Y	6.7	61.5	47.5	65.9
1	CC002	F	Y	27.7	63.9	29	36.7
1	CC002	F	Y	19.8	54.4	62.3	62.7
1	CC002	M	Y	6.7	61.5	47.5	65.9
1	CC002	M	Y	27	54.2	56.3	49.7
1	CC002	F	Y	27.7	63.9	29	36.7
1	CC002	F	Y	15.9	67.1	60.2	60.6
2	CC002	M	Y	25.1	63.8	72.9	57.5
2	CC002	F	Y	24.8	59.1	69.3	58.7
2	CC002	M	Y	39	70.9	85.8	47.2
0	CC005	F	Y	12.1	57.7	71.2	53.1
0	CC005	M	Y	14.3	62.6	89.5	63.7
0	CC005	M	Y	23.7	26.4	78.9	29.6
1	CC005	F	Y	14.3	48.6	85.7	45.6
1	CC005	M	Y	10.9	86.3	74.6	74
1	CC005	F	Y	7.9	44.5	74.8	28.3
1	CC005	M	Y	14.4	39.3	68.9	43.5
2	CC005	F	Y	20.1	29.9	66.5	22.3
2	CC005	M	Y	17.6	46.6	56.1	40.5
2	CC005	M	Y	20.6	39.1	81.6	41
2	CC005	M	Y	17.6	46.6	56.1	40.5
2	CC005	F	Y	17.5	27.5	78.9	28.3
0	CC006	F	Y	17.8	45.7	77.7	63
0	CC006	M	Y	10	60.2	71.7	59.3
0	CC006	F	Y	20.8	57.1	75.7	54.3
1	CC006	F	Y	33.4	47.4	62.8	48.9
1	CC006	M	Y	33.7	50.6	58.5	47.8
1	CC006	F	Y	7.2	44.6	90	51.2
1	CC006	M	Y	18.1	66.3	71	31.9
2	CC006	F	Y	8.4	51.3	87.8	46

Trial#	Strain	Sex	Inf?	%BrakeStride_RH	%PropelStance_LF	%PropelStance_LH	%PropelStance_RF
2	CC006	M	Y	16.3	36.1	66.2	25.7
0	CC011	F	Y	2.7	71.2	94.8	66.4
0	CC011	M	Y	18.3	69.2	75	56.8
0	CC011	F	Y	11	64.9	79.9	66.2
0	CC011	F	Y	15.6	47.5	55.3	50.3
1	CC011	F	Y	20.5	56.7	69.5	39.3
1	CC011	M	Y	23.1	42.8	68.5	70.1
1	CC011	F	Y	19.1	43.9	60.4	64.7
1	CC011	M	Y	24.4	39.6	58.7	28.9
2	CC011	F	Y	15	76.4	70.9	51.6
2	CC011	M	Y	15.2	64	83.5	43
2	CC011	F	Y	15.7	51.7	66.9	54.8
2	CC011	M	Y	26.9	34.9	65.4	49.4
0	CC012	M	Y	14.2	69.1	82.3	72.4
0	CC012	M	Y	16.8	60	60.9	46.8
0	CC012	M	Y	15.5	66.3	83.8	61.7
0	CC012	M	Y	16	73.7	86.4	60.9
0	CC012	M	Y	5.6	58.2	79.3	66.2
0	CC012	F	Y	20.3	62.4	74.7	51.1
0	CC012	F	Y	9.7	71.8	73.9	48.9
0	CC012	F	Y	23.5	54.9	70.4	55.2
0	CC012	F	Y	11	41.9	72.7	56.4
0	CC012	F	Y	11	68.6	85	53.9
2	CC012	M	Y	12.5	39.7	92.9	60
2	CC012	M	Y	9.4	53.8	82.3	65.6
2	CC012	M	Y	17.8	78.3	87.6	43.3
2	CC012	M	Y	24.5	68.5	82.2	43
2	CC012	M	Y	12.5	44.4	72.4	47
2	CC012	F	Y	24	64.9	51.4	62.2
2	CC012	F	Y	26.5	32.2	64.4	41.6
2	CC012	F	Y	25.5	69.9	60.7	53
2	CC012	F	Y	19.8	68.1	78.9	62.8
2	CC012	F	Y	37.1	63.1	60.8	58.9
1	CC012XCC032	F	Y	29.2	67.7	55.2	74.6
1	CC012xCC032	F	Y	28.7	47.7	56	52.4
1	CC012XCC032	M	Y	16	46.8	68	62.9
1	CC012xCC032	M	Y	14.4	52	82.2	50.7
1	CC012XCC032	M	Y	16.1	45	87.8	50.3
1	CC012xCC032	M	Y	24.2	63	45.7	60.1
2	CC012XCC032	F	Y	29.6	33.6	58.2	50.4
2	CC012XCC032	F	Y	38.9	45.8	64.8	71.1
2	CC012XCC032	M	Y	27	59.4	70.1	47
2	CC012XCC032	M	Y	22.3	48	72.6	59.7
2	CC012XCC032	M	Y	26.2	37.6	64.7	57.6
2	CC012XCC032	M	Y	34.1	35.6	92.6	37.2
1	CC013xCC041	F	Y	22.6	50.4	81.7	39.3
1	CC013xCC041	F	Y	26.5	47.1	64.9	47.6
1	CC013xCC041	F	Y	18.8	59.6	68.8	69.3

Trial#	Strain	Sex	Inf?	%BrakeStride_RH	%PropelStance_LF	%PropelStance_LH	%PropelStance_RF
1	CC013xCC041	M	Y	32.3	47.7	88.6	47
1	CC013xCC041	M	Y	27.6	61.2	71.5	62.1
2	CC013XCC041	F	Y	10.4	55.5	63.4	51.7
2	CC013XCC041	F	Y	30	47.2	55.7	54.7
2	CC013XCC041	F	Y	10.7	27.3	85.3	28.6
2	CC013XCC041	M	Y	54.5	35.9	67.8	50.4
2	CC013XCC041	M	Y	25.7	54.9	87.3	54.9
0	CC015	F	Y	42.6	43.6	65	68.7
0	CC015	M	Y	24.1	47.6	70.4	56.2
0	CC015	M	Y	36.3	50.4	55.4	48.6
1	CC015	F	Y	27.7	58.4	66.1	56.1
1	CC015	M	Y	23.3	51.3	54.9	42.3
1	CC015	M	Y	32.8	48.7	45.4	45.6
1	CC015	M	Y	24.7	37.4	66.1	33.1
2	CC015	F	Y	34.8	46.7	48.8	41.4
0	CC017	F	Y	18.7	68.3	74.3	59.7
0	CC017	M	Y	18.5	60.8	88.7	51.1
0	CC017	F	Y	37.3	47.7	73.7	50.5
0	CC017	M	Y	19.2	33.6	64.3	55.1
1	CC017	M	Y	19.9	43.6	73	37.7
1	CC017	F	Y	17.4	49.4	70.4	39.2
0	CC023	M	Y	8.5	85.6	87.6	86.1
0	CC023	F	Y	29.2	69	83.6	53.6
0	CC023	F	Y	16.7	55.3	58.4	59.3
0	CC023	M	Y	23.1	43.6	87.7	30
1	CC023	M	Y	15	61.4	78	48.1
1	CC023	F	Y	25.9	53.2	81.4	55.7
1	CC023	M	Y	27.7	49.2	70.5	29.5
1	CC023	M	Y	28.2	54.3	67.8	46.7
2	CC023	M	Y	15.6	54.6	86.4	76.2
2	CC023	F	Y	39.4	39.2	83.3	62.2
2	CC023	M	Y	13.5	58.2	88.1	54.3
0	CC025	M	Y	24.1	72	63.1	58.2
0	CC025	F	Y	23	63	64.6	44.4
0	CC025	M	Y	11.3	53.5	92.5	58.9
1	CC025	M	Y	24.4	73.7	67.9	59.8
1	CC025	F	Y	17.8	61.1	75.6	60.3
1	CC025	F	Y	26.4	57.9	60.8	51.8
1	CC025	F	Y	20.4	73	73.1	67.5
2	CC025	F	Y	18.6	41	72.2	67.3
2	CC025	M	Y	10.1	59.1	64.3	50.2
0	CC027	F	Y	12.8	46.9	82.3	44.3
0	CC027	M	Y	8.4	63.1	88.6	44.2
1	CC027	M	Y	28.7	71.8	46.7	76.3
1	CC027	M	Y	28.7	71.8	46.7	76.3
1	CC027	F	Y	21.4	60.3	75.4	48.5
1	CC027	F	Y	16.3	50.7	56.2	53.3
1	CC027	M	Y	16.1	50.6	49.7	22.4

Trial#	Strain	Sex	Inf?	%BrakeStride_RH	%PropelStance_LF	%PropelStance_LH	%PropelStance_RF
1	CC027	M	Y	19.6	44.1	75.5	53.4
2	CC027	F	Y	39.4	39.2	83.3	62.2
2	CC027	M	Y	18.9	60.2	67.1	63.5
2	CC027	F	Y	27.8	53	58	43.5
2	CC027	M	Y	23.3	44	78.8	44.7
1	CC032XCC013	M	Y	14.1	29.3	62.6	44.3
1	CC032XCC013	M	Y	13.9	47.8	70.1	63.7
1	CC032XCC013	M	Y	18.1	47.5	68.2	51
1	CC032XCC013	F	Y	18.3	59.3	62.2	57.2
1	CC032XCC013	F	Y	26	50.4	60.6	54
1	CC032XCC013	F	Y	24	36.9	65.7	53.1
1	CC032XCC013	F	Y	24.5	46.5	66.5	53.4
1	CC032XCC013	F	Y	19.4	61	47.9	85.5
2	CC032XCC013	M	Y	39.4	65.6	62.9	22.3
2	CC032XCC013	M	Y	28.2	40.4	85.3	29.4
2	CC032XCC013	M	Y	16.9	61.3	71	47.5
2	CC032XCC013	M	Y	28.1	47.9	57.4	34.7
2	CC032XCC013	F	Y	33.6	51.8	56.9	56.9
2	CC032XCC013	F	Y	30	37.3	54.5	55.8
2	CC032XCC013	F	Y	29.2	51.7	57.4	58.7
2	CC032XCC013	F	Y	18.9	54.6	70.7	71.7
0	CC037	F	Y	17.9	56.6	75.7	56
0	CC037	M	Y	23.4	60.3	73.4	60.9
0	CC037	M	Y	8.4	56.9	75.8	55.7
0	CC037	M	Y	18.3	52.3	74.3	74.6
1	CC037	F	Y	15.4	46.6	50.4	35.8
1	CC037	M	Y	24.2	59.5	75.5	72
1	CC037	M	Y	18.3	42.4	60.8	43.3
1	CC037	M	Y	19.9	61.7	57.8	62.4
2	CC037	M	Y	45	31.2	50	66.4
2	CC037	M	Y	27	69.1	65.5	64.6
1	CC041XCC012	M	Y	7.8	40.1	82.8	44.4
1	CC041XCC012	M	Y	20	41.1	76	34
1	CC041XCC012	M	Y	14.9	45	70.1	46.9
1	CC041XCC012	M	Y	12.8	38.8	67.6	54.4
1	CC041XCC012	M	Y	10.3	49.6	65.6	51
1	CC041XCC012	F	Y	28.6	44.8	54.3	47.9
1	CC041XCC012	F	Y	16.4	70.2	69.7	49.3
1	CC041XCC012	F	Y	17.5	53.8	78.1	58.7
1	CC041XCC012	F	Y	12.5	32.7	44.8	38.5
1	CC041XCC012	F	Y	12.8	51.1	77	40.9
1	CC041XCC012	F	Y	21	55.5	47.3	48.2
1	CC041XCC012	F	Y	27.8	46.2	64.7	53.9
1	CC041XCC012	F	Y	17.7	58.7	77.6	37.9
2	CC041XCC012	M	Y	24.4	49.5	65.2	44.2
2	CC041XCC012	M	Y	7.9	42.2	68.2	28.6
2	CC041XCC012	F	Y	8.5	54.5	72.9	53.5
2	CC041XCC012	M	Y	20.4	41.9	69.2	28.9

Trial#	Strain	Sex	Inf?	%BrakeStride_RH	%PropelStance_LF	%PropelStance_LH	%PropelStance_RF
2	CC041XCC012	M	Y	17.3	54.4	70.5	48.1
2	CC041XCC012	M	Y	11.8	57.9	89.9	34.8
2	CC041XCC012	F	Y	17.7	46	58.2	42.3
2	CC041XCC012	F	Y	21.2	45.4	77	51.3
2	CC041XCC012	F	Y	30.5	54.4	57.4	32.4
2	CC041XCC012	F	Y	21.8	56.8	64.8	51.9
2	CC041XCC012	F	Y	28.9	57.6	59.7	51.1
2	CC041XCC012	F	Y	25.5	47.1	72.1	44.3
2	CC041XCC012	F	Y	11.2	55.3	85.5	43.7
0	CC051	F	Y	26	55.3	63.1	43
0	CC051	F	Y	32.9	66.6	77.6	63
0	CC051	F	Y	25.5	61.8	78.3	58.6
0	CC051	M	Y	36.6	52.5	72.2	55
1	CC051	F	Y	29.9	57.1	64.4	34.7
1	CC051	F	Y	32.4	59.5	62.3	46
1	CC051	F	Y	20.8	54.2	67.2	59
0	CC057	F	Y	16.4	54	63.1	68.4
0	CC057	F	Y	17.1	52.5	77.7	55.9
0	CC057	F	Y	23.1	48.4	59.8	67.3
0	CC057	M	Y	26.9	53.9	54.2	59.1
0	CC057	M	Y	20.3	44.1	42.4	46.1
0	CC057	M	Y	24.8	37.7	71.2	39.6
1	CC057	F	Y	16.3	54.1	89.9	65.3
1	CC057	F	Y	13.6	57.3	70.5	65.6
1	CC057	F	Y	20.9	56.3	79.3	38.9
1	CC057	F	Y	19.7	41.2	73.4	39.4
1	CC057	M	Y	18.9	41.8	93.6	61.6
1	CC057	M	Y	30.1	48.6	64.9	59.2
2	CC057	F	Y	19.7	41.2	73.4	39.4
0	CC078	F	Y	18.7	63.7	91.8	51.1
0	CC078	F	Y	14.2	70.4	62.6	72.9
0	CC078	F	Y	11.1	61.5	82.5	68.8
0	CC078	M	Y	26.8	61.1	77.7	69
1	CC078	F	Y	15.4	65.8	75.2	74.7
1	CC078	F	Y	7	52.6	89.5	71.4
1	CC078	M	Y	28.9	45.7	71.5	41.8
2	CC078	F	Y	21.3	78.6	66.2	48.2
2	CC078	M	Y	32.2	38.2	78.2	42.8
2	CC005	M	N	17.3	38.5	80	49.6
2	CC005	M	Y	29.7	54	77.8	60.9
2	CC015	F	N	26.4	46.7	61.1	70.7
2	CC015	M	N	32.3	38.2	64.9	45.5
2	CC015	M	Y	32.1	37.2	57.7	45.5
2	CC015	M	Y	33.2	49	46.8	57.6
2	CC017	F	N	19.3	42.2	62.6	61.1
2	CC017	M	N	20.5	58	68.8	57.4
2	CC017	F	Y	8.3	26.4	83	45.7
2	CC023	F	N	17.1	30.9	63.3	36.1

Trial#	Strain	Sex	Inf?	%BrakeStride_RH	%PropelStance_LF	%PropelStance_LH	%PropelStance_RF

Table S3. Raw DigiGait measurement data. Far left column indicates time point at which data was measured: T0 = pre-infection, T1 = 21dpi, and T3 = 89dpi. DigiGait parameters listed across the top indicate which limb is associated with the data, where appropriate: FL for left fore limb, FR for right fore limb, HL for left hind limb, and HR for right hind limb.

Table S3

Trial#	Strain	Sex	Inf?	%PropelStance_RH	%PropelStride_LF	%PropelStride_LH	%PropelStride_RF
1	CC002	F	N	77.1	48.4	39.2	46.1
1	CC025	F	N	64.5	33.5	41.7	34.2
1	CC012XCC032	F	N	81.1	37.4	59.5	53.5
2	CC012XCC032	F	N	64.2	40.8	38	38.1
1	CC012XCC032	M	N	95.5	42.6	79.9	41.9
2	CC012XCC032	M	N	91.2	25.9	74.5	21
1	CC013xCC041	F	N	70.3	40.6	40.3	31.7
2	CC013XCC041	F	N	67.6	34.5	61	19.9
1	CC013xCC041	M	N	50.5	25.7	47.8	31.8
2	CC013XCC041	M	N	67.7	38.5	57.7	37
1	CC032XCC013	F	N	57.8	34.3	52.8	31.5
2	CC032XCC013	F	N	71.3	35.8	54.6	35.6
1	CC041XCC012	F	N	67.4	38.1	49.8	36
1	CC041XCC012	F	N	67.5	35.9	47.6	40.2
2	CC041XCC012	F	N	68.3	37.7	46.3	30.2
1	CC032XCC013	M	N	58.3	27.4	49.2	38.5
2	CC032XCC013	M	N	68.9	33.1	46.1	22.8
1	CC041XCC012	M	N	79.2	22.8	48.5	25.2
2	CC041XCC012	M	N	83.7	22.4	60.9	26.2
0	CC012	F	N	68.6	47.2	58.7	40.6
2	CC012	F	N	84.7	28.2	53.1	24.2
0	CC012	M	N	82.2	30.2	64.1	24.9
2	CC012	M	N	71.1	38.9	58.9	34.9
0	CC057	F	N	63.7	40.1	46.1	37
1	CC057	F	N	66.4	46.7	52.8	48.9
0	CC057	M	N	69.4	43.3	39	39.9
1	CC057	M	N	69.1	47.9	53.1	54.9
0	CC078	F	N	83.9	47.1	65.6	47.5
1	CC078	F	N	87.3	31.3	62.1	31.6
2	CC078	F	N	56.2	35.3	73.2	29.5
0	CC078	M	N	79.6	48	55.8	38.2
2	CC078	M	N	63.4	41.7	47.8	32.6
0	CC002	F	N	54.6	39.7	48.1	29.7
1	CC002	F	N	77.1	48.4	39.2	46.1
0	CC002	M	N	69.8	33.3	55.8	35
1	CC002	M	N	77.8	33.8	38.3	51.1
2	CC002	M	N	75.4	17.8	65.1	21.4
0	CC006	F	N	86.4	10.8	67.8	33.3
1	CC006	F	N	84.9	23.9	55.5	23.2
2	CC006	F	N	71.1	29.6	42.6	38.4
0	CC006	M	N	65.4	34.8	43.4	23.6
0	CC023	F	N	70.6	37.3	36.3	41.7
1	CC023	F	N	70.9	37.9	56.5	25.6
2	CC023	F	N	71.9	34.8	45.5	45.3
0	CC023	M	N	89.3	49.8	65.1	48.7
1	CC023	M	N	69.3	43.1	57	44.2
0	CC027	F	N	65.1	24.5	18.7	30.8

Trial#	Strain	Sex	Inf?	%PropelStance_RH	%PropelStride_LF	%PropelStride_LH	%PropelStride_RF
1	CC027	F	N	49	37.4	23.9	46.9
2	CC027	F	N	82.9	48.7	50	40.6
0	CC027	M	N	35.4	35.7	28.1	49.2
1	CC027	M	N	60.4	39.5	55.9	29.3
1	CC027	M	N	51	37.6	32.7	17.3
2	CC027	M	N	60.4	33.4	49.5	26.2
0	CC005	F	N	87.3	36.4	58.8	33
1	CC005	F	N	62.5	32.1	19.2	27.5
2	CC005	F	N	52.7	24.2	27	35.3
0	CC011	F	N	92.8	30.3	39.4	41.7
1	CC011	F	N	80.8	39.2	75.8	35.3
2	CC011	F	N	82.8	43.2	64.3	40.3
1	CC017	F	N	76.1	38	56.3	41.1
0	CC005	M	N	82.6	29.4	60.6	34.3
1	CC005	M	N	85.5	28.7	50.5	21.5
2	CC005	M	N	80.7	35.8	61	30.7
0	CC011	M	N	85.4	41.8	59	38
1	CC011	M	N	80.5	32.8	51.1	46.8
2	CC011	M	N	80.3	33.6	65	30.4
0	CC017	M	N	52.9	37.9	24.3	40.3
1	CC017	M	N	82.3	35.2	60.3	43.6
1	CC006	F	N	38.9	17.1	18.8	18.5
0	CC037	F	N	53.4	33.3	52.7	38.8
0	CC051	F	N	64.8	43.4	58.4	34
1	CC051	F	N	62.4	43.5	54.6	45.7
1	CC006	M	N	65.7	32	49.4	24.9
0	CC037	M	N	77.9	32.7	40.7	34.2
1	CC037	M	N	67	31.3	53.1	23.5
0	CC005	F	N	87.4	30.7	60.2	35.3
1	CC005	F	N	82.4	44.1	55.9	39.7
2	CC005	F	N	75.6	30.6	47.6	24.2
0	CC011	F	N	76.9	44.6	65.1	38.2
1	CC011	F	N	68.2	45.2	54.5	43.6
2	CC011	F	N	54.5	39.8	38.1	36.7
0	CC011	M	N	61.3	30.4	50.6	40.5
1	CC011	M	N	57	33.6	43.9	45.6
2	CC011	M	N	57.7	26.1	44.9	43.5
1	CC037	M	N	63.3	32.1	52	32.8
2	CC037	M	N	41	40.6	54.6	23
1	CC051	M	N	73.2	43.7	48	39.8
2	CC051	M	N	73.4	32.4	35.6	38.4
0	CC027	F	N	66.4	36.5	63.6	28
1	CC027	F	N	67.2	36.8	45.4	37.5
2	CC027	F	N	66.8	30.3	52	30.9
0	CC015	M	N	64.6	51.2	45.2	51
1	CC015	M	N	54.5	27.7	35.1	31.2
2	CC015	M	N	83.3	41	44.6	44.5
0	CC027	M	N	61.5	36.4	57.2	56.5

Trial#	Strain	Sex	Inf?	%PropelStance_RH	%PropelStride_LF	%PropelStride_LH	%PropelStride_RF
1	CC027	M	N	67	31.8	53.3	36
2	CC027	M	N	80.8	34.2	46.9	38.9
0	CC015	F	N	53	53.5	40.5	46
1	CC015	F	N	62.3	32.1	37.7	33.5
0	CC017	F	N	63.1	28	44.4	37.5
0	CC023	F	N	44.3	39.6	29.9	27.6
1	CC023	F	N	67.4	35.5	47.5	43
0	CC005	M	N	50.5	36.7	59.5	16.1
1	CC005	M	N	71.1	17.9	69.4	19.9
0	CC015	M	N	58.4	36.4	58.5	33.5
1	CC015	M	N	66.4	43.2	44.4	24.8
0	CC017	M	N	56.5	45.8	40.1	42
1	CC017	M	N	56	26.7	49.5	26.3
0	CC023	M	N	62.1	31.1	54.9	42.7
1	CC023	M	N	82.9	25.7	57	38.6
0	CC051	M	N	62.3	22.8	65.3	33
0	CC002	F	Y	51.2	27.3	38.5	21.5
0	CC002	M	Y	58.5	33.1	48	33.9
1	CC002	M	Y	56.2	37.6	24.3	43.4
1	CC002	F	Y	48.8	41.6	14.1	26.3
1	CC002	F	Y	71.5	36.7	45.5	43.6
1	CC002	M	Y	56.2	37.6	24.3	43.4
1	CC002	M	Y	61.7	40.5	41.2	36.6
1	CC002	F	Y	48.8	41.6	14.1	26.3
1	CC002	F	Y	67.6	49.5	28.4	41.7
2	CC002	M	Y	65.2	41	47.5	36.3
2	CC002	F	Y	66.6	39.3	51.4	41.4
2	CC002	M	Y	46.4	52.2	63.9	34.3
0	CC005	F	Y	83.5	33.5	54.1	30.9
0	CC005	M	Y	81.1	41.3	64.8	41.1
0	CC005	M	Y	69.2	19.4	59.7	19.9
1	CC005	F	Y	78.7	30.2	64.4	28.1
1	CC005	M	Y	57.8	62.7	47.5	56.1
1	CC005	F	Y	82.8	28.6	48.8	19.6
1	CC005	M	Y	80.4	26	47.9	27.8
2	CC005	F	Y	75.1	17.4	51.4	14.9
2	CC005	M	Y	75.6	31.7	43.3	30
2	CC005	M	Y	53.4	24.9	65.2	26.8
2	CC005	M	Y	75.6	31.7	43.3	30
2	CC005	F	Y	63.4	18.7	61.8	21.7
0	CC006	F	Y	75.8	29.6	55.1	40.2
0	CC006	M	Y	63.2	42	42.1	33.6
0	CC006	F	Y	73.1	37.5	56.1	33
1	CC006	F	Y	56.4	30.3	46.2	28.1
1	CC006	M	Y	55.6	40.1	41	32.9
1	CC006	F	Y	89.7	28.9	62	30.7
1	CC006	M	Y	75.5	42.1	48.8	21.9
2	CC006	F	Y	88.2	30.5	55.3	30.7

Trial#	Strain	Sex	Inf?	%PropelStance_RH	%PropelStride_LF	%PropelStride_LH	%PropelStride_RF
2	CC006	M	Y	73.7	26.4	45	19.4
0	CC011	F	Y	96.6	36.7	74.8	38.2
0	CC011	M	Y	75	48.7	57.2	39.4
0	CC011	F	Y	83.3	39.9	53.4	42.9
0	CC011	F	Y	61.4	32.4	12.9	28.8
1	CC011	F	Y	71.2	38.1	51	26.7
1	CC011	M	Y	68.6	30.8	51.7	55.3
1	CC011	F	Y	69.1	30.7	42.4	46.7
1	CC011	M	Y	66.3	25.3	42.3	20.6
2	CC011	F	Y	79.1	52.4	51	34.1
2	CC011	M	Y	81.2	49	66.5	32.4
2	CC011	F	Y	78.5	38.1	52.3	40.6
2	CC011	M	Y	59.9	22	46.4	31.8
0	CC012	M	Y	78.9	44	58.6	46.1
0	CC012	M	Y	74.7	40.3	38.4	30.1
0	CC012	M	Y	77.4	43.3	57.1	38.1
0	CC012	M	Y	77.7	48.3	57.4	37
0	CC012	M	Y	92.1	39.9	52.9	43.9
0	CC012	F	Y	73.5	38.7	54.2	28
0	CC012	F	Y	86.3	49.3	54.3	32.4
0	CC012	F	Y	67	40.5	45	36.8
0	CC012	F	Y	81	23.9	43.9	33.7
0	CC012	F	Y	80.9	42.9	46.3	33.9
2	CC012	M	Y	82.1	26.7	67.2	37.7
2	CC012	M	Y	85.7	37	52.6	40.5
2	CC012	M	Y	73.4	50	60.5	27.4
2	CC012	M	Y	68.3	49	59.6	29
2	CC012	M	Y	81.4	28	50.2	30.5
2	CC012	F	Y	68.5	42.6	39.1	37.8
2	CC012	F	Y	58	19.4	42.5	28.6
2	CC012	F	Y	66.7	46.7	42.7	34.6
2	CC012	F	Y	70.5	44.7	55.4	46.1
2	CC012	F	Y	49.7	44.4	43.4	43
1	CC012XCC032	F	Y	52.3	52.9	36	55
1	CC012xCC032	F	Y	46.2	33.7	33.8	34.7
1	CC012XCC032	M	Y	77.4	32.7	50.2	43.7
1	CC012xCC032	M	Y	79.2	32	55.7	32.6
1	CC012XCC032	M	Y	71.9	26.2	60.9	36.1
1	CC012xCC032	M	Y	66.8	49.5	33.2	49.3
2	CC012XCC032	F	Y	59.1	24.3	40.6	31.6
2	CC012XCC032	F	Y	45.1	32.5	44.8	52.9
2	CC012XCC032	M	Y	62.3	40	52	26.9
2	CC012XCC032	M	Y	64.4	27	48.7	39.3
2	CC012XCC032	M	Y	65.4	25.4	49.7	34.6
2	CC012XCC032	M	Y	56.3	24.6	58.3	25.6
1	CC013xCC041	F	Y	67.9	34.8	54	27.5
1	CC013xCC041	F	Y	56.7	32.7	43	32.5
1	CC013xCC041	F	Y	76.2	40.8	53.3	46.6

Trial#	Strain	Sex	Inf?	%PropelStance_RH	%PropelStride_LF	%PropelStride_LH	%PropelStride_RF
1	CC013xCC041	M	Y	56.1	34.8	67.1	36.8
1	CC013xCC041	M	Y	61.7	44.4	55.6	43.1
2	CC013XCC041	F	Y	86	38.5	45.8	35.5
2	CC013XCC041	F	Y	63.5	33.6	41.8	41.9
2	CC013XCC041	F	Y	86	18	63.3	20.1
2	CC013XCC041	M	Y	29.5	26.3	54.4	38.1
2	CC013XCC041	M	Y	60.5	39.5	67.9	40.3
0	CC015	F	Y	41.5	29.3	44.2	48.1
0	CC015	M	Y	67.7	32	54	39.5
0	CC015	M	Y	51.4	34.8	41.9	33.6
1	CC015	F	Y	54.8	40	45	38.5
1	CC015	M	Y	62.2	37.4	39.3	32
1	CC015	M	Y	54.6	36.9	30.6	31.6
1	CC015	M	Y	68	25.6	50.7	23.2
2	CC015	F	Y	51.5	33.5	35.7	31.6
0	CC017	F	Y	70.5	39.4	46.5	35.8
0	CC017	M	Y	75.9	42.6	59.7	30.2
0	CC017	F	Y	52.7	31.8	57.6	35.4
0	CC017	M	Y	71.1	21.6	47.4	31.9
1	CC017	M	Y	73.5	25.3	54.9	21.6
1	CC017	F	Y	75.7	32	43.1	30.5
0	CC023	M	Y	86.6	49.8	62.3	52.6
0	CC023	F	Y	51.3	42.9	59.9	34.5
0	CC023	F	Y	70.8	33.3	36	35.8
0	CC023	M	Y	62.4	14.8	73.3	19.4
1	CC023	M	Y	82.5	43.4	64.6	30.4
1	CC023	F	Y	70.1	32.2	58.7	31
1	CC023	M	Y	59.3	36.5	58	20.4
1	CC023	M	Y	59.3	36.3	47.3	31.2
2	CC023	M	Y	76.4	44.8	45.7	57.8
2	CC023	F	Y	49	28	63	40.6
2	CC023	M	Y	82.1	35.9	57.8	37.2
0	CC025	M	Y	64.5	48.3	48.8	40.8
0	CC025	F	Y	67.9	38.1	52.4	30.5
0	CC025	M	Y	81.3	37.2	70.3	40
1	CC025	M	Y	64.1	49.7	53.1	43.2
1	CC025	F	Y	72.7	31.7	51.8	31.2
1	CC025	F	Y	61.6	36.9	44.1	34.1
1	CC025	F	Y	67.9	48.2	50.2	42.8
2	CC025	F	Y	71	29	51.6	47.5
2	CC025	M	Y	85.8	39.1	49	35.2
0	CC027	F	Y	78.9	31	60.4	26.9
0	CC027	M	Y	87.2	39.4	62.6	25.8
1	CC027	M	Y	62	64.6	32.8	56.9
1	CC027	M	Y	62	64.6	32.8	56.9
1	CC027	F	Y	67.3	32.1	43.6	26.8
1	CC027	F	Y	66.6	30.6	39.5	30.7
1	CC027	M	Y	80.4	36.7	40.3	17.3

Trial#	Strain	Sex	Inf?	%PropelStance_RH	%PropelStride_LF	%PropelStride_LH	%PropelStride_RF
1	CC027	M	Y	73.4	31.4	57.2	37.2
2	CC027	F	Y	49	28	63	40.6
2	CC027	M	Y	71.6	38.7	46.9	42.7
2	CC027	F	Y	54.2	36.9	40.6	28.4
2	CC027	M	Y	69.5	28.2	59.2	30
1	CC032XCC013	M	Y	81	20	45.4	30.7
1	CC032XCC013	M	Y	79.7	34.7	56.8	45.3
1	CC032XCC013	M	Y	66.2	26	42.2	29.5
1	CC032XCC013	F	Y	74.5	38	41.4	44
1	CC032XCC013	F	Y	59.7	36.2	36.9	32.7
1	CC032XCC013	F	Y	63.5	25.6	47	37.1
1	CC032XCC013	F	Y	62.4	34.5	47.8	36.3
1	CC032XCC013	F	Y	73.6	42.3	36.4	48.4
2	CC032XCC013	M	Y	48.7	49	48.2	16.1
2	CC032XCC013	M	Y	62.8	26.8	58.4	16.7
2	CC032XCC013	M	Y	74.7	30.4	52.5	37
2	CC032XCC013	M	Y	62.3	32.4	37.2	19.2
2	CC032XCC013	F	Y	52.9	36.5	44.7	37.2
2	CC032XCC013	F	Y	58.8	25.3	40.6	39.7
2	CC032XCC013	F	Y	57	38.6	41.7	35.5
2	CC032XCC013	F	Y	72.3	37.5	55.4	47.3
0	CC037	F	Y	76.7	36.7	56.7	36.7
0	CC037	M	Y	70.5	34	47.4	37.2
0	CC037	M	Y	82.2	36.8	51.2	34.6
0	CC037	M	Y	49.6	29.9	28.5	44.7
1	CC037	F	Y	79.1	31.8	37.9	24.5
1	CC037	M	Y	63.5	38.5	47	43.9
1	CC037	M	Y	72.9	29.5	42.7	29.3
1	CC037	M	Y	71.6	41	41.8	40
2	CC037	M	Y	37.4	21.8	38	45.2
2	CC037	M	Y	51.7	46	45.8	42.3
1	CC041XCC012	M	Y	89.9	30.7	66.7	27.2
1	CC041XCC012	M	Y	73.9	24.1	58.8	25.5
1	CC041XCC012	M	Y	79.6	33.7	45.7	34.5
1	CC041XCC012	M	Y	81.4	28.9	49.1	40.2
1	CC041XCC012	M	Y	86.5	35	48.8	34.4
1	CC041XCC012	F	Y	60.9	32.5	41.6	32.8
1	CC041XCC012	F	Y	77.9	47.2	51.3	32.9
1	CC041XCC012	F	Y	77.8	38.7	56.4	42.1
1	CC041XCC012	F	Y	82.7	23.7	35	28.6
1	CC041XCC012	F	Y	81.8	38.1	55.8	26.7
1	CC041XCC012	F	Y	68.7	37.2	34.1	31.6
1	CC041XCC012	F	Y	62	27.8	47.5	38.9
1	CC041XCC012	F	Y	77.3	45.9	61.2	24.9
2	CC041XCC012	M	Y	66.1	35.7	48.8	31.7
2	CC041XCC012	M	Y	89.5	27.2	49.3	18.6
2	CC041XCC012	F	Y	86.2	34.4	52.5	34
2	CC041XCC012	M	Y	74.1	31.9	37.8	20.4

Trial#	Strain	Sex	Inf?	%PropelStance_RH	%PropelStride_LF	%PropelStride_LH	%PropelStride_RF
2	CC041XCC012	M	Y	77.5	36.8	49.3	35.1
2	CC041XCC012	M	Y	83.7	41	43.9	24.2
2	CC041XCC012	F	Y	77	31.8	42	29.5
2	CC041XCC012	F	Y	71.8	33.1	61.5	37.4
2	CC041XCC012	F	Y	57.5	38.2	42.9	23.2
2	CC041XCC012	F	Y	68.4	38.5	44.1	35.2
2	CC041XCC012	F	Y	63.1	40.5	45.6	36.5
2	CC041XCC012	F	Y	68.2	33.7	56.1	33.5
2	CC041XCC012	F	Y	84.8	36.2	63.7	30.1
0	CC051	F	Y	65.2	36.7	47.9	29
0	CC051	F	Y	57.9	45.9	60.3	35.4
0	CC051	F	Y	65.3	39.9	58.5	40.8
0	CC051	M	Y	51.3	34.8	54.3	39.4
1	CC051	F	Y	61	37.9	47.4	22.9
1	CC051	F	Y	56.8	42.9	46.6	30.8
1	CC051	F	Y	73.1	36	52	40.3
0	CC057	F	Y	78	38.3	46.7	49.4
0	CC057	F	Y	75.1	34.6	54.4	40.1
0	CC057	F	Y	68.2	35.9	46.4	52.6
0	CC057	M	Y	65.8	40.1	42.6	39.8
0	CC057	M	Y	68.2	34.1	25.7	36
0	CC057	M	Y	67.3	28.5	52.2	27
1	CC057	F	Y	78.4	42.2	61.1	48.8
1	CC057	F	Y	79.7	40	55	51.2
1	CC057	F	Y	65.9	41	64.3	31.5
1	CC057	F	Y	72	31	52.1	26.7
1	CC057	M	Y	74.5	29.9	63.4	40.4
1	CC057	M	Y	59.3	37.4	47.6	46.8
2	CC057	F	Y	72	31	52.1	26.7
0	CC078	F	Y	72.6	37.9	59.9	32.7
0	CC078	F	Y	79	46.4	43.2	46.8
0	CC078	F	Y	83.7	39.5	57.7	43.8
0	CC078	M	Y	61.3	41.6	47.6	46.3
1	CC078	F	Y	77.5	43.5	55.6	57.2
1	CC078	F	Y	87.8	38.3	66.4	48
1	CC078	M	Y	57.7	28.7	43.5	28.6
2	CC078	F	Y	68.6	53.1	49.6	34.2
2	CC078	M	Y	55.1	23.7	51.6	30.4
2	CC005	M	N	75.8	24.2	64.3	42.1
2	CC005	M	Y	53.6	26.7	51.3	35
2	CC015	F	N	62.7	32.5	39.5	42.6
2	CC015	M	N	54.9	25.8	47.2	31.5
2	CC015	M	Y	57.4	27.8	45.1	30.3
2	CC015	M	Y	56.2	37.1	36	40.1
2	CC017	F	N	73.4	29.2	44.6	38.3
2	CC017	M	N	67.7	31.4	45.9	29.3
2	CC017	F	Y	87.6	19.9	53.5	32.9
2	CC023	F	N	71.4	20.9	43.8	24.3

Trial#	Strain	Sex	Inf?	%PropelStance_RH	%PropelStride_LF	%PropelStride_LH	%PropelStride_RF

Table S3. Raw DigiGait measurement data. Far left column indicates time point at which data was measured: T0 = pre-infection, T1 = 21dpi, and T3 = 89dpi. DigiGait parameters listed across the top indicate which limb is associated with the data, where appropriate: FL for left fore limb, FR for right fore limb, HL for left hind limb, and HR for right hind limb.

Table S3

Trial#	Strain	Sex	Inf?	%PropelStride_RH	%SharedStance_LH	%SharedStance_RH	%StanceStride_LF
1	CC002	F	N	56.9	73.9	50.9	69.9
1	CC025	F	N	48	74	90	68.8
1	CC012XCC032	F	N	56.7	59.4	70.6	66
2	CC012XCC032	F	N	47.2	59.7	56	65.1
1	CC012XCC032	M	N	79.2	100.5	99.8	71.4
2	CC012XCC032	M	N	77.3	90.3	85.7	67
1	CC013xCC041	F	N	52.5	70.1	49	70.5
2	CC013XCC041	F	N	52.8	77.8	73.6	76.8
1	CC013xCC041	M	N	32.5	75.3	150.5	48.3
2	CC013XCC041	M	N	51.6	67.3	63.2	69.5
1	CC032XCC013	F	N	43.8	70.7	104.9	75.8
2	CC032XCC013	F	N	56.6	98.9	75.1	71.3
1	CC041XCC012	F	N	46.1	66.6	60	77.2
1	CC041XCC012	F	N	46.3	79.2	56.5	72.8
2	CC041XCC012	F	N	49.1	68	98.2	61.5
1	CC032XCC013	M	N	36.7	49.5	63.8	77.8
2	CC032XCC013	M	N	40.7	38.9	107.9	50
1	CC041XCC012	M	N	56	59.5	82	65.3
2	CC041XCC012	M	N	55.8	71.5	81	72
0	CC012	F	N	49.2	60.2	54.1	65.7
2	CC012	F	N	55.2	46.9	58.3	68.5
0	CC012	M	N	60.3	80.5	58.9	56.4
2	CC012	M	N	58.7	100.4	81.9	73.4
0	CC057	F	N	45.9	72	115.9	70.5
1	CC057	F	N	48.9	66	63.7	72.2
0	CC057	M	N	52.9	60.8	66.5	74.8
1	CC057	M	N	51.1	67.3	64.9	69.4
0	CC078	F	N	60.8	64	62.7	64.6
1	CC078	F	N	61.3	63.9	63.5	66.1
2	CC078	F	N	35.6	54.1	82.5	64.3
0	CC078	M	N	51.7	43.6	40.8	58.9
2	CC078	M	N	43.1	56.2	59.8	60.5
0	CC002	F	N	34.7	57.9	80.2	70.2
1	CC002	F	N	56.9	73.9	50.9	69.9
0	CC002	M	N	42.6	51.7	98.4	62.5
1	CC002	M	N	55.2	92.4	67	72.9
2	CC002	M	N	57.5	85.1	122.5	65.7
0	CC006	F	N	57.8	51	54.8	60.2
1	CC006	F	N	59.1	99.8	83	66.3
2	CC006	F	N	45.2	59.8	84.8	69.3
0	CC006	M	N	35	43.1	109.4	74.6
0	CC023	F	N	52.2	86.2	63.8	68.3
1	CC023	F	N	46	53.6	57.5	65.2
2	CC023	F	N	46.8	51.9	67	64
0	CC023	M	N	59.2	57.7	73.6	66
1	CC023	M	N	49.5	61.6	63.1	62.8
0	CC027	F	N	28.9	22.2	17.1	40.8

Trial#	Strain	Sex	Inf?	%PropelStride_RH	%SharedStance_LH	%SharedStance_RH	%StanceStride_LF
1	CC027	F	N	33.7	91.7	80.2	70.6
2	CC027	F	N	57.8	66.5	87.8	65.7
0	CC027	M	N	14.7	37.9	22.8	51
1	CC027	M	N	44.5	87.6	108	60.5
1	CC027	M	N	38.1	74.6	101.2	82.6
2	CC027	M	N	37.6	51.8	56.1	63.1
0	CC005	F	N	64.6	72.5	72.6	63.9
1	CC005	F	N	47.8	66.8	70.7	67.3
2	CC005	F	N	32.5	50.5	46.6	67
0	CC011	F	N	71.7	89.1	72.4	57.2
1	CC011	F	N	63.8	74.5	83.6	58.4
2	CC011	F	N	62.6	70	69	67
1	CC017	F	N	60.4	73.8	80.3	72.2
0	CC005	M	N	60.8	69.9	70.8	66.4
1	CC005	M	N	64.6	137.6	64.9	66.9
2	CC005	M	N	68.4	97.5	73.4	70.3
0	CC011	M	N	60.7	69	77.6	64.9
1	CC011	M	N	58.3	72.4	70.3	60.9
2	CC011	M	N	64.1	74.6	85.1	69
0	CC017	M	N	22	33	31.7	56.4
1	CC017	M	N	56.9	65.7	90.4	61.2
1	CC006	F	N	20.9	44.2	24.3	58.2
0	CC037	F	N	32	51.9	67.8	58.7
0	CC051	F	N	49.9	68.1	68.9	63.4
1	CC051	F	N	48.1	103.7	78.9	64.1
1	CC006	M	N	44.4	62.2	57	62.2
0	CC037	M	N	55.2	58.6	58.2	59.8
1	CC037	M	N	52.2	75	69	62.7
0	CC005	F	N	62.9	61.1	68.4	64.5
1	CC005	F	N	61.1	70.7	71.5	75.4
2	CC005	F	N	37.5	27.3	34.1	64.1
0	CC011	F	N	57.1	68.4	70.7	68.4
1	CC011	F	N	51.3	65.2	68.3	66.3
2	CC011	F	N	43.1	74.4	72.2	76.6
0	CC011	M	N	44.7	64	60.8	63.6
1	CC011	M	N	40	61.7	61.4	62.6
2	CC011	M	N	39.8	59.2	61.1	62
1	CC037	M	N	49.1	74.6	75.5	70.2
2	CC037	M	N	33.8	95	76.4	56.9
1	CC051	M	N	56.3	80.8	74.3	65.2
2	CC051	M	N	56.5	73.9	58.5	62.5
0	CC027	F	N	45.8	67.2	76.4	56.8
1	CC027	F	N	39.9	49.9	57	65.9
2	CC027	F	N	38	46.5	59.3	71.4
0	CC015	M	N	44.3	54.3	52.5	74.7
1	CC015	M	N	23.2	52.1	84.2	54.3
2	CC015	M	N	49.7	48.1	55	68.7
0	CC027	M	N	42.1	71.5	66.3	69

Trial#	Strain	Sex	Inf?	%PropelStride_RH	%SharedStance_LH	%SharedStance_RH	%StanceStride_LF
1	CC027	M	N	47	58.9	74.1	58.5
2	CC027	M	N	58.2	76.6	57	61.1
0	CC015	F	N	41.4	78.8	69.3	71.7
1	CC015	F	N	39.8	58.1	68.5	73
0	CC017	F	N	38.8	50.5	53.9	59.8
0	CC023	F	N	32.8	67.7	65.1	64.8
1	CC023	F	N	44.8	57.4	59.7	66.4
0	CC005	M	N	25.8	50.9	65.1	65.8
1	CC005	M	N	32.6	32.8	56.4	71
0	CC015	M	N	43.6	71	70.6	65.9
1	CC015	M	N	41.9	52.6	160.5	72.6
0	CC017	M	N	43.4	75.9	74.2	72.4
1	CC017	M	N	40.7	95.3	76.8	68.2
0	CC023	M	N	44.3	61.6	60.8	60.2
1	CC023	M	N	63	76.8	68.9	62.1
0	CC051	M	N	46.5	65.5	77.3	68.8
0	CC002	F	Y	36.8	63.8	83.9	58
0	CC002	M	Y	40.2	65.1	102.7	66.9
1	CC002	M	Y	8.6	6.9	13.2	61.1
1	CC002	F	Y	26.3	71	101.6	65.2
1	CC002	F	Y	49.7	63.7	76.7	67.4
1	CC002	M	Y	8.6	6.9	13.2	61.1
1	CC002	M	Y	43.5	64.4	88.1	74.7
1	CC002	F	Y	26.3	71	101.6	65.2
1	CC002	F	Y	33.2	41.4	38.7	73.8
2	CC002	M	Y	46.9	56.8	96.2	64.2
2	CC002	F	Y	49.6	67.9	62.2	66.5
2	CC002	M	Y	33.7	76.5	129.6	73.5
0	CC005	F	Y	61.3	65.4	68	58.1
0	CC005	M	Y	61.6	68.9	65.9	65.9
0	CC005	M	Y	53.2	67.1	66.7	73.3
1	CC005	F	Y	53.1	62	76.6	62.1
1	CC005	M	Y	15	23.9	92.5	72.7
1	CC005	F	Y	38.3	22.4	31	64.1
1	CC005	M	Y	59.1	107.8	72.3	66.2
2	CC005	F	Y	60.7	101.4	91	58
2	CC005	M	Y	54.4	65.3	75	68
2	CC005	M	Y	23.6	45.6	117.7	63.8
2	CC005	M	Y	54.4	65.3	75	68
2	CC005	F	Y	30.2	40.8	100.2	68
0	CC006	F	Y	55.6	58.8	57.8	64.7
0	CC006	M	Y	17.3	22.9	73.9	69.6
0	CC006	F	Y	56.5	68.2	65.4	65.6
1	CC006	F	Y	43.1	88.7	77.1	64
1	CC006	M	Y	42.2	79.9	64.6	79.2
1	CC006	F	Y	62.8	56.9	58.2	64.9
1	CC006	M	Y	55.7	71.2	64.4	63.5
2	CC006	F	Y	63	65.3	57.6	59.3

Trial#	Strain	Sex	Inf?	%PropelStride_RH	%SharedStance_LH	%SharedStance_RH	%StanceStride_LF
2	CC006	M	Y	45.8	62	53.5	73.1
0	CC011	F	Y	76.8	105.4	79.2	51.5
0	CC011	M	Y	55	75	81.2	70.4
0	CC011	F	Y	54.6	58.4	58.6	61.5
0	CC011	F	Y	24.7	36.6	24.6	68.2
1	CC011	F	Y	50.7	64	67.7	67.2
1	CC011	M	Y	50.4	75.2	84.3	72
1	CC011	F	Y	42.8	52.4	60.8	70.1
1	CC011	M	Y	48.1	70.6	64.9	64
2	CC011	F	Y	56.8	68	67.5	68.6
2	CC011	M	Y	65.5	106.8	79.4	76.5
2	CC011	F	Y	57.6	66.4	69.3	73.8
2	CC011	M	Y	40.2	53.5	58.6	63.1
0	CC012	M	Y	52.9	58.3	69.6	63.6
0	CC012	M	Y	49.6	58.3	55.3	67.2
0	CC012	M	Y	53.2	64.1	64.1	65.3
0	CC012	M	Y	55.6	71.2	69.4	65.6
0	CC012	M	Y	66.1	60.5	72.3	68.5
0	CC012	F	Y	56.2	83.7	71.2	62.1
0	CC012	F	Y	61.5	62.1	66	68.6
0	CC012	F	Y	47.7	59.9	47	73.7
0	CC012	F	Y	46.8	53.8	51.9	57
0	CC012	F	Y	46.4	25.1	30.1	62.6
2	CC012	M	Y	57.1	55.1	61	67.1
2	CC012	M	Y	56.5	48.9	47.6	68.6
2	CC012	M	Y	49.1	60.8	59.7	63.9
2	CC012	M	Y	52.9	71.3	67.2	71.5
2	CC012	M	Y	54.6	50	52.4	63
2	CC012	F	Y	52.1	85	89.9	65.7
2	CC012	F	Y	36.5	81.4	82.5	60.3
2	CC012	F	Y	51.2	81.3	104.9	66.9
2	CC012	F	Y	47.1	58.5	69.4	65.6
2	CC012	F	Y	36.6	67.3	67.3	70.3
1	CC012XCC032	F	Y	32.1	48.8	68.2	78.1
1	CC012xCC032	F	Y	24.7	41.2	53.2	70.6
1	CC012XCC032	M	Y	54.7	60.1	60.2	69.9
1	CC012xCC032	M	Y	55	85.5	75.8	61.4
1	CC012XCC032	M	Y	41.1	71.9	120.4	58.2
1	CC012xCC032	M	Y	48.6	76.6	99.1	78.7
2	CC012XCC032	F	Y	42.7	66.5	54.8	72.3
2	CC012XCC032	F	Y	32	65.8	55.2	70.9
2	CC012XCC032	M	Y	44.6	80.9	120.8	67.5
2	CC012XCC032	M	Y	40.3	54.9	50.9	56.3
2	CC012XCC032	M	Y	49.6	78.7	79.2	67.5
2	CC012XCC032	M	Y	44	87.7	54.3	69.1
1	CC013xCC041	F	Y	47.8	65.3	76.7	69.1
1	CC013xCC041	F	Y	34.6	61.9	57.8	69.5
1	CC013xCC041	F	Y	60.2	80.9	75.9	68.4

Trial#	Strain	Sex	Inf?	%PropelStride_RH	%SharedStance_LH	%SharedStance_RH	%StanceStride_LF
1	CC013xCC041	M	Y	41.4	78.2	74.5	72.9
1	CC013xCC041	M	Y	44.4	83.1	187.4	72.5
2	CC013XCC041	F	Y	64	74.1	71.8	69.4
2	CC013XCC041	F	Y	52.3	114.5	70.2	71.2
2	CC013XCC041	F	Y	65.8	73	65.5	66.1
2	CC013XCC041	M	Y	22.8	83.5	103	73.2
2	CC013XCC041	M	Y	39.3	91.7	158.7	72
0	CC015	F	Y	30.1	69.2	56.6	67.4
0	CC015	M	Y	50.5	71.1	72.6	67.1
0	CC015	M	Y	38.4	70.5	80.6	69
1	CC015	F	Y	33.5	51.3	62.6	68.5
1	CC015	M	Y	38.3	65	67.2	72.9
1	CC015	M	Y	39.5	71.9	59.3	75.8
1	CC015	M	Y	52.6	68.5	83.5	68.6
2	CC015	F	Y	37	66.6	68.2	71.9
0	CC017	F	Y	44.5	52.2	64	57.7
0	CC017	M	Y	58.4	98.4	62.8	70.1
0	CC017	F	Y	41.6	84	92.3	66.6
0	CC017	M	Y	47.2	58.4	76.3	64.3
1	CC017	M	Y	55.2	74.4	66.4	58
1	CC017	F	Y	54.1	62.5	57.8	64.8
0	CC023	M	Y	55.1	55.3	59.6	58.2
0	CC023	F	Y	30.7	46.4	122.3	62.1
0	CC023	F	Y	40.5	42	43.8	60.2
0	CC023	M	Y	38.3	64.3	85.4	33.8
1	CC023	M	Y	70.5	112.5	82.2	70.6
1	CC023	F	Y	60.5	117.5	72.4	60.5
1	CC023	M	Y	40.4	76.7	129.4	74.1
1	CC023	M	Y	41	70.7	62.9	66.8
2	CC023	M	Y	50.6	84.2	55.7	82
2	CC023	F	Y	37.8	81.9	73.9	71.4
2	CC023	M	Y	62.1	85.1	109.3	61.8
0	CC025	M	Y	43.8	64.7	75.1	67.1
0	CC025	F	Y	48.7	66.7	74.4	60.6
0	CC025	M	Y	49	76.8	83.6	69.4
1	CC025	M	Y	43.7	59.4	74.2	67.4
1	CC025	F	Y	47.6	68.4	64.2	51.8
1	CC025	F	Y	42.4	71.6	76.7	63.8
1	CC025	F	Y	43.1	50.2	67.5	66.1
2	CC025	F	Y	45.5	48.6	56.6	70.8
2	CC025	M	Y	61	66.3	71.7	66.2
0	CC027	F	Y	48	55.5	64.8	66.1
0	CC027	M	Y	57.2	72.4	56.6	62.4
1	CC027	M	Y	46.8	146.7	185.9	90.1
1	CC027	M	Y	46.8	146.7	185.9	90.1
1	CC027	F	Y	44	70.4	89.3	53.2
1	CC027	F	Y	32.4	36.2	84.4	60.4
1	CC027	M	Y	66.1	114.4	109.3	72.7

Trial#	Strain	Sex	Inf?	%PropelStride_RH	%SharedStance_LH	%SharedStance_RH	%StanceStride_LF
1	CC027	M	Y	53.9	74.1	78.4	71.3
2	CC027	F	Y	37.8	81.9	73.9	71.4
2	CC027	M	Y	47.7	96	58.1	64.4
2	CC027	F	Y	32.9	48.8	66	69.6
2	CC027	M	Y	53.1	78	76.2	64.2
1	CC032XCC013	M	Y	59.8	62.6	66.7	68
1	CC032XCC013	M	Y	54.5	62.7	73.2	72.5
1	CC032XCC013	M	Y	35.5	60	62.8	54.7
1	CC032XCC013	F	Y	53.3	89.9	80	64.2
1	CC032XCC013	F	Y	38.6	57.7	60.3	71.8
1	CC032XCC013	F	Y	41.7	69.5	79.2	69.5
1	CC032XCC013	F	Y	40.6	53	69.7	74.2
1	CC032XCC013	F	Y	54.2	114.9	94.2	69.3
2	CC032XCC013	M	Y	37.5	88.9	86.7	74.7
2	CC032XCC013	M	Y	47.7	78.3	96.6	66.4
2	CC032XCC013	M	Y	49.8	64.9	89.4	49.6
2	CC032XCC013	M	Y	46.5	58.5	72.5	67.6
2	CC032XCC013	F	Y	37.8	70.8	80.3	70.6
2	CC032XCC013	F	Y	43	62.7	62.1	67.8
2	CC032XCC013	F	Y	38.8	64.8	62.7	74.8
2	CC032XCC013	F	Y	49.5	63.1	69.7	68.6
0	CC037	F	Y	58.7	85.1	66.7	64.9
0	CC037	M	Y	56	85.4	55.7	56.4
0	CC037	M	Y	38.5	26.9	39.7	64.6
0	CC037	M	Y	18	12.5	22.6	57.2
1	CC037	F	Y	58.4	73.1	77.3	68.2
1	CC037	M	Y	42	44.3	42.4	64.7
1	CC037	M	Y	49	61.2	64.9	69.6
1	CC037	M	Y	50.3	58.5	78.4	66.5
2	CC037	M	Y	26.9	69.2	75.8	69.9
2	CC037	M	Y	28.9	39.6	50.2	66.6
1	CC041XCC012	M	Y	69.4	106	78.3	76.5
1	CC041XCC012	M	Y	56.6	69.5	72.4	58.5
1	CC041XCC012	M	Y	57.9	53.9	55.8	74.9
1	CC041XCC012	M	Y	55.8	56.2	63.6	74.5
1	CC041XCC012	M	Y	65.9	68.9	66.9	70.4
1	CC041XCC012	F	Y	44.6	73.3	89.4	72.5
1	CC041XCC012	F	Y	57.6	80.8	91.1	67.2
1	CC041XCC012	F	Y	61.2	74.8	81.8	71.8
1	CC041XCC012	F	Y	59.8	65.1	71.8	72.4
1	CC041XCC012	F	Y	57.4	69.6	80.9	74.5
1	CC041XCC012	F	Y	46.1	58.9	66.4	67.1
1	CC041XCC012	F	Y	45.5	74.9	69.2	60.3
1	CC041XCC012	F	Y	60.3	80	94.9	78.2
2	CC041XCC012	M	Y	47.6	90.3	98.2	72
2	CC041XCC012	M	Y	67.5	70.4	66.5	64.5
2	CC041XCC012	F	Y	53.5	51.1	57.1	63.1
2	CC041XCC012	M	Y	58.4	78.3	41.9	76.2

Trial#	Strain	Sex	Inf?	%PropelStride_RH	%SharedStance_LH	%SharedStance_RH	%StanceStride_LF
2	CC041XCC012	M	Y	59.6	74.3	68.8	67.6
2	CC041XCC012	M	Y	60.4	51.3	30.7	70.8
2	CC041XCC012	F	Y	59.1	76	70.8	69.1
2	CC041XCC012	F	Y	54	73	77.1	72.9
2	CC041XCC012	F	Y	41.2	73.6	84.4	70.3
2	CC041XCC012	F	Y	47.3	55.3	54.4	67.8
2	CC041XCC012	F	Y	49.5	68.1	68.3	70.2
2	CC041XCC012	F	Y	54.6	78.3	80	71.6
2	CC041XCC012	F	Y	62.5	64.1	64.1	65.5
0	CC051	F	Y	48.8	68	69.3	66.4
0	CC051	F	Y	45.3	72.3	76.9	69
0	CC051	F	Y	47.9	69.2	65	64.5
0	CC051	M	Y	38.5	65.9	68.4	66.4
1	CC051	F	Y	46.9	67.5	66.6	66.4
1	CC051	F	Y	42.6	72.1	72.8	72.1
1	CC051	F	Y	56.5	74.2	89.7	66.3
0	CC057	F	Y	58.2	66.5	67.4	70.9
0	CC057	F	Y	51.5	58.5	60.2	65.8
0	CC057	F	Y	49.5	74.3	95.2	74.2
0	CC057	M	Y	51.9	81.4	82.8	74.3
0	CC057	M	Y	43.6	53.9	54.9	77.3
0	CC057	M	Y	51.1	67.4	81.9	75.5
1	CC057	F	Y	59.3	68.5	58	77.9
1	CC057	F	Y	53.4	56.2	66.4	69.8
1	CC057	F	Y	40.4	56.5	73.3	72.8
1	CC057	F	Y	50.7	68.6	65.2	75.2
1	CC057	M	Y	55.2	74	58.8	71.4
1	CC057	M	Y	43.9	80.6	79	76.9
2	CC057	F	Y	50.7	68.6	65.2	75.2
0	CC078	F	Y	49.7	50.5	60.5	59.5
0	CC078	F	Y	53.6	55.5	50.8	65.8
0	CC078	F	Y	56.7	57.6	57.7	64.3
0	CC078	M	Y	42.5	59.1	51.2	68.1
1	CC078	F	Y	53.2	64.3	69.3	66.1
1	CC078	F	Y	50	42.5	61.9	72.8
1	CC078	M	Y	39.4	47.8	40.1	62.8
2	CC078	F	Y	46.6	55.7	69.4	67.6
2	CC078	M	Y	39.6	58	54.6	61.9
2	CC005	M	N	54.3	67.6	88.2	62.8
2	CC005	M	Y	34.2	62.4	53.8	49.5
2	CC015	F	N	44.4	59.8	53.5	69.5
2	CC015	M	N	39.3	65.1	68.9	67.6
2	CC015	M	Y	43.2	67.2	78.6	74.7
2	CC015	M	Y	42.6	65.9	79.5	75.8
2	CC017	F	N	53	60.2	66.2	69.3
2	CC017	M	N	43	80.4	72.7	54.1
2	CC017	F	Y	59	61.7	55.9	75.1
2	CC023	F	N	42.7	43.1	47.4	67.7

Trial#	Strain	Sex	Inf?	%PropelStride_RH	%SharedStance_LH	%SharedStance_RH	%StanceStride_LF

Table S3. Raw DigiGait measurement data. Far left column indicates time point at which data was measured: T0 = pre-infection, T1 = 21dpi, and T3 = 89dpi. DigiGait parameters listed across the top indicate which limb is associated with the data, where appropriate: FL for left fore limb, FR for right fore limb, HL for left hind limb, and HR for right hind limb.

Table S3

Trial#	Strain	Sex	Inf?	%StanceStride_LH	%StanceStride_RF	%StanceStride_RH	%SwingStride_LF
1	CC002	F	N	72.1	64.6	73.8	30.1
1	CC025	F	N	77.8	69.6	74.4	31.2
1	CC012XCC032	F	N	72.3	75.3	69.9	34
2	CC012XCC032	F	N	66.4	65.9	73.5	34.9
1	CC012XCC032	M	N	84.2	74.7	82.9	28.6
2	CC012XCC032	M	N	82.5	66.3	84.8	33
1	CC013xCC041	F	N	65.9	69.2	74.7	29.5
2	CC013XCC041	F	N	74.1	75.3	78	23.2
1	CC013xCC041	M	N	65.4	67	64.4	51.7
2	CC013XCC041	M	N	76.3	70	76.2	30.5
1	CC032XCC013	F	N	67.9	66.2	75.9	24.2
2	CC032XCC013	F	N	78.4	66.1	79.3	28.7
1	CC041XCC012	F	N	66.9	74.3	68.4	22.8
1	CC041XCC012	F	N	67.8	66	68.6	27.2
2	CC041XCC012	F	N	76.2	52.1	71.9	38.5
1	CC032XCC013	M	N	65.8	72.3	63	22.2
2	CC032XCC013	M	N	78	58.8	59.1	50
1	CC041XCC012	M	N	71.4	65.2	70.7	34.7
2	CC041XCC012	M	N	74.8	71	66.6	28
0	CC012	F	N	68.7	69.6	71.8	34.3
2	CC012	F	N	71.5	67.5	65.1	31.5
0	CC012	M	N	70.5	58.3	73.4	43.6
2	CC012	M	N	77.2	70.5	82.6	26.6
0	CC057	F	N	74	70	72.1	29.5
1	CC057	F	N	73.2	72.3	73.7	27.8
0	CC057	M	N	73.6	75.7	76.3	25.2
1	CC057	M	N	71.4	75.3	73.9	30.6
0	CC078	F	N	72.6	65.8	72.4	35.4
1	CC078	F	N	68.4	64.4	70.2	33.9
2	CC078	F	N	80.9	59.6	63.5	35.7
0	CC078	M	N	61.8	62	65	41.1
2	CC078	M	N	69.8	62.8	68	39.5
0	CC002	F	N	67.3	67.3	63.6	29.8
1	CC002	F	N	72.1	64.6	73.8	30.1
0	CC002	M	N	72.2	69.4	61	37.5
1	CC002	M	N	75.5	86.8	70.9	27.1
2	CC002	M	N	76.6	65.9	76.3	34.3
0	CC006	F	N	70.5	63.2	66.9	39.8
1	CC006	F	N	72.5	71.4	69.6	33.7
2	CC006	F	N	72.8	68	63.6	30.7
0	CC006	M	N	76.7	69.6	53.6	25.4
0	CC023	F	N	70.5	66.2	73.9	31.7
1	CC023	F	N	67.3	51.9	64.9	34.8
2	CC023	F	N	72.5	63.8	65.1	36
0	CC023	M	N	74.1	64.3	66.3	34
1	CC023	M	N	73.4	67.7	71.4	37.2
0	CC027	F	N	29.6	52.6	44.4	59.2

Trial#	Strain	Sex	Inf?	%StanceStride_LH	%StanceStride_RF	%StanceStride_RH	%SwingStride_LF
1	CC027	F	N	72	73.4	68.8	29.4
2	CC027	F	N	72	71.2	69.8	34.3
0	CC027	M	N	43.5	65.1	41.5	49
1	CC027	M	N	73.6	45.9	73.6	39.5
1	CC027	M	N	86.2	72.4	74.8	17.4
2	CC027	M	N	64.5	61	62.4	36.9
0	CC005	F	N	73.5	61	73.9	36.1
1	CC005	F	N	75.2	68.5	76.4	32.7
2	CC005	F	N	58	78.9	61.6	33
0	CC011	F	N	70.7	61.3	77.2	42.8
1	CC011	F	N	80	56.6	79	41.6
2	CC011	F	N	73.6	65.4	75.6	33
1	CC017	F	N	75.2	74.9	79.4	27.8
0	CC005	M	N	73.6	65.9	73.6	33.6
1	CC005	M	N	65.4	64	75.5	33.1
2	CC005	M	N	77.2	62.9	84.8	29.7
0	CC011	M	N	74.1	67.9	71.1	35.1
1	CC011	M	N	72.7	71.9	72.4	39.1
2	CC011	M	N	78.3	68.3	79.8	31
0	CC017	M	N	45.1	62.1	41.6	43.6
1	CC017	M	N	74.1	76.8	69.2	38.8
1	CC006	F	N	37	51.6	53.7	41.8
0	CC037	F	N	72.6	61.8	60	41.3
0	CC051	F	N	73.1	61.9	77	36.6
1	CC051	F	N	74.9	70.2	77	35.9
1	CC006	M	N	65.3	60.8	67.6	37.8
0	CC037	M	N	70.3	62.2	70.8	40.2
1	CC037	M	N	65.5	70.2	78	37.3
0	CC005	F	N	76.9	70.2	72	35.5
1	CC005	F	N	76.8	75.2	74.2	24.6
2	CC005	F	N	65	45.5	49.5	35.9
0	CC011	F	N	75.2	70.8	74.3	31.6
1	CC011	F	N	76	60	75.2	33.7
2	CC011	F	N	76.7	74.5	79.1	23.4
0	CC011	M	N	70.6	65	72.8	36.4
1	CC011	M	N	70.7	60.4	70.3	37.4
2	CC011	M	N	74.2	63.5	68.9	38
1	CC037	M	N	76.3	65.3	77.6	29.8
2	CC037	M	N	78.5	47.1	82.3	43.1
1	CC051	M	N	73.4	67.3	76.9	34.8
2	CC051	M	N	64.6	71.9	77	37.5
0	CC027	F	N	77.4	67.5	68.9	43.2
1	CC027	F	N	70.8	60.9	59.5	34.1
2	CC027	F	N	76.8	71.7	56.9	28.6
0	CC015	M	N	66.5	68.4	68.6	25.3
1	CC015	M	N	50.8	69.2	42.6	45.7
2	CC015	M	N	68.4	68.4	59.6	31.3
0	CC027	M	N	73	71.3	68.5	31

Trial#	Strain	Sex	Inf?	%StanceStride_LH	%StanceStride_RF	%StanceStride_RH	%SwingStride_LF
1	CC027	M	N	71.9	64.4	70.1	41.5
2	CC027	M	N	61.9	64.2	72	38.9
0	CC015	F	N	64.4	78.3	24	28.3
1	CC015	F	N	67.4	71.5	63.8	27
0	CC017	F	N	64.1	66.2	61.4	40.2
0	CC023	F	N	72	69.3	74.1	35.2
1	CC023	F	N	72.1	67.6	66.4	33.6
0	CC005	M	N	70.3	63.7	51.1	34.2
1	CC005	M	N	77.3	76.8	45.9	29
0	CC015	M	N	74.8	67.7	74.7	34.1
1	CC015	M	N	73.4	73	63.1	27.4
0	CC017	M	N	81	70.1	76.9	27.6
1	CC017	M	N	68.2	67.7	72.8	31.8
0	CC023	M	N	71	64.1	71.3	39.8
1	CC023	M	N	70.4	67.3	75.9	37.9
0	CC051	M	N	76.5	64.8	74.7	31.2
0	CC002	F	Y	80.6	66.5	71.9	42
0	CC002	M	Y	76.3	72.1	68.7	33.1
1	CC002	M	Y	51.3	65.9	15.4	38.9
1	CC002	F	Y	48.8	71.7	54	34.8
1	CC002	F	Y	73	69.6	69.4	32.6
1	CC002	M	Y	51.3	65.9	15.4	38.9
1	CC002	M	Y	73.1	73.7	70.5	25.3
1	CC002	F	Y	48.8	71.7	54	34.8
1	CC002	F	Y	47.2	68.8	49.1	26.2
2	CC002	M	Y	65.1	63.2	72	35.8
2	CC002	F	Y	74.2	70.5	74.4	33.5
2	CC002	M	Y	74.5	72.7	72.8	26.5
0	CC005	F	Y	75.9	58.3	73.3	41.9
0	CC005	M	Y	72.4	64.5	75.9	34.1
0	CC005	M	Y	75.7	67.4	76.9	26.7
1	CC005	F	Y	75.2	61.6	67.4	37.9
1	CC005	M	Y	63.7	75.9	25.9	27.3
1	CC005	F	Y	65.2	69.1	46.2	35.9
1	CC005	M	Y	69.5	63.9	73.5	33.8
2	CC005	F	Y	77.3	67.1	80.8	42
2	CC005	M	Y	77.3	74	71.9	32
2	CC005	M	Y	79.9	65.3	44.2	36.2
2	CC005	M	Y	77.3	74	71.9	32
2	CC005	F	Y	78.3	76.7	47.6	32
0	CC006	F	Y	71	63.8	73.4	35.3
0	CC006	M	Y	58.7	56.6	27.3	30.4
0	CC006	F	Y	74	60.8	77.4	34.4
1	CC006	F	Y	73.6	57.6	76.5	36
1	CC006	M	Y	70.1	68.7	75.9	20.8
1	CC006	F	Y	68.9	60	70	35.1
1	CC006	M	Y	68.6	68.6	73.8	36.5
2	CC006	F	Y	63	66.8	71.4	40.7

Trial#	Strain	Sex	Inf?	%StanceStride_LH	%StanceStride_RF	%StanceStride_RH	%SwingStride_LF
2	CC006	M	Y	68	75.4	62.2	26.9
0	CC011	F	Y	79	57.6	79.5	48.5
0	CC011	M	Y	76.2	69.4	73.4	29.6
0	CC011	F	Y	66.9	64.7	65.6	38.5
0	CC011	F	Y	23.3	57.3	40.3	31.8
1	CC011	F	Y	73.4	68	71.2	32.8
1	CC011	M	Y	75.5	78.8	73.5	28
1	CC011	F	Y	70.2	72.2	61.8	29.9
1	CC011	M	Y	72	71.4	72.5	36
2	CC011	F	Y	72	66.2	71.8	31.4
2	CC011	M	Y	79.6	75.4	80.7	23.5
2	CC011	F	Y	78.1	74	73.4	26.2
2	CC011	M	Y	70.9	64.3	67.1	36.9
0	CC012	M	Y	71.2	63.7	67.1	36.4
0	CC012	M	Y	63	64.4	66.4	32.8
0	CC012	M	Y	68.1	61.7	68.7	34.7
0	CC012	M	Y	66.4	60.7	71.6	34.4
0	CC012	M	Y	66.7	66.2	71.8	31.5
0	CC012	F	Y	72.5	54.8	76.5	37.9
0	CC012	F	Y	73.5	66.2	71.3	31.4
0	CC012	F	Y	63.9	66.7	71.3	26.3
0	CC012	F	Y	60.5	59.8	57.8	43
0	CC012	F	Y	54.4	62.8	57.4	37.4
2	CC012	M	Y	72.3	62.8	69.5	32.9
2	CC012	M	Y	63.9	61.8	65.9	31.4
2	CC012	M	Y	69.1	63.3	66.9	36.1
2	CC012	M	Y	72.6	67.4	77.4	28.5
2	CC012	M	Y	69.4	64.9	67.1	37
2	CC012	F	Y	76.1	60.8	76	34.3
2	CC012	F	Y	66	68.9	63	39.7
2	CC012	F	Y	70.4	65.2	76.7	33.1
2	CC012	F	Y	70.2	73.3	66.9	34.4
2	CC012	F	Y	71.4	73	73.7	29.7
1	CC012XCC032	F	Y	65.2	73.7	61.4	21.9
1	CC012xCC032	F	Y	60.4	66.1	53.3	29.4
1	CC012XCC032	M	Y	73.9	69.4	70.7	30.1
1	CC012xCC032	M	Y	67.8	64.2	69.4	38.6
1	CC012XCC032	M	Y	69.4	71.7	57.2	41.8
1	CC012xCC032	M	Y	72.7	82	72.8	21.3
2	CC012XCC032	F	Y	69.7	62.7	72.3	27.7
2	CC012XCC032	F	Y	69.1	74.4	70.9	29.1
2	CC012XCC032	M	Y	74.2	57.3	71.6	32.5
2	CC012XCC032	M	Y	67	65.8	62.6	43.7
2	CC012XCC032	M	Y	76.9	60	75.8	32.5
2	CC012XCC032	M	Y	63	68.7	78.1	30.9
1	CC013xCC041	F	Y	66.1	70.1	70.3	30.9
1	CC013xCC041	F	Y	66.2	68.2	61.1	30.5
1	CC013xCC041	F	Y	77.5	67.2	79.1	31.6

Trial#	Strain	Sex	Inf?	%StanceStride_LH	%StanceStride_RF	%StanceStride_RH	%SwingStride_LF
1	CC013xCC041	M	Y	75.7	78.3	73.7	27.1
1	CC013xCC041	M	Y	77.8	69.4	72	27.5
2	CC013XCC041	F	Y	72.2	68.7	74.4	30.6
2	CC013XCC041	F	Y	75	76.6	82.4	28.8
2	CC013XCC041	F	Y	74.2	70.4	76.5	33.9
2	CC013XCC041	M	Y	80.3	75.7	77.3	26.8
2	CC013XCC041	M	Y	77.8	73.4	65.1	28
0	CC015	F	Y	68	69.9	72.7	32.6
0	CC015	M	Y	76.8	70.2	74.6	32.9
0	CC015	M	Y	75.6	69.2	74.6	31
1	CC015	F	Y	68.1	68.6	61.2	31.5
1	CC015	M	Y	71.6	75.5	61.6	27.1
1	CC015	M	Y	67.4	69.4	72.3	24.2
1	CC015	M	Y	76.6	70	77.3	31.4
2	CC015	F	Y	73.2	76.4	71.8	28.1
0	CC017	F	Y	62.7	60	63.1	42.3
0	CC017	M	Y	67.4	59	76.9	29.9
0	CC017	F	Y	78.2	70.1	78.9	33.4
0	CC017	M	Y	73.7	58	66.4	35.7
1	CC017	M	Y	75.1	57.2	75.1	42
1	CC017	F	Y	61.2	77.8	71.5	35.2
0	CC023	M	Y	71.1	61.1	63.6	41.8
0	CC023	F	Y	71.6	64.3	59.9	37.9
0	CC023	F	Y	61.7	60.5	57.2	39.8
0	CC023	M	Y	83.5	64.5	61.4	66.2
1	CC023	M	Y	82.8	63.3	85.5	29.4
1	CC023	F	Y	72.2	55.6	86.4	39.5
1	CC023	M	Y	82.2	68.9	68.1	25.9
1	CC023	M	Y	69.8	66.9	69.2	33.2
2	CC023	M	Y	52.9	75.9	66.2	18
2	CC023	F	Y	75.6	65.2	77.2	28.6
2	CC023	M	Y	65.7	68.5	75.7	38.2
0	CC025	M	Y	77.3	70.1	68	32.9
0	CC025	F	Y	81.1	68.7	71.6	39.4
0	CC025	M	Y	76	68	60.3	30.6
1	CC025	M	Y	78.2	72.3	68.1	32.6
1	CC025	F	Y	68.6	51.8	65.4	48.2
1	CC025	F	Y	72.4	65.8	68.8	36.2
1	CC025	F	Y	68.6	63.5	63.4	33.9
2	CC025	F	Y	71.5	70.5	64.1	29.2
2	CC025	M	Y	76.2	70.2	71	33.8
0	CC027	F	Y	73.4	60.6	60.8	33.9
0	CC027	M	Y	70.7	58.3	65.6	37.6
1	CC027	M	Y	70.3	74.5	75.5	9.9
1	CC027	M	Y	70.3	74.5	75.5	9.9
1	CC027	F	Y	57.8	55.3	65.4	46.8
1	CC027	F	Y	70.3	57.6	48.7	39.6
1	CC027	M	Y	81	77.4	82.1	27.3

Trial#	Strain	Sex	Inf?	%StanceStride_LH	%StanceStride_RF	%StanceStride_RH	%SwingStride_LF
1	CC027	M	Y	75.7	69.6	73.4	28.7
2	CC027	F	Y	75.6	65.2	77.2	28.6
2	CC027	M	Y	70	67.3	66.6	35.6
2	CC027	F	Y	69.9	65.4	60.8	30.4
2	CC027	M	Y	75.1	67.2	76.5	35.8
1	CC032XCC013	M	Y	72.6	69.3	73.9	32
1	CC032XCC013	M	Y	81.1	71	68.4	27.5
1	CC032XCC013	M	Y	61.9	57.8	53.6	45.3
1	CC032XCC013	F	Y	66.7	77	71.6	35.8
1	CC032XCC013	F	Y	61	60.6	64.6	28.2
1	CC032XCC013	F	Y	71.5	69.8	65.7	30.5
1	CC032XCC013	F	Y	71.8	68	65.2	25.8
1	CC032XCC013	F	Y	76	56.6	73.6	30.7
2	CC032XCC013	M	Y	76.8	72.2	76.9	25.3
2	CC032XCC013	M	Y	68.5	56.8	75.9	33.6
2	CC032XCC013	M	Y	73.8	77.9	66.7	50.4
2	CC032XCC013	M	Y	64.8	55.4	74.5	32.4
2	CC032XCC013	F	Y	78.6	65.3	71.4	29.4
2	CC032XCC013	F	Y	74.4	71.1	73	32.2
2	CC032XCC013	F	Y	72.7	60.4	68.1	25.2
2	CC032XCC013	F	Y	78.4	66	68.5	31.4
0	CC037	F	Y	74.9	65.5	76.6	35.1
0	CC037	M	Y	64.5	61.2	79.4	43.6
0	CC037	M	Y	67.6	62.1	46.9	35.4
0	CC037	M	Y	38.4	59.8	36.3	42.8
1	CC037	F	Y	75.3	68.3	73.8	31.8
1	CC037	M	Y	62.3	61.1	66.2	35.3
1	CC037	M	Y	70.3	67.7	67.3	30.4
1	CC037	M	Y	72.2	64	70.2	33.5
2	CC037	M	Y	76.1	68.1	71.9	30.1
2	CC037	M	Y	69.9	65.5	55.8	33.4
1	CC041XCC012	M	Y	80.5	61.2	77.2	23.5
1	CC041XCC012	M	Y	77.4	74.9	76.6	41.5
1	CC041XCC012	M	Y	65.1	73.5	72.8	25.1
1	CC041XCC012	M	Y	72.7	73.9	68.6	25.5
1	CC041XCC012	M	Y	74.4	67.4	76.2	29.6
1	CC041XCC012	F	Y	76.7	68.5	73.2	27.5
1	CC041XCC012	F	Y	73.5	66.7	74	32.8
1	CC041XCC012	F	Y	72.2	71.7	78.7	28.2
1	CC041XCC012	F	Y	78.1	74.3	72.3	27.6
1	CC041XCC012	F	Y	72.5	65.2	70.2	25.5
1	CC041XCC012	F	Y	72.2	65.6	67.1	32.9
1	CC041XCC012	F	Y	73.4	72.1	73.3	39.7
1	CC041XCC012	F	Y	78.8	65.7	78	21.8
2	CC041XCC012	M	Y	74.9	71.8	72	28
2	CC041XCC012	M	Y	72.2	65.1	75.4	35.5
2	CC041XCC012	F	Y	72	63.6	62	36.9
2	CC041XCC012	M	Y	54.5	70.5	78.8	23.8

Trial#	Strain	Sex	Inf?	%StanceStride_LH	%StanceStride_RF	%StanceStride_RH	%SwingStride_LF
2	CC041XCC012	M	Y	69.9	73.1	76.9	32.4
2	CC041XCC012	M	Y	48.9	69.5	72.1	29.2
2	CC041XCC012	F	Y	72.2	69.6	76.8	30.9
2	CC041XCC012	F	Y	79.8	72.9	75.2	27.1
2	CC041XCC012	F	Y	74.8	71.5	71.7	29.7
2	CC041XCC012	F	Y	68.1	67.7	69.1	32.2
2	CC041XCC012	F	Y	76.3	71.4	78.4	29.8
2	CC041XCC012	F	Y	77.9	75.5	80.1	28.4
2	CC041XCC012	F	Y	74.5	68.8	73.7	34.5
0	CC051	F	Y	75.8	67.4	74.7	33.6
0	CC051	F	Y	77.7	56.1	78.1	31
0	CC051	F	Y	74.7	69.5	73.4	35.5
0	CC051	M	Y	75.2	71.6	75.1	33.6
1	CC051	F	Y	73.6	66	76.8	33.6
1	CC051	F	Y	74.8	66.9	75	27.9
1	CC051	F	Y	77.3	68.3	77.3	33.7
0	CC057	F	Y	74	72.2	74.6	29.1
0	CC057	F	Y	70.1	71.7	68.7	34.2
0	CC057	F	Y	77.5	78.2	72.7	25.8
0	CC057	M	Y	78.6	67.3	78.8	25.7
0	CC057	M	Y	60.5	78.1	63.9	22.7
0	CC057	M	Y	73.3	68.1	75.9	24.5
1	CC057	F	Y	67.9	74.7	75.7	22.1
1	CC057	F	Y	78.1	78	67	30.2
1	CC057	F	Y	81.1	81.1	61.3	27.2
1	CC057	F	Y	71	67.8	70.4	24.8
1	CC057	M	Y	67.8	65.5	74.2	28.6
1	CC057	M	Y	73.3	79.1	73.9	23.1
2	CC057	F	Y	71	67.8	70.4	24.8
0	CC078	F	Y	65.2	63.9	68.4	40.5
0	CC078	F	Y	69.1	64.1	67.8	34.2
0	CC078	F	Y	69.9	63.6	67.7	35.7
0	CC078	M	Y	61.2	67.2	69.3	31.9
1	CC078	F	Y	74	76.5	68.6	33.9
1	CC078	F	Y	74.3	67.3	57	27.2
1	CC078	M	Y	60.8	68.5	68.3	37.2
2	CC078	F	Y	74.9	71	68	32.4
2	CC078	M	Y	66	71.2	71.8	38.1
2	CC005	M	N	80.4	85	71.6	37.2
2	CC005	M	Y	66	57.5	63.9	50.5
2	CC015	F	N	64.7	60.2	70.7	30.5
2	CC015	M	N	72.8	69.2	71.6	32.4
2	CC015	M	Y	78.2	66.5	75.3	25.3
2	CC015	M	Y	76.9	69.6	75.7	24.2
2	CC017	F	N	71.2	62.8	72.3	30.7
2	CC017	M	N	66.7	51	63.5	45.9
2	CC017	F	Y	64.5	72	67.3	24.9
2	CC023	F	N	69.2	67.3	59.8	32.3

Trial#	Strain	Sex	Inf?	%StanceStride_LH	%StanceStride_RF	%StanceStride_RH	%SwingStride_LF

Table S3. Raw DigiGait measurement data. Far left column indicates time point at which data was measured: T0 = pre-infection, T1 = 21dpi, and T3 = 89dpi. DigiGait parameters listed across the top indicate which limb is associated with the data, where appropriate: FL for left fore limb, FR for right fore limb, HL for left hind limb, and HR for right hind limb.

Table S3

Trial#	Strain	Sex	Inf?	%SwingStride_LH	%SwingStride_RF	%SwingStride_RH	AbsolutePawAngle_LF
1	CC002	F	N	27.9	35.4	26.2	9
1	CC025	F	N	22.2	30.4	25.6	8.2
1	CC012XCC032	F	N	27.7	24.7	30.1	9.7
2	CC012XCC032	F	N	33.6	34.1	26.5	6.2
1	CC012XCC032	M	N	15.8	25.3	17.1	8.4
2	CC012XCC032	M	N	17.5	33.7	15.2	7.9
1	CC013xCC041	F	N	34.1	30.8	25.3	6.5
2	CC013XCC041	F	N	25.9	24.7	22	9.4
1	CC013xCC041	M	N	34.6	33	35.6	1.2
2	CC013XCC041	M	N	23.7	30	23.8	5.9
1	CC032XCC013	F	N	32.1	33.8	24.1	5.8
2	CC032XCC013	F	N	21.6	33.9	20.7	0.7
1	CC041XCC012	F	N	33.1	25.7	31.6	11.4
1	CC041XCC012	F	N	32.2	34	31.4	10.6
2	CC041XCC012	F	N	23.8	47.9	28.1	4.5
1	CC032XCC013	M	N	34.2	27.7	37	4.1
2	CC032XCC013	M	N	22	41.2	40.9	33.1
1	CC041XCC012	M	N	28.6	34.8	29.3	18.6
2	CC041XCC012	M	N	25.2	29	33.4	15.3
0	CC012	F	N	31.3	30.4	28.2	21.6
2	CC012	F	N	28.5	32.5	34.9	2.3
0	CC012	M	N	29.5	41.7	26.6	10.8
2	CC012	M	N	22.8	29.5	17.4	19.3
0	CC057	F	N	26	30	27.9	5.8
1	CC057	F	N	26.8	27.7	26.3	4.4
0	CC057	M	N	26.4	24.3	23.7	8.3
1	CC057	M	N	28.6	24.7	26.1	3.7
0	CC078	F	N	27.4	34.2	27.6	3.8
1	CC078	F	N	31.6	35.6	29.8	6.2
2	CC078	F	N	19.1	40.4	36.5	1.7
0	CC078	M	N	38.2	38	35	8.7
2	CC078	M	N	30.2	37.2	32	3.2
0	CC002	F	N	32.7	32.7	36.4	3.6
1	CC002	F	N	27.9	35.4	26.2	9
0	CC002	M	N	27.8	30.6	39	12.2
1	CC002	M	N	24.5	13.2	29.1	13.6
2	CC002	M	N	23.4	34.1	23.7	7.1
0	CC006	F	N	29.5	36.8	33.1	1.5
1	CC006	F	N	27.5	28.6	30.4	1.3
2	CC006	F	N	27.2	32	36.4	6.5
0	CC006	M	N	23.3	30.4	46.4	5.6
0	CC023	F	N	29.5	33.8	26.1	0.6
1	CC023	F	N	32.7	48.1	35.1	7.9
2	CC023	F	N	27.5	36.2	34.9	1.1
0	CC023	M	N	25.9	35.7	33.7	7
1	CC023	M	N	26.6	32.3	28.6	1.3
0	CC027	F	N	70.4	47.4	55.6	4.9

Trial#	Strain	Sex	Inf?	%SwingStride_LH	%SwingStride_RF	%SwingStride_RH	AbsolutePawAngle_LF
1	CC027	F	N	28	26.6	31.3	2.5
2	CC027	F	N	28	28.8	30.2	1.5
0	CC027	M	N	56.5	34.9	58.5	2.5
1	CC027	M	N	26.4	54.1	26.4	8.4
1	CC027	M	N	13.8	27.6	25.2	1.3
2	CC027	M	N	35.5	39	37.6	3.3
0	CC005	F	N	26.5	39	26.1	25
1	CC005	F	N	24.8	31.5	23.6	20.6
2	CC005	F	N	42	21.1	38.4	0.7
0	CC011	F	N	29.3	38.7	22.8	3.8
1	CC011	F	N	20	43.4	21	0
2	CC011	F	N	26.4	34.6	24.4	3.8
1	CC017	F	N	24.8	25.1	20.6	1.9
0	CC005	M	N	26.4	34.1	26.4	11.8
1	CC005	M	N	34.6	36	24.5	1.4
2	CC005	M	N	22.8	37.1	15.2	4.5
0	CC011	M	N	25.9	32.1	28.9	8.5
1	CC011	M	N	27.3	28.1	27.6	11.1
2	CC011	M	N	21.7	31.7	20.2	15.5
0	CC017	M	N	54.9	37.9	58.4	2.3
1	CC017	M	N	25.9	23.2	30.8	4.6
1	CC006	F	N	63	48.4	46.3	10.5
0	CC037	F	N	27.4	38.2	40	4.3
0	CC051	F	N	26.9	38.1	23	8.4
1	CC051	F	N	25.1	29.8	23	5.9
1	CC006	M	N	34.7	39.2	32.4	8.6
0	CC037	M	N	29.7	37.8	29.2	14.3
1	CC037	M	N	34.5	29.8	22	9.5
0	CC005	F	N	23.1	29.8	28	18.2
1	CC005	F	N	23.2	24.8	25.8	26.3
2	CC005	F	N	35	54.5	50.5	0.2
0	CC011	F	N	24.8	29.2	25.7	9.2
1	CC011	F	N	24	40	24.8	1.8
2	CC011	F	N	23.3	25.5	20.9	18.7
0	CC011	M	N	29.4	35	27.2	7.7
1	CC011	M	N	29.3	39.6	29.7	9.1
2	CC011	M	N	25.8	36.5	31.1	3.6
1	CC037	M	N	23.7	34.7	22.4	5.7
2	CC037	M	N	21.5	52.9	17.7	2.9
1	CC051	M	N	26.6	32.7	23.1	16.2
2	CC051	M	N	35.4	28.1	23	23.1
0	CC027	F	N	22.6	32.5	31.1	2.3
1	CC027	F	N	29.2	39.1	40.5	4.7
2	CC027	F	N	23.2	28.3	43.1	3.5
0	CC015	M	N	33.5	31.6	31.4	0.9
1	CC015	M	N	49.2	30.8	57.4	8.4
2	CC015	M	N	31.6	31.6	40.4	7.3
0	CC027	M	N	27	28.7	31.5	12.3

Trial#	Strain	Sex	Inf?	%SwingStride_LH	%SwingStride_RF	%SwingStride_RH	AbsolutePawAngle_LF
1	CC027	M	N	28.1	35.6	29.9	11.5
2	CC027	M	N	38.1	35.8	28	17.7
0	CC015	F	N	35.6	21.7	0.6	5.5
1	CC015	F	N	32.6	28.5	36.2	6.8
0	CC017	F	N	35.9	33.8	38.6	1.6
0	CC023	F	N	28	30.7	25.9	2.7
1	CC023	F	N	27.9	32.4	33.6	5.9
0	CC005	M	N	29.7	36.3	48.9	10.3
1	CC005	M	N	22.7	23.2	54.1	20
0	CC015	M	N	25.2	32.3	25.3	10
1	CC015	M	N	26.6	27	36.9	2.4
0	CC017	M	N	19	29.9	23.1	9.5
1	CC017	M	N	31.8	32.3	27.2	1.1
0	CC023	M	N	29	35.9	28.7	6.8
1	CC023	M	N	29.6	32.7	24.1	0.9
0	CC051	M	N	23.5	35.2	25.3	0.3
0	CC002	F	Y	19.4	33.5	28.1	1.8
0	CC002	M	Y	23.7	27.9	31.3	10.4
1	CC002	M	Y	48.7	34.1	84.6	0.7
1	CC002	F	Y	51.2	28.3	46	15.1
1	CC002	F	Y	27	30.4	30.6	0.7
1	CC002	M	Y	48.7	34.1	84.6	0.7
1	CC002	M	Y	26.9	26.3	29.5	1
1	CC002	F	Y	51.2	28.3	46	15.1
1	CC002	F	Y	52.8	31.2	50.9	4.2
2	CC002	M	Y	34.9	36.8	28	8.4
2	CC002	F	Y	25.8	29.5	25.6	13.6
2	CC002	M	Y	25.5	27.3	27.2	8.5
0	CC005	F	Y	24.1	41.7	26.7	12.9
0	CC005	M	Y	27.6	35.5	24.1	17
0	CC005	M	Y	24.3	32.6	23.1	1.7
1	CC005	F	Y	24.8	38.4	32.6	14.2
1	CC005	M	Y	36.3	24.1	74.1	1.7
1	CC005	F	Y	34.8	30.9	53.8	15
1	CC005	M	Y	30.5	36.1	26.5	13.6
2	CC005	F	Y	22.7	32.9	19.2	9.1
2	CC005	M	Y	22.7	26	28.1	0.8
2	CC005	M	Y	20.1	34.7	55.8	10.2
2	CC005	M	Y	22.7	26	28.1	0.8
2	CC005	F	Y	21.7	23.3	52.4	6.2
0	CC006	F	Y	29	36.2	26.6	2.4
0	CC006	M	Y	41.3	43.4	72.7	2.9
0	CC006	F	Y	26	39.2	22.6	4.7
1	CC006	F	Y	26.4	42.4	23.5	8.9
1	CC006	M	Y	29.9	31.3	24.1	1.2
1	CC006	F	Y	31.1	40	30	4.6
1	CC006	M	Y	31.4	31.4	26.2	1.3
2	CC006	F	Y	37	33.2	28.6	8.5

Trial#	Strain	Sex	Inf?	%SwingStride_LH	%SwingStride_RF	%SwingStride_RH	AbsolutePawAngle_LF
2	CC006	M	Y	32	24.6	37.8	1.5
0	CC011	F	Y	21	42.4	20.5	2.4
0	CC011	M	Y	23.8	30.6	26.6	5.9
0	CC011	F	Y	33.1	35.3	34.4	17.7
0	CC011	F	Y	76.7	42.7	59.7	16.8
1	CC011	F	Y	26.6	32	28.8	18.3
1	CC011	M	Y	24.5	21.2	26.5	11.6
1	CC011	F	Y	29.8	27.8	38.2	26.1
1	CC011	M	Y	28	28.6	27.5	23.9
2	CC011	F	Y	28	33.8	28.2	9.6
2	CC011	M	Y	20.4	24.6	19.3	1.6
2	CC011	F	Y	21.9	26	26.6	21.2
2	CC011	M	Y	29.1	35.7	32.9	12
0	CC012	M	Y	28.8	36.3	32.9	1.7
0	CC012	M	Y	37	35.6	33.6	6.1
0	CC012	M	Y	31.9	38.3	31.3	2.4
0	CC012	M	Y	33.6	39.3	28.4	2.2
0	CC012	M	Y	33.3	33.8	28.2	1.7
0	CC012	F	Y	27.5	45.2	23.5	2.1
0	CC012	F	Y	26.5	33.8	28.7	9.3
0	CC012	F	Y	36.1	33.3	28.7	11.2
0	CC012	F	Y	39.5	40.2	42.2	0
0	CC012	F	Y	45.6	37.2	42.6	12.2
2	CC012	M	Y	27.7	37.2	30.5	4.7
2	CC012	M	Y	36.1	38.2	34.1	0.3
2	CC012	M	Y	30.9	36.7	33.1	16.8
2	CC012	M	Y	27.4	32.6	22.6	4.1
2	CC012	M	Y	30.6	35.1	32.9	8.5
2	CC012	F	Y	23.9	39.2	24	1.9
2	CC012	F	Y	34	31.1	37	4.8
2	CC012	F	Y	29.6	34.8	23.3	0.5
2	CC012	F	Y	29.8	26.7	33.1	6.8
2	CC012	F	Y	28.6	27	26.3	4.7
1	CC012XCC032	F	Y	34.8	26.3	38.6	10.3
1	CC012xCC032	F	Y	39.6	33.9	46.7	1.1
1	CC012XCC032	M	Y	26.1	30.6	29.3	7.3
1	CC012xCC032	M	Y	32.2	35.8	30.6	1.3
1	CC012XCC032	M	Y	30.6	28.3	42.8	1.3
1	CC012xCC032	M	Y	27.3	18	27.2	15.4
2	CC012XCC032	F	Y	30.3	37.3	27.7	1.9
2	CC012XCC032	F	Y	30.9	25.6	29.1	2.2
2	CC012XCC032	M	Y	25.8	42.7	28.4	7.8
2	CC012XCC032	M	Y	33	34.2	37.4	16.4
2	CC012XCC032	M	Y	23.1	40	24.2	2.1
2	CC012XCC032	M	Y	37	31.3	21.9	5.5
1	CC013xCC041	F	Y	33.9	29.9	29.7	2.2
1	CC013xCC041	F	Y	33.8	31.8	38.9	1.4
1	CC013xCC041	F	Y	22.5	32.8	20.9	1.5

Trial#	Strain	Sex	Inf?	%SwingStride_LH	%SwingStride_RF	%SwingStride_RH	AbsolutePawAngle_LF
1	CC013xCC041	M	Y	24.3	21.7	26.3	4.7
1	CC013xCC041	M	Y	22.2	30.6	28	2
2	CC013XCC041	F	Y	27.8	31.3	25.6	3
2	CC013XCC041	F	Y	25	23.4	17.6	1.4
2	CC013XCC041	F	Y	25.8	29.6	23.5	1.3
2	CC013XCC041	M	Y	19.7	24.3	22.7	3.3
2	CC013XCC041	M	Y	22.2	26.6	34.9	1.7
0	CC015	F	Y	32	30.1	27.3	16.7
0	CC015	M	Y	23.2	29.8	25.4	2
0	CC015	M	Y	24.4	30.8	25.4	7.2
1	CC015	F	Y	31.9	31.4	38.8	4.2
1	CC015	M	Y	28.4	24.5	38.4	5.3
1	CC015	M	Y	32.6	30.6	27.7	2.5
1	CC015	M	Y	23.4	30	22.7	13.5
2	CC015	F	Y	26.8	23.6	28.2	1.5
0	CC017	F	Y	37.3	40	36.9	11.1
0	CC017	M	Y	32.6	41	23.1	7.9
0	CC017	F	Y	21.8	29.9	21.1	7.4
0	CC017	M	Y	26.3	42	33.6	4.8
1	CC017	M	Y	24.9	42.8	24.9	9.2
1	CC017	F	Y	38.8	22.2	28.5	18.4
0	CC023	M	Y	28.9	38.9	36.4	3.5
0	CC023	F	Y	28.4	35.7	40.1	3.6
0	CC023	F	Y	38.3	39.5	42.8	1.8
0	CC023	M	Y	16.5	35.5	38.6	14.6
1	CC023	M	Y	17.2	36.7	14.5	8.9
1	CC023	F	Y	27.8	44.4	13.6	14.8
1	CC023	M	Y	17.8	31.1	31.9	13.6
1	CC023	M	Y	30.2	33.1	30.8	16.8
2	CC023	M	Y	47.1	24.1	33.8	5.6
2	CC023	F	Y	24.4	34.8	22.8	7.1
2	CC023	M	Y	34.3	31.5	24.3	27.5
0	CC025	M	Y	22.7	29.9	32	5.1
0	CC025	F	Y	18.9	31.3	28.4	3.2
0	CC025	M	Y	24	32	39.7	4.4
1	CC025	M	Y	21.8	27.7	31.9	5.4
1	CC025	F	Y	31.4	48.2	34.6	0.3
1	CC025	F	Y	27.6	34.2	31.2	1.5
1	CC025	F	Y	31.4	36.5	36.6	3
2	CC025	F	Y	28.5	29.5	35.9	0.6
2	CC025	M	Y	23.8	29.8	29	13.9
0	CC027	F	Y	26.6	39.4	39.2	0.6
0	CC027	M	Y	29.3	41.7	34.4	11.4
1	CC027	M	Y	29.7	25.5	24.5	33.4
1	CC027	M	Y	29.7	25.5	24.5	33.4
1	CC027	F	Y	42.2	44.7	34.6	14.3
1	CC027	F	Y	29.7	42.4	51.3	19.9
1	CC027	M	Y	19	22.6	17.9	2.9

Trial#	Strain	Sex	Inf?	%SwingStride_LH	%SwingStride_RF	%SwingStride_RH	AbsolutePawAngle_LF
1	CC027	M	Y	24.3	30.4	26.6	1.4
2	CC027	F	Y	24.4	34.8	22.8	7.1
2	CC027	M	Y	30	32.7	33.4	10.1
2	CC027	F	Y	30.1	34.6	39.2	8.9
2	CC027	M	Y	24.9	32.8	23.5	3.5
1	CC032XCC013	M	Y	27.4	30.7	26.1	0.3
1	CC032XCC013	M	Y	18.9	29	31.6	6.8
1	CC032XCC013	M	Y	38.1	42.2	46.4	10
1	CC032XCC013	F	Y	33.3	23	28.4	21.2
1	CC032XCC013	F	Y	39	39.4	35.4	5.4
1	CC032XCC013	F	Y	28.5	30.2	34.3	11.8
1	CC032XCC013	F	Y	28.2	32	34.8	12.1
1	CC032XCC013	F	Y	24	43.4	26.4	18
2	CC032XCC013	M	Y	23.2	27.8	23.1	3.8
2	CC032XCC013	M	Y	31.5	43.2	24.1	6.1
2	CC032XCC013	M	Y	26.2	22.1	33.3	12
2	CC032XCC013	M	Y	35.2	44.6	25.5	13.2
2	CC032XCC013	F	Y	21.4	34.7	28.6	10.9
2	CC032XCC013	F	Y	25.6	28.9	27	14
2	CC032XCC013	F	Y	27.3	39.6	31.9	1.2
2	CC032XCC013	F	Y	21.6	34	31.5	1.5
0	CC037	F	Y	25.1	34.5	23.4	5.5
0	CC037	M	Y	35.5	38.8	20.6	1.6
0	CC037	M	Y	32.4	37.9	53.1	0.6
0	CC037	M	Y	61.6	40.2	63.7	16.4
1	CC037	F	Y	24.7	31.7	26.2	1.2
1	CC037	M	Y	37.7	38.9	33.8	12.5
1	CC037	M	Y	29.7	32.3	32.7	6.6
1	CC037	M	Y	27.8	36	29.8	3.4
2	CC037	M	Y	23.9	31.9	28.1	7.5
2	CC037	M	Y	30.1	34.5	44.2	4.4
1	CC041XCC012	M	Y	19.5	38.8	22.8	3.7
1	CC041XCC012	M	Y	22.6	25.1	23.4	4.3
1	CC041XCC012	M	Y	34.9	26.5	27.2	28.2
1	CC041XCC012	M	Y	27.3	26.1	31.4	12.3
1	CC041XCC012	M	Y	25.6	32.6	23.8	8.8
1	CC041XCC012	F	Y	23.3	31.5	26.8	2.5
1	CC041XCC012	F	Y	26.5	33.3	26	5.8
1	CC041XCC012	F	Y	27.8	28.3	21.3	9.5
1	CC041XCC012	F	Y	21.9	25.7	27.7	11.4
1	CC041XCC012	F	Y	27.5	34.8	29.8	2.2
1	CC041XCC012	F	Y	27.8	34.4	32.9	4.5
1	CC041XCC012	F	Y	26.6	27.9	26.7	2.5
1	CC041XCC012	F	Y	21.2	34.3	22	3.4
2	CC041XCC012	M	Y	25.1	28.2	28	4.6
2	CC041XCC012	M	Y	27.8	34.9	24.6	5.4
2	CC041XCC012	F	Y	28	36.4	38	2.9
2	CC041XCC012	M	Y	45.5	29.5	21.2	21.6

Trial#	Strain	Sex	Inf?	%SwingStride_LH	%SwingStride_RF	%SwingStride_RH	AbsolutePawAngle_LF
2	CC041XCC012	M	Y	30.1	26.9	23.1	11
2	CC041XCC012	M	Y	51.1	30.5	27.9	17.1
2	CC041XCC012	F	Y	27.8	30.4	23.2	5.9
2	CC041XCC012	F	Y	20.2	27.1	24.8	2
2	CC041XCC012	F	Y	25.2	28.5	28.3	8
2	CC041XCC012	F	Y	31.9	32.3	30.9	0.1
2	CC041XCC012	F	Y	23.7	28.6	21.6	11.7
2	CC041XCC012	F	Y	22.1	24.5	19.9	2
2	CC041XCC012	F	Y	25.5	31.2	26.3	3.5
0	CC051	F	Y	24.2	32.6	25.3	12.3
0	CC051	F	Y	22.3	43.9	21.9	12.9
0	CC051	F	Y	25.3	30.5	26.6	4.3
0	CC051	M	Y	24.8	28.4	24.9	11.7
1	CC051	F	Y	26.4	34	23.2	9.9
1	CC051	F	Y	25.2	33.1	25	2.9
1	CC051	F	Y	22.7	31.7	22.7	5.1
0	CC057	F	Y	26	27.8	25.4	11.7
0	CC057	F	Y	29.9	28.3	31.3	18.2
0	CC057	F	Y	22.5	21.8	27.3	7
0	CC057	M	Y	21.4	32.7	21.2	14.3
0	CC057	M	Y	39.5	21.9	36.1	2.1
0	CC057	M	Y	26.7	31.9	24.1	10.3
1	CC057	F	Y	32.1	25.3	24.3	14.5
1	CC057	F	Y	21.9	22	33	13.2
1	CC057	F	Y	18.9	18.9	38.7	9.9
1	CC057	F	Y	29	32.2	29.6	8.3
1	CC057	M	Y	32.2	34.5	25.8	13.9
1	CC057	M	Y	26.7	20.9	26.1	15.2
2	CC057	F	Y	29	32.2	29.6	8.3
0	CC078	F	Y	34.8	36.1	31.6	4.6
0	CC078	F	Y	30.9	35.9	32.2	0.1
0	CC078	F	Y	30.1	36.4	32.3	3.9
0	CC078	M	Y	38.8	32.8	30.7	1.1
1	CC078	F	Y	26	23.5	31.4	7
1	CC078	F	Y	25.7	32.7	43	19.5
1	CC078	M	Y	39.2	31.5	31.7	7.4
2	CC078	F	Y	25.1	29	32	2.9
2	CC078	M	Y	34	28.8	28.2	9.6
2	CC005	M	N	19.6	15	28.4	7.4
2	CC005	M	Y	34	42.5	36.1	1.7
2	CC015	F	N	35.3	39.8	29.3	13.3
2	CC015	M	N	27.2	30.8	28.4	2.2
2	CC015	M	Y	21.8	33.5	24.7	8.4
2	CC015	M	Y	23.1	30.4	24.3	0.2
2	CC017	F	N	28.8	37.2	27.7	3.8
2	CC017	M	N	33.3	49	36.5	0.5
2	CC017	F	Y	35.5	28	32.7	5.7
2	CC023	F	N	30.8	32.7	40.2	0.1

Trial#	Strain	Sex	Inf?	%SwingStride_LH	%SwingStride_RF	%SwingStride_RH	AbsolutePawAngle_LF

Table S3. Raw DigiGait measurement data. Far left column indicates time point at which data was measured: T0 = pre-infection, T1 = 21dpi, and T3 = 89dpi. DigiGait parameters listed across the top indicate which limb is associated with the data, where appropriate: FL for left fore limb, FR for right fore limb, HL for left hind limb, and HR for right hind limb.

Table S3

Trial#	Strain	Sex	Inf?	AbsolutePawAngle_LH	AbsolutePawAngle_RF	AbsolutePawAngle_RH	AtaxiaCoefficient_LF
1	CC002	F	N	10.2	12.8	17.5	2.58
1	CC025	F	N	15.9	1.7	11.4	1.05
1	CC012XCC032	F	N	26.5	4.5	28.4	1.18
2	CC012XCC032	F	N	17.3	8.9	10.9	1.14
1	CC012XCC032	M	N	16.3	7.9	18.9	1.2
2	CC012XCC032	M	N	10.8	3.8	16.8	0.4
1	CC013xCC041	F	N	10.5	1.3	5.9	1.84
2	CC013XCC041	F	N	8.6	11.3	16	0.31
1	CC013xCC041	M	N	12.3	26.7	5.9	1.26
2	CC013XCC041	M	N	12	0.7	4.1	0.97
1	CC032XCC013	F	N	4.6	6.6	18.2	1.19
2	CC032XCC013	F	N	12.3	7.1	7.8	1.24
1	CC041XCC012	F	N	6.8	5.1	14.5	1.89
1	CC041XCC012	F	N	0.8	3.6	12.9	2.36
2	CC041XCC012	F	N	8.9	25	20.7	1.57
1	CC032XCC013	M	N	8.6	10.1	11.3	2.04
2	CC032XCC013	M	N	11.6	22.5	22.8	1.3
1	CC041XCC012	M	N	34.1	20.5	26	1.07
2	CC041XCC012	M	N	37.3	14.1	21	0.93
0	CC012	F	N	16.7	5.9	24.1	1.31
2	CC012	F	N	11.4	25.9	19.9	0.98
0	CC012	M	N	18.3	17.9	27.6	1.5
2	CC012	M	N	10.9	1.3	22.3	2.32
0	CC057	F	N	16	12	19.1	0.71
1	CC057	F	N	13.2	3.3	20.8	0.83
0	CC057	M	N	19.1	8.6	21.2	1.66
1	CC057	M	N	12.1	11.6	22.8	0.39
0	CC078	F	N	24.5	3.7	12.9	0.31
1	CC078	F	N	17.3	9.8	13.8	0.56
2	CC078	F	N	12.8	1.3	18.3	2.72
0	CC078	M	N	16.7	17.2	10.9	0.66
2	CC078	M	N	6.4	10.1	7.3	1.41
0	CC002	F	N	11	4.6	10.3	1.58
1	CC002	F	N	10.2	12.8	17.5	2.58
0	CC002	M	N	8.5	1.5	5.8	1.95
1	CC002	M	N	9.5	10.5	5.6	1.8
2	CC002	M	N	3.5	4.5	8.2	0.99
0	CC006	F	N	3.3	4.4	16.8	0.52
1	CC006	F	N	15.8	4.7	15.4	1.69
2	CC006	F	N	20.2	0.6	13	1.55
0	CC006	M	N	1	5.9	10.1	1.77
0	CC023	F	N	20.6	2.3	15.5	1.21
1	CC023	F	N	14	1.1	6.5	2.4
2	CC023	F	N	8.3	4.9	4.9	1.35
0	CC023	M	N	16.1	7.5	8.9	0.46
1	CC023	M	N	16.8	2.8	13.3	1.01
0	CC027	F	N	2.7	10	9.3	2

Trial#	Strain	Sex	Inf?	AbsolutePawAngle_LH	AbsolutePawAngle_RF	AbsolutePawAngle_RH	AtaxiaCoefficient_LF
1	CC027	F	N	8.5	5.9	17.6	1.67
2	CC027	F	N	20.1	5.6	13.2	1.05
0	CC027	M	N	2.5	5.6	5.2	1.18
1	CC027	M	N	0.2	2.1	4.1	0.92
1	CC027	M	N	1.6	1.2	1.6	1.29
2	CC027	M	N	18.5	3.7	11.1	0.45
0	CC005	F	N	22.4	13.3	13.4	1.49
1	CC005	F	N	9.6	31.4	4.4	1.31
2	CC005	F	N	3.4	16.7	14.6	2.04
0	CC011	F	N	26.5	0.5	9	1.13
1	CC011	F	N	1.1	4.7	12.4	1.71
2	CC011	F	N	3	5.3	8	1.65
1	CC017	F	N	23.8	12.6	22.5	2.16
0	CC005	M	N	21.2	18	14.5	0.28
1	CC005	M	N	4.2	17.9	2.8	2.13
2	CC005	M	N	14.2	17.5	20.8	3.94
0	CC011	M	N	18.9	10.7	16.8	0.25
1	CC011	M	N	8.8	0.9	12.5	0.51
2	CC011	M	N	14.8	19.9	19.8	3.01
0	CC017	M	N	6	12.2	6.4	2.68
1	CC017	M	N	17.5	10.9	17.3	0.77
1	CC006	F	N	4.7	7.8	2.6	1.9
0	CC037	F	N	26.5	0.8	29.3	1.38
0	CC051	F	N	16.3	2.3	15.1	1.64
1	CC051	F	N	12.5	8.5	15.4	1.54
1	CC006	M	N	7.7	0.8	8.6	1.56
0	CC037	M	N	29.3	13.9	23.3	2.34
1	CC037	M	N	26.4	1.8	7.7	0.77
0	CC005	F	N	14.1	1.7	9.8	1.73
1	CC005	F	N	17	21.4	13.2	0.43
2	CC005	F	N	24	22	13.4	0.28
0	CC011	F	N	18.7	4.5	17.9	0.44
1	CC011	F	N	15.5	4.5	9.8	0.65
2	CC011	F	N	12.8	18.3	8.8	0.74
0	CC011	M	N	14.3	3.8	18.2	0.98
1	CC011	M	N	17.7	9.6	20.6	0.81
2	CC011	M	N	8.5	12.2	24.2	0.57
1	CC037	M	N	17.2	10.5	12.8	0.75
2	CC037	M	N	20.8	7.8	12.7	1.22
1	CC051	M	N	20.9	11.1	22.1	0.45
2	CC051	M	N	15.4	8.5	10.9	1.32
0	CC027	F	N	25.9	3	19.3	0.34
1	CC027	F	N	23	4.8	16.9	0.62
2	CC027	F	N	10.4	2.8	20.5	3.09
0	CC015	M	N	8.9	9.9	23	0.81
1	CC015	M	N	25.3	3.8	2.4	1.19
2	CC015	M	N	9	20	18.1	0.41
0	CC027	M	N	21.3	2.8	14.5	1.88

Trial#	Strain	Sex	Inf?	AbsolutePawAngle_LH	AbsolutePawAngle_RF	AbsolutePawAngle_RH	AtaxiaCoefficient_LF
1	CC027	M	N	16.6	15	13.1	1.26
2	CC027	M	N	20	13	4.9	1.35
0	CC015	F	N	5.8	14.3	0.87	0.44
1	CC015	F	N	11.5	1.7	7	3.88
0	CC017	F	N	20.3	5.2	12.7	1.77
0	CC023	F	N	9.6	0.2	9.3	0.74
1	CC023	F	N	23.1	1.8	20.4	0.72
0	CC005	M	N	27.8	5	17.4	0.58
1	CC005	M	N	11.8	8.3	11.3	0.78
0	CC015	M	N	4.1	10.8	11.2	0.82
1	CC015	M	N	12.2	1.9	5.3	1.29
0	CC017	M	N	2.1	8.5	13.1	1.2
1	CC017	M	N	6.4	5.7	15.6	1.49
0	CC023	M	N	20.7	4.3	14.3	0.44
1	CC023	M	N	20.3	5.9	17.7	1.8
0	CC051	M	N	3.6	5	19.4	0.53
0	CC002	F	Y	21.2	1.6	1.9	2.13
0	CC002	M	Y	8.9	1.1	0.1	1.57
1	CC002	M	Y	6.4	21.3	26.2	1.2
1	CC002	F	Y	49.5	21.3	24.5	1.74
1	CC002	F	Y	7.3	4.6	16.2	2.3
1	CC002	M	Y	6.4	21.3	26.2	1.2
1	CC002	M	Y	5.8	5	29.1	2.57
1	CC002	F	Y	49.5	21.3	24.5	1.74
1	CC002	F	Y	4.5	0.4	2.4	1.28
2	CC002	M	Y	15	12	8.3	1.56
2	CC002	F	Y	5.9	10.4	7.5	0.83
2	CC002	M	Y	11.2	8.9	13.1	1.81
0	CC005	F	Y	19	6.9	6.3	1.02
0	CC005	M	Y	17.5	3.5	13.4	1.04
0	CC005	M	Y	5.8	3.5	18.4	0.67
1	CC005	F	Y	17	17.5	4.1	1
1	CC005	M	Y	34.6	22.6	34.6	1.43
1	CC005	F	Y	7.9	27.4	10.3	0.46
1	CC005	M	Y	24	2	8.7	1.47
2	CC005	F	Y	10.8	28	12.9	2.95
2	CC005	M	Y	4.6	4.6	17.5	1.68
2	CC005	M	Y	13.7	15	6.9	1.97
2	CC005	M	Y	4.6	4.6	17.5	1.68
2	CC005	F	Y	15.7	20.4	3	1.31
0	CC006	F	Y	15.2	1.5	17	1.26
0	CC006	M	Y	33.9	6.5	2.2	1.41
0	CC006	F	Y	18.1	3.7	18.9	2.83
1	CC006	F	Y	4.8	3.9	3.8	2.02
1	CC006	M	Y	2.9	4.2	1.3	3.15
1	CC006	F	Y	1.9	7	11.6	0.52
1	CC006	M	Y	21.6	1.9	9.8	1.29
2	CC006	F	Y	8.9	0.4	17.2	1.54

Trial#	Strain	Sex	Inf?	AbsolutePawAngle_LH	AbsolutePawAngle_RF	AbsolutePawAngle_RH	AtaxiaCoefficient_LF
2	CC006	M	Y	9.7	2.5	12	3.07
0	CC011	F	Y	0.4	11.1	3.9	1.82
0	CC011	M	Y	19.6	7.5	18.2	1.67
0	CC011	F	Y	23.7	12	27.2	0.6
0	CC011	F	Y	18.4	1.3	26.4	1.04
1	CC011	F	Y	24.3	14.7	24.6	1.31
1	CC011	M	Y	19.5	15.3	17.6	0.51
1	CC011	F	Y	13.7	2.9	20.3	0.52
1	CC011	M	Y	19.2	19.9	16.1	0.53
2	CC011	F	Y	20.3	16.1	20.1	0.47
2	CC011	M	Y	22.6	14.9	12.9	2.2
2	CC011	F	Y	16.4	20.1	18.7	0.28
2	CC011	M	Y	15.7	16.5	13.8	0.66
0	CC012	M	Y	11.1	3.4	23.3	0.3
0	CC012	M	Y	23.2	10.6	18.3	0.51
0	CC012	M	Y	21.5	7.1	27.1	0.27
0	CC012	M	Y	33.4	0.2	9	2.01
0	CC012	M	Y	21.2	8.1	10.3	0.61
0	CC012	F	Y	7.6	3.4	12.7	1.18
0	CC012	F	Y	19.8	9.4	24.5	0.76
0	CC012	F	Y	22.9	1.8	24.6	2.17
0	CC012	F	Y	11.7	0.5	25.3	1.89
0	CC012	F	Y	20.8	4.1	6	0.57
2	CC012	M	Y	16.5	1.2	13.3	0.35
2	CC012	M	Y	9.9	15.9	24.5	0.45
2	CC012	M	Y	6.9	11.8	16.2	0.28
2	CC012	M	Y	9.1	5	12.9	0.33
2	CC012	M	Y	11.2	3.3	13.6	0.5
2	CC012	F	Y	8.7	5.4	23.1	0.82
2	CC012	F	Y	19	1.8	31.8	0.89
2	CC012	F	Y	20.5	2.4	14.5	2.29
2	CC012	F	Y	21.7	17.8	26.8	1.33
2	CC012	F	Y	25	6.5	21.8	0.99
1	CC012XCC032	F	Y	1.3	9.8	15.3	3
1	CC012xCC032	F	Y	2.3	10.1	5.2	2.47
1	CC012XCC032	M	Y	15.4	1.7	17.4	1.89
1	CC012xCC032	M	Y	6.6	3.1	21.1	2.16
1	CC012XCC032	M	Y	17.8	0.4	8.6	1.63
1	CC012xCC032	M	Y	9.6	2.7	16.2	3.37
2	CC012XCC032	F	Y	12.2	12.1	23.7	1.29
2	CC012XCC032	F	Y	7.4	3.7	9.3	0.25
2	CC012XCC032	M	Y	15.9	6.5	12.2	1.64
2	CC012XCC032	M	Y	16.8	6.4	36.1	1.99
2	CC012XCC032	M	Y	12.4	14.1	13	0.46
2	CC012XCC032	M	Y	14.2	9.4	12.6	1.35
1	CC013xCC041	F	Y	12.5	0.1	6.3	2.17
1	CC013xCC041	F	Y	5.3	0.1	5.2	1.99
1	CC013xCC041	F	Y	7.8	3.2	13.5	2.69

Trial#	Strain	Sex	Inf?	AbsolutePawAngle_LH	AbsolutePawAngle_RF	AbsolutePawAngle_RH	AtaxiaCoefficient_LF
1	CC013xCC041	M	Y	15.6	8.9	4.9	1.22
1	CC013xCC041	M	Y	7.7	5	2.5	1.6
2	CC013XCC041	F	Y	2.4	6.1	1.7	0.48
2	CC013XCC041	F	Y	5	3.6	0	0.97
2	CC013XCC041	F	Y	0.1	0.9	2.2	0.52
2	CC013XCC041	M	Y	5.6	2	2.6	1.61
2	CC013XCC041	M	Y	9	1.9	6.4	2.69
0	CC015	F	Y	4.9	1.7	15.5	0.84
0	CC015	M	Y	7.8	6	6.2	0.49
0	CC015	M	Y	9	0.1	8.5	0.45
1	CC015	F	Y	4.5	8.3	13.7	0.53
1	CC015	M	Y	7.8	0.5	8.4	2.38
1	CC015	M	Y	5.4	2.5	11.6	1.89
1	CC015	M	Y	24.8	12.8	18.5	2.01
2	CC015	F	Y	2	2.5	11.4	1.03
0	CC017	F	Y	11.4	0.3	15.1	0.47
0	CC017	M	Y	5.2	18.1	7.3	0.52
0	CC017	F	Y	3.8	11.9	1.3	2.23
0	CC017	M	Y	6.5	1.9	6	1.08
1	CC017	M	Y	15.7	1.1	15.1	1.29
1	CC017	F	Y	8.9	13	14.5	1.4
0	CC023	M	Y	17	6.8	13.7	0.23
0	CC023	F	Y	18.9	2.7	4.6	1.57
0	CC023	F	Y	17.6	0	19.1	1.43
0	CC023	M	Y	28.3	1.2	5.8	1.3
1	CC023	M	Y	3	4.8	4.7	2.44
1	CC023	F	Y	4	0.1	7	3.75
1	CC023	M	Y	18.7	1.1	15.1	1.64
1	CC023	M	Y	16.7	9.7	17.5	0.62
2	CC023	M	Y	7.4	13.1	7	0.39
2	CC023	F	Y	3.8	16.4	15	1.42
2	CC023	M	Y	16.2	6	1.3	2.07
0	CC025	M	Y	16.7	7.1	21.4	1.32
0	CC025	F	Y	12.3	17.9	8.7	1.31
0	CC025	M	Y	29.4	9.1	19.5	0.48
1	CC025	M	Y	18.8	9.1	20.9	1.39
1	CC025	F	Y	13.7	9.8	21.8	1.44
1	CC025	F	Y	19.1	2.2	17.6	2.84
1	CC025	F	Y	1.4	2.1	10	1.27
2	CC025	F	Y	6.3	5.2	10.2	0.9
2	CC025	M	Y	12.3	11.7	22.9	0.64
0	CC027	F	Y	20.8	2.1	9.8	0.4
0	CC027	M	Y	30.7	8.3	23.9	2.03
1	CC027	M	Y	11.1	14.8	19.5	1.69
1	CC027	M	Y	11.1	14.8	19.5	1.69
1	CC027	F	Y	13.7	11.3	10.3	1.66
1	CC027	F	Y	30.6	7.1	11.5	1.32
1	CC027	M	Y	22.5	12.9	10.6	0.23

Trial#	Strain	Sex	Inf?	AbsolutePawAngle_LH	AbsolutePawAngle_RF	AbsolutePawAngle_RH	AtaxiaCoefficient_LF
1	CC027	M	Y	20.7	13.6	23.6	0.25
2	CC027	F	Y	3.8	16.4	15	1.42
2	CC027	M	Y	18.8	2.4	23.8	1.42
2	CC027	F	Y	24.8	3.6	15	1.74
2	CC027	M	Y	14.8	9.7	12.2	1.49
1	CC032XCC013	M	Y	11.8	14.5	23.6	2.11
1	CC032XCC013	M	Y	8.5	0.4	24.8	1.38
1	CC032XCC013	M	Y	8.5	2.5	21.7	1.8
1	CC032XCC013	F	Y	15.7	4.3	14.9	1.46
1	CC032XCC013	F	Y	7.1	12.5	15.9	2.06
1	CC032XCC013	F	Y	9.5	8	20.2	1.34
1	CC032XCC013	F	Y	10	7.5	20	0.91
1	CC032XCC013	F	Y	8.8	14.7	30.3	0.44
2	CC032XCC013	M	Y	18.6	3.2	18.2	0.53
2	CC032XCC013	M	Y	8.8	17.5	19.2	1.59
2	CC032XCC013	M	Y	15.9	9.2	22.3	3.23
2	CC032XCC013	M	Y	14.8	8.4	7.6	0.84
2	CC032XCC013	F	Y	7	11.5	15	0.53
2	CC032XCC013	F	Y	11	6	11.1	1.22
2	CC032XCC013	F	Y	11.8	1.3	12.5	0.53
2	CC032XCC013	F	Y	12.4	3.4	9.6	0.9
0	CC037	F	Y	18.1	0.6	35.3	1.7
0	CC037	M	Y	23.3	9.1	19.2	2.12
0	CC037	M	Y	9.8	9	21.2	2.02
0	CC037	M	Y	6	12.8	21.3	0.76
1	CC037	F	Y	14.6	1.3	20.5	1.84
1	CC037	M	Y	17	4.1	22.8	1.07
1	CC037	M	Y	19.7	14.1	9.6	1.21
1	CC037	M	Y	22.6	10.7	15.6	0.48
2	CC037	M	Y	15.4	22.7	10.9	1.32
2	CC037	M	Y	22.9	11	27.2	0.32
1	CC041XCC012	M	Y	6.5	7.3	3.7	1.41
1	CC041XCC012	M	Y	8.4	12.4	3.2	2.23
1	CC041XCC012	M	Y	11.9	1.8	0.9	0.85
1	CC041XCC012	M	Y	6.9	4.2	0.6	2.01
1	CC041XCC012	M	Y	21.2	0.9	6	0.88
1	CC041XCC012	F	Y	13.5	8.7	28.5	1.14
1	CC041XCC012	F	Y	10.4	8.3	10.4	0.71
1	CC041XCC012	F	Y	17.8	14.3	17	0.76
1	CC041XCC012	F	Y	8.6	2	3.6	1.77
1	CC041XCC012	F	Y	11.8	7.4	24.2	0.75
1	CC041XCC012	F	Y	13.9	13	14.3	1.12
1	CC041XCC012	F	Y	10.8	4.3	15.2	0.36
1	CC041XCC012	F	Y	10.7	16.2	14.3	0.16
2	CC041XCC012	M	Y	12.1	7.7	13.4	0.79
2	CC041XCC012	M	Y	0.9	8.9	0.4	0.4
2	CC041XCC012	F	Y	12.4	8	16.6	0.29
2	CC041XCC012	M	Y	4.4	13	7.8	1.53

Trial#	Strain	Sex	Inf?	AbsolutePawAngle_LH	AbsolutePawAngle_RF	AbsolutePawAngle_RH	AtaxiaCoefficient_LF
2	CC041XCC012	M	Y	5.1	2.7	5.4	0.21
2	CC041XCC012	M	Y	7.8	2.6	4.3	0.81
2	CC041XCC012	F	Y	6.8	0.9	10	0.32
2	CC041XCC012	F	Y	2.1	3.4	7.3	1.24
2	CC041XCC012	F	Y	18.4	9	22.1	0.62
2	CC041XCC012	F	Y	14.9	3.3	21.3	0.55
2	CC041XCC012	F	Y	9.1	1.3	13.8	0.51
2	CC041XCC012	F	Y	5.4	1.2	16.7	0.63
2	CC041XCC012	F	Y	6.5	1.5	1.5	0.63
0	CC051	F	Y	28.1	10	22.6	1.49
0	CC051	F	Y	25.3	2	21	2.26
0	CC051	F	Y	19.6	3.3	21.6	1.32
0	CC051	M	Y	23.9	7.7	16.5	1.2
1	CC051	F	Y	21.5	8.9	20.5	0.89
1	CC051	F	Y	14.2	10.8	20.1	0.49
1	CC051	F	Y	14.7	6.5	17.8	1.19
0	CC057	F	Y	16.2	12	27.5	0.82
0	CC057	F	Y	28.9	8	23.4	1.3
0	CC057	F	Y	10.5	10.1	21	2.36
0	CC057	M	Y	20.4	18.2	22.8	1.86
0	CC057	M	Y	7.5	8.2	14.7	1.66
0	CC057	M	Y	22.3	7.2	16.8	2.14
1	CC057	F	Y	13.6	10.2	22.6	0.84
1	CC057	F	Y	21.6	14.6	29.2	1.25
1	CC057	F	Y	13.3	30.2	26.5	2.55
1	CC057	F	Y	12.9	14.3	21.5	2.21
1	CC057	M	Y	16.9	11.5	19	1.85
1	CC057	M	Y	12.3	10.3	15.2	0.28
2	CC057	F	Y	12.9	14.3	21.5	2.21
0	CC078	F	Y	15.6	17.4	13.9	0.28
0	CC078	F	Y	5.6	2	19.3	0.55
0	CC078	F	Y	18.5	3.2	20.8	0.35
0	CC078	M	Y	24.7	2.9	25.7	1.01
1	CC078	F	Y	17.1	22.1	11	0.92
1	CC078	F	Y	13.5	5.4	17.7	1.46
1	CC078	M	Y	11.9	2.7	8.8	0.77
2	CC078	F	Y	2	1.5	24.4	2.44
2	CC078	M	Y	2.7	6.7	6.3	1.92
2	CC005	M	N	10.2	1.9	1.2	2.8
2	CC005	M	Y	10.6	12.9	15.1	3.2
2	CC015	F	N	0.7	8.5	5.2	1.87
2	CC015	M	N	10.2	1.1	11.5	0.84
2	CC015	M	Y	12	1.2	13.4	1.05
2	CC015	M	Y	1.7	6.2	10.3	2.05
2	CC017	F	N	12	11.8	15.2	1.23
2	CC017	M	N	9.4	11.9	15.1	1.48
2	CC017	F	Y	8.9	5.7	15.8	3.38
2	CC023	F	N	21.1	2.3	13.6	1

Trial#	Strain	Sex	Inf?	AbsolutePawAngle_LH	AbsolutePawAngle_RF	AbsolutePawAngle_RH	AtaxiaCoefficient_LF

Table S3. Raw DigiGait measurement data. Far left column indicates time point at which data was measured: T0 = pre-infection, T1 = 21dpi, and T3 = 89dpi. DigiGait parameters listed across the top indicate which limb is associated with the data, where appropriate: FL for left fore limb, FR for right fore limb, HL for left hind limb, and HR for right hind limb.

Table S3

Trial#	Strain	Sex	Inf?	AtaxiaCoefficient_LH	AtaxiaCoefficient_RF	AtaxiaCoefficient_RH	AxisDistance_LF
1	CC002	F	N	1.97	1.77	1.54	0.08
1	CC025	F	N	1.58	1.15	1.78	-0.3
1	CC012XCC032	F	N	0.45	1.87	1.16	-0.68
2	CC012XCC032	F	N	0.29	0.09	0.21	-0.66
1	CC012XCC032	M	N	0.16	1.17	0.25	-0.57
2	CC012XCC032	M	N	0.42	0.27	0.56	-0.43
1	CC013xCC041	F	N	2.91	1.97	2.36	-0.83
2	CC013XCC041	F	N	0.23	0.55	0.44	-0.88
1	CC013xCC041	M	N	1.38	1.28	1.68	-1.04
2	CC013XCC041	M	N	0.46	1.15	0.5	-0.89
1	CC032XCC013	F	N	1.43	2.18	1.93	-0.14
2	CC032XCC013	F	N	1.29	1.24	0.87	-0.88
1	CC041XCC012	F	N	2.02	1.9	1.86	-0.88
1	CC041XCC012	F	N	1.97	2.71	2.11	-0.65
2	CC041XCC012	F	N	0.39	1.93	1.14	-0.13
1	CC032XCC013	M	N	1.51	1.65	1.8	-1.13
2	CC032XCC013	M	N	0.87	1.8	1.44	-0.21
1	CC041XCC012	M	N	1.19	1.84	1.4	-0.56
2	CC041XCC012	M	N	0.53	1.4	0.29	-0.41
0	CC012	F	N	1.19	1.32	1.08	-0.71
2	CC012	F	N	0.26	1.18	1.27	-0.44
0	CC012	M	N	1.59	1.41	1.28	-0.45
2	CC012	M	N	0.81	1.77	0.58	-0.69
0	CC057	F	N	0.27	0.99	1.48	-0.86
1	CC057	F	N	0.23	0.76	0.52	-0.98
0	CC057	M	N	0.61	0.44	1.2	-1.07
1	CC057	M	N	0.25	0.49	0.24	-1.06
0	CC078	F	N	0.15	0.29	0.55	-0.63
1	CC078	F	N	0.56	1.18	0.28	-0.66
2	CC078	F	N	1.39	2.45	1.67	-0.44
0	CC078	M	N	0.42	0.59	0.55	-0.45
2	CC078	M	N	0.85	2.07	1.49	-0.84
0	CC002	F	N	2.55	1.8	2.32	-0.72
1	CC002	F	N	1.97	1.77	1.54	0.08
0	CC002	M	N	1.46	2.82	2.76	-0.78
1	CC002	M	N	1.71	1.96	1.23	-0.63
2	CC002	M	N	0.28	1.14	1.53	-0.4
0	CC006	F	N	0.44	0.48	0.4	-0.76
1	CC006	F	N	1.73	1.01	0.59	-0.77
2	CC006	F	N	1.37	1.32	2.13	-0.56
0	CC006	M	N	2.5	1.57	1.76	-0.59
0	CC023	F	N	1.77	1.51	0.75	-0.78
1	CC023	F	N	2.03	2.53	1.61	-0.89
2	CC023	F	N	1.32	1.79	1.14	-0.85
0	CC023	M	N	0.32	0.33	1.12	-0.5
1	CC023	M	N	0.5	0.54	0.52	-0.61
0	CC027	F	N	1.79	1.62	3.86	-0.47

Trial#	Strain	Sex	Inf?	AtaxiaCoefficient_LH	AtaxiaCoefficient_RF	AtaxiaCoefficient_RH	AxisDistance_LF
1	CC027	F	N	2	1.84	1.78	-0.81
2	CC027	F	N	1.32	1.13	1.64	-0.8
0	CC027	M	N	1.8	1.52	2.97	-1.13
1	CC027	M	N	1.93	1.89	2.08	-0.52
1	CC027	M	N	2.07	4.25	2.11	-0.83
2	CC027	M	N	0.2	0.33	0.48	-0.95
0	CC005	F	N	0.35	0.5	0.26	-0.65
1	CC005	F	N	0.88	1.86	1.13	-0.8
2	CC005	F	N	1.93	3.09	2.68	-1.08
0	CC011	F	N	1.6	1.48	1.12	-0.62
1	CC011	F	N	1.13	2.55	1.3	-0.56
2	CC011	F	N	0.44	1.57	0.49	-0.66
1	CC017	F	N	1.38	1.62	1.29	-0.57
0	CC005	M	N	0.22	0.62	0.18	-0.77
1	CC005	M	N	2.34	1.87	1.11	-0.74
2	CC005	M	N	2.82	2.99	3.99	-0.5
0	CC011	M	N	0.57	0.43	0.65	-0.66
1	CC011	M	N	0.31	0.76	0.54	-0.84
2	CC011	M	N	2.13	4.11	3.27	-0.92
0	CC017	M	N	3.36	2.53	2	-0.79
1	CC017	M	N	0.58	0.82	1.6	-0.72
1	CC006	F	N	2.07	2.08	2.15	-0.47
0	CC037	F	N	1.24	1.1	0.63	-0.46
0	CC051	F	N	0.72	1.48	0.93	-0.77
1	CC051	F	N	1.17	1.06	0.97	-1.01
1	CC006	M	N	0.86	1.17	0.73	-0.72
0	CC037	M	N	1.02	1.2	1.19	-0.77
1	CC037	M	N	0.67	0.6	0.99	-1.2
0	CC005	F	N	1.11	1.34	1.14	-0.65
1	CC005	F	N	1.13	1.19	1.46	-0.97
2	CC005	F	N	1.24	1.13	0.23	-0.68
0	CC011	F	N	0.62	0.63	0.55	-0.58
1	CC011	F	N	0.47	0.52	0.89	-0.96
2	CC011	F	N	0.48	0.59	0.45	-0.74
0	CC011	M	N	0.5	0.71	0.35	-1.06
1	CC011	M	N	0.27	0.31	0.1	-0.98
2	CC011	M	N	0.47	0.69	0.18	-0.63
1	CC037	M	N	0.63	1.35	0.5	-0.71
2	CC037	M	N	0.83	1.6	0.58	-0.64
1	CC051	M	N	0.21	0.64	0.4	-1.01
2	CC051	M	N	0.34	1.43	0.8	-0.76
0	CC027	F	N	0.26	0.75	0.24	-0.75
1	CC027	F	N	1.12	1.06	0.99	-0.7
2	CC027	F	N	2.25	2.25	2.4	-0.59
0	CC015	M	N	0.37	0.33	0.47	-0.79
1	CC015	M	N	0.76	1.7	1.71	-0.97
2	CC015	M	N	0.56	0.96	0.42	-0.57
0	CC027	M	N	1.11	0.58	0.22	-0.5

Trial#	Strain	Sex	Inf?	AtaxiaCoefficient_LH	AtaxiaCoefficient_RF	AtaxiaCoefficient_RH	AxisDistance_LF
1	CC027	M	N	0.26	0.38	1.22	-1.06
2	CC027	M	N	1.52	0.35	0.96	-1.13
0	CC015	F	N	0.57	0.56	-0.76	-1.02
1	CC015	F	N	3.49	3.13	2.46	-0.78
0	CC017	F	N	2.1	1.31	0.39	-0.47
0	CC023	F	N	0.49	1.29	0.57	-0.88
1	CC023	F	N	0.52	1.34	0.38	-0.5
0	CC005	M	N	1.29	0.44	1.19	-0.78
1	CC005	M	N	0.78	0.45	0.57	-0.63
0	CC015	M	N	0.8	0.92	0.89	-0.58
1	CC015	M	N	0.24	2.17	1.87	-0.68
0	CC017	M	N	1.17	1.3	0.73	-0.64
1	CC017	M	N	2.64	1.96	2.04	-0.79
0	CC023	M	N	0.32	0.28	0.22	-0.84
1	CC023	M	N	1.05	0.98	1.02	-0.55
0	CC051	M	N	0.71	0.44	0.56	-0.74
0	CC002	F	Y	1.61	1.63	2.23	-0.57
0	CC002	M	Y	1.16	1.42	1.54	-0.97
1	CC002	M	Y	1.79	1.77	3.81	-0.06
1	CC002	F	Y	1.23	2.63	2.84	-0.48
1	CC002	F	Y	2.14	2.03	2.14	-0.84
1	CC002	M	Y	1.79	1.77	3.81	-0.06
1	CC002	M	Y	2.08	2.37	2.24	-0.89
1	CC002	F	Y	1.23	2.63	2.84	-0.48
1	CC002	F	Y	2.41	1.79	2.16	-0.6
2	CC002	M	Y	0.52	1.67	1.23	-0.57
2	CC002	F	Y	0.84	0.69	0.34	-0.85
2	CC002	M	Y	0.96	2.03	2.26	-0.87
0	CC005	F	Y	0.23	0.96	0.57	-0.62
0	CC005	M	Y	0.35	0.93	0.31	-0.69
0	CC005	M	Y	0.55	1.07	0.44	-0.32
1	CC005	F	Y	2.02	1.67	1.07	-0.75
1	CC005	M	Y	1.21	1.92	2.95	0.26
1	CC005	F	Y	0.15	0.75	0.4	-0.62
1	CC005	M	Y	2.29	0.82	1.06	-0.69
2	CC005	F	Y	1.84	2.84	1.86	-0.48
2	CC005	M	Y	0.71	1.28	0.75	-0.52
2	CC005	M	Y	1.37	1.61	3.02	-0.33
2	CC005	M	Y	0.71	1.28	0.75	-0.52
2	CC005	F	Y	0.64	0.53	1.35	0.1
0	CC006	F	Y	1.21	1.57	1.07	-0.4
0	CC006	M	Y	1.5	2.71	2.76	-0.75
0	CC006	F	Y	2.44	2.35	1.78	-0.57
1	CC006	F	Y	1.92	1.86	2.91	-0.69
1	CC006	M	Y	3.23	2.04	2.79	-0.75
1	CC006	F	Y	0.33	0.38	0.26	-0.61
1	CC006	M	Y	0.34	0.74	0.2	-0.79
2	CC006	F	Y	1.76	2.66	1.69	-0.61

Trial#	Strain	Sex	Inf?	AtaxiaCoefficient_LH	AtaxiaCoefficient_RF	AtaxiaCoefficient_RH	AxisDistance_LF
2	CC006	M	Y	2.25	1.41	1.73	-1
0	CC011	F	Y	1.22	2.52	0.24	-0.42
0	CC011	M	Y	1.46	1.47	1.47	-0.47
0	CC011	F	Y	0.41	0.44	0.44	-0.68
0	CC011	F	Y	1.74	1.09	1.6	-0.75
1	CC011	F	Y	0.55	0.82	0.38	-0.61
1	CC011	M	Y	0.89	0.47	0.35	-0.89
1	CC011	F	Y	0.76	0.87	0.77	-0.86
1	CC011	M	Y	0.28	0.71	0.45	-0.82
2	CC011	F	Y	0.51	0.49	0.56	-0.75
2	CC011	M	Y	1.33	2.51	0.66	-0.55
2	CC011	F	Y	0.36	1.01	0.47	-0.57
2	CC011	M	Y	0.22	0.45	0.08	-0.92
0	CC012	M	Y	0.24	0.44	0.69	-0.43
0	CC012	M	Y	1.3	1.63	1.44	-0.55
0	CC012	M	Y	0.26	0.44	0.41	-0.49
0	CC012	M	Y	1.14	2.03	2.07	-0.64
0	CC012	M	Y	0.4	1.05	1.33	-0.5
0	CC012	F	Y	1.53	1.2	0.51	-0.66
0	CC012	F	Y	0.79	1.2	0.62	-0.55
0	CC012	F	Y	2.15	1.89	1.66	-0.82
0	CC012	F	Y	2.52	2.98	3.14	-0.73
0	CC012	F	Y	0.41	0.78	0.86	-0.65
2	CC012	M	Y	0.24	1.03	0.57	-0.65
2	CC012	M	Y	0.29	0.22	0.28	-0.51
2	CC012	M	Y	0.27	0.98	0.23	-0.19
2	CC012	M	Y	0.49	0.92	1.01	-0.87
2	CC012	M	Y	0.56	0.4	0.35	-0.65
2	CC012	F	Y	0.82	0.66	0.46	-0.6
2	CC012	F	Y	0.52	0.73	0.53	-0.62
2	CC012	F	Y	1.16	2.21	2.37	-0.69
2	CC012	F	Y	0.34	1.05	0.27	-0.39
2	CC012	F	Y	0.78	1.18	0.88	-0.51
1	CC012XCC032	F	Y	3.01	2.38	3.02	-0.92
1	CC012xCC032	F	Y	2.56	1.92	1.95	-0.35
1	CC012XCC032	M	Y	1.17	1.29	0.66	-0.38
1	CC012xCC032	M	Y	1.71	1.99	1.51	-0.7
1	CC012XCC032	M	Y	0.91	1.2	1.8	-0.75
1	CC012xCC032	M	Y	1.57	1.51	2.62	-0.45
2	CC012XCC032	F	Y	0.7	1.37	0.1	-0.36
2	CC012XCC032	F	Y	0.15	1.07	1.09	-0.85
2	CC012XCC032	M	Y	0.29	2.02	1.21	-0.18
2	CC012XCC032	M	Y	0.89	1.2	0.3	-0.61
2	CC012XCC032	M	Y	0.25	0.43	0.69	-0.58
2	CC012XCC032	M	Y	0.57	0.7	1.04	-0.93
1	CC013xCC041	F	Y	1.57	0.98	2.28	-1.15
1	CC013xCC041	F	Y	2.66	2.99	1.87	-0.78
1	CC013xCC041	F	Y	2.08	2	1.86	-0.67

Trial#	Strain	Sex	Inf?	AtaxiaCoefficient_LH	AtaxiaCoefficient_RF	AtaxiaCoefficient_RH	AxisDistance_LF
1	CC013xCC041	M	Y	1.78	1.35	1.02	-0.95
1	CC013xCC041	M	Y	0.79	2.14	2.26	-0.32
2	CC013XCC041	F	Y	0.15	0.51	0.28	-0.93
2	CC013XCC041	F	Y	0.7	1.32	1.4	-1.07
2	CC013XCC041	F	Y	0.46	0.31	0.23	-0.83
2	CC013XCC041	M	Y	0.89	1.21	0.87	-0.82
2	CC013XCC041	M	Y	2.85	1.93	2.24	-0.56
0	CC015	F	Y	1.28	0.21	0.51	-0.95
0	CC015	M	Y	0.23	0.53	0.19	-0.76
0	CC015	M	Y	0.41	0.88	0.73	-0.7
1	CC015	F	Y	0.35	0.31	1.15	-0.64
1	CC015	M	Y	2.38	2.9	3.24	-0.86
1	CC015	M	Y	1.79	1.97	2.66	-0.64
1	CC015	M	Y	1.92	4.08	3.73	-0.8
2	CC015	F	Y	0.38	0.45	0.18	-0.59
0	CC017	F	Y	1.11	1.5	1.53	-0.84
0	CC017	M	Y	1.65	0.37	1.03	-0.78
0	CC017	F	Y	0.57	1.66	1.67	-0.78
0	CC017	M	Y	0.99	2.29	0.72	-0.63
1	CC017	M	Y	2.27	1.81	2.23	-0.6
1	CC017	F	Y	1.93	1.89	1.84	-0.55
0	CC023	M	Y	0.15	0.19	0.25	-0.52
0	CC023	F	Y	0.29	1.47	2.12	-0.63
0	CC023	F	Y	1.54	1.5	1.6	-0.64
0	CC023	M	Y	0.41	2.24	0.43	-0.42
1	CC023	M	Y	2.6	2.76	2.62	0.08
1	CC023	F	Y	1.63	2.73	3.02	-0.91
1	CC023	M	Y	1.18	2.69	1.44	-0.39
1	CC023	M	Y	0.17	0.85	0.61	-0.5
2	CC023	M	Y	0.96	0.31	0.17	-0.63
2	CC023	F	Y	1.07	1.48	1.31	-0.76
2	CC023	M	Y	0.62	1.8	2.23	-0.8
0	CC025	M	Y	1.87	1.15	0.95	-0.93
0	CC025	F	Y	1.32	0.74	0.63	-1.18
0	CC025	M	Y	0.14	0.75	0.44	-0.78
1	CC025	M	Y	1.77	1.4	0.96	-0.95
1	CC025	F	Y	1.97	1.25	2.1	-1.66
1	CC025	F	Y	1.25	1.12	0.88	-1.18
1	CC025	F	Y	0.41	1.24	1.23	-0.63
2	CC025	F	Y	0.76	0.39	0.67	-0.82
2	CC025	M	Y	0.29	0.43	0.4	-0.25
0	CC027	F	Y	1.22	0.56	0.58	-0.37
0	CC027	M	Y	0.79	2.6	0.8	-0.19
1	CC027	M	Y	0.71	0.97	0.89	-1.38
1	CC027	M	Y	0.71	0.97	0.89	-1.38
1	CC027	F	Y	1.58	2.01	2.41	-1.06
1	CC027	F	Y	1.68	1.73	2.33	-0.54
1	CC027	M	Y	0.13	0.16	0.15	-0.3

Trial#	Strain	Sex	Inf?	AtaxiaCoefficient_LH	AtaxiaCoefficient_RF	AtaxiaCoefficient_RH	AxisDistance_LF
1	CC027	M	Y	0.3	0.63	0.43	-0.2
2	CC027	F	Y	1.07	1.48	1.31	-0.76
2	CC027	M	Y	1.73	0.65	0.64	-0.54
2	CC027	F	Y	1.26	2.05	1.66	-0.66
2	CC027	M	Y	0.37	0.38	0.26	-1.23
1	CC032XCC013	M	Y	1.26	2.27	1.9	-0.54
1	CC032XCC013	M	Y	0.54	0.86	0.71	-0.85
1	CC032XCC013	M	Y	1.12	1.66	1.2	-0.33
1	CC032XCC013	F	Y	1.96	1.22	1.73	-0.82
1	CC032XCC013	F	Y	0.62	1.89	1.16	-0.9
1	CC032XCC013	F	Y	0.76	0.52	0.31	-0.77
1	CC032XCC013	F	Y	0.44	0.52	0.31	-0.78
1	CC032XCC013	F	Y	0.67	0.48	0.37	-0.63
2	CC032XCC013	M	Y	0.25	0.99	0.5	-0.81
2	CC032XCC013	M	Y	1.17	1.87	1.17	-0.53
2	CC032XCC013	M	Y	1.42	3.44	1.23	-1.46
2	CC032XCC013	M	Y	0.27	2.24	1.39	-0.84
2	CC032XCC013	F	Y	0.41	0.76	0.32	-0.55
2	CC032XCC013	F	Y	0.37	0.92	0.38	-0.91
2	CC032XCC013	F	Y	0.71	1.15	0.32	-0.65
2	CC032XCC013	F	Y	0.22	0.64	0.63	-0.77
0	CC037	F	Y	2.19	2.82	1.84	-0.72
0	CC037	M	Y	1.58	2	2.49	-0.68
0	CC037	M	Y	0.15	0.93	0.16	-0.74
0	CC037	M	Y	1.73	1.14	1.36	-0.59
1	CC037	F	Y	1.81	1.11	1.19	-0.69
1	CC037	M	Y	0.46	0.66	0.23	-0.64
1	CC037	M	Y	0.53	1.62	0.88	-0.76
1	CC037	M	Y	0.35	0.37	1.37	-0.64
2	CC037	M	Y	0.25	1.87	0.48	-1.06
2	CC037	M	Y	0.78	0.6	0.22	-0.7
1	CC041XCC012	M	Y	1.76	1.28	1.28	-0.44
1	CC041XCC012	M	Y	0.14	0.57	0.41	-0.47
1	CC041XCC012	M	Y	1.89	1.71	2.05	-0.71
1	CC041XCC012	M	Y	1.35	2.13	1.6	-0.62
1	CC041XCC012	M	Y	0.23	1.41	0.51	-0.67
1	CC041XCC012	F	Y	0.59	2.3	1.05	-0.36
1	CC041XCC012	F	Y	0.23	1.62	1.19	-0.01
1	CC041XCC012	F	Y	0.34	1.25	0.96	-0.61
1	CC041XCC012	F	Y	0.39	1.16	0.4	-0.6
1	CC041XCC012	F	Y	0.78	1.78	1.13	-0.43
1	CC041XCC012	F	Y	0.66	0.91	0.73	-0.57
1	CC041XCC012	F	Y	0.31	0.27	0.48	-0.51
1	CC041XCC012	F	Y	1.74	2.53	1.31	-0.5
2	CC041XCC012	M	Y	0.25	0.24	1	-0.69
2	CC041XCC012	M	Y	0.62	1.08	0.62	-0.49
2	CC041XCC012	F	Y	0.33	1.09	0.23	-0.52
2	CC041XCC012	M	Y	1.32	0.75	0.23	-0.94

Trial#	Strain	Sex	Inf?	AtaxiaCoefficient_LH	AtaxiaCoefficient_RF	AtaxiaCoefficient_RH	AxisDistance_LF
2	CC041XCC012	M	Y	0.4	0.55	0.76	-0.8
2	CC041XCC012	M	Y	1.17	1.62	0.52	-0.79
2	CC041XCC012	F	Y	0.39	0.45	0.29	-0.62
2	CC041XCC012	F	Y	0.92	1.25	1.17	-0.86
2	CC041XCC012	F	Y	0.23	1.09	0.41	-0.88
2	CC041XCC012	F	Y	0.41	0.46	0.43	-0.7
2	CC041XCC012	F	Y	0.68	0.77	0.55	-0.68
2	CC041XCC012	F	Y	0.18	0.65	0.25	-0.99
2	CC041XCC012	F	Y	0.57	1.06	0.66	-0.5
0	CC051	F	Y	0.79	1.37	1.26	-0.51
0	CC051	F	Y	1.89	2.52	2.02	-0.86
0	CC051	F	Y	1.52	1.59	1.37	-0.72
0	CC051	M	Y	0.34	0.72	0.45	-0.76
1	CC051	F	Y	0.62	0.71	0.18	-0.58
1	CC051	F	Y	0.53	1.18	0.38	-0.64
1	CC051	F	Y	0.4	0.9	0.63	-0.74
0	CC057	F	Y	0.5	0.74	0.3	-0.98
0	CC057	F	Y	1.19	1.31	1.22	-1.09
0	CC057	F	Y	1.23	1.74	2.07	-0.72
0	CC057	M	Y	1.64	1.53	1.32	-1.16
0	CC057	M	Y	1.7	2.06	2.2	-0.09
0	CC057	M	Y	2.15	2.15	1.99	-0.99
1	CC057	F	Y	0.19	0.28	0.14	-1.03
1	CC057	F	Y	1.25	1.41	1.61	-1.05
1	CC057	F	Y	2.17	2.01	1.79	-0.67
1	CC057	F	Y	2.66	2.48	2.14	-1.22
1	CC057	M	Y	0.6	1.33	0.26	-1.13
1	CC057	M	Y	0.66	0.38	0.55	-1.19
2	CC057	F	Y	2.66	2.48	2.14	-1.22
0	CC078	F	Y	0.16	0.88	1.33	-0.64
0	CC078	F	Y	0.84	0.66	1.13	-0.55
0	CC078	F	Y	0.27	0.29	0.24	-0.53
0	CC078	M	Y	0.41	0.63	0.34	-0.62
1	CC078	F	Y	0.28	0.24	0.22	-0.59
1	CC078	F	Y	0.31	1.07	1.22	-0.35
1	CC078	M	Y	0.34	0.85	0.59	-0.57
2	CC078	F	Y	2.72	2.18	3.08	-0.53
2	CC078	M	Y	1.78	1.28	1.59	-0.99
2	CC005	M	N	1.98	2.13	1.59	-0.16
2	CC005	M	Y	3.5	5.05	3.51	-0.07
2	CC015	F	N	2.35	2.57	2.03	-0.88
2	CC015	M	N	0.63	0.76	0.31	-0.8
2	CC015	M	Y	0.63	1.4	0.43	-0.92
2	CC015	M	Y	1.52	1.97	2.04	-0.75
2	CC017	F	N	0.42	2.43	1.48	-0.77
2	CC017	M	N	1.14	1.62	0.93	-0.57
2	CC017	F	Y	2.67	2.04	3.54	-0.61
2	CC023	F	N	1.16	0.9	0.73	-0.65

Trial#	Strain	Sex	Inf?	AtaxiaCoefficient_LH	AtaxiaCoefficient_RF	AtaxiaCoefficient_RH	AxisDistance_LF

Table S3. Raw DigiGait measurement data. Far left column indicates time point at which data was measured: T0 = pre-infection, T1 = 21dpi, and T3 = 89dpi. DigiGait parameters listed across the top indicate which limb is associated with the data, where appropriate: FL for left fore limb, FR for right fore limb, HL for left hind limb, and HR for right hind limb.

Table S3

Trial#	Strain	Sex	Inf?	AxisDistance_LH	AxisDistance_RF	AxisDistance_RH	Brake_LF	Brake_LH	Brake_RF
1	CC002	F	N	-0.89	0.9	1.72	0.047	0.055	0.042
1	CC025	F	N	-0.96	0.72	1.03	0.074	0.087	0.077
1	CC012XCC032	F	N	-1.42	0.62	1.59	0.083	0.044	0.065
2	CC012XCC032	F	N	-1.46	0.73	1.3	0.078	0.111	0.11
1	CC012XCC032	M	N	-0.91	0.55	0.94	0.076	0.013	0.098
2	CC012XCC032	M	N	-1.07	0.46	0.9	0.14	0.023	0.152
1	CC013xCC041	F	N	-1.47	0.64	1.08	0.075	0.039	0.073
2	CC013XCC041	F	N	-1.55	1.04	1.76	0.14	0.044	0.184
1	CC013xCC041	M	N	-1.56	0.19	1.21	0.021	0.025	0.05
2	CC013XCC041	M	N	-1.38	0.59	1.35	0.079	0.052	0.084
1	CC032XCC013	F	N	-1.34	0.14	1.01	0.051	0.029	0.032
2	CC032XCC013	F	N	-1.22	0.1	0.8	0.087	0.059	0.077
1	CC041XCC012	F	N	-0.96	0.56	0.96	0.073	0.029	0.108
1	CC041XCC012	F	N	-0.52	0.71	1.08	0.045	0.025	0.034
2	CC041XCC012	F	N	-1.15	0.92	1.3	0.05	0.095	0.036
1	CC032XCC013	M	N	-1.2	1.22	0.83	0.074	0.028	0.053
2	CC032XCC013	M	N	-1.44	0.88	1.21	0.023	0.098	0.039
1	CC041XCC012	M	N	-1.5	0.88	1.39	0.11	0.067	0.071
2	CC041XCC012	M	N	-1.64	0.85	1.33	0.155	0.05	0.141
0	CC012	F	N	-1.06	0.78	1.17	0.036	0.024	0.062
2	CC012	F	N	-1.49	1.24	1.07	0.172	0.079	0.135
0	CC012	M	N	-1.19	0.66	0.75	0.055	0.011	0.08
2	CC012	M	N	-1.2	0.46	1.86	0.061	0.054	0.059
0	CC057	F	N	-1.4	1.09	1.5	0.084	0.082	0.077
1	CC057	F	N	-1.71	0.98	1.74	0.076	0.058	0.069
0	CC057	M	N	-1.39	0.71	1.59	0.065	0.101	0.111
1	CC057	M	N	-1.96	1.07	1.86	0.075	0.064	0.076
0	CC078	F	N	-1.22	0.69	1.13	0.049	0.019	0.05
1	CC078	F	N	-1.07	0.78	0.95	0.099	0.018	0.08
2	CC078	F	N	-0.81	0.77	0.8	0.047	0.017	0.041
0	CC078	M	N	-0.97	0.62	1.13	0.027	0.015	0.057
2	CC078	M	N	-1.13	0.86	1.21	0.046	0.058	0.068
0	CC002	F	N	-0.72	0.38	0.53	0.066	0.026	0.064
1	CC002	F	N	-0.89	0.9	1.72	0.047	0.055	0.042
0	CC002	M	N	-0.94	0.44	0.96	0.04	0.028	0.063
1	CC002	M	N	-0.61	0.76	0.98	0.057	0.063	0.081
2	CC002	M	N	-0.83	1.08	1.3	0.169	0.047	0.141
0	CC006	F	N	-1.02	0.76	1.05	0.144	0.008	0.089
1	CC006	F	N	-1.13	0.62	1.34	0.082	0.042	0.122
2	CC006	F	N	-1.03	0.74	1.04	0.055	0.058	0.063
0	CC006	M	N	-0.78	0.75	0.77	0.074	0.056	0.101
0	CC023	F	N	-0.9	0.61	1.11	0.079	0.067	0.062
1	CC023	F	N	-0.89	0.9	1.3	0.055	0.026	0.046
2	CC023	F	N	-0.8	0.36	1.27	0.094	0.094	0.038
0	CC023	M	N	-1.24	0.56	0.99	0.046	0.026	0.046
1	CC023	M	N	-1.24	0.63	1.21	0.052	0.046	0.067
0	CC027	F	N	-1.02	0.21	0.08	0.032	0.017	0.026

Trial#	Strain	Sex	Inf?	AxisDistance_LH	AxisDistance_RF	AxisDistance_RH	Brake_LF	Brake_LH	Brake_RF
1	CC027	F	N	-0.92	0.91	1.02	0.08	0.098	0.057
2	CC027	F	N	-1.37	0.96	1.33	0.05	0.074	0.096
0	CC027	M	N	-1.39	0.69	0.63	0.03	0.016	0.031
1	CC027	M	N	-0.81	0.95	1.5	0.028	0.027	0.026
1	CC027	M	N	-0.99	0.57	0.75	0.123	0.108	0.108
2	CC027	M	N	-1.24	1.07	1.44	0.084	0.039	0.083
0	CC005	F	N	-0.98	0.67	1.22	0.054	0.037	0.071
1	CC005	F	N	-1.21	0.32	1.12	0.068	0.125	0.092
2	CC005	F	N	-0.77	0.83	0.73	0.051	0.03	0.053
0	CC011	F	N	-0.78	0.51	0.83	0.039	0.048	0.028
1	CC011	F	N	-1.06	0.3	0.8	0.041	0.013	0.04
2	CC011	F	N	-1.06	0.59	0.78	0.072	0.031	0.076
1	CC017	F	N	-1.2	0.92	1.31	0.061	0.047	0.077
0	CC005	M	N	-1.23	0.66	1.33	0.105	0.037	0.084
1	CC005	M	N	-1.07	0.31	1.27	0.07	0.022	0.087
2	CC005	M	N	-1.47	0.09	0.76	0.057	0.028	0.057
0	CC011	M	N	-0.92	0.66	0.81	0.051	0.036	0.065
1	CC011	M	N	-0.95	0.69	0.78	0.075	0.056	0.066
2	CC011	M	N	-1.01	0.83	0.94	0.075	0.032	0.067
0	CC017	M	N	-1.26	0.7	0.7	0.036	0.026	0.033
1	CC017	M	N	-1.33	0.45	1.09	0.043	0.037	0.116
1	CC006	F	N	-0.53	0.39	0.17	0.044	0.016	0.031
0	CC037	F	N	-0.99	0.54	1.01	0.068	0.057	0.054
0	CC051	F	N	-1.17	0.54	1.12	0.039	0.045	0.057
1	CC051	F	N	-1.24	0.21	1.2	0.031	0.035	0.061
1	CC006	M	N	-0.98	0.58	1.11	0.078	0.048	0.099
0	CC037	M	N	-1.04	0.62	1.19	0.062	0.085	0.078
1	CC037	M	N	-0.98	0.59	1.14	0.08	0.034	0.132
0	CC005	F	N	-1.15	0.52	1.15	0.07	0.042	0.077
1	CC005	F	N	-1.53	0.97	1.44	0.098	0.076	0.114
2	CC005	F	N	-1.3	0.7	1.23	0.115	0.059	0.04
0	CC011	F	N	-0.75	0.5	1.04	0.058	0.025	0.077
1	CC011	F	N	-1.25	1.1	1.31	0.058	0.062	0.044
2	CC011	F	N	-0.92	0.69	0.83	0.108	0.11	0.107
0	CC011	M	N	-1.61	0.9	1.54	0.088	0.053	0.068
1	CC011	M	N	-1.69	1	1.64	0.09	0.081	0.046
2	CC011	M	N	-1.3	0.94	1.34	0.105	0.086	0.061
1	CC037	M	N	-1.31	0.68	1.42	0.113	0.072	0.064
2	CC037	M	N	-1.41	0.63	1.32	0.041	0.068	0.047
1	CC051	M	N	-1.91	1.19	1.78	0.065	0.074	0.082
2	CC051	M	N	-0.91	0.53	0.91	0.079	0.085	0.088
0	CC027	F	N	-1.41	0.61	1.12	0.071	0.05	0.14
1	CC027	F	N	-1.05	0.77	1.18	0.083	0.053	0.054
2	CC027	F	N	-1.12	0.79	1.27	0.094	0.063	0.103
0	CC015	M	N	-1.14	0.63	0.72	0.06	0.056	0.046
1	CC015	M	N	-0.72	0.45	0.79	0.063	0.043	0.085
2	CC015	M	N	-1.2	0.95	1.06	0.094	0.08	0.07
0	CC027	M	N	-0.95	0.59	0.92	0.085	0.041	0.043

Trial#	Strain	Sex	Inf?	AxisDistance_LH	AxisDistance_RF	AxisDistance_RH	Brake_LF	Brake_LH	Brake_RF
1	CC027	M	N	-1.44	1.03	1.15	0.077	0.06	0.097
2	CC027	M	N	-1.1	0.74	0.93	0.061	0.029	0.07
0	CC015	F	N	0.68	1.17	0.064	0.088	0.054	0.108
1	CC015	F	N	-1.1	0.87	1.28	0.052	0.04	0.048
0	CC017	F	N	-1.03	0.51	0.93	0.078	0.055	0.074
0	CC023	F	N	-1.29	0.71	1.16	0.064	0.098	0.099
1	CC023	F	N	-1.24	0.67	1.18	0.094	0.071	0.064
0	CC005	M	N	-1.05	0.48	1.4	0.079	0.024	0.126
1	CC005	M	N	-1.45	0.82	1.4	0.18	0.029	0.198
0	CC015	M	N	-0.94	0.78	0.99	0.088	0.048	0.1
1	CC015	M	N	-0.9	1.06	1.12	0.064	0.087	0.07
0	CC017	M	N	-0.75	0.83	1.09	0.071	0.101	0.066
1	CC017	M	N	-1.05	0.81	1.18	0.054	0.022	0.049
0	CC023	M	N	-1.2	0.61	1.22	0.093	0.052	0.071
1	CC023	M	N	-1.09	0.65	1.18	0.096	0.04	0.091
0	CC051	M	N	-1.17	0.84	1.41	0.135	0.037	0.097
0	CC002	F	Y	-0.69	0.36	0.88	0.044	0.066	0.065
0	CC002	M	Y	-1.1	0.78	1.06	0.067	0.105	0.077
1	CC002	M	Y	-0.36	0.78	0.84	0.057	0.032	0.05
1	CC002	F	Y	-1.26	0.17	0.44	0.027	0.056	0.063
1	CC002	F	Y	-0.93	0.36	1.08	0.037	0.051	0.039
1	CC002	M	Y	-0.36	0.78	0.84	0.057	0.032	0.05
1	CC002	M	Y	-1.09	0.62	0.93	0.057	0.05	0.058
1	CC002	F	Y	-1.26	0.17	0.44	0.027	0.056	0.063
1	CC002	F	Y	-0.72	0.39	0.35	0.045	0.018	0.038
2	CC002	M	Y	-1.46	0.53	0.89	0.046	0.049	0.039
2	CC002	F	Y	-0.83	0.79	0.99	0.084	0.072	0.09
2	CC002	M	Y	-0.83	0.89	0.73	0.041	0.032	0.057
0	CC005	F	Y	-0.81	0.64	1.08	0.056	0.059	0.062
0	CC005	M	Y	-1.05	0.52	1.21	0.051	0.021	0.057
0	CC005	M	Y	-1.16	0.86	1.17	0.132	0.043	0.117
1	CC005	F	Y	-0.91	0.5	0.99	0.045	0.029	0.058
1	CC005	M	Y	-1.39	1.29	0.83	0.027	0.03	0.031
1	CC005	F	Y	-2.03	0.83	1.83	0.126	0.057	0.17
1	CC005	M	Y	-0.92	0.67	1.25	0.073	0.035	0.102
2	CC005	F	Y	-1.06	0.52	0.7	0.06	0.041	0.078
2	CC005	M	Y	-1.68	0.79	1.26	0.077	0.105	0.123
2	CC005	M	Y	-1.38	0.5	0.47	0.097	0.035	0.068
2	CC005	M	Y	-1.68	0.79	1.26	0.077	0.105	0.123
2	CC005	F	Y	-1.36	0.79	1.07	0.153	0.065	0.198
0	CC006	F	Y	-0.89	0.59	0.94	0.069	0.039	0.046
0	CC006	M	Y	-0.83	0.41	0.26	0.054	0.028	0.03
0	CC006	F	Y	-0.88	0.61	0.98	0.056	0.038	0.053
1	CC006	F	Y	-0.91	0.51	0.76	0.045	0.037	0.04
1	CC006	M	Y	-0.76	0.5	0.58	0.044	0.029	0.044
1	CC006	F	Y	-0.97	0.59	0.89	0.121	0.023	0.098
1	CC006	M	Y	-1.11	0.61	1.38	0.064	0.069	0.154
2	CC006	F	Y	-1.06	0.57	0.83	0.057	0.016	0.075

Trial#	Strain	Sex	Inf?	AxisDistance_LH	AxisDistance_RF	AxisDistance_RH	Brake_LF	Brake_LH	Brake_RF
2	CC006	M	Y	-1.03	0.97	0.98	0.061	0.028	0.109
0	CC011	F	Y	-0.69	0.06	0.84	0.021	0.008	0.023
0	CC011	M	Y	-0.8	0.63	0.88	0.044	0.035	0.051
0	CC011	F	Y	-1.01	0.67	0.9	0.041	0.029	0.047
0	CC011	F	Y	-0.61	0.57	0.33	0.076	0.016	0.056
1	CC011	F	Y	-0.99	0.85	0.69	0.07	0.061	0.099
1	CC011	M	Y	-1.15	0.79	0.78	0.111	0.071	0.068
1	CC011	F	Y	-1.03	0.89	1.3	0.115	0.082	0.074
1	CC011	M	Y	-1.15	1.15	1.41	0.094	0.076	0.125
2	CC011	F	Y	-0.92	0.77	0.73	0.045	0.06	0.087
2	CC011	M	Y	-1.14	0.29	0.87	0.058	0.034	0.065
2	CC011	F	Y	-0.91	0.63	0.94	0.116	0.079	0.107
2	CC011	M	Y	-1.39	1.03	1.41	0.135	0.087	0.112
0	CC012	M	Y	-1.23	0.71	0.96	0.066	0.042	0.06
0	CC012	M	Y	-0.9	0.75	1.19	0.089	0.059	0.072
0	CC012	M	Y	-1.06	0.7	1.02	0.057	0.029	0.062
0	CC012	M	Y	-0.76	0.61	1.04	0.045	0.025	0.063
0	CC012	M	Y	-1.12	0.68	1.01	0.108	0.052	0.073
0	CC012	F	Y	-0.91	0.59	0.91	0.043	0.044	0.053
0	CC012	F	Y	-1.02	0.8	1.3	0.054	0.056	0.094
0	CC012	F	Y	-1.05	0.64	1.19	0.055	0.039	0.081
0	CC012	F	Y	-1.31	0.5	0.99	0.066	0.031	0.044
0	CC012	F	Y	-0.61	0.63	0.98	0.023	0.012	0.039
2	CC012	M	Y	-1	0.71	1.11	0.133	0.018	0.073
2	CC012	M	Y	-1.03	0.61	0.98	0.116	0.043	0.082
2	CC012	M	Y	-1.4	0.88	1.1	0.046	0.029	0.122
2	CC012	M	Y	-1.17	0.07	1.13	0.095	0.054	0.166
2	CC012	M	Y	-1.24	0.83	1.16	0.122	0.071	0.113
2	CC012	F	Y	-1.11	0.57	1.02	0.068	0.137	0.087
2	CC012	F	Y	-1.53	0.81	1.16	0.095	0.06	0.107
2	CC012	F	Y	-0.86	0.34	1.19	0.04	0.078	0.059
2	CC012	F	Y	-1.56	0.78	0.3	0.067	0.062	0.065
2	CC012	F	Y	-1.56	0.36	1.27	0.063	0.085	0.079
1	CC012XCC032	F	Y	-0.44	0.91	0.43	0.037	0.039	0.028
1	CC012xCC032	F	Y	-0.82	0.32	0.91	0.036	0.028	0.04
1	CC012XCC032	M	Y	-1.35	0.44	1.18	0.094	0.074	0.067
1	CC012xCC032	M	Y	-0.9	0.6	1.03	0.066	0.031	0.078
1	CC012XCC032	M	Y	-1.38	1.01	0.3	0.037	0.014	0.049
1	CC012xCC032	M	Y	-0.78	0.47	1.19	0.044	0.057	0.039
2	CC012XCC032	F	Y	-1.44	0.83	1.35	0.128	0.098	0.068
2	CC012XCC032	F	Y	-1.57	0.82	1.46	0.144	0.089	0.094
2	CC012XCC032	M	Y	-1.02	0.76	1.36	0.057	0.076	0.052
2	CC012XCC032	M	Y	-1.18	0.43	1.11	0.057	0.058	0.064
2	CC012XCC032	M	Y	-1.44	0.67	1.43	0.127	0.084	0.079
2	CC012XCC032	M	Y	-1.22	0.8	0.76	0.128	0.015	0.119
1	CC013xCC041	F	Y	-1.56	0.56	0.58	0.057	0.021	0.099
1	CC013xCC041	F	Y	-1.23	0.14	0.3	0.03	0.018	0.039
1	CC013xCC041	F	Y	-1.1	0.14	0.94	0.036	0.036	0.028

Trial#	Strain	Sex	Inf?	AxisDistance_LH	AxisDistance_RF	AxisDistance_RH	Brake_LF	Brake_LH	Brake_RF
1	CC013xCC041	M	Y	-1.06	0.94	1.41	0.064	0.016	0.07
1	CC013xCC041	M	Y	-1.46	0.75	1.1	0.072	0.056	0.037
2	CC013XCC041	F	Y	-1.59	1.05	1.49	0.091	0.078	0.096
2	CC013XCC041	F	Y	-1.54	1.2	1.61	0.09	0.09	0.089
2	CC013XCC041	F	Y	-1.5	0.87	1.29	0.139	0.036	0.171
2	CC013XCC041	M	Y	-1.22	0.86	1.36	0.082	0.07	0.08
2	CC013XCC041	M	Y	-1.02	1.04	0.48	0.035	0.014	0.034
0	CC015	F	Y	-1.29	0.42	0.5	0.092	0.05	0.051
0	CC015	M	Y	-1.34	0.71	1.22	0.102	0.065	0.088
0	CC015	M	Y	-1.05	0.72	1.01	0.086	0.083	0.084
1	CC015	F	Y	-1.44	0.94	1.09	0.079	0.065	0.085
1	CC015	M	Y	-1.14	0.89	1.45	0.045	0.037	0.063
1	CC015	M	Y	-0.94	0.66	0.91	0.059	0.041	0.066
1	CC015	M	Y	-1.5	0.81	1.14	0.086	0.064	0.08
2	CC015	F	Y	-1.43	1.04	1.28	0.095	0.102	0.121
0	CC017	F	Y	-1.01	0.86	1.11	0.029	0.026	0.033
0	CC017	M	Y	-0.58	0.3	0.96	0.06	0.011	0.063
0	CC017	F	Y	-0.95	0.71	0.79	0.067	0.054	0.062
0	CC017	M	Y	-1.01	0.92	1.04	0.093	0.076	0.04
1	CC017	M	Y	-1.17	0.81	1.21	0.06	0.042	0.066
1	CC017	F	Y	-1.16	0.73	0.78	0.051	0.034	0.095
0	CC023	M	Y	-1.16	0.61	1.29	0.023	0.025	0.025
0	CC023	F	Y	-1.18	0.54	0.44	0.044	0.035	0.074
0	CC023	F	Y	-0.77	0.64	0.89	0.045	0.038	0.032
0	CC023	M	Y	-0.8	0.51	1.25	0.021	0.024	0.056
1	CC023	M	Y	-0.47	0.86	1.14	0.028	0.029	0.037
1	CC023	F	Y	-0.89	-0.29	0.7	0.04	0.024	0.031
1	CC023	M	Y	-1.03	0.5	1.17	0.065	0.066	0.067
1	CC023	M	Y	-0.97	0.36	1.31	0.079	0.057	0.083
2	CC023	M	Y	-1.05	0.32	1.24	0.133	0.021	0.063
2	CC023	F	Y	-1.43	0.93	1.18	0.119	0.038	0.052
2	CC023	M	Y	-1.23	-0.1	0.87	0.04	0.027	0.061
0	CC025	M	Y	-0.85	0.71	1.14	0.038	0.06	0.062
0	CC025	F	Y	-1.16	-0.39	0.59	0.048	0.07	0.096
0	CC025	M	Y	-1.28	0.83	1.31	0.089	0.015	0.064
1	CC025	M	Y	-0.81	0.64	1.15	0.036	0.058	0.061
1	CC025	F	Y	-2.37	-0.39	-0.23	0.038	0.03	0.04
1	CC025	F	Y	-1.97	0.03	0.29	0.058	0.059	0.061
1	CC025	F	Y	-1.32	0.43	0.56	0.044	0.054	0.047
2	CC025	F	Y	-1.03	0.85	1.02	0.128	0.068	0.079
2	CC025	M	Y	-1.26	1.17	1.42	0.103	0.107	0.134
0	CC027	F	Y	-0.83	0.22	0.72	0.082	0.029	0.064
0	CC027	M	Y	-1.08	0.62	1.29	0.042	0.021	0.052
1	CC027	M	Y	-1.57	0.18	0.72	0.046	0.071	0.036
1	CC027	M	Y	-1.57	0.18	0.72	0.046	0.071	0.036
1	CC027	F	Y	-1.4	0.3	0.67	0.027	0.02	0.035
1	CC027	F	Y	-0.59	0.66	1.2	0.072	0.08	0.05
1	CC027	M	Y	-1.07	1.67	1.34	0.105	0.171	0.248

Trial#	Strain	Sex	Inf?	AxisDistance_LH	AxisDistance_RF	AxisDistance_RH	Brake_LF	Brake_LH	Brake_RF
1	CC027	M	Y	-1.15	1.5	1.34	0.131	0.062	0.106
2	CC027	F	Y	-1.43	0.93	1.18	0.119	0.038	0.052
2	CC027	M	Y	-1.5	0.88	1.23	0.065	0.048	0.085
2	CC027	F	Y	-1.14	0.78	0.63	0.063	0.069	0.056
2	CC027	M	Y	-1.46	0.77	1.18	0.108	0.057	0.135
1	CC032XCC013	M	Y	-1.09	1.42	1.57	0.114	0.069	0.074
1	CC032XCC013	M	Y	-1.02	0.8	1.88	0.106	0.074	0.076
1	CC032XCC013	M	Y	-0.78	0.75	1.65	0.032	0.029	0.041
1	CC032XCC013	F	Y	-1.31	0.38	1.41	0.034	0.04	0.066
1	CC032XCC013	F	Y	-1.5	0.91	1.35	0.056	0.054	0.05
1	CC032XCC013	F	Y	-1.29	0.81	1.51	0.098	0.073	0.088
1	CC032XCC013	F	Y	-1.29	0.91	1.43	0.131	0.082	0.086
1	CC032XCC013	F	Y	-1.15	1.07	2	0.066	0.087	0.024
2	CC032XCC013	M	Y	-1.32	0.57	1.37	0.061	0.073	0.122
2	CC032XCC013	M	Y	-1.16	1.1	1.3	0.08	0.023	0.058
2	CC032XCC013	M	Y	-1.83	-0.47	0.73	0.029	0.058	0.061
2	CC032XCC013	M	Y	-1.28	0.83	1.31	0.096	0.088	0.053
2	CC032XCC013	F	Y	-1.21	1.05	1.37	0.103	0.107	0.084
2	CC032XCC013	F	Y	-1.5	0.75	1.67	0.094	0.098	0.083
2	CC032XCC013	F	Y	-1.25	0.95	1.42	0.099	0.087	0.05
2	CC032XCC013	F	Y	-1.03	0.84	1.36	0.094	0.078	0.055
0	CC037	F	Y	-1.35	0.54	1.19	0.053	0.044	0.057
0	CC037	M	Y	-1.09	0.5	0.86	0.031	0.034	0.035
0	CC037	M	Y	-1.11	0.45	1.07	0.04	0.05	0.057
0	CC037	M	Y	-0.89	0.32	0.01	0.061	0.027	0.026
1	CC037	F	Y	-1.11	0.76	1.15	0.097	0.126	0.138
1	CC037	M	Y	-1.11	0.69	1.22	0.073	0.046	0.053
1	CC037	M	Y	-1.43	0.88	1.36	0.101	0.096	0.094
1	CC037	M	Y	-1.04	0.74	0.86	0.087	0.105	0.074
2	CC037	M	Y	-1.48	0.86	1.39	0.12	0.119	0.048
2	CC037	M	Y	-1.13	0.88	1.17	0.08	0.094	0.087
1	CC041XCC012	M	Y	-0.81	0.61	1.02	0.119	0.03	0.084
1	CC041XCC012	M	Y	-0.98	0.88	1.5	0.07	0.053	0.133
1	CC041XCC012	M	Y	-0.9	0.89	1.44	0.102	0.036	0.079
1	CC041XCC012	M	Y	-0.91	0.76	1.14	0.072	0.05	0.081
1	CC041XCC012	M	Y	-1.27	0.72	1.08	0.102	0.082	0.093
1	CC041XCC012	F	Y	-1.49	0.88	1.29	0.095	0.125	0.089
1	CC041XCC012	F	Y	-1.04	0.93	1.08	0.062	0.068	0.064
1	CC041XCC012	F	Y	-0.85	0.49	1.12	0.105	0.054	0.061
1	CC041XCC012	F	Y	-1.25	1.03	1.26	0.119	0.143	0.126
1	CC041XCC012	F	Y	-1.13	0.46	1.27	0.107	0.052	0.088
1	CC041XCC012	F	Y	-1.5	0.85	1.09	0.084	0.117	0.08
1	CC041XCC012	F	Y	-0.83	0.78	1.19	0.103	0.074	0.097
1	CC041XCC012	F	Y	-1.17	0.98	1.18	0.094	0.056	0.086
2	CC041XCC012	M	Y	-1.01	0.94	1.16	0.115	0.081	0.129
2	CC041XCC012	M	Y	-1.34	0.84	1.17	0.168	0.105	0.166
2	CC041XCC012	F	Y	-1.4	0.86	1.02	0.112	0.075	0.101
2	CC041XCC012	M	Y	-0.83	0.39	1.37	0.088	0.038	0.126

Trial#	Strain	Sex	Inf?	AxisDistance_LH	AxisDistance_RF	AxisDistance_RH	Brake_LF	Brake_LH	Brake_RF
2	CC041XCC012	M	Y	-1.25	0.55	1.21	0.108	0.072	0.131
2	CC041XCC012	M	Y	-0.97	0.39	1.21	0.078	0.015	0.132
2	CC041XCC012	F	Y	-1.52	0.84	1.28	0.152	0.127	0.172
2	CC041XCC012	F	Y	-1.37	0.91	1.91	0.118	0.061	0.119
2	CC041XCC012	F	Y	-1.16	0.82	1.31	0.113	0.117	0.157
2	CC041XCC012	F	Y	-1.22	0.72	1.21	0.099	0.082	0.112
2	CC041XCC012	F	Y	-1.27	0.71	1.2	0.096	0.117	0.115
2	CC041XCC012	F	Y	-1.55	0.95	1.37	0.135	0.081	0.153
2	CC041XCC012	F	Y	-1.02	0.81	1.19	0.102	0.038	0.123
0	CC051	F	Y	-1.18	0.8	1.2	0.074	0.078	0.092
0	CC051	F	Y	-1.46	0.56	1.36	0.046	0.047	0.039
0	CC051	F	Y	-1.07	0.67	1.18	0.061	0.041	0.073
0	CC051	M	Y	-1.22	0.85	1.2	0.072	0.049	0.066
1	CC051	F	Y	-1.42	1	1.34	0.073	0.079	0.13
1	CC051	F	Y	-1.4	1.13	1.39	0.082	0.081	0.084
1	CC051	F	Y	-1.11	0.77	1.47	0.08	0.085	0.08
0	CC057	F	Y	-1.42	0.95	1.52	0.084	0.072	0.06
0	CC057	F	Y	-1.58	0.82	1.73	0.062	0.035	0.075
0	CC057	F	Y	-1.35	0.89	1.54	0.067	0.055	0.043
0	CC057	M	Y	-1.53	1.14	1.83	0.04	0.068	0.066
0	CC057	M	Y	-1.28	0.91	1.4	0.091	0.037	0.071
0	CC057	M	Y	-1.55	0.86	1.5	0.075	0.034	0.049
1	CC057	F	Y	-1.5	0.84	1.48	0.087	0.017	0.063
1	CC057	F	Y	-1.49	1.38	1.81	0.048	0.041	0.047
1	CC057	F	Y	-1.11	1.39	1.43	0.046	0.023	0.058
1	CC057	F	Y	-1.72	0.97	1.51	0.074	0.03	0.066
1	CC057	M	Y	-1.66	0.85	1.94	0.11	0.013	0.065
1	CC057	M	Y	-1.44	1.02	1.43	0.104	0.068	0.082
2	CC057	F	Y	-1.72	0.97	1.51	0.074	0.03	0.066
0	CC078	F	Y	-1.03	0.56	0.98	0.053	0.014	0.075
0	CC078	F	Y	-0.78	0.63	1.08	0.049	0.053	0.043
0	CC078	F	Y	-1.08	0.59	1.03	0.065	0.031	0.05
0	CC078	M	Y	-1.11	0.64	1.01	0.065	0.034	0.051
1	CC078	F	Y	-1.23	0.42	0.73	0.056	0.052	0.054
1	CC078	F	Y	-0.82	0.46	0.81	0.084	0.021	0.048
1	CC078	M	Y	-0.98	0.55	0.76	0.085	0.042	0.095
2	CC078	F	Y	-1.44	0.65	0.72	0.035	0.06	0.071
2	CC078	M	Y	-1.16	0.63	0.9	0.084	0.039	0.094
2	CC005	M	N	-0.9	0.66	0.86	0.056	0.026	0.092
2	CC005	M	Y	-0.7	0.93	1.21	0.041	0.03	0.037
2	CC015	F	N	-1.05	0.11	1.23	0.045	0.04	0.019
2	CC015	M	N	-1.24	0.9	1.18	0.129	0.091	0.129
2	CC015	M	Y	-1.38	0.91	1.51	0.138	0.109	0.106
2	CC015	M	Y	-0.81	0.72	0.86	0.069	0.09	0.05
2	CC017	F	N	-1.39	0.73	1.49	0.11	0.089	0.044
2	CC017	M	N	-1.42	0.98	1.3	0.032	0.041	0.028
2	CC017	F	Y	-1.17	0.86	1.18	0.133	0.03	0.129
2	CC023	F	N	-1.11	0.71	1.26	0.131	0.077	0.147

Trial#	Strain	Sex	Inf?	AxisDistance_LH	AxisDistance_RF	AxisDistance_RH	Brake_LF	Brake_LH	Brake_RF

Table S3. Raw DigiGait measurement data. Far left column indicates time point at which data was measured: T0 = pre-infection, T1 = 21dpi, and T3 = 89dpi. DigiGait parameters listed across the top indicate which limb is associated with the data, where appropriate: FL for left fore limb, FR for right fore limb, HL for left hind limb, and HR for right hind limb.

Table S3

Trial#	Strain	Sex	Inf?	Brake_RH	GaitSymmetry_LF	GaitSymmetry_LH	GaitSymmetry_RF	GaitSymmetry_RH
1	CC002	F	N	0.04	0.88	0.88	0.88	0.88
1	CC025	F	N	0.055	1.05	1.05	1.05	1.05
1	CC012XCC032	F	N	0.04	1.09	1.09	1.09	1.09
2	CC012XCC032	F	N	0.099	1.11	1.11	1.11	1.11
1	CC012XCC032	M	N	0.012	1.12	1.12	1.12	1.12
2	CC012XCC032	M	N	0.022	0.83	0.83	0.83	0.83
1	CC013xCC041	F	N	0.043	0.77	0.77	0.77	0.77
2	CC013XCC041	F	N	0.086	0.99	0.99	0.99	0.99
1	CC013xCC041	M	N	0.023	0.82	0.82	0.82	0.82
2	CC013XCC041	M	N	0.074	1.16	1.16	1.16	1.16
1	CC032XCC013	F	N	0.038	1.4	1.4	1.4	1.4
2	CC032XCC013	F	N	0.074	1.13	1.13	1.13	1.13
1	CC041XCC012	F	N	0.042	0.79	0.79	0.79	0.79
1	CC041XCC012	F	N	0.038	1.12	1.12	1.12	1.12
2	CC041XCC012	F	N	0.053	1.46	1.46	1.46	1.46
1	CC032XCC013	M	N	0.035	0.99	0.99	0.99	0.99
2	CC032XCC013	M	N	0.027	1.69	1.69	1.69	1.69
1	CC041XCC012	M	N	0.032	1.17	1.17	1.17	1.17
2	CC041XCC012	M	N	0.039	1.14	1.14	1.14	1.14
0	CC012	F	N	0.057	1.22	1.22	1.22	1.22
2	CC012	F	N	0.038	1.13	1.13	1.13	1.13
0	CC012	M	N	0.03	0.9	0.9	0.9	0.9
2	CC012	M	N	0.081	1.87	1.87	1.87	1.87
0	CC057	F	N	0.049	0.88	0.88	0.88	0.88
1	CC057	F	N	0.073	1	1	1	1
0	CC057	M	N	0.06	1.08	1.08	1.08	1.08
1	CC057	M	N	0.08	1	1	1	1
0	CC078	F	N	0.033	0.98	0.98	0.98	0.98
1	CC078	F	N	0.025	1.06	1.06	1.06	1.06
2	CC078	F	N	0.052	1.37	1.37	1.37	1.37
0	CC078	M	N	0.033	1.03	1.03	1.03	1.03
2	CC078	M	N	0.063	1.11	1.11	1.11	1.11
0	CC002	F	N	0.029	0.58	0.58	0.58	0.58
1	CC002	F	N	0.04	0.88	0.88	0.88	0.88
0	CC002	M	N	0.02	0.83	0.83	0.83	0.83
1	CC002	M	N	0.039	1.16	1.16	1.16	1.16
2	CC002	M	N	0.053	0.96	0.96	0.96	0.96
0	CC006	F	N	0.026	0.99	0.99	0.99	0.99
1	CC006	F	N	0.033	1.26	1.26	1.26	1.26
2	CC006	F	N	0.029	0.99	0.99	0.99	0.99
0	CC006	M	N	0.018	0.59	0.59	0.59	0.59
0	CC023	F	N	0.055	0.88	0.88	0.88	0.88
1	CC023	F	N	0.045	1.29	1.29	1.29	1.29
2	CC023	F	N	0.055	1.29	1.29	1.29	1.29
0	CC023	M	N	0.018	0.9	0.9	0.9	0.9
1	CC023	M	N	0.062	1.04	1.04	1.04	1.04
0	CC027	F	N	0.021	1	1	1	1

Trial#	Strain	Sex	Inf?	Brake_RH	GaitSymmetry_LF	GaitSymmetry_LH	GaitSymmetry_RF	GaitSymmetry_RH
1	CC027	F	N	0.085	0.99	0.99	0.99	0.99
2	CC027	F	N	0.031	0.96	0.96	0.96	0.96
0	CC027	M	N	0.049	0.66	0.66	0.66	0.66
1	CC027	M	N	0.036	0.93	0.93	0.93	0.93
1	CC027	M	N	0.063	0.8	0.8	0.8	0.8
2	CC027	M	N	0.061	1	1	1	1
0	CC005	F	N	0.024	1.13	1.13	1.13	1.13
1	CC005	F	N	0.059	1.04	1.04	1.04	1.04
2	CC005	F	N	0.029	0.81	0.81	0.81	0.81
0	CC011	F	N	0.01	1.13	1.13	1.13	1.13
1	CC011	F	N	0.043	1.51	1.51	1.51	1.51
2	CC011	F	N	0.043	1.08	1.08	1.08	1.08
1	CC017	F	N	0.041	1.15	1.15	1.15	1.15
0	CC005	M	N	0.036	0.99	0.99	0.99	0.99
1	CC005	M	N	0.03	0.96	0.96	0.96	0.96
2	CC005	M	N	0.035	1.13	1.13	1.13	1.13
0	CC011	M	N	0.023	1.01	1.01	1.01	1.01
1	CC011	M	N	0.038	1.01	1.01	1.01	1.01
2	CC011	M	N	0.032	1.13	1.13	1.13	1.13
0	CC017	M	N	0.028	0.79	0.79	0.79	0.79
1	CC017	M	N	0.026	1.09	1.09	1.09	1.09
1	CC006	F	N	0.037	0.99	0.99	0.99	0.99
0	CC037	F	N	0.075	1.12	1.12	1.12	1.12
0	CC051	F	N	0.078	1.52	1.52	1.52	1.52
1	CC051	F	N	0.064	1.02	1.02	1.02	1.02
1	CC006	M	N	0.074	1.16	1.16	1.16	1.16
0	CC037	M	N	0.045	1.15	1.15	1.15	1.15
1	CC037	M	N	0.064	0.93	0.93	0.93	0.93
0	CC005	F	N	0.022	1.18	1.18	1.18	1.18
1	CC005	F	N	0.049	1.13	1.13	1.13	1.13
2	CC005	F	N	0.043	1.48	1.48	1.48	1.48
0	CC011	F	N	0.041	0.98	0.98	0.98	0.98
1	CC011	F	N	0.066	1.02	1.02	1.02	1.02
2	CC011	F	N	0.102	0.96	0.96	0.96	0.96
0	CC011	M	N	0.077	0.99	0.99	0.99	0.99
1	CC011	M	N	0.093	0.96	0.96	0.96	0.96
2	CC011	M	N	0.089	0.99	0.99	0.99	0.99
1	CC037	M	N	0.082	1.21	1.21	1.21	1.21
2	CC037	M	N	0.164	1.41	1.41	1.41	1.41
1	CC051	M	N	0.062	0.96	0.96	0.96	0.96
2	CC051	M	N	0.063	1.17	1.17	1.17	1.17
0	CC027	F	N	0.083	0.99	0.99	0.99	0.99
1	CC027	F	N	0.042	0.82	0.82	0.82	0.82
2	CC027	F	N	0.051	1.09	1.09	1.09	1.09
0	CC015	M	N	0.064	1.03	1.03	1.03	1.03
1	CC015	M	N	0.039	1.04	1.04	1.04	1.04
2	CC015	M	N	0.033	1.07	1.07	1.07	1.07
0	CC027	M	N	0.079	1.01	1.01	1.01	1.01

Trial#	Strain	Sex	Inf?	Brake_RH	GaitSymmetry_LF	GaitSymmetry_LH	GaitSymmetry_RF	GaitSymmetry_RH
1	CC027	M	N	0.061	0.92	0.92	0.92	0.92
2	CC027	M	N	0.03	0.79	0.79	0.79	0.79
0	CC015	F	N	1	1	1	1	0.159
1	CC015	F	N	0.029	1	1	1	1
0	CC017	F	N	0.062	1.11	1.11	1.11	1.11
0	CC023	F	N	0.097	0.95	0.95	0.95	0.95
1	CC023	F	N	0.065	1.05	1.05	1.05	1.05
0	CC005	M	N	0.061	0.87	0.87	0.87	0.87
1	CC005	M	N	0.047	0.98	0.98	0.98	0.98
0	CC015	M	N	0.092	0.98	0.98	0.98	0.98
1	CC015	M	N	0.024	0.95	0.95	0.95	0.95
0	CC017	M	N	0.089	1.01	1.01	1.01	1.01
1	CC017	M	N	0.043	1.01	1.01	1.01	1.01
0	CC023	M	N	0.087	0.98	0.98	0.98	0.98
1	CC023	M	N	0.04	1.06	1.06	1.06	1.06
0	CC051	M	N	0.081	1.04	1.04	1.04	1.04
0	CC002	F	Y	0.047	1	1	1	1
0	CC002	M	Y	0.075	1.54	1.54	1.54	1.54
1	CC002	M	Y	0.014	0.61	0.61	0.61	0.61
1	CC002	F	Y	0.028	0.98	0.98	0.98	0.98
1	CC002	F	Y	0.032	1.29	1.29	1.29	1.29
1	CC002	M	Y	0.014	0.61	0.61	0.61	0.61
1	CC002	M	Y	0.032	0.82	0.82	0.82	0.82
1	CC002	F	Y	0.028	0.98	0.98	0.98	0.98
1	CC002	F	Y	0.016	0.61	0.61	0.61	0.61
2	CC002	M	Y	0.037	1.16	1.16	1.16	1.16
2	CC002	F	Y	0.086	1.06	1.06	1.06	1.06
2	CC002	M	Y	0.072	1.38	1.38	1.38	1.38
0	CC005	F	Y	0.032	1.19	1.19	1.19	1.19
0	CC005	M	Y	0.04	1.24	1.24	1.24	1.24
0	CC005	M	Y	0.063	1.09	1.09	1.09	1.09
1	CC005	F	Y	0.034	1.64	1.64	1.64	1.64
1	CC005	M	Y	0.013	0.71	0.71	0.71	0.71
1	CC005	F	Y	0.028	0.98	0.98	0.98	0.98
1	CC005	M	Y	0.033	0.81	0.81	0.81	0.81
2	CC005	F	Y	0.034	1.07	1.07	1.07	1.07
2	CC005	M	Y	0.051	1.23	1.23	1.23	1.23
2	CC005	M	Y	0.035	0.95	0.95	0.95	0.95
2	CC005	M	Y	0.051	1.23	1.23	1.23	1.23
2	CC005	F	Y	0.046	0.91	0.91	0.91	0.91
0	CC006	F	Y	0.043	1.25	1.25	1.25	1.25
0	CC006	M	Y	0.011	0.83	0.83	0.83	0.83
0	CC006	F	Y	0.044	1.08	1.08	1.08	1.08
1	CC006	F	Y	0.049	1.02	1.02	1.02	1.02
1	CC006	M	Y	0.038	0.88	0.88	0.88	0.88
1	CC006	F	Y	0.023	0.98	0.98	0.98	0.98
1	CC006	M	Y	0.064	1.1	1.1	1.1	1.1
2	CC006	F	Y	0.017	0.96	0.96	0.96	0.96

Trial#	Strain	Sex	Inf?	Brake_RH	GaitSymmetry_LF	GaitSymmetry_LH	GaitSymmetry_RF	GaitSymmetry_RH
2	CC006	M	Y	0.025	0.87	0.87	0.87	0.87
0	CC011	F	Y	0.007	1.65	1.65	1.65	1.65
0	CC011	M	Y	0.032	0.98	0.98	0.98	0.98
0	CC011	F	Y	0.024	1.06	1.06	1.06	1.06
0	CC011	F	Y	0.021	0.73	0.73	0.73	0.73
1	CC011	F	Y	0.055	1.12	1.12	1.12	1.12
1	CC011	M	Y	0.063	1.02	1.02	1.02	1.02
1	CC011	F	Y	0.055	0.99	0.99	0.99	0.99
1	CC011	M	Y	0.068	1.08	1.08	1.08	1.08
2	CC011	F	Y	0.043	1.03	1.03	1.03	1.03
2	CC011	M	Y	0.052	1.65	1.65	1.65	1.65
2	CC011	F	Y	0.049	0.95	0.95	0.95	0.95
2	CC011	M	Y	0.092	1.01	1.01	1.01	1.01
0	CC012	M	Y	0.042	0.91	0.91	0.91	0.91
0	CC012	M	Y	0.04	0.91	0.91	0.91	0.91
0	CC012	M	Y	0.041	0.98	0.98	0.98	0.98
0	CC012	M	Y	0.042	1.02	1.02	1.02	1.02
0	CC012	M	Y	0.017	0.93	0.93	0.93	0.93
0	CC012	F	Y	0.054	1.34	1.34	1.34	1.34
0	CC012	F	Y	0.028	1.02	1.02	1.02	1.02
0	CC012	F	Y	0.056	1.09	1.09	1.09	1.09
0	CC012	F	Y	0.022	1.06	1.06	1.06	1.06
0	CC012	F	Y	0.013	1.1	1.1	1.1	1.1
2	CC012	M	Y	0.041	1.07	1.07	1.07	1.07
2	CC012	M	Y	0.036	0.99	0.99	0.99	0.99
2	CC012	M	Y	0.062	1.02	1.02	1.02	1.02
2	CC012	M	Y	0.102	0.96	0.96	0.96	0.96
2	CC012	M	Y	0.046	1.08	1.08	1.08	1.08
2	CC012	F	Y	0.084	1.07	1.07	1.07	1.07
2	CC012	F	Y	0.069	1.03	1.03	1.03	1.03
2	CC012	F	Y	0.051	1.21	1.21	1.21	1.21
2	CC012	F	Y	0.073	1.47	1.47	1.47	1.47
2	CC012	F	Y	0.109	1.24	1.24	1.24	1.24
1	CC012XCC032	F	Y	0.03	0.78	0.78	0.78	0.78
1	CC012xCC032	F	Y	0.026	0.88	0.88	0.88	0.88
1	CC012XCC032	M	Y	0.052	1.24	1.24	1.24	1.24
1	CC012xCC032	M	Y	0.04	1.13	1.13	1.13	1.13
1	CC012XCC032	M	Y	0.019	1.09	1.09	1.09	1.09
1	CC012xCC032	M	Y	0.027	0.94	0.94	0.94	0.94
2	CC012XCC032	F	Y	0.116	1.49	1.49	1.49	1.49
2	CC012XCC032	F	Y	0.166	0.98	0.98	0.98	0.98
2	CC012XCC032	M	Y	0.065	1.52	1.52	1.52	1.52
2	CC012XCC032	M	Y	0.081	1.59	1.59	1.59	1.59
2	CC012XCC032	M	Y	0.081	0.99	0.99	0.99	0.99
2	CC012XCC032	M	Y	0.141	1.25	1.25	1.25	1.25
1	CC013xCC041	F	Y	0.032	0.79	0.79	0.79	0.79
1	CC013xCC041	F	Y	0.023	0.85	0.85	0.85	0.85
1	CC013xCC041	F	Y	0.029	1.16	1.16	1.16	1.16

Trial#	Strain	Sex	Inf?	Brake_RH	GaitSymmetry_LF	GaitSymmetry_LH	GaitSymmetry_RF	GaitSymmetry_RH
1	CC013xCC041	M	Y	0.066	1.18	1.18	1.18	1.18
1	CC013xCC041	M	Y	0.033	0.9	0.9	0.9	0.9
2	CC013XCC041	F	Y	0.031	0.99	0.99	0.99	0.99
2	CC013XCC041	F	Y	0.12	1.3	1.3	1.3	1.3
2	CC013XCC041	F	Y	0.038	1.07	1.07	1.07	1.07
2	CC013XCC041	M	Y	0.124	1.31	1.31	1.31	1.31
2	CC013XCC041	M	Y	0.025	1.1	1.1	1.1	1.1
0	CC015	F	Y	0.103	0.95	0.95	0.95	0.95
0	CC015	M	Y	0.069	0.98	0.98	0.98	0.98
0	CC015	M	Y	0.079	0.94	0.94	0.94	0.94
1	CC015	F	Y	0.071	0.93	0.93	0.93	0.93
1	CC015	M	Y	0.03	0.9	0.9	0.9	0.9
1	CC015	M	Y	0.041	0.72	0.72	0.72	0.72
1	CC015	M	Y	0.05	1.21	1.21	1.21	1.21
2	CC015	F	Y	0.094	1.06	1.06	1.06	1.06
0	CC017	F	Y	0.024	0.97	0.97	0.97	0.97
0	CC017	M	Y	0.036	0.73	0.73	0.73	0.73
0	CC017	F	Y	0.089	1.33	1.33	1.33	1.33
0	CC017	M	Y	0.047	1.48	1.48	1.48	1.48
1	CC017	M	Y	0.047	1.17	1.17	1.17	1.17
1	CC017	F	Y	0.03	1.01	1.01	1.01	1.01
0	CC023	M	Y	0.025	1.02	1.02	1.02	1.02
0	CC023	F	Y	0.04	0.79	0.79	0.79	0.79
0	CC023	F	Y	0.025	1.01	1.01	1.01	1.01
0	CC023	M	Y	0.055	1.98	1.98	1.98	1.98
1	CC023	M	Y	0.031	1.67	1.67	1.67	1.67
1	CC023	F	Y	0.063	1.56	1.56	1.56	1.56
1	CC023	M	Y	0.054	1.51	1.51	1.51	1.51
1	CC023	M	Y	0.08	1.02	1.02	1.02	1.02
2	CC023	M	Y	0.055	0.94	0.94	0.94	0.94
2	CC023	F	Y	0.13	1.32	1.32	1.32	1.32
2	CC023	M	Y	0.032	1.6	1.6	1.6	1.6
0	CC025	M	Y	0.05	1.01	1.01	1.01	1.01
0	CC025	F	Y	0.057	1.08	1.08	1.08	1.08
0	CC025	M	Y	0.035	1.1	1.1	1.1	1.1
1	CC025	M	Y	0.052	1.06	1.06	1.06	1.06
1	CC025	F	Y	0.036	0.98	0.98	0.98	0.98
1	CC025	F	Y	0.054	1.01	1.01	1.01	1.01
1	CC025	F	Y	0.048	1.09	1.09	1.09	1.09
2	CC025	F	Y	0.061	1.03	1.03	1.03	1.03
2	CC025	M	Y	0.039	1.02	1.02	1.02	1.02
0	CC027	F	Y	0.029	1.04	1.04	1.04	1.04
0	CC027	M	Y	0.03	1.88	1.88	1.88	1.88
1	CC027	M	Y	0.04	0.88	0.88	0.88	0.88
1	CC027	M	Y	0.04	0.88	0.88	0.88	0.88
1	CC027	F	Y	0.021	0.88	0.88	0.88	0.88
1	CC027	F	Y	0.026	0.95	0.95	0.95	0.95
1	CC027	M	Y	0.069	1.13	1.13	1.13	1.13

Trial#	Strain	Sex	Inf?	Brake_RH	GaitSymmetry_LF	GaitSymmetry_LH	GaitSymmetry_RF	GaitSymmetry_RH
1	CC027	M	Y	0.063	0.98	0.98	0.98	0.98
2	CC027	F	Y	0.13	1.32	1.32	1.32	1.32
2	CC027	M	Y	0.069	0.9	0.9	0.9	0.9
2	CC027	F	Y	0.056	1.3	1.3	1.3	1.3
2	CC027	M	Y	0.085	1.08	1.08	1.08	1.08
1	CC032XCC013	M	Y	0.033	1.15	1.15	1.15	1.15
1	CC032XCC013	M	Y	0.043	1.07	1.07	1.07	1.07
1	CC032XCC013	M	Y	0.029	1.24	1.24	1.24	1.24
1	CC032XCC013	F	Y	0.031	1.04	1.04	1.04	1.04
1	CC032XCC013	F	Y	0.053	1.32	1.32	1.32	1.32
1	CC032XCC013	F	Y	0.068	1.17	1.17	1.17	1.17
1	CC032XCC013	F	Y	0.07	1.03	1.03	1.03	1.03
1	CC032XCC013	F	Y	0.053	0.87	0.87	0.87	0.87
2	CC032XCC013	M	Y	0.103	1.08	1.08	1.08	1.08
2	CC032XCC013	M	Y	0.048	1.14	1.14	1.14	1.14
2	CC032XCC013	M	Y	0.037	1.67	1.67	1.67	1.67
2	CC032XCC013	M	Y	0.063	1.39	1.39	1.39	1.39
2	CC032XCC013	F	Y	0.103	1.03	1.03	1.03	1.03
2	CC032XCC013	F	Y	0.089	1.23	1.23	1.23	1.23
2	CC032XCC013	F	Y	0.09	1.29	1.29	1.29	1.29
2	CC032XCC013	F	Y	0.066	1.15	1.15	1.15	1.15
0	CC037	F	Y	0.053	1.39	1.39	1.39	1.39
0	CC037	M	Y	0.059	1.58	1.58	1.58	1.58
0	CC037	M	Y	0.025	1.87	1.87	1.87	1.87
0	CC037	M	Y	0.029	1.03	1.03	1.03	1.03
1	CC037	F	Y	0.05	1.15	1.15	1.15	1.15
1	CC037	M	Y	0.072	1.03	1.03	1.03	1.03
1	CC037	M	Y	0.063	1.4	1.4	1.4	1.4
1	CC037	M	Y	0.053	0.92	0.92	0.92	0.92
2	CC037	M	Y	0.136	1.41	1.41	1.41	1.41
2	CC037	M	Y	0.104	1	1	1	1
1	CC041XCC012	M	Y	0.024	1.24	1.12	1.12	1.12
1	CC041XCC012	M	Y	0.055	1.12	1.06	1.06	1.06
1	CC041XCC012	M	Y	0.024	1.06	0.97	0.97	0.97
1	CC041XCC012	M	Y	0.025	0.97	0.79	0.79	0.79
1	CC041XCC012	M	Y	0.033	0.79	1.09	1.09	1.09
1	CC041XCC012	F	Y	0.087	1.35	1.35	1.35	1.35
1	CC041XCC012	F	Y	0.044	1.21	1.21	1.21	1.21
1	CC041XCC012	F	Y	0.051	1.09	1.41	1.41	1.41
1	CC041XCC012	F	Y	0.041	1.41	1.13	1.13	1.13
1	CC041XCC012	F	Y	0.036	1.13	1.14	1.14	1.14
1	CC041XCC012	F	Y	0.062	1.14	1.07	1.07	1.07
1	CC041XCC012	F	Y	0.086	1.07	1.01	1.01	1.01
1	CC041XCC012	F	Y	0.048	1.01	1.24	1.24	1.24
2	CC041XCC012	M	Y	0.073	0.94	0.94	0.94	0.94
2	CC041XCC012	M	Y	0.037	1.15	1.15	1.15	1.15
2	CC041XCC012	F	Y	0.034	1.06	1.06	1.06	1.06
2	CC041XCC012	M	Y	0.061	1.16	1.16	1.16	1.16

Trial#	Strain	Sex	Inf?	Brake_RH	GaitSymmetry_LF	GaitSymmetry_LH	GaitSymmetry_RF	GaitSymmetry_RH
2	CC041XCC012	M	Y	0.059	0.98	0.98	0.98	0.98
2	CC041XCC012	M	Y	0.039	1.11	1.11	1.11	1.11
2	CC041XCC012	F	Y	0.075	0.96	0.96	0.96	0.96
2	CC041XCC012	F	Y	0.071	1.03	1.03	1.03	1.03
2	CC041XCC012	F	Y	0.102	1.02	1.02	1.02	1.02
2	CC041XCC012	F	Y	0.074	0.97	0.97	0.97	0.97
2	CC041XCC012	F	Y	0.107	1.16	1.16	1.16	1.16
2	CC041XCC012	F	Y	0.091	0.99	0.99	0.99	0.99
2	CC041XCC012	F	Y	0.04	1.07	1.07	1.07	1.07
0	CC051	F	Y	0.072	1.13	1.13	1.13	1.13
0	CC051	F	Y	0.082	1.34	1.34	1.34	1.34
0	CC051	F	Y	0.07	1.04	1.04	1.04	1.04
0	CC051	M	Y	0.083	1.06	1.06	1.06	1.06
1	CC051	F	Y	0.088	1.07	1.07	1.07	1.07
1	CC051	F	Y	0.092	1.11	1.11	1.11	1.11
1	CC051	F	Y	0.058	1.11	1.11	1.11	1.11
0	CC057	F	Y	0.042	1.01	1.01	1.01	1.01
0	CC057	F	Y	0.037	1.01	1.01	1.01	1.01
0	CC057	F	Y	0.034	0.94	0.94	0.94	0.94
0	CC057	M	Y	0.05	1.19	1.19	1.19	1.19
0	CC057	M	Y	0.02	0.53	0.53	0.53	0.53
0	CC057	M	Y	0.031	1.03	1.03	1.03	1.03
1	CC057	F	Y	0.042	1.01	1.01	1.01	1.01
1	CC057	F	Y	0.024	1.04	1.04	1.04	1.04
1	CC057	F	Y	0.029	1.07	1.07	1.07	1.07
1	CC057	F	Y	0.034	1.01	1.01	1.01	1.01
1	CC057	M	Y	0.063	1.19	1.19	1.19	1.19
1	CC057	M	Y	0.08	1.03	1.03	1.03	1.03
2	CC057	F	Y	0.034	1.01	1.01	1.01	1.01
0	CC078	F	Y	0.038	0.95	0.95	0.95	0.95
0	CC078	F	Y	0.032	0.86	0.86	0.86	0.86
0	CC078	F	Y	0.029	1	1	1	1
0	CC078	M	Y	0.067	1.01	1.01	1.01	1.01
1	CC078	F	Y	0.044	1.05	1.05	1.05	1.05
1	CC078	F	Y	0.017	1.05	1.05	1.05	1.05
1	CC078	M	Y	0.074	1.04	1.04	1.04	1.04
2	CC078	F	Y	0.044	1.02	1.02	1.02	1.02
2	CC078	M	Y	0.085	1.18	1.18	1.18	1.18
2	CC005	M	N	0.024	0.86	0.86	0.86	0.86
2	CC005	M	Y	0.074	1.31	1.31	1.31	1.31
2	CC015	F	N	0.042	1.42	1.42	1.42	1.42
2	CC015	M	N	0.11	1.07	1.07	1.07	1.07
2	CC015	M	Y	0.094	1.06	1.06	1.06	1.06
2	CC015	M	Y	0.062	1.17	1.17	1.17	1.17
2	CC017	F	N	0.058	1.48	1.48	1.48	1.48
2	CC017	M	N	0.047	1.6	1.6	1.6	1.6
2	CC017	F	Y	0.024	1.02	1.02	1.02	1.02
2	CC023	F	N	0.055	1.02	1.02	1.02	1.02

Trial#	Strain	Sex	Inf?	Brake_RH	GaitSymmetry_LF	GaitSymmetry_LH	GaitSymmetry_RF	GaitSymmetry_RH

Table S3. Raw DigiGait measurement data. Far left column indicates time point at which data was measured: T0 = pre-infection, T1 = 21dpi, and T3 = 89dpi. DigiGait parameters listed across the top indicate which limb is associated with the data, where appropriate: FL for left fore limb, FR for right fore limb, HL for left hind limb, and HR for right hind limb.

Table S3

Trial#	Strain	Sex	Inf?	HindLimbSharedStanceTime_LH	MAXdA/dT_LF	MAXdA/dT_LH	MAXdA/dT_RF	MAXdA/dT_RH
1	CC002	F	N	0.089	14.2	9.53	15.37	20.41
1	CC025	F	N	0.139	13.17	49.22	12.38	43.21
1	CC012XCC032	F	N	0.149	17.15	25.96	17.5	26.28
2	CC012XCC032	F	N	0.156	27.65	58.71	27.54	54.51
1	CC012XCC032	M	N	0.266	21.3	49.15	20.73	32.1
2	CC012XCC032	M	N	0.215	11.53	57.5	16.19	63.89
1	CC013xCC041	F	N	0.071	40.49	33.52	35.05	51.77
2	CC013XCC041	F	N	0.195	27.17	51.15	19.5	58.73
1	CC013xCC041	M	N	0.069	8.33	15.38	5.53	4.18
2	CC013XCC041	M	N	0.145	27.19	59.26	28.75	70.13
1	CC032XCC013	F	N	0.093	12.83	27.81	12.32	19.26
2	CC032XCC013	F	N	0.193	30.28	29.23	23.11	39.16
1	CC041XCC012	F	N	0.077	31.36	42.82	31.4	40.99
1	CC041XCC012	F	N	0.065	15.83	16.34	14.63	26.96
2	CC041XCC012	F	N	0.164	13.28	49.62	16.78	43.55
1	CC032XCC013	M	N	0.054	33.24	44.92	23.5	34.64
2	CC032XCC013	M	N	0.093	20.72	42.16	15.69	26.07
1	CC041XCC012	M	N	0.125	21.03	25.86	14.76	19.66
2	CC041XCC012	M	N	0.194	23.07	48.61	25.17	55.15
0	CC012	F	N	0.098	16	31.68	20.92	33.4
2	CC012	F	N	0.145	18.12	52.79	34.03	62.67
0	CC012	M	N	0.101	11.37	31.73	17.1	39.62
2	CC012	M	N	0.231	23.63	34.8	29.14	39.15
0	CC057	F	N	0.156	28.68	31.99	22.95	25.96
1	CC057	F	N	0.138	36.36	77.39	33.7	53.31
0	CC057	M	N	0.13	26.13	19.7	30.85	46.61
1	CC057	M	N	0.168	36.58	65.72	43.2	69.12
0	CC078	F	N	0.128	35.67	38.22	29.19	50.96
1	CC078	F	N	0.127	20.13	68.48	24.89	81.59
2	CC078	F	N	0.097	20.85	46.44	17.8	22.36
0	CC078	M	N	0.066	28.6	39.67	19.79	38.5
2	CC078	M	N	0.103	24.55	38.8	16.84	45.41
0	CC002	F	N	0.052	32.5	27.54	18.74	16.8
1	CC002	F	N	0.089	14.2	9.53	15.37	20.41
0	CC002	M	N	0.065	24.33	30.51	17.07	17.09
1	CC002	M	N	0.118	32.8	39.87	24.08	24.24
2	CC002	M	N	0.264	24.59	65.42	25.53	49.52
0	CC006	F	N	0.105	27.94	100.8	30.31	67.66
1	CC006	F	N	0.18	25.41	53.58	26.91	30.19
2	CC006	F	N	0.084	13.5	18.79	12.87	32.74
0	CC006	M	N	0.056	24.5	21.52	21.65	18.97
0	CC023	F	N	0.119	13.03	23.92	13.18	18.13
1	CC023	F	N	0.088	29.68	44.31	24.84	41.22
2	CC023	F	N	0.131	36.91	70.22	41.39	75.72
0	CC023	M	N	0.124	19.33	43.24	11.18	25.07
1	CC023	M	N	0.128	23.96	54.29	21.03	49.46
0	CC027	F	N	0.01	15.42	5.41	10.48	7.43

Trial#	Strain	Sex	Inf?	HindLimbSharedStanceTime_LH	MAXdA/dT_LF	MAXdA/dT_LH	MAXdA/dT_RF	MAXdA/dT_RH
1	CC027	F	N	0.134	23.72	20.32	24.65	15.42
2	CC027	F	N	0.161	36.56	60.3	24.77	40.6
0	CC027	M	N	0.017	15.71	6.19	18.42	7.08
1	CC027	M	N	0.099	28.9	33.57	29.02	27.01
1	CC027	M	N	0.13	26.88	16.61	17.17	15.75
2	CC027	M	N	0.086	42.8	77.33	23.17	69.71
0	CC005	F	N	0.136	15.8	57.48	14.91	47.11
1	CC005	F	N	0.112	9.08	16.31	7.64	16.93
2	CC005	F	N	0.028	6.83	4.83	7.42	5.69
0	CC011	F	N	0.097	30.08	28.95	38.47	71.54
1	CC011	F	N	0.186	67.58	103.05	58.37	86.78
2	CC011	F	N	0.172	38.74	86.14	39.52	92.89
1	CC017	F	N	0.138	29.15	40.98	24.53	35.37
0	CC005	M	N	0.145	24.24	74.06	24.1	64.57
1	CC005	M	N	0.133	12.96	21.51	18.29	28.42
2	CC005	M	N	0.132	12.3	20.86	12.32	25.16
0	CC011	M	N	0.121	19.35	59.04	21.05	66.8
1	CC011	M	N	0.138	23.19	63.62	21.23	63.83
2	CC011	M	N	0.138	36.25	69.14	27.13	58.96
0	CC017	M	N	0.019	7.63	3.74	5.91	4.84
1	CC017	M	N	0.131	21.28	51.06	19.87	37.52
1	CC006	F	N	0.015	14.14	10.93	11.74	18.46
0	CC037	F	N	0.109	16.25	35.03	11.66	27.05
0	CC051	F	N	0.152	16.07	33.58	17.59	36.91
1	CC051	F	N	0.135	12.55	21.14	10.55	26.2
1	CC006	M	N	0.123	33.37	56.63	22.33	41.83
0	CC037	M	N	0.118	8.92	34.52	12.54	32
1	CC037	M	N	0.133	19.28	42.49	24.25	42.25
0	CC005	F	N	0.12	18.98	29.33	17.51	29.99
1	CC005	F	N	0.199	27.37	51.02	21.77	59.58
2	CC005	F	N	0.06	15.44	12.85	17.97	17.61
0	CC011	F	N	0.125	21.7	51.29	23.72	33.81
1	CC011	F	N	0.143	23.67	109.08	18.74	88.5
2	CC011	F	N	0.162	13.83	55.48	12.3	47.72
0	CC011	M	N	0.121	26.65	65.46	21.8	75.39
1	CC011	M	N	0.133	26.16	75.4	25.16	68.36
2	CC011	M	N	0.129	14.34	40.11	13.21	34.85
1	CC037	M	N	0.169	40.2	69.04	37.77	60.48
2	CC037	M	N	0.212	12.54	35.38	12.97	21.06
1	CC051	M	N	0.172	14.76	58.64	16.66	67.82
2	CC051	M	N	0.14	13.7	45.34	16.8	48.04
0	CC027	F	N	0.188	27.24	40.8	21.05	25.22
1	CC027	F	N	0.074	15.88	26.66	13.89	26.37
2	CC027	F	N	0.091	22.21	28.86	16.61	21.18
0	CC015	M	N	0.095	18.7	22.77	16.47	23.67
1	CC015	M	N	0.073	12.54	38.9	13.41	30.69
2	CC015	M	N	0.111	10.58	35.56	9.1	34.09
0	CC027	M	N	0.137	15.28	24.25	16.38	17.75

Trial#	Strain	Sex	Inf?	HindLimbSharedStanceTime_LH	MAXdA/dT_LF	MAXdA/dT_LH	MAXdA/dT_RF	MAXdA/dT_RH
1	CC027	M	N	0.138	22.37	82.99	23.76	71.19
2	CC027	M	N	0.09	20.54	35.57	13.66	34.29
0	CC015	F	N	18.29	21.31	17.27	22.77	-0.78
1	CC015	F	N	0.052	13.76	20.45	14.12	22.87
0	CC017	F	N	0.09	21.98	33.36	19.09	27.08
0	CC023	F	N	0.114	15.24	25.38	17.59	26.24
1	CC023	F	N	0.119	22.23	45.42	24.13	43.09
0	CC005	M	N	0.081	29.73	38.91	20.66	13.04
1	CC005	M	N	0.092	21.91	49.86	17.55	30.5
0	CC015	M	N	0.155	30.34	51.63	18.6	30.57
1	CC015	M	N	0.116	21.85	42.74	19.48	25.46
0	CC017	M	N	0.152	29.89	28.71	24.72	29.4
1	CC017	M	N	0.075	18.48	23.66	13.97	26.96
0	CC023	M	N	0.14	31.25	44.99	34.53	32.61
1	CC023	M	N	0.163	25.17	46.78	31.06	57.84
0	CC051	M	N	0.167	24.02	63.89	28.35	48.8
0	CC002	F	Y	0.081	27.61	24.75	27.53	23.84
0	CC002	M	Y	0.185	21.02	31.27	17.77	21.27
1	CC002	M	Y	0.004	11.48	10.03	10.87	6.69
1	CC002	F	Y	0.056	12.08	9.14	13.03	11.91
1	CC002	F	Y	0.086	23.64	32.64	21.75	30.8
1	CC002	M	Y	0.004	11.48	10.03	10.87	6.69
1	CC002	M	Y	0.073	21.39	17.87	13.36	9.54
1	CC002	F	Y	0.056	12.08	9.14	13.03	11.91
1	CC002	F	Y	0.019	21.54	11.1	18.99	12.54
2	CC002	M	Y	0.102	31.31	32.55	30.66	31.08
2	CC002	F	Y	0.16	20.05	47.25	17.43	46.85
2	CC002	M	Y	0.174	13.76	44.03	12.28	22.79
0	CC005	F	Y	0.134	13.78	50.61	16.51	59.42
0	CC005	M	Y	0.139	19.85	59	19.72	46.35
0	CC005	M	Y	0.135	33.96	70.08	32.94	54.65
1	CC005	F	Y	0.124	17.27	35.44	13.79	27.33
1	CC005	M	Y	0.028	24.05	12.65	16.22	10.02
1	CC005	F	Y	0.051	34.39	81.23	26.84	86.97
1	CC005	M	Y	0.12	17.99	36.79	22.72	52.62
2	CC005	F	Y	0.123	23.11	32.56	25.08	32.21
2	CC005	M	Y	0.157	36.19	55.82	20.98	63.08
2	CC005	M	Y	0.088	38.58	63.19	23.74	27.5
2	CC005	M	Y	0.157	36.19	55.82	20.98	63.08
2	CC005	F	Y	0.127	19.75	34.21	17.09	22.36
0	CC006	F	Y	0.102	17.86	30.26	15.06	27.75
0	CC006	M	Y	0.022	11.63	6.82	4.96	4.43
0	CC006	F	Y	0.106	21.98	31.08	20.11	29.25
1	CC006	F	Y	0.087	14.17	15.86	9.75	13.91
1	CC006	M	Y	0.055	15.9	11.78	14.89	12.08
1	CC006	F	Y	0.131	33.49	78.92	33.08	57.92
1	CC006	M	Y	0.169	11.57	44.3	17.82	45.93
2	CC006	F	Y	0.083	23.64	40.45	16.19	36.83

Trial#	Strain	Sex	Inf?	HindLimbSharedStanceTime_LH	MAXdA/dT_LF	MAXdA/dT_LH	MAXdA/dT_RF	MAXdA/dT_RH
2	CC006	M	Y	0.052	24.37	31.19	23.04	37.79
0	CC011	F	Y	0.156	57.28	85.75	45.05	86.34
0	CC011	M	Y	0.105	21.24	54.62	16.95	45.78
0	CC011	F	Y	0.083	10.03	28.47	11.02	32.69
0	CC011	F	Y	0.013	22.2	34.97	24.4	45.43
1	CC011	F	Y	0.128	19.73	53.62	13.4	58.38
1	CC011	M	Y	0.169	15.8	50.75	21.62	53.2
1	CC011	F	Y	0.109	25.73	80.93	21.14	65.26
1	CC011	M	Y	0.131	18.21	86.83	19.57	77.76
2	CC011	F	Y	0.14	37.7	88.14	30.5	87.91
2	CC011	M	Y	0.221	33.44	62.74	30.71	74.77
2	CC011	F	Y	0.159	15.11	53.13	14.05	56.48
2	CC011	M	Y	0.135	20.86	54.95	14.63	58.87
0	CC012	M	Y	0.138	32.75	69.18	34.29	57.63
0	CC012	M	Y	0.088	28.38	55.8	25.63	43.56
0	CC012	M	Y	0.116	21.04	45.78	21.86	46.16
0	CC012	M	Y	0.131	20.33	36.81	20.21	45.88
0	CC012	M	Y	0.152	25.1	59.26	28.23	57.34
0	CC012	F	Y	0.146	24.15	51.16	16.13	51.81
0	CC012	F	Y	0.133	27.55	62.23	17.73	56.76
0	CC012	F	Y	0.08	20.78	29.61	13.85	33.89
0	CC012	F	Y	0.06	40.31	64.25	46.75	65.68
0	CC012	F	Y	0.021	33.48	74.64	23.22	44.83
2	CC012	M	Y	0.141	43.12	103.07	41.62	79.87
2	CC012	M	Y	0.12	30.47	67.6	35.5	63.72
2	CC012	M	Y	0.14	47.14	75.06	52.42	77.63
2	CC012	M	Y	0.217	22.45	55.64	20.39	63.25
2	CC012	M	Y	0.129	29	82.42	32.62	72.61
2	CC012	F	Y	0.239	30.63	62.57	27.63	68.19
2	CC012	F	Y	0.136	27.7	48.34	25.48	45.95
2	CC012	F	Y	0.162	29.32	43.73	22.35	25.21
2	CC012	F	Y	0.172	25.95	75.51	40.64	57.87
2	CC012	F	Y	0.146	29.68	38.35	27.84	38.15
1	CC012XCC032	F	Y	0.043	23.93	40.95	30.69	41.99
1	CC012xCC032	F	Y	0.026	14.48	9.51	14.28	8.44
1	CC012XCC032	M	Y	0.138	17.35	39.54	18.43	33.43
1	CC012xCC032	M	Y	0.147	18.52	32.52	17.38	29.28
1	CC012XCC032	M	Y	0.081	13.84	27.13	21.51	27.46
1	CC012xCC032	M	Y	0.08	14.36	15.16	10.07	16.31
2	CC012XCC032	F	Y	0.156	21.99	48.46	28.03	56.12
2	CC012XCC032	F	Y	0.167	22	52.6	23.65	43.64
2	CC012XCC032	M	Y	0.207	24.65	71.51	18.3	43.99
2	CC012XCC032	M	Y	0.116	20.24	49.68	18.23	55.05
2	CC012XCC032	M	Y	0.186	19.65	80.48	24.66	68.44
2	CC012XCC032	M	Y	0.176	27.59	90.93	26.32	41.67
1	CC013xCC041	F	Y	0.076	34.23	45.79	29.17	45.67
1	CC013xCC041	F	Y	0.031	23.92	26.11	24.57	19.87
1	CC013xCC041	F	Y	0.093	17.79	16.22	17.55	18.79

Trial#	Strain	Sex	Inf?	HindLimbSharedStanceTime_LH	MAXdA/dT_LF	MAXdA/dT_LH	MAXdA/dT_RF	MAXdA/dT_RH
1	CC013xCC041	M	Y	0.112	30.48	47.85	28.28	37.2
1	CC013xCC041	M	Y	0.163	23.6	40.66	35.15	28.06
2	CC013XCC041	F	Y	0.158	23.11	65.6	34.47	88.05
2	CC013XCC041	F	Y	0.232	32.75	68.61	54.39	91.53
2	CC013XCC041	F	Y	0.177	40.97	98.96	33.32	107.03
2	CC013XCC041	M	Y	0.181	24.77	97.61	25.53	68.95
2	CC013XCC041	M	Y	0.102	19.12	29.69	19.28	15.04
0	CC015	F	Y	0.1	17.88	15.42	21.96	21.98
0	CC015	M	Y	0.156	22.51	26.26	26.04	36.94
0	CC015	M	Y	0.132	21.12	33.36	17.79	36.03
1	CC015	F	Y	0.098	15.76	27.61	13.17	20.18
1	CC015	M	Y	0.054	17.74	22.49	16.89	26.9
1	CC015	M	Y	0.054	18.77	28.54	23.9	43.9
1	CC015	M	Y	0.13	17.92	28.91	18.62	29.79
2	CC015	F	Y	0.133	17.32	26.16	10.18	27.59
0	CC017	F	Y	0.053	16.78	35.53	18.79	28.15
0	CC017	M	Y	0.095	10.88	40.47	15.31	43.7
0	CC017	F	Y	0.173	31.41	62.72	25.89	43.99
0	CC017	M	Y	0.124	24.24	37.2	22.49	34.97
1	CC017	M	Y	0.116	28	46.64	17.92	32.67
1	CC017	F	Y	0.071	15.75	22.78	17.23	28.67
0	CC023	M	Y	0.11	33.27	49.38	26.93	36.13
0	CC023	F	Y	0.099	14.85	23.49	10.23	7.62
0	CC023	F	Y	0.038	14.9	20.01	20.38	24.42
0	CC023	M	Y	0.126	18.6	34.86	22.23	35.15
1	CC023	M	Y	0.146	15.83	25.54	16.12	22.15
1	CC023	F	Y	0.152	25.93	18.28	18.41	22.04
1	CC023	M	Y	0.173	20.02	29.63	10.29	15.92
1	CC023	M	Y	0.124	18.84	28.52	8.29	20.46
2	CC023	M	Y	0.129	29.31	46.47	29.48	46.72
2	CC023	F	Y	0.189	21.52	33.16	31.5	37.2
2	CC023	M	Y	0.194	10	21.26	10.6	11.89
0	CC025	M	Y	0.106	19.86	50.43	17.53	34.45
0	CC025	F	Y	0.131	21.73	31.65	21.45	36.54
0	CC025	M	Y	0.155	14.56	36.56	11.53	28.27
1	CC025	M	Y	0.107	19.72	50.78	17.16	34.13
1	CC025	F	Y	0.084	17.9	63.95	18.31	44.87
1	CC025	F	Y	0.108	16.89	44.24	14.07	37.16
1	CC025	F	Y	0.1	15.74	47.11	13.59	34.27
2	CC025	F	Y	0.119	18.04	44.41	11.84	33.79
2	CC025	M	Y	0.199	20.7	53.61	13	63.03
0	CC027	F	Y	0.09	18.82	36.67	14.79	29.2
0	CC027	M	Y	0.134	19.78	36.33	13.39	31.41
1	CC027	M	Y	0.196	25.76	21.47	22.77	13.1
1	CC027	M	Y	0.196	25.76	21.47	22.77	13.1
1	CC027	F	Y	0.056	22.13	38.42	24.88	17.68
1	CC027	F	Y	0.066	12.76	25.69	8.77	12.76
1	CC027	M	Y	0.388	13.37	41.79	12.17	45.1

Trial#	Strain	Sex	Inf?	HindLimbSharedStanceTime_LH	MAXdA/dT_LF	MAXdA/dT_LH	MAXdA/dT_RF	MAXdA/dT_RH
1	CC027	M	Y	0.186	12.02	40.32	11.77	35.01
2	CC027	F	Y	0.189	21.52	33.16	31.5	37.2
2	CC027	M	Y	0.141	28.79	52.04	25.71	49.95
2	CC027	F	Y	0.08	15.76	31.05	15.76	19.71
2	CC027	M	Y	0.211	14.92	57.97	16.15	49.75
1	CC032XCC013	M	Y	0.116	18.14	38.96	23.16	47.47
1	CC032XCC013	M	Y	0.155	31.39	73.62	22.96	43.87
1	CC032XCC013	M	Y	0.055	17.34	36.32	14.24	28.93
1	CC032XCC013	F	Y	0.096	10.21	9.74	14.13	26.45
1	CC032XCC013	F	Y	0.08	15.28	28.49	12.97	33.49
1	CC032XCC013	F	Y	0.147	19.9	50.31	30.63	42.85
1	CC032XCC013	F	Y	0.13	19.33	53.15	30.35	42.06
1	CC032XCC013	F	Y	0.191	13.11	33.8	8.34	35.77
2	CC032XCC013	M	Y	0.174	26.09	57.49	17.15	57.63
2	CC032XCC013	M	Y	0.125	29.24	54.48	22.75	45.42
2	CC032XCC013	M	Y	0.131	18.18	58.72	22.25	41.86
2	CC032XCC013	M	Y	0.122	24.94	57.93	23.35	40.74
2	CC032XCC013	F	Y	0.176	16.4	52.98	13.16	43.14
2	CC032XCC013	F	Y	0.135	22.58	62.64	26.75	62.18
2	CC032XCC013	F	Y	0.132	42.71	69.11	32.89	54.88
2	CC032XCC013	F	Y	0.167	20.65	63.46	21.94	44.21
0	CC037	F	Y	0.153	13.48	18.41	16.73	33.43
0	CC037	M	Y	0.111	15.44	33.63	18.31	39.47
0	CC037	M	Y	0.056	11.93	35.05	14.2	41.3
0	CC037	M	Y	0.013	8.29	15.32	12.07	16.73
1	CC037	F	Y	0.186	21.66	42.87	19.82	51.51
1	CC037	M	Y	0.084	16.02	25.6	12.87	28.2
1	CC037	M	Y	0.15	14.52	40.47	17.16	47.38
1	CC037	M	Y	0.146	15.09	31.95	13.98	34.69
2	CC037	M	Y	0.165	12.87	28.27	15.39	25.4
2	CC037	M	Y	0.108	14.87	39.21	13.77	29.89
1	CC041XCC012	M	Y	0.183	32.36	41.31	22.72	45.18
1	CC041XCC012	M	Y	0.153	24.8	48.08	12.29	30.69
1	CC041XCC012	M	Y	0.065	19.93	28.08	29.11	28
1	CC041XCC012	M	Y	0.087	29.13	43.39	25.1	43.1
1	CC041XCC012	M	Y	0.164	25.7	51.7	23.26	50.64
1	CC041XCC012	F	Y	0.2	32.77	55.03	30.73	34.59
1	CC041XCC012	F	Y	0.182	35.88	61.58	27.86	46.49
1	CC041XCC012	F	Y	0.186	32.31	54.48	19.08	62.48
1	CC041XCC012	F	Y	0.169	22.59	61.4	23.85	39.96
1	CC041XCC012	F	Y	0.158	20.01	56.52	31.37	49.47
1	CC041XCC012	F	Y	0.131	36.93	41.32	11.37	57.02
1	CC041XCC012	F	Y	0.157	13.6	75.6	25.7	61.06
1	CC041XCC012	F	Y	0.2	23.38	54.46	30.6	41.5
2	CC041XCC012	M	Y	0.21	36.25	68.64	38.35	55.82
2	CC041XCC012	M	Y	0.234	40.56	80.94	33.11	89.48
2	CC041XCC012	F	Y	0.142	43.94	75.49	40.97	83.46
2	CC041XCC012	M	Y	0.098	21.47	19.52	27.39	69.99

Trial#	Strain	Sex	Inf?	HindLimbSharedStanceTime_LH	MAXdA/dT_LF	MAXdA/dT_LH	MAXdA/dT_RF	MAXdA/dT_RH
2	CC041XCC012	M	Y	0.181	38.16	58.61	37	85.23
2	CC041XCC012	M	Y	0.074	27.46	36.54	31.02	66.11
2	CC041XCC012	F	Y	0.23	32.99	50.19	32.57	59.3
2	CC041XCC012	F	Y	0.193	34.34	47.06	39.04	55.63
2	CC041XCC012	F	Y	0.203	35.03	67.08	28.01	58.37
2	CC041XCC012	F	Y	0.128	33.92	50.07	35.89	54.79
2	CC041XCC012	F	Y	0.198	36.9	53.58	28.76	47.14
2	CC041XCC012	F	Y	0.229	31.78	62.66	34.75	67.75
2	CC041XCC012	F	Y	0.168	42.85	119.53	35.37	91.01
0	CC051	F	Y	0.144	15.85	39.04	13.53	34.13
0	CC051	F	Y	0.15	14.79	32.59	7.31	33.57
0	CC051	F	Y	0.131	14.95	28.46	9.53	28.67
0	CC051	M	Y	0.116	15.38	32.37	18.43	30.86
1	CC051	F	Y	0.15	12.86	46.06	14.86	39.15
1	CC051	F	Y	0.155	22.56	48.78	14.34	56.93
1	CC051	F	Y	0.192	14.19	44.02	13.43	38.72
0	CC057	F	Y	0.13	22.65	41.66	23.07	44.16
0	CC057	F	Y	0.09	25.44	36.86	23.18	34.94
0	CC057	F	Y	0.102	14.36	12.56	13.54	12.55
0	CC057	M	Y	0.12	23.95	24.57	24.81	22.61
0	CC057	M	Y	0.034	26.35	13.35	20.6	16.48
0	CC057	M	Y	0.079	19.2	28.73	22.68	26.66
1	CC057	F	Y	0.112	27.72	47.28	26.93	59.31
1	CC057	F	Y	0.077	30.51	42.61	31.93	46.99
1	CC057	F	Y	0.062	18.11	30.22	19.29	24.21
1	CC057	F	Y	0.079	20.01	26.89	15.09	14.46
1	CC057	M	Y	0.145	30.94	44.35	35.06	65.81
1	CC057	M	Y	0.156	31.29	83.97	26.54	55.19
2	CC057	F	Y	0.079	20.01	26.89	15.09	14.46
0	CC078	F	Y	0.084	25.24	41.47	23.49	46.95
0	CC078	F	Y	0.078	22.49	37.35	19.63	37.57
0	CC078	F	Y	0.101	24.92	35.65	22.68	43.17
0	CC078	M	Y	0.089	21.49	34.45	25.91	37.68
1	CC078	F	Y	0.134	25.64	40.15	26.49	40.78
1	CC078	F	Y	0.087	30.12	60.2	32.21	37.41
1	CC078	M	Y	0.07	24.71	49.33	21.82	62.72
2	CC078	F	Y	0.098	26.38	43.75	15.47	27.15
2	CC078	M	Y	0.103	19.06	44.89	16.11	47
2	CC005	M	N	0.088	10.96	24.63	13.1	18.16
2	CC005	M	Y	0.085	8.31	16.57	8.72	18.36
2	CC015	F	N	0.061	30.88	51.44	19.99	42.01
2	CC015	M	N	0.169	24.94	62.22	17.06	58.78
2	CC015	M	Y	0.174	19.85	54.41	20.42	52.48
2	CC015	M	Y	0.112	36.82	65.34	23.13	62.13
2	CC017	F	N	0.144	14.4	49.29	13.61	47.92
2	CC017	M	N	0.107	7.36	48.26	10.87	56.16
2	CC017	F	Y	0.109	22.49	39.16	29.23	59.31
2	CC023	F	N	0.091	17	46.16	15.08	45.05

Trial#	Strain	Sex	Inf?	HindLimbSharedStanceTime_LH	MAXdA/dT_LF	MAXdA/dT_LH	MAXdA/dT_RF	MAXdA/dT_RH

Table S3. Raw DigiGait measurement data. Far left column indicates time point at which data was measured: T0 = pre-infection, T1 = 21dpi, and T3 = 89dpi. DigiGait parameters listed across the top indicate which limb is associated with the data, where appropriate: FL for left fore limb, FR for right fore limb, HL for left hind limb, and HR for right hind limb.

Table S3

Trial#	Strain	Sex	Inf?	MidlineDistance_LF	MidlineDistance_LH	MidlineDistance_RF	MidlineDistance_RH
1	CC002	F	N	-3.11	1.71	-2.27	1.85
1	CC025	F	N	-2.29	1.4	-2.2	0.79
1	CC012XCC032	F	N	-2.57	2.39	-2.9	3.42
2	CC012XCC032	F	N	-3.38	1.39	-2.88	1.29
1	CC012XCC032	M	N	-2.84	1.36	-2.82	0.85
2	CC012XCC032	M	N	-2.26	1.61	-2.18	1.06
1	CC013xCC041	F	N	-0.52	4.14	0.13	2.82
2	CC013XCC041	F	N	-2.69	1.7	-2.02	2.13
1	CC013xCC041	M	N	-1.18	1.93	-1.51	1.53
2	CC013XCC041	M	N	-3.31	1.37	-3.01	1.15
1	CC032XCC013	F	N	-2.39	0.14	-2.77	1.03
2	CC032XCC013	F	N	-3.1	0.05	-3.25	0.52
1	CC041XCC012	F	N	-1.65	0.57	-1.87	0.67
1	CC041XCC012	F	N	-2.2	0.5	-2.27	0.56
2	CC041XCC012	F	N	-2.86	1.22	-2.55	0.6
1	CC032XCC013	M	N	-1.17	1.32	-0.86	2.35
2	CC032XCC013	M	N	-0.71	0.6	-0.07	1.52
1	CC041XCC012	M	N	-2.88	0.84	-2.78	-0.36
2	CC041XCC012	M	N	-3.7	1.24	-3.77	0.77
0	CC012	F	N	-2.75	0.43	-1.6	0.96
2	CC012	F	N	-3.98	-0.46	-3.2	-0.44
0	CC012	M	N	-3.13	-0.72	-2.96	-0.88
2	CC012	M	N	-2.97	0.73	-2.31	1.53
0	CC057	F	N	-1.99	1.98	-2.03	2.36
1	CC057	F	N	-3.28	1.1	-3.16	1.82
0	CC057	M	N	-1.58	1.87	-1.95	1.4
1	CC057	M	N	-3.22	1.7	-3.13	2.31
0	CC078	F	N	-1.93	1.43	-1.79	1.45
1	CC078	F	N	-1.85	0.92	-2.17	0.75
2	CC078	F	N	-2.42	-0.01	-2.04	0.32
0	CC078	M	N	-1.97	1.33	-1.25	1.18
2	CC078	M	N	-2.3	1.29	-1.87	1.19
0	CC002	F	N	-2.41	0.77	-2.64	1.16
1	CC002	F	N	-3.11	1.71	-2.27	1.85
0	CC002	M	N	-2.37	-0.98	-2.03	0.55
1	CC002	M	N	-1.15	1.11	-1.17	0.86
2	CC002	M	N	-2.96	0.79	-2.7	-0.27
0	CC006	F	N	-0.96	0.47	-1.93	1.13
1	CC006	F	N	-1.56	0.9	-2.05	0.91
2	CC006	F	N	-1.69	0.66	-1.79	0.56
0	CC006	M	N	-1.78	0.23	-1.53	1.63
0	CC023	F	N	-2.51	1.03	-3.03	0.89
1	CC023	F	N	-1.11	1.32	-1.14	1.42
2	CC023	F	N	-2.58	0.86	-3.43	0.98
0	CC023	M	N	-2.57	0.92	-2.62	0.37
1	CC023	M	N	-3.05	1.01	-2.81	1.02
0	CC027	F	N	-3.63	-0.57	-4.57	-2.38

Trial#	Strain	Sex	Inf?	MidlineDistance_LF	MidlineDistance_LH	MidlineDistance_RF	MidlineDistance_RH
1	CC027	F	N	-2.1	1.37	-2.36	0.94
2	CC027	F	N	-3.6	0.49	-3.14	0.33
0	CC027	M	N	-2.2	0.81	-3.05	-0.17
1	CC027	M	N	-3.03	-1.58	-4.05	-3.09
1	CC027	M	N	-4.22	-2.1	-3.93	-2.03
2	CC027	M	N	-3.05	1.03	-2.77	1.03
0	CC005	F	N	-2.08	1.01	-1.91	0.88
1	CC005	F	N	-2.69	1.62	-2.19	0.73
2	CC005	F	N	0.99	1.51	0.86	1.59
0	CC011	F	N	-1.82	-0.17	-1.77	0.31
1	CC011	F	N	-3.37	0.31	-4.24	1
2	CC011	F	N	-3.46	1.1	-3.51	1.35
1	CC017	F	N	-3.98	0.09	-2.6	0.41
0	CC005	M	N	-1.63	1.17	-1.89	1.31
1	CC005	M	N	-2.92	-0.99	-2.88	1.03
2	CC005	M	N	-3.01	0.83	-3.28	0.49
0	CC011	M	N	-1.66	1.18	-1.54	1.17
1	CC011	M	N	-2.29	1.44	-2.34	1.08
2	CC011	M	N	-2.24	1.23	-1.94	0.94
0	CC017	M	N	-2.39	-0.25	-2.15	-1.01
1	CC017	M	N	-3.5	0.52	-4.3	0.38
1	CC006	F	N	0.02	0.54	-1.24	0.31
0	CC037	F	N	-2.08	1.25	-2.19	1.5
0	CC051	F	N	-2	1.15	-1.91	1.28
1	CC051	F	N	-2.14	0.86	-2.73	1.09
1	CC006	M	N	-1.83	1.22	-1.17	1.44
0	CC037	M	N	-2.1	1.59	-2.08	1.34
1	CC037	M	N	-2.5	1.54	-1.24	1.2
0	CC005	F	N	-1.32	1.52	-1.41	1.89
1	CC005	F	N	-1.65	2.3	-2.2	2.11
2	CC005	F	N	-1.99	1.34	-1.97	2.06
0	CC011	F	N	-2.42	0.4	-2.23	0.63
1	CC011	F	N	-3.38	2.55	-3.41	3.17
2	CC011	F	N	-1.63	2.44	-1.57	2.45
0	CC011	M	N	-2.02	1.47	-2.78	2.33
1	CC011	M	N	-2.85	2.96	-3.42	3.13
2	CC011	M	N	-3.09	0.8	-3.5	0.94
1	CC037	M	N	-2.15	2.41	-2.33	2.49
2	CC037	M	N	-2.81	1.54	-2.49	1.73
1	CC051	M	N	-2.63	2.53	-2.39	2.45
2	CC051	M	N	-2.25	1.74	-2.24	0.86
0	CC027	F	N	-1.89	1.74	-1.8	2.19
1	CC027	F	N	-1.85	1.24	-1.69	1
2	CC027	F	N	-1.47	1.38	-1.64	1.96
0	CC015	M	N	-1.19	1.24	-2	1.75
1	CC015	M	N	-0.63	2.82	0.55	3.2
2	CC015	M	N	-3.21	0.38	-3.86	0.22
0	CC027	M	N	-2.15	1.57	-2.02	2.29

Trial#	Strain	Sex	Inf?	MidlineDistance_LF	MidlineDistance_LH	MidlineDistance_RF	MidlineDistance_RH
1	CC027	M	N	-1.82	2.55	-2.41	2.53
2	CC027	M	N	-1.74	0.99	-2.34	0.78
0	CC015	F	N	1.9	-1.78	2.14	-5.84
1	CC015	F	N	-1.16	1.01	-0.7	1.18
0	CC017	F	N	-1.87	1.39	-1.87	1.17
0	CC023	F	N	-1.76	1.79	-0.99	1.98
1	CC023	F	N	-2.21	1.55	-2.41	1.4
0	CC005	M	N	-2.71	-0.26	-2.08	1.11
1	CC005	M	N	-1.27	1.93	-1.23	1.92
0	CC015	M	N	-1.39	1.38	-1.27	1.86
1	CC015	M	N	-2.31	1.88	-0.08	1.73
0	CC017	M	N	-2.41	0.46	-2.52	1.63
1	CC017	M	N	-1.64	1.04	-2.2	0.65
0	CC023	M	N	-1.58	1.61	-1.86	1.79
1	CC023	M	N	-1.93	1.18	-2.18	1.08
0	CC051	M	N	-1.72	1.05	-2.12	1.86
0	CC002	F	Y	-2.34	-0.02	-2.45	0.07
0	CC002	M	Y	-1.8	0.71	-2.16	0.83
1	CC002	M	Y	-2.1	-4.74	-2.44	0.28
1	CC002	F	Y	-1.31	1.17	-2.12	-0.5
1	CC002	F	Y	-1.62	0.52	-2.04	1.02
1	CC002	M	Y	-2.1	-4.74	-2.44	0.28
1	CC002	M	Y	-3.42	-0.21	-3.94	-0.57
1	CC002	F	Y	-1.31	1.17	-2.12	-0.5
1	CC002	F	Y	-1.2	2.23	-1.78	1.74
2	CC002	M	Y	-1.36	1.62	-0.66	0.45
2	CC002	F	Y	-3.25	1.58	-2.77	1.62
2	CC002	M	Y	-0.84	1.06	-0.67	1.52
0	CC005	F	Y	-1.94	0.82	-1.86	0.48
0	CC005	M	Y	-2.34	0.69	-2.35	0.82
0	CC005	M	Y	-1.77	0.32	-1.32	1.06
1	CC005	F	Y	-2	0.84	-1.85	1.78
1	CC005	M	Y	-3.27	2.49	-3.08	0.13
1	CC005	F	Y	-2.74	2.97	-1.88	3.01
1	CC005	M	Y	-2.07	1.69	-2.19	1.49
2	CC005	F	Y	-3.87	-1.27	-3	-0.25
2	CC005	M	Y	-4.66	0.56	-4.03	-0.17
2	CC005	M	Y	-2.79	-0.7	-4.42	-1.38
2	CC005	M	Y	-4.66	0.56	-4.03	-0.17
2	CC005	F	Y	-1.75	2.24	-1.64	0.98
0	CC006	F	Y	-1.65	0.66	-1.93	1.21
0	CC006	M	Y	-1.93	1.11	-1.79	0.2
0	CC006	F	Y	-1.23	1.26	-1.82	0.78
1	CC006	F	Y	-1.35	0.14	-1.56	1.16
1	CC006	M	Y	-1.6	0.23	-2.05	0.62
1	CC006	F	Y	-1.97	0.12	-2.38	0.45
1	CC006	M	Y	-2.34	1.66	-1.36	1.75
2	CC006	F	Y	-1.75	0.6	-1.89	0.87

Trial#	Strain	Sex	Inf?	MidlineDistance_LF	MidlineDistance_LH	MidlineDistance_RF	MidlineDistance_RH
2	CC006	M	Y	-1.29	-0.07	-1.04	0.84
0	CC011	F	Y	-2.87	-0.54	-3.68	-0.02
0	CC011	M	Y	-1.82	1.27	-1.41	0.83
0	CC011	F	Y	-1.43	1.21	-1.38	1.37
0	CC011	F	Y	-0.27	2.85	0.13	2.19
1	CC011	F	Y	-2.33	1.55	-1.71	1.39
1	CC011	M	Y	-1.96	1.74	-2.52	2.13
1	CC011	F	Y	-1.94	3.4	-2.52	3.33
1	CC011	M	Y	-2.05	2.49	-1.85	2.47
2	CC011	F	Y	-2.7	2.27	-2.09	2.01
2	CC011	M	Y	-5.03	-0.99	-5.02	-0.55
2	CC011	F	Y	-1.65	2.04	-1.64	1.87
2	CC011	M	Y	-1.87	1.96	-2.12	1.79
0	CC012	M	Y	-3.86	0.26	-3.86	0.49
0	CC012	M	Y	-1.86	0.94	-2.42	0.28
0	CC012	M	Y	-3.46	0	-3.22	0.06
0	CC012	M	Y	-2.62	0.55	-2.79	-0.33
0	CC012	M	Y	-2.72	0.75	-2.95	0.28
0	CC012	F	Y	-2.64	0.45	-2.65	0.41
0	CC012	F	Y	-4.25	-0.06	-3.65	-0.31
0	CC012	F	Y	-1.63	0.48	-2.85	0.76
0	CC012	F	Y	-2.11	0.24	-2.4	1.04
0	CC012	F	Y	-2.05	0.3	-1.97	0.21
2	CC012	M	Y	-3.07	-0.98	-3.02	0.14
2	CC012	M	Y	-4.29	-0.73	-4.24	-0.59
2	CC012	M	Y	-4.22	-0.39	-2.88	0.08
2	CC012	M	Y	-6.09	0.34	-5.83	0.36
2	CC012	M	Y	-4.13	-0.17	-3.96	-0.5
2	CC012	F	Y	-3.52	1.36	-3.07	1.33
2	CC012	F	Y	-2.47	2.31	-2.69	0.72
2	CC012	F	Y	-3.36	5.35	-4.14	-0.63
2	CC012	F	Y	-3.55	-0.05	-2.25	1.24
2	CC012	F	Y	-3.07	0.4	-3.08	1.17
1	CC012XCC032	F	Y	-0.18	2.66	0.31	2.09
1	CC012xCC032	F	Y	-1.95	0.4	-2.61	0.29
1	CC012XCC032	M	Y	-2.06	1.09	-2.52	0.84
1	CC012xCC032	M	Y	-1.97	1.16	-2.07	0.41
1	CC012XCC032	M	Y	-2.47	1.25	-1.06	0.85
1	CC012xCC032	M	Y	-3.08	0.9	-2.57	0.42
2	CC012XCC032	F	Y	-2.74	1.71	-3.47	2.03
2	CC012XCC032	F	Y	-2.88	1.47	-3.46	1.63
2	CC012XCC032	M	Y	-3.31	1.11	-2.75	0.25
2	CC012XCC032	M	Y	-2.62	0.98	-2.55	1.7
2	CC012XCC032	M	Y	-2.84	0.94	-3.4	0.63
2	CC012XCC032	M	Y	-2.1	0.4	-1.57	2.2
1	CC013xCC041	F	Y	-2.34	1.37	0.81	3.37
1	CC013xCC041	F	Y	-0.19	2.59	-1.34	0.86
1	CC013xCC041	F	Y	-1.4	2.3	-1.28	1.36

Trial#	Strain	Sex	Inf?	MidlineDistance_LF	MidlineDistance_LH	MidlineDistance_RF	MidlineDistance_RH
1	CC013xCC041	M	Y	-1.61	0.51	-1.6	0.47
1	CC013xCC041	M	Y	-3.34	1.8	-2.24	1.27
2	CC013XCC041	F	Y	-2.66	1.68	-2.77	1.19
2	CC013XCC041	F	Y	-2.75	1.44	-1.63	0.92
2	CC013XCC041	F	Y	-2.51	1.15	-2.22	1.23
2	CC013XCC041	M	Y	-2.52	0.41	-2.82	1.23
2	CC013XCC041	M	Y	-1.75	0.43	-1.3	1.49
0	CC015	F	Y	-1.02	0.36	-1.36	1.43
0	CC015	M	Y	-1.63	2.06	-1.64	1.86
0	CC015	M	Y	-1.25	2	-1.43	1.63
1	CC015	F	Y	-1.61	1.98	-1.46	2.11
1	CC015	M	Y	-1.26	1.02	-1.25	0.95
1	CC015	M	Y	-1.22	1.91	-1.38	1.72
1	CC015	M	Y	-1.45	1.32	-1.54	1.22
2	CC015	F	Y	-1.65	1.89	-1.29	2.02
0	CC017	F	Y	-2.24	0.38	-1.37	0.41
0	CC017	M	Y	-2.07	0.69	-2.29	0.37
0	CC017	F	Y	-2.16	0.56	-2.16	0.17
0	CC017	M	Y	-1.85	0.25	-2.05	-0.01
1	CC017	M	Y	-1.88	1.04	-2.6	-0.05
1	CC017	F	Y	-1.76	0.39	-1.27	0.49
0	CC023	M	Y	-3.69	0.32	-3.75	0.27
0	CC023	F	Y	-2.74	0.58	-2.98	0.52
0	CC023	F	Y	-1.2	0.44	-1.16	0.26
0	CC023	M	Y	-3.6	0.12	-2.51	-0.59
1	CC023	M	Y	-3.99	-0.91	-3.9	-0.6
1	CC023	F	Y	-4.32	-1.32	-4.53	-2.33
1	CC023	M	Y	-1.96	0.93	-2.05	1.19
1	CC023	M	Y	-1.27	2.53	-2.04	2.5
2	CC023	M	Y	-3.49	0.74	-4.23	0.87
2	CC023	F	Y	-1.76	0.59	-1.03	1.21
2	CC023	M	Y	-1.51	-0.56	-3.75	-0.85
0	CC025	M	Y	-1.01	2.22	-1.13	2.07
0	CC025	F	Y	-2.01	1.3	-1.61	1.74
0	CC025	M	Y	-1.44	0.76	-1.59	0.98
1	CC025	M	Y	-1.05	2.27	-1.16	1.99
1	CC025	F	Y	-0.58	2.67	-0.73	1.49
1	CC025	F	Y	-1.11	2.95	-0.9	2.35
1	CC025	F	Y	-3.23	0.33	-2.68	0.66
2	CC025	F	Y	-1.64	0.75	-3.35	1.31
2	CC025	M	Y	-2.09	1.77	-2.6	1.43
0	CC027	F	Y	-1.45	0.52	-3.61	0.22
0	CC027	M	Y	-3.27	-0.31	-3.19	-0.09
1	CC027	M	Y	-1.6	1.84	-4.5	-1.01
1	CC027	M	Y	-1.6	1.84	-4.5	-1.01
1	CC027	F	Y	-1.81	0.54	-2.42	-0.19
1	CC027	F	Y	-1.59	1.04	-2.27	0.39
1	CC027	M	Y	-1.96	1.78	-1.32	1.66

Trial#	Strain	Sex	Inf?	MidlineDistance_LF	MidlineDistance_LH	MidlineDistance_RF	MidlineDistance_RH
1	CC027	M	Y	-1.96	1.2	-1.87	1.64
2	CC027	F	Y	-1.76	0.59	-1.03	1.21
2	CC027	M	Y	-2.82	0.95	-2.86	1.66
2	CC027	F	Y	-2.61	0.59	-2.73	0.37
2	CC027	M	Y	-2.49	1.82	-1.87	1.83
1	CC032XCC013	M	Y	-0.92	1.11	-2.02	0.55
1	CC032XCC013	M	Y	-2.43	1.37	-2.85	1.6
1	CC032XCC013	M	Y	-0.35	1.21	-3.49	1.53
1	CC032XCC013	F	Y	-2.31	0.78	-2.58	1
1	CC032XCC013	F	Y	-2.64	0.6	-2.19	0.78
1	CC032XCC013	F	Y	-2.97	-0.01	-3.33	0.94
1	CC032XCC013	F	Y	-1.45	1.51	-2.52	2.3
1	CC032XCC013	F	Y	-3.22	1.22	-3.1	1.5
2	CC032XCC013	M	Y	-2.59	1.3	-1.65	1.87
2	CC032XCC013	M	Y	-0.45	2.09	-0.63	2.44
2	CC032XCC013	M	Y	-2.07	1.75	-2.78	2.3
2	CC032XCC013	M	Y	-2.91	1.12	-2.61	0.19
2	CC032XCC013	F	Y	-2.83	1.43	-2.69	1.86
2	CC032XCC013	F	Y	-2.35	2.1	-2.71	1.9
2	CC032XCC013	F	Y	-2.16	1.65	-2.37	2.19
2	CC032XCC013	F	Y	-2.98	1.08	-3.05	0.86
0	CC037	F	Y	-2.24	0.65	-2.26	0.89
0	CC037	M	Y	-1.49	1.23	-1.58	1.41
0	CC037	M	Y	-0.51	1.22	-1.57	1.19
0	CC037	M	Y	-0.02	1.4	0	2.51
1	CC037	F	Y	-2.13	0.87	-2.42	1.03
1	CC037	M	Y	-1.78	1.72	-2.3	2.01
1	CC037	M	Y	-2.44	2.47	-2.39	2.33
1	CC037	M	Y	-1.94	1.81	-1.39	2.01
2	CC037	M	Y	-2.47	1.84	-3.32	1.62
2	CC037	M	Y	-2.34	1.72	-2.42	1.98
1	CC041XCC012	M	Y	-3.86	-1.37	-3.41	-1.14
1	CC041XCC012	M	Y	-3.66	-0.72	-3.87	-0.7
1	CC041XCC012	M	Y	-4.11	0.02	-2.63	0.79
1	CC041XCC012	M	Y	-3.13	-0.18	-2.06	0.05
1	CC041XCC012	M	Y	-3.55	1.5	-2.46	0.25
1	CC041XCC012	F	Y	-2.88	1.13	-3.12	1.39
1	CC041XCC012	F	Y	-2.51	1.11	-2.51	1.09
1	CC041XCC012	F	Y	-2.6	1.47	-3.45	1.1
1	CC041XCC012	F	Y	-2.67	1.4	-2.33	0.27
1	CC041XCC012	F	Y	-2.81	0.42	-4.26	0.15
1	CC041XCC012	F	Y	-4.07	1.44	-2.61	1.33
1	CC041XCC012	F	Y	-2.52	1.06	-2.71	1.36
1	CC041XCC012	F	Y	-2.56	0.62	-2.82	0.42
2	CC041XCC012	M	Y	-2.32	2.01	-2.45	1.3
2	CC041XCC012	M	Y	-2.75	1.3	-2.26	0.28
2	CC041XCC012	F	Y	-2.79	1.4	-3.09	1.41
2	CC041XCC012	M	Y	-2.09	1.78	-1.49	1.03

Trial#	Strain	Sex	Inf?	MidlineDistance_LF	MidlineDistance_LH	MidlineDistance_RF	MidlineDistance_RH
2	CC041XCC012	M	Y	-2.49	1.83	-2.81	1.35
2	CC041XCC012	M	Y	-2.34	2.04	-1.73	0.89
2	CC041XCC012	F	Y	-2.56	1.59	-2.52	1.36
2	CC041XCC012	F	Y	-1.77	1.43	-2.42	1.53
2	CC041XCC012	F	Y	-2.17	1.92	-2.1	2.23
2	CC041XCC012	F	Y	-2.34	2.16	-2.42	1.91
2	CC041XCC012	F	Y	-3	1.71	-2.93	2.01
2	CC041XCC012	F	Y	-2.68	1.9	-2.3	2.29
2	CC041XCC012	F	Y	-2.88	0.11	-2.43	0.7
0	CC051	F	Y	-2.7	1.33	-1.9	0.92
0	CC051	F	Y	-1.93	1.29	-1.73	1.57
0	CC051	F	Y	-2.12	1.16	-1.97	1.57
0	CC051	M	Y	-1.38	1.42	-1.69	1.61
1	CC051	F	Y	-2.27	2.24	-0.98	2.42
1	CC051	F	Y	-1.92	2.01	-1.38	2.19
1	CC051	F	Y	-2.52	1.67	-2.05	1.73
0	CC057	F	Y	-2.01	2.08	-2.14	2.11
0	CC057	F	Y	-2.28	1.04	-2.7	1.54
0	CC057	F	Y	-2.15	0.8	-2.19	1.06
0	CC057	M	Y	-0.75	1.76	-1.62	3.04
0	CC057	M	Y	-2.6	0.32	-2	1.2
0	CC057	M	Y	-1.64	1.87	-1.42	0.58
1	CC057	F	Y	-2.3	1.13	-3	1.34
1	CC057	F	Y	-2.6	0.07	-1.27	1.52
1	CC057	F	Y	-2.24	0.54	-0.42	1.56
1	CC057	F	Y	-3.02	-0.69	-4	-0.66
1	CC057	M	Y	-2.61	1.17	-3.39	1.45
1	CC057	M	Y	-2.26	1.9	-2.71	1.99
2	CC057	F	Y	-3.02	-0.69	-4	-0.66
0	CC078	F	Y	-1.71	1.19	-1.33	1.28
0	CC078	F	Y	-1.66	1.36	-1.88	1.9
0	CC078	F	Y	-0.88	1.82	-1.71	1.84
0	CC078	M	Y	-1.36	1.37	-1.47	1.72
1	CC078	F	Y	-3.29	1.17	-3.04	1.07
1	CC078	F	Y	-1.15	1.15	-1.65	1.96
1	CC078	M	Y	-1.44	1.34	-1.71	1.04
2	CC078	F	Y	-3.64	0.29	-2.28	0.31
2	CC078	M	Y	-2.14	1.27	-2.56	0.82
2	CC005	M	N	-2.18	0.8	-1.91	2.5
2	CC005	M	Y	-0.93	1.5	-1.23	1.79
2	CC015	F	N	-0.16	1.79	-2.34	0.94
2	CC015	M	N	-1.74	2.07	-1.64	2.33
2	CC015	M	Y	-2.23	2	-1.92	1.65
2	CC015	M	Y	-1.05	1.63	-1.8	1.02
2	CC017	F	N	-1.74	1.55	-2.35	1.2
2	CC017	M	N	-0.32	1.32	-0.84	1.85
2	CC017	F	Y	-1.61	1.01	-1.36	1.07
2	CC023	F	N	-1.34	2.17	-1.19	1.86

Trial#	Strain	Sex	Inf?	MidlineDistance_LF	MidlineDistance_LH	MidlineDistance_RF	MidlineDistance_RH

Table S3. Raw DigiGait measurement data. Far left column indicates time point at which data was measured: T0 = pre-infection, T1 = 21dpi, and T3 = 89dpi. DigiGait parameters listed across the top indicate which limb is associated with the data, where appropriate: FL for left fore limb, FR for right fore limb, HL for left hind limb, and HR for right hind limb.

Table S3

Trial#	Strain	Sex	Inf?	MINdA/dT_LF	MINdA/dT_LH	MINdA/dT_RF	MINdA/dT_RH	OverlapDistance_LF
1	CC002	F	N	-6.3	-5.37	-4.04	-9.04	1.66
1	CC025	F	N	-3.61	-11.05	-2.56	-8.74	1.33
1	CC012XCC032	F	N	-6.17	-9.12	-7.74	-11.1	2.26
2	CC012XCC032	F	N	-9.6	-13.17	-8.72	-11.66	0.77
1	CC012XCC032	M	N	-6.29	-10.26	-8.56	-9.28	1.92
2	CC012XCC032	M	N	-5.68	-8.02	-3.84	-12.72	0.55
1	CC013xCC041	F	N	-12.32	-16.08	-17.65	-23.26	2.27
2	CC013XCC041	F	N	-6.87	-7.54	-5.29	-8.88	2.56
1	CC013xCC041	M	N	-4.07	-9.05	-4.2	-2.94	1.69
2	CC013XCC041	M	N	-8.33	-7.22	-5.53	-11.96	2.65
1	CC032XCC013	F	N	-5.86	-18.71	-10.5	-11.85	1.55
2	CC032XCC013	F	N	-14.13	-11.26	-9.27	-13.4	0.52
1	CC041XCC012	F	N	-16.22	-21.97	-21.18	-15.67	-0.14
1	CC041XCC012	F	N	-10.2	-11.23	-10.39	-16.82	2.21
2	CC041XCC012	F	N	-3.72	-21.5	-12.33	-9.84	1.23
1	CC032XCC013	M	N	-25.12	-16.93	-13.42	-15.99	0.94
2	CC032XCC013	M	N	-11.68	-9.57	-13.69	-7.25	-6.64
1	CC041XCC012	M	N	-9.93	-8.24	-8.91	-7.24	1.87
2	CC041XCC012	M	N	-12.03	-7.02	-8.04	-8.51	2.63
0	CC012	F	N	-7.22	-10.79	-17.29	-12.11	0.89
2	CC012	F	N	-13.37	-8.02	-18.06	-18.13	1.35
0	CC012	M	N	-6.65	-9.18	-6.17	-9.71	1.03
2	CC012	M	N	-10.99	-12.1	-8.22	-10.88	0.85
0	CC057	F	N	-5.42	-12.02	-7.12	-11.16	1.61
1	CC057	F	N	-4.9	-13.09	-5.43	-15.53	1.72
0	CC057	M	N	-8.07	-7.4	-12.27	-15.48	2.07
1	CC057	M	N	-5.46	-14.79	-6.48	-10.5	1.85
0	CC078	F	N	-5.3	-8.6	-5.21	-7.02	1.3
1	CC078	F	N	-8.76	-8.28	-8.82	-9.08	1.09
2	CC078	F	N	-8.37	-10.28	-10.86	-11.46	1.14
0	CC078	M	N	-5.54	-7.43	-5.41	-6.51	1.35
2	CC078	M	N	-9.8	-8.78	-8.77	-12.59	1.13
0	CC002	F	N	-11.18	-20.7	-12.21	-13.95	1.41
1	CC002	F	N	-6.3	-5.37	-4.04	-9.04	1.66
0	CC002	M	N	-10.66	-14.46	-10.72	-8.4	0.73
1	CC002	M	N	-23.92	-23.09	-13.06	-9.17	0.09
2	CC002	M	N	-5.48	-12.56	-9.15	-20.59	2.14
0	CC006	F	N	-18.2	-13.35	-6.62	-12	0.8
1	CC006	F	N	-8.99	-12.89	-9.2	-6.28	0.58
2	CC006	F	N	-6.56	-13.09	-10.43	-19.23	1.22
0	CC006	M	N	-9.18	-15.63	-8.95	-13.43	1.07
0	CC023	F	N	-6.72	-8.54	-4.86	-8.26	1.4
1	CC023	F	N	-16.28	-14.21	-14.38	-12.82	0.73
2	CC023	F	N	-28.36	-8.68	-18.74	-10.1	0.16
0	CC023	M	N	-6.21	-7.13	-5.08	-8.7	1.09
1	CC023	M	N	-6.88	-8.8	-3.46	-10.1	1.4
0	CC027	F	N	-8.79	-3.94	-7.17	-6.06	1.63

Trial#	Strain	Sex	Inf?	MINdA/dT_LF	MINdA/dT_LH	MINdA/dT_RF	MINdA/dT_RH	OverlapDistance_LF
1	CC027	F	N	-15.85	-11.08	-9.6	-8.03	1.14
2	CC027	F	N	-3.69	-10.64	-4.75	-16.65	1.94
0	CC027	M	N	-5.38	-5.32	-7.6	-6	1.42
1	CC027	M	N	-11.05	-11.55	-13.19	-11.8	0.77
1	CC027	M	N	-12.7	-15.19	-8.8	-10.3	-0.41
2	CC027	M	N	-5.14	-9.52	-7.02	-10.72	1.75
0	CC005	F	N	-8.14	-10.85	-4.59	-12.11	1.07
1	CC005	F	N	-2.55	-8.03	-4.95	-8.2	2.08
2	CC005	F	N	-3.86	-4.71	-5.16	-4.74	0.52
0	CC011	F	N	-12.26	-8.12	-17.6	-28.51	0.6
1	CC011	F	N	-27.01	-26.9	-25.31	-20.33	1.39
2	CC011	F	N	-6.5	-6.66	-11.79	-7.69	2.08
1	CC017	F	N	-13.37	-11.2	-6.77	-11.01	2.01
0	CC005	M	N	-5.9	-8.39	-4.51	-13.13	1.39
1	CC005	M	N	-10.43	-9.31	-12.57	-10.47	1.47
2	CC005	M	N	-7.08	-8.16	-9.42	-6.65	2.94
0	CC011	M	N	-2.5	-7.61	-1.97	-16.48	0.94
1	CC011	M	N	-3.22	-8.1	-3.93	-7.44	1.26
2	CC011	M	N	-23.54	-21.39	-14.72	-19.67	0.75
0	CC017	M	N	-4.15	-2.45	-3.58	-3.67	1.75
1	CC017	M	N	-7.96	-12.33	-5.67	-10.34	2.19
1	CC006	F	N	-12.6	-15.55	-9.46	-15.14	-0.11
0	CC037	F	N	-8.83	-10.54	-6.78	-11.84	-0.96
0	CC051	F	N	-6.15	-6.65	-4.94	-7.02	-0.46
1	CC051	F	N	-7.62	-4.14	-3.17	-5.06	1.66
1	CC006	M	N	-12.91	-10.26	-9.81	-15.13	1.07
0	CC037	M	N	-3.54	-8.52	-5.6	-10.38	1.48
1	CC037	M	N	-5.89	-9.7	-13.69	-11.87	0.84
0	CC005	F	N	-6.88	-8.43	-3.47	-9.53	0.86
1	CC005	F	N	-13.49	-12.47	-8.15	-12.91	1.92
2	CC005	F	N	-6	-6.69	-5.85	-8.93	3.43
0	CC011	F	N	-2.99	-5.93	-6.6	-5.74	0.96
1	CC011	F	N	-4.38	-12.68	-5.32	-16.31	3.47
2	CC011	F	N	-2.23	-6.27	-2.14	-6.19	1.78
0	CC011	M	N	-3.66	-11.39	-4.47	-15.64	1.76
1	CC011	M	N	-3.45	-11.51	-3.53	-15.97	3.31
2	CC011	M	N	-1.84	-6.25	-2.07	-5.9	1.06
1	CC037	M	N	-13.65	-11.87	-18.36	-12.09	1.95
2	CC037	M	N	-3.06	-5.63	-5.55	-7.84	1.45
1	CC051	M	N	-3.58	-9.44	-4.87	-7.5	2.56
2	CC051	M	N	-4.17	-6.06	-3.48	-6.39	0.39
0	CC027	F	N	-7.75	-10.81	-4.92	-8.91	1.92
1	CC027	F	N	-8.3	-8.19	-10.86	-6.61	1.99
2	CC027	F	N	-11.61	-8.53	-9.55	-12.12	0.7
0	CC015	M	N	-2.82	-4.56	-3.74	-5.18	-0.45
1	CC015	M	N	-5.94	-23.39	-10.16	-17.89	1.6
2	CC015	M	N	-3.12	-8.05	-2.45	-15.48	0.55
0	CC027	M	N	-4.99	-5.67	-2.88	-6.32	1.58

Trial#	Strain	Sex	Inf?	MINdA/dT_LF	MINdA/dT_LH	MINdA/dT_RF	MINdA/dT_RH	OverlapDistance_LF
1	CC027	M	N	-14.03	-10.33	-4.52	-26.59	2.6
2	CC027	M	N	-3.72	-16.01	-1.83	-7.62	2.07
0	CC015	F	N	-9.19	-6.96	-8	-0.69	-0.69
1	CC015	F	N	-9.03	-12.91	-10.59	-13.64	0.96
0	CC017	F	N	-7.25	-17.88	-8.14	-10.02	1.15
0	CC023	F	N	-7.92	-6.73	-10.18	-8.5	0.84
1	CC023	F	N	-6.4	-12.2	-7.35	-12.44	1.37
0	CC005	M	N	-7.27	-13.36	-12.41	-5.41	1.61
1	CC005	M	N	-11.42	-10.93	-7.94	-18.19	1.47
0	CC015	M	N	-8.8	-9.4	-9.89	-7.05	0.43
1	CC015	M	N	-14.33	-14.34	-14.18	-15.22	1.01
0	CC017	M	N	-10.9	-11.84	-4.4	-10.27	1.44
1	CC017	M	N	-13.43	-13.69	-9.3	-10.98	1.62
0	CC023	M	N	-6.9	-8.95	-4.77	-8.44	0.79
1	CC023	M	N	-13.41	-15.76	-8.62	-13.11	1.14
0	CC051	M	N	-6.02	-7.19	-3.43	-8.24	1.22
0	CC002	F	Y	-17.27	-13.19	-21.82	-15.11	1.29
0	CC002	M	Y	-10.5	-7.05	-11.85	-10.38	0.85
1	CC002	M	Y	-5.97	-7.51	-7.39	-5.63	-0.98
1	CC002	F	Y	-7.85	-5.16	-10.47	-9.71	2.17
1	CC002	F	Y	-13.67	-17.27	-14.07	-19.04	0.93
1	CC002	M	Y	-5.97	-7.51	-7.39	-5.63	-0.98
1	CC002	M	Y	-10	-13.6	-8.56	-6.16	1.25
1	CC002	F	Y	-7.85	-5.16	-10.47	-9.71	2.17
1	CC002	F	Y	-12.64	-7.35	-12.19	-8.2	2.2
2	CC002	M	Y	-14.17	-13.07	-15.53	-12.69	2.53
2	CC002	F	Y	-4.86	-8.54	-5.93	-10.94	1.89
2	CC002	M	Y	-6.57	-22.73	-8.85	-11.29	0.82
0	CC005	F	Y	-4.1	-8.9	-4.32	-9.86	0.49
0	CC005	M	Y	-5.62	-8.53	-8.65	-7.92	1.27
0	CC005	M	Y	-10.07	-16.03	-24.06	-11.11	0.5
1	CC005	F	Y	-6.94	-10.03	-10.04	-9.28	1.33
1	CC005	M	Y	-8.88	-5.16	-8.96	-10.53	4.05
1	CC005	F	Y	-11.6	-15.9	-11.82	-15.25	3.97
1	CC005	M	Y	-8.79	-16.48	-14.53	-15.32	2.76
2	CC005	F	Y	-14.15	-14.11	-13.55	-12.77	1.43
2	CC005	M	Y	-10.65	-8.06	-10.82	-17.19	2.78
2	CC005	M	Y	-20.91	-16.78	-13.23	-14.73	0.84
2	CC005	M	Y	-10.65	-8.06	-10.82	-17.19	2.78
2	CC005	F	Y	-9.99	-7.62	-6.1	-15.49	2.21
0	CC006	F	Y	-3.97	-6.72	-7.16	-7.7	0.86
0	CC006	M	Y	-4.9	-4.97	-4.59	-3.22	1.32
0	CC006	F	Y	-11.34	-11.93	-10.26	-6.92	0.49
1	CC006	F	Y	-9.64	-9.4	-5.18	-7.85	0.96
1	CC006	M	Y	-9.76	-9.36	-11.43	-9.33	1.21
1	CC006	F	Y	-17.9	-9.19	-7.05	-9.28	0.5
1	CC006	M	Y	-6.4	-13.77	-7.07	-8.36	1.01
2	CC006	F	Y	-7.06	-14.27	-6.08	-16.33	1.12

Trial#	Strain	Sex	Inf?	MINdA/dT_LF	MINdA/dT_LH	MINdA/dT_RF	MINdA/dT_RH	OverlapDistance_LF
2	CC006	M	Y	-14.1	-22.98	-12.39	-20.38	-0.07
0	CC011	F	Y	-24.83	-20.59	-23.46	-22.71	1.27
0	CC011	M	Y	-7.08	-14.94	-5.23	-12.14	1.09
0	CC011	F	Y	-2.06	-9.6	-2.94	-7.5	1.42
0	CC011	F	Y	-9.28	-27.39	-6.42	-23.18	1
1	CC011	F	Y	-4.1	-5.53	-3	-6.67	1.7
1	CC011	M	Y	-3.49	-5.7	-2.81	-6.23	2.26
1	CC011	F	Y	-5.67	-11.77	-4.64	-12.55	2.52
1	CC011	M	Y	-6.54	-10.55	-5.92	-14.51	2.58
2	CC011	F	Y	-3.65	-8.22	-4.32	-8.97	1.77
2	CC011	M	Y	-17.34	-10.19	-12.9	-6.41	1.96
2	CC011	F	Y	-1.71	-6.11	-2.07	-6.15	0.75
2	CC011	M	Y	-7.97	-8.05	-2.43	-9.25	1.09
0	CC012	M	Y	-6.73	-11.64	-7.02	-10.3	1.06
0	CC012	M	Y	-5.68	-27.37	-12.58	-11.27	0
0	CC012	M	Y	-5.67	-7.83	-4.8	-9.88	1.15
0	CC012	M	Y	-9.59	-9.02	-8.62	-12.47	0.82
0	CC012	M	Y	-6.56	-9.15	-7.67	-12.6	0.7
0	CC012	F	Y	-9.67	-17.08	-7.34	-12	1.76
0	CC012	F	Y	-7.53	-13.5	-6.97	-10.12	1.24
0	CC012	F	Y	-12.78	-13.65	-5.37	-9.97	0.42
0	CC012	F	Y	-18.72	-20.26	-25.15	-24.46	0.8
0	CC012	F	Y	-12.69	-26.72	-5.37	-16.3	0.74
2	CC012	M	Y	-5.74	-11.45	-6.08	-11.12	0.65
2	CC012	M	Y	-8.18	-12.87	-8.71	-14.07	0.86
2	CC012	M	Y	-10.01	-10.44	-15.29	-12.7	0.82
2	CC012	M	Y	-6.98	-10.56	-10.77	-11.82	-0.93
2	CC012	M	Y	-9.66	-11.73	-6.82	-12.06	1.06
2	CC012	F	Y	-5.28	-11.12	-7.23	-10.92	2.41
2	CC012	F	Y	-9.64	-10.36	-5.5	-7.67	1.15
2	CC012	F	Y	-15.46	-12.49	-11.54	-12.44	2.83
2	CC012	F	Y	-10.71	-11.4	-26.88	-15.92	0.32
2	CC012	F	Y	-6.23	-7.67	-13.32	-6.89	0.76
1	CC012XCC032	F	Y	-14.61	-24.41	-19.72	-26.96	-0.74
1	CC012xCC032	F	Y	-11.13	-7.96	-6.66	-6.29	1.1
1	CC012XCC032	M	Y	-6.97	-12.1	-6.2	-9.76	0.51
1	CC012xCC032	M	Y	-10.82	-16.08	-11.96	-10.02	-0.53
1	CC012XCC032	M	Y	-10.5	-9.41	-13.37	-16.41	1.5
1	CC012xCC032	M	Y	-9.35	-11.16	-7.32	-11.24	1.41
2	CC012XCC032	F	Y	-13.2	-14.92	-5.72	-12.66	2.29
2	CC012XCC032	F	Y	-4.56	-9.14	-9.41	-10.26	1.05
2	CC012XCC032	M	Y	-10.99	-14.36	-16.16	-15.21	1.46
2	CC012XCC032	M	Y	-14.79	-12.31	-9	-11.26	1.25
2	CC012XCC032	M	Y	-5.18	-13.36	-10.23	-10.69	1.35
2	CC012XCC032	M	Y	-8.16	-14.83	-17.34	-11.81	0.06
1	CC013xCC041	F	Y	-21.01	-15.52	-7.84	-19.55	1.5
1	CC013xCC041	F	Y	-18.62	-18.08	-17.32	-16.18	1.96
1	CC013xCC041	F	Y	-9.76	-8.47	-9.98	-9.64	1.9

Trial#	Strain	Sex	Inf?	MINdA/dT_LF	MINdA/dT_LH	MINdA/dT_RF	MINdA/dT_RH	OverlapDistance_LF
1	CC013xCC041	M	Y	-10.51	-6.72	-6.42	-12.61	0.88
1	CC013xCC041	M	Y	-15.93	-11.94	-14.55	-18.18	2.7
2	CC013XCC041	F	Y	-9.23	-11.31	-6.13	-15.16	1.76
2	CC013XCC041	F	Y	-12.72	-15.12	-21.89	-15.46	1.8
2	CC013XCC041	F	Y	-15.75	-10.7	-9.78	-14.55	2.15
2	CC013XCC041	M	Y	-8.83	-13.23	-5.9	-17.36	1.58
2	CC013XCC041	M	Y	-10.25	-18.92	-10.22	-10.98	1.77
0	CC015	F	Y	-8.64	-6.78	-11.47	-10.26	0.23
0	CC015	M	Y	-6.07	-7.04	-8.26	-9.27	1.59
0	CC015	M	Y	-6.06	-8.03	-8.39	-10.05	0.65
1	CC015	F	Y	-8.95	-7.85	-4.32	-9.03	0.69
1	CC015	M	Y	-9.14	-17.66	-9.35	-17.2	1.4
1	CC015	M	Y	-13.34	-18.2	-19.28	-24.8	2.21
1	CC015	M	Y	-9.2	-8.05	-11.45	-11.08	1.26
2	CC015	F	Y	-7.35	-6.78	-2.75	-9.41	1.44
0	CC017	F	Y	-3.01	-14.58	-9.99	-9.49	1.33
0	CC017	M	Y	-5.15	-14.59	-3.62	-11.16	1.44
0	CC017	F	Y	-17.79	-17.33	-13.49	-13.64	1
0	CC017	M	Y	-12.67	-11.94	-10.26	-6.91	0.49
1	CC017	M	Y	-9.66	-8.14	-9.96	-7.54	1.35
1	CC017	F	Y	-9.01	-11.85	-13.82	-14.68	0.51
0	CC023	M	Y	-7.13	-7.02	-5.77	-7.34	1.56
0	CC023	F	Y	-7.77	-5.73	-6.92	-5.44	0.67
0	CC023	F	Y	-8.39	-10.64	-5.96	-8.66	2.97
0	CC023	M	Y	-18.66	-9.34	-15.51	-14.07	1.72
1	CC023	M	Y	-8.95	-9.32	-9.82	-7.52	2.09
1	CC023	F	Y	-17.62	-6.6	-16.54	-7.79	0.53
1	CC023	M	Y	-13.02	-8.35	-8.43	-6.29	0.98
1	CC023	M	Y	-12.09	-8.63	-7.53	-5.02	4.07
2	CC023	M	Y	-6.17	-10.66	-5.16	-9.42	2.14
2	CC023	F	Y	-16.64	-6.78	-25.08	-25.89	1.35
2	CC023	M	Y	-3.29	-5.3	-7.73	-4.89	0.76
0	CC025	M	Y	-2.51	-15.1	-3.31	-8.2	1.76
0	CC025	F	Y	-7.83	-8.01	-8.77	-6.65	1.39
0	CC025	M	Y	-6.58	-4.37	-4.22	-5.14	0.52
1	CC025	M	Y	-2.71	-13.65	-4.08	-8.47	1.14
1	CC025	F	Y	-6.73	-16.79	-4.94	-12.77	1.43
1	CC025	F	Y	-3.98	-8.02	-3.73	-7.13	0.94
1	CC025	F	Y	-8.32	-9.33	-2.69	-6.5	1.22
2	CC025	F	Y	-6.93	-6.46	-2.58	-9.35	0.39
2	CC025	M	Y	-2.92	-22.67	-2.98	-7.93	0.75
0	CC027	F	Y	-6.94	-15.7	-10.31	-8.54	3.71
0	CC027	M	Y	-6.59	-10.86	-5.91	-6.13	1.14
1	CC027	M	Y	-12.22	-7.08	-16.76	-11.01	0.71
1	CC027	M	Y	-12.22	-7.08	-16.76	-11.01	0.71
1	CC027	F	Y	-9.6	-21.65	-10.25	-10.35	1.09
1	CC027	F	Y	-11.48	-9.21	-6.98	-8.14	0.48
1	CC027	M	Y	-5.37	-4.78	-2.53	-16.77	2.9

Trial#	Strain	Sex	Inf?	MINdA/dT_LF	MINdA/dT_LH	MINdA/dT_RF	MINdA/dT_RH	OverlapDistance_LF
1	CC027	M	Y	-3.95	-4.87	-2.55	-6.65	1.03
2	CC027	F	Y	-16.64	-6.78	-25.08	-25.89	1.35
2	CC027	M	Y	-8.11	-19.19	-5.1	-11.48	1.15
2	CC027	F	Y	-11	-13.35	-7.8	-8.51	0.97
2	CC027	M	Y	-6.82	-7.68	-7.46	-7.8	1.28
1	CC032XCC013	M	Y	-9.44	-10.17	-11	-10.93	1.39
1	CC032XCC013	M	Y	-9.53	-13.41	-10.35	-7.2	1.2
1	CC032XCC013	M	Y	-17.23	-14.32	-6.32	-11.64	0.92
1	CC032XCC013	F	Y	-6.99	-5.29	-2.72	-13.11	2.21
1	CC032XCC013	F	Y	-4.43	-11.84	-6.2	-9.1	2.32
1	CC032XCC013	F	Y	-8.2	-9.54	-6.58	-8.64	1.61
1	CC032XCC013	F	Y	-7.64	-10.64	-6.77	-8.82	1.77
1	CC032XCC013	F	Y	-3.73	-7.04	-3.3	-7.41	1.57
2	CC032XCC013	M	Y	-7.32	-6.94	-9.24	-8.25	1.66
2	CC032XCC013	M	Y	-15.49	-16.78	-19.59	-15.48	1.05
2	CC032XCC013	M	Y	-9.04	-11.45	-7.2	-14.79	2.35
2	CC032XCC013	M	Y	-7.85	-12.93	-13.1	-16.43	1.81
2	CC032XCC013	F	Y	-3.36	-8.11	-4.97	-7.79	1.19
2	CC032XCC013	F	Y	-8.64	-8.54	-12.51	-12.48	2.72
2	CC032XCC013	F	Y	-14.31	-17.24	-9.13	-16.49	1.37
2	CC032XCC013	F	Y	-6.46	-10.37	-6.68	-10.87	0.98
0	CC037	F	Y	-8.98	-10.68	-8.57	-7.14	1.01
0	CC037	M	Y	-10.17	-16.01	-13.78	-13.96	1.35
0	CC037	M	Y	-8.12	-13.55	-3.85	-8.58	1.12
0	CC037	M	Y	-1.96	-10.9	-7.42	-9.76	0.34
1	CC037	F	Y	-13.25	-13.47	-9.49	-9.71	1.05
1	CC037	M	Y	-7.26	-8.54	-4.65	-9.5	0.95
1	CC037	M	Y	-5.33	-9.84	-3.78	-19.67	1.7
1	CC037	M	Y	-3.37	-7.12	-3.19	-14.57	0.46
2	CC037	M	Y	-6.41	-8.49	-4.86	-9.54	1.32
2	CC037	M	Y	-3.47	-7.72	-2.25	-7.35	0.29
1	CC041XCC012	M	Y	-17.63	-7.19	-9.14	-12.38	2.81
1	CC041XCC012	M	Y	-12.9	-7.76	-2.61	-5.62	0.61
1	CC041XCC012	M	Y	-7.42	-12.41	-13.1	-12.97	1.22
1	CC041XCC012	M	Y	-15.3	-16.59	-22.04	-19.46	1.83
1	CC041XCC012	M	Y	-9.69	-10	-9.85	-13.1	1.13
1	CC041XCC012	F	Y	-16.3	-10.23	-12.48	-12.81	1.88
1	CC041XCC012	F	Y	-6.68	-20.58	-11.85	-17.05	0.33
1	CC041XCC012	F	Y	-7.47	-10.96	-6	-9.08	1.12
1	CC041XCC012	F	Y	-10.94	-10.25	-11.31	-5.7	0.57
1	CC041XCC012	F	Y	-8.83	-16.35	-19.92	-8.22	1.31
1	CC041XCC012	F	Y	-7.03	-9.31	-4.73	-10.07	2.26
1	CC041XCC012	F	Y	-6.14	-8.24	-6.31	-10.31	1.27
1	CC041XCC012	F	Y	-7.54	-21.6	-18.25	-17.62	0.51
2	CC041XCC012	M	Y	-5.78	-10.65	-6.8	-14.48	3.08
2	CC041XCC012	M	Y	-6.79	-13.38	-11.56	-13.05	0.63
2	CC041XCC012	F	Y	-9.07	-10.24	-9.77	-13.49	1.62
2	CC041XCC012	M	Y	-5.3	-10.52	-8.03	-15.7	1.94

Trial#	Strain	Sex	Inf?	MINdA/dT_LF	MINdA/dT_LH	MINdA/dT_RF	MINdA/dT_RH	OverlapDistance_LF
2	CC041XCC012	M	Y	-9.04	-12.66	-7.36	-15.18	1.3
2	CC041XCC012	M	Y	-7.4	-10.03	-9.25	-10.95	2.03
2	CC041XCC012	F	Y	-5.74	-7.93	-6.24	-8.9	0.64
2	CC041XCC012	F	Y	-11.66	-13.25	-6.47	-7.09	0.93
2	CC041XCC012	F	Y	-6.41	-9.51	-9.33	-10.35	0.61
2	CC041XCC012	F	Y	-4.76	-8.58	-6.44	-9.22	1.36
2	CC041XCC012	F	Y	-8.01	-8.87	-6.78	-9.09	1.72
2	CC041XCC012	F	Y	-4.48	-8.52	-5.24	-10.77	2.08
2	CC041XCC012	F	Y	-9.34	-11.24	-4.93	-10.43	0.36
0	CC051	F	Y	-4.07	-5.55	-5.03	-6.25	1.41
0	CC051	F	Y	-3.96	-5.84	-4.23	-6.96	1.21
0	CC051	F	Y	-3.16	-6.06	-3.04	-6.85	1.25
0	CC051	M	Y	-2.51	-4.6	-4.35	-6.61	0.81
1	CC051	F	Y	-3.61	-7.04	-3.12	-8.4	2.27
1	CC051	F	Y	-2.91	-8.74	-3.63	-8.27	1.53
1	CC051	F	Y	-2.39	-6.81	-2.11	-7.19	0.95
0	CC057	F	Y	-3.82	-13.84	-4.18	-12.8	1.74
0	CC057	F	Y	-3.57	-15.83	-6.93	-9.43	1.68
0	CC057	F	Y	-6.47	-6.88	-5.49	-4.82	1.25
0	CC057	M	Y	-11.61	-15.84	-10.87	-14.1	2.33
0	CC057	M	Y	-14.92	-10.45	-12.76	-12.03	1.1
0	CC057	M	Y	-14.8	-16	-14.4	-12.49	2
1	CC057	F	Y	-5.72	-13.05	-6.66	-11.39	1.99
1	CC057	F	Y	-4.97	-13.79	-16.19	-21.9	1.04
1	CC057	F	Y	-10.49	-13.23	-10.23	-15.7	1.82
1	CC057	F	Y	-14.24	-15.67	-11.32	-8.2	0.43
1	CC057	M	Y	-9.84	-9.27	-14.47	-11.25	1.89
1	CC057	M	Y	-6.26	-8.74	-6.01	-15.53	1.76
2	CC057	F	Y	-14.24	-15.67	-11.32	-8.2	0.43
0	CC078	F	Y	-6.44	-12.93	-5.87	-14.2	1.2
0	CC078	F	Y	-6.08	-14.06	-5.21	-12.88	1.32
0	CC078	F	Y	-6.3	-5.99	-6.81	-8.61	1.78
0	CC078	M	Y	-4.82	-7.07	-4.68	-8.5	0.87
1	CC078	F	Y	-8.34	-9.51	-6.79	-12.06	2.11
1	CC078	F	Y	-6.73	-9.62	-8.81	-11.03	0.89
1	CC078	M	Y	-6.29	-11.01	-6.03	-12.11	0.86
2	CC078	F	Y	-10.55	-11.6	-10.51	-11.21	1.89
2	CC078	M	Y	-11.3	-9.9	-11.73	-12.9	1.53
2	CC005	M	N	-8.65	-9.18	-4.82	-8.63	2.27
2	CC005	M	Y	-6.11	-8.7	-4.7	-9.88	1.23
2	CC015	F	N	-22.1	-38.33	-13.3	-26.92	1.51
2	CC015	M	N	-5.5	-11.66	-4.37	-12.76	1.15
2	CC015	M	Y	-7.12	-11.47	-6.48	-19.28	1.74
2	CC015	M	Y	-15.27	-17.29	-11.58	-20.88	1.22
2	CC017	F	N	-5	-11.34	-7.06	-13.54	1.37
2	CC017	M	N	-3.81	-11.69	-7.76	-23.1	0.54
2	CC017	F	Y	-9.6	-9.33	-7.57	-13.35	1.25
2	CC023	F	N	-9.53	-12.42	-4.57	-10.51	1.06

Trial#	Strain	Sex	Inf?	MINdA/dT_LF	MINdA/dT_LH	MINdA/dT_RF	MINdA/dT_RH	OverlapDistance_LF

Table S3. Raw DigiGait measurement data. Far left column indicates time point at which data was measured: T0 = pre-infection, T1 = 21dpi, and T3 = 89dpi. DigiGait parameters listed across the top indicate which limb is associated with the data, where appropriate: FL for left fore limb, FR for right fore limb, HL for left hind limb, and HR for right hind limb.

Table S3

Trial#	Strain	Sex	Inf?	OverlapDistance_LH	OverlapDistance_RF	OverlapDistance_RH	PawAngle_LF	PawAngle_LH
1	CC002	F	N	1.66	0.96	0.96	-9	-10.2
1	CC025	F	N	1.33	1.11	1.11	8.2	-15.9
1	CC012XCC032	F	N	2.26	1.6	1.6	-9.7	-26.5
2	CC012XCC032	F	N	0.77	0.45	0.45	-6.2	-17.3
1	CC012XCC032	M	N	1.92	1.5	1.5	-8.4	-16.3
2	CC012XCC032	M	N	0.55	1.5	1.5	-7.9	-10.8
1	CC013xCC041	F	N	2.27	0.74	0.74	-6.5	-10.5
2	CC013XCC041	F	N	2.56	2.06	2.06	-9.4	-8.6
1	CC013xCC041	M	N	1.69	1.9	1.9	-1.2	-12.3
2	CC013XCC041	M	N	2.65	1.86	1.86	-5.9	-12
1	CC032XCC013	F	N	1.55	3.21	3.21	5.8	-4.6
2	CC032XCC013	F	N	0.52	0.47	0.47	-0.7	-12.3
1	CC041XCC012	F	N	-0.14	-0.48	-0.48	-11.4	-6.8
1	CC041XCC012	F	N	2.21	0.75	0.75	-10.6	-0.8
2	CC041XCC012	F	N	1.23	1.39	1.39	-4.5	-8.9
1	CC032XCC013	M	N	0.94	1.8	1.8	-4.1	-8.6
2	CC032XCC013	M	N	-6.64	2.55	2.55	-33.1	-11.6
1	CC041XCC012	M	N	1.87	1.42	1.42	-18.6	-34.1
2	CC041XCC012	M	N	2.63	2.22	2.22	-15.3	-37.3
0	CC012	F	N	0.89	0.1	0.1	-21.6	-16.7
2	CC012	F	N	1.35	0.38	0.38	-2.3	-11.4
0	CC012	M	N	1.03	0.67	0.67	-10.8	-18.3
2	CC012	M	N	0.85	1.17	1.17	-19.3	-10.9
0	CC057	F	N	1.61	2.49	2.49	-5.8	-16
1	CC057	F	N	1.72	2.4	2.4	4.4	-13.2
0	CC057	M	N	2.07	2.01	2.01	-8.3	-19.1
1	CC057	M	N	1.85	1.9	1.9	-3.7	-12.1
0	CC078	F	N	1.3	0.87	0.87	-3.8	-24.5
1	CC078	F	N	1.09	1.15	1.15	-6.2	-17.3
2	CC078	F	N	1.14	1.62	1.62	-1.7	-12.8
0	CC078	M	N	1.35	0.75	0.75	8.7	-16.7
2	CC078	M	N	1.13	0.69	0.69	3.2	-6.4
0	CC002	F	N	1.41	2.82	2.82	3.6	-11
1	CC002	F	N	1.66	0.96	0.96	-9	-10.2
0	CC002	M	N	0.73	0.16	0.16	-12.2	-8.5
1	CC002	M	N	0.09	-0.45	-0.45	13.6	-9.5
2	CC002	M	N	2.14	0.43	0.43	-7.1	-3.5
0	CC006	F	N	0.8	1.54	1.54	1.5	-3.3
1	CC006	F	N	0.58	1.5	1.5	1.3	-15.8
2	CC006	F	N	1.22	0.86	0.86	-6.5	-20.2
0	CC006	M	N	1.07	1.12	1.12	-5.6	-1
0	CC023	F	N	1.4	1.98	1.98	0.6	-20.6
1	CC023	F	N	0.73	1.12	1.12	-7.9	-14
2	CC023	F	N	0.16	3	3	1.1	-8.3
0	CC023	M	N	1.09	0.62	0.62	7	-16.1
1	CC023	M	N	1.4	0.9	0.9	1.3	-16.8
0	CC027	F	N	1.63	1.01	1.01	-4.9	2.7

Trial#	Strain	Sex	Inf?	OverlapDistance_LH	OverlapDistance_RF	OverlapDistance_RH	PawAngle_LF	PawAngle_LH
1	CC027	F	N	1.14	1.2	1.2	-2.5	8.5
2	CC027	F	N	1.94	1.48	1.48	-1.5	-20.1
0	CC027	M	N	1.42	1.83	1.83	2.5	2.5
1	CC027	M	N	0.77	0.48	0.48	8.4	0.2
1	CC027	M	N	-0.41	0.52	0.52	-1.3	1.6
2	CC027	M	N	1.75	1.33	1.33	-3.3	-18.5
0	CC005	F	N	1.07	1.27	1.27	-25	-22.4
1	CC005	F	N	2.08	1.33	1.33	-20.6	-9.6
2	CC005	F	N	0.52	-0.57	-0.57	-0.7	3.4
0	CC011	F	N	0.6	0.96	0.96	-3.8	-26.5
1	CC011	F	N	1.39	2.56	2.56	0	1.1
2	CC011	F	N	2.08	1.96	1.96	-3.8	-3
1	CC017	F	N	2.01	1.44	1.44	1.9	-23.8
0	CC005	M	N	1.39	1.44	1.44	-11.8	-21.2
1	CC005	M	N	1.47	1.52	1.52	1.4	4.2
2	CC005	M	N	2.94	1.92	1.92	-4.5	-14.2
0	CC011	M	N	0.94	1.04	1.04	-8.5	-18.9
1	CC011	M	N	1.26	1.18	1.18	-11.1	-8.8
2	CC011	M	N	0.75	1.55	1.55	-15.5	-14.8
0	CC017	M	N	1.75	1.6	1.6	-2.3	-6
1	CC017	M	N	2.19	1.99	1.99	-4.6	-17.5
1	CC006	F	N	-0.11	1.01	1.01	10.5	-4.7
0	CC037	F	N	-0.96	-0.21	-0.21	-4.3	-26.5
0	CC051	F	N	-0.46	-0.45	-0.45	-8.4	-16.3
1	CC051	F	N	1.66	1.3	1.3	-5.9	-12.5
1	CC006	M	N	1.07	0.57	0.57	-8.6	-7.7
0	CC037	M	N	1.48	0.86	0.86	-14.3	-29.3
1	CC037	M	N	0.84	1.36	1.36	-9.5	-26.4
0	CC005	F	N	0.86	1.82	1.82	-18.2	-14.1
1	CC005	F	N	1.92	2.25	2.25	-26.3	-17
2	CC005	F	N	3.43	1.9	1.9	0.2	-24
0	CC011	F	N	0.96	1.02	1.02	-9.2	-18.7
1	CC011	F	N	3.47	4.17	4.17	-1.8	-15.5
2	CC011	F	N	1.78	0.93	0.93	-18.7	-12.8
0	CC011	M	N	1.76	2.29	2.29	-7.7	-14.3
1	CC011	M	N	3.31	3.2	3.2	-9.1	-17.7
2	CC011	M	N	1.06	1.4	1.4	-3.6	-8.5
1	CC037	M	N	1.95	2.84	2.84	5.7	-17.2
2	CC037	M	N	1.45	1.73	1.73	-2.9	-20.8
1	CC051	M	N	2.56	2.29	2.29	-16.2	-20.9
2	CC051	M	N	0.39	0.86	0.86	-23.1	-15.4
0	CC027	F	N	1.92	2.87	2.87	2.3	-25.9
1	CC027	F	N	1.99	1.76	1.76	-4.7	-23
2	CC027	F	N	0.7	1.49	1.49	-3.5	-10.4
0	CC015	M	N	-0.45	0.62	0.62	-0.9	-8.9
1	CC015	M	N	1.6	1.13	1.13	-8.4	-25.3
2	CC015	M	N	0.55	0.62	0.62	-7.3	-9
0	CC027	M	N	1.58	0.54	0.54	-12.3	-21.3

Trial#	Strain	Sex	Inf?	OverlapDistance_LH	OverlapDistance_RF	OverlapDistance_RH	PawAngle_LF	PawAngle_LH
1	CC027	M	N	2.6	2.35	2.35	-11.5	-16.6
2	CC027	M	N	2.07	1.59	1.59	-17.7	-20
0	CC015	F	N	0.75	0.75	-0.6	-5.5	5.8
1	CC015	F	N	0.96	0.72	0.72	-6.8	-11.5
0	CC017	F	N	1.15	0.66	0.66	1.6	-20.3
0	CC023	F	N	0.84	0.76	0.76	2.7	-9.6
1	CC023	F	N	1.37	1.33	1.33	5.9	-23.1
0	CC005	M	N	1.61	2.04	2.04	-10.3	-27.8
1	CC005	M	N	1.47	1.92	1.92	-20	-11.8
0	CC015	M	N	0.43	0.32	0.32	10	-4.1
1	CC015	M	N	1.01	0.66	0.66	-2.4	-12.2
0	CC017	M	N	1.44	1.89	1.89	9.5	-2.1
1	CC017	M	N	1.62	1.65	1.65	-1.1	-6.4
0	CC023	M	N	0.79	0.53	0.53	6.8	-20.7
1	CC023	M	N	1.14	1.3	1.3	-0.9	-20.3
0	CC051	M	N	1.22	1.41	1.41	-0.3	-3.6
0	CC002	F	Y	1.29	0.89	0.89	1.8	21.2
0	CC002	M	Y	0.85	1.11	1.11	-10.4	-8.9
1	CC002	M	Y	-0.98	1.04	1.04	-0.7	6.4
1	CC002	F	Y	2.17	1.07	1.07	15.1	49.5
1	CC002	F	Y	0.93	1.29	1.29	-0.7	-7.3
1	CC002	M	Y	-0.98	1.04	1.04	-0.7	6.4
1	CC002	M	Y	1.25	1.67	1.67	-1	-5.8
1	CC002	F	Y	2.17	1.07	1.07	15.1	49.5
1	CC002	F	Y	2.2	2.29	2.29	-4.2	-4.5
2	CC002	M	Y	2.53	1.51	1.51	-8.4	-15
2	CC002	F	Y	1.89	1.34	1.34	-13.6	-5.9
2	CC002	M	Y	0.82	-0.12	-0.12	-8.5	-11.2
0	CC005	F	Y	0.49	0.66	0.66	-12.9	-19
0	CC005	M	Y	1.27	1.18	1.18	-17	-17.5
0	CC005	M	Y	0.5	1.12	1.12	-1.7	5.8
1	CC005	F	Y	1.33	2.11	2.11	-14.2	-17
1	CC005	M	Y	4.05	2.24	2.24	1.7	34.6
1	CC005	F	Y	3.97	3.73	3.73	-15	-7.9
1	CC005	M	Y	2.76	2.25	2.25	-13.6	-24
2	CC005	F	Y	1.43	1.66	1.66	-9.1	-10.8
2	CC005	M	Y	2.78	1.89	1.89	0.8	-4.6
2	CC005	M	Y	0.84	1.61	1.61	-10.2	-13.7
2	CC005	M	Y	2.78	1.89	1.89	0.8	-4.6
2	CC005	F	Y	2.21	2.13	2.13	-6.2	-15.7
0	CC006	F	Y	0.86	1.02	1.02	2.4	-15.2
0	CC006	M	Y	1.32	1.35	1.35	-2.9	-33.9
0	CC006	F	Y	0.49	0.96	0.96	-4.7	-18.1
1	CC006	F	Y	0.96	1.1	1.1	8.9	-4.8
1	CC006	M	Y	1.21	1.41	1.41	-1.2	-2.9
1	CC006	F	Y	0.5	1.09	1.09	4.6	-1.9
1	CC006	M	Y	1.01	1.21	1.21	1.3	-21.6
2	CC006	F	Y	1.12	0.65	0.65	8.5	-8.9

Trial#	Strain	Sex	Inf?	OverlapDistance_LH	OverlapDistance_RF	OverlapDistance_RH	PawAngle_LF	PawAngle_LH
2	CC006	M	Y	-0.07	0.56	0.56	1.5	-9.7
0	CC011	F	Y	1.27	0.9	0.9	-2.4	0.4
0	CC011	M	Y	1.09	1.05	1.05	-5.9	-19.6
0	CC011	F	Y	1.42	1.01	1.01	-17.7	-23.7
0	CC011	F	Y	1	0.58	0.58	16.8	-18.4
1	CC011	F	Y	1.7	1.51	1.51	-18.3	-24.3
1	CC011	M	Y	2.26	1.43	1.43	-11.6	-19.5
1	CC011	F	Y	2.52	3.02	3.02	-26.1	-13.7
1	CC011	M	Y	2.58	1.89	1.89	-23.9	-19.2
2	CC011	F	Y	1.77	1.56	1.56	-9.6	-20.3
2	CC011	M	Y	1.96	2.39	2.39	1.6	-22.6
2	CC011	F	Y	0.75	0.96	0.96	-21.2	-16.4
2	CC011	M	Y	1.09	1.43	1.43	-12	-15.7
0	CC012	M	Y	1.06	1.74	1.74	-1.7	-11.1
0	CC012	M	Y	0	1.47	1.47	-6.1	-23.2
0	CC012	M	Y	1.15	1.37	1.37	-2.4	-21.5
0	CC012	M	Y	0.82	0.7	0.7	-2.2	-33.4
0	CC012	M	Y	0.7	0.67	0.67	-1.7	-21.2
0	CC012	F	Y	1.76	1.25	1.25	-2.1	-7.6
0	CC012	F	Y	1.24	1.11	1.11	9.3	-19.8
0	CC012	F	Y	0.42	1.48	1.48	-11.2	-22.9
0	CC012	F	Y	0.8	1.86	1.86	0	-11.7
0	CC012	F	Y	0.74	1.24	1.24	-12.2	-20.8
2	CC012	M	Y	0.65	1.2	1.2	4.7	16.5
2	CC012	M	Y	0.86	0.73	0.73	0.3	-9.9
2	CC012	M	Y	0.82	0.38	0.38	16.8	-6.9
2	CC012	M	Y	-0.93	-4.8	-4.8	4.1	-9.1
2	CC012	M	Y	1.06	0.84	0.84	-8.5	-11.2
2	CC012	F	Y	2.41	0.69	0.69	-1.9	-8.7
2	CC012	F	Y	1.15	1.52	1.52	-4.8	-19
2	CC012	F	Y	2.83	1.65	1.65	-0.5	-20.5
2	CC012	F	Y	0.32	2.42	2.42	6.8	21.7
2	CC012	F	Y	0.76	-1.68	-1.68	4.7	-25
1	CC012XCC032	F	Y	-0.74	0.74	0.74	-10.3	-1.3
1	CC012xCC032	F	Y	1.1	1.82	1.82	1.1	-2.3
1	CC012XCC032	M	Y	0.51	0.81	0.81	7.3	-15.4
1	CC012xCC032	M	Y	-0.53	0.53	0.53	-1.3	-6.6
1	CC012XCC032	M	Y	1.5	-1.23	-1.23	-1.3	-17.8
1	CC012xCC032	M	Y	1.41	0.93	0.93	15.4	-9.6
2	CC012XCC032	F	Y	2.29	2.95	2.95	-1.9	-12.2
2	CC012XCC032	F	Y	1.05	0.12	0.12	-2.2	-7.4
2	CC012XCC032	M	Y	1.46	0.91	0.91	-7.8	-15.9
2	CC012XCC032	M	Y	1.25	2.4	2.4	-16.4	-16.8
2	CC012XCC032	M	Y	1.35	0.15	0.15	2.1	-12.4
2	CC012XCC032	M	Y	0.06	-0.78	-0.78	-5.5	-14.2
1	CC013xCC041	F	Y	1.5	0.96	0.96	-2.2	-12.5
1	CC013xCC041	F	Y	1.96	1.35	1.35	1.4	-5.3
1	CC013xCC041	F	Y	1.9	2.42	2.42	-1.5	7.8

Trial#	Strain	Sex	Inf?	OverlapDistance_LH	OverlapDistance_RF	OverlapDistance_RH	PawAngle_LF	PawAngle_LH
1	CC013xCC041	M	Y	0.88	0.97	0.97	-4.7	-15.6
1	CC013xCC041	M	Y	2.7	2.31	2.31	2	-7.7
2	CC013XCC041	F	Y	1.76	1.85	1.85	-3	-2.4
2	CC013XCC041	F	Y	1.8	0.18	0.18	-1.4	-5
2	CC013XCC041	F	Y	2.15	1.93	1.93	-1.3	-0.1
2	CC013XCC041	M	Y	1.58	2.61	2.61	-3.3	-5.6
2	CC013XCC041	M	Y	1.77	2.7	2.7	1.7	-9
0	CC015	F	Y	0.23	0.19	0.19	16.7	4.9
0	CC015	M	Y	1.59	1.11	1.11	2	-7.8
0	CC015	M	Y	0.65	0.98	0.98	7.2	-9
1	CC015	F	Y	0.69	1.38	1.38	4.2	-4.5
1	CC015	M	Y	1.4	0.33	0.33	-5.3	-7.8
1	CC015	M	Y	2.21	1.31	1.31	2.5	-5.4
1	CC015	M	Y	1.26	1.02	1.02	-13.5	-24.8
2	CC015	F	Y	1.44	0.8	0.8	1.5	2
0	CC017	F	Y	1.33	0.41	0.41	-11.1	-11.4
0	CC017	M	Y	1.44	1.48	1.48	-7.9	-5.2
0	CC017	F	Y	1	0.48	0.48	7.4	-3.8
0	CC017	M	Y	0.49	0.51	0.51	4.8	-6.5
1	CC017	M	Y	1.35	1.56	1.56	-9.2	-15.7
1	CC017	F	Y	0.51	-0.21	-0.21	-18.4	-8.9
0	CC023	M	Y	1.56	1.31	1.31	3.5	-17
0	CC023	F	Y	0.67	1.22	1.22	-3.6	-18.9
0	CC023	F	Y	2.97	0.82	0.82	-1.8	-17.6
0	CC023	M	Y	1.72	4.27	4.27	-14.6	-28.3
1	CC023	M	Y	2.09	2.1	2.1	8.9	3
1	CC023	F	Y	0.53	1.22	1.22	-14.8	-4
1	CC023	M	Y	0.98	4.45	4.45	-13.6	-18.7
1	CC023	M	Y	4.07	5.55	5.55	-16.8	-16.7
2	CC023	M	Y	2.14	1.46	1.46	-5.6	-7.4
2	CC023	F	Y	1.35	0.43	0.43	7.1	-3.8
2	CC023	M	Y	0.76	2.55	2.55	-27.5	-16.2
0	CC025	M	Y	1.76	1.48	1.48	-5.1	-16.7
0	CC025	F	Y	1.39	1.13	1.13	-3.2	-12.3
0	CC025	M	Y	0.52	1.3	1.3	-4.4	-29.4
1	CC025	M	Y	1.14	1.66	1.66	-5.4	-18.8
1	CC025	F	Y	1.43	1.27	1.27	0.3	-13.7
1	CC025	F	Y	0.94	1.98	1.98	-1.5	-19.1
1	CC025	F	Y	1.22	1.54	1.54	3	1.4
2	CC025	F	Y	0.39	0.2	0.2	0.6	-6.3
2	CC025	M	Y	0.75	1.31	1.31	-13.9	-12.3
0	CC027	F	Y	3.71	1.34	1.34	-0.6	-20.8
0	CC027	M	Y	1.14	2.43	2.43	-11.4	-30.7
1	CC027	M	Y	0.71	1.83	1.83	-33.4	11.1
1	CC027	M	Y	0.71	1.83	1.83	-33.4	11.1
1	CC027	F	Y	1.09	1.03	1.03	14.3	-13.7
1	CC027	F	Y	0.48	1.11	1.11	-19.9	-30.6
1	CC027	M	Y	2.9	2.78	2.78	-2.9	-22.5

Trial#	Strain	Sex	Inf?	OverlapDistance_LH	OverlapDistance_RF	OverlapDistance_RH	PawAngle_LF	PawAngle_LH
1	CC027	M	Y	1.03	1.38	1.38	-1.4	-20.7
2	CC027	F	Y	1.35	0.43	0.43	7.1	-3.8
2	CC027	M	Y	1.15	0.85	0.85	-10.1	-18.8
2	CC027	F	Y	0.97	1.53	1.53	-8.9	-24.8
2	CC027	M	Y	1.28	1.7	1.7	3.5	-14.8
1	CC032XCC013	M	Y	1.39	1.74	1.74	-0.3	-11.8
1	CC032XCC013	M	Y	1.2	2	2	-6.8	-8.5
1	CC032XCC013	M	Y	0.92	1.57	1.57	-10	-8.5
1	CC032XCC013	F	Y	2.21	-2.53	-2.53	-21.2	-15.7
1	CC032XCC013	F	Y	2.32	0.92	0.92	-5.4	-7.1
1	CC032XCC013	F	Y	1.61	1.94	1.94	-11.8	-9.5
1	CC032XCC013	F	Y	1.77	1.93	1.93	-12.1	-10
1	CC032XCC013	F	Y	1.57	2.48	2.48	-18	-8.8
2	CC032XCC013	M	Y	1.66	2.21	2.21	-3.8	-18.6
2	CC032XCC013	M	Y	1.05	1.58	1.58	6.1	-8.8
2	CC032XCC013	M	Y	2.35	2.11	2.11	-12	-15.9
2	CC032XCC013	M	Y	1.81	1.14	1.14	-13.2	-14.8
2	CC032XCC013	F	Y	1.19	1.03	1.03	-10.9	-7
2	CC032XCC013	F	Y	2.72	1.89	1.89	-14	-11
2	CC032XCC013	F	Y	1.37	2.13	2.13	-1.2	-11.8
2	CC032XCC013	F	Y	0.98	1.27	1.27	-1.5	-12.4
0	CC037	F	Y	1.01	1.22	1.22	-5.5	-18.1
0	CC037	M	Y	1.35	1.37	1.37	-1.6	-23.3
0	CC037	M	Y	1.12	1.3	1.3	-0.6	-9.8
0	CC037	M	Y	0.34	1.91	1.91	16.4	6
1	CC037	F	Y	1.05	1.21	1.21	-1.2	-14.6
1	CC037	M	Y	0.95	1.28	1.28	-12.5	-17
1	CC037	M	Y	1.7	2.1	2.1	-6.6	-19.7
1	CC037	M	Y	0.46	0.81	0.81	3.4	-22.6
2	CC037	M	Y	1.32	2.84	2.84	-7.5	-15.4
2	CC037	M	Y	0.29	0.62	0.62	-4.4	-22.9
1	CC041XCC012	M	Y	0.61	0.05	0.05	-3.7	-6.5
1	CC041XCC012	M	Y	1.22	1.48	1.48	-4.3	-8.4
1	CC041XCC012	M	Y	1.83	2.01	2.01	-28.2	-11.9
1	CC041XCC012	M	Y	1.13	0.2	0.2	-12.3	-6.9
1	CC041XCC012	M	Y	1.12	0.62	0.62	-8.8	-21.2
1	CC041XCC012	F	Y	1.88	2.11	2.11	-2.5	-13.5
1	CC041XCC012	F	Y	0.33	1.23	1.23	5.8	-10.4
1	CC041XCC012	F	Y	0.57	2.99	2.99	-9.5	-17.8
1	CC041XCC012	F	Y	1.31	0.56	0.56	-11.4	-8.6
1	CC041XCC012	F	Y	2.26	2.33	2.33	-2.2	-11.8
1	CC041XCC012	F	Y	1.27	1.91	1.91	-4.5	-13.9
1	CC041XCC012	F	Y	0.51	0.85	0.85	-2.5	-10.8
1	CC041XCC012	F	Y	2.81	0.84	0.84	-3.4	-10.7
2	CC041XCC012	M	Y	3.08	-0.64	-0.64	-4.6	-12.1
2	CC041XCC012	M	Y	0.63	0.48	0.48	5.4	0.9
2	CC041XCC012	F	Y	1.62	1.85	1.85	2.9	-12.4
2	CC041XCC012	M	Y	1.94	1.35	1.35	-21.6	4.4

Trial#	Strain	Sex	Inf?	OverlapDistance_LH	OverlapDistance_RF	OverlapDistance_RH	PawAngle_LF	PawAngle_LH
2	CC041XCC012	M	Y	1.3	1.61	1.61	-11	-5.1
2	CC041XCC012	M	Y	2.03	1.32	1.32	-17.1	-7.8
2	CC041XCC012	F	Y	0.64	0.8	0.8	-5.9	-6.8
2	CC041XCC012	F	Y	0.93	1.37	1.37	2	2.1
2	CC041XCC012	F	Y	0.61	1.8	1.8	-8	-18.4
2	CC041XCC012	F	Y	1.36	1.32	1.32	-0.1	-14.9
2	CC041XCC012	F	Y	1.72	1.2	1.2	-11.7	-9.1
2	CC041XCC012	F	Y	2.08	1.59	1.59	2	-5.4
2	CC041XCC012	F	Y	0.36	0.69	0.69	3.5	6.5
0	CC051	F	Y	1.41	1.39	1.39	-12.3	-28.1
0	CC051	F	Y	1.21	0.72	0.72	-12.9	-25.3
0	CC051	F	Y	1.25	1.07	1.07	-4.3	-19.6
0	CC051	M	Y	0.81	1.15	1.15	-11.7	-23.9
1	CC051	F	Y	2.27	1.44	1.44	-9.9	-21.5
1	CC051	F	Y	1.53	1.95	1.95	-2.9	-14.2
1	CC051	F	Y	0.95	1.52	1.52	-5.1	-14.7
0	CC057	F	Y	1.74	1.97	1.97	-11.7	-16.2
0	CC057	F	Y	1.68	2.12	2.12	-18.2	-28.9
0	CC057	F	Y	1.25	2.09	2.09	-7	-10.5
0	CC057	M	Y	2.33	0.92	0.92	-14.3	-20.4
0	CC057	M	Y	1.1	1.72	1.72	2.1	-7.5
0	CC057	M	Y	2	0.94	0.94	-10.3	-22.3
1	CC057	F	Y	1.99	1.96	1.96	-14.5	-13.6
1	CC057	F	Y	1.04	1.43	1.43	-13.2	-21.6
1	CC057	F	Y	1.82	0.45	0.45	-9.9	-13.3
1	CC057	F	Y	0.43	1.78	1.78	-8.3	-12.9
1	CC057	M	Y	1.89	1.98	1.98	-13.9	-16.9
1	CC057	M	Y	1.76	1.77	1.77	-15.2	-12.3
2	CC057	F	Y	0.43	1.78	1.78	-8.3	-12.9
0	CC078	F	Y	1.2	1.06	1.06	4.6	-15.6
0	CC078	F	Y	1.32	1.7	1.7	-0.1	-5.6
0	CC078	F	Y	1.78	1.28	1.28	3.9	-18.5
0	CC078	M	Y	0.87	0.52	0.52	1.1	-24.7
1	CC078	F	Y	2.11	1.17	1.17	-7	-17.1
1	CC078	F	Y	0.89	1.23	1.23	19.5	-13.5
1	CC078	M	Y	0.86	0.8	0.8	7.4	-11.9
2	CC078	F	Y	1.89	0.99	0.99	2.9	-2
2	CC078	M	Y	1.53	0.72	0.72	-9.6	-2.7
2	CC005	M	N	2.27	2.67	2.67	-7.4	-10.2
2	CC005	M	Y	1.23	0.87	0.87	-1.7	-10.6
2	CC015	F	N	1.51	2.37	2.37	13.3	0.7
2	CC015	M	N	1.15	0.98	0.98	-2.2	-10.2
2	CC015	M	Y	1.74	0.89	0.89	-8.4	-12
2	CC015	M	Y	1.22	1.43	1.43	-0.2	1.7
2	CC017	F	N	1.37	2.85	2.85	3.8	-12
2	CC017	M	N	0.54	1.33	1.33	0.5	-9.4
2	CC017	F	Y	1.25	-0.46	-0.46	-5.7	-8.9
2	CC023	F	N	1.06	0.87	0.87	0.1	-21.1

Trial#	Strain	Sex	Inf?	OverlapDistance_LH	OverlapDistance_RF	OverlapDistance_RH	PawAngle_LF	PawAngle_LH

Table S3. Raw DigiGait measurement data. Far left column indicates time point at which data was measured: T0 = pre-infection, T1 = 21dpi, and T3 = 89dpi. DigiGait parameters listed across the top indicate which limb is associated with the data, where appropriate: FL for left fore limb, FR for right fore limb, HL for left hind limb, and HR for right hind limb.

Table S3

Trial#	Strain	Sex	Inf?	PawAngle_RF	PawAngle_RH	PawAngleVariability_LF	PawAngleVariability_LH
1	CC002	F	N	12.8	17.5	14.6	29
1	CC025	F	N	1.7	11.4	8.9	4.6
1	CC012XCC032	F	N	-4.5	28.4	8.8	7.8
2	CC012XCC032	F	N	-8.9	10.9	10	4
1	CC012XCC032	M	N	7.9	18.9	13.8	3.2
2	CC012XCC032	M	N	3.8	16.8	5	1.5
1	CC013xCC041	F	N	1.3	5.9	6.6	9.9
2	CC013XCC041	F	N	11.3	16	5.8	5.7
1	CC013xCC041	M	N	26.7	5.9	25.2	40.9
2	CC013XCC041	M	N	-0.7	4.1	8.6	9.7
1	CC032XCC013	F	N	6.6	18.2	27.8	27.1
2	CC032XCC013	F	N	-7.1	7.8	11.2	6.5
1	CC041XCC012	F	N	5.1	14.5	9.7	6.7
1	CC041XCC012	F	N	3.6	12.9	15.2	12.9
2	CC041XCC012	F	N	25	20.7	25	3.4
1	CC032XCC013	M	N	10.1	11.3	20.3	8.5
2	CC032XCC013	M	N	22.5	22.8	46.2	4.5
1	CC041XCC012	M	N	20.5	26	13.2	7.2
2	CC041XCC012	M	N	14.1	21	14	12.7
0	CC012	F	N	5.9	24.1	13.6	11.1
2	CC012	F	N	25.9	19.9	7	11.4
0	CC012	M	N	17.9	27.6	25.2	20.1
2	CC012	M	N	1.3	22.3	11.2	4
0	CC057	F	N	12	19.1	4.7	8.3
1	CC057	F	N	3.3	20.8	5	4.2
0	CC057	M	N	8.6	21.2	20.8	12.3
1	CC057	M	N	11.6	22.8	5.1	4.3
0	CC078	F	N	3.7	12.9	3.8	1.9
1	CC078	F	N	9.8	13.8	2.6	4.2
2	CC078	F	N	-1.3	18.3	10.2	11.8
0	CC078	M	N	17.2	10.9	4.7	3.9
2	CC078	M	N	-10.1	7.3	8.7	8.8
0	CC002	F	N	4.6	10.3	11.1	14.5
1	CC002	F	N	12.8	17.5	14.6	29
0	CC002	M	N	1.5	5.8	7.7	13.3
1	CC002	M	N	10.5	5.6	13.8	26.2
2	CC002	M	N	4.5	8.2	8	4.7
0	CC006	F	N	-4.4	16.8	5.3	7.3
1	CC006	F	N	-4.7	15.4	14.8	11.5
2	CC006	F	N	-0.6	13	19.9	13.2
0	CC006	M	N	5.9	10.1	8.1	30.7
0	CC023	F	N	2.3	15.5	15.9	12.8
1	CC023	F	N	-1.1	6.5	21.7	9.2
2	CC023	F	N	-4.9	4.9	5.7	10.4
0	CC023	M	N	-7.5	8.9	3.5	3.1
1	CC023	M	N	2.8	13.3	14.8	5.3
0	CC027	F	N	-10	-9.3	15.5	21.5

Trial#	Strain	Sex	Inf?	PawAngle_RF	PawAngle_RH	PawAngleVariability_LF	PawAngleVariability_LH
1	CC027	F	N	5.9	17.6	5.7	27.4
2	CC027	F	N	5.6	13.2	7.2	4.4
0	CC027	M	N	5.6	5.2	13.7	18.8
1	CC027	M	N	-2.1	-4.1	20.3	19.6
1	CC027	M	N	-1.2	1.6	4.6	24.1
2	CC027	M	N	-3.7	11.1	3.4	4
0	CC005	F	N	13.3	13.4	16.3	4.8
1	CC005	F	N	-31.4	4.4	44.3	10
2	CC005	F	N	-16.7	-14.6	35.6	33.1
0	CC011	F	N	0.5	9	11.6	18.9
1	CC011	F	N	4.7	12.4	11.4	6.5
2	CC011	F	N	5.3	8	5	5.8
1	CC017	F	N	12.6	22.5	15.1	14.2
0	CC005	M	N	18	14.5	5.6	7.3
1	CC005	M	N	17.9	-2.8	22.5	23.8
2	CC005	M	N	17.5	20.8	25.7	36.9
0	CC011	M	N	10.7	16.8	7	6.3
1	CC011	M	N	0.9	12.5	5.8	2.3
2	CC011	M	N	19.9	19.8	29.3	11.1
0	CC017	M	N	12.2	6.4	34.7	30.1
1	CC017	M	N	10.9	17.3	13.2	9.4
1	CC006	F	N	-7.8	2.6	24.5	23.4
0	CC037	F	N	0.8	29.3	17.6	5.8
0	CC051	F	N	-2.3	15.1	8.6	5.1
1	CC051	F	N	-8.5	15.4	2.4	6.1
1	CC006	M	N	0.8	8.6	6.5	12.7
0	CC037	M	N	13.9	23.3	15.1	4.9
1	CC037	M	N	1.8	7.7	4.4	5.8
0	CC005	F	N	1.7	9.8	5.2	3.5
1	CC005	F	N	21.4	13.2	5.9	4.4
2	CC005	F	N	22	13.4	6.3	13.6
0	CC011	F	N	4.5	17.9	7.1	12.2
1	CC011	F	N	-4.5	9.8	2.6	2.3
2	CC011	F	N	18.3	8.8	3.5	2.7
0	CC011	M	N	3.8	18.2	5.4	7.6
1	CC011	M	N	9.6	20.6	5.5	5.2
2	CC011	M	N	12.2	24.2	4.8	4
1	CC037	M	N	-10.5	12.8	13.1	5.6
2	CC037	M	N	7.8	12.7	13.4	9.4
1	CC051	M	N	11.1	22.1	5.3	4.5
2	CC051	M	N	8.5	10.9	3.9	5.2
0	CC027	F	N	-3	19.3	5.3	5.2
1	CC027	F	N	4.8	16.9	10	7.3
2	CC027	F	N	2.8	20.5	8.8	7.3
0	CC015	M	N	9.9	23	10.4	6.5
1	CC015	M	N	3.8	-2.4	19	10.7
2	CC015	M	N	20	18.1	6	7.5
0	CC027	M	N	2.8	14.5	8	7.4

Trial#	Strain	Sex	Inf?	PawAngle_RF	PawAngle_RH	PawAngleVariability_LF	PawAngleVariability_LH
1	CC027	M	N	15	13.1	14	3.6
2	CC027	M	N	13	4.9	8.2	6.7
0	CC015	F	N	14.3	4.1	8.8	17.9
1	CC015	F	N	1.7	7	15.4	11.7
0	CC017	F	N	-5.2	12.7	6.1	2.9
0	CC023	F	N	0.2	9.3	14.1	11.5
1	CC023	F	N	-1.8	20.4	9.5	7.6
0	CC005	M	N	-5	17.4	3.4	8.7
1	CC005	M	N	8.3	11.3	6.4	5.2
0	CC015	M	N	-10.8	11.2	6	14
1	CC015	M	N	1.9	5.3	17	12.2
0	CC017	M	N	-8.5	13.1	14.4	18.3
1	CC017	M	N	5.7	15.6	16.8	10.3
0	CC023	M	N	-4.3	14.3	3.3	2.3
1	CC023	M	N	5.9	17.7	13.9	9.7
0	CC051	M	N	-5	19.4	4.9	7.8
0	CC002	F	Y	1.6	1.9	19.7	26.9
0	CC002	M	Y	-1.1	0.1	13.2	5.2
1	CC002	M	Y	21.3	26.2	13.6	23.3
1	CC002	F	Y	21.3	24.5	26.2	18.4
1	CC002	F	Y	4.6	16.2	20	14.3
1	CC002	M	Y	21.3	26.2	13.6	23.3
1	CC002	M	Y	5	29.1	12.1	18.5
1	CC002	F	Y	21.3	24.5	26.2	18.4
1	CC002	F	Y	0.4	-2.4	21.3	24.7
2	CC002	M	Y	12	8.3	9.1	20.1
2	CC002	F	Y	-10.4	7.5	6.1	2.6
2	CC002	M	Y	8.9	13.1	14.8	10.4
0	CC005	F	Y	6.9	6.3	5.9	5.9
0	CC005	M	Y	3.5	13.4	12.3	4.8
0	CC005	M	Y	3.5	18.4	9	12.7
1	CC005	F	Y	17.5	-4.1	11.1	7.6
1	CC005	M	Y	22.6	34.6	11.1	36.2
1	CC005	F	Y	27.4	10.3	4	3.1
1	CC005	M	Y	2	8.7	15.9	17.6
2	CC005	F	Y	28	12.9	18.5	7
2	CC005	M	Y	4.6	17.5	4.9	4.3
2	CC005	M	Y	15	6.9	5.6	9.2
2	CC005	M	Y	4.6	17.5	4.9	4.3
2	CC005	F	Y	20.4	3	11.8	8.1
0	CC006	F	Y	-1.5	17	9.7	7.3
0	CC006	M	Y	-6.5	-2.2	7.7	16.6
0	CC006	F	Y	3.7	18.9	10.3	15.5
1	CC006	F	Y	3.9	3.8	19	24.7
1	CC006	M	Y	4.2	1.3	24.5	39
1	CC006	F	Y	-7	11.6	8.2	11.9
1	CC006	M	Y	-1.9	9.8	22.6	5.6
2	CC006	F	Y	0.4	17.2	19.8	7

Trial#	Strain	Sex	Inf?	PawAngle_RF	PawAngle_RH	PawAngleVariability_LF	PawAngleVariability_LH
2	CC006	M	Y	2.5	12	22.1	16.2
0	CC011	F	Y	-11.1	3.9	16.8	10
0	CC011	M	Y	7.5	18.2	14.6	18.1
0	CC011	F	Y	12	27.2	1.7	3.7
0	CC011	F	Y	1.3	26.4	6.8	20.5
1	CC011	F	Y	14.7	24.6	3.2	1.9
1	CC011	M	Y	15.3	17.6	8.8	6.5
1	CC011	F	Y	2.9	20.3	4.8	3.5
1	CC011	M	Y	19.9	16.1	5.2	4.5
2	CC011	F	Y	16.1	20.1	2.1	3.6
2	CC011	M	Y	14.9	12.9	7.4	5.4
2	CC011	F	Y	20.1	18.7	1.5	3.1
2	CC011	M	Y	16.5	13.8	13.9	3.5
0	CC012	M	Y	3.4	23.3	7	7.5
0	CC012	M	Y	-10.6	18.3	12.5	5.3
0	CC012	M	Y	7.1	27.1	7.7	4.3
0	CC012	M	Y	-0.2	9	23.5	15.5
0	CC012	M	Y	-8.1	10.3	9.7	3.5
0	CC012	F	Y	-3.4	12.7	14.5	6
0	CC012	F	Y	-9.4	24.5	7.5	3.6
0	CC012	F	Y	-1.8	24.6	16.7	18.2
0	CC012	F	Y	-0.5	25.3	27.6	33.1
0	CC012	F	Y	-4.1	6	10.2	3.1
2	CC012	M	Y	1.2	13.3	6.9	18.5
2	CC012	M	Y	15.9	24.5	7.4	2.9
2	CC012	M	Y	11.8	16.2	6.7	10.7
2	CC012	M	Y	5	12.9	5	14.8
2	CC012	M	Y	3.3	13.6	6.9	10.1
2	CC012	F	Y	-5.4	23.1	10.7	13.3
2	CC012	F	Y	1.8	31.8	8.4	4.1
2	CC012	F	Y	-2.4	14.5	10.5	6.3
2	CC012	F	Y	17.8	26.8	9.3	14.1
2	CC012	F	Y	-6.5	21.8	7.1	7.4
1	CC012XCC032	F	Y	9.8	15.3	22.2	22.6
1	CC012xCC032	F	Y	10.1	5.2	16.7	26.3
1	CC012XCC032	M	Y	1.7	17.4	13.7	5.6
1	CC012xCC032	M	Y	3.1	21.1	13.4	15.5
1	CC012XCC032	M	Y	0.4	-8.6	24.7	17.8
1	CC012xCC032	M	Y	2.7	16.2	19.7	25.1
2	CC012XCC032	F	Y	12.1	23.7	9.1	2.4
2	CC012XCC032	F	Y	3.7	9.3	3.5	2.7
2	CC012XCC032	M	Y	-6.5	12.2	15.8	2.4
2	CC012XCC032	M	Y	6.4	36.1	15.2	7
2	CC012XCC032	M	Y	-14.1	13	5.1	3.9
2	CC012XCC032	M	Y	-9.4	12.6	10.1	13.7
1	CC013xCC041	F	Y	0.1	6.3	7.2	9.8
1	CC013xCC041	F	Y	-0.1	5.2	16.7	10.2
1	CC013xCC041	F	Y	-3.2	13.5	11.1	20.2

Trial#	Strain	Sex	Inf?	PawAngle_RF	PawAngle_RH	PawAngleVariability_LF	PawAngleVariability_LH
1	CC013xCC041	M	Y	-8.9	-4.9	5.2	17.4
1	CC013xCC041	M	Y	-5	2.5	14.1	5.4
2	CC013XCC041	F	Y	6.1	1.7	3.2	1.8
2	CC013XCC041	F	Y	-3.6	0	3.5	6.8
2	CC013XCC041	F	Y	0.9	2.2	4	6.6
2	CC013XCC041	M	Y	2	2.6	7.1	7.5
2	CC013XCC041	M	Y	-1.9	6.4	16.9	6.9
0	CC015	F	Y	1.7	15.5	10.8	11.7
0	CC015	M	Y	-6	6.2	3.8	5.5
0	CC015	M	Y	-0.1	8.5	3.2	4.1
1	CC015	F	Y	8.3	13.7	6.2	5.1
1	CC015	M	Y	-0.5	8.4	15.6	11.5
1	CC015	M	Y	-2.5	11.6	23.1	10.6
1	CC015	M	Y	12.8	18.5	13.2	8.9
2	CC015	F	Y	2.5	11.4	4.3	2.8
0	CC017	F	Y	0.3	15.1	13.2	14.3
0	CC017	M	Y	-18.1	7.3	7.7	7
0	CC017	F	Y	-11.9	-1.3	28	17.4
0	CC017	M	Y	1.9	6	13.3	14.2
1	CC017	M	Y	-1.1	15.1	17.5	8.6
1	CC017	F	Y	13	14.5	29.4	15.1
0	CC023	M	Y	-6.8	13.7	3.6	6.1
0	CC023	F	Y	-2.7	4.6	10.2	6.6
0	CC023	F	Y	0	19.1	11	9.5
0	CC023	M	Y	-1.2	5.8	8.2	5.1
1	CC023	M	Y	-4.8	4.7	14.2	13.3
1	CC023	F	Y	-0.1	7	16.3	20.2
1	CC023	M	Y	-1.1	15.1	6	9.8
1	CC023	M	Y	-9.7	17.5	7.3	9
2	CC023	M	Y	-13.1	7	3	15.1
2	CC023	F	Y	16.4	15	9.9	7.6
2	CC023	M	Y	-6	1.3	18.4	2.9
0	CC025	M	Y	7.1	21.4	6.8	6
0	CC025	F	Y	-17.9	8.7	16.2	10.2
0	CC025	M	Y	9.1	19.5	2.5	3.4
1	CC025	M	Y	9.1	20.9	6.7	12.1
1	CC025	F	Y	9.8	21.8	7.6	7.9
1	CC025	F	Y	2.2	17.6	7.4	8.2
1	CC025	F	Y	2.1	10	14.2	6.6
2	CC025	F	Y	5.2	10.2	5.9	5.3
2	CC025	M	Y	11.7	22.9	4.4	5.2
0	CC027	F	Y	-2.1	9.8	17.3	7.2
0	CC027	M	Y	8.3	23.9	16.5	7.1
1	CC027	M	Y	-14.8	19.5	3.9	23
1	CC027	M	Y	-14.8	19.5	3.9	23
1	CC027	F	Y	11.3	10.3	28.3	25.4
1	CC027	F	Y	-7.1	11.5	16.6	7.3
1	CC027	M	Y	12.9	10.6	12.5	7.1

Trial#	Strain	Sex	Inf?	PawAngle_RF	PawAngle_RH	PawAngleVariability_LF	PawAngleVariability_LH
1	CC027	M	Y	13.6	23.6	2.1	2.4
2	CC027	F	Y	16.4	15	9.9	7.6
2	CC027	M	Y	2.4	23.8	13.2	7.1
2	CC027	F	Y	3.6	15	15	9.8
2	CC027	M	Y	9.7	12.2	5.5	5.9
1	CC032XCC013	M	Y	14.5	23.6	6.1	6.9
1	CC032XCC013	M	Y	-0.4	24.8	3.6	3.3
1	CC032XCC013	M	Y	2.5	21.7	20.9	5.2
1	CC032XCC013	F	Y	4.3	14.9	14.9	15.3
1	CC032XCC013	F	Y	12.5	15.9	5.1	7.4
1	CC032XCC013	F	Y	-8	20.2	5.1	4.9
1	CC032XCC013	F	Y	-7.5	20	1.2	5.1
1	CC032XCC013	F	Y	14.7	30.3	3.9	13.8
2	CC032XCC013	M	Y	3.2	18.2	5.2	6
2	CC032XCC013	M	Y	17.5	19.2	17.1	3.5
2	CC032XCC013	M	Y	9.2	22.3	16.1	12.7
2	CC032XCC013	M	Y	8.4	7.6	11.7	4.1
2	CC032XCC013	F	Y	11.5	15	7.9	3
2	CC032XCC013	F	Y	-6	11.1	4.5	3.9
2	CC032XCC013	F	Y	1.3	12.5	4.6	2.7
2	CC032XCC013	F	Y	3.4	9.6	9.6	1.4
0	CC037	F	Y	0.6	35.3	12.3	14.5
0	CC037	M	Y	9.1	19.2	19.8	14.7
0	CC037	M	Y	9	21.2	5.9	6.2
0	CC037	M	Y	12.8	21.3	8.5	21.2
1	CC037	F	Y	1.3	20.5	20	15.9
1	CC037	M	Y	4.1	22.8	13.5	8.3
1	CC037	M	Y	14.1	9.6	7.2	3.7
1	CC037	M	Y	10.7	15.6	8.2	6.4
2	CC037	M	Y	22.7	10.9	14.1	3.3
2	CC037	M	Y	11	27.2	3.1	6.1
1	CC041XCC012	M	Y	7.3	-3.7	13	12.2
1	CC041XCC012	M	Y	12.4	3.2	7.2	1.6
1	CC041XCC012	M	Y	-1.8	0.9	5.8	10.1
1	CC041XCC012	M	Y	4.2	0.6	5.5	9.8
1	CC041XCC012	M	Y	0.9	6	3.5	5.8
1	CC041XCC012	F	Y	8.7	28.5	7.2	4.7
1	CC041XCC012	F	Y	8.3	10.4	7.6	2.6
1	CC041XCC012	F	Y	14.3	17	5	3.6
1	CC041XCC012	F	Y	2	3.6	7	4.4
1	CC041XCC012	F	Y	7.4	24.2	4.7	2.3
1	CC041XCC012	F	Y	13	14.3	7.6	5.5
1	CC041XCC012	F	Y	4.3	15.2	5.2	6.6
1	CC041XCC012	F	Y	16.2	14.3	6.6	10.9
2	CC041XCC012	M	Y	7.7	13.4	4.5	2.1
2	CC041XCC012	M	Y	8.9	0.4	2.9	6.8
2	CC041XCC012	F	Y	8	16.6	1.5	2.9
2	CC041XCC012	M	Y	-13	7.8	3.9	15.3

Trial#	Strain	Sex	Inf?	PawAngle_RF	PawAngle_RH	PawAngleVariability_LF	PawAngleVariability_LH
2	CC041XCC012	M	Y	-2.7	5.4	5.3	2.5
2	CC041XCC012	M	Y	2.6	4.3	9.5	3.5
2	CC041XCC012	F	Y	-0.9	10	6.4	4.1
2	CC041XCC012	F	Y	3.4	7.3	12.4	11.7
2	CC041XCC012	F	Y	9	22.1	3.8	4.3
2	CC041XCC012	F	Y	3.3	21.3	6.1	4.5
2	CC041XCC012	F	Y	1.3	13.8	7.3	7.5
2	CC041XCC012	F	Y	-1.2	16.7	5.3	4.3
2	CC041XCC012	F	Y	-1.5	-1.5	4.6	13.8
0	CC051	F	Y	10	22.6	6.5	5.6
0	CC051	F	Y	2	21	10.1	5.5
0	CC051	F	Y	3.3	21.6	10.8	4
0	CC051	M	Y	7.7	16.5	7.7	4.5
1	CC051	F	Y	8.9	20.5	6.6	2.2
1	CC051	F	Y	10.8	20.1	8.2	3.9
1	CC051	F	Y	6.5	17.8	2.5	5.8
0	CC057	F	Y	12	27.5	6.5	3.8
0	CC057	F	Y	8	23.4	5.3	9.2
0	CC057	F	Y	10.1	21	13	12.4
0	CC057	M	Y	18.2	22.8	9.8	8.9
0	CC057	M	Y	8.2	14.7	10	15
0	CC057	M	Y	7.2	16.8	12.4	19
1	CC057	F	Y	10.2	22.6	8.1	1.9
1	CC057	F	Y	14.6	29.2	5.5	16.1
1	CC057	F	Y	30.2	26.5	17.3	22.7
1	CC057	F	Y	14.3	21.5	12.5	9.1
1	CC057	M	Y	11.5	19	13.1	3.5
1	CC057	M	Y	10.3	15.2	5.2	3.3
2	CC057	F	Y	14.3	21.5	12.5	9.1
0	CC078	F	Y	-17.4	13.9	5.2	3.8
0	CC078	F	Y	2	19.3	3.4	16.3
0	CC078	F	Y	3.2	20.8	7.9	3.2
0	CC078	M	Y	2.9	25.7	8.2	14.2
1	CC078	F	Y	22.1	11	5	2.8
1	CC078	F	Y	5.4	17.7	22.8	3.9
1	CC078	M	Y	2.7	8.8	10.1	4.7
2	CC078	F	Y	-1.5	24.4	6.2	7.3
2	CC078	M	Y	6.7	6.3	11.2	4.3
2	CC005	M	N	1.9	-1.2	12.8	18.4
2	CC005	M	Y	12.9	15.1	41.8	24
2	CC015	F	N	-8.5	-5.2	20.5	18.9
2	CC015	M	N	1.1	11.5	4.9	4.8
2	CC015	M	Y	-1.2	13.4	8.4	3.5
2	CC015	M	Y	-6.2	10.3	8.6	11.2
2	CC017	F	N	-11.8	15.2	22.4	7.8
2	CC017	M	N	11.9	15.1	12.8	4.4
2	CC017	F	Y	5.7	15.8	8	15.8
2	CC023	F	N	-2.3	13.6	4.2	9.5

Trial#	Strain	Sex	Inf?	PawAngle_RF	PawAngle_RH	PawAngleVariability_LF	PawAngleVariability_LH

Table S3. Raw DigiGait measurement data. Far left column indicates time point at which data was measured: T0 = pre-infection, T1 = 21dpi, and T3 = 89dpi. DigiGait parameters listed across the top indicate which limb is associated with the data, where appropriate: FL for left fore limb, FR for right fore limb, HL for left hind limb, and HR for right hind limb.

Table S3

Trial#	Strain	Sex	Inf?	PawAngleVariability_RF	PawAngleVariability_RH	PawAreaatPeakStanceinsq.cm_LF
1	CC002	F	N	14.5	7.1	0.19
1	CC025	F	N	7.1	7	0.19
1	CC012XCC032	F	N	15.5	5	0.25
2	CC012XCC032	F	N	7.9	2.4	0.35
1	CC012XCC032	M	N	2.5	13.1	0.28
2	CC012XCC032	M	N	3.2	2.8	0.19
1	CC013xCC041	F	N	6.6	13.2	0.5
2	CC013XCC041	F	N	5.4	3.7	0.38
1	CC013xCC041	M	N	34.3	31.9	0.08
2	CC013XCC041	M	N	13.7	3.9	0.36
1	CC032XCC013	F	N	23.6	20.6	0.16
2	CC032XCC013	F	N	15.5	4.6	0.37
1	CC041XCC012	F	N	11.4	8.7	0.39
1	CC041XCC012	F	N	20.5	19	0.2
2	CC041XCC012	F	N	16.4	8.9	0.17
1	CC032XCC013	M	N	8.7	12	0.4
2	CC032XCC013	M	N	26.6	15.8	0.22
1	CC041XCC012	M	N	10.8	11	0.27
2	CC041XCC012	M	N	9.5	3.6	0.36
0	CC012	F	N	20.1	7.3	0.2
2	CC012	F	N	19.1	18.3	0.48
0	CC012	M	N	4.2	11.6	0.19
2	CC012	M	N	11.1	11.4	0.29
0	CC057	F	N	5.6	10.2	0.35
1	CC057	F	N	3	4.9	0.44
0	CC057	M	N	3.3	16.6	0.3
1	CC057	M	N	6.2	2.6	0.45
0	CC078	F	N	2.9	6.5	0.42
1	CC078	F	N	7.7	7	0.29
2	CC078	F	N	19.1	9.4	0.26
0	CC078	M	N	5	12.6	0.3
2	CC078	M	N	21	11	0.3
0	CC002	F	N	12	22.2	0.39
1	CC002	F	N	14.5	7.1	0.19
0	CC002	M	N	15.2	18.1	0.32
1	CC002	M	N	9.5	10.6	0.41
2	CC002	M	N	3.1	6.5	0.39
0	CC006	F	N	2.4	4.6	0.53
1	CC006	F	N	11.4	6.1	0.34
2	CC006	F	N	4.3	18.2	0.2
0	CC006	M	N	14.2	32.6	0.3
0	CC023	F	N	14.6	12	0.21
1	CC023	F	N	24.4	11.7	0.37
2	CC023	F	N	5.4	10.4	0.55
0	CC023	M	N	5.6	12.7	0.24
1	CC023	M	N	6.7	4.3	0.32
0	CC027	F	N	27.1	19.8	0.18

Trial#	Strain	Sex	Inf?	PawAngleVariability_RF	PawAngleVariability_RH	PawAreaatPeakStanceinsq.cm_LF
1	CC027	F	N	8.3	15.6	0.28
2	CC027	F	N	5.3	6.2	0.4
0	CC027	M	N	22.8	21.2	0.17
1	CC027	M	N	6.4	31.7	0.35
1	CC027	M	N	8.6	30.9	0.32
2	CC027	M	N	11.4	3.5	0.5
0	CC005	F	N	5.6	4.3	0.26
1	CC005	F	N	26.5	29.5	0.13
2	CC005	F	N	41.4	41.5	0.08
0	CC011	F	N	12.6	10.1	0.38
1	CC011	F	N	7.2	8.7	0.74
2	CC011	F	N	1.9	3.9	0.47
1	CC017	F	N	12.9	13.1	0.39
0	CC005	M	N	4.9	4.1	0.37
1	CC005	M	N	18	34.7	0.16
2	CC005	M	N	21.7	32.9	0.16
0	CC011	M	N	3.6	9.9	0.26
1	CC011	M	N	2.1	3	0.3
2	CC011	M	N	21.7	9.7	0.53
0	CC017	M	N	32.1	27.3	0.1
1	CC017	M	N	17.8	18.4	0.27
1	CC006	F	N	24.2	21	0.18
0	CC037	F	N	14.8	7.1	0.24
0	CC051	F	N	8.2	5.6	0.18
1	CC051	F	N	4.8	11.2	0.14
1	CC006	M	N	9.6	16.5	0.46
0	CC037	M	N	10.3	4	0.12
1	CC037	M	N	13.2	12.9	0.25
0	CC005	F	N	8	17.5	0.25
1	CC005	F	N	4.6	4.6	0.37
2	CC005	F	N	10.4	4.7	0.23
0	CC011	F	N	10	3.4	0.26
1	CC011	F	N	4.3	2	0.29
2	CC011	F	N	2.3	3.7	0.19
0	CC011	M	N	8.2	6.4	0.32
1	CC011	M	N	5.5	5.1	0.33
2	CC011	M	N	7.3	4.5	0.19
1	CC037	M	N	12.7	4.6	0.53
2	CC037	M	N	11.8	8.9	0.16
1	CC051	M	N	4	3.5	0.18
2	CC051	M	N	15.1	1.7	0.2
0	CC027	F	N	3.8	2.8	0.44
1	CC027	F	N	11.2	8.9	0.28
2	CC027	F	N	10.3	10.7	0.32
0	CC015	M	N	8.5	4.7	0.25
1	CC015	M	N	16.7	28.6	0.17
2	CC015	M	N	7	7.5	0.19
0	CC027	M	N	5.3	7.1	0.2

Trial#	Strain	Sex	Inf?	PawAngleVariability_RF	PawAngleVariability_RH	PawAreaatPeakStanceinsq.cm_LF
1	CC027	M	N	5.4	4.4	0.41
2	CC027	M	N	7.7	13.1	0.26
0	CC015	F	N	3.3	0.31	0.38
1	CC015	F	N	13.4	9	0.19
0	CC017	F	N	3.5	8.6	0.27
0	CC023	F	N	12	6.5	0.28
1	CC023	F	N	6.1	7.7	0.34
0	CC005	M	N	10.8	15.3	0.38
1	CC005	M	N	9	3.7	0.37
0	CC015	M	N	11.8	10.2	0.4
1	CC015	M	N	16.2	12.5	0.31
0	CC017	M	N	7.8	8.1	0.41
1	CC017	M	N	20.3	9.4	0.26
0	CC023	M	N	3.5	4.1	0.44
1	CC023	M	N	24	4.5	0.45
0	CC051	M	N	6.1	4	0.38
0	CC002	F	Y	8.6	16.2	0.36
0	CC002	M	Y	15	10.2	0.26
1	CC002	M	Y	7.4	40.3	0.15
1	CC002	F	Y	20.9	33.8	0.14
1	CC002	F	Y	18.5	18.4	0.32
1	CC002	M	Y	7.4	40.3	0.15
1	CC002	M	Y	27	23.6	0.27
1	CC002	F	Y	20.9	33.8	0.14
1	CC002	F	Y	14.3	23.5	0.29
2	CC002	M	Y	22.1	23.5	0.45
2	CC002	F	Y	3.7	2.5	0.27
2	CC002	M	Y	10.8	11.4	0.16
0	CC005	F	Y	7.4	9.5	0.24
0	CC005	M	Y	8	8.2	0.3
0	CC005	M	Y	14	12	0.46
1	CC005	F	Y	16.3	14.1	0.24
1	CC005	M	Y	16.2	19.8	0.26
1	CC005	F	Y	4.3	4.1	0.58
1	CC005	M	Y	22	8.7	0.31
2	CC005	F	Y	17.3	37.4	0.3
2	CC005	M	Y	18.5	3.4	0.46
2	CC005	M	Y	18.1	22.1	0.52
2	CC005	M	Y	18.5	3.4	0.46
2	CC005	F	Y	13.3	30	0.3
0	CC006	F	Y	8.6	10	0.23
0	CC006	M	Y	12.4	17.4	0.16
0	CC006	F	Y	10.9	13.4	0.31
1	CC006	F	Y	26.7	22.7	0.18
1	CC006	M	Y	20	21.9	0.2
1	CC006	F	Y	7.2	12.8	0.61
1	CC006	M	Y	11.6	4.1	0.17
2	CC006	F	Y	10	15.1	0.27

Trial#	Strain	Sex	Inf?	PawAngleVariability_RF	PawAngleVariability_RH	PawAreaatPeakStanceinsq.cm_LF
2	CC006	M	Y	18.4	20.8	0.32
0	CC011	F	Y	12.5	1.9	0.74
0	CC011	M	Y	17.7	12.7	0.26
0	CC011	F	Y	4.7	5.5	0.14
0	CC011	F	Y	15.7	19.4	0.3
1	CC011	F	Y	3.5	1.5	0.23
1	CC011	M	Y	5.4	2.1	0.22
1	CC011	F	Y	6.8	5	0.4
1	CC011	M	Y	5.2	3.2	0.23
2	CC011	F	Y	7.1	3.6	0.45
2	CC011	M	Y	7.3	3.1	0.36
2	CC011	F	Y	3.5	5	0.25
2	CC011	M	Y	7.4	3.1	0.28
0	CC012	M	Y	8.1	7.9	0.43
0	CC012	M	Y	12.6	8.9	0.39
0	CC012	M	Y	12.4	3.4	0.28
0	CC012	M	Y	15.8	16.5	0.26
0	CC012	M	Y	9.8	13.7	0.36
0	CC012	F	Y	12	2.3	0.33
0	CC012	F	Y	18	8.7	0.33
0	CC012	F	Y	13.1	12.5	0.24
0	CC012	F	Y	32.1	22.9	0.47
0	CC012	F	Y	27.1	4.5	0.41
2	CC012	M	Y	9.6	19.9	0.55
2	CC012	M	Y	6	10.3	0.53
2	CC012	M	Y	18.8	8.5	0.53
2	CC012	M	Y	5.3	5.4	0.43
2	CC012	M	Y	4.1	10.5	0.49
2	CC012	F	Y	11.9	3.5	0.41
2	CC012	F	Y	4	8	0.37
2	CC012	F	Y	10.8	14.7	0.38
2	CC012	F	Y	4.4	2.1	0.37
2	CC012	F	Y	6.9	9.7	0.38
1	CC012XCC032	F	Y	22.1	20.4	0.28
1	CC012xCC032	F	Y	16.2	27.3	0.18
1	CC012XCC032	M	Y	7.8	10.4	0.25
1	CC012xCC032	M	Y	19.3	16.4	0.28
1	CC012XCC032	M	Y	21.6	20.1	0.19
1	CC012xCC032	M	Y	29.4	20.9	0.18
2	CC012XCC032	F	Y	3.2	2.5	0.35
2	CC012XCC032	F	Y	5.7	4.2	0.35
2	CC012XCC032	M	Y	10.3	10.8	0.28
2	CC012XCC032	M	Y	24.4	5.3	0.26
2	CC012XCC032	M	Y	13.3	4.7	0.43
2	CC012XCC032	M	Y	14	6.3	0.35
1	CC013xCC041	F	Y	9.4	10	0.43
1	CC013xCC041	F	Y	14.7	14.4	0.3
1	CC013xCC041	F	Y	14	17.6	0.22

Trial#	Strain	Sex	Inf?	PawAngleVariability_RF	PawAngleVariability_RH	PawAreaatPeakStanceinsq.cm_LF
1	CC013xCC041	M	Y	7.9	7.5	0.37
1	CC013xCC041	M	Y	13.2	27.1	0.34
2	CC013XCC041	F	Y	2.7	4.8	0.4
2	CC013XCC041	F	Y	7.9	10.8	0.5
2	CC013XCC041	F	Y	3.1	7.3	0.6
2	CC013XCC041	M	Y	7.3	3.5	0.36
2	CC013XCC041	M	Y	19.8	22.8	0.23
0	CC015	F	Y	4.9	11.9	0.31
0	CC015	M	Y	2.5	4.8	0.35
0	CC015	M	Y	9.5	7.7	0.33
1	CC015	F	Y	2.3	6.7	0.29
1	CC015	M	Y	11.5	8.4	0.23
1	CC015	M	Y	21	9	0.29
1	CC015	M	Y	13.1	11.7	0.24
2	CC015	F	Y	2.9	3.6	0.3
0	CC017	F	Y	11.7	5.5	0.22
0	CC017	M	Y	7.2	5.2	0.22
0	CC017	F	Y	19.9	33.7	0.44
0	CC017	M	Y	25.9	14	0.42
1	CC017	M	Y	14.8	13.7	0.4
1	CC017	F	Y	29.4	10.2	0.22
0	CC023	M	Y	3.3	4.6	0.35
0	CC023	F	Y	6.5	33.8	0.18
0	CC023	F	Y	16.1	12.8	0.24
0	CC023	M	Y	11.7	6.1	0.22
1	CC023	M	Y	13.7	12.5	0.2
1	CC023	F	Y	26.3	16.9	0.31
1	CC023	M	Y	13.5	8.1	0.27
1	CC023	M	Y	19.3	4.1	0.25
2	CC023	M	Y	5.1	2.5	0.41
2	CC023	F	Y	7.5	5	0.31
2	CC023	M	Y	14.7	20.1	0.13
0	CC025	M	Y	6.9	6.9	0.22
0	CC025	F	Y	14.1	9.9	0.28
0	CC025	M	Y	2.2	3.3	0.19
1	CC025	M	Y	8.5	7.4	0.22
1	CC025	F	Y	8.7	9	0.29
1	CC025	F	Y	8	6.7	0.2
1	CC025	F	Y	15	30.7	0.24
2	CC025	F	Y	5	5.7	0.25
2	CC025	M	Y	3	2.1	0.28
0	CC027	F	Y	12.9	2.6	0.3
0	CC027	M	Y	8.6	2.2	0.22
1	CC027	M	Y	19.3	27.3	0.29
1	CC027	M	Y	19.3	27.3	0.29
1	CC027	F	Y	17.7	21.8	0.26
1	CC027	F	Y	10	26.2	0.22
1	CC027	M	Y	7.5	12.3	0.19

Trial#	Strain	Sex	Inf?	PawAngleVariability_RF	PawAngleVariability_RH	PawAreaatPeakStanceinsq.cm_LF
1	CC027	M	Y	3.2	1.9	0.21
2	CC027	F	Y	7.5	5	0.31
2	CC027	M	Y	4.1	5.1	0.37
2	CC027	F	Y	16.6	13	0.27
2	CC027	M	Y	2.1	3.4	0.24
1	CC032XCC013	M	Y	7.5	5.9	0.28
1	CC032XCC013	M	Y	19.3	4.1	0.43
1	CC032XCC013	M	Y	7.6	14.4	0.21
1	CC032XCC013	F	Y	8.3	8.4	0.11
1	CC032XCC013	F	Y	7.2	4.1	0.19
1	CC032XCC013	F	Y	3.1	1.2	0.3
1	CC032XCC013	F	Y	3	1.2	0.34
1	CC032XCC013	F	Y	12	6.6	0.16
2	CC032XCC013	M	Y	11.3	3.4	0.36
2	CC032XCC013	M	Y	14.9	13.8	0.34
2	CC032XCC013	M	Y	25.2	14.1	0.19
2	CC032XCC013	M	Y	12.9	3.9	0.38
2	CC032XCC013	F	Y	9	1.6	0.21
2	CC032XCC013	F	Y	4.2	4	0.31
2	CC032XCC013	F	Y	8.1	2.3	0.52
2	CC032XCC013	F	Y	5	4.3	0.29
0	CC037	F	Y	9.8	7.2	0.22
0	CC037	M	Y	12.6	14.1	0.19
0	CC037	M	Y	14.4	2.1	0.16
0	CC037	M	Y	16.4	12.5	0.12
1	CC037	F	Y	8	3.4	0.39
1	CC037	M	Y	9.7	2.6	0.21
1	CC037	M	Y	6	5.6	0.24
1	CC037	M	Y	8.7	18.4	0.21
2	CC037	M	Y	4.3	6.5	0.21
2	CC037	M	Y	3	2.9	0.24
1	CC041XCC012	M	Y	16.1	17.4	0.41
1	CC041XCC012	M	Y	10.6	2.6	0.37
1	CC041XCC012	M	Y	13.2	8.5	0.26
1	CC041XCC012	M	Y	16.8	17.4	0.39
1	CC041XCC012	M	Y	3.2	6.2	0.39
1	CC041XCC012	F	Y	7.8	21.1	0.42
1	CC041XCC012	F	Y	5.3	2.6	0.47
1	CC041XCC012	F	Y	9.7	7.1	0.42
1	CC041XCC012	F	Y	7.6	4.5	0.33
1	CC041XCC012	F	Y	10.7	3.6	0.38
1	CC041XCC012	F	Y	7.1	6.5	0.43
1	CC041XCC012	F	Y	1.7	7.1	0.33
1	CC041XCC012	F	Y	9.3	8.1	0.33
2	CC041XCC012	M	Y	4.8	4.2	0.44
2	CC041XCC012	M	Y	10.6	5.6	0.66
2	CC041XCC012	F	Y	8.7	3.4	0.6
2	CC041XCC012	M	Y	8.6	2.6	0.33

Trial#	Strain	Sex	Inf?	PawAngleVariability_RF	PawAngleVariability_RH	PawAreaatPeakStanceinsq.cm_LF
2	CC041XCC012	M	Y	4.4	4.5	0.52
2	CC041XCC012	M	Y	4	2.4	0.33
2	CC041XCC012	F	Y	4.1	9.6	0.48
2	CC041XCC012	F	Y	4.4	12.6	0.5
2	CC041XCC012	F	Y	2.9	4	0.46
2	CC041XCC012	F	Y	3.7	2.1	0.48
2	CC041XCC012	F	Y	9.5	3.8	0.46
2	CC041XCC012	F	Y	5.6	5.6	0.44
2	CC041XCC012	F	Y	9.7	10.6	0.61
0	CC051	F	Y	11.1	5.1	0.2
0	CC051	F	Y	8.4	5.7	0.17
0	CC051	F	Y	5.5	3.9	0.18
0	CC051	M	Y	5.6	6.4	0.22
1	CC051	F	Y	4.3	4	0.18
1	CC051	F	Y	6.7	6.4	0.29
1	CC051	F	Y	6.1	3	0.21
0	CC057	F	Y	4.8	5.4	0.26
0	CC057	F	Y	3.1	5.4	0.35
0	CC057	F	Y	8.7	13.5	0.18
0	CC057	M	Y	9.8	6.4	0.27
0	CC057	M	Y	13	10.2	0.3
0	CC057	M	Y	15.2	15.6	0.29
1	CC057	F	Y	11.7	6.1	0.37
1	CC057	F	Y	16.1	9.2	0.34
1	CC057	F	Y	9.2	11.8	0.22
1	CC057	F	Y	19.9	16.5	0.24
1	CC057	M	Y	2.9	1.5	0.37
1	CC057	M	Y	6.9	3	0.44
2	CC057	F	Y	19.9	16.5	0.24
0	CC078	F	Y	18.3	17.3	0.31
0	CC078	F	Y	5.5	6.9	0.28
0	CC078	F	Y	4.1	3.4	0.3
0	CC078	M	Y	3	2.8	0.25
1	CC078	F	Y	4.1	1.9	0.29
1	CC078	F	Y	3.7	5.5	0.37
1	CC078	M	Y	4.4	7.2	0.36
2	CC078	F	Y	12.8	12.3	0.32
2	CC078	M	Y	10.7	8.5	0.3
2	CC005	M	N	17.5	26.4	0.16
2	CC005	M	Y	27.9	25.1	0.11
2	CC015	F	N	28.8	18.9	0.35
2	CC015	M	N	8.3	5	0.32
2	CC015	M	Y	10.4	12.9	0.33
2	CC015	M	Y	18.1	11.6	0.48
2	CC017	F	N	23	7.2	0.25
2	CC017	M	N	9	6.5	0.11
2	CC017	F	Y	8.6	16.6	0.37
2	CC023	F	N	9.6	4.8	0.26

Trial#	Strain	Sex	Inf?	PawAngleVariability_RF	PawAngleVariability_RH	PawAreaatPeakStanceinsq.cm_LF

Table S3. Raw DigiGait measurement data. Far left column indicates time point at which data was measured: T0 = pre-infection, T1 = 21dpi, and T3 = 89dpi. DigiGait parameters listed across the top indicate which limb is associated with the data, where appropriate: FL for left fore limb, FR for right fore limb, HL for left hind limb, and HR for right hind limb.

Table S3

Trial#	Strain	Sex	Inf?	PawAreaatPeakStanceinsq.cm_LH	PawAreaatPeakStanceinsq.cm_RF
1	CC002	F	N	0.11	0.19
1	CC025	F	N	0.66	0.18
1	CC012XCC032	F	N	0.33	0.23
2	CC012XCC032	F	N	0.84	0.45
1	CC012XCC032	M	N	0.51	0.26
2	CC012XCC032	M	N	0.56	0.25
1	CC013xCC041	F	N	0.4	0.45
2	CC013XCC041	F	N	0.56	0.29
1	CC013xCC041	M	N	0.16	0.07
2	CC013XCC041	M	N	0.6	0.39
1	CC032XCC013	F	N	0.33	0.15
2	CC032XCC013	F	N	0.38	0.32
1	CC041XCC012	F	N	0.47	0.38
1	CC041XCC012	F	N	0.19	0.17
2	CC041XCC012	F	N	0.67	0.21
1	CC032XCC013	M	N	0.54	0.3
2	CC032XCC013	M	N	0.5	0.19
1	CC041XCC012	M	N	0.31	0.19
2	CC041XCC012	M	N	0.57	0.32
0	CC012	F	N	0.33	0.26
2	CC012	F	N	0.69	0.47
0	CC012	M	N	0.32	0.28
2	CC012	M	N	0.44	0.34
0	CC057	F	N	0.49	0.29
1	CC057	F	N	0.83	0.43
0	CC057	M	N	0.28	0.42
1	CC057	M	N	0.78	0.47
0	CC078	F	N	0.38	0.35
1	CC078	F	N	0.69	0.33
2	CC078	F	N	0.46	0.23
0	CC078	M	N	0.4	0.25
2	CC078	M	N	0.49	0.22
0	CC002	F	N	0.34	0.23
1	CC002	F	N	0.11	0.19
0	CC002	M	N	0.38	0.23
1	CC002	M	N	0.51	0.4
2	CC002	M	N	0.75	0.37
0	CC006	F	N	1.07	0.41
1	CC006	F	N	0.62	0.4
2	CC006	F	N	0.25	0.25
0	CC006	M	N	0.27	0.3
0	CC023	F	N	0.3	0.15
1	CC023	F	N	0.49	0.32
2	CC023	F	N	0.92	0.47
0	CC023	M	N	0.45	0.19
1	CC023	M	N	0.64	0.29
0	CC027	F	N	0.07	0.13

Trial#	Strain	Sex	Inf?	PawAreaatPeakStanceinsq.cm_LH	PawAreaatPeakStanceinsq.cm_RF
1	CC027	F	N	0.27	0.29
2	CC027	F	N	0.62	0.32
0	CC027	M	N	0.08	0.21
1	CC027	M	N	0.42	0.32
1	CC027	M	N	0.25	0.24
2	CC027	M	N	0.79	0.38
0	CC005	F	N	0.64	0.3
1	CC005	F	N	0.21	0.14
2	CC005	F	N	0.07	0.08
0	CC011	F	N	0.48	0.46
1	CC011	F	N	1.4	0.68
2	CC011	F	N	0.99	0.47
1	CC017	F	N	0.49	0.34
0	CC005	M	N	0.81	0.34
1	CC005	M	N	0.24	0.25
2	CC005	M	N	0.23	0.16
0	CC011	M	N	0.68	0.27
1	CC011	M	N	0.7	0.26
2	CC011	M	N	0.77	0.36
0	CC017	M	N	0.05	0.09
1	CC017	M	N	0.55	0.3
1	CC006	F	N	0.16	0.14
0	CC037	F	N	0.44	0.17
0	CC051	F	N	0.37	0.19
1	CC051	F	N	0.23	0.14
1	CC006	M	N	0.7	0.35
0	CC037	M	N	0.44	0.16
1	CC037	M	N	0.44	0.35
0	CC005	F	N	0.33	0.24
1	CC005	F	N	0.69	0.35
2	CC005	F	N	0.18	0.21
0	CC011	F	N	0.57	0.29
1	CC011	F	N	1.21	0.24
2	CC011	F	N	0.68	0.18
0	CC011	M	N	0.67	0.25
1	CC011	M	N	0.83	0.3
2	CC011	M	N	0.49	0.16
1	CC037	M	N	0.91	0.49
2	CC037	M	N	0.42	0.18
1	CC051	M	N	0.67	0.23
2	CC051	M	N	0.54	0.22
0	CC027	F	N	0.52	0.4
1	CC027	F	N	0.38	0.21
2	CC027	F	N	0.4	0.27
0	CC015	M	N	0.34	0.23
1	CC015	M	N	0.44	0.19
2	CC015	M	N	0.51	0.15
0	CC027	M	N	0.28	0.2

Trial#	Strain	Sex	Inf?	PawAreaatPeakStanceinsq.cm_LH	PawAreaatPeakStanceinsq.cm_RF
1	CC027	M	N	0.95	0.35
2	CC027	M	N	0.39	0.2
0	CC015	F	N	0.26	0.41
1	CC015	F	N	0.25	0.19
0	CC017	F	N	0.45	0.29
0	CC023	F	N	0.4	0.35
1	CC023	F	N	0.58	0.3
0	CC005	M	N	0.43	0.35
1	CC005	M	N	0.55	0.36
0	CC015	M	N	0.63	0.31
1	CC015	M	N	0.65	0.25
0	CC017	M	N	0.42	0.36
1	CC017	M	N	0.28	0.21
0	CC023	M	N	0.58	0.46
1	CC023	M	N	0.56	0.43
0	CC051	M	N	0.77	0.38
0	CC002	F	Y	0.3	0.34
0	CC002	M	Y	0.41	0.28
1	CC002	M	Y	0.11	0.17
1	CC002	F	Y	0.1	0.17
1	CC002	F	Y	0.42	0.28
1	CC002	M	Y	0.11	0.17
1	CC002	M	Y	0.23	0.17
1	CC002	F	Y	0.1	0.17
1	CC002	F	Y	0.13	0.25
2	CC002	M	Y	0.42	0.36
2	CC002	F	Y	0.54	0.22
2	CC002	M	Y	0.46	0.16
0	CC005	F	Y	0.6	0.3
0	CC005	M	Y	0.63	0.38
0	CC005	M	Y	0.84	0.52
1	CC005	F	Y	0.39	0.17
1	CC005	M	Y	0.15	0.19
1	CC005	F	Y	0.98	0.35
1	CC005	M	Y	0.45	0.38
2	CC005	F	Y	0.39	0.31
2	CC005	M	Y	0.76	0.29
2	CC005	M	Y	0.7	0.29
2	CC005	M	Y	0.76	0.29
2	CC005	F	Y	0.42	0.3
0	CC006	F	Y	0.33	0.18
0	CC006	M	Y	0.08	0.07
0	CC006	F	Y	0.38	0.27
1	CC006	F	Y	0.2	0.11
1	CC006	M	Y	0.15	0.18
1	CC006	F	Y	0.84	0.53
1	CC006	M	Y	0.55	0.29
2	CC006	F	Y	0.52	0.22

Trial#	Strain	Sex	Inf?	PawAreaatPeakStanceinsq.cm_LH	PawAreaatPeakStanceinsq.cm_RF
2	CC006	M	Y	0.38	0.32
0	CC011	F	Y	1.01	0.6
0	CC011	M	Y	0.6	0.21
0	CC011	F	Y	0.29	0.13
0	CC011	F	Y	0.4	0.31
1	CC011	F	Y	0.58	0.19
1	CC011	M	Y	0.54	0.27
1	CC011	F	Y	0.9	0.29
1	CC011	M	Y	1.02	0.27
2	CC011	F	Y	0.96	0.39
2	CC011	M	Y	0.66	0.34
2	CC011	F	Y	0.66	0.2
2	CC011	M	Y	0.71	0.2
0	CC012	M	Y	0.75	0.43
0	CC012	M	Y	0.64	0.31
0	CC012	M	Y	0.49	0.28
0	CC012	M	Y	0.39	0.28
0	CC012	M	Y	0.64	0.34
0	CC012	F	Y	0.61	0.29
0	CC012	F	Y	0.69	0.22
0	CC012	F	Y	0.33	0.18
0	CC012	F	Y	0.68	0.51
0	CC012	F	Y	0.67	0.31
2	CC012	M	Y	1.08	0.52
2	CC012	M	Y	0.77	0.65
2	CC012	M	Y	0.79	0.72
2	CC012	M	Y	0.66	0.38
2	CC012	M	Y	0.92	0.48
2	CC012	F	Y	0.82	0.41
2	CC012	F	Y	0.57	0.31
2	CC012	F	Y	0.54	0.3
2	CC012	F	Y	0.79	0.47
2	CC012	F	Y	0.6	0.36
1	CC012XCC032	F	Y	0.51	0.34
1	CC012xCC032	F	Y	0.13	0.18
1	CC012XCC032	M	Y	0.52	0.24
1	CC012xCC032	M	Y	0.39	0.24
1	CC012XCC032	M	Y	0.28	0.29
1	CC012xCC032	M	Y	0.19	0.14
2	CC012XCC032	F	Y	0.7	0.32
2	CC012XCC032	F	Y	0.71	0.38
2	CC012XCC032	M	Y	0.86	0.27
2	CC012XCC032	M	Y	0.56	0.22
2	CC012XCC032	M	Y	0.97	0.36
2	CC012XCC032	M	Y	0.95	0.38
1	CC013xCC041	F	Y	0.49	0.42
1	CC013xCC041	F	Y	0.31	0.3
1	CC013xCC041	F	Y	0.2	0.22

Trial#	Strain	Sex	Inf?	PawAreaatPeakStanceinsq.cm_LH	PawAreaatPeakStanceinsq.cm_RF
1	CC013xCC041	M	Y	0.51	0.34
1	CC013xCC041	M	Y	0.46	0.4
2	CC013XCC041	F	Y	0.79	0.43
2	CC013XCC041	F	Y	0.97	0.7
2	CC013XCC041	F	Y	1.06	0.54
2	CC013XCC041	M	Y	1.15	0.37
2	CC013XCC041	M	Y	0.35	0.22
0	CC015	F	Y	0.23	0.31
0	CC015	M	Y	0.39	0.38
0	CC015	M	Y	0.52	0.28
1	CC015	F	Y	0.34	0.22
1	CC015	M	Y	0.27	0.24
1	CC015	M	Y	0.34	0.34
1	CC015	M	Y	0.33	0.23
2	CC015	F	Y	0.45	0.22
0	CC017	F	Y	0.4	0.25
0	CC017	M	Y	0.41	0.28
0	CC017	F	Y	0.79	0.39
0	CC017	M	Y	0.52	0.29
1	CC017	M	Y	0.49	0.32
1	CC017	F	Y	0.29	0.29
0	CC023	M	Y	0.52	0.3
0	CC023	F	Y	0.31	0.16
0	CC023	F	Y	0.27	0.26
0	CC023	M	Y	0.38	0.26
1	CC023	M	Y	0.29	0.21
1	CC023	F	Y	0.22	0.28
1	CC023	M	Y	0.38	0.14
1	CC023	M	Y	0.38	0.15
2	CC023	M	Y	0.49	0.45
2	CC023	F	Y	0.42	0.41
2	CC023	M	Y	0.25	0.14
0	CC025	M	Y	0.59	0.21
0	CC025	F	Y	0.4	0.38
0	CC025	M	Y	0.38	0.14
1	CC025	M	Y	0.59	0.21
1	CC025	F	Y	0.7	0.26
1	CC025	F	Y	0.53	0.17
1	CC025	F	Y	0.6	0.21
2	CC025	F	Y	0.52	0.19
2	CC025	M	Y	0.67	0.2
0	CC027	F	Y	0.44	0.21
0	CC027	M	Y	0.4	0.17
1	CC027	M	Y	0.26	0.33
1	CC027	M	Y	0.26	0.33
1	CC027	F	Y	0.44	0.28
1	CC027	F	Y	0.37	0.15
1	CC027	M	Y	0.52	0.19

Trial#	Strain	Sex	Inf?	PawAreaatPeakStanceinsq.cm_LH	PawAreaatPeakStanceinsq.cm_RF
1	CC027	M	Y	0.46	0.16
2	CC027	F	Y	0.42	0.41
2	CC027	M	Y	0.56	0.35
2	CC027	F	Y	0.46	0.19
2	CC027	M	Y	0.69	0.27
1	CC032XCC013	M	Y	0.45	0.28
1	CC032XCC013	M	Y	0.87	0.33
1	CC032XCC013	M	Y	0.38	0.16
1	CC032XCC013	F	Y	0.14	0.15
1	CC032XCC013	F	Y	0.32	0.14
1	CC032XCC013	F	Y	0.6	0.41
1	CC032XCC013	F	Y	0.64	0.39
1	CC032XCC013	F	Y	0.4	0.12
2	CC032XCC013	M	Y	0.62	0.24
2	CC032XCC013	M	Y	0.6	0.29
2	CC032XCC013	M	Y	0.66	0.23
2	CC032XCC013	M	Y	0.73	0.3
2	CC032XCC013	F	Y	0.62	0.22
2	CC032XCC013	F	Y	0.78	0.36
2	CC032XCC013	F	Y	0.84	0.4
2	CC032XCC013	F	Y	0.75	0.29
0	CC037	F	Y	0.24	0.24
0	CC037	M	Y	0.37	0.22
0	CC037	M	Y	0.41	0.19
0	CC037	M	Y	0.18	0.17
1	CC037	F	Y	0.59	0.41
1	CC037	M	Y	0.32	0.19
1	CC037	M	Y	0.56	0.24
1	CC037	M	Y	0.43	0.2
2	CC037	M	Y	0.45	0.18
2	CC037	M	Y	0.55	0.2
1	CC041XCC012	M	Y	0.49	0.3
1	CC041XCC012	M	Y	0.52	0.17
1	CC041XCC012	M	Y	0.41	0.37
1	CC041XCC012	M	Y	0.48	0.34
1	CC041XCC012	M	Y	0.61	0.37
1	CC041XCC012	F	Y	0.7	0.42
1	CC041XCC012	F	Y	0.73	0.35
1	CC041XCC012	F	Y	0.62	0.28
1	CC041XCC012	F	Y	0.87	0.32
1	CC041XCC012	F	Y	0.66	0.4
1	CC041XCC012	F	Y	0.55	0.29
1	CC041XCC012	F	Y	0.82	0.37
1	CC041XCC012	F	Y	0.61	0.35
2	CC041XCC012	M	Y	0.81	0.45
2	CC041XCC012	M	Y	1.03	0.5
2	CC041XCC012	F	Y	0.89	0.5
2	CC041XCC012	M	Y	0.26	0.38

Trial#	Strain	Sex	Inf?	PawAreaatPeakStanceinsq.cm_LH	PawAreaatPeakStanceinsq.cm_RF
2	CC041XCC012	M	Y	0.69	0.49
2	CC041XCC012	M	Y	0.38	0.41
2	CC041XCC012	F	Y	0.75	0.49
2	CC041XCC012	F	Y	0.55	0.52
2	CC041XCC012	F	Y	0.84	0.38
2	CC041XCC012	F	Y	0.65	0.48
2	CC041XCC012	F	Y	0.78	0.44
2	CC041XCC012	F	Y	0.69	0.54
2	CC041XCC012	F	Y	1.28	0.59
0	CC051	F	Y	0.43	0.16
0	CC051	F	Y	0.35	0.1
0	CC051	F	Y	0.3	0.13
0	CC051	M	Y	0.4	0.23
1	CC051	F	Y	0.5	0.19
1	CC051	F	Y	0.56	0.18
1	CC051	F	Y	0.52	0.17
0	CC057	F	Y	0.51	0.29
0	CC057	F	Y	0.42	0.33
0	CC057	F	Y	0.16	0.18
0	CC057	M	Y	0.3	0.31
0	CC057	M	Y	0.18	0.26
0	CC057	M	Y	0.31	0.27
1	CC057	F	Y	0.51	0.37
1	CC057	F	Y	0.46	0.34
1	CC057	F	Y	0.31	0.21
1	CC057	F	Y	0.32	0.19
1	CC057	M	Y	0.48	0.41
1	CC057	M	Y	0.85	0.39
2	CC057	F	Y	0.32	0.19
0	CC078	F	Y	0.44	0.3
0	CC078	F	Y	0.43	0.23
0	CC078	F	Y	0.36	0.26
0	CC078	M	Y	0.37	0.28
1	CC078	F	Y	0.44	0.3
1	CC078	F	Y	0.64	0.34
1	CC078	M	Y	0.56	0.32
2	CC078	F	Y	0.48	0.2
2	CC078	M	Y	0.48	0.25
2	CC005	M	N	0.27	0.19
2	CC005	M	Y	0.21	0.11
2	CC015	F	N	0.7	0.26
2	CC015	M	N	0.86	0.23
2	CC015	M	Y	0.8	0.35
2	CC015	M	Y	0.82	0.3
2	CC017	F	N	0.75	0.18
2	CC017	M	N	0.6	0.14
2	CC017	F	Y	0.47	0.46
2	CC023	F	N	0.58	0.26

Trial#	Strain	Sex	Inf?	PawAreaatPeakStanceinsq.cm_LH	PawAreaatPeakStanceinsq.cm_RF

Table S3. Raw DigiGait measurement data. Far left column indicates time point at which data was measured: T0 = pre-infection, T1 = 21dpi, and T3 = 89dpi. DigiGait parameters listed across the top indicate which limb is associated with the data, where appropriate: FL for left fore limb, FR for right fore limb, HL for left hind limb, and HR for right hind limb.

Table S3

Trial#	Strain	Sex	Inf?	PawAreaatPeakStanceinsq.cm_RH	PawAreaVariabilityatPeakStanceinsq.cm_LF
1	CC002	F	N	0.25	0.03
1	CC025	F	N	0.53	0.02
1	CC012XCC032	F	N	0.34	0.02
2	CC012XCC032	F	N	0.84	0.05
1	CC012XCC032	M	N	0.33	0.02
2	CC012XCC032	M	N	0.64	0.02
1	CC013xCC041	F	N	0.61	0.05
2	CC013XCC041	F	N	0.64	0.01
1	CC013xCC041	M	N	0.05	0.05
2	CC013XCC041	M	N	0.76	0.05
1	CC032XCC013	F	N	0.22	0.03
2	CC032XCC013	F	N	0.52	0.05
1	CC041XCC012	F	N	0.49	0.14
1	CC041XCC012	F	N	0.29	0.04
2	CC041XCC012	F	N	0.48	0.01
1	CC032XCC013	M	N	0.39	0.2
2	CC032XCC013	M	N	0.3	0.04
1	CC041XCC012	M	N	0.23	0.04
2	CC041XCC012	M	N	0.56	0.06
0	CC012	F	N	0.42	0.03
2	CC012	F	N	0.68	0.03
0	CC012	M	N	0.41	0.04
2	CC012	M	N	0.62	0.04
0	CC057	F	N	0.35	0.04
1	CC057	F	N	0.7	0.05
0	CC057	M	N	0.57	0.12
1	CC057	M	N	0.83	0.01
0	CC078	F	N	0.57	0.02
1	CC078	F	N	0.85	0.05
2	CC078	F	N	0.25	0.04
0	CC078	M	N	0.4	0.02
2	CC078	M	N	0.52	0.05
0	CC002	F	N	0.21	0.06
1	CC002	F	N	0.25	0.03
0	CC002	M	N	0.22	0.04
1	CC002	M	N	0.37	0.16
2	CC002	M	N	0.56	0.09
0	CC006	F	N	0.69	0.04
1	CC006	F	N	0.4	0.07
2	CC006	F	N	0.39	0.08
0	CC006	M	N	0.23	0.05
0	CC023	F	N	0.27	0.06
1	CC023	F	N	0.49	0.11
2	CC023	F	N	0.93	0.18
0	CC023	M	N	0.28	0.01
1	CC023	M	N	0.64	0.06
0	CC027	F	N	0.09	0.05

Trial#	Strain	Sex	Inf?	PawAreaatPeakStanceinsq.cm_RH	PawAreaVariabilityatPeakStanceinsq.cm_LF
1	CC027	F	N	0.23	0.07
2	CC027	F	N	0.49	0.02
0	CC027	M	N	0.08	0.08
1	CC027	M	N	0.32	0.09
1	CC027	M	N	0.19	0.07
2	CC027	M	N	0.76	0.02
0	CC005	F	N	0.53	0.07
1	CC005	F	N	0.2	0.04
2	CC005	F	N	0.08	0.04
0	CC011	F	N	0.83	0.07
1	CC011	F	N	0.93	0.23
2	CC011	F	N	0.95	0.04
1	CC017	F	N	0.41	0.08
0	CC005	M	N	0.71	0.02
1	CC005	M	N	0.32	0.07
2	CC005	M	N	0.29	0.04
0	CC011	M	N	0.75	0.01
1	CC011	M	N	0.68	0.02
2	CC011	M	N	0.64	0.13
0	CC017	M	N	0.06	0.04
1	CC017	M	N	0.41	0.06
1	CC006	F	N	0.22	0.13
0	CC037	F	N	0.36	0.05
0	CC051	F	N	0.43	0.05
1	CC051	F	N	0.29	0.04
1	CC006	M	N	0.55	0.04
0	CC037	M	N	0.39	0.03
1	CC037	M	N	0.55	0.04
0	CC005	F	N	0.31	0.03
1	CC005	F	N	0.69	0.18
2	CC005	F	N	0.24	0.02
0	CC011	F	N	0.34	0.02
1	CC011	F	N	1.01	0.02
2	CC011	F	N	0.65	0.02
0	CC011	M	N	0.88	0.16
1	CC011	M	N	0.86	0.03
2	CC011	M	N	0.43	0.02
1	CC037	M	N	0.8	0.07
2	CC037	M	N	0.38	0.03
1	CC051	M	N	0.74	0.01
2	CC051	M	N	0.61	0.01
0	CC027	F	N	0.43	0.03
1	CC027	F	N	0.34	0.07
2	CC027	F	N	0.3	0.09
0	CC015	M	N	0.32	0.03
1	CC015	M	N	0.38	0.05
2	CC015	M	N	0.42	0.02
0	CC027	M	N	0.27	0.02

Trial#	Strain	Sex	Inf?	PawAreaatPeakStanceinsq.cm_RH	PawAreaVariabilityatPeakStanceinsq.cm_LF
1	CC027	M	N	0.84	0.17
2	CC027	M	N	0.39	0.05
0	CC015	F	N	0.08	0.09
1	CC015	F	N	0.27	0.07
0	CC017	F	N	0.43	0.03
0	CC023	F	N	0.46	0.07
1	CC023	F	N	0.58	0.04
0	CC005	M	N	0.25	0.02
1	CC005	M	N	0.39	0.04
0	CC015	M	N	0.47	0.05
1	CC015	M	N	0.32	0.14
0	CC017	M	N	0.43	0.06
1	CC017	M	N	0.33	0.09
0	CC023	M	N	0.58	0.02
1	CC023	M	N	0.71	0.15
0	CC051	M	N	0.61	0.05
0	CC002	F	Y	0.32	0.09
0	CC002	M	Y	0.28	0.08
1	CC002	M	Y	0.07	0.01
1	CC002	F	Y	0.14	0.03
1	CC002	F	Y	0.37	0.1
1	CC002	M	Y	0.07	0.01
1	CC002	M	Y	0.12	0.08
1	CC002	F	Y	0.14	0.03
1	CC002	F	Y	0.15	0.11
2	CC002	M	Y	0.32	0.03
2	CC002	F	Y	0.59	0.02
2	CC002	M	Y	0.31	0.03
0	CC005	F	Y	0.63	0.07
0	CC005	M	Y	0.53	0.09
0	CC005	M	Y	0.76	0.05
1	CC005	F	Y	0.31	0.04
1	CC005	M	Y	0.13	0.03
1	CC005	F	Y	0.94	0.03
1	CC005	M	Y	0.63	0.1
2	CC005	F	Y	0.35	0.06
2	CC005	M	Y	0.75	0.13
2	CC005	M	Y	0.32	0.06
2	CC005	M	Y	0.75	0.13
2	CC005	F	Y	0.27	0.04
0	CC006	F	Y	0.34	0.02
0	CC006	M	Y	0.05	0.05
0	CC006	F	Y	0.35	0.07
1	CC006	F	Y	0.17	0.05
1	CC006	M	Y	0.17	0.09
1	CC006	F	Y	0.69	0.23
1	CC006	M	Y	0.6	0.05
2	CC006	F	Y	0.4	0.08

Trial#	Strain	Sex	Inf?	PawAreaatPeakStanceinsq.cm_RH	PawAreaVariabilityatPeakStanceinsq.cm_LF
2	CC006	M	Y	0.43	0.12
0	CC011	F	Y	1.18	0.24
0	CC011	M	Y	0.51	0.06
0	CC011	F	Y	0.32	0.01
0	CC011	F	Y	0.46	0.04
1	CC011	F	Y	0.66	0.02
1	CC011	M	Y	0.6	0.02
1	CC011	F	Y	0.73	0.02
1	CC011	M	Y	0.91	0.02
2	CC011	F	Y	0.95	0.01
2	CC011	M	Y	0.85	0.04
2	CC011	F	Y	0.64	0.01
2	CC011	M	Y	0.69	0.16
0	CC012	M	Y	0.63	0.03
0	CC012	M	Y	0.46	0.16
0	CC012	M	Y	0.52	0.03
0	CC012	M	Y	0.51	0.06
0	CC012	M	Y	0.64	0.07
0	CC012	F	Y	0.61	0.09
0	CC012	F	Y	0.61	0.02
0	CC012	F	Y	0.38	0.11
0	CC012	F	Y	0.68	0.12
0	CC012	F	Y	0.5	0.16
2	CC012	M	Y	0.86	0.06
2	CC012	M	Y	0.79	0.05
2	CC012	M	Y	0.84	0.03
2	CC012	M	Y	0.84	0.04
2	CC012	M	Y	0.85	0.02
2	CC012	F	Y	0.89	0.1
2	CC012	F	Y	0.54	0.05
2	CC012	F	Y	0.33	0.1
2	CC012	F	Y	0.75	0.06
2	CC012	F	Y	0.51	0.04
1	CC012XCC032	F	Y	0.48	0.14
1	CC012xCC032	F	Y	0.11	0.04
1	CC012XCC032	M	Y	0.41	0.06
1	CC012xCC032	M	Y	0.36	0.05
1	CC012XCC032	M	Y	0.3	0.02
1	CC012xCC032	M	Y	0.18	0.04
2	CC012XCC032	F	Y	0.78	0.03
2	CC012XCC032	F	Y	0.58	0.02
2	CC012XCC032	M	Y	0.57	0.05
2	CC012XCC032	M	Y	0.63	0.04
2	CC012XCC032	M	Y	0.78	0.04
2	CC012XCC032	M	Y	0.65	0.04
1	CC013xCC041	F	Y	0.5	0.06
1	CC013xCC041	F	Y	0.26	0.08
1	CC013xCC041	F	Y	0.22	0.06

Trial#	Strain	Sex	Inf?	PawAreaatPeakStanceinsq.cm_RH	PawAreaVariabilityatPeakStanceinsq.cm_LF
1	CC013xCC041	M	Y	0.51	0.02
1	CC013xCC041	M	Y	0.32	0.04
2	CC013XCC041	F	Y	0.95	0.02
2	CC013XCC041	F	Y	1.2	0.08
2	CC013XCC041	F	Y	1.16	0.06
2	CC013XCC041	M	Y	0.82	0.03
2	CC013XCC041	M	Y	0.19	0.08
0	CC015	F	Y	0.33	0.04
0	CC015	M	Y	0.52	0.03
0	CC015	M	Y	0.48	0.06
1	CC015	F	Y	0.33	0.02
1	CC015	M	Y	0.32	0.08
1	CC015	M	Y	0.55	0.07
1	CC015	M	Y	0.34	0.08
2	CC015	F	Y	0.54	0.06
0	CC017	F	Y	0.32	0.07
0	CC017	M	Y	0.5	0.04
0	CC017	F	Y	0.64	0.17
0	CC017	M	Y	0.41	0.08
1	CC017	M	Y	0.36	0.09
1	CC017	F	Y	0.34	0.09
0	CC023	M	Y	0.45	0.01
0	CC023	F	Y	0.1	0.02
0	CC023	F	Y	0.31	0.06
0	CC023	M	Y	0.48	0.06
1	CC023	M	Y	0.27	0.07
1	CC023	F	Y	0.28	0.09
1	CC023	M	Y	0.21	0.09
1	CC023	M	Y	0.24	0.03
2	CC023	M	Y	0.6	0.02
2	CC023	F	Y	0.49	0.11
2	CC023	M	Y	0.14	0.06
0	CC025	M	Y	0.39	0.01
0	CC025	F	Y	0.42	0.02
0	CC025	M	Y	0.32	0.02
1	CC025	M	Y	0.39	0.01
1	CC025	F	Y	0.5	0.06
1	CC025	F	Y	0.4	0.02
1	CC025	F	Y	0.41	0.1
2	CC025	F	Y	0.41	0.1
2	CC025	M	Y	0.67	0.16
0	CC027	F	Y	0.34	0.09
0	CC027	M	Y	0.35	0.09
1	CC027	M	Y	0.2	0.12
1	CC027	M	Y	0.2	0.12
1	CC027	F	Y	0.21	0.1
1	CC027	F	Y	0.16	0.06
1	CC027	M	Y	0.5	0.02

Trial#	Strain	Sex	Inf?	PawAreaatPeakStanceinsq.cm_RH	PawAreaVariabilityatPeakStanceinsq.cm_LF
1	CC027	M	Y	0.38	0.02
2	CC027	F	Y	0.49	0.11
2	CC027	M	Y	0.55	0.1
2	CC027	F	Y	0.26	0.1
2	CC027	M	Y	0.68	0.06
1	CC032XCC013	M	Y	0.48	0.04
1	CC032XCC013	M	Y	0.5	0.03
1	CC032XCC013	M	Y	0.33	0.12
1	CC032XCC013	F	Y	0.28	0.02
1	CC032XCC013	F	Y	0.38	0.06
1	CC032XCC013	F	Y	0.51	0.07
1	CC032XCC013	F	Y	0.51	0.03
1	CC032XCC013	F	Y	0.39	0.02
2	CC032XCC013	M	Y	0.68	0.02
2	CC032XCC013	M	Y	0.53	0.09
2	CC032XCC013	M	Y	0.44	0.04
2	CC032XCC013	M	Y	0.5	0.06
2	CC032XCC013	F	Y	0.49	0.02
2	CC032XCC013	F	Y	0.74	0.03
2	CC032XCC013	F	Y	0.76	0.08
2	CC032XCC013	F	Y	0.53	0.04
0	CC037	F	Y	0.42	0.06
0	CC037	M	Y	0.47	0.09
0	CC037	M	Y	0.45	0.05
0	CC037	M	Y	0.2	0.07
1	CC037	F	Y	0.62	0.1
1	CC037	M	Y	0.37	0.02
1	CC037	M	Y	0.56	0.04
1	CC037	M	Y	0.43	0.01
2	CC037	M	Y	0.45	0.04
2	CC037	M	Y	0.41	0.01
1	CC041XCC012	M	Y	0.49	0.09
1	CC041XCC012	M	Y	0.39	0.06
1	CC041XCC012	M	Y	0.32	0.02
1	CC041XCC012	M	Y	0.48	0.05
1	CC041XCC012	M	Y	0.61	0.04
1	CC041XCC012	F	Y	0.43	0.09
1	CC041XCC012	F	Y	0.5	0.11
1	CC041XCC012	F	Y	0.64	0.04
1	CC041XCC012	F	Y	0.41	0.04
1	CC041XCC012	F	Y	0.53	0.03
1	CC041XCC012	F	Y	0.6	0.12
1	CC041XCC012	F	Y	0.69	0.03
1	CC041XCC012	F	Y	0.5	0.01
2	CC041XCC012	M	Y	0.63	0.03
2	CC041XCC012	M	Y	1.06	0.03
2	CC041XCC012	F	Y	0.85	0.05
2	CC041XCC012	M	Y	0.78	0.05

Trial#	Strain	Sex	Inf?	PawAreaatPeakStanceinsq.cm_RH	PawAreaVariabilityatPeakStanceinsq.cm_LF
2	CC041XCC012	M	Y	0.95	0.04
2	CC041XCC012	M	Y	0.7	0.03
2	CC041XCC012	F	Y	0.78	0.01
2	CC041XCC012	F	Y	0.64	0.08
2	CC041XCC012	F	Y	0.84	0.08
2	CC041XCC012	F	Y	0.72	0.04
2	CC041XCC012	F	Y	0.73	0.02
2	CC041XCC012	F	Y	0.79	0.04
2	CC041XCC012	F	Y	1.04	0.04
0	CC051	F	Y	0.4	0.07
0	CC051	F	Y	0.37	0.02
0	CC051	F	Y	0.33	0.03
0	CC051	M	Y	0.44	0.05
1	CC051	F	Y	0.45	0.02
1	CC051	F	Y	0.68	0.03
1	CC051	F	Y	0.44	0.02
0	CC057	F	Y	0.5	0.02
0	CC057	F	Y	0.4	0.04
0	CC057	F	Y	0.14	0.05
0	CC057	M	Y	0.3	0.07
0	CC057	M	Y	0.2	0.03
0	CC057	M	Y	0.31	0.07
1	CC057	F	Y	0.59	0.06
1	CC057	F	Y	0.5	0.05
1	CC057	F	Y	0.28	0.1
1	CC057	F	Y	0.17	0.05
1	CC057	M	Y	0.71	0.05
1	CC057	M	Y	0.69	0.03
2	CC057	F	Y	0.17	0.05
0	CC078	F	Y	0.46	0.03
0	CC078	F	Y	0.38	0.03
0	CC078	F	Y	0.44	0.02
0	CC078	M	Y	0.42	0.03
1	CC078	F	Y	0.45	0.02
1	CC078	F	Y	0.37	0.02
1	CC078	M	Y	0.76	0.03
2	CC078	F	Y	0.3	0.06
2	CC078	M	Y	0.59	0.06
2	CC005	M	N	0.22	0.05
2	CC005	M	Y	0.27	0.06
2	CC015	F	N	0.58	0.23
2	CC015	M	N	0.96	0.04
2	CC015	M	Y	0.87	0.09
2	CC015	M	Y	0.79	0.23
2	CC017	F	N	0.59	0.08
2	CC017	M	N	0.66	0.05
2	CC017	F	Y	0.66	0.06
2	CC023	F	N	0.53	0.06

Trial#	Strain	Sex	Inf?	PawAreaatPeakStanceinsq.cm_RH	PawAreaVariabilityatPeakStanceinsq.cm_LF

Table S3. Raw DigiGait measurement data. Far left column indicates time point at which data was measured: T0 = pre-infection, T1 = 21dpi, and T3 = 89dpi. DigiGait parameters listed across the top indicate which limb is associated with the data, where appropriate: FL for left fore limb, FR for right fore limb, HL for left hind limb, and HR for right hind limb.

Table S3

Trial#	Strain	Sex	Inf?	PawAreaVariabilityatPeakStanceinsq.cm_LH	PawAreaVariabilityatPeakStanceinsq.cm_RF
1	CC002	F	N	0.02	0.03
1	CC025	F	N	0.08	0.02
1	CC012XCC032	F	N	0.04	0.03
2	CC012XCC032	F	N	0.04	0.03
1	CC012XCC032	M	N	0.02	0.01
2	CC012XCC032	M	N	0.04	0.02
1	CC013xCC041	F	N	0.16	0.04
2	CC013XCC041	F	N	0.02	0.02
1	CC013xCC041	M	N	0.05	0.02
2	CC013XCC041	M	N	0.03	0.03
1	CC032XCC013	F	N	0.14	0.03
2	CC032XCC013	F	N	0.08	0.06
1	CC041XCC012	F	N	0.13	0.16
1	CC041XCC012	F	N	0.04	0.06
2	CC041XCC012	F	N	0.04	0.1
1	CC032XCC013	M	N	0.15	0.1
2	CC032XCC013	M	N	0.07	0.06
1	CC041XCC012	M	N	0.04	0.04
2	CC041XCC012	M	N	0.09	0.04
0	CC012	F	N	0.1	0.11
2	CC012	F	N	0.08	0.07
0	CC012	M	N	0.19	0.04
2	CC012	M	N	0.04	0.05
0	CC057	F	N	0.08	0.04
1	CC057	F	N	0.03	0.03
0	CC057	M	N	0.06	0.02
1	CC057	M	N	0.03	0.02
0	CC078	F	N	0.01	0.03
1	CC078	F	N	0.06	0.05
2	CC078	F	N	0.15	0.07
0	CC078	M	N	0.04	0.02
2	CC078	M	N	0.09	0.07
0	CC002	F	N	0.08	0.09
1	CC002	F	N	0.02	0.03
0	CC002	M	N	0.08	0.06
1	CC002	M	N	0.16	0.15
2	CC002	M	N	0.13	0.03
0	CC006	F	N	0.16	0.04
1	CC006	F	N	0.21	0.16
2	CC006	F	N	0.13	0.06
0	CC006	M	N	0.06	0.11
0	CC023	F	N	0.07	0.04
1	CC023	F	N	0.13	0.13
2	CC023	F	N	0.04	0.05
0	CC023	M	N	0.02	0.04
1	CC023	M	N	0.04	0.02
0	CC027	F	N	0.03	0.05

Trial#	Strain	Sex	Inf?	PawAreaVariabilityatPeakStanceinsq.cm_LH	PawAreaVariabilityatPeakStanceinsq.cm_RF
1	CC027	F	N	0.12	0.1
2	CC027	F	N	0.05	0.02
0	CC027	M	N	0.07	0.07
1	CC027	M	N	0.09	0.08
1	CC027	M	N	0.1	0.07
2	CC027	M	N	0.06	0.09
0	CC005	F	N	0.04	0.02
1	CC005	F	N	0.02	0.06
2	CC005	F	N	0.05	0.04
0	CC011	F	N	0.24	0.1
1	CC011	F	N	0.18	0.15
2	CC011	F	N	0.07	0.04
1	CC017	F	N	0.07	0.1
0	CC005	M	N	0.07	0.02
1	CC005	M	N	0.11	0.05
2	CC005	M	N	0.12	0.05
0	CC011	M	N	0.12	0.01
1	CC011	M	N	0.02	0.01
2	CC011	M	N	0.21	0.09
0	CC017	M	N	0.03	0.04
1	CC017	M	N	0.03	0.06
1	CC006	F	N	0.11	0.09
0	CC037	F	N	0.05	0.04
0	CC051	F	N	0.01	0.01
1	CC051	F	N	0.07	0.02
1	CC006	M	N	0.21	0.08
0	CC037	M	N	0.07	0.05
1	CC037	M	N	0.07	0.14
0	CC005	F	N	0.04	0.04
1	CC005	F	N	0.11	0.05
2	CC005	F	N	0.04	0.06
0	CC011	F	N	0.08	0.08
1	CC011	F	N	0.04	0.02
2	CC011	F	N	0.02	0.01
0	CC011	M	N	0.05	0.03
1	CC011	M	N	0.04	0.02
2	CC011	M	N	0.02	0.01
1	CC037	M	N	0.1	0.08
2	CC037	M	N	0.05	0.03
1	CC051	M	N	0.07	0.04
2	CC051	M	N	0.03	0.08
0	CC027	F	N	0.04	0.02
1	CC027	F	N	0.15	0.06
2	CC027	F	N	0.11	0.07
0	CC015	M	N	0.06	0.02
1	CC015	M	N	0.12	0.11
2	CC015	M	N	0.04	0.03
0	CC027	M	N	0.03	0.02

Trial#	Strain	Sex	Inf?	PawAreaVariabilityatPeakStanceinsq.cm_LH	PawAreaVariabilityatPeakStanceinsq.cm_RF
1	CC027	M	N	0.06	0.04
2	CC027	M	N	0.08	0.02
0	CC015	F	N	0.06	0.05
1	CC015	F	N	0.11	0.06
0	CC017	F	N	0.03	0.03
0	CC023	F	N	0.11	0.08
1	CC023	F	N	0.05	0.06
0	CC005	M	N	0.11	0.06
1	CC005	M	N	0.14	0.02
0	CC015	M	N	0.08	0.09
1	CC015	M	N	0.19	0.16
0	CC017	M	N	0.13	0.05
1	CC017	M	N	0.12	0.08
0	CC023	M	N	0.06	0.03
1	CC023	M	N	0.09	0.08
0	CC051	M	N	0.16	0.05
0	CC002	F	Y	0.09	0.09
0	CC002	M	Y	0.04	0.05
1	CC002	M	Y	0.05	0.03
1	CC002	F	Y	0.03	0.05
1	CC002	F	Y	0.16	0.11
1	CC002	M	Y	0.05	0.03
1	CC002	M	Y	0.06	0.06
1	CC002	F	Y	0.03	0.05
1	CC002	F	Y	0.07	0.04
2	CC002	M	Y	0.07	0.05
2	CC002	F	Y	0.12	0.04
2	CC002	M	Y	0.04	0.06
0	CC005	F	Y	0.02	0.03
0	CC005	M	Y	0.06	0.04
0	CC005	M	Y	0.16	0.2
1	CC005	F	Y	0.08	0.02
1	CC005	M	Y	0.1	0.03
1	CC005	F	Y	0.13	0.05
1	CC005	M	Y	0.27	0.06
2	CC005	F	Y	0.09	0.05
2	CC005	M	Y	0.09	0.03
2	CC005	M	Y	0.14	0.09
2	CC005	M	Y	0.09	0.03
2	CC005	F	Y	0.04	0.02
0	CC006	F	Y	0.07	0.04
0	CC006	M	Y	0.03	0.03
0	CC006	F	Y	0.1	0.07
1	CC006	F	Y	0.06	0.05
1	CC006	M	Y	0.07	0.09
1	CC006	F	Y	0.17	0.06
1	CC006	M	Y	0.05	0.05
2	CC006	F	Y	0.2	0.05

Trial#	Strain	Sex	Inf?	PawAreaVariabilityatPeakStanceinsq.cm_LH	PawAreaVariabilityatPeakStanceinsq.cm_RF
2	CC006	M	Y	0.14	0.08
0	CC011	F	Y	0.32	0.22
0	CC011	M	Y	0.13	0.04
0	CC011	F	Y	0.02	0.02
0	CC011	F	Y	0.06	0.05
1	CC011	F	Y	0.02	0.01
1	CC011	M	Y	0.03	0.01
1	CC011	F	Y	0.05	0.02
1	CC011	M	Y	0.04	0.05
2	CC011	F	Y	0.02	0.04
2	CC011	M	Y	0.19	0.04
2	CC011	F	Y	0.03	0.02
2	CC011	M	Y	0.07	0.01
0	CC012	M	Y	0.12	0.03
0	CC012	M	Y	0.07	0.09
0	CC012	M	Y	0.05	0.02
0	CC012	M	Y	0.09	0.04
0	CC012	M	Y	0.06	0.03
0	CC012	F	Y	0.13	0.08
0	CC012	F	Y	0.05	0.03
0	CC012	F	Y	0.12	0.04
0	CC012	F	Y	0.25	0.17
0	CC012	F	Y	0.11	0.06
2	CC012	M	Y	0.16	0.05
2	CC012	M	Y	0.07	0.03
2	CC012	M	Y	0.04	0.28
2	CC012	M	Y	0.18	0.02
2	CC012	M	Y	0.05	0.03
2	CC012	F	Y	0.06	0.01
2	CC012	F	Y	0.05	0.02
2	CC012	F	Y	0.04	0.07
2	CC012	F	Y	0.14	0.05
2	CC012	F	Y	0.06	0.11
1	CC012XCC032	F	Y	0.16	0.14
1	CC012xCC032	F	Y	0.05	0.03
1	CC012XCC032	M	Y	0.05	0.03
1	CC012xCC032	M	Y	0.1	0.04
1	CC012XCC032	M	Y	0.12	0.09
1	CC012xCC032	M	Y	0.06	0.06
2	CC012XCC032	F	Y	0.09	0.04
2	CC012XCC032	F	Y	0.05	0.01
2	CC012XCC032	M	Y	0.09	0.07
2	CC012XCC032	M	Y	0.14	0.02
2	CC012XCC032	M	Y	0.14	0.02
2	CC012XCC032	M	Y	0.06	0.05
1	CC013xCC041	F	Y	0.09	0.06
1	CC013xCC041	F	Y	0.11	0.04
1	CC013xCC041	F	Y	0.04	0.05

Trial#	Strain	Sex	Inf?	PawAreaVariabilityatPeakStanceinsq.cm_LH	PawAreaVariabilityatPeakStanceinsq.cm_RF
1	CC013xCC041	M	Y	0.1	0.1
1	CC013xCC041	M	Y	0.14	0.04
2	CC013XCC041	F	Y	0.03	0.05
2	CC013XCC041	F	Y	0.14	0.29
2	CC013XCC041	F	Y	0.15	0.05
2	CC013XCC041	M	Y	0.12	0.03
2	CC013XCC041	M	Y	0.12	0.09
0	CC015	F	Y	0.08	0.04
0	CC015	M	Y	0.08	0.04
0	CC015	M	Y	0.05	0.04
1	CC015	F	Y	0.04	0.02
1	CC015	M	Y	0.11	0.07
1	CC015	M	Y	0.12	0.18
1	CC015	M	Y	0.08	0.09
2	CC015	F	Y	0.04	0.04
0	CC017	F	Y	0.04	0.04
0	CC017	M	Y	0.11	0.12
0	CC017	F	Y	0.13	0.14
0	CC017	M	Y	0.11	0.13
1	CC017	M	Y	0.12	0.09
1	CC017	F	Y	0.13	0.11
0	CC023	M	Y	0.06	0.02
0	CC023	F	Y	0.03	0.02
0	CC023	F	Y	0.09	0.06
0	CC023	M	Y	0.02	0.09
1	CC023	M	Y	0.06	0.05
1	CC023	F	Y	0.08	0.12
1	CC023	M	Y	0.07	0.06
1	CC023	M	Y	0.06	0.02
2	CC023	M	Y	0.18	0.01
2	CC023	F	Y	0.09	0.09
2	CC023	M	Y	0.06	0.02
0	CC025	M	Y	0.1	0.02
0	CC025	F	Y	0.08	0.13
0	CC025	M	Y	0.01	0.01
1	CC025	M	Y	0.09	0.02
1	CC025	F	Y	0.13	0.04
1	CC025	F	Y	0.05	0.02
1	CC025	F	Y	0.06	0.03
2	CC025	F	Y	0.04	0.02
2	CC025	M	Y	0.08	0.01
0	CC027	F	Y	0.06	0.03
0	CC027	M	Y	0.03	0.06
1	CC027	M	Y	0.1	0.02
1	CC027	M	Y	0.1	0.02
1	CC027	F	Y	0.19	0.09
1	CC027	F	Y	0.11	0.05
1	CC027	M	Y	0.02	0.02

Trial#	Strain	Sex	Inf?	PawAreaVariabilityatPeakStanceinsq.cm_LH	PawAreaVariabilityatPeakStanceinsq.cm_RF
1	CC027	M	Y	0.01	0.01
2	CC027	F	Y	0.09	0.09
2	CC027	M	Y	0.11	0.04
2	CC027	F	Y	0.13	0.1
2	CC027	M	Y	0.08	0.02
1	CC032XCC013	M	Y	0.05	0.06
1	CC032XCC013	M	Y	0.11	0.09
1	CC032XCC013	M	Y	0.12	0.02
1	CC032XCC013	F	Y	0.05	0.09
1	CC032XCC013	F	Y	0.02	0.07
1	CC032XCC013	F	Y	0.12	0.05
1	CC032XCC013	F	Y	0.06	0.04
1	CC032XCC013	F	Y	0.12	0.02
2	CC032XCC013	M	Y	0.04	0.03
2	CC032XCC013	M	Y	0.17	0.08
2	CC032XCC013	M	Y	0.18	0.13
2	CC032XCC013	M	Y	0.05	0.09
2	CC032XCC013	F	Y	0.05	0.09
2	CC032XCC013	F	Y	0.08	0.13
2	CC032XCC013	F	Y	0.15	0.04
2	CC032XCC013	F	Y	0.04	0.05
0	CC037	F	Y	0.05	0.05
0	CC037	M	Y	0.07	0.1
0	CC037	M	Y	0.03	0.03
0	CC037	M	Y	0.1	0.07
1	CC037	F	Y	0.15	0.07
1	CC037	M	Y	0.05	0.02
1	CC037	M	Y	0.05	0.06
1	CC037	M	Y	0.08	0.13
2	CC037	M	Y	0.03	0.04
2	CC037	M	Y	0.02	0.01
1	CC041XCC012	M	Y	0.16	0.1
1	CC041XCC012	M	Y	0.02	0.02
1	CC041XCC012	M	Y	0.12	0.11
1	CC041XCC012	M	Y	0.07	0.12
1	CC041XCC012	M	Y	0.04	0.05
1	CC041XCC012	F	Y	0.07	0.03
1	CC041XCC012	F	Y	0.04	0.07
1	CC041XCC012	F	Y	0.02	0.03
1	CC041XCC012	F	Y	0.05	0.08
1	CC041XCC012	F	Y	0.09	0.06
1	CC041XCC012	F	Y	0.08	0.07
1	CC041XCC012	F	Y	0.08	0.02
1	CC041XCC012	F	Y	0.16	0.07
2	CC041XCC012	M	Y	0.05	0.02
2	CC041XCC012	M	Y	0.05	0.08
2	CC041XCC012	F	Y	0.11	0.09
2	CC041XCC012	M	Y	0.07	0.04

Trial#	Strain	Sex	Inf?	PawAreaVariabilityatPeakStanceinsq.cm_LH	PawAreaVariabilityatPeakStanceinsq.cm_RF
2	CC041XCC012	M	Y	0.05	0.04
2	CC041XCC012	M	Y	0.07	0.04
2	CC041XCC012	F	Y	0.09	0.01
2	CC041XCC012	F	Y	0.16	0.05
2	CC041XCC012	F	Y	0.05	0.04
2	CC041XCC012	F	Y	0.03	0.03
2	CC041XCC012	F	Y	0.05	0.03
2	CC041XCC012	F	Y	0.19	0.04
2	CC041XCC012	F	Y	0.16	0.2
0	CC051	F	Y	0.02	0.05
0	CC051	F	Y	0.04	0.06
0	CC051	F	Y	0.02	0.01
0	CC051	M	Y	0.04	0.03
1	CC051	F	Y	0.02	0.02
1	CC051	F	Y	0.02	0.02
1	CC051	F	Y	0.05	0.01
0	CC057	F	Y	0.05	0.04
0	CC057	F	Y	0.06	0.03
0	CC057	F	Y	0.05	0.04
0	CC057	M	Y	0.04	0.02
0	CC057	M	Y	0.08	0.06
0	CC057	M	Y	0.12	0.07
1	CC057	F	Y	0.03	0.03
1	CC057	F	Y	0.13	0.07
1	CC057	F	Y	0.13	0.09
1	CC057	F	Y	0.08	0.05
1	CC057	M	Y	0.17	0.02
1	CC057	M	Y	0.08	0.03
2	CC057	F	Y	0.08	0.05
0	CC078	F	Y	0.07	0.05
0	CC078	F	Y	0.16	0.03
0	CC078	F	Y	0.03	0.02
0	CC078	M	Y	0.08	0.02
1	CC078	F	Y	0.02	0.02
1	CC078	F	Y	0.06	0.02
1	CC078	M	Y	0.04	0.04
2	CC078	F	Y	0.07	0.07
2	CC078	M	Y	0.05	0.08
2	CC005	M	N	0.15	0.06
2	CC005	M	Y	0.09	0.06
2	CC015	F	N	0.3	0.07
2	CC015	M	N	0.04	0.02
2	CC015	M	Y	0.12	0.11
2	CC015	M	Y	0.13	0.11
2	CC017	F	N	0.17	0.09
2	CC017	M	N	0.12	0.04
2	CC017	F	Y	0.18	0.07
2	CC023	F	N	0.18	0.04

Trial#	Strain	Sex	Inf?	PawAreaVariabilityatPeakStanceinsq.cm_LH	PawAreaVariabilityatPeakStanceinsq.cm_RF

Table S3. Raw DigiGait measurement data. Far left column indicates time point at which data was measured: T0 = pre-infection, T1 = 21dpi, and T3 = 89dpi. DigiGait parameters listed across the top indicate which limb is associated with the data, where appropriate: FL for left fore limb, FR for right fore limb, HL for left hind limb, and HR for right hind limb.

Table S3

Trial#	Strain	Sex	Inf?	PawAreaVariabilityatPeakStanceinsq.cm_RH	PawDrag_LH	PawDrag_RH
1	CC002	F	N	0.02	-0.989	-0.915
1	CC025	F	N	0.04	-5.54	-5.036
1	CC012XCC032	F	N	0.04	0.621	-0.593
2	CC012XCC032	F	N	0.07	-0.83	-2.887
1	CC012XCC032	M	N	0.05	-2.95	-2.821
2	CC012XCC032	M	N	0.05	0.244	-3.053
1	CC013xCC041	F	N	0.18	-3.264	-2.728
2	CC013XCC041	F	N	0.07	-2.232	-4.005
1	CC013xCC041	M	N	0.02	0.441	-1.121
2	CC013XCC041	M	N	0.22	-5.441	-5.203
1	CC032XCC013	F	N	0.08	-2.013	0.304
2	CC032XCC013	F	N	0.1	-1.866	-1.996
1	CC041XCC012	F	N	0.15	-2.249	-2.757
1	CC041XCC012	F	N	0.05	-0.303	-2.242
2	CC041XCC012	F	N	0.21	-3.976	-4.431
1	CC032XCC013	M	N	0.11	-3.362	-2.676
2	CC032XCC013	M	N	0.12	-3.677	-4.936
1	CC041XCC012	M	N	0.06	-1.316	-1.419
2	CC041XCC012	M	N	0.04	-2.804	-2.311
0	CC012	F	N	0.08	-2.624	-2.676
2	CC012	F	N	0.09	-1.287	0.841
0	CC012	M	N	0.12	-0.507	0.362
2	CC012	M	N	0.13	-0.877	-2.781
0	CC057	F	N	0.19	-1.978	-0.145
1	CC057	F	N	0.05	-5.201	-1.427
0	CC057	M	N	0.09	-1.287	-0.432
1	CC057	M	N	0.04	-3.214	-3.833
0	CC078	F	N	0.09	-0.07	-1.629
1	CC078	F	N	0.15	-2.093	-2.913
2	CC078	F	N	0.09	-1.881	-1.162
0	CC078	M	N	0.09	-1.352	-2.09
2	CC078	M	N	0.11	-3.053	-5.324
0	CC002	F	N	0.11	-1.243	-2.052
1	CC002	F	N	0.02	-0.989	-0.915
0	CC002	M	N	0.09	-3.954	-2.674
1	CC002	M	N	0.14	-6.18	0.294
2	CC002	M	N	0.14	-1.408	-2.838
0	CC006	F	N	0.04	-3.966	-1.105
1	CC006	F	N	0.02	-3.382	-1.409
2	CC006	F	N	0.13	-1.099	-5.151
0	CC006	M	N	0.11	-1.895	-0.353
0	CC023	F	N	0.03	-1.266	-2.068
1	CC023	F	N	0.12	-0.81	-4.502
2	CC023	F	N	0.1	-3.526	-4.211
0	CC023	M	N	0.07	-1.122	-2.065
1	CC023	M	N	0.05	-2.184	-3.459
0	CC027	F	N	0.04	-0.092	-0.611

Trial#	Strain	Sex	Inf?	PawAreaVariabilityatPeakStanceinsq.cm_RH	PawDrag_LH	PawDrag_RH
1	CC027	F	N	0.06	-2.815	-1.401
2	CC027	F	N	0.1	-3.354	-1.131
0	CC027	M	N	0.07	-0.874	0.177
1	CC027	M	N	0.12	-1.195	1.338
1	CC027	M	N	0.11	-0.563	-3.99
2	CC027	M	N	0.06	-5.04	-9.219
0	CC005	F	N	0.07	-1.396	-1.182
1	CC005	F	N	0.05	-0.07	-1.612
2	CC005	F	N	0.04	-0.085	-0.748
0	CC011	F	N	0.26	-0.892	-1.195
1	CC011	F	N	0.16	-3.428	-3.982
2	CC011	F	N	0.03	-3.658	-3.417
1	CC017	F	N	0.15	-2.183	-0.414
0	CC005	M	N	0.03	-3.302	-3.761
1	CC005	M	N	0.08	-2.14	-0.198
2	CC005	M	N	0.15	-1.653	-0.365
0	CC011	M	N	0.11	-4.847	-4.816
1	CC011	M	N	0.01	-3.577	-4.231
2	CC011	M	N	0.16	1.629	-3.555
0	CC017	M	N	0.03	-0.427	-0.944
1	CC017	M	N	0.18	-1.677	-2.513
1	CC006	F	N	0.17	-0.992	-1.284
0	CC037	F	N	0.03	-2.11	-4.189
0	CC051	F	N	0.05	-2.073	-3.29
1	CC051	F	N	0.08	-1.004	-2.739
1	CC006	M	N	0.06	-1.95	-1.397
0	CC037	M	N	0.03	-3.216	-2.035
1	CC037	M	N	0.14	-0.807	-3.512
0	CC005	F	N	0.11	-0.92	-0.774
1	CC005	F	N	0.06	-1.733	-4.808
2	CC005	F	N	0.06	-0.87	0.088
0	CC011	F	N	0.03	-3.363	-2.427
1	CC011	F	N	0.05	-11.306	-7.704
2	CC011	F	N	0.02	-7.082	-5.887
0	CC011	M	N	0.09	-3.838	-5.587
1	CC011	M	N	0.04	-6.22	-7.984
2	CC011	M	N	0.02	-4.602	-4.19
1	CC037	M	N	0.07	-4.339	-5.575
2	CC037	M	N	0.06	-1.866	-3.61
1	CC051	M	N	0.04	-3.864	-5.872
2	CC051	M	N	0.04	-4.585	-3.881
0	CC027	F	N	0.03	-2.108	0.637
1	CC027	F	N	0.05	-1.904	-3.298
2	CC027	F	N	0.12	-3.46	-2.125
0	CC015	M	N	0.06	-3.306	-1.938
1	CC015	M	N	0.16	-1.862	-3.138
2	CC015	M	N	0.07	-2.526	-3.936
0	CC027	M	N	0.03	-0.702	-1.811

Trial#	Strain	Sex	Inf?	PawAreaVariabilityatPeakStanceinsq.cm_RH	PawDrag_LH	PawDrag_RH
1	CC027	M	N	0.18	-2.581	-2.645
2	CC027	M	N	0.1	-3.967	-3.225
0	CC015	F	N	-4.096	-2.632	0.31
1	CC015	F	N	0.09	-2.135	-1.955
0	CC017	F	N	0.09	-1.771	-5.491
0	CC023	F	N	0.08	-5.164	-5.927
1	CC023	F	N	0.08	-1.199	-1.786
0	CC005	M	N	0.09	-1.284	-2.416
1	CC005	M	N	0.03	-3.625	-1.797
0	CC015	M	N	0.03	-4.686	-5.481
1	CC015	M	N	0.09	-3.584	0.283
0	CC017	M	N	0.05	-2.59	-5.149
1	CC017	M	N	0.13	-3.181	-2.415
0	CC023	M	N	0.03	-0.036	-1.988
1	CC023	M	N	0.2	-3.124	-0.061
0	CC051	M	N	0.04	-3.632	-3.091
0	CC002	F	Y	0.1	-1.141	-2.695
0	CC002	M	Y	0.03	-2.449	-4.376
1	CC002	M	Y	0.05	-1.41	-2.019
1	CC002	F	Y	0.04	-0.694	-2.658
1	CC002	F	Y	0.15	-1.903	-1.705
1	CC002	M	Y	0.05	-1.41	-2.019
1	CC002	M	Y	0.06	-2.428	-0.869
1	CC002	F	Y	0.04	-0.694	-2.658
1	CC002	F	Y	0.06	-1.249	-0.389
2	CC002	M	Y	0.03	-1.674	-0.961
2	CC002	F	Y	0.04	-3.282	-2.641
2	CC002	M	Y	0.15	-0.945	-2.819
0	CC005	F	Y	0.08	-2.813	-2.982
0	CC005	M	Y	0.06	-1.287	-1.859
0	CC005	M	Y	0.09	-4.338	-4.173
1	CC005	F	Y	0.11	-1.439	-2.995
1	CC005	M	Y	0.07	-1.914	0.235
1	CC005	F	Y	0.07	-2.104	-6.095
1	CC005	M	Y	0.18	-2.903	-1.592
2	CC005	F	Y	0.16	-1.179	-0.983
2	CC005	M	Y	0.05	-6.9	-2.064
2	CC005	M	Y	0.17	-2.504	-2.873
2	CC005	M	Y	0.05	-6.9	-2.064
2	CC005	F	Y	0.09	-0.997	-1.973
0	CC006	F	Y	0.07	-2.25	-2.102
0	CC006	M	Y	0.02	-0.634	0.201
0	CC006	F	Y	0.09	-2.332	-1.744
1	CC006	F	Y	0.08	-0.38	-1.379
1	CC006	M	Y	0.09	-1.01	1.88
1	CC006	F	Y	0.17	-2.718	-2.388
1	CC006	M	Y	0.06	-2.354	-2.707
2	CC006	F	Y	0.11	-2.111	-4.101

Trial#	Strain	Sex	Inf?	PawAreaVariabilityatPeakStanceinsq.cm_RH	PawDrag_LH	PawDrag_RH
2	CC006	M	Y	0.14	-2.838	-1.108
0	CC011	F	Y	0.06	-0.647	-2.546
0	CC011	M	Y	0.22	-3.022	-4.434
0	CC011	F	Y	0.01	-3.035	-2.103
0	CC011	F	Y	0.15	-2.907	-3.949
1	CC011	F	Y	0.02	-4.238	-4.383
1	CC011	M	Y	0.04	-5.433	-2.899
1	CC011	F	Y	0.05	-8.519	-6.536
1	CC011	M	Y	0.11	-8.084	-4.785
2	CC011	F	Y	0.02	-6.568	-4.79
2	CC011	M	Y	0.11	-1.403	-2.29
2	CC011	F	Y	0.03	-4.808	-5.052
2	CC011	M	Y	0.05	-3.846	-3.978
0	CC012	M	Y	0.12	-0.749	-3.628
0	CC012	M	Y	0.16	-4.957	-2.608
0	CC012	M	Y	0.04	-1.744	-1.033
0	CC012	M	Y	0.15	-0.275	-2.23
0	CC012	M	Y	0.14	-2.243	-2.309
0	CC012	F	Y	0.04	-5.186	-2.927
0	CC012	F	Y	0.08	-4.97	-3.023
0	CC012	F	Y	0.12	-1.68	-1.338
0	CC012	F	Y	0.22	-5.266	-4.752
0	CC012	F	Y	0.24	-2.548	-3.972
2	CC012	M	Y	0.15	-4.924	-2.805
2	CC012	M	Y	0.15	-3.834	-2.235
2	CC012	M	Y	0.08	-3.092	-2.84
2	CC012	M	Y	0.13	-1.119	-2.853
2	CC012	M	Y	0.13	-5.381	-3.529
2	CC012	F	Y	0.1	-5.617	-3.897
2	CC012	F	Y	0.01	-5.506	-6.185
2	CC012	F	Y	0.13	-2.615	-2.261
2	CC012	F	Y	0.06	-6.345	-6.264
2	CC012	F	Y	0.07	-3.751	-6.737
1	CC012XCC032	F	Y	0.13	-1.171	1.211
1	CC012xCC032	F	Y	0.04	0.024	-0.489
1	CC012XCC032	M	Y	0.04	-2.778	-1.375
1	CC012xCC032	M	Y	0.09	-2.353	-1.31
1	CC012XCC032	M	Y	0.07	-0.979	-0.37
1	CC012xCC032	M	Y	0.11	-0.943	-1.339
2	CC012XCC032	F	Y	0.07	-3.301	-2.272
2	CC012XCC032	F	Y	0.11	-3.873	-4.226
2	CC012XCC032	M	Y	0.23	-3.501	-4.138
2	CC012XCC032	M	Y	0.08	-2.81	-2.793
2	CC012XCC032	M	Y	0.07	-5.115	-6.392
2	CC012XCC032	M	Y	0.08	-0.565	-3.064
1	CC013xCC041	F	Y	0.13	-4.054	-3.328
1	CC013xCC041	F	Y	0.08	-1.579	-3.499
1	CC013xCC041	F	Y	0.05	0.191	-1.149

Trial#	Strain	Sex	Inf?	PawAreaVariabilityatPeakStanceinsq.cm_RH	PawDrag_LH	PawDrag_RH
1	CC013xCC041	M	Y	0.1	-2.273	-3.632
1	CC013xCC041	M	Y	0.1	-2.227	-2.961
2	CC013XCC041	F	Y	0.05	-7.033	-3.833
2	CC013XCC041	F	Y	0.18	-9.703	-9.912
2	CC013XCC041	F	Y	0.13	-6.058	-3.964
2	CC013XCC041	M	Y	0.08	-7.926	-4.022
2	CC013XCC041	M	Y	0.08	-0.771	-0.79
0	CC015	F	Y	0.03	-1.833	-3.869
0	CC015	M	Y	0.06	-1.953	-3.273
0	CC015	M	Y	0.08	-6.314	-6.286
1	CC015	F	Y	0.05	-3.46	-3.761
1	CC015	M	Y	0.09	-0.719	-3.253
1	CC015	M	Y	0.22	-2.934	-1.223
1	CC015	M	Y	0.09	-2.453	-2.803
2	CC015	F	Y	0.05	-6.184	-6.092
0	CC017	F	Y	0.06	-2.985	-3.704
0	CC017	M	Y	0.12	-2.794	-4.675
0	CC017	F	Y	0.18	-2.177	-4.193
0	CC017	M	Y	0.12	-4.764	-3.413
1	CC017	M	Y	0.1	-4.596	-3.356
1	CC017	F	Y	0.12	-2.919	-1.51
0	CC023	M	Y	0.02	-1.582	-3.199
0	CC023	F	Y	0.03	-1.081	-0.967
0	CC023	F	Y	0.06	-4.509	-5.59
0	CC023	M	Y	0.04	-2.057	-4.001
1	CC023	M	Y	0.1	-0.766	-2.194
1	CC023	F	Y	0.12	-0.814	-1.038
1	CC023	M	Y	0.08	-1.921	-3.198
1	CC023	M	Y	0	-1.714	-1.622
2	CC023	M	Y	0.01	-1.308	-1.002
2	CC023	F	Y	0.03	-1.983	-2.013
2	CC023	M	Y	0.03	-3.325	-1.669
0	CC025	M	Y	0.05	-4.61	-4.442
0	CC025	F	Y	0.07	-1.582	-3.373
0	CC025	M	Y	0.02	-2.07	-1.621
1	CC025	M	Y	0.04	-4.316	-4.301
1	CC025	F	Y	0.13	-7.316	-4.862
1	CC025	F	Y	0.04	-5.752	-4.32
1	CC025	F	Y	0.14	-3.388	-3.738
2	CC025	F	Y	0.04	-3.786	-1.68
2	CC025	M	Y	0.03	-1.168	-1.853
0	CC027	F	Y	0.09	0.171	-3.057
0	CC027	M	Y	0.01	-0.96	-0.725
1	CC027	M	Y	0.08	-0.191	-1.919
1	CC027	M	Y	0.08	-0.191	-1.919
1	CC027	F	Y	0.1	-6.579	-2.968
1	CC027	F	Y	0.07	-2.752	0.109
1	CC027	M	Y	0.15	-3.205	-3.272

Trial#	Strain	Sex	Inf?	PawAreaVariabilityatPeakStanceinsq.cm_RH	PawDrag_LH	PawDrag_RH
1	CC027	M	Y	0.02	-2.322	-1.635
2	CC027	F	Y	0.03	-1.983	-2.013
2	CC027	M	Y	0.05	-1.522	-1.153
2	CC027	F	Y	0.08	-5.023	-2.594
2	CC027	M	Y	0.04	-0.973	-1.89
1	CC032XCC013	M	Y	0.12	-3.733	-4.293
1	CC032XCC013	M	Y	0.08	-6.095	-3.156
1	CC032XCC013	M	Y	0.12	-3.858	-1.968
1	CC032XCC013	F	Y	0.11	-0.807	-1.039
1	CC032XCC013	F	Y	0.09	-3.068	-2.915
1	CC032XCC013	F	Y	0.03	-3.447	-2.802
1	CC032XCC013	F	Y	0.03	-3.447	-2.867
1	CC032XCC013	F	Y	0.07	-5.027	-1.499
2	CC032XCC013	M	Y	0.06	-6.187	-7.95
2	CC032XCC013	M	Y	0.23	-5.483	-5.915
2	CC032XCC013	M	Y	0.18	-6.292	-3.654
2	CC032XCC013	M	Y	0.16	-4.61	-3.697
2	CC032XCC013	F	Y	0.04	-5.515	-5.351
2	CC032XCC013	F	Y	0.08	-6.862	-4.996
2	CC032XCC013	F	Y	0.1	-5.775	-4.741
2	CC032XCC013	F	Y	0.06	-5.484	-2.383
0	CC037	F	Y	0.08	-0.61	-1.413
0	CC037	M	Y	0.05	-1.366	-3.314
0	CC037	M	Y	0.03	-1.685	-5.531
0	CC037	M	Y	0.12	-0.008	-0.963
1	CC037	F	Y	0.05	-2.109	-0.942
1	CC037	M	Y	0.02	-1.954	-1.869
1	CC037	M	Y	0.04	-1.649	-1.984
1	CC037	M	Y	0.05	-1.929	-1.692
2	CC037	M	Y	0.03	-2.22	-4.475
2	CC037	M	Y	0.02	-1.509	-3.451
1	CC041XCC012	M	Y	0.16	-1.719	-3.394
1	CC041XCC012	M	Y	0.02	-2.907	-4.372
1	CC041XCC012	M	Y	0.14	-2.261	-1.165
1	CC041XCC012	M	Y	0.13	-3.346	-3.969
1	CC041XCC012	M	Y	0.11	-3.738	-2.32
1	CC041XCC012	F	Y	0.13	-3.832	-1.903
1	CC041XCC012	F	Y	0.12	-2.612	-3.884
1	CC041XCC012	F	Y	0.17	-2.294	-0.987
1	CC041XCC012	F	Y	0.09	-7.182	-4.025
1	CC041XCC012	F	Y	0.13	-0.953	-1.629
1	CC041XCC012	F	Y	0.13	-4.774	-2.996
1	CC041XCC012	F	Y	0.06	-5.713	-5.958
1	CC041XCC012	F	Y	0.21	-1.651	-1.462
2	CC041XCC012	M	Y	0.07	-6.312	-4.118
2	CC041XCC012	M	Y	0.23	-3.831	-2.625
2	CC041XCC012	F	Y	0.05	-2.494	-0.885
2	CC041XCC012	M	Y	0.04	-2.312	-3.145

Trial#	Strain	Sex	Inf?	PawAreaVariabilityatPeakStanceinsq.cm_RH	PawDrag_LH	PawDrag_RH
2	CC041XCC012	M	Y	0.1	-4.684	-4.604
2	CC041XCC012	M	Y	0.05	-1.313	-3.512
2	CC041XCC012	F	Y	0.14	-4.553	-3.316
2	CC041XCC012	F	Y	0.17	-2.224	-3.021
2	CC041XCC012	F	Y	0.04	-6.407	-5.979
2	CC041XCC012	F	Y	0.05	-3.122	-3.546
2	CC041XCC012	F	Y	0.07	-4.781	-5.581
2	CC041XCC012	F	Y	0.06	-3.219	-2.081
2	CC041XCC012	F	Y	0.11	-8.305	-4.416
0	CC051	F	Y	0.03	-2.475	-2.244
0	CC051	F	Y	0.03	-2.329	-2.722
0	CC051	F	Y	0.03	-1.428	-2.044
0	CC051	M	Y	0.04	-4.111	-6.001
1	CC051	F	Y	0.03	-2.448	-1.875
1	CC051	F	Y	0.05	-3.831	-6.036
1	CC051	F	Y	0.02	-2.843	-3.672
0	CC057	F	Y	0.03	-0.802	-0.902
0	CC057	F	Y	0.09	0.414	0.573
0	CC057	F	Y	0.04	-0.005	0.118
0	CC057	M	Y	0.04	-0.33	-1.839
0	CC057	M	Y	0.07	0.177	-0.788
0	CC057	M	Y	0.08	0.197	-0.077
1	CC057	F	Y	0.04	-2.33	-2.272
1	CC057	F	Y	0.08	-1.286	-2.81
1	CC057	F	Y	0.07	-2.108	-1.537
1	CC057	F	Y	0.06	-0.784	-1.103
1	CC057	M	Y	0.03	-2.348	-0.983
1	CC057	M	Y	0.06	-8.898	-4.707
2	CC057	F	Y	0.06	-0.784	-1.103
0	CC078	F	Y	0.11	-2.231	-0.642
0	CC078	F	Y	0.05	-1.499	-2.077
0	CC078	F	Y	0.03	-0.405	-0.543
0	CC078	M	Y	0.05	-3.466	-3.572
1	CC078	F	Y	0.02	-1.162	-1.684
1	CC078	F	Y	0.04	-0.709	-1.579
1	CC078	M	Y	0.05	-3.576	-8.186
2	CC078	F	Y	0.09	-4.35	-3.304
2	CC078	M	Y	0.07	-4.111	-7.226
2	CC005	M	N	0.12	-2.256	-1.298
2	CC005	M	Y	0.11	-2.3	-3.122
2	CC015	F	N	0.3	-8.445	-4.473
2	CC015	M	N	0.06	-7.258	-7.922
2	CC015	M	Y	0.18	-8.476	-6.682
2	CC015	M	Y	0.26	-4.493	-5.927
2	CC017	F	N	0.1	-3.537	-0.864
2	CC017	M	N	0.08	-5.951	-4.652
2	CC017	F	Y	0.19	-3.46	-3.378
2	CC023	F	N	0.06	-3.968	-3.574

Trial#	Strain	Sex	Inf?	PawAreaVariabilityatPeakStanceinsq.cm_RH	PawDrag_LH	PawDrag_RH

Table S3. Raw DigiGait measurement data. Far left column indicates time point at which data was measured: T0 = pre-infection, T1 = 21dpi, and T3 = 89dpi. DigiGait parameters listed across the top indicate which limb is associated with the data, where appropriate: FL for left fore limb, FR for right fore limb, HL for left hind limb, and HR for right hind limb.

Table S3

Trial#	Strain	Sex	Inf?	PawPlacementPositioning[PPP]_LF	PawPlacementPositioning[PPP]_LH
1	CC002	F	N	0.5	0.5
1	CC025	F	N	0.32	0.32
1	CC012XCC032	F	N	0.62	0.62
2	CC012XCC032	F	N	0.72	0.72
1	CC012XCC032	M	N	0.61	0.61
2	CC012XCC032	M	N	0.44	0.44
1	CC013xCC041	F	N	0.52	0.52
2	CC013XCC041	F	N	0.51	0.51
1	CC013xCC041	M	N	0.22	0.22
2	CC013XCC041	M	N	0.2	0.2
1	CC032XCC013	F	N	0.65	0.65
2	CC032XCC013	F	N	0.43	0.43
1	CC041XCC012	F	N	0.01	0.01
1	CC041XCC012	F	N	-0.1	-0.1
2	CC041XCC012	F	N	1.57	1.57
1	CC032XCC013	M	N	0.12	0.12
2	CC032XCC013	M	N	1.16	1.16
1	CC041XCC012	M	N	1.13	1.13
2	CC041XCC012	M	N	1.21	1.21
0	CC012	F	N	0.11	0.11
2	CC012	F	N	0.69	0.69
0	CC012	M	N	0.24	0.24
2	CC012	M	N	0.37	0.37
0	CC057	F	N	0.19	0.19
1	CC057	F	N	0.18	0.18
0	CC057	M	N	0.54	0.54
1	CC057	M	N	0.72	0.72
0	CC078	F	N	0.17	0.17
1	CC078	F	N	0.19	0.19
2	CC078	F	N	-0.04	-0.04
0	CC078	M	N	0.07	0.07
2	CC078	M	N	0.2	0.2
0	CC002	F	N	0.17	0.17
1	CC002	F	N	0.5	0.5
0	CC002	M	N	0.13	0.13
1	CC002	M	N	-0.03	-0.03
2	CC002	M	N	0.85	0.85
0	CC006	F	N	0.32	0.32
1	CC006	F	N	0.36	0.36
2	CC006	F	N	0.93	0.93
0	CC006	M	N	0.08	0.08
0	CC023	F	N	0.35	0.35
1	CC023	F	N	-0.09	-0.09
2	CC023	F	N	0	0
0	CC023	M	N	0.49	0.49
1	CC023	M	N	0.43	0.43
0	CC027	F	N	0.61	0.61

Trial#	Strain	Sex	Inf?	PawPlacementPositioning[PPP]_LF	PawPlacementPositioning[PPP]_LH
1	CC027	F	N	0.32	0.32
2	CC027	F	N	0.58	0.58
0	CC027	M	N	0.07	0.07
1	CC027	M	N	0.18	0.18
1	CC027	M	N	0.06	0.06
2	CC027	M	N	0.28	0.28
0	CC005	F	N	0.33	0.33
1	CC005	F	N	0.68	0.68
2	CC005	F	N	-0.29	-0.29
0	CC011	F	N	0.08	0.08
1	CC011	F	N	0.43	0.43
2	CC011	F	N	0.49	0.49
1	CC017	F	N	0.61	0.61
0	CC005	M	N	0.5	0.5
1	CC005	M	N	-0.05	-0.05
2	CC005	M	N	0.59	0.59
0	CC011	M	N	0.02	0.02
1	CC011	M	N	0.12	0.12
2	CC011	M	N	0.11	0.11
0	CC017	M	N	0.41	0.41
1	CC017	M	N	1.31	1.31
1	CC006	F	N	-0.01	-0.01
0	CC037	F	N	0.49	0.49
0	CC051	F	N	0.13	0.13
1	CC051	F	N	0.3	0.3
1	CC006	M	N	0.52	0.52
0	CC037	M	N	0.38	0.38
1	CC037	M	N	-0.22	-0.22
0	CC005	F	N	0.3	0.3
1	CC005	F	N	0.89	0.89
2	CC005	F	N	0.55	0.55
0	CC011	F	N	0.09	0.09
1	CC011	F	N	-0.24	-0.24
2	CC011	F	N	0.02	0.02
0	CC011	M	N	0.06	0.06
1	CC011	M	N	0.54	0.54
2	CC011	M	N	1.1	1.1
1	CC037	M	N	0.66	0.66
2	CC037	M	N	-0.03	-0.03
1	CC051	M	N	0.5	0.5
2	CC051	M	N	0.48	0.48
0	CC027	F	N	0.02	0.02
1	CC027	F	N	0.38	0.38
2	CC027	F	N	0.55	0.55
0	CC015	M	N	-0.05	-0.05
1	CC015	M	N	-0.33	-0.33
2	CC015	M	N	0.32	0.32
0	CC027	M	N	0.38	0.38

Trial#	Strain	Sex	Inf?	PawPlacementPositioning[PPP]_LF	PawPlacementPositioning[PPP]_LH
1	CC027	M	N	-0.16	-0.16
2	CC027	M	N	0.06	0.06
0	CC015	F	N	0.31	0.38
1	CC015	F	N	0.37	0.37
0	CC017	F	N	0.28	0.28
0	CC023	F	N	0.12	0.12
1	CC023	F	N	0.45	0.45
0	CC005	M	N	-0.35	-0.35
1	CC005	M	N	0.77	0.77
0	CC015	M	N	0.44	0.44
1	CC015	M	N	0.06	0.06
0	CC017	M	N	-0.12	-0.12
1	CC017	M	N	0.21	0.21
0	CC023	M	N	0.19	0.19
1	CC023	M	N	0.9	0.9
0	CC051	M	N	0.32	0.32
0	CC002	F	Y	0.31	0.31
0	CC002	M	Y	-0.03	-0.03
1	CC002	M	Y	0.04	0.04
1	CC002	F	Y	0.63	0.63
1	CC002	F	Y	0.13	0.13
1	CC002	M	Y	0.04	0.04
1	CC002	M	Y	0.16	0.16
1	CC002	F	Y	0.63	0.63
1	CC002	F	Y	0.43	0.43
2	CC002	M	Y	0.58	0.58
2	CC002	F	Y	0.31	0.31
2	CC002	M	Y	-0.19	-0.19
0	CC005	F	Y	0.18	0.18
0	CC005	M	Y	0.3	0.3
0	CC005	M	Y	0.6	0.6
1	CC005	F	Y	0.06	0.06
1	CC005	M	Y	1.31	1.31
1	CC005	F	Y	1.14	1.14
1	CC005	M	Y	0.73	0.73
2	CC005	F	Y	0.54	0.54
2	CC005	M	Y	2.06	2.06
2	CC005	M	Y	1.07	1.07
2	CC005	M	Y	2.06	2.06
2	CC005	F	Y	1.86	1.86
0	CC006	F	Y	0.55	0.55
0	CC006	M	Y	0.11	0.11
0	CC006	F	Y	0.07	0.07
1	CC006	F	Y	0.42	0.42
1	CC006	M	Y	0.23	0.23
1	CC006	F	Y	0.25	0.25
1	CC006	M	Y	0.27	0.27
2	CC006	F	Y	0.25	0.25

Trial#	Strain	Sex	Inf?	PawPlacementPositioning[PPP]_LF	PawPlacementPositioning[PPP]_LH
2	CC006	M	Y	-0.16	-0.16
0	CC011	F	Y	0.44	0.44
0	CC011	M	Y	0.11	0.11
0	CC011	F	Y	0.22	0.22
0	CC011	F	Y	-0.22	-0.22
1	CC011	F	Y	0.01	0.01
1	CC011	M	Y	0.09	0.09
1	CC011	F	Y	-0.1	-0.1
1	CC011	M	Y	0.24	0.24
2	CC011	F	Y	0.09	0.09
2	CC011	M	Y	0.32	0.32
2	CC011	F	Y	0.38	0.38
2	CC011	M	Y	0.19	0.19
0	CC012	M	Y	0.31	0.31
0	CC012	M	Y	0.35	0.35
0	CC012	M	Y	0.41	0.41
0	CC012	M	Y	0.12	0.12
0	CC012	M	Y	0.42	0.42
0	CC012	F	Y	0.02	0.02
0	CC012	F	Y	0.7	0.7
0	CC012	F	Y	0.17	0.17
0	CC012	F	Y	0.3	0.3
0	CC012	F	Y	-0.02	-0.02
2	CC012	M	Y	0.41	0.41
2	CC012	M	Y	-0.08	-0.08
2	CC012	M	Y	0.12	0.12
2	CC012	M	Y	0.61	0.61
2	CC012	M	Y	0.55	0.55
2	CC012	F	Y	0.74	0.74
2	CC012	F	Y	0.78	0.78
2	CC012	F	Y	0.96	0.96
2	CC012	F	Y	0.57	0.57
2	CC012	F	Y	0.84	0.84
1	CC012XCC032	F	Y	-0.55	-0.55
1	CC012xCC032	F	Y	0.73	0.73
1	CC012XCC032	M	Y	0.97	0.97
1	CC012xCC032	M	Y	0.13	0.13
1	CC012XCC032	M	Y	0.07	0.07
1	CC012xCC032	M	Y	0.55	0.55
2	CC012XCC032	F	Y	1.77	1.77
2	CC012XCC032	F	Y	0.72	0.72
2	CC012XCC032	M	Y	0.88	0.88
2	CC012XCC032	M	Y	0.48	0.48
2	CC012XCC032	M	Y	1.06	1.06
2	CC012XCC032	M	Y	-0.32	-0.32
1	CC013xCC041	F	Y	0.42	0.42
1	CC013xCC041	F	Y	0.42	0.42
1	CC013xCC041	F	Y	0.64	0.64

Trial#	Strain	Sex	Inf?	PawPlacementPositioning[PPP]_LF	PawPlacementPositioning[PPP]_LH
1	CC013xCC041	M	Y	-0.04	-0.04
1	CC013xCC041	M	Y	0.71	0.71
2	CC013XCC041	F	Y	0.79	0.79
2	CC013XCC041	F	Y	0.82	0.82
2	CC013XCC041	F	Y	0.52	0.52
2	CC013XCC041	M	Y	0.41	0.41
2	CC013XCC041	M	Y	0.19	0.19
0	CC015	F	Y	0.42	0.42
0	CC015	M	Y	0.3	0.3
0	CC015	M	Y	0.28	0.28
1	CC015	F	Y	1.29	1.29
1	CC015	M	Y	0.41	0.41
1	CC015	M	Y	0.42	0.42
1	CC015	M	Y	0.47	0.47
2	CC015	F	Y	0.41	0.41
0	CC017	F	Y	0.04	0.04
0	CC017	M	Y	0.16	0.16
0	CC017	F	Y	-0.02	-0.02
0	CC017	M	Y	0.28	0.28
1	CC017	M	Y	0.53	0.53
1	CC017	F	Y	0.37	0.37
0	CC023	M	Y	0.64	0.64
0	CC023	F	Y	0.58	0.58
0	CC023	F	Y	0.17	0.17
0	CC023	M	Y	0.75	0.75
1	CC023	M	Y	0.32	0.32
1	CC023	F	Y	0.39	0.39
1	CC023	M	Y	0.89	0.89
1	CC023	M	Y	0.42	0.42
2	CC023	M	Y	0.74	0.74
2	CC023	F	Y	0.18	0.18
2	CC023	M	Y	0.72	0.72
0	CC025	M	Y	0	0
0	CC025	F	Y	0.28	0.28
0	CC025	M	Y	0.09	0.09
1	CC025	M	Y	0.02	0.02
1	CC025	F	Y	0.28	0.28
1	CC025	F	Y	0.14	0.14
1	CC025	F	Y	0.3	0.3
2	CC025	F	Y	-0.31	-0.31
2	CC025	M	Y	1.09	1.09
0	CC027	F	Y	-0.61	-0.61
0	CC027	M	Y	0.62	0.62
1	CC027	M	Y	0.66	0.66
1	CC027	M	Y	0.66	0.66
1	CC027	F	Y	0.31	0.31
1	CC027	F	Y	0.56	0.56
1	CC027	M	Y	0.3	0.3

Trial#	Strain	Sex	Inf?	PawPlacementPositioning[PPP]_LF	PawPlacementPositioning[PPP]_LH
1	CC027	M	Y	0.58	0.58
2	CC027	F	Y	0.18	0.18
2	CC027	M	Y	0.81	0.81
2	CC027	F	Y	0.34	0.34
2	CC027	M	Y	0.2	0.2
1	CC032XCC013	M	Y	0.61	0.61
1	CC032XCC013	M	Y	0.55	0.55
1	CC032XCC013	M	Y	0.6	0.6
1	CC032XCC013	F	Y	0.68	0.68
1	CC032XCC013	F	Y	0.41	0.41
1	CC032XCC013	F	Y	0.71	0.71
1	CC032XCC013	F	Y	0.81	0.81
1	CC032XCC013	F	Y	0.84	0.84
2	CC032XCC013	M	Y	-0.18	-0.18
2	CC032XCC013	M	Y	0.48	0.48
2	CC032XCC013	M	Y	0.15	0.15
2	CC032XCC013	M	Y	0.53	0.53
2	CC032XCC013	F	Y	0.98	0.98
2	CC032XCC013	F	Y	0.99	0.99
2	CC032XCC013	F	Y	0.6	0.6
2	CC032XCC013	F	Y	0.84	0.84
0	CC037	F	Y	0.42	0.42
0	CC037	M	Y	0.17	0.17
0	CC037	M	Y	0.56	0.56
0	CC037	M	Y	0.54	0.54
1	CC037	F	Y	0.36	0.36
1	CC037	M	Y	0.4	0.4
1	CC037	M	Y	0.3	0.3
1	CC037	M	Y	0.75	0.75
2	CC037	M	Y	0.51	0.51
2	CC037	M	Y	0.54	0.54
1	CC041XCC012	M	Y	0.74	0.4
1	CC041XCC012	M	Y	0.4	0.43
1	CC041XCC012	M	Y	0.43	0.67
1	CC041XCC012	M	Y	0.67	0.26
1	CC041XCC012	M	Y	0.26	0.48
1	CC041XCC012	F	Y	0.8	0.8
1	CC041XCC012	F	Y	0.74	0.74
1	CC041XCC012	F	Y	0.48	0.69
1	CC041XCC012	F	Y	0.69	0.76
1	CC041XCC012	F	Y	0.76	0.74
1	CC041XCC012	F	Y	0.74	1.06
1	CC041XCC012	F	Y	1.06	0.49
1	CC041XCC012	F	Y	0.49	0.74
2	CC041XCC012	M	Y	0.4	0.4
2	CC041XCC012	M	Y	0.91	0.91
2	CC041XCC012	F	Y	1	1
2	CC041XCC012	M	Y	0.03	0.03

Trial#	Strain	Sex	Inf?	PawPlacementPositioning[PPP]_LF	PawPlacementPositioning[PPP]_LH
2	CC041XCC012	M	Y	0.42	0.42
2	CC041XCC012	M	Y	0.23	0.23
2	CC041XCC012	F	Y	0.7	0.7
2	CC041XCC012	F	Y	0.93	0.93
2	CC041XCC012	F	Y	0.29	0.29
2	CC041XCC012	F	Y	0.56	0.56
2	CC041XCC012	F	Y	0.53	0.53
2	CC041XCC012	F	Y	0.52	0.52
2	CC041XCC012	F	Y	0.37	0.37
0	CC051	F	Y	0.87	0.87
0	CC051	F	Y	0.55	0.55
0	CC051	F	Y	0.15	0.15
0	CC051	M	Y	0.19	0.19
1	CC051	F	Y	0.26	0.26
1	CC051	F	Y	0.67	0.67
1	CC051	F	Y	0.4	0.4
0	CC057	F	Y	0.27	0.27
0	CC057	F	Y	0.3	0.3
0	CC057	F	Y	0.83	0.83
0	CC057	M	Y	0.36	0.36
0	CC057	M	Y	0.77	0.77
0	CC057	M	Y	0.4	0.4
1	CC057	F	Y	0.2	0.2
1	CC057	F	Y	0.47	0.47
1	CC057	F	Y	0.23	0.23
1	CC057	F	Y	0.31	0.31
1	CC057	M	Y	0.26	0.26
1	CC057	M	Y	0.28	0.28
2	CC057	F	Y	0.31	0.31
0	CC078	F	Y	0.26	0.26
0	CC078	F	Y	0.3	0.3
0	CC078	F	Y	0.31	0.31
0	CC078	M	Y	0.4	0.4
1	CC078	F	Y	0.93	0.93
1	CC078	F	Y	0.79	0.79
1	CC078	M	Y	0.12	0.12
2	CC078	F	Y	0.52	0.52
2	CC078	M	Y	-0.12	-0.12
2	CC005	M	N	0.86	0.86
2	CC005	M	Y	0.76	0.76
2	CC015	F	N	0.26	0.26
2	CC015	M	N	0.16	0.16
2	CC015	M	Y	0.4	0.4
2	CC015	M	Y	0.11	0.11
2	CC017	F	N	0.89	0.89
2	CC017	M	N	0.49	0.49
2	CC017	F	Y	0.55	0.55
2	CC023	F	N	0.61	0.61

Trial#	Strain	Sex	Inf?	PawPlacementPositioning[PPP]_LF	PawPlacementPositioning[PPP]_LH

Table S3. Raw DigiGait measurement data. Far left column indicates time point at which data was measured: T0 = pre-infection, T1 = 21dpi, and T3 = 89dpi. DigiGait parameters listed across the top indicate which limb is associated with the data, where appropriate: FL for left fore limb, FR for right fore limb, HL for left hind limb, and HR for right hind limb.

Table S3

Trial#	Strain	Sex	Inf?	PawPlacementPositioning[PPP]_RF	PawPlacementPositioning[PPP]_RH	Propel_LF
1	CC002	F	N	0.33	0.33	0.105
1	CC025	F	N	0.29	0.29	0.07
1	CC012XCC032	F	N	1.1	1.1	0.108
2	CC012XCC032	F	N	0.76	0.76	0.132
1	CC012XCC032	M	N	0.05	0.05	0.112
2	CC012XCC032	M	N	0.38	0.38	0.088
1	CC013xCC041	F	N	0.62	0.62	0.101
2	CC013XCC041	F	N	0.43	0.43	0.114
1	CC013xCC041	M	N	1.38	1.38	0.024
2	CC013XCC041	M	N	0.59	0.59	0.099
1	CC032XCC013	F	N	1.21	1.21	0.042
2	CC032XCC013	F	N	0.76	0.76	0.087
1	CC041XCC012	F	N	0.36	0.36	0.071
1	CC041XCC012	F	N	0.31	0.31	0.043
2	CC041XCC012	F	N	0.29	0.29	0.08
1	CC032XCC013	M	N	-0.5	-0.5	0.04
2	CC032XCC013	M	N	0.15	0.15	0.045
1	CC041XCC012	M	N	0.49	0.49	0.059
2	CC041XCC012	M	N	0.26	0.26	0.07
0	CC012	F	N	0.43	0.43	0.093
2	CC012	F	N	0.3	0.3	0.121
0	CC012	M	N	0.37	0.37	0.064
2	CC012	M	N	1.68	1.68	0.068
0	CC057	F	N	0.41	0.41	0.111
1	CC057	F	N	1.36	1.36	0.139
0	CC057	M	N	0.76	0.76	0.09
1	CC057	M	N	0.88	0.88	0.167
0	CC078	F	N	0.28	0.28	0.132
1	CC078	F	N	0.16	0.16	0.089
2	CC078	F	N	0.42	0.42	0.057
0	CC078	M	N	0.5	0.5	0.117
2	CC078	M	N	0.12	0.12	0.102
0	CC002	F	N	0.36	0.36	0.087
1	CC002	F	N	0.33	0.33	0.105
0	CC002	M	N	0.45	0.45	0.046
1	CC002	M	N	0.12	0.12	0.049
2	CC002	M	N	0.07	0.07	0.063
0	CC006	F	N	-0.02	-0.02	0.031
1	CC006	F	N	0.64	0.64	0.046
2	CC006	F	N	-0.11	-0.11	0.041
0	CC006	M	N	0.06	0.06	0.065
0	CC023	F	N	0.03	0.03	0.095
1	CC023	F	N	0.28	0.28	0.077
2	CC023	F	N	0.54	0.54	0.112
0	CC023	M	N	0.61	0.61	0.141
1	CC023	M	N	0.52	0.52	0.114
0	CC027	F	N	0.1	0.1	0.048

Trial#	Strain	Sex	Inf?	PawPlacementPositioning[PPP]_RF	PawPlacementPositioning[PPP]_RH	Propel_LF
1	CC027	F	N	0.1	0.1	0.09
2	CC027	F	N	0.31	0.31	0.144
0	CC027	M	N	-0.05	-0.05	0.069
1	CC027	M	N	1.03	1.03	0.054
1	CC027	M	N	0.85	0.85	0.103
2	CC027	M	N	0.01	0.01	0.095
0	CC005	F	N	0.23	0.23	0.071
1	CC005	F	N	0.68	0.68	0.062
2	CC005	F	N	0	0	0.029
0	CC011	F	N	0.27	0.27	0.044
1	CC011	F	N	0.31	0.31	0.083
2	CC011	F	N	0.08	0.08	0.131
1	CC017	F	N	0.16	0.16	0.068
0	CC005	M	N	0.38	0.38	0.083
1	CC005	M	N	1.31	1.31	0.052
2	CC005	M	N	0.85	0.85	0.059
0	CC011	M	N	-0.03	-0.03	0.092
1	CC011	M	N	0.06	0.06	0.087
2	CC011	M	N	0.13	0.13	0.071
0	CC017	M	N	0.43	0.43	0.073
1	CC017	M	N	0.26	0.26	0.058
1	CC006	F	N	-0.36	-0.36	0.018
0	CC037	F	N	0.43	0.43	0.089
0	CC051	F	N	0.61	0.61	0.084
1	CC051	F	N	1.05	1.05	0.066
1	CC006	M	N	0.76	0.76	0.082
0	CC037	M	N	0.36	0.36	0.075
1	CC037	M	N	0.87	0.87	0.079
0	CC005	F	N	0.53	0.53	0.063
1	CC005	F	N	-0.05	-0.05	0.138
2	CC005	F	N	0.23	0.23	0.105
0	CC011	F	N	0.23	0.23	0.109
1	CC011	F	N	0.62	0.62	0.125
2	CC011	F	N	0.31	0.31	0.117
0	CC011	M	N	0.39	0.39	0.081
1	CC011	M	N	0.64	0.64	0.105
2	CC011	M	N	0.26	0.26	0.076
1	CC037	M	N	0.49	0.49	0.096
2	CC037	M	N	1.36	1.36	0.103
1	CC051	M	N	0.16	0.16	0.131
2	CC051	M	N	0.49	0.49	0.085
0	CC027	F	N	1.13	1.13	0.127
1	CC027	F	N	0.38	0.38	0.105
2	CC027	F	N	0.41	0.41	0.069
0	CC015	M	N	0.03	0.03	0.131
1	CC015	M	N	0.22	0.22	0.065
2	CC015	M	N	-0.03	-0.03	0.139
0	CC027	M	N	0.46	0.46	0.095

Trial#	Strain	Sex	Inf?	PawPlacementPositioning[PPP]_RF	PawPlacementPositioning[PPP]_RH	Propel_LF
1	CC027	M	N	0.41	0.41	0.091
2	CC027	M	N	0.15	0.15	0.077
0	CC015	F	N	0.38	0.151	0.114
1	CC015	F	N	0.42	0.42	0.041
0	CC017	F	N	0.63	0.63	0.069
0	CC023	F	N	0.8	0.8	0.1
1	CC023	F	N	0.5	0.5	0.108
0	CC005	M	N	1.3	1.3	0.1
1	CC005	M	N	0.36	0.36	0.061
0	CC015	M	N	0.11	0.11	0.108
1	CC015	M	N	-0.01	-0.01	0.094
0	CC017	M	N	0.37	0.37	0.123
1	CC017	M	N	0.09	0.09	0.035
0	CC023	M	N	0.58	0.58	0.099
1	CC023	M	N	0.3	0.3	0.068
0	CC051	M	N	0.48	0.48	0.067
0	CC002	F	Y	0.13	0.13	0.04
0	CC002	M	Y	0.1	0.1	0.066
1	CC002	M	Y	-0.23	-0.23	0.091
1	CC002	F	Y	0.13	0.13	0.047
1	CC002	F	Y	0.48	0.48	0.045
1	CC002	M	Y	-0.23	-0.23	0.091
1	CC002	M	Y	0.12	0.12	0.067
1	CC002	F	Y	0.13	0.13	0.047
1	CC002	F	Y	-0.51	-0.51	0.091
2	CC002	M	Y	0.21	0.21	0.082
2	CC002	F	Y	-0.05	-0.05	0.121
2	CC002	M	Y	-0.26	-0.26	0.099
0	CC005	F	Y	0.08	0.08	0.077
0	CC005	M	Y	0.99	0.99	0.085
0	CC005	M	Y	0.39	0.39	0.048
1	CC005	F	Y	0.37	0.37	0.043
1	CC005	M	Y	-0.39	-0.39	0.172
1	CC005	F	Y	0.73	0.73	0.101
1	CC005	M	Y	0.35	0.35	0.048
2	CC005	F	Y	0.27	0.27	0.025
2	CC005	M	Y	0.72	0.72	0.067
2	CC005	M	Y	-0.27	-0.27	0.062
2	CC005	M	Y	0.72	0.72	0.067
2	CC005	F	Y	0.68	0.68	0.058
0	CC006	F	Y	0.1	0.1	0.058
0	CC006	M	Y	-0.21	-0.21	0.082
0	CC006	F	Y	0.36	0.36	0.074
1	CC006	F	Y	-0.08	-0.08	0.041
1	CC006	M	Y	0.08	0.08	0.045
1	CC006	F	Y	0.11	0.11	0.097
1	CC006	M	Y	0.68	0.68	0.126
2	CC006	F	Y	0.16	0.16	0.06

Trial#	Strain	Sex	Inf?	PawPlacementPositioning[PPP]_RF	PawPlacementPositioning[PPP]_RH	Propel_LF
2	CC006	M	Y	0.08	0.08	0.035
0	CC011	F	Y	-0.31	-0.31	0.051
0	CC011	M	Y	0.2	0.2	0.099
0	CC011	F	Y	0.12	0.12	0.077
0	CC011	F	Y	0.11	0.11	0.069
1	CC011	F	Y	0.19	0.19	0.091
1	CC011	M	Y	0	0	0.083
1	CC011	F	Y	0.35	0.35	0.09
1	CC011	M	Y	0.1	0.1	0.061
2	CC011	F	Y	0.01	0.01	0.145
2	CC011	M	Y	0.44	0.44	0.103
2	CC011	F	Y	0.1	0.1	0.124
2	CC011	M	Y	0.29	0.29	0.072
0	CC012	M	Y	0.19	0.19	0.147
0	CC012	M	Y	-0.09	-0.09	0.134
0	CC012	M	Y	0.44	0.44	0.113
0	CC012	M	Y	0.12	0.12	0.125
0	CC012	M	Y	0.41	0.41	0.151
0	CC012	F	Y	0.4	0.4	0.071
0	CC012	F	Y	0.19	0.19	0.137
0	CC012	F	Y	0.51	0.51	0.067
0	CC012	F	Y	0.76	0.76	0.048
0	CC012	F	Y	0.06	0.06	0.051
2	CC012	M	Y	0.31	0.31	0.087
2	CC012	M	Y	0.66	0.66	0.135
2	CC012	M	Y	0.71	0.71	0.167
2	CC012	M	Y	0.37	0.37	0.206
2	CC012	M	Y	0.22	0.22	0.097
2	CC012	F	Y	0.43	0.43	0.125
2	CC012	F	Y	0.52	0.52	0.045
2	CC012	F	Y	0.67	0.67	0.093
2	CC012	F	Y	0.73	0.73	0.144
2	CC012	F	Y	0.93	0.93	0.108
1	CC012XCC032	F	Y	-0.59	-0.59	0.078
1	CC012xCC032	F	Y	0.62	0.62	0.033
1	CC012XCC032	M	Y	0.74	0.74	0.083
1	CC012xCC032	M	Y	0.5	0.5	0.071
1	CC012XCC032	M	Y	-0.48	-0.48	0.031
1	CC012xCC032	M	Y	0.44	0.44	0.076
2	CC012XCC032	F	Y	0.15	0.15	0.064
2	CC012XCC032	F	Y	0.55	0.55	0.122
2	CC012XCC032	M	Y	0.56	0.56	0.083
2	CC012XCC032	M	Y	0.99	0.99	0.052
2	CC012XCC032	M	Y	0.35	0.35	0.076
2	CC012XCC032	M	Y	0.15	0.15	0.071
1	CC013xCC041	F	Y	0.49	0.49	0.058
1	CC013xCC041	F	Y	0.27	0.27	0.027
1	CC013xCC041	F	Y	0.82	0.82	0.053

Trial#	Strain	Sex	Inf?	PawPlacementPositioning[PPP]_RF	PawPlacementPositioning[PPP]_RH	Propel_LF
1	CC013xCC041	M	Y	0.7	0.7	0.058
1	CC013xCC041	M	Y	0.34	0.34	0.114
2	CC013XCC041	F	Y	0.04	0.04	0.113
2	CC013XCC041	F	Y	0.22	0.22	0.081
2	CC013XCC041	F	Y	0.37	0.37	0.052
2	CC013XCC041	M	Y	0.44	0.44	0.046
2	CC013XCC041	M	Y	-0.21	-0.21	0.043
0	CC015	F	Y	0.39	0.39	0.071
0	CC015	M	Y	0.29	0.29	0.093
0	CC015	M	Y	0.26	0.26	0.088
1	CC015	F	Y	0.67	0.67	0.111
1	CC015	M	Y	0.43	0.43	0.047
1	CC015	M	Y	0.45	0.45	0.055
1	CC015	M	Y	0.39	0.39	0.052
2	CC015	F	Y	0.08	0.08	0.083
0	CC017	F	Y	0.13	0.13	0.062
0	CC017	M	Y	0.2	0.2	0.092
0	CC017	F	Y	0.09	0.09	0.062
0	CC017	M	Y	-0.39	-0.39	0.047
1	CC017	M	Y	0.43	0.43	0.047
1	CC017	F	Y	0.08	0.08	0.05
0	CC023	M	Y	0.49	0.49	0.136
0	CC023	F	Y	-0.3	-0.3	0.097
0	CC023	F	Y	0.08	0.08	0.056
0	CC023	M	Y	2.21	2.21	0.017
1	CC023	M	Y	0.47	0.47	0.045
1	CC023	F	Y	0.66	0.66	0.046
1	CC023	M	Y	1.14	1.14	0.063
1	CC023	M	Y	1.07	1.07	0.094
2	CC023	M	Y	0.15	0.15	0.159
2	CC023	F	Y	0.57	0.57	0.077
2	CC023	M	Y	0.59	0.59	0.055
0	CC025	M	Y	0.53	0.53	0.098
0	CC025	F	Y	0.84	0.84	0.081
0	CC025	M	Y	0.44	0.44	0.102
1	CC025	M	Y	0.51	0.51	0.102
1	CC025	F	Y	0.31	0.31	0.059
1	CC025	F	Y	0.39	0.39	0.079
1	CC025	F	Y	0.2	0.2	0.119
2	CC025	F	Y	-0.72	-0.72	0.089
2	CC025	M	Y	0.15	0.15	0.148
0	CC027	F	Y	-0.9	-0.9	0.072
0	CC027	M	Y	1.31	1.31	0.071
1	CC027	M	Y	-0.72	-0.72	0.116
1	CC027	M	Y	-0.72	-0.72	0.116
1	CC027	F	Y	0.17	0.17	0.041
1	CC027	F	Y	-0.12	-0.12	0.074
1	CC027	M	Y	-0.57	-0.57	0.107

Trial#	Strain	Sex	Inf?	PawPlacementPositioning[PPP]_RF	PawPlacementPositioning[PPP]_RH	Propel_LF
1	CC027	M	Y	0.11	0.11	0.103
2	CC027	F	Y	0.57	0.57	0.077
2	CC027	M	Y	0.03	0.03	0.099
2	CC027	F	Y	-0.45	-0.45	0.071
2	CC027	M	Y	0.3	0.3	0.085
1	CC032XCC013	M	Y	0.31	0.31	0.047
1	CC032XCC013	M	Y	0.29	0.29	0.097
1	CC032XCC013	M	Y	0.27	0.27	0.029
1	CC032XCC013	F	Y	1.15	1.15	0.049
1	CC032XCC013	F	Y	0.64	0.64	0.057
1	CC032XCC013	F	Y	0.71	0.71	0.057
1	CC032XCC013	F	Y	0.46	0.46	0.114
1	CC032XCC013	F	Y	0.36	0.36	0.104
2	CC032XCC013	M	Y	1.43	1.43	0.117
2	CC032XCC013	M	Y	0.36	0.36	0.054
2	CC032XCC013	M	Y	1.2	1.2	0.045
2	CC032XCC013	M	Y	0.34	0.34	0.088
2	CC032XCC013	F	Y	-0.03	-0.03	0.111
2	CC032XCC013	F	Y	0.19	0.19	0.056
2	CC032XCC013	F	Y	0.56	0.56	0.105
2	CC032XCC013	F	Y	0.04	0.04	0.114
0	CC037	F	Y	0.74	0.74	0.069
0	CC037	M	Y	0.55	0.55	0.048
0	CC037	M	Y	0.46	0.46	0.053
0	CC037	M	Y	0.16	0.16	0.067
1	CC037	F	Y	0.22	0.22	0.085
1	CC037	M	Y	0.58	0.58	0.107
1	CC037	M	Y	-0.21	-0.21	0.074
1	CC037	M	Y	0.17	0.17	0.139
2	CC037	M	Y	0.34	0.34	0.054
2	CC037	M	Y	0.02	0.02	0.179
1	CC041XCC012	M	Y	0.18	0.18	0.08
1	CC041XCC012	M	Y	0.61	0.61	0.049
1	CC041XCC012	M	Y	0.26	0.26	0.084
1	CC041XCC012	M	Y	0.26	0.26	0.046
1	CC041XCC012	M	Y	0.38	0.38	0.101
1	CC041XCC012	F	Y	0.7	0.7	0.077
1	CC041XCC012	F	Y	0.35	0.35	0.145
1	CC041XCC012	F	Y	-0.06	-0.06	0.122
1	CC041XCC012	F	Y	0.19	0.19	0.058
1	CC041XCC012	F	Y	0.76	0.76	0.111
1	CC041XCC012	F	Y	0.02	0.02	0.105
1	CC041XCC012	F	Y	0.08	0.08	0.088
1	CC041XCC012	F	Y	0.21	0.21	0.134
2	CC041XCC012	M	Y	0.25	0.25	0.113
2	CC041XCC012	M	Y	0.39	0.39	0.122
2	CC041XCC012	F	Y	0.18	0.18	0.134
2	CC041XCC012	M	Y	0.86	0.86	0.064

Trial#	Strain	Sex	Inf?	PawPlacementPositioning[PPP]_RF	PawPlacementPositioning[PPP]_RH	Propel_LF
2	CC041XCC012	M	Y	0.29	0.29	0.128
2	CC041XCC012	M	Y	0.72	0.72	0.108
2	CC041XCC012	F	Y	0.39	0.39	0.13
2	CC041XCC012	F	Y	0.78	0.78	0.098
2	CC041XCC012	F	Y	0.58	0.58	0.135
2	CC041XCC012	F	Y	0.16	0.16	0.131
2	CC041XCC012	F	Y	0.79	0.79	0.13
2	CC041XCC012	F	Y	0.32	0.32	0.12
2	CC041XCC012	F	Y	0.3	0.3	0.126
0	CC051	F	Y	0.52	0.52	0.091
0	CC051	F	Y	0.65	0.65	0.092
0	CC051	F	Y	0.29	0.29	0.098
0	CC051	M	Y	0.23	0.23	0.08
1	CC051	F	Y	0.24	0.24	0.097
1	CC051	F	Y	0.23	0.23	0.12
1	CC051	F	Y	0.35	0.35	0.094
0	CC057	F	Y	0.5	0.5	0.098
0	CC057	F	Y	0.58	0.58	0.069
0	CC057	F	Y	0.47	0.47	0.063
0	CC057	M	Y	0.59	0.59	0.047
0	CC057	M	Y	0.75	0.75	0.071
0	CC057	M	Y	0.42	0.42	0.045
1	CC057	F	Y	1.04	1.04	0.102
1	CC057	F	Y	0.06	0.06	0.065
1	CC057	F	Y	0.03	0.03	0.059
1	CC057	F	Y	0.71	0.71	0.052
1	CC057	M	Y	1.02	1.02	0.079
1	CC057	M	Y	0.37	0.37	0.099
2	CC057	F	Y	0.71	0.71	0.052
0	CC078	F	Y	0.2	0.2	0.093
0	CC078	F	Y	0.08	0.08	0.117
0	CC078	F	Y	0.08	0.08	0.103
0	CC078	M	Y	0.27	0.27	0.102
1	CC078	F	Y	0.17	0.17	0.109
1	CC078	F	Y	-0.4	-0.4	0.093
1	CC078	M	Y	0.24	0.24	0.072
2	CC078	F	Y	0.15	0.15	0.13
2	CC078	M	Y	0.22	0.22	0.052
2	CC005	M	N	0.11	0.11	0.035
2	CC005	M	Y	-0.01	-0.01	0.049
2	CC015	F	N	0.41	0.41	0.04
2	CC015	M	N	0.42	0.42	0.08
2	CC015	M	Y	0.48	0.48	0.081
2	CC015	M	Y	-0.31	-0.31	0.066
2	CC017	F	N	0.21	0.21	0.08
2	CC017	M	N	0.11	0.11	0.044
2	CC017	F	Y	0.39	0.39	0.048
2	CC023	F	N	0.43	0.43	0.058

Trial#	Strain	Sex	Inf?	PawPlacementPositioning[PPP]_RF	PawPlacementPositioning[PPP]_RH	Propel_LF

Table S3. Raw DigiGait measurement data. Far left column indicates time point at which data was measured: T0 = pre-infection, T1 = 21dpi, and T3 = 89dpi. DigiGait parameters listed across the top indicate which limb is associated with the data, where appropriate: FL for left fore limb, FR for right fore limb, HL for left hind limb, and HR for right hind limb.

Table S3

Trial#	Strain	Sex	Inf?	Propel_LH	Propel_RF	Propel_RH	SLVar_LF	SLVar_LH	SLVar_RF	SLVar_RH	Stance_LF
1	CC002	F	N	0.065	0.105	0.135	1.88	1.3	1.4	1.43	0.152
1	CC025	F	N	0.101	0.074	0.1	0.8	1.31	0.9	1.23	0.144
1	CC012XCC032	F	N	0.206	0.159	0.17	1.44	0.71	2.16	1.48	0.191
2	CC012XCC032	F	N	0.149	0.15	0.179	1.87	0.64	0.2	0.5	0.21
1	CC012XCC032	M	N	0.251	0.125	0.255	1.31	0.25	1.39	0.34	0.187
2	CC012XCC032	M	N	0.215	0.071	0.228	0.86	0.74	0.54	0.89	0.228
1	CC013xCC041	F	N	0.062	0.062	0.101	1.44	1.55	1.54	1.48	0.175
2	CC013XCC041	F	N	0.206	0.066	0.179	0.58	0.4	1.11	0.79	0.254
1	CC013xCC041	M	N	0.067	0.045	0.023	0.63	1.16	1.12	0.44	0.045
2	CC013XCC041	M	N	0.163	0.095	0.156	1.17	0.73	1.47	0.94	0.178
1	CC032XCC013	F	N	0.103	0.029	0.051	0.68	1.23	0.67	1.11	0.092
2	CC032XCC013	F	N	0.136	0.09	0.184	1.69	1.54	1.53	1.31	0.174
1	CC041XCC012	F	N	0.086	0.101	0.086	1.71	1.64	2.52	1.62	0.145
1	CC041XCC012	F	N	0.058	0.053	0.078	1.04	1.28	1.4	2.02	0.088
2	CC041XCC012	F	N	0.147	0.05	0.114	1.69	0.68	1.65	1.7	0.13
1	CC032XCC013	M	N	0.082	0.06	0.049	1.36	1.16	1.2	1.21	0.114
2	CC032XCC013	M	N	0.141	0.025	0.059	0.81	1.62	0.79	1.17	0.067
1	CC041XCC012	M	N	0.143	0.045	0.121	1.49	1.23	1.46	1.51	0.17
2	CC041XCC012	M	N	0.221	0.083	0.2	1.48	0.96	2.16	0.5	0.225
0	CC012	F	N	0.139	0.087	0.124	1.33	1.39	1.55	1.35	0.129
2	CC012	F	N	0.229	0.076	0.21	1.9	0.71	1.78	2.38	0.293
0	CC012	M	N	0.114	0.06	0.141	1.65	1.43	2	1.59	0.119
2	CC012	M	N	0.175	0.058	0.2	2.03	1.14	1.45	1.35	0.129
0	CC057	F	N	0.135	0.087	0.086	1.4	0.43	1.35	1.76	0.195
1	CC057	F	N	0.151	0.144	0.144	1.48	0.43	1.41	0.9	0.215
0	CC057	M	N	0.113	0.124	0.136	1.74	1.05	0.73	1.53	0.155
1	CC057	M	N	0.186	0.203	0.179	0.73	0.42	0.91	0.41	0.242
0	CC078	F	N	0.18	0.131	0.171	0.41	0.23	0.43	0.85	0.18
1	CC078	F	N	0.18	0.077	0.174	1.23	0.75	1.58	0.46	0.189
2	CC078	F	N	0.163	0.041	0.066	1.54	1.59	1.18	1.32	0.105
0	CC078	M	N	0.136	0.092	0.128	0.73	0.54	0.68	0.69	0.143
2	CC078	M	N	0.125	0.073	0.109	1.42	0.95	1.7	1.38	0.148
0	CC002	F	N	0.064	0.051	0.035	1.75	1.13	1.46	0.93	0.153
1	CC002	F	N	0.065	0.105	0.135	1.88	1.3	1.4	1.43	0.152
0	CC002	M	N	0.097	0.064	0.046	1.11	1.35	2.17	1.21	0.086
1	CC002	M	N	0.065	0.115	0.138	1.18	1.77	2.24	1.51	0.106
2	CC002	M	N	0.264	0.068	0.163	1.74	0.67	2	2.12	0.233
0	CC006	F	N	0.198	0.099	0.166	0.7	0.62	0.77	0.61	0.176
1	CC006	F	N	0.138	0.059	0.184	1.54	2.08	1.45	1.01	0.128
2	CC006	F	N	0.082	0.082	0.07	1.07	1.11	1.56	1.47	0.096
0	CC006	M	N	0.073	0.052	0.033	1.46	1.48	1.62	0.76	0.139
0	CC023	F	N	0.071	0.105	0.131	1.66	1.84	1.76	0.98	0.173
1	CC023	F	N	0.138	0.044	0.108	1.93	2	1.69	1.59	0.132
2	CC023	F	N	0.158	0.092	0.141	2.51	2.58	2.15	1.88	0.206
0	CC023	M	N	0.189	0.143	0.15	0.84	0.58	0.58	1.54	0.187
1	CC023	M	N	0.161	0.127	0.14	1.16	0.7	0.75	0.75	0.166
0	CC027	F	N	0.029	0.036	0.039	1.62	1.67	0.85	1.63	0.08

Trial#	Strain	Sex	Inf?	Propel_LH	Propel_RF	Propel_RH	SLVar_LF	SLVar_LH	SLVar_RF	SLVar_RH	Stance_LF
1	CC027	F	N	0.049	0.101	0.082	2.46	1.9	2.3	2.05	0.17
2	CC027	F	N	0.169	0.127	0.152	1.45	2.3	1.94	1.94	0.194
0	CC027	M	N	0.029	0.095	0.027	1.14	0.78	1.38	2.5	0.098
1	CC027	M	N	0.086	0.047	0.055	0.56	1.41	1.54	1.1	0.082
1	CC027	M	N	0.066	0.034	0.065	2	1.8	3.29	1.45	0.226
2	CC027	M	N	0.128	0.063	0.093	0.93	0.41	0.53	0.72	0.179
0	CC005	F	N	0.151	0.084	0.164	1.4	0.43	0.77	0.37	0.124
1	CC005	F	N	0.043	0.061	0.099	1.43	1.15	1.98	0.95	0.129
2	CC005	F	N	0.026	0.043	0.032	0.92	0.74	1.57	0.86	0.079
0	CC011	F	N	0.061	0.06	0.125	0.84	1.25	1.02	0.87	0.083
1	CC011	F	N	0.236	0.066	0.18	1.52	1.11	1.82	1.39	0.123
2	CC011	F	N	0.214	0.122	0.206	2.04	0.7	1.65	0.71	0.203
1	CC017	F	N	0.14	0.094	0.131	1.67	1.57	1.87	1.48	0.129
0	CC005	M	N	0.171	0.092	0.169	0.47	0.32	0.92	0.29	0.188
1	CC005	M	N	0.075	0.044	0.175	1.58	1.6	2.06	1.68	0.122
2	CC005	M	N	0.107	0.054	0.145	1.62	1.88	1.7	2.21	0.116
0	CC011	M	N	0.14	0.083	0.133	0.28	0.64	0.56	1	0.143
1	CC011	M	N	0.134	0.122	0.158	0.73	0.55	1.05	0.67	0.162
2	CC011	M	N	0.154	0.053	0.13	2.08	2.09	1.9	2.04	0.146
0	CC017	M	N	0.031	0.06	0.031	2.05	1.32	1.49	1.26	0.109
1	CC017	M	N	0.163	0.153	0.12	0.72	1	2.18	2.02	0.101
1	CC006	F	N	0.017	0.017	0.023	0.87	0.66	0.7	0.88	0.062
0	CC037	F	N	0.152	0.092	0.086	2.75	2.16	1.87	1.18	0.157
0	CC051	F	N	0.179	0.07	0.143	2.56	1.62	1.89	1.93	0.123
1	CC051	F	N	0.095	0.113	0.107	1.03	1.18	1.59	1.28	0.098
1	CC006	M	N	0.15	0.069	0.142	2.05	1.35	1.43	1.14	0.16
0	CC037	M	N	0.117	0.096	0.158	1.82	1.11	1.44	1.3	0.137
1	CC037	M	N	0.144	0.066	0.129	1.41	1.25	1.11	1.45	0.159
0	CC005	F	N	0.153	0.078	0.153	1.54	1.19	1.37	1.15	0.133
1	CC005	F	N	0.204	0.128	0.229	0.63	2.14	2.01	3.12	0.236
2	CC005	F	N	0.16	0.045	0.132	0.58	2.3	0.87	0.44	0.22
0	CC011	F	N	0.159	0.09	0.136	0.5	0.77	0.7	0.63	0.167
1	CC011	F	N	0.157	0.117	0.142	0.94	0.6	0.76	1.15	0.183
2	CC011	F	N	0.108	0.104	0.122	1.35	0.84	1.02	0.64	0.225
0	CC011	M	N	0.135	0.112	0.122	1.41	0.86	1.05	0.48	0.169
1	CC011	M	N	0.134	0.141	0.123	1.36	0.53	0.53	0.17	0.195
2	CC011	M	N	0.132	0.132	0.122	1.03	0.83	1.47	0.27	0.182
1	CC037	M	N	0.154	0.064	0.142	1.64	1.02	1.31	0.83	0.208
2	CC037	M	N	0.155	0.045	0.114	1.35	1.24	1.37	1.13	0.144
1	CC051	M	N	0.14	0.119	0.17	0.77	0.43	1.11	0.7	0.196
2	CC051	M	N	0.104	0.101	0.176	1.64	0.72	2.28	1.2	0.164
0	CC027	F	N	0.23	0.099	0.164	0.71	0.49	1.46	0.52	0.197
1	CC027	F	N	0.094	0.087	0.087	0.85	1.47	1.19	1.45	0.189
2	CC027	F	N	0.132	0.078	0.102	2.31	1.83	2.07	2.21	0.164
0	CC015	M	N	0.119	0.134	0.117	1.19	0.65	0.54	0.67	0.191
1	CC015	M	N	0.097	0.07	0.047	1.59	1.1	1.77	1.15	0.128
2	CC015	M	N	0.15	0.13	0.168	0.69	0.91	1.43	0.74	0.232
0	CC027	M	N	0.15	0.165	0.127	2.47	1.34	0.95	0.38	0.18

Trial#	Strain	Sex	Inf?	Propel_LH	Propel_RF	Propel_RH	SLVar_LF	SLVar_LH	SLVar_RF	SLVar_RH	Stance_LF
1	CC027	M	N	0.173	0.123	0.124	1.81	0.44	0.79	1.66	0.168
2	CC027	M	N	0.089	0.107	0.128	1.6	1.69	0.47	1.2	0.138
0	CC015	F	N	0.134	0.122	1.23	0.74	0.9	0.82	0.215	0.202
1	CC015	F	N	0.05	0.042	0.047	1.3	1.32	1.13	1.04	0.092
0	CC017	F	N	0.124	0.097	0.106	1.62	2.11	1.16	0.69	0.147
0	CC023	F	N	0.07	0.065	0.077	1	0.56	1.42	0.65	0.164
1	CC023	F	N	0.137	0.112	0.134	1.04	0.78	1.5	0.55	0.201
0	CC005	M	N	0.134	0.043	0.063	0.88	1.57	0.68	1.53	0.18
1	CC005	M	N	0.253	0.069	0.117	1.33	1.73	0.92	1.17	0.241
0	CC015	M	N	0.171	0.098	0.129	1.04	1.17	1.26	1.04	0.197
1	CC015	M	N	0.133	0.036	0.048	1.66	0.36	1.44	0.84	0.157
0	CC017	M	N	0.099	0.099	0.116	1.38	1.3	1.5	0.93	0.194
1	CC017	M	N	0.057	0.031	0.055	0.9	1.15	0.96	1.2	0.089
0	CC023	M	N	0.176	0.141	0.143	0.64	0.56	0.41	0.39	0.192
1	CC023	M	N	0.172	0.122	0.197	1.87	1.32	1.41	1.37	0.163
0	CC051	M	N	0.217	0.101	0.134	0.67	1.07	0.7	0.73	0.202
0	CC002	F	Y	0.061	0.031	0.049	1.29	1.38	1.16	1.4	0.084
0	CC002	M	Y	0.178	0.069	0.105	1.49	2.41	1.28	2.17	0.134
1	CC002	M	Y	0.029	0.097	0.018	1.29	0.91	1.58	2.67	0.148
1	CC002	F	Y	0.023	0.037	0.027	0.4	0.39	0.65	0.55	0.074
1	CC002	F	Y	0.084	0.066	0.08	0.99	1.51	1.37	1.44	0.082
1	CC002	M	Y	0.029	0.097	0.018	1.29	0.91	1.58	2.67	0.148
1	CC002	M	Y	0.064	0.057	0.051	1.89	1.51	1.5	0.94	0.124
1	CC002	F	Y	0.023	0.037	0.027	0.4	0.39	0.65	0.55	0.074
1	CC002	F	Y	0.028	0.058	0.033	0.89	0.72	0.8	0.63	0.135
2	CC002	M	Y	0.131	0.053	0.069	1.54	0.72	0.9	0.62	0.128
2	CC002	F	Y	0.163	0.129	0.172	1.17	1.24	1.03	0.72	0.205
2	CC002	M	Y	0.196	0.051	0.062	1.56	1.43	1.4	1.89	0.14
0	CC005	F	Y	0.145	0.071	0.164	1.04	0.37	1.05	0.77	0.133
0	CC005	M	Y	0.18	0.099	0.171	1.02	0.51	1.13	0.49	0.135
0	CC005	M	Y	0.159	0.049	0.14	1.01	0.7	1.29	0.7	0.18
1	CC005	F	Y	0.172	0.048	0.127	0.59	2.31	1.27	1.2	0.088
1	CC005	M	Y	0.087	0.087	0.017	2.05	1.1	1.53	1.48	0.199
1	CC005	F	Y	0.17	0.067	0.136	0.94	0.39	1.44	0.79	0.227
1	CC005	M	Y	0.077	0.079	0.134	1.67	1.8	1.36	1.19	0.121
2	CC005	F	Y	0.081	0.022	0.102	1.59	1.14	1.73	1.49	0.085
2	CC005	M	Y	0.134	0.084	0.158	1.47	1.01	1.58	1	0.143
2	CC005	M	Y	0.158	0.047	0.04	1.87	1.38	1.48	1.99	0.159
2	CC005	M	Y	0.134	0.084	0.158	1.47	1.01	1.58	1	0.143
2	CC005	F	Y	0.245	0.078	0.08	2.15	1.55	1.13	1.63	0.21
0	CC006	F	Y	0.135	0.078	0.134	1.18	1.28	1.48	1.34	0.127
0	CC006	M	Y	0.07	0.043	0.019	1.31	1.22	1.23	1.07	0.135
0	CC006	F	Y	0.117	0.063	0.118	1.67	1.82	1.72	1.39	0.13
1	CC006	F	Y	0.062	0.038	0.064	1.1	1.08	1.02	1.74	0.086
1	CC006	M	Y	0.04	0.04	0.047	1.13	1.11	1.05	1.28	0.09
1	CC006	F	Y	0.207	0.103	0.202	0.96	0.59	0.8	0.43	0.218
1	CC006	M	Y	0.168	0.072	0.198	2.24	0.58	1.16	0.32	0.191
2	CC006	F	Y	0.111	0.064	0.127	1.64	1.74	2.28	1.87	0.117

Trial#	Strain	Sex	Inf?	Propel_LH	Propel_RF	Propel_RH	SLVar_LF	SLVar_LH	SLVar_RF	SLVar_RH	Stance_LF
2	CC006	M	Y	0.055	0.038	0.071	1.37	1.1	1.28	1.43	0.096
0	CC011	F	Y	0.14	0.046	0.19	1.06	1.34	1.2	0.28	0.072
0	CC011	M	Y	0.106	0.068	0.097	1.38	1.24	1.42	1.12	0.143
0	CC011	F	Y	0.113	0.092	0.118	0.61	0.61	0.59	0.58	0.118
0	CC011	F	Y	0.02	0.057	0.033	0.98	1.57	1.02	1.05	0.145
1	CC011	F	Y	0.139	0.064	0.134	1.3	0.73	0.95	0.49	0.161
1	CC011	M	Y	0.153	0.159	0.137	0.66	1.43	0.92	0.58	0.195
1	CC011	F	Y	0.125	0.135	0.124	0.78	0.98	1.46	1.11	0.205
1	CC011	M	Y	0.109	0.051	0.134	0.74	0.41	0.95	0.81	0.155
2	CC011	F	Y	0.145	0.093	0.164	0.6	0.77	0.82	0.82	0.19
2	CC011	M	Y	0.173	0.049	0.226	2.26	1.8	1.67	1.43	0.162
2	CC011	F	Y	0.161	0.13	0.181	0.58	0.76	1.74	0.73	0.24
2	CC011	M	Y	0.165	0.11	0.138	1.08	0.46	0.87	0.22	0.208
0	CC012	M	Y	0.194	0.157	0.156	0.48	0.45	0.76	1.03	0.212
0	CC012	M	Y	0.092	0.063	0.118	1.04	1.67	1.87	2	0.223
0	CC012	M	Y	0.151	0.1	0.14	0.37	0.36	0.69	0.56	0.17
0	CC012	M	Y	0.159	0.099	0.147	1.66	1.29	1.67	1.8	0.17
0	CC012	M	Y	0.2	0.144	0.194	1.28	0.88	1.75	2.08	0.259
0	CC012	F	Y	0.131	0.055	0.151	1.16	1.66	1.27	0.88	0.114
0	CC012	F	Y	0.159	0.09	0.174	1.18	1.21	1.64	1.12	0.19
0	CC012	F	Y	0.094	0.099	0.113	1.17	1.78	1.74	1.38	0.122
0	CC012	F	Y	0.082	0.057	0.094	1.7	1.86	1.8	1.85	0.114
0	CC012	F	Y	0.071	0.045	0.056	0.46	0.45	0.77	0.83	0.074
2	CC012	M	Y	0.238	0.11	0.19	0.55	0.46	1.51	1.19	0.22
2	CC012	M	Y	0.201	0.156	0.215	1.06	0.7	0.5	0.64	0.251
2	CC012	M	Y	0.202	0.093	0.172	0.59	0.51	1.6	0.52	0.214
2	CC012	M	Y	0.25	0.125	0.22	0.65	0.88	1.91	1.33	0.301
2	CC012	M	Y	0.186	0.1	0.2	0.84	1.15	0.82	0.78	0.219
2	CC012	F	Y	0.145	0.144	0.182	1.32	1.65	1.67	1	0.193
2	CC012	F	Y	0.108	0.076	0.096	1.19	0.9	1.1	0.85	0.14
2	CC012	F	Y	0.121	0.066	0.103	1.83	1.65	1.81	1.56	0.133
2	CC012	F	Y	0.232	0.11	0.175	2.3	0.96	1.5	0.65	0.211
2	CC012	F	Y	0.132	0.113	0.108	0.98	1.23	1.76	1.32	0.172
1	CC012XCC032	F	Y	0.048	0.081	0.033	1.44	1.42	1.3	0.96	0.115
1	CC012xCC032	F	Y	0.036	0.044	0.023	0.83	0.9	0.98	0.73	0.069
1	CC012XCC032	M	Y	0.157	0.114	0.178	1.92	1.05	1.49	0.77	0.177
1	CC012xCC032	M	Y	0.141	0.08	0.154	1.98	2.25	1.99	1.98	0.137
1	CC012XCC032	M	Y	0.099	0.05	0.048	0.78	0.89	0.85	0.94	0.068
1	CC012xCC032	M	Y	0.048	0.059	0.054	2.18	1.18	0.85	1.17	0.12
2	CC012XCC032	F	Y	0.136	0.069	0.168	1.84	1.39	1.5	0.23	0.192
2	CC012XCC032	F	Y	0.164	0.231	0.136	0.54	0.34	2.95	2.38	0.265
2	CC012XCC032	M	Y	0.18	0.046	0.107	1.56	0.61	1.7	1.69	0.14
2	CC012XCC032	M	Y	0.154	0.095	0.147	1.79	1.43	1.62	0.65	0.109
2	CC012XCC032	M	Y	0.153	0.108	0.154	0.91	0.44	0.77	1.18	0.203
2	CC012XCC032	M	Y	0.186	0.07	0.182	1.79	1.1	1.05	2.45	0.199
1	CC013xCC041	F	Y	0.095	0.064	0.067	1.27	1.23	1.04	1.14	0.115
1	CC013xCC041	F	Y	0.033	0.036	0.03	0.66	0.58	0.86	0.65	0.057
1	CC013xCC041	F	Y	0.079	0.062	0.094	1.22	1.29	1.02	1.31	0.088

Trial#	Strain	Sex	Inf?	Propel_LH	Propel_RF	Propel_RH	SLVar_LF	SLVar_LH	SLVar_RF	SLVar_RH	Stance_LF
1	CC013xCC041	M	Y	0.127	0.063	0.085	0.94	1.54	1.12	1	0.122
1	CC013xCC041	M	Y	0.14	0.06	0.054	2.28	1.19	1.33	1.18	0.187
2	CC013XCC041	F	Y	0.135	0.102	0.19	0.85	0.28	0.75	0.49	0.204
2	CC013XCC041	F	Y	0.113	0.108	0.21	1.35	1.17	1.76	3.81	0.171
2	CC013XCC041	F	Y	0.207	0.069	0.232	0.98	0.83	0.6	0.48	0.191
2	CC013XCC041	M	Y	0.147	0.081	0.052	1.01	1.54	1.06	1.01	0.127
2	CC013XCC041	M	Y	0.097	0.042	0.039	0.94	1.71	0.76	0.67	0.078
0	CC015	F	Y	0.094	0.111	0.073	1.12	1.39	0.32	0.58	0.164
0	CC015	M	Y	0.154	0.112	0.145	0.7	0.33	0.74	0.26	0.195
0	CC015	M	Y	0.103	0.079	0.084	0.58	0.48	0.92	0.81	0.174
1	CC015	F	Y	0.126	0.109	0.086	0.86	0.48	0.5	1.32	0.19
1	CC015	M	Y	0.045	0.046	0.05	1.28	1.05	1.52	1.37	0.092
1	CC015	M	Y	0.034	0.056	0.05	1.21	0.73	1.53	1.31	0.114
1	CC015	M	Y	0.126	0.04	0.106	1.43	1.55	1.83	1.82	0.138
2	CC015	F	Y	0.097	0.085	0.1	1.27	0.53	0.7	0.24	0.179
0	CC017	F	Y	0.075	0.049	0.058	0.35	0.82	0.94	1.03	0.092
0	CC017	M	Y	0.085	0.066	0.114	0.58	1.09	0.51	0.98	0.152
0	CC017	F	Y	0.152	0.063	0.099	1.66	0.72	1.4	1.8	0.129
0	CC017	M	Y	0.137	0.049	0.116	1.28	1.29	1.74	0.91	0.14
1	CC017	M	Y	0.114	0.04	0.129	1.27	2.32	1.87	2.78	0.107
1	CC017	F	Y	0.08	0.061	0.093	1.06	1.68	1.91	1.6	0.101
0	CC023	M	Y	0.174	0.153	0.16	0.35	0.25	0.32	0.4	0.159
0	CC023	F	Y	0.179	0.085	0.042	1.99	0.48	1.97	1.52	0.141
0	CC023	F	Y	0.053	0.046	0.062	1.19	1.29	1.08	1.32	0.1
0	CC023	M	Y	0.172	0.024	0.092	0.79	0.68	1.4	0.67	0.038
1	CC023	M	Y	0.101	0.034	0.147	0.83	1.42	0.99	2.38	0.074
1	CC023	F	Y	0.105	0.039	0.147	1.95	1.4	1.18	3.05	0.086
1	CC023	M	Y	0.159	0.028	0.079	1.49	1.76	1.51	1.5	0.128
1	CC023	M	Y	0.119	0.073	0.117	1.44	0.28	1.4	1.42	0.173
2	CC023	M	Y	0.133	0.201	0.177	0.89	1.77	0.74	0.36	0.292
2	CC023	F	Y	0.192	0.086	0.125	2.26	1.77	1.68	2.84	0.196
2	CC023	M	Y	0.201	0.073	0.146	1.38	1.28	1.77	2.38	0.095
0	CC025	M	Y	0.104	0.087	0.091	0.97	1.25	0.9	0.8	0.137
0	CC025	F	Y	0.127	0.077	0.12	1.12	1.48	0.87	0.84	0.129
0	CC025	M	Y	0.187	0.092	0.151	1.1	0.29	1.25	1	0.191
1	CC025	M	Y	0.122	0.091	0.092	1.01	1.42	0.98	0.82	0.138
1	CC025	F	Y	0.092	0.061	0.095	0.93	1.11	0.83	1.18	0.097
1	CC025	F	Y	0.092	0.066	0.087	1.67	0.95	0.7	0.7	0.137
1	CC025	F	Y	0.146	0.098	0.101	1.33	0.68	1.32	1.24	0.162
2	CC025	F	Y	0.177	0.164	0.149	1.63	1.47	0.82	1	0.218
2	CC025	M	Y	0.193	0.135	0.238	1.04	0.6	0.81	0.73	0.251
0	CC027	F	Y	0.133	0.051	0.109	0.48	1.31	0.65	0.72	0.154
0	CC027	M	Y	0.163	0.041	0.206	1.69	1.02	1.56	1.85	0.113
1	CC027	M	Y	0.063	0.115	0.065	3.41	1.39	1.62	1	0.162
1	CC027	M	Y	0.063	0.115	0.065	3.41	1.39	1.62	1	0.162
1	CC027	F	Y	0.06	0.033	0.042	1.01	1.14	0.98	0.69	0.069
1	CC027	F	Y	0.103	0.058	0.052	1.28	1.73	1.34	1.43	0.146
1	CC027	M	Y	0.169	0.071	0.286	0.42	0.4	0.63	0.63	0.212

Trial#	Strain	Sex	Inf?	Propel_LH	Propel_RF	Propel_RH	SLVar_LF	SLVar_LH	SLVar_RF	SLVar_RH	Stance_LF
1	CC027	M	Y	0.189	0.121	0.174	0.53	0.68	1.22	0.73	0.234
2	CC027	F	Y	0.192	0.086	0.125	2.26	1.77	1.68	2.84	0.196
2	CC027	M	Y	0.098	0.148	0.173	1.99	1.73	1.23	1.26	0.164
2	CC027	F	Y	0.095	0.043	0.066	1.37	1.27	1.21	1.51	0.134
2	CC027	M	Y	0.214	0.109	0.193	2.3	0.8	0.78	0.48	0.192
1	CC032XCC013	M	Y	0.116	0.058	0.141	1.93	1.58	1.76	2.06	0.161
1	CC032XCC013	M	Y	0.173	0.134	0.168	1.57	0.69	1.04	1	0.203
1	CC032XCC013	M	Y	0.062	0.042	0.058	0.78	0.7	1.14	0.84	0.06
1	CC032XCC013	F	Y	0.066	0.087	0.089	0.74	1.23	1.31	1.58	0.083
1	CC032XCC013	F	Y	0.084	0.058	0.079	1.36	0.96	1.75	1.19	0.113
1	CC032XCC013	F	Y	0.139	0.1	0.118	1.57	1.38	0.68	0.49	0.155
1	CC032XCC013	F	Y	0.162	0.099	0.116	1.82	1.07	0.75	0.55	0.246
1	CC032XCC013	F	Y	0.08	0.144	0.149	0.71	0.93	0.81	0.56	0.17
2	CC032XCC013	M	Y	0.123	0.035	0.098	0.76	0.42	1.43	0.92	0.179
2	CC032XCC013	M	Y	0.136	0.024	0.081	1.79	1.76	1.24	0.84	0.134
2	CC032XCC013	M	Y	0.143	0.055	0.109	1.71	1.89	1.8	1.41	0.074
2	CC032XCC013	M	Y	0.119	0.028	0.105	1.19	0.51	1.38	1.68	0.184
2	CC032XCC013	F	Y	0.141	0.112	0.116	0.86	0.71	1	0.48	0.214
2	CC032XCC013	F	Y	0.117	0.104	0.128	1.12	0.65	1.24	0.53	0.15
2	CC032XCC013	F	Y	0.117	0.072	0.12	0.85	1.04	1.2	0.57	0.204
2	CC032XCC013	F	Y	0.187	0.14	0.173	1.21	0.39	0.96	1.24	0.208
0	CC037	F	Y	0.136	0.073	0.176	1.35	1.88	1.98	1.66	0.122
0	CC037	M	Y	0.095	0.055	0.14	1.08	1.45	0.95	1.82	0.079
0	CC037	M	Y	0.158	0.072	0.116	1.54	0.27	1.01	0.28	0.093
0	CC037	M	Y	0.077	0.076	0.028	0.96	2.48	0.96	0.92	0.129
1	CC037	F	Y	0.128	0.077	0.19	1.83	2.72	1.45	1.69	0.181
1	CC037	M	Y	0.143	0.136	0.125	1.2	0.65	1	0.44	0.18
1	CC037	M	Y	0.149	0.072	0.168	1.38	1.05	1.65	1.52	0.175
1	CC037	M	Y	0.145	0.122	0.134	1.06	0.61	0.68	2.08	0.226
2	CC037	M	Y	0.119	0.095	0.081	1.66	0.44	1.82	1	0.174
2	CC037	M	Y	0.179	0.159	0.111	0.78	1.2	1.03	0.41	0.259
1	CC041XCC012	M	Y	0.143	0.067	0.21	1.71	1.72	1.84	2.12	0.199
1	CC041XCC012	M	Y	0.167	0.069	0.156	2	0.25	0.78	0.6	0.119
1	CC041XCC012	M	Y	0.084	0.07	0.092	1.02	1.39	1.82	1.71	0.186
1	CC041XCC012	M	Y	0.105	0.096	0.111	1.22	1.43	2.14	1.65	0.118
1	CC041XCC012	M	Y	0.156	0.096	0.212	1	0.45	1.91	0.96	0.203
1	CC041XCC012	F	Y	0.148	0.082	0.136	1.33	1.02	2.48	1.36	0.173
1	CC041XCC012	F	Y	0.157	0.062	0.155	1.28	0.46	1.49	1.67	0.207
1	CC041XCC012	F	Y	0.194	0.086	0.177	1.24	0.63	1.18	1.5	0.227
1	CC041XCC012	F	Y	0.116	0.079	0.194	2.18	0.82	1.59	0.86	0.177
1	CC041XCC012	F	Y	0.175	0.061	0.16	1.22	1.21	2.09	1.75	0.218
1	CC041XCC012	F	Y	0.105	0.074	0.135	1.61	1.3	1.57	1.25	0.189
1	CC041XCC012	F	Y	0.136	0.114	0.141	0.78	0.49	0.55	1	0.192
1	CC041XCC012	F	Y	0.193	0.052	0.162	0.32	2.58	2.6	2.15	0.228
2	CC041XCC012	M	Y	0.152	0.102	0.142	1.2	0.48	0.43	1.4	0.228
2	CC041XCC012	M	Y	0.227	0.066	0.315	0.82	1.58	1.71	1.27	0.29
2	CC041XCC012	F	Y	0.203	0.117	0.215	0.57	0.57	1.78	0.5	0.246
2	CC041XCC012	M	Y	0.087	0.051	0.173	1.35	1.65	0.96	0.39	0.152

Trial#	Strain	Sex	Inf?	Propel_LH	Propel_RF	Propel_RH	SLVar_LF	SLVar_LH	SLVar_RF	SLVar_RH	Stance_LF
2	CC041XCC012	M	Y	0.172	0.122	0.204	0.4	0.66	0.97	1.24	0.236
2	CC041XCC012	M	Y	0.129	0.07	0.2	0.94	1.31	1.91	0.93	0.186
2	CC041XCC012	F	Y	0.176	0.127	0.25	0.73	0.84	1.01	0.71	0.283
2	CC041XCC012	F	Y	0.204	0.125	0.18	1.75	1.65	1.97	2.01	0.216
2	CC041XCC012	F	Y	0.158	0.075	0.138	1.02	0.51	1.61	0.68	0.248
2	CC041XCC012	F	Y	0.15	0.122	0.161	0.96	0.71	0.93	0.65	0.23
2	CC041XCC012	F	Y	0.174	0.12	0.183	0.76	1.34	1.66	1.24	0.226
2	CC041XCC012	F	Y	0.21	0.122	0.195	1.2	0.39	1.31	0.58	0.255
2	CC041XCC012	F	Y	0.223	0.096	0.222	1.03	0.96	1.5	1.04	0.228
0	CC051	F	Y	0.133	0.07	0.135	1.17	0.92	1.25	1.17	0.165
0	CC051	F	Y	0.161	0.067	0.113	1.37	1.78	1.86	1.54	0.138
0	CC051	F	Y	0.148	0.104	0.131	1.43	1.43	1.52	1.25	0.159
0	CC051	M	Y	0.127	0.081	0.087	1.26	0.42	0.62	0.63	0.152
1	CC051	F	Y	0.143	0.069	0.138	1.05	0.8	0.87	0.28	0.17
1	CC051	F	Y	0.134	0.072	0.121	0.67	0.74	1.46	0.52	0.202
1	CC051	F	Y	0.174	0.116	0.157	1.59	0.67	1.2	0.89	0.174
0	CC057	F	Y	0.123	0.13	0.15	1.1	0.68	1.03	0.48	0.182
0	CC057	F	Y	0.12	0.095	0.113	1.26	1.26	1.37	1.45	0.131
0	CC057	F	Y	0.082	0.089	0.073	1.55	1.03	1.51	1.02	0.131
0	CC057	M	Y	0.08	0.095	0.096	0.89	1.37	1.67	1.44	0.087
0	CC057	M	Y	0.027	0.061	0.043	1.98	0.74	1.45	0.84	0.162
0	CC057	M	Y	0.083	0.032	0.065	1.37	1.54	0.99	0.91	0.121
1	CC057	F	Y	0.147	0.119	0.152	1	0.27	0.36	0.19	0.189
1	CC057	F	Y	0.097	0.089	0.093	1.1	1.03	1.19	1.61	0.113
1	CC057	F	Y	0.087	0.037	0.056	1.39	1.31	1.07	1.23	0.105
1	CC057	F	Y	0.084	0.043	0.087	1.61	1.64	1.64	1.62	0.126
1	CC057	M	Y	0.184	0.104	0.184	2.4	0.97	2.08	0.43	0.19
1	CC057	M	Y	0.125	0.119	0.117	0.42	0.95	0.57	0.92	0.203
2	CC057	F	Y	0.084	0.043	0.087	1.61	1.64	1.64	1.62	0.126
0	CC078	F	Y	0.152	0.079	0.101	0.37	0.2	0.99	1.27	0.145
0	CC078	F	Y	0.088	0.117	0.122	0.64	0.94	0.72	1.22	0.165
0	CC078	F	Y	0.145	0.111	0.147	0.53	0.32	0.36	0.38	0.168
0	CC078	M	Y	0.117	0.113	0.106	1.22	0.55	0.75	0.48	0.167
1	CC078	F	Y	0.157	0.159	0.15	1.01	0.37	0.37	0.3	0.165
1	CC078	F	Y	0.182	0.118	0.123	1.46	0.46	1.29	1.33	0.177
1	CC078	M	Y	0.105	0.068	0.101	0.97	0.6	1.02	0.81	0.157
2	CC078	F	Y	0.117	0.066	0.097	1.69	2.04	1.51	2.13	0.165
2	CC078	M	Y	0.139	0.07	0.104	1.39	1.38	1.13	1.1	0.136
2	CC005	M	N	0.104	0.091	0.076	1.68	1.45	1.67	0.97	0.09
2	CC005	M	Y	0.107	0.058	0.085	1.79	2.29	1.96	2.36	0.09
2	CC015	F	N	0.062	0.046	0.071	0.84	1.65	0.93	1.12	0.085
2	CC015	M	N	0.168	0.108	0.135	1.35	0.96	1.35	0.54	0.208
2	CC015	M	Y	0.149	0.088	0.127	1.32	1.03	1.87	0.64	0.219
2	CC015	M	Y	0.079	0.068	0.079	1.29	1.17	1.15	1.39	0.136
2	CC017	F	N	0.15	0.068	0.16	1.44	0.74	1.77	1.97	0.191
2	CC017	M	N	0.091	0.038	0.099	1.06	1.35	1.06	1.55	0.076
2	CC017	F	Y	0.147	0.109	0.171	2.83	2.47	2.63	3.32	0.181
2	CC023	F	N	0.133	0.083	0.137	1.31	1.45	1.23	1.21	0.189

Trial#	Strain	Sex	Inf?	Propel_LH	Propel_RF	Propel_RH	SLVar_LF	SLVar_LH	SLVar_RF	SLVar_RH	Stance_LF

Table S3. Raw DigiGait measurement data. Far left column indicates time point at which data was measured: T0 = pre-infection, T1 = 21dpi, and T3 = 89dpi. DigiGait parameters listed across the top indicate which limb is associated with the data, where appropriate: FL for left fore limb, FR for right fore limb, HL for left hind limb, and HR for right hind limb.

Table S3

Trial#	Strain	Sex	Inf?	Stance_LH	Stance_RF	Stance_RH	Stance/Swing_LF	Stance/Swing_LH	Stance/Swing_RF
1	CC002	F	N	0.12	0.147	0.175	2.3	2.6	1.8
1	CC025	F	N	0.188	0.151	0.155	2.2	3.5	2.3
1	CC012XCC032	F	N	0.25	0.223	0.21	1.9	2.6	3
2	CC012XCC032	F	N	0.261	0.26	0.278	1.9	2	1.9
1	CC012XCC032	M	N	0.265	0.223	0.267	2.5	5.3	2.9
2	CC012XCC032	M	N	0.238	0.223	0.25	2	4.7	2
1	CC013xCC041	F	N	0.101	0.136	0.144	2.4	1.9	2.3
2	CC013XCC041	F	N	0.25	0.25	0.265	3.3	2.9	3.1
1	CC013xCC041	M	N	0.092	0.096	0.046	0.9	1.9	2
2	CC013XCC041	M	N	0.216	0.179	0.23	2.3	3.2	2.3
1	CC032XCC013	F	N	0.132	0.061	0.089	3.1	2.1	2
2	CC032XCC013	F	N	0.195	0.167	0.257	2.5	3.6	2
1	CC041XCC012	F	N	0.115	0.209	0.128	3.4	2	2.9
1	CC041XCC012	F	N	0.082	0.088	0.116	2.7	2.1	1.9
2	CC041XCC012	F	N	0.242	0.086	0.167	1.6	3.2	1.1
1	CC032XCC013	M	N	0.109	0.113	0.085	3.5	1.9	2.6
2	CC032XCC013	M	N	0.239	0.064	0.086	1	3.5	1.4
1	CC041XCC012	M	N	0.21	0.116	0.153	1.9	2.5	1.9
2	CC041XCC012	M	N	0.271	0.224	0.239	2.6	3	2.4
0	CC012	F	N	0.162	0.149	0.181	1.9	2.2	2.3
2	CC012	F	N	0.308	0.211	0.248	2.2	2.5	2.1
0	CC012	M	N	0.125	0.141	0.171	1.3	2.4	1.4
2	CC012	M	N	0.23	0.118	0.282	2.8	3.4	2.4
0	CC057	F	N	0.217	0.164	0.135	2.4	2.8	2.3
1	CC057	F	N	0.209	0.213	0.216	2.6	2.7	2.6
0	CC057	M	N	0.214	0.235	0.196	3	2.8	3.1
1	CC057	M	N	0.25	0.279	0.259	2.3	2.5	3.1
0	CC078	F	N	0.2	0.181	0.204	1.8	2.6	1.9
1	CC078	F	N	0.198	0.157	0.199	2	2.2	1.8
2	CC078	F	N	0.18	0.082	0.118	1.8	4.2	1.5
0	CC078	M	N	0.15	0.149	0.161	1.4	1.6	1.6
2	CC078	M	N	0.183	0.141	0.172	1.5	2.3	1.7
0	CC002	F	N	0.089	0.115	0.065	2.4	2.1	2.1
1	CC002	F	N	0.12	0.147	0.175	2.3	2.6	1.8
0	CC002	M	N	0.126	0.127	0.066	1.7	2.6	2.3
1	CC002	M	N	0.128	0.196	0.177	2.7	3.1	6.6
2	CC002	M	N	0.31	0.209	0.216	1.9	3.3	1.9
0	CC006	F	N	0.206	0.187	0.192	1.5	2.4	1.7
1	CC006	F	N	0.18	0.181	0.216	2	2.6	2.5
2	CC006	F	N	0.14	0.145	0.099	2.3	2.7	2.1
0	CC006	M	N	0.129	0.153	0.051	2.9	3.3	2.3
0	CC023	F	N	0.138	0.166	0.186	2.2	2.4	2
1	CC023	F	N	0.164	0.09	0.153	1.9	2.1	1.1
2	CC023	F	N	0.252	0.13	0.195	1.8	2.6	1.8
0	CC023	M	N	0.215	0.189	0.168	1.9	2.9	1.8
1	CC023	M	N	0.208	0.194	0.203	1.7	2.8	2.1
0	CC027	F	N	0.047	0.062	0.061	0.7	0.4	1.1

Trial#	Strain	Sex	Inf?	Stance_LH	Stance_RF	Stance_RH	Stance/Swing_LF	Stance/Swing_LH	Stance/Swing_RF
1	CC027	F	N	0.146	0.158	0.167	2.4	2.6	2.8
2	CC027	F	N	0.243	0.223	0.184	1.9	2.6	2.5
0	CC027	M	N	0.045	0.126	0.075	1	0.8	1.9
1	CC027	M	N	0.113	0.073	0.091	1.5	2.8	0.8
1	CC027	M	N	0.174	0.142	0.128	4.8	6.2	2.6
2	CC027	M	N	0.167	0.145	0.154	1.7	1.8	1.6
0	CC005	F	N	0.188	0.156	0.188	1.8	2.8	1.6
1	CC005	F	N	0.167	0.153	0.158	2.1	3	2.2
2	CC005	F	N	0.056	0.095	0.061	2	1.4	3.7
0	CC011	F	N	0.109	0.088	0.135	1.3	2.4	1.6
1	CC011	F	N	0.249	0.106	0.222	1.4	4	1.3
2	CC011	F	N	0.245	0.198	0.249	2	2.8	1.9
1	CC017	F	N	0.187	0.171	0.172	2.6	3	3
0	CC005	M	N	0.207	0.176	0.205	2	2.8	1.9
1	CC005	M	N	0.097	0.131	0.205	2	1.9	1.8
2	CC005	M	N	0.135	0.111	0.179	2.4	3.4	1.7
0	CC011	M	N	0.176	0.149	0.156	1.9	2.9	2.1
1	CC011	M	N	0.19	0.188	0.196	1.6	2.7	2.6
2	CC011	M	N	0.185	0.12	0.162	2.2	3.6	2.2
0	CC017	M	N	0.057	0.093	0.059	1.3	0.8	1.6
1	CC017	M	N	0.2	0.269	0.145	1.6	2.9	3.3
1	CC006	F	N	0.033	0.048	0.06	1.4	0.6	1.1
0	CC037	F	N	0.21	0.146	0.161	1.4	2.6	1.6
0	CC051	F	N	0.224	0.127	0.221	1.7	2.7	1.6
1	CC051	F	N	0.13	0.173	0.171	1.8	3	2.4
1	CC006	M	N	0.198	0.168	0.215	1.6	1.9	1.6
0	CC037	M	N	0.202	0.174	0.203	1.5	2.4	1.6
1	CC037	M	N	0.177	0.198	0.193	1.7	1.9	2.4
0	CC005	F	N	0.196	0.155	0.175	1.8	3.3	2.4
1	CC005	F	N	0.281	0.242	0.278	3.1	3.3	3
2	CC005	F	N	0.218	0.085	0.174	1.8	1.9	0.8
0	CC011	F	N	0.183	0.168	0.177	2.2	3	2.4
1	CC011	F	N	0.219	0.161	0.209	2	3.2	1.5
2	CC011	F	N	0.218	0.211	0.225	3.3	3.3	2.9
0	CC011	M	N	0.188	0.18	0.198	1.8	2.4	1.9
1	CC011	M	N	0.215	0.186	0.216	1.7	2.4	1.5
2	CC011	M	N	0.218	0.192	0.211	1.6	2.9	1.7
1	CC037	M	N	0.226	0.128	0.224	2.4	3.2	1.9
2	CC037	M	N	0.223	0.092	0.278	1.3	3.6	0.9
1	CC051	M	N	0.213	0.202	0.232	1.9	2.8	2.1
2	CC051	M	N	0.189	0.19	0.239	1.7	1.8	2.6
0	CC027	F	N	0.28	0.24	0.246	1.3	3.4	2.1
1	CC027	F	N	0.147	0.141	0.129	1.9	2.4	1.6
2	CC027	F	N	0.195	0.182	0.153	2.5	3.3	2.5
0	CC015	M	N	0.176	0.179	0.182	3	2	2.2
1	CC015	M	N	0.14	0.155	0.087	1.2	1	2.3
2	CC015	M	N	0.23	0.2	0.201	2.2	2.2	2.2
0	CC027	M	N	0.191	0.208	0.206	2.2	2.7	2.5

Trial#	Strain	Sex	Inf?	Stance_LH	Stance_RF	Stance_RH	Stance/Swing_LF	Stance/Swing_LH	Stance/Swing_RF
1	CC027	M	N	0.234	0.22	0.186	1.4	2.6	1.8
2	CC027	M	N	0.118	0.177	0.158	1.6	1.6	1.8
0	CC015	F	N	0.187	0.23	3.2	2.5	1.8	3.6
1	CC015	F	N	0.09	0.09	0.076	2.7	2.1	2.5
0	CC017	F	N	0.179	0.171	0.168	1.5	1.8	2
0	CC023	F	N	0.168	0.164	0.175	1.8	2.6	2.3
1	CC023	F	N	0.207	0.175	0.199	2	2.6	2.1
0	CC005	M	N	0.158	0.168	0.124	1.9	2.4	1.8
1	CC005	M	N	0.282	0.268	0.164	2.5	3.4	3.3
0	CC015	M	N	0.219	0.198	0.22	1.9	3	2.1
1	CC015	M	N	0.22	0.106	0.072	2.6	2.8	2.7
0	CC017	M	N	0.2	0.165	0.205	2.6	4.3	2.3
1	CC017	M	N	0.079	0.08	0.098	2.1	2.1	2.1
0	CC023	M	N	0.227	0.211	0.23	1.5	2.5	1.8
1	CC023	M	N	0.212	0.213	0.237	1.6	2.4	2.1
0	CC051	M	N	0.254	0.197	0.216	2.2	3.3	1.8
0	CC002	F	Y	0.127	0.096	0.096	1.4	4.1	2
0	CC002	M	Y	0.283	0.146	0.18	2	3.2	2.6
1	CC002	M	Y	0.061	0.147	0.032	1.6	1.1	1.9
1	CC002	F	Y	0.078	0.1	0.055	1.9	1	2.5
1	CC002	F	Y	0.135	0.105	0.112	2.1	2.7	2.3
1	CC002	M	Y	0.061	0.147	0.032	1.6	1.1	1.9
1	CC002	M	Y	0.113	0.116	0.083	2.9	2.7	2.8
1	CC002	F	Y	0.078	0.1	0.055	1.9	1	2.5
1	CC002	F	Y	0.046	0.096	0.049	2.8	0.9	2.2
2	CC002	M	Y	0.18	0.093	0.107	1.8	1.9	1.7
2	CC002	F	Y	0.236	0.219	0.257	2	2.9	2.4
2	CC002	M	Y	0.228	0.107	0.135	2.8	2.9	2.7
0	CC005	F	Y	0.204	0.133	0.196	1.4	3.2	1.4
0	CC005	M	Y	0.201	0.156	0.21	1.9	2.6	1.8
0	CC005	M	Y	0.202	0.166	0.203	2.8	3.1	2.1
1	CC005	F	Y	0.2	0.106	0.162	1.6	3	1.6
1	CC005	M	Y	0.117	0.117	0.03	2.7	1.8	3.1
1	CC005	F	Y	0.227	0.236	0.164	1.8	1.9	2.2
1	CC005	M	Y	0.112	0.181	0.166	2	2.3	1.8
2	CC005	F	Y	0.122	0.101	0.135	1.4	3.4	2
2	CC005	M	Y	0.24	0.206	0.209	2.1	3.4	2.9
2	CC005	M	Y	0.193	0.114	0.075	1.8	4	1.9
2	CC005	M	Y	0.24	0.206	0.209	2.1	3.4	2.9
2	CC005	F	Y	0.311	0.277	0.126	2.1	3.6	3.3
0	CC006	F	Y	0.174	0.123	0.177	1.8	2.4	1.8
0	CC006	M	Y	0.098	0.073	0.03	2.3	1.4	1.3
0	CC006	F	Y	0.155	0.116	0.162	1.9	2.8	1.5
1	CC006	F	Y	0.098	0.079	0.113	1.8	2.8	1.4
1	CC006	M	Y	0.069	0.084	0.085	3.8	2.3	2.2
1	CC006	F	Y	0.23	0.2	0.225	1.9	2.2	1.5
1	CC006	M	Y	0.237	0.226	0.262	1.7	2.2	2.2
2	CC006	F	Y	0.127	0.139	0.143	1.5	1.7	2

Trial#	Strain	Sex	Inf?	Stance_LH	Stance_RF	Stance_RH	Stance/Swing_LF	Stance/Swing_LH	Stance/Swing_RF
2	CC006	M	Y	0.083	0.147	0.097	2.7	2.1	3.1
0	CC011	F	Y	0.148	0.069	0.196	1.1	3.8	1.4
0	CC011	M	Y	0.141	0.119	0.13	2.4	3.2	2.3
0	CC011	F	Y	0.142	0.139	0.142	1.6	2	1.8
0	CC011	F	Y	0.036	0.114	0.053	2.1	0.3	1.3
1	CC011	F	Y	0.2	0.163	0.189	2.1	2.8	2.1
1	CC011	M	Y	0.224	0.227	0.2	2.6	3.1	3.7
1	CC011	F	Y	0.208	0.209	0.179	2.3	2.4	2.6
1	CC011	M	Y	0.185	0.176	0.202	1.8	2.6	2.5
2	CC011	F	Y	0.205	0.18	0.207	2.2	2.6	2
2	CC011	M	Y	0.207	0.114	0.278	3.3	3.9	3.1
2	CC011	F	Y	0.24	0.237	0.23	2.8	3.6	2.8
2	CC011	M	Y	0.252	0.222	0.23	1.7	2.4	1.8
0	CC012	M	Y	0.236	0.217	0.198	1.7	2.5	1.8
0	CC012	M	Y	0.15	0.135	0.158	2.1	1.7	1.8
0	CC012	M	Y	0.18	0.163	0.18	1.9	2.1	1.6
0	CC012	M	Y	0.184	0.162	0.189	1.9	2	1.5
0	CC012	M	Y	0.252	0.217	0.211	2.2	2	2
0	CC012	F	Y	0.175	0.108	0.205	1.6	2.6	1.2
0	CC012	F	Y	0.215	0.184	0.202	2.2	2.8	2
0	CC012	F	Y	0.133	0.18	0.169	2.8	1.8	2
0	CC012	F	Y	0.112	0.101	0.116	1.3	1.5	1.5
0	CC012	F	Y	0.083	0.084	0.069	1.7	1.2	1.7
2	CC012	M	Y	0.256	0.183	0.231	2	2.6	1.7
2	CC012	M	Y	0.244	0.238	0.251	2.2	1.8	1.6
2	CC012	M	Y	0.23	0.215	0.235	1.8	2.2	1.7
2	CC012	M	Y	0.304	0.291	0.323	2.5	2.6	2.1
2	CC012	M	Y	0.257	0.213	0.245	1.7	2.3	1.9
2	CC012	F	Y	0.282	0.231	0.266	1.9	3.2	1.5
2	CC012	F	Y	0.167	0.182	0.165	1.5	1.9	2.2
2	CC012	F	Y	0.199	0.125	0.154	2	2.4	1.9
2	CC012	F	Y	0.294	0.175	0.248	1.9	2.4	2.7
2	CC012	F	Y	0.217	0.192	0.217	2.4	2.5	2.7
1	CC012XCC032	F	Y	0.088	0.109	0.063	3.6	1.9	2.8
1	CC012xCC032	F	Y	0.064	0.085	0.049	2.4	1.5	2
1	CC012XCC032	M	Y	0.231	0.181	0.23	2.3	2.8	2.3
1	CC012xCC032	M	Y	0.172	0.158	0.194	1.6	2.1	1.8
1	CC012XCC032	M	Y	0.113	0.099	0.067	1.4	2.3	2.5
1	CC012xCC032	M	Y	0.105	0.097	0.081	3.7	2.7	4.6
2	CC012XCC032	F	Y	0.234	0.138	0.284	2.6	2.3	1.7
2	CC012XCC032	F	Y	0.253	0.325	0.302	2.4	2.2	2.9
2	CC012XCC032	M	Y	0.256	0.098	0.171	2.1	2.9	1.3
2	CC012XCC032	M	Y	0.212	0.158	0.229	1.3	2	1.9
2	CC012XCC032	M	Y	0.237	0.187	0.235	2.1	3.3	1.5
2	CC012XCC032	M	Y	0.2	0.189	0.324	2.2	1.7	2.2
1	CC013xCC041	F	Y	0.116	0.164	0.099	2.2	2	2.3
1	CC013xCC041	F	Y	0.05	0.075	0.054	2.3	2	2.1
1	CC013xCC041	F	Y	0.115	0.09	0.123	2.2	3.5	2.1

Trial#	Strain	Sex	Inf?	Stance_LH	Stance_RF	Stance_RH	Stance/Swing_LF	Stance/Swing_LH	Stance/Swing_RF
1	CC013xCC041	M	Y	0.143	0.133	0.151	2.7	3.1	3.6
1	CC013xCC041	M	Y	0.195	0.097	0.087	2.6	3.5	2.3
2	CC013XCC041	F	Y	0.214	0.198	0.22	2.3	2.6	2.2
2	CC013XCC041	F	Y	0.203	0.197	0.33	2.5	3	3.3
2	CC013XCC041	F	Y	0.242	0.24	0.27	1.9	2.9	2.4
2	CC013XCC041	M	Y	0.217	0.161	0.176	2.7	4.1	3.1
2	CC013XCC041	M	Y	0.111	0.076	0.064	2.6	3.5	2.8
0	CC015	F	Y	0.144	0.162	0.176	2.1	2.1	2.3
0	CC015	M	Y	0.219	0.2	0.214	2	3.3	2.4
0	CC015	M	Y	0.187	0.163	0.163	2.2	3.1	2.2
1	CC015	F	Y	0.19	0.194	0.156	2.2	2.1	2.2
1	CC015	M	Y	0.083	0.11	0.08	2.7	2.5	3.1
1	CC015	M	Y	0.075	0.122	0.091	3.1	2.1	2.3
1	CC015	M	Y	0.19	0.12	0.156	2.2	3.3	2.3
2	CC015	F	Y	0.199	0.206	0.195	2.6	2.7	3.2
0	CC017	F	Y	0.101	0.082	0.083	1.4	1.7	1.5
0	CC017	M	Y	0.096	0.129	0.151	2.3	2.1	1.4
0	CC017	F	Y	0.206	0.125	0.188	2	3.6	2.3
0	CC017	M	Y	0.213	0.089	0.163	1.8	2.8	1.4
1	CC017	M	Y	0.156	0.105	0.175	1.4	3	1.3
1	CC017	F	Y	0.114	0.156	0.123	1.8	1.6	3.5
0	CC023	M	Y	0.199	0.178	0.185	1.4	2.5	1.6
0	CC023	F	Y	0.214	0.159	0.081	1.6	2.5	1.8
0	CC023	F	Y	0.091	0.078	0.087	1.5	1.6	1.5
0	CC023	M	Y	0.196	0.08	0.147	0.5	5.1	1.8
1	CC023	M	Y	0.13	0.071	0.178	2.4	4.8	1.7
1	CC023	F	Y	0.129	0.071	0.21	1.5	2.6	1.3
1	CC023	M	Y	0.225	0.096	0.133	2.9	4.6	2.2
1	CC023	M	Y	0.176	0.156	0.197	2	2.3	2
2	CC023	M	Y	0.153	0.263	0.232	4.6	1.1	3.1
2	CC023	F	Y	0.23	0.139	0.255	2.5	3.1	1.9
2	CC023	M	Y	0.228	0.134	0.177	1.6	1.9	2.2
0	CC025	M	Y	0.164	0.149	0.141	2	3.4	2.3
0	CC025	F	Y	0.197	0.172	0.177	1.5	4.3	2.2
0	CC025	M	Y	0.202	0.155	0.185	2.3	3.2	2.1
1	CC025	M	Y	0.18	0.152	0.144	2.1	3.6	2.6
1	CC025	F	Y	0.122	0.101	0.13	1.1	2.2	1.1
1	CC025	F	Y	0.151	0.127	0.141	1.8	2.6	1.9
1	CC025	F	Y	0.199	0.145	0.148	1.9	2.2	1.7
2	CC025	F	Y	0.245	0.243	0.21	2.4	2.5	2.4
2	CC025	M	Y	0.3	0.27	0.278	2	3.2	2.4
0	CC027	F	Y	0.162	0.115	0.139	1.9	2.8	1.5
0	CC027	M	Y	0.184	0.093	0.236	1.7	2.4	1.4
1	CC027	M	Y	0.134	0.151	0.106	9.1	2.4	2.9
1	CC027	M	Y	0.134	0.151	0.106	9.1	2.4	2.9
1	CC027	F	Y	0.079	0.069	0.063	1.1	1.4	1.2
1	CC027	F	Y	0.184	0.108	0.079	1.5	2.4	1.4
1	CC027	M	Y	0.339	0.319	0.355	2.7	4.3	3.4

Trial#	Strain	Sex	Inf?	Stance_LH	Stance_RF	Stance_RH	Stance/Swing_LF	Stance/Swing_LH	Stance/Swing_RF
1	CC027	M	Y	0.251	0.228	0.237	2.5	3.1	2.3
2	CC027	F	Y	0.23	0.139	0.255	2.5	3.1	1.9
2	CC027	M	Y	0.147	0.234	0.242	1.8	2.3	2.1
2	CC027	F	Y	0.164	0.099	0.121	2.3	2.3	1.9
2	CC027	M	Y	0.271	0.245	0.277	1.8	3	2
1	CC032XCC013	M	Y	0.185	0.132	0.174	2.1	2.6	2.3
1	CC032XCC013	M	Y	0.247	0.21	0.211	2.6	4.3	2.5
1	CC032XCC013	M	Y	0.091	0.083	0.087	1.2	1.6	1.4
1	CC032XCC013	F	Y	0.107	0.153	0.12	1.8	2	3.3
1	CC032XCC013	F	Y	0.138	0.108	0.132	2.5	1.6	1.5
1	CC032XCC013	F	Y	0.212	0.189	0.186	2.3	2.5	2.3
1	CC032XCC013	F	Y	0.244	0.185	0.186	2.9	2.6	2.1
1	CC032XCC013	F	Y	0.166	0.168	0.203	2.3	3.2	1.3
2	CC032XCC013	M	Y	0.196	0.157	0.201	3	3.3	2.6
2	CC032XCC013	M	Y	0.16	0.082	0.13	2	2.2	1.3
2	CC032XCC013	M	Y	0.202	0.116	0.146	1	2.8	3.5
2	CC032XCC013	M	Y	0.208	0.082	0.168	2.1	1.8	1.2
2	CC032XCC013	F	Y	0.248	0.196	0.219	2.4	3.7	1.9
2	CC032XCC013	F	Y	0.215	0.187	0.217	2.1	2.9	2.5
2	CC032XCC013	F	Y	0.203	0.122	0.21	3	2.7	1.5
2	CC032XCC013	F	Y	0.264	0.195	0.239	2.2	3.6	1.9
0	CC037	F	Y	0.18	0.13	0.229	1.9	3	1.9
0	CC037	M	Y	0.13	0.09	0.199	1.3	1.8	1.6
0	CC037	M	Y	0.208	0.129	0.141	1.8	2.1	1.6
0	CC037	M	Y	0.104	0.102	0.057	1.3	0.6	1.5
1	CC037	F	Y	0.254	0.214	0.24	2.1	3	2.2
1	CC037	M	Y	0.189	0.19	0.198	1.8	1.7	1.6
1	CC037	M	Y	0.245	0.166	0.23	2.3	2.4	2.1
1	CC037	M	Y	0.25	0.196	0.187	2	2.6	1.8
2	CC037	M	Y	0.238	0.143	0.217	2.3	3.2	2.1
2	CC037	M	Y	0.272	0.247	0.215	2	2.3	1.9
1	CC041XCC012	M	Y	0.173	0.152	0.234	3.3	4.1	1.6
1	CC041XCC012	M	Y	0.22	0.202	0.212	1.4	3.4	3
1	CC041XCC012	M	Y	0.12	0.149	0.116	3	1.9	2.8
1	CC041XCC012	M	Y	0.155	0.177	0.137	2.9	2.7	2.8
1	CC041XCC012	M	Y	0.238	0.189	0.245	2.4	2.9	2.1
1	CC041XCC012	F	Y	0.272	0.171	0.223	2.6	3.3	2.2
1	CC041XCC012	F	Y	0.225	0.127	0.2	2	2.8	2
1	CC041XCC012	F	Y	0.249	0.147	0.227	2.5	2.6	2.5
1	CC041XCC012	F	Y	0.259	0.205	0.235	2.6	3.6	2.9
1	CC041XCC012	F	Y	0.227	0.15	0.195	2.9	2.6	1.9
1	CC041XCC012	F	Y	0.222	0.154	0.197	2	2.6	1.9
1	CC041XCC012	F	Y	0.21	0.211	0.227	1.5	2.8	2.6
1	CC041XCC012	F	Y	0.249	0.139	0.21	3.6	3.7	1.9
2	CC041XCC012	M	Y	0.233	0.232	0.214	2.6	3	2.5
2	CC041XCC012	M	Y	0.332	0.232	0.352	1.8	2.6	1.9
2	CC041XCC012	F	Y	0.278	0.218	0.249	1.7	2.6	1.7
2	CC041XCC012	M	Y	0.125	0.177	0.234	3.2	1.2	2.4

Trial#	Strain	Sex	Inf?	Stance_LH	Stance_RF	Stance_RH	Stance/Swing_LF	Stance/Swing_LH	Stance/Swing_RF
2	CC041XCC012	M	Y	0.244	0.253	0.263	2.1	2.3	2.7
2	CC041XCC012	M	Y	0.144	0.202	0.24	2.4	1	2.3
2	CC041XCC012	F	Y	0.303	0.299	0.325	2.2	2.6	2.3
2	CC041XCC012	F	Y	0.265	0.244	0.251	2.7	3.9	2.7
2	CC041XCC012	F	Y	0.275	0.232	0.24	2.4	3	2.5
2	CC041XCC012	F	Y	0.232	0.234	0.236	2.1	2.1	2.1
2	CC041XCC012	F	Y	0.291	0.235	0.291	2.4	3.2	2.5
2	CC041XCC012	F	Y	0.292	0.275	0.286	2.5	3.5	3.1
2	CC041XCC012	F	Y	0.261	0.219	0.261	1.9	2.9	2.2
0	CC051	F	Y	0.211	0.162	0.207	2	3.1	2.1
0	CC051	F	Y	0.207	0.107	0.195	2.2	3.5	1.3
0	CC051	F	Y	0.189	0.177	0.201	1.8	3	2.3
0	CC051	M	Y	0.176	0.147	0.17	2	3	2.5
1	CC051	F	Y	0.223	0.2	0.226	2	2.8	1.9
1	CC051	F	Y	0.215	0.156	0.213	2.6	3	2
1	CC051	F	Y	0.259	0.196	0.215	2	3.4	2.2
0	CC057	F	Y	0.195	0.19	0.192	2.4	2.8	2.6
0	CC057	F	Y	0.155	0.17	0.15	1.9	2.3	2.5
0	CC057	F	Y	0.138	0.133	0.108	2.9	3.4	3.6
0	CC057	M	Y	0.148	0.161	0.145	2.9	3.7	2.1
0	CC057	M	Y	0.064	0.131	0.063	3.4	1.5	3.6
0	CC057	M	Y	0.117	0.081	0.096	3.1	2.7	2.1
1	CC057	F	Y	0.164	0.182	0.194	3.5	2.1	3
1	CC057	F	Y	0.138	0.136	0.116	2.3	3.6	3.5
1	CC057	F	Y	0.11	0.095	0.085	2.7	4.3	4.3
1	CC057	F	Y	0.114	0.108	0.12	3	2.4	2.1
1	CC057	M	Y	0.196	0.169	0.247	2.5	2.1	1.9
1	CC057	M	Y	0.193	0.201	0.197	3.3	2.7	3.8
2	CC057	F	Y	0.114	0.108	0.12	3	2.4	2.1
0	CC078	F	Y	0.166	0.154	0.138	1.5	1.9	1.8
0	CC078	F	Y	0.141	0.16	0.154	1.9	2.2	1.8
0	CC078	F	Y	0.176	0.161	0.176	1.8	2.3	1.7
0	CC078	M	Y	0.15	0.164	0.173	2.1	1.6	2
1	CC078	F	Y	0.209	0.213	0.193	2	2.8	3.3
1	CC078	F	Y	0.204	0.166	0.14	2.7	2.9	2.1
1	CC078	M	Y	0.147	0.162	0.175	1.7	1.6	2.2
2	CC078	F	Y	0.176	0.136	0.142	2.1	3	2.4
2	CC078	M	Y	0.178	0.163	0.189	1.6	1.9	2.5
2	CC005	M	N	0.13	0.183	0.1	1.7	4.1	5.6
2	CC005	M	Y	0.137	0.095	0.159	1	1.9	1.4
2	CC015	F	N	0.102	0.065	0.114	2.3	1.8	1.5
2	CC015	M	N	0.259	0.237	0.245	2.1	2.7	2.2
2	CC015	M	Y	0.258	0.194	0.221	3	3.6	2
2	CC015	M	Y	0.17	0.117	0.141	3.1	3.3	2.3
2	CC017	F	N	0.239	0.112	0.218	2.3	2.5	1.7
2	CC017	M	N	0.133	0.066	0.146	1.2	2	1
2	CC017	F	Y	0.177	0.238	0.195	3	1.8	2.6
2	CC023	F	N	0.211	0.231	0.191	2.1	2.2	2.1

Trial#	Strain	Sex	Inf?	Stance_LH	Stance_RF	Stance_RH	Stance/Swing_LF	Stance/Swing_LH	Stance/Swing_RF

Table S3. Raw DigiGait measurement data. Far left column indicates time point at which data was measured: T0 = pre-infection, T1 = 21dpi, and T3 = 89dpi. DigiGait parameters listed across the top indicate which limb is associated with the data, where appropriate: FL for left fore limb, FR for right fore limb, HL for left hind limb, and HR for right hind limb.

Table S3

Trial#	Strain	Sex	Inf?	Stance/Swing_RH	StanceFactor_LF	StanceFactor_LH	StanceWidth_LF	StanceWidth_LH
1	CC002	F	N	2.8	1.03	0.69	1.4	2.1
1	CC025	F	N	2.9	0.95	1.22	1.5	2
1	CC012XCC032	F	N	2.3	0.86	1.19	1.6	2.9
2	CC012XCC032	F	N	2.8	0.81	0.94	1.6	2.9
1	CC012XCC032	M	N	4.8	0.84	0.99	1.1	1.9
2	CC012XCC032	M	N	5.6	1.02	0.95	1	1.7
1	CC013xCC041	F	N	3	1.29	0.7	1.6	2.6
2	CC013XCC041	F	N	3.5	1.01	0.95	2	3
1	CC013xCC041	M	N	1.8	0.47	2	1.2	2.6
2	CC013XCC041	M	N	3.2	0.99	0.94	1.6	2.4
1	CC032XCC013	F	N	3.1	1.51	1.48	1.1	2.2
2	CC032XCC013	F	N	3.8	1.04	0.76	1	2.2
1	CC041XCC012	F	N	2.2	0.69	0.9	1.6	2
1	CC041XCC012	F	N	2.2	1.01	0.71	1.5	1.7
2	CC041XCC012	F	N	2.6	1.5	1.45	1.1	2.6
1	CC032XCC013	M	N	1.7	1.01	1.29	2.4	2.1
2	CC032XCC013	M	N	1.4	1.05	2.77	1.3	2.7
1	CC041XCC012	M	N	2.4	1.47	1.38	1.5	3
2	CC041XCC012	M	N	2	1.01	1.13	1.2	3
0	CC012	F	N	2.5	0.87	0.9	1.8	2.3
2	CC012	F	N	1.9	1.39	1.24	1.4	2.5
0	CC012	M	N	2.8	0.84	0.73	1.2	1.8
2	CC012	M	N	4.7	1.1	0.82	1.1	3
0	CC057	F	N	2.6	1.19	1.61	2.1	3.1
1	CC057	F	N	2.8	1.01	0.97	2.1	3.5
0	CC057	M	N	3.2	0.66	1.09	2	2.9
1	CC057	M	N	2.8	0.87	0.96	1.9	3.5
0	CC078	F	N	2.6	1	0.98	1.4	1.9
1	CC078	F	N	2.4	1.2	0.99	1.6	1.8
2	CC078	F	N	1.7	1.28	1.53	1.4	1.7
0	CC078	M	N	1.9	0.96	0.94	1.1	1.7
2	CC078	M	N	2.1	1.06	1.06	1.9	2.2
0	CC002	F	N	1.7	1.33	1.38	1.2	1.5
1	CC002	F	N	2.8	1.03	0.69	1.4	2.1
0	CC002	M	N	1.6	0.68	1.91	1.3	1.7
1	CC002	M	N	2.4	0.54	0.73	1.5	1.8
2	CC002	M	N	3.2	1.11	1.44	1.2	2.2
0	CC006	F	N	2	0.94	1.08	1.6	1.9
1	CC006	F	N	2.3	0.71	0.83	1.2	2.5
2	CC006	F	N	1.7	0.66	1.42	1.6	2.2
0	CC006	M	N	1.2	0.91	2.54	1.3	1.7
0	CC023	F	N	2.8	1.04	0.74	1.7	2
1	CC023	F	N	1.8	1.46	1.07	1.7	2.2
2	CC023	F	N	1.9	1.58	1.29	1.7	2
0	CC023	M	N	2	0.99	1.27	1.2	2.4
1	CC023	M	N	2.5	0.86	1.02	1.2	2.4
0	CC027	F	N	0.8	1.29	0.77	2.1	1.7

Trial#	Strain	Sex	Inf?	Stance/Swing_RH	StanceFactor_LF	StanceFactor_LH	StanceWidth_LF	StanceWidth_LH
1	CC027	F	N	2.2	1.08	0.88	1.1	2
2	CC027	F	N	2.3	0.87	1.32	1.8	2.6
0	CC027	M	N	0.7	0.78	0.6	1.7	2.1
1	CC027	M	N	2.8	1.12	1.23	1.8	2.4
1	CC027	M	N	3	1.6	1.36	1.3	1.9
2	CC027	M	N	1.7	1.23	1.08	2.2	2.4
0	CC005	F	N	2.8	0.8	1	1.4	2
1	CC005	F	N	3.2	0.84	1.06	1.3	2.4
2	CC005	F	N	1.6	0.83	0.92	1.9	1.5
0	CC011	F	N	3.4	0.95	0.81	1.1	1.6
1	CC011	F	N	3.8	1.16	1.12	1.1	1.8
2	CC011	F	N	3.1	1.02	0.98	1.2	1.8
1	CC017	F	N	3.8	0.76	1.09	1.7	2.1
0	CC005	M	N	2.8	1.07	1.01	1.6	2.3
1	CC005	M	N	3.1	0.93	0.47	0.9	2.4
2	CC005	M	N	5.6	1.04	0.75	0.8	2.1
0	CC011	M	N	2.5	0.96	1.12	1.4	1.5
1	CC011	M	N	2.6	0.86	0.97	1.6	1.8
2	CC011	M	N	4	1.22	1.14	1.8	2
0	CC017	M	N	0.7	1.17	0.96	1.8	1.9
1	CC017	M	N	2.2	0.38	1.38	1.4	2.8
1	CC006	F	N	1.2	1.28	0.55	0.9	1.1
0	CC037	F	N	1.5	1.08	1.31	1	1.9
0	CC051	F	N	3.3	0.97	1.01	1.4	2.2
1	CC051	F	N	3.3	0.56	0.76	1	2.3
1	CC006	M	N	2.1	0.95	0.92	1	2.4
0	CC037	M	N	2.4	0.79	0.99	1.4	2.1
1	CC037	M	N	3.5	0.8	0.92	1.7	2.3
0	CC005	F	N	2.6	0.86	1.12	1.3	2.1
1	CC005	F	N	2.9	0.98	1.01	2.2	2.8
2	CC005	F	N	1	2.6	1.25	1.4	2.2
0	CC011	F	N	2.9	1	1.03	1.4	1.7
1	CC011	F	N	3	1.13	1.05	2.1	2.4
2	CC011	F	N	3.8	1.07	0.97	1.5	1.8
0	CC011	M	N	2.7	0.94	0.95	2.2	2.9
1	CC011	M	N	2.4	1.05	1	2.1	3.3
2	CC011	M	N	2.2	0.94	1.03	1.6	2.8
1	CC037	M	N	3.5	1.62	1.01	1.3	2.6
2	CC037	M	N	4.7	1.56	0.8	1.4	2.7
1	CC051	M	N	3.3	0.97	0.92	2.7	3.3
2	CC051	M	N	3.3	0.86	0.79	1.4	2
0	CC027	F	N	2.2	0.82	1.14	1.5	2.4
1	CC027	F	N	1.5	1.34	1.14	1.5	2.2
2	CC027	F	N	1.3	0.9	1.28	1.6	2.4
0	CC015	M	N	2.2	1.07	0.97	2	1.6
1	CC015	M	N	0.7	0.83	1.62	2.3	1.9
2	CC015	M	N	1.5	1.16	1.14	1.8	2.3
0	CC027	M	N	2.2	0.86	0.93	1.4	2.2

Trial#	Strain	Sex	Inf?	Stance/Swing_RH	StanceFactor_LF	StanceFactor_LH	StanceWidth_LF	StanceWidth_LH
1	CC027	M	N	2.3	0.76	1.26	2.5	2.4
2	CC027	M	N	2.6	0.78	0.74	1.6	1.6
0	CC015	F	N	1.15	0.88	1.5	2.3	21.15
1	CC015	F	N	1.8	1.03	1.18	1.7	2.4
0	CC017	F	N	1.6	0.86	1.07	1	2
0	CC023	F	N	2.9	1	0.96	1.9	2.7
1	CC023	F	N	2	1.15	1.04	1.5	2.4
0	CC005	M	N	1	1.07	1.28	1.2	1.9
1	CC005	M	N	0.8	0.9	1.72	1.4	2.7
0	CC015	M	N	3	0.99	0.99	1.3	1.9
1	CC015	M	N	1.7	1.48	3.05	1.7	2.4
0	CC017	M	N	3.3	1.18	0.98	1.9	1.8
1	CC017	M	N	2.7	1.11	0.81	2	2.1
0	CC023	M	N	2.5	0.91	0.99	1.6	2.4
1	CC023	M	N	3.2	0.77	0.9	1.1	2.2
0	CC051	M	N	2.9	1.02	1.18	1.5	2.3
0	CC002	F	Y	2.6	0.88	1.32	1.1	1.6
0	CC002	M	Y	2.2	0.92	1.58	1.9	2
1	CC002	M	Y	0.2	1.01	1.9	1.4	1.2
1	CC002	F	Y	1.2	0.74	1.43	0.7	1.5
1	CC002	F	Y	2.3	0.78	1.2	1.3	1.9
1	CC002	M	Y	0.2	1.01	1.9	1.4	1.2
1	CC002	M	Y	2.4	1.07	1.37	1.8	2
1	CC002	F	Y	1.2	0.74	1.43	0.7	1.5
1	CC002	F	Y	1	1.41	0.94	1.1	1.2
2	CC002	M	Y	2.6	1.38	1.69	1.3	2
2	CC002	F	Y	2.9	0.94	0.92	1.7	2
2	CC002	M	Y	2.7	1.31	1.7	1.9	2.1
0	CC005	F	Y	2.8	1	1.04	1.2	1.8
0	CC005	M	Y	3.1	0.87	0.96	1.1	2.4
0	CC005	M	Y	3.3	1.09	0.99	1.2	2.4
1	CC005	F	Y	2.1	0.83	1.24	1.4	1.8
1	CC005	M	Y	0.3	1.7	3.87	1.1	1.8
1	CC005	F	Y	0.9	0.96	1.39	1.5	3.3
1	CC005	M	Y	2.8	0.67	0.67	1.2	2.3
2	CC005	F	Y	4.2	0.84	0.9	1	1.9
2	CC005	M	Y	2.6	0.7	1.15	1	3.4
2	CC005	M	Y	0.8	1.39	2.58	1.2	1.9
2	CC005	M	Y	2.6	0.7	1.15	1	3.4
2	CC005	F	Y	0.9	0.76	2.46	1.1	2.5
0	CC006	F	Y	2.8	1.03	0.98	1.3	1.8
0	CC006	M	Y	0.4	1.85	3.22	1.2	1.2
0	CC006	F	Y	3.4	1.12	0.96	1.7	2
1	CC006	F	Y	3.3	1.09	0.87	1.2	1.8
1	CC006	M	Y	3.1	1.06	0.81	1.4	1.3
1	CC006	F	Y	2.3	1.09	1.02	1.2	1.6
1	CC006	M	Y	2.8	0.84	0.9	1.5	2.4
2	CC006	F	Y	2.5	0.84	0.88	1.2	1.8

Trial#	Strain	Sex	Inf?	Stance/Swing_RH	StanceFactor_LF	StanceFactor_LH	StanceWidth_LF	StanceWidth_LH
2	CC006	M	Y	1.6	0.65	0.86	2.2	2.1
0	CC011	F	Y	3.9	1.05	0.75	1.3	1.3
0	CC011	M	Y	2.8	1.2	1.08	1.2	1.6
0	CC011	F	Y	1.9	0.85	1	1.4	1.7
0	CC011	F	Y	0.7	1.28	0.67	1.5	1.4
1	CC011	F	Y	2.5	0.99	1.06	1.6	1.8
1	CC011	M	Y	2.8	0.86	1.12	1.7	1.8
1	CC011	F	Y	1.6	0.98	1.16	2	2.2
1	CC011	M	Y	2.6	0.88	0.92	2.2	2.6
2	CC011	F	Y	2.5	1.06	0.99	1.5	1.6
2	CC011	M	Y	4.2	1.41	0.74	0.9	1.9
2	CC011	F	Y	2.8	1.01	1.04	1.2	1.7
2	CC011	M	Y	2	0.94	1.09	2.3	2.8
0	CC012	M	Y	2	0.98	1.19	1.2	1.9
0	CC012	M	Y	2	1.65	0.95	2.1	2.2
0	CC012	M	Y	2.2	1.05	1	1.2	2.1
0	CC012	M	Y	2.5	1.05	0.97	1.4	1.8
0	CC012	M	Y	2.5	1.19	1.2	1.1	2.1
0	CC012	F	Y	3.3	1.05	0.85	1.4	1.9
0	CC012	F	Y	2.5	1.04	1.06	1.3	2.3
0	CC012	F	Y	2.5	0.68	0.78	1.5	2.2
0	CC012	F	Y	1.4	1.13	0.96	1.3	2.5
0	CC012	F	Y	1.3	0.88	1.2	1.3	1.4
2	CC012	M	Y	2.3	1.2	1.11	1.3	2
2	CC012	M	Y	1.9	1.06	0.97	1.5	2
2	CC012	M	Y	2	1	0.98	1.7	2.5
2	CC012	M	Y	3.4	1.03	0.94	1.4	2.4
2	CC012	M	Y	2	1.02	1.05	1.5	2.3
2	CC012	F	Y	3.2	0.84	1.06	1.4	2.2
2	CC012	F	Y	1.7	0.77	1.01	1.4	2.6
2	CC012	F	Y	3.3	1.06	1.29	1.2	2.9
2	CC012	F	Y	2	1.21	1.19	1.2	2.3
2	CC012	F	Y	2.8	0.89	1	1.4	3.1
1	CC012XCC032	F	Y	1.6	1.05	1.4	1.9	1.2
1	CC012xCC032	F	Y	1.1	0.82	1.29	1	1.9
1	CC012XCC032	M	Y	2.4	0.98	1	1	2.6
1	CC012xCC032	M	Y	2.3	0.87	0.89	1.2	2.1
1	CC012XCC032	M	Y	1.3	0.69	1.67	2.1	2.3
1	CC012xCC032	M	Y	2.7	1.23	1.29	1.2	2.1
2	CC012XCC032	F	Y	2.6	1.4	0.82	1.3	2.7
2	CC012XCC032	F	Y	2.4	0.82	0.84	1.8	3.1
2	CC012XCC032	M	Y	2.5	1.42	1.49	1.1	2.5
2	CC012XCC032	M	Y	1.7	0.69	0.93	1.2	2.3
2	CC012XCC032	M	Y	3.1	1.09	1.01	1.4	2.7
2	CC012XCC032	M	Y	3.6	1.05	0.62	2	2.3
1	CC013xCC041	F	Y	2.4	0.7	1.17	1.5	2.2
1	CC013xCC041	F	Y	1.6	0.76	0.93	1.1	1.6
1	CC013xCC041	F	Y	3.8	0.98	0.94	1	2.1

Trial#	Strain	Sex	Inf?	Stance/Swing_RH	StanceFactor_LF	StanceFactor_LH	StanceWidth_LF	StanceWidth_LH
1	CC013xCC041	M	Y	2.8	0.92	0.95	1.9	2.7
1	CC013xCC041	M	Y	2.6	1.92	2.25	1.2	2.8
2	CC013XCC041	F	Y	2.9	1.03	0.97	2	2.9
2	CC013XCC041	F	Y	4.7	0.87	0.61	2.1	3.3
2	CC013XCC041	F	Y	3.3	0.8	0.9	1.7	2.5
2	CC013XCC041	M	Y	3.4	0.79	1.23	1.6	2.6
2	CC013XCC041	M	Y	1.9	1.02	1.73	1.7	1.4
0	CC015	F	Y	2.7	1.01	0.82	1.2	1.7
0	CC015	M	Y	2.9	0.97	1.02	1.7	2.2
0	CC015	M	Y	2.9	1.06	1.14	1.5	2
1	CC015	F	Y	1.6	0.98	1.22	1.3	3
1	CC015	M	Y	1.6	0.84	1.03	1.8	2.6
1	CC015	M	Y	2.6	0.94	0.82	1.5	2.2
1	CC015	M	Y	3.4	1.15	1.22	1.8	2.5
2	CC015	F	Y	2.5	0.87	1.02	1.8	2.6
0	CC017	F	Y	1.7	1.12	1.22	1.9	2
0	CC017	M	Y	3.3	1.17	0.64	1.1	1.4
0	CC017	F	Y	3.8	1.03	1.1	2.1	1.8
0	CC017	M	Y	2	1.58	1.31	2.1	2
1	CC017	M	Y	3	1.01	0.89	1.5	2.6
1	CC017	F	Y	2.5	0.65	0.92	1.7	1.9
0	CC023	M	Y	1.7	0.89	1.08	0.9	2
0	CC023	F	Y	1.5	0.89	2.64	1.3	1.4
0	CC023	F	Y	1.3	1.29	1.04	1.3	1.4
0	CC023	M	Y	1.6	0.47	1.33	1.3	2.5
1	CC023	M	Y	5.9	1.04	0.73	0.9	1.6
1	CC023	F	Y	6.3	1.21	0.62	0.7	1.8
1	CC023	M	Y	2.1	1.33	1.69	1	3
1	CC023	M	Y	2.2	1.1	0.89	1.1	2.1
2	CC023	M	Y	2	1.11	0.66	1.2	2.2
2	CC023	F	Y	3.4	1.41	0.9	1.7	2.7
2	CC023	M	Y	3.1	0.71	1.29	1.2	2.2
0	CC025	M	Y	2.1	0.91	1.16	1.6	2.2
0	CC025	F	Y	2.5	0.75	1.11	0.9	1.8
0	CC025	M	Y	1.5	1.23	1.09	1.7	2.3
1	CC025	M	Y	2.1	0.91	1.25	1.7	2.2
1	CC025	F	Y	1.9	0.96	0.94	1.3	2.1
1	CC025	F	Y	2.2	1.07	1.07	1.3	1.9
1	CC025	F	Y	1.7	1.12	1.34	1.3	2.1
2	CC025	F	Y	1.8	0.9	1.16	2.8	1.7
2	CC025	M	Y	2.5	0.93	1.08	1.2	2.5
0	CC027	F	Y	1.5	1.33	1.17	2.5	1.6
0	CC027	M	Y	1.9	1.22	0.78	1	3
1	CC027	M	Y	3.1	1.07	1.27	1.8	3.4
1	CC027	M	Y	3.1	1.07	1.27	1.8	3.4
1	CC027	F	Y	1.9	1	1.27	1.4	2.2
1	CC027	F	Y	1	1.35	2.33	1.5	2
1	CC027	M	Y	4.6	0.66	0.96	2.5	2.4

Trial#	Strain	Sex	Inf?	Stance/Swing_RH	StanceFactor_LF	StanceFactor_LH	StanceWidth_LF	StanceWidth_LH
1	CC027	M	Y	2.8	1.03	1.06	1.8	2.4
2	CC027	F	Y	3.4	1.41	0.9	1.7	2.7
2	CC027	M	Y	2	0.7	0.61	1.3	2.5
2	CC027	F	Y	1.5	1.36	1.35	1.7	2.1
2	CC027	M	Y	3.3	0.79	0.98	1.7	2.4
1	CC032XCC013	M	Y	2.8	1.22	1.07	1.7	2.8
1	CC032XCC013	M	Y	2.2	0.97	1.17	1.9	2.7
1	CC032XCC013	M	Y	1.2	0.73	1.05	1.4	2.2
1	CC032XCC013	F	Y	2.5	0.54	0.89	1.2	2.7
1	CC032XCC013	F	Y	1.8	1.04	1.05	1.9	2.7
1	CC032XCC013	F	Y	1.9	0.82	1.14	1.5	2.7
1	CC032XCC013	F	Y	1.9	1.33	1.31	1.3	2.9
1	CC032XCC013	F	Y	2.8	1.01	0.82	2	3.2
2	CC032XCC013	M	Y	3.3	1.14	0.97	1.6	2.8
2	CC032XCC013	M	Y	3.1	1.63	1.23	1.6	2.4
2	CC032XCC013	M	Y	2	0.64	1.38	0.9	2.4
2	CC032XCC013	M	Y	2.9	2.25	1.24	1.8	2.8
2	CC032XCC013	F	Y	2.5	1.09	1.13	1.7	2.6
2	CC032XCC013	F	Y	2.7	0.8	0.99	1.7	3.2
2	CC032XCC013	F	Y	2.1	1.68	0.97	1.6	2.8
2	CC032XCC013	F	Y	2.2	1.07	1.1	1.5	2.4
0	CC037	F	Y	3.3	0.93	0.78	1.5	2.6
0	CC037	M	Y	3.9	0.88	0.65	1.4	2.1
0	CC037	M	Y	0.9	0.73	1.48	1.3	1.9
0	CC037	M	Y	0.6	1.26	1.81	0.7	1.7
1	CC037	F	Y	2.8	0.85	1.06	1.6	2.2
1	CC037	M	Y	2	0.95	0.96	1.5	2.5
1	CC037	M	Y	2.1	1.06	1.06	2	2.6
1	CC037	M	Y	2.4	1.15	1.34	1.3	2.4
2	CC037	M	Y	2.6	1.21	1.1	1.9	3
2	CC037	M	Y	1.3	1.05	1.27	1.8	2.3
1	CC041XCC012	M	Y	3.4	1.44	0.74	1.3	1.8
1	CC041XCC012	M	Y	3.3	0.79	1.04	0.9	2.5
1	CC041XCC012	M	Y	2.7	0.92	1.04	1.5	2.2
1	CC041XCC012	M	Y	2.2	0.79	1.13	1.5	2
1	CC041XCC012	M	Y	3.2	1.15	0.97	1.4	2.2
1	CC041XCC012	F	Y	2.7	1.01	1.22	1.3	2.5
1	CC041XCC012	F	Y	2.8	1.63	1.13	1	2
1	CC041XCC012	F	Y	3.7	1.2	1.09	1.3	1.9
1	CC041XCC012	F	Y	2.6	1.2	1.1	1.4	2.5
1	CC041XCC012	F	Y	2.4	1.06	1.16	1.4	2.5
1	CC041XCC012	F	Y	2	1.26	1.13	1.2	2.5
1	CC041XCC012	F	Y	2.7	1.24	0.92	1.4	2.1
1	CC041XCC012	F	Y	3.5	1.08	1.19	1.5	2.2
2	CC041XCC012	M	Y	2.6	0.98	1.09	1.7	2.1
2	CC041XCC012	M	Y	3.1	1.25	0.94	1.3	2.5
2	CC041XCC012	F	Y	1.6	1.13	1.12	1.3	2.3
2	CC041XCC012	M	Y	3.7	0.86	0.54	1.3	2

Trial#	Strain	Sex	Inf?	Stance/Swing_RH	StanceFactor_LF	StanceFactor_LH	StanceWidth_LF	StanceWidth_LH
2	CC041XCC012	M	Y	3.3	0.93	0.92	1.6	2.3
2	CC041XCC012	M	Y	2.6	0.92	0.6	1	2.1
2	CC041XCC012	F	Y	3.3	0.94	0.93	1.6	2.6
2	CC041XCC012	F	Y	3	0.89	1.06	1.7	3.2
2	CC041XCC012	F	Y	2.5	1.07	1.15	1.6	2.4
2	CC041XCC012	F	Y	2.2	0.98	0.98	1.6	2.4
2	CC041XCC012	F	Y	3.6	0.96	1	1.4	2.7
2	CC041XCC012	F	Y	4	0.93	1.02	1.8	2.8
2	CC041XCC012	F	Y	2.8	1.04	1	1.6	2.2
0	CC051	F	Y	3	1.02	1.02	1.1	2.5
0	CC051	F	Y	3.6	1.29	1.06	1.4	2.9
0	CC051	F	Y	2.8	0.9	0.94	1.8	2.2
0	CC051	M	Y	3	1.03	1.04	1.8	2.3
1	CC051	F	Y	3.3	0.85	0.99	2	2.4
1	CC051	F	Y	3	1.29	1.01	2	2.8
1	CC051	F	Y	3.4	0.89	1.21	2.1	2.7
0	CC057	F	Y	2.9	0.96	1.01	2	2.8
0	CC057	F	Y	2.2	0.77	1.03	2.2	3.1
0	CC057	F	Y	2.7	0.98	1.28	1.8	3
0	CC057	M	Y	3.7	0.54	1.02	2.3	3
0	CC057	M	Y	1.8	1.23	1.02	1.2	2.8
0	CC057	M	Y	3.2	1.49	1.22	1.8	3.2
1	CC057	F	Y	3.1	1.04	0.85	1.7	2.9
1	CC057	F	Y	2	0.83	1.18	2.6	3.1
1	CC057	F	Y	1.6	1.1	1.3	2.3	2.6
1	CC057	F	Y	2.4	1.16	0.95	2.3	3.1
1	CC057	M	Y	2.9	1.12	0.79	1.9	3.5
1	CC057	M	Y	2.8	1.01	0.98	2.2	3
2	CC057	F	Y	2.4	1.16	0.95	2.3	3.1
0	CC078	F	Y	2.2	0.94	1.2	1.2	1.7
0	CC078	F	Y	2.1	1.03	0.92	1.2	1.8
0	CC078	F	Y	2.1	1.04	1	1.2	1.6
0	CC078	M	Y	2.3	1.02	0.87	1.3	1.8
1	CC078	F	Y	2.2	0.77	1.08	1	2
1	CC078	F	Y	1.3	1.07	1.46	0.9	1.4
1	CC078	M	Y	2.2	0.97	0.84	1.3	1.6
2	CC078	F	Y	2.1	1.21	1.25	1.4	2
2	CC078	M	Y	2.5	0.83	0.94	1.8	2
2	CC005	M	N	2.5	0.49	1.31	0.8	1.9
2	CC005	M	Y	1.8	0.95	0.86	1.2	1.9
2	CC015	F	N	2.4	1.3	0.89	1.3	2.4
2	CC015	M	N	2.5	0.88	1.06	1.7	2.3
2	CC015	M	Y	3	1.13	1.17	2	2.8
2	CC015	M	Y	3.1	1.16	1.21	1.8	1.7
2	CC017	F	N	2.6	1.7	1.1	1.6	2.8
2	CC017	M	N	1.7	1.14	0.91	1.7	2.5
2	CC017	F	Y	2.1	0.76	0.91	1.7	2.6
2	CC023	F	N	1.5	0.82	1.1	1.3	2.4

Trial#	Strain	Sex	Inf?	Stance/Swing_RH	StanceFactor_LF	StanceFactor_LH	StanceWidth_LF	StanceWidth_LH

Table S3. Raw DigiGait measurement data. Far left column indicates time point at which data was measured: T0 = pre-infection, T1 = 21dpi, and T3 = 89dpi. DigiGait parameters listed across the top indicate which limb is associated with the data, where appropriate: FL for left fore limb, FR for right fore limb, HL for left hind limb, and HR for right hind limb.

Table S3

Trial#	Strain	Sex	Inf?	StanceWidthCV_LF	StanceWidthCV_LH	StepAngle_LF	StepAngle_LH	StepAngleCV_LF
1	CC002	F	N	64.25	42.65	71.9	78.9	21.02
1	CC025	F	N	48.27	30.2	68.8	56.5	20.9
1	CC012XCC032	F	N	58.91	14.8	67.4	70.4	30.49
2	CC012XCC032	F	N	45.55	10.15	66.3	57.8	12.61
1	CC012XCC032	M	N	30.58	13.53	65.6	38.1	22.1
2	CC012XCC032	M	N	24.84	17.09	73.4	49.5	4.64
1	CC013xCC041	F	N	37.3	22.98	75.7	76.7	17.37
2	CC013XCC041	F	N	19.93	6.38	57.6	42.2	13.53
1	CC013xCC041	M	N	88.92	33.37	61.6	42.5	31.87
2	CC013XCC041	M	N	22.53	21.99	58.5	37.2	18.49
1	CC032XCC013	F	N	72.62	49.14	76.4	56.5	15.84
2	CC032XCC013	F	N	39.15	23.73	60.1	63	36.28
1	CC041XCC012	F	N	33.47	27.09	78	61.3	14.39
1	CC041XCC012	F	N	62.71	36.94	64.9	65.9	29.29
2	CC041XCC012	F	N	62.2	14.84	66.7	72.5	23.16
1	CC032XCC013	M	N	15.2	40.56	55.7	61.6	33.66
2	CC032XCC013	M	N	70.14	18.48	47.4	72.7	49.09
1	CC041XCC012	M	N	53.47	12.45	76.4	62.4	17.2
2	CC041XCC012	M	N	51.05	11.8	69.9	55.1	19.39
0	CC012	F	N	42.11	16.67	58.7	42.8	33.62
2	CC012	F	N	42.16	7.58	79.6	74.9	10.46
0	CC012	M	N	7.78	19.27	58.3	66.8	30.2
2	CC012	M	N	78.5	11.48	71.7	47.3	25.11
0	CC057	F	N	16.79	10.47	55.5	66.6	30.45
1	CC057	F	N	44.52	6.49	47.1	34.6	40.05
0	CC057	M	N	20.01	15.84	53.6	36.4	44.31
1	CC057	M	N	21.42	4.72	65.5	51.8	18.59
0	CC078	F	N	16.95	4.85	53.8	55.7	11.97
1	CC078	F	N	24.15	6.44	52.7	43.7	30.47
2	CC078	F	N	47.03	35.07	62.5	65.6	32.78
0	CC078	M	N	21.34	11.42	61	54	23.35
2	CC078	M	N	23.28	12.83	63.3	48.5	25.81
0	CC002	F	N	60.36	63.24	72.7	69.7	22.01
1	CC002	F	N	64.25	42.65	71.9	78.9	21.02
0	CC002	M	N	44.83	38.78	63.9	77.1	32.05
1	CC002	M	N	37.34	22.17	55.8	50.6	36.74
2	CC002	M	N	10.58	11.21	68.3	73	25.26
0	CC006	F	N	9.65	7.5	40.9	64.4	41.09
1	CC006	F	N	40.82	18.04	68.3	46.7	28.68
2	CC006	F	N	48.72	36.19	64.8	58.9	35.03
0	CC006	M	N	43.18	42.12	62.2	68.2	32.68
0	CC023	F	N	43.8	48.87	52	68.2	45.74
1	CC023	F	N	49.47	37.71	77.3	65.2	17.24
2	CC023	F	N	36.92	46.85	65	45.9	18.74
0	CC023	M	N	12.11	32.4	67.8	46	16.92
1	CC023	M	N	48.46	13.25	61.9	51.7	30.16
0	CC027	F	N	67.76	84.84	72.9	58.3	21.83

Trial#	Strain	Sex	Inf?	StanceWidthCV_LF	StanceWidthCV_LH	StepAngle_LF	StepAngle_LH	StepAngleCV_LF
1	CC027	F	N	75.52	33.58	64.4	63.2	36.72
2	CC027	F	N	55.54	26.96	60.1	66.5	35.41
0	CC027	M	N	57.73	52.04	48.5	59.4	43.15
1	CC027	M	N	52.15	34.96	53.7	45.3	41.18
1	CC027	M	N	50.22	63.52	74.2	61.4	14.35
2	CC027	M	N	28.44	31.67	57.7	43.9	29.78
0	CC005	F	N	59.18	11.62	58.2	56.8	23.44
1	CC005	F	N	66.56	35.55	60.6	54.5	38.05
2	CC005	F	N	39.21	57.83	59.1	49.8	30.15
0	CC011	F	N	26.91	16.52	50.8	50.6	35.29
1	CC011	F	N	56.45	22.88	82.9	81	10.94
2	CC011	F	N	46.63	17.53	68.8	66	23.58
1	CC017	F	N	75.24	46.91	78.2	54.4	16.63
0	CC005	M	N	38.63	9.15	49.9	45.7	39.12
1	CC005	M	N	55.52	35.8	73.9	69.4	13.47
2	CC005	M	N	76.51	37.27	77.4	64.1	18.85
0	CC011	M	N	8.62	5.04	48.2	47.9	35.11
1	CC011	M	N	25.94	12.04	44.9	57.1	34.88
2	CC011	M	N	32.84	31.51	83.6	73.8	8.09
0	CC017	M	N	44.59	45.98	76.6	57	12.53
1	CC017	M	N	74.74	40.99	56	60.1	45.87
1	CC006	F	N	71.28	72.47	76.6	67	19.18
0	CC037	F	N	62.1	28.42	69.2	69.2	29.92
0	CC051	F	N	49.59	9.72	71.1	70.9	26.55
1	CC051	F	N	56.56	16.76	54.4	51.1	55.21
1	CC006	M	N	56.54	28.99	72.9	58.8	18.22
0	CC037	M	N	59.01	23.18	59	58	43.46
1	CC037	M	N	41.85	34.24	55	47.9	29.58
0	CC005	F	N	28.6	25.2	53.4	46	33.98
1	CC005	F	N	32.06	6.79	46.2	40.3	46.16
2	CC005	F	N	35.8	17.93	81.5	63.3	10.13
0	CC011	F	N	22.47	29.93	56.8	59.4	21.86
1	CC011	F	N	8.45	8.42	56.2	56.8	27.53
2	CC011	F	N	19.53	5.62	64.6	60.5	21.41
0	CC011	M	N	33.74	16.24	39.4	49	50.44
1	CC011	M	N	34.15	10.2	41.6	34.3	49.62
2	CC011	M	N	45.81	25.4	41.9	42.4	54.34
1	CC037	M	N	58.7	13.39	79.1	55.9	10.68
2	CC037	M	N	40.89	19.39	63.6	39.5	33.48
1	CC051	M	N	17.31	11.95	55.2	31.5	25.94
2	CC051	M	N	52.14	26.73	60.4	43	26.43
0	CC027	F	N	27.9	27.23	64.8	56.8	18.77
1	CC027	F	N	18.73	15.84	62.8	53.5	20.94
2	CC027	F	N	54.49	31.69	65.9	64	32.62
0	CC015	M	N	53.26	35.44	44.2	65.8	54.57
1	CC015	M	N	40.2	41.61	69.8	70.9	17.05
2	CC015	M	N	21.77	14.09	68.9	43.3	19.45
0	CC027	M	N	74.32	47.24	65.6	64.9	28.33

Trial#	Strain	Sex	Inf?	StanceWidthCV_LF	StanceWidthCV_LH	StepAngle_LF	StepAngle_LH	StepAngleCV_LF
1	CC027	M	N	21.29	32.74	52.4	76.3	45.63
2	CC027	M	N	33.45	36.12	64.6	67.8	26.16
0	CC015	F	N	6.26	47.5	55.9	39.82	26.77
1	CC015	F	N	57.99	35.29	67.5	64.9	29.16
0	CC017	F	N	44.25	11.15	67.9	51.7	23.28
0	CC023	F	N	38.36	13.26	49.5	48.3	45.32
1	CC023	F	N	23.19	8.85	67	36	17.92
0	CC005	M	N	82.83	64	71.2	68.6	23.43
1	CC005	M	N	32.2	6.13	56	52	28.42
0	CC015	M	N	26.57	8.81	67.4	59.7	20.34
1	CC015	M	N	45.97	10.09	77.4	69.5	8.3
0	CC017	M	N	47.11	43.42	61.7	53.6	29.18
1	CC017	M	N	50.65	34.14	60	56.4	36.16
0	CC023	M	N	19.05	4.67	45.1	47.3	45.4
1	CC023	M	N	63.17	16.93	76.3	56.5	22.1
0	CC051	M	N	25.04	15.59	64.6	56.9	19.41
0	CC002	F	Y	60.3	33.12	73.2	59.1	22.13
0	CC002	M	Y	39.61	26.11	47.9	73	38.91
1	CC002	M	Y	23.33	50.67	70.4	84.3	19.11
1	CC002	F	Y	77.23	44.29	64.6	51.5	33.53
1	CC002	F	Y	75.63	46.39	64.5	72	32.93
1	CC002	M	Y	23.33	50.67	70.4	84.3	19.11
1	CC002	M	Y	54.21	48.71	52.3	52.6	46.52
1	CC002	F	Y	77.23	44.29	64.6	51.5	33.53
1	CC002	F	Y	64.76	48.46	81.3	66.2	14.29
2	CC002	M	Y	36.9	13.23	74.1	48.8	11.93
2	CC002	F	Y	45.4	43.36	69.9	64.7	16.24
2	CC002	M	Y	41.96	31.7	70.7	51.5	25.07
0	CC005	F	Y	53.13	22.61	52.1	42.6	32.92
0	CC005	M	Y	56.93	16.48	60	53.1	35.53
0	CC005	M	Y	50.34	12.24	61.5	54.6	30.26
1	CC005	F	Y	64.25	24.34	73.7	62.9	13.37
1	CC005	M	Y	54.75	27.21	77.5	69.2	13.21
1	CC005	F	Y	22.13	6.7	72	51.9	14.02
1	CC005	M	Y	45.19	42.04	76.8	70	12.33
2	CC005	F	Y	46.88	25.52	65.6	53.4	29
2	CC005	M	Y	76.24	22.26	73.4	48.2	34.73
2	CC005	M	Y	57.74	34.85	75.4	66.9	10.97
2	CC005	M	Y	76.24	22.26	73.4	48.2	34.73
2	CC005	F	Y	72.48	51.51	69	73.6	22.82
0	CC006	F	Y	48.98	13.49	62.6	59.8	27.92
0	CC006	M	Y	47.98	52.27	81.3	71.7	12.23
0	CC006	F	Y	66	63.48	71.2	62.4	25.83
1	CC006	F	Y	58.82	33.67	67.1	56.8	30.87
1	CC006	M	Y	59.09	47.79	55.2	65.2	41.92
1	CC006	F	Y	55	20.12	61.1	68.2	20.36
1	CC006	M	Y	50.1	14.62	65.9	62.8	20.94
2	CC006	F	Y	45.28	36.59	57.6	54.4	31.64

Trial#	Strain	Sex	Inf?	StanceWidthCV_LF	StanceWidthCV_LH	StepAngle_LF	StepAngle_LH	StepAngleCV_LF
2	CC006	M	Y	32.87	33.51	70.8	58.9	19.71
0	CC011	F	Y	96.82	10.96	70.6	70.5	19.79
0	CC011	M	Y	39.94	14.52	70.8	61.7	18.31
0	CC011	F	Y	13.91	7.39	44.4	55.6	21.69
0	CC011	F	Y	17.91	25.86	50.4	65.6	28.93
1	CC011	F	Y	12.24	8.67	60	56.2	20.14
1	CC011	M	Y	15.9	11.38	35.6	56.1	40.28
1	CC011	F	Y	16.29	10.99	35	40.3	54.05
1	CC011	M	Y	20.07	7.49	70.3	37.4	19.28
2	CC011	F	Y	37.61	7.92	58.6	61.5	16.63
2	CC011	M	Y	66.68	10.84	70.3	56.9	18.38
2	CC011	F	Y	18.83	9.12	59.5	50.8	18.92
2	CC011	M	Y	13.83	18.3	41	47.4	40.88
0	CC012	M	Y	52.48	23.13	72.4	58.5	19.02
0	CC012	M	Y	23.49	44.86	63.4	42.2	30.6
0	CC012	M	Y	65.63	16.23	59.1	56.7	22.53
0	CC012	M	Y	33.4	35.38	81	84	8.98
0	CC012	M	Y	34.51	16.64	65.2	60.8	25.47
0	CC012	F	Y	35.15	23.05	56.9	56.7	39.72
0	CC012	F	Y	44.71	9.67	59.6	57.7	28.8
0	CC012	F	Y	36.98	29.15	85	50.4	7.33
0	CC012	F	Y	45.09	21.12	71.1	53	24.95
0	CC012	F	Y	46.32	52.88	48.4	39.2	35.93
2	CC012	M	Y	24.76	10.91	73.5	63.9	12.93
2	CC012	M	Y	23.84	17.17	72.2	50	14.8
2	CC012	M	Y	33.71	9.79	70.6	44.1	16.69
2	CC012	M	Y	57.84	10.67	60.2	57.5	35.8
2	CC012	M	Y	42.03	23.03	54.4	42	37.09
2	CC012	F	Y	39.71	16.61	59.2	51.8	29.18
2	CC012	F	Y	18.05	3.86	63.2	38.8	21.38
2	CC012	F	Y	62.25	9.43	66.1	50.1	29.42
2	CC012	F	Y	33.38	8.2	84	71.7	2.98
2	CC012	F	Y	35.47	11.71	51.2	49.7	36.56
1	CC012XCC032	F	Y	55.59	73.5	57.3	82.5	42.34
1	CC012xCC032	F	Y	65.93	43.98	75.7	62.1	23.33
1	CC012XCC032	M	Y	55.27	14.5	71.7	55.6	26.34
1	CC012xCC032	M	Y	44.96	20.68	65	43.9	39.15
1	CC012XCC032	M	Y	37.85	42.26	52	50.2	44.34
1	CC012xCC032	M	Y	63.14	47.14	69.2	58.7	28.58
2	CC012XCC032	F	Y	67.4	4.14	63	53.8	28.03
2	CC012XCC032	F	Y	23.71	5.73	50	47.3	33.41
2	CC012XCC032	M	Y	61.77	15.19	72.2	71.7	16.04
2	CC012XCC032	M	Y	60.91	7.11	63.8	61.7	36.25
2	CC012XCC032	M	Y	28.16	11.98	70.1	48.1	16.42
2	CC012XCC032	M	Y	41.33	8.44	56.1	54.4	30.08
1	CC013xCC041	F	Y	45.48	26.26	69.2	70.3	29.39
1	CC013xCC041	F	Y	66.05	50.56	79.2	59	19.37
1	CC013xCC041	F	Y	63.69	22.56	68.9	39.7	36.42

Trial#	Strain	Sex	Inf?	StanceWidthCV_LF	StanceWidthCV_LH	StepAngle_LF	StepAngle_LH	StepAngleCV_LF
1	CC013xCC041	M	Y	34.15	17.09	46.4	31.3	46.15
1	CC013xCC041	M	Y	63.77	12.44	77	53	19.73
2	CC013XCC041	F	Y	10.38	5.81	48.8	49.2	18.78
2	CC013XCC041	F	Y	11.07	5.58	58.1	46.4	29.06
2	CC013XCC041	F	Y	10.53	7.85	59.3	43.3	18.9
2	CC013XCC041	M	Y	21.3	6.67	45.8	59.3	46.25
2	CC013XCC041	M	Y	47.82	82.47	52	71.3	45.83
0	CC015	F	Y	44.15	35.04	64.6	51.5	25.33
0	CC015	M	Y	19.28	12.92	64.7	53.3	18.93
0	CC015	M	Y	19.31	8.79	76.7	52.1	5.59
1	CC015	F	Y	77.96	43.2	72.1	39.3	22.01
1	CC015	M	Y	44.89	12.58	52.1	54.1	35.57
1	CC015	M	Y	54.86	31.71	63.1	48.5	37.5
1	CC015	M	Y	46.32	28.23	80.1	77.9	16.7
2	CC015	F	Y	34.52	5.86	60.6	50.1	34.39
0	CC017	F	Y	42.16	38.97	53	27.5	30.12
0	CC017	M	Y	57.47	52.04	56.2	52.3	36.85
0	CC017	F	Y	56.7	41.2	69.2	78.2	20.92
0	CC017	M	Y	49.29	39.1	69.7	62.4	18.74
1	CC017	M	Y	84.31	35.88	52.8	37.5	46.49
1	CC017	F	Y	53.93	38.71	68.1	47.6	20.56
0	CC023	M	Y	57.15	30.09	60.5	41	18.23
0	CC023	F	Y	54.88	70.46	74	79.5	22.73
0	CC023	F	Y	30.26	32.42	71.5	51.9	20.08
0	CC023	M	Y	87.8	43.19	61.4	40.4	38.84
1	CC023	M	Y	56.49	40.91	64	76.1	29.32
1	CC023	F	Y	98.03	25.82	72.9	75.5	24.01
1	CC023	M	Y	93.75	26.38	77.6	62.5	16.74
1	CC023	M	Y	54.49	7.03	54.4	40.7	55.16
2	CC023	M	Y	15.44	8.25	51.9	48.3	31.32
2	CC023	F	Y	47.99	15.37	74.1	48.5	17.3
2	CC023	M	Y	71.28	15.64	63.8	68.9	35.49
0	CC025	M	Y	35.27	21.53	61.9	65.4	31.95
0	CC025	F	Y	74.22	34.22	72.2	60.1	17.37
0	CC025	M	Y	26.07	4.47	55.4	41.9	48.88
1	CC025	M	Y	29.52	21.57	52.4	73.3	38.91
1	CC025	F	Y	50.62	39.94	53.7	62.4	38.81
1	CC025	F	Y	44.17	24.13	72.5	39.7	28.25
1	CC025	F	Y	56.4	28.22	65.2	70.7	15.93
2	CC025	F	Y	27.16	44.91	45	69.8	30.59
2	CC025	M	Y	51.07	43.05	61.9	43.6	35.31
0	CC027	F	Y	66.5	27.29	69	45.8	17.91
0	CC027	M	Y	62.87	43.4	78.9	59.7	11.03
1	CC027	M	Y	77.26	43.39	54.4	36.9	29.5
1	CC027	M	Y	77.26	43.39	54.4	36.9	29.5
1	CC027	F	Y	70	40.07	43.5	63.3	53.75
1	CC027	F	Y	46.09	19.88	72.3	81.9	18.51
1	CC027	M	Y	15.15	49.52	65.5	59.7	23.3

Trial#	Strain	Sex	Inf?	StanceWidthCV_LF	StanceWidthCV_LH	StepAngle_LF	StepAngle_LH	StepAngleCV_LF
1	CC027	M	Y	8.59	5.74	46.4	59.8	37.91
2	CC027	F	Y	47.99	15.37	74.1	48.5	17.3
2	CC027	M	Y	41.34	23.24	59.1	52.4	41.34
2	CC027	F	Y	57.45	51.93	63.2	76	36.44
2	CC027	M	Y	23.3	12.12	67.9	51.6	23.95
1	CC032XCC013	M	Y	51.49	12.82	71.8	55.2	25.31
1	CC032XCC013	M	Y	29.11	16.74	56	57.3	40.18
1	CC032XCC013	M	Y	58.33	21.02	45.3	40.7	49.06
1	CC032XCC013	F	Y	66.3	29.22	61.5	42	45.19
1	CC032XCC013	F	Y	34.56	7.05	50.8	30.1	42.01
1	CC032XCC013	F	Y	29.01	16.16	48.8	44.6	39.08
1	CC032XCC013	F	Y	26.58	12.1	49	41.6	37.7
1	CC032XCC013	F	Y	15.88	5.02	37.3	35.5	55.79
2	CC032XCC013	M	Y	5.92	5.24	53.4	40.1	41.11
2	CC032XCC013	M	Y	45.06	7.73	64.6	52	31.57
2	CC032XCC013	M	Y	109.23	37.36	76	55	26.71
2	CC032XCC013	M	Y	27.59	14.66	61.3	66.9	33.73
2	CC032XCC013	F	Y	11.75	6.53	79.2	54.6	4.24
2	CC032XCC013	F	Y	30.41	7.27	47.4	50.8	50.41
2	CC032XCC013	F	Y	41.25	6.84	58.2	45.5	40.69
2	CC032XCC013	F	Y	32.25	7.93	66.6	59.1	21.78
0	CC037	F	Y	42.65	10.8	64.1	71.5	33.81
0	CC037	M	Y	61.9	38.14	53.5	69.2	42.11
0	CC037	M	Y	41.57	5.33	54.7	48.1	50.23
0	CC037	M	Y	49.64	50.18	85.2	77.8	2.78
1	CC037	F	Y	28.67	20.34	56.9	58	28.88
1	CC037	M	Y	63.39	23.36	56.8	47.4	32.86
1	CC037	M	Y	41.04	13	46.1	54.9	51.05
1	CC037	M	Y	43.82	15.47	70.3	62.3	21.54
2	CC037	M	Y	25.22	6.74	43.8	39.1	45.84
2	CC037	M	Y	12.81	4.77	66.8	63.1	16.53
1	CC041XCC012	M	Y	50.2	24.34	83	60.7	6.97
1	CC041XCC012	M	Y	54.52	5.06	76.7	52.3	16.48
1	CC041XCC012	M	Y	10.79	18.8	56.3	71	33.65
1	CC041XCC012	M	Y	36.44	17.22	72.1	62.9	21.81
1	CC041XCC012	M	Y	17.18	11.96	71	32.1	19.45
1	CC041XCC012	F	Y	55.35	15.95	57.7	63.8	36.91
1	CC041XCC012	F	Y	47.99	10.46	62.8	76.4	35.22
1	CC041XCC012	F	Y	10.15	12.14	66.4	62.3	18.85
1	CC041XCC012	F	Y	20.01	16.08	48.5	30.4	46.75
1	CC041XCC012	F	Y	31.68	34.94	66.8	42.8	23.72
1	CC041XCC012	F	Y	55.82	6.89	63.1	55.3	37.28
1	CC041XCC012	F	Y	26.12	14.1	55.8	45.9	37.18
1	CC041XCC012	F	Y	15.86	37.13	63.8	57.9	19.61
2	CC041XCC012	M	Y	11.71	8.78	61.7	64.2	39.51
2	CC041XCC012	M	Y	12.06	8.24	75.4	63.3	8.53
2	CC041XCC012	F	Y	36.12	6.97	80.9	62.4	7.71
2	CC041XCC012	M	Y	47.73	35.48	63.3	37	27.33

Trial#	Strain	Sex	Inf?	StanceWidthCV_LF	StanceWidthCV_LH	StepAngle_LF	StepAngle_LH	StepAngleCV_LF
2	CC041XCC012	M	Y	25.33	11.08	63.6	58.9	22.14
2	CC041XCC012	M	Y	42.74	10.52	73.1	35.9	13.54
2	CC041XCC012	F	Y	20.91	8	63.4	43.5	10
2	CC041XCC012	F	Y	18.69	19.82	64.7	52.8	24.27
2	CC041XCC012	F	Y	20.09	9.24	79.9	59.6	7.8
2	CC041XCC012	F	Y	27.59	4.61	52.5	43.9	22.47
2	CC041XCC012	F	Y	51.77	20.45	57.9	46.9	31.25
2	CC041XCC012	F	Y	13.6	10.1	68	53.4	17.2
2	CC041XCC012	F	Y	48.05	13.95	75	54	15.32
0	CC051	F	Y	76.93	38.85	68.6	64.1	27.46
0	CC051	F	Y	35.97	13.35	63.5	66.9	35.51
0	CC051	F	Y	28.93	15.87	63.5	48.1	24.97
0	CC051	M	Y	18.77	5.42	57	37.5	28.2
1	CC051	F	Y	45.02	28.24	65.1	56.3	16.48
1	CC051	F	Y	26.99	19.77	54.8	35.1	28.31
1	CC051	F	Y	66.9	20.28	60.3	51.4	33.47
0	CC057	F	Y	27.06	8.52	36.7	50.5	44.87
0	CC057	F	Y	34.61	9.34	36.8	43.4	52.83
0	CC057	F	Y	46.55	11.08	59.6	58.5	35.21
0	CC057	M	Y	29.01	22.37	49.1	44.9	46.5
0	CC057	M	Y	65.15	24.52	82.1	48.5	7.52
0	CC057	M	Y	53.73	8.06	56.9	34	43.78
1	CC057	F	Y	49.26	8.92	24.2	41.9	68.96
1	CC057	F	Y	22.61	16.04	49.7	51.8	33.25
1	CC057	F	Y	39.01	35.44	69.7	43.7	24.91
1	CC057	F	Y	47.99	16.65	51.9	39.2	49.32
1	CC057	M	Y	39.27	3.1	48.6	41.3	50.17
1	CC057	M	Y	20.31	13.62	48.5	32.2	43.06
2	CC057	F	Y	47.99	16.65	51.9	39.2	49.32
0	CC078	F	Y	15.47	10.72	67.7	72	17.37
0	CC078	F	Y	23.62	24.22	65.9	55.1	20.26
0	CC078	F	Y	24.43	6.41	69	44.5	15.1
0	CC078	M	Y	25.26	4.89	66.6	53.7	22.32
1	CC078	F	Y	15	7.71	65.9	44.5	21.93
1	CC078	F	Y	33.69	9.01	55.6	65.6	44.6
1	CC078	M	Y	28.65	11.81	47.3	49.3	36.04
2	CC078	F	Y	25.02	23.33	74.1	47.2	18.53
2	CC078	M	Y	27.47	26.11	48.4	56.7	45.3
2	CC005	M	N	71.35	53.12	84.8	66.6	6.51
2	CC005	M	Y	61.97	50.87	77	84.3	18.96
2	CC015	F	N	66.72	26.62	58.9	56.3	39.74
2	CC015	M	N	8.23	3.44	54.2	59.5	33.97
2	CC015	M	Y	11.86	9.19	51	67	35.21
2	CC015	M	Y	42.61	47.64	63.2	60.1	29.34
2	CC017	F	N	36.62	4.62	78.8	52.7	14.03
2	CC017	M	N	28.52	12.28	56.4	42	42.05
2	CC017	F	Y	45.8	20.35	72.5	52.4	26.27
2	CC023	F	N	28.81	10.04	65.7	56.4	21.16

Trial#	Strain	Sex	Inf?	StanceWidthCV_LF	StanceWidthCV_LH	StepAngle_LF	StepAngle_LH	StepAngleCV_LF

Table S3. Raw DigiGait measurement data. Far left column indicates time point at which data was measured: T0 = pre-infection, T1 = 21dpi, and T3 = 89dpi. DigiGait parameters listed across the top indicate which limb is associated with the data, where appropriate: FL for left fore limb, FR for right fore limb, HL for left hind limb, and HR for right hind limb.

Table S3

Trial#	Strain	Sex	Inf?	StepAngleCV_LH	StepAngleVar_LF	StepAngleVar_LH	Stride_LF	Stride_LH	Stride_RF
1	CC002	F	N	18.36	15.12	14.48	0.217	0.167	0.227
1	CC025	F	N	40.33	14.38	22.78	0.209	0.242	0.217
1	CC012XCC032	F	N	21.01	20.54	14.79	0.29	0.346	0.297
2	CC012XCC032	F	N	25.52	8.35	14.74	0.323	0.392	0.394
1	CC012XCC032	M	N	73.83	14.51	28.11	0.263	0.315	0.298
2	CC012XCC032	M	N	44.78	3.4	22.15	0.341	0.288	0.336
1	CC013xCC041	F	N	20.39	13.15	15.63	0.249	0.153	0.196
2	CC013XCC041	F	N	18	7.8	7.59	0.331	0.338	0.332
1	CC013xCC041	M	N	38.12	19.63	16.18	0.093	0.141	0.143
2	CC013XCC041	M	N	45.58	10.81	16.96	0.256	0.283	0.256
1	CC032XCC013	F	N	43.34	12.1	24.48	0.122	0.194	0.092
2	CC032XCC013	F	N	23.2	21.8	14.63	0.244	0.249	0.252
1	CC041XCC012	F	N	26.97	11.22	16.53	0.188	0.172	0.281
1	CC041XCC012	F	N	23.24	19.01	15.32	0.121	0.122	0.133
2	CC041XCC012	F	N	14.9	15.45	10.81	0.211	0.317	0.166
1	CC032XCC013	M	N	30.46	18.76	18.75	0.146	0.166	0.156
2	CC032XCC013	M	N	20.67	23.29	15.03	0.135	0.306	0.109
1	CC041XCC012	M	N	27.44	13.13	17.12	0.26	0.294	0.177
2	CC041XCC012	M	N	30.35	13.55	16.72	0.313	0.363	0.315
0	CC012	F	N	31.91	19.73	13.65	0.197	0.236	0.214
2	CC012	F	N	13.92	8.32	10.42	0.428	0.432	0.312
0	CC012	M	N	20.75	17.62	13.87	0.211	0.177	0.241
2	CC012	M	N	42.87	18	20.28	0.176	0.298	0.167
0	CC057	F	N	35.41	16.89	23.57	0.276	0.293	0.234
1	CC057	F	N	36.3	18.86	12.58	0.298	0.285	0.294
0	CC057	M	N	54.33	23.73	19.75	0.208	0.291	0.31
1	CC057	M	N	27.45	12.18	14.21	0.348	0.35	0.371
0	CC078	F	N	28.82	6.44	16.04	0.279	0.275	0.275
1	CC078	F	N	34.63	16.07	15.14	0.285	0.29	0.244
2	CC078	F	N	24.1	20.47	15.8	0.163	0.223	0.137
0	CC078	M	N	30.84	14.25	16.66	0.243	0.243	0.241
2	CC078	M	N	38.75	16.34	18.8	0.245	0.263	0.224
0	CC002	F	N	25.6	16.01	17.86	0.218	0.133	0.171
1	CC002	F	N	18.36	15.12	14.48	0.217	0.167	0.227
0	CC002	M	N	10.11	20.47	7.79	0.138	0.174	0.183
1	CC002	M	N	40.38	20.51	20.43	0.145	0.17	0.226
2	CC002	M	N	24.78	17.27	18.09	0.354	0.405	0.316
0	CC006	F	N	15.62	16.8	10.05	0.292	0.293	0.297
1	CC006	F	N	27.81	19.6	13	0.193	0.248	0.254
2	CC006	F	N	34.05	22.71	20.06	0.139	0.192	0.214
0	CC006	M	N	27.18	20.33	18.52	0.187	0.168	0.22
0	CC023	F	N	20.84	23.78	14.22	0.254	0.195	0.251
1	CC023	F	N	27.79	13.33	18.13	0.202	0.244	0.174
2	CC023	F	N	49.59	12.18	22.75	0.323	0.347	0.204
0	CC023	M	N	38.34	11.47	17.64	0.283	0.29	0.293
1	CC023	M	N	35.48	18.68	18.32	0.265	0.283	0.287
0	CC027	F	N	43.74	15.92	25.51	0.197	0.157	0.119

Trial#	Strain	Sex	Inf?	StepAngleCV_LH	StepAngleVar_LF	StepAngleVar_LH	Stride_LF	Stride_LH	Stride_RF
1	CC027	F	N	23.8	23.64	15.04	0.241	0.203	0.215
2	CC027	F	N	32.39	21.3	21.53	0.295	0.337	0.313
0	CC027	M	N	37.29	20.91	22.15	0.192	0.104	0.194
1	CC027	M	N	40.66	22.14	18.43	0.136	0.153	0.16
1	CC027	M	N	46.44	10.66	28.53	0.274	0.202	0.196
2	CC027	M	N	49.59	17.18	21.76	0.284	0.258	0.238
0	CC005	F	N	27.31	13.63	15.51	0.195	0.256	0.255
1	CC005	F	N	40.77	23.05	22.23	0.192	0.223	0.223
2	CC005	F	N	45.49	17.81	22.64	0.118	0.097	0.121
0	CC011	F	N	30.11	17.94	15.25	0.145	0.155	0.143
1	CC011	F	N	9.7	9.06	7.86	0.211	0.312	0.187
2	CC011	F	N	16.67	16.21	11	0.303	0.333	0.303
1	CC017	F	N	43.49	13	23.66	0.179	0.249	0.228
0	CC005	M	N	13.01	19.5	5.94	0.283	0.282	0.267
1	CC005	M	N	25.06	9.95	17.39	0.183	0.148	0.205
2	CC005	M	N	30.15	14.58	19.33	0.164	0.175	0.176
0	CC011	M	N	31	16.93	14.85	0.221	0.237	0.219
1	CC011	M	N	28.47	15.67	16.25	0.266	0.262	0.261
2	CC011	M	N	15.91	6.76	11.74	0.212	0.236	0.176
0	CC017	M	N	33.11	9.6	18.87	0.193	0.126	0.149
1	CC017	M	N	38.21	25.67	22.96	0.165	0.27	0.35
1	CC006	F	N	23.48	14.69	15.74	0.107	0.089	0.094
0	CC037	F	N	17.91	20.7	12.39	0.267	0.289	0.236
0	CC051	F	N	17.23	18.87	12.22	0.194	0.306	0.205
1	CC051	F	N	29.11	30.05	14.87	0.152	0.174	0.247
1	CC006	M	N	28.29	13.29	16.64	0.257	0.303	0.276
0	CC037	M	N	31.02	25.64	18.01	0.229	0.287	0.28
1	CC037	M	N	46.51	16.26	22.26	0.254	0.271	0.283
0	CC005	F	N	33.39	18.15	15.36	0.206	0.255	0.22
1	CC005	F	N	32.36	21.33	13.05	0.313	0.365	0.322
2	CC005	F	N	20.36	8.26	12.88	0.343	0.336	0.186
0	CC011	F	N	27.26	12.42	16.18	0.245	0.243	0.237
1	CC011	F	N	25.01	15.48	14.19	0.276	0.288	0.269
2	CC011	F	N	24.57	13.83	14.88	0.294	0.284	0.284
0	CC011	M	N	33.84	19.87	16.57	0.266	0.266	0.276
1	CC011	M	N	25.06	20.66	8.58	0.311	0.304	0.308
2	CC011	M	N	27.72	22.77	11.76	0.293	0.294	0.303
1	CC037	M	N	30.84	8.45	17.23	0.297	0.297	0.197
2	CC037	M	N	54.43	21.28	21.51	0.253	0.285	0.196
1	CC051	M	N	42.93	14.31	13.51	0.301	0.29	0.3
2	CC051	M	N	37	15.96	15.9	0.262	0.293	0.264
0	CC027	F	N	27.35	12.17	15.54	0.347	0.362	0.355
1	CC027	F	N	36.59	13.14	19.59	0.287	0.208	0.232
2	CC027	F	N	25.9	21.51	16.58	0.229	0.254	0.253
0	CC015	M	N	21.35	24.13	14.04	0.256	0.264	0.262
1	CC015	M	N	17.67	11.91	12.53	0.236	0.275	0.223
2	CC015	M	N	49.13	13.4	21.26	0.338	0.336	0.293
0	CC027	M	N	19.19	18.58	12.46	0.26	0.262	0.291

Trial#	Strain	Sex	Inf?	StepAngleCV_LH	StepAngleVar_LF	StepAngleVar_LH	Stride_LF	Stride_LH	Stride_RF
1	CC027	M	N	11.02	23.91	8.41	0.287	0.325	0.341
2	CC027	M	N	26.28	16.89	17.82	0.226	0.19	0.276
0	CC015	F	N	18.9	14.98	0.283	0.281	0.29	0.293
1	CC015	F	N	29.56	19.69	19.17	0.127	0.133	0.126
0	CC017	F	N	29.3	15.8	15.16	0.245	0.279	0.258
0	CC023	F	N	41.03	22.43	19.82	0.254	0.234	0.237
1	CC023	F	N	44.27	12	15.94	0.304	0.288	0.259
0	CC005	M	N	24.22	16.68	16.61	0.273	0.225	0.264
1	CC005	M	N	33.46	15.91	17.38	0.339	0.365	0.349
0	CC015	M	N	24.56	13.71	14.67	0.298	0.293	0.293
1	CC015	M	N	28.08	6.43	19.52	0.217	0.299	0.145
0	CC017	M	N	44.97	18	24.1	0.268	0.247	0.235
1	CC017	M	N	35.21	21.7	19.85	0.13	0.116	0.118
0	CC023	M	N	16.2	20.45	7.66	0.319	0.32	0.33
1	CC023	M	N	30.96	16.86	17.5	0.263	0.302	0.316
0	CC051	M	N	26.12	12.55	14.86	0.293	0.333	0.304
0	CC002	F	Y	27.14	16.2	16.04	0.145	0.157	0.144
0	CC002	M	Y	15.83	18.63	11.55	0.2	0.372	0.203
1	CC002	M	Y	7.84	13.45	6.61	0.242	0.118	0.223
1	CC002	F	Y	38.41	21.67	19.76	0.113	0.161	0.139
1	CC002	F	Y	16.41	21.23	11.82	0.122	0.184	0.151
1	CC002	M	Y	7.84	13.45	6.61	0.242	0.118	0.223
1	CC002	M	Y	40.19	24.35	21.16	0.166	0.155	0.157
1	CC002	F	Y	38.41	21.67	19.76	0.113	0.161	0.139
1	CC002	F	Y	32.36	11.62	21.41	0.183	0.098	0.139
2	CC002	M	Y	59.89	8.85	29.22	0.199	0.277	0.146
2	CC002	F	Y	23.15	11.35	14.97	0.309	0.318	0.311
2	CC002	M	Y	43.36	17.72	22.31	0.19	0.306	0.148
0	CC005	F	Y	38.77	17.16	16.52	0.229	0.269	0.228
0	CC005	M	Y	29.98	21.3	15.92	0.205	0.278	0.241
0	CC005	M	Y	25.55	18.61	13.96	0.246	0.266	0.246
1	CC005	F	Y	28.28	9.85	17.8	0.142	0.266	0.172
1	CC005	M	Y	25.15	10.24	17.4	0.274	0.183	0.155
1	CC005	F	Y	28.31	10.1	14.7	0.354	0.348	0.342
1	CC005	M	Y	28.87	9.47	20.2	0.183	0.16	0.283
2	CC005	F	Y	42.94	19.02	22.95	0.147	0.157	0.15
2	CC005	M	Y	33.18	25.5	15.99	0.211	0.31	0.279
2	CC005	M	Y	25.38	8.27	16.99	0.25	0.242	0.175
2	CC005	M	Y	33.18	25.5	15.99	0.211	0.31	0.279
2	CC005	F	Y	15.69	15.74	11.55	0.309	0.397	0.361
0	CC006	F	Y	26	17.49	15.56	0.197	0.245	0.194
0	CC006	M	Y	22.85	9.95	16.38	0.194	0.167	0.13
0	CC006	F	Y	38.26	18.39	23.86	0.198	0.209	0.191
1	CC006	F	Y	33.9	20.72	19.27	0.134	0.133	0.136
1	CC006	M	Y	39.47	23.16	25.74	0.113	0.098	0.123
1	CC006	F	Y	15.35	12.44	10.47	0.335	0.335	0.334
1	CC006	M	Y	19.1	13.79	12	0.3	0.345	0.33
2	CC006	F	Y	41.87	18.22	22.79	0.197	0.201	0.208

Trial#	Strain	Sex	Inf?	StepAngleCV_LH	StepAngleVar_LF	StepAngleVar_LH	Stride_LF	Stride_LH	Stride_RF
2	CC006	M	Y	32.44	13.95	19.12	0.131	0.123	0.195
0	CC011	F	Y	21.71	13.98	15.31	0.14	0.187	0.119
0	CC011	M	Y	27.38	12.97	16.91	0.203	0.184	0.172
0	CC011	F	Y	25.88	9.64	14.38	0.192	0.212	0.215
0	CC011	F	Y	27.35	14.58	17.95	0.212	0.154	0.198
1	CC011	F	Y	28.99	12.07	16.29	0.24	0.272	0.24
1	CC011	M	Y	37.25	14.36	20.9	0.27	0.297	0.288
1	CC011	F	Y	33.8	18.89	13.62	0.292	0.296	0.29
1	CC011	M	Y	31.13	13.56	11.63	0.243	0.257	0.247
2	CC011	F	Y	22.75	9.75	13.99	0.277	0.285	0.272
2	CC011	M	Y	39.74	12.92	22.63	0.211	0.26	0.152
2	CC011	F	Y	21.53	11.26	10.93	0.325	0.307	0.32
2	CC011	M	Y	7.12	16.76	3.37	0.329	0.355	0.345
0	CC012	M	Y	20.02	13.77	11.72	0.334	0.332	0.341
0	CC012	M	Y	37.52	19.39	15.83	0.332	0.239	0.21
0	CC012	M	Y	25.12	13.31	14.23	0.261	0.265	0.264
0	CC012	M	Y	2.92	7.28	2.45	0.259	0.277	0.267
0	CC012	M	Y	32.11	16.61	19.52	0.378	0.377	0.327
0	CC012	F	Y	36.88	22.59	20.89	0.183	0.241	0.197
0	CC012	F	Y	28.43	17.17	16.39	0.277	0.292	0.278
0	CC012	F	Y	42.91	6.23	21.64	0.166	0.208	0.27
0	CC012	F	Y	38.53	17.74	20.43	0.2	0.186	0.169
0	CC012	F	Y	50.56	17.39	19.83	0.118	0.153	0.134
2	CC012	M	Y	21.27	9.5	13.6	0.328	0.354	0.291
2	CC012	M	Y	28.42	10.69	14.21	0.366	0.383	0.385
2	CC012	M	Y	21.91	11.78	9.66	0.334	0.333	0.339
2	CC012	M	Y	24.54	21.56	14.12	0.421	0.419	0.431
2	CC012	M	Y	37.03	20.16	15.57	0.347	0.371	0.329
2	CC012	F	Y	19.33	17.28	10.02	0.294	0.37	0.38
2	CC012	F	Y	43.82	13.51	16.99	0.231	0.254	0.265
2	CC012	F	Y	44.01	19.44	22.05	0.198	0.283	0.192
2	CC012	F	Y	9.5	2.5	6.82	0.321	0.419	0.238
2	CC012	F	Y	36.19	18.7	18	0.244	0.304	0.263
1	CC012XCC032	F	Y	11.01	24.26	9.08	0.147	0.135	0.148
1	CC012xCC032	F	Y	38.83	17.66	24.11	0.098	0.105	0.128
1	CC012XCC032	M	Y	27.51	18.88	15.28	0.254	0.312	0.26
1	CC012xCC032	M	Y	64.19	25.45	28.19	0.223	0.254	0.246
1	CC012XCC032	M	Y	42.55	23.06	21.37	0.117	0.163	0.138
1	CC012xCC032	M	Y	37.31	19.78	21.9	0.153	0.144	0.119
2	CC012XCC032	F	Y	21.32	17.65	11.47	0.266	0.335	0.22
2	CC012XCC032	F	Y	29.2	16.72	13.81	0.374	0.367	0.437
2	CC012XCC032	M	Y	26.95	11.58	19.31	0.207	0.345	0.172
2	CC012XCC032	M	Y	21.88	23.12	13.49	0.194	0.316	0.241
2	CC012XCC032	M	Y	42.48	11.52	20.43	0.301	0.308	0.312
2	CC012XCC032	M	Y	30.59	16.86	16.65	0.289	0.318	0.276
1	CC013xCC041	F	Y	26.39	20.32	18.56	0.166	0.176	0.233
1	CC013xCC041	F	Y	38.54	15.35	22.75	0.083	0.076	0.11
1	CC013xCC041	F	Y	55.71	25.1	22.13	0.129	0.149	0.134

Trial#	Strain	Sex	Inf?	StepAngleCV_LH	StepAngleVar_LF	StepAngleVar_LH	Stride_LF	Stride_LH	Stride_RF
1	CC013xCC041	M	Y	62.84	21.42	19.67	0.167	0.19	0.17
1	CC013xCC041	M	Y	41.11	15.2	21.78	0.258	0.251	0.14
2	CC013XCC041	F	Y	34.71	9.16	17.09	0.294	0.296	0.288
2	CC013XCC041	F	Y	42.48	16.87	19.71	0.241	0.27	0.257
2	CC013XCC041	F	Y	25.04	11.21	10.85	0.289	0.327	0.341
2	CC013XCC041	M	Y	35.41	21.18	21.01	0.174	0.27	0.212
2	CC013XCC041	M	Y	29.79	23.83	21.24	0.108	0.142	0.104
0	CC015	F	Y	34.12	16.36	17.58	0.243	0.212	0.232
0	CC015	M	Y	30.6	12.25	16.31	0.29	0.285	0.285
0	CC015	M	Y	31.64	4.29	16.48	0.252	0.247	0.236
1	CC015	F	Y	27.89	15.86	10.97	0.278	0.28	0.282
1	CC015	M	Y	33.37	18.52	18.04	0.127	0.116	0.145
1	CC015	M	Y	44.28	23.67	21.46	0.151	0.112	0.176
1	CC015	M	Y	21.45	13.38	16.7	0.201	0.248	0.171
2	CC015	F	Y	33.18	20.84	16.61	0.249	0.272	0.27
0	CC017	F	Y	63.96	15.96	17.59	0.158	0.161	0.137
0	CC017	M	Y	47.06	20.69	24.61	0.217	0.143	0.219
0	CC017	F	Y	6.54	14.47	5.11	0.193	0.264	0.178
0	CC017	M	Y	33.44	13.05	20.88	0.218	0.289	0.154
1	CC017	M	Y	48.23	24.54	18.09	0.184	0.208	0.184
1	CC017	F	Y	50.18	14	23.86	0.156	0.185	0.2
0	CC023	M	Y	22.03	11.04	9.02	0.273	0.28	0.291
0	CC023	F	Y	12.24	16.81	9.74	0.226	0.299	0.246
0	CC023	F	Y	45.3	14.35	23.52	0.167	0.147	0.128
0	CC023	M	Y	40.4	23.84	16.34	0.112	0.234	0.124
1	CC023	M	Y	19.5	18.76	14.85	0.104	0.157	0.112
1	CC023	F	Y	9.58	17.5	7.23	0.142	0.179	0.127
1	CC023	M	Y	30.47	13	19.03	0.172	0.274	0.139
1	CC023	M	Y	33	29.99	13.42	0.258	0.251	0.234
2	CC023	M	Y	38.55	16.25	18.61	0.356	0.29	0.347
2	CC023	F	Y	46.76	12.83	22.67	0.274	0.305	0.212
2	CC023	M	Y	20.46	22.65	14.1	0.154	0.347	0.196
0	CC025	M	Y	22.98	19.77	15.02	0.204	0.212	0.213
0	CC025	F	Y	33.41	12.54	20.08	0.213	0.243	0.251
0	CC025	M	Y	46.1	27.09	19.3	0.276	0.266	0.229
1	CC025	M	Y	11.29	20.38	8.28	0.205	0.23	0.21
1	CC025	F	Y	37.09	20.85	23.13	0.186	0.178	0.195
1	CC025	F	Y	59.97	20.47	23.8	0.214	0.209	0.193
1	CC025	F	Y	17.11	10.39	12.09	0.246	0.291	0.228
2	CC025	F	Y	19.78	13.78	13.8	0.307	0.343	0.345
2	CC025	M	Y	38.4	21.84	16.75	0.379	0.394	0.384
0	CC027	F	Y	52.06	12.36	23.85	0.233	0.221	0.19
0	CC027	M	Y	20.38	8.7	12.16	0.181	0.261	0.159
1	CC027	M	Y	43.72	16.06	16.13	0.18	0.19	0.202
1	CC027	M	Y	43.72	16.06	16.13	0.18	0.19	0.202
1	CC027	F	Y	27.82	23.37	17.62	0.129	0.137	0.124
1	CC027	F	Y	8.97	13.39	7.34	0.241	0.261	0.187
1	CC027	M	Y	40.16	15.25	23.96	0.292	0.419	0.413

Trial#	Strain	Sex	Inf?	StepAngleCV_LH	StepAngleVar_LF	StepAngleVar_LH	Stride_LF	Stride_LH	Stride_RF
1	CC027	M	Y	21.95	17.6	13.13	0.328	0.331	0.327
2	CC027	F	Y	46.76	12.83	22.67	0.274	0.305	0.212
2	CC027	M	Y	30.18	24.41	15.82	0.255	0.209	0.347
2	CC027	F	Y	13.01	23.04	9.89	0.193	0.235	0.151
2	CC027	M	Y	27.06	16.26	13.97	0.299	0.361	0.365
1	CC032XCC013	M	Y	30.46	18.16	16.81	0.237	0.255	0.191
1	CC032XCC013	M	Y	26.38	22.5	15.11	0.281	0.305	0.295
1	CC032XCC013	M	Y	47.2	22.24	19.19	0.111	0.147	0.143
1	CC032XCC013	F	Y	67.79	27.78	28.49	0.129	0.16	0.199
1	CC032XCC013	F	Y	39.85	21.36	11.98	0.157	0.227	0.178
1	CC032XCC013	F	Y	29.79	19.09	13.29	0.223	0.296	0.27
1	CC032XCC013	F	Y	40.74	18.47	16.93	0.331	0.34	0.271
1	CC032XCC013	F	Y	54.04	20.79	19.16	0.246	0.219	0.297
2	CC032XCC013	M	Y	63.06	21.94	25.28	0.239	0.255	0.217
2	CC032XCC013	M	Y	33.19	20.4	17.24	0.202	0.233	0.145
2	CC032XCC013	M	Y	45.27	20.3	24.91	0.15	0.273	0.149
2	CC032XCC013	M	Y	20.65	20.66	13.82	0.272	0.321	0.148
2	CC032XCC013	F	Y	28.56	3.36	15.59	0.304	0.316	0.3
2	CC032XCC013	F	Y	26.56	23.89	13.5	0.221	0.289	0.263
2	CC032XCC013	F	Y	35.66	23.68	16.24	0.273	0.279	0.202
2	CC032XCC013	F	Y	23.2	14.5	13.7	0.303	0.337	0.295
0	CC037	F	Y	24.01	21.67	17.16	0.188	0.24	0.199
0	CC037	M	Y	23.56	22.51	16.29	0.14	0.201	0.147
0	CC037	M	Y	14.04	27.47	6.76	0.144	0.308	0.207
0	CC037	M	Y	11.14	2.37	8.66	0.225	0.271	0.17
1	CC037	F	Y	33.96	16.43	19.7	0.266	0.337	0.314
1	CC037	M	Y	16.62	18.67	7.89	0.279	0.304	0.31
1	CC037	M	Y	31.6	23.52	17.33	0.252	0.348	0.245
1	CC037	M	Y	36.26	15.14	22.6	0.34	0.346	0.306
2	CC037	M	Y	21.81	20.06	8.54	0.249	0.313	0.211
2	CC037	M	Y	18.83	11.03	11.88	0.39	0.39	0.376
1	CC041XCC012	M	Y	33.16	5.78	20.11	0.26	0.215	0.248
1	CC041XCC012	M	Y	28.44	12.63	14.88	0.204	0.284	0.27
1	CC041XCC012	M	Y	20.15	18.93	14.3	0.248	0.184	0.203
1	CC041XCC012	M	Y	26.16	15.73	16.44	0.158	0.213	0.239
1	CC041XCC012	M	Y	33.05	13.82	10.6	0.289	0.32	0.28
1	CC041XCC012	F	Y	20.04	21.3	12.8	0.238	0.355	0.25
1	CC041XCC012	F	Y	8.44	22.12	6.45	0.308	0.306	0.19
1	CC041XCC012	F	Y	19.78	12.53	12.32	0.316	0.344	0.205
1	CC041XCC012	F	Y	44.32	22.68	13.48	0.245	0.331	0.276
1	CC041XCC012	F	Y	31.94	15.85	13.67	0.292	0.313	0.23
1	CC041XCC012	F	Y	35.73	23.51	19.75	0.282	0.308	0.235
1	CC041XCC012	F	Y	39.12	20.75	17.94	0.318	0.286	0.293
1	CC041XCC012	F	Y	43.45	12.51	25.16	0.291	0.316	0.211
2	CC041XCC012	M	Y	32.72	24.36	21.01	0.317	0.311	0.323
2	CC041XCC012	M	Y	20.3	6.44	12.85	0.449	0.46	0.357
2	CC041XCC012	F	Y	19.94	6.23	12.44	0.389	0.386	0.343
2	CC041XCC012	M	Y	61.99	17.3	22.96	0.199	0.229	0.251

Trial#	Strain	Sex	Inf?	StepAngleCV_LH	StepAngleVar_LF	StepAngleVar_LH	Stride_LF	Stride_LH	Stride_RF
2	CC041XCC012	M	Y	24.46	14.09	14.4	0.349	0.348	0.346
2	CC041XCC012	M	Y	50.36	9.9	18.09	0.263	0.294	0.291
2	CC041XCC012	F	Y	36.92	6.34	16.06	0.409	0.42	0.43
2	CC041XCC012	F	Y	39.51	15.71	20.86	0.296	0.332	0.334
2	CC041XCC012	F	Y	27.34	6.23	16.3	0.353	0.368	0.325
2	CC041XCC012	F	Y	21.38	11.79	9.38	0.339	0.341	0.346
2	CC041XCC012	F	Y	26.05	18.11	12.22	0.322	0.382	0.329
2	CC041XCC012	F	Y	34.39	11.7	18.36	0.356	0.375	0.365
2	CC041XCC012	F	Y	22.28	11.49	12.03	0.349	0.351	0.319
0	CC051	F	Y	25.23	18.84	16.18	0.249	0.278	0.24
0	CC051	F	Y	14.5	22.53	9.7	0.2	0.267	0.19
0	CC051	F	Y	20.7	15.87	9.95	0.246	0.253	0.255
0	CC051	M	Y	24.43	16.08	9.16	0.229	0.234	0.205
1	CC051	F	Y	26.88	10.73	15.13	0.255	0.303	0.303
1	CC051	F	Y	36.72	15.51	12.9	0.28	0.287	0.234
1	CC051	F	Y	31.83	20.19	16.35	0.262	0.335	0.287
0	CC057	F	Y	30.29	16.46	15.29	0.257	0.263	0.263
0	CC057	F	Y	45.39	19.43	19.69	0.2	0.221	0.237
0	CC057	F	Y	24.09	20.98	14.1	0.176	0.178	0.17
0	CC057	M	Y	45.36	22.83	20.37	0.117	0.188	0.239
0	CC057	M	Y	38.84	6.17	18.84	0.209	0.105	0.168
0	CC057	M	Y	43.07	24.92	14.65	0.16	0.159	0.119
1	CC057	F	Y	46.22	16.69	19.37	0.242	0.241	0.243
1	CC057	F	Y	35.66	16.53	18.48	0.162	0.176	0.174
1	CC057	F	Y	51.85	17.36	22.65	0.144	0.136	0.117
1	CC057	F	Y	50.1	25.58	19.66	0.168	0.161	0.16
1	CC057	M	Y	21.78	24.41	8.98	0.266	0.29	0.258
1	CC057	M	Y	34.44	20.89	11.1	0.263	0.263	0.254
2	CC057	F	Y	50.1	25.58	19.66	0.168	0.161	0.16
0	CC078	F	Y	18.45	11.76	13.27	0.244	0.255	0.242
0	CC078	F	Y	31.96	13.34	17.61	0.251	0.204	0.249
0	CC078	F	Y	26.89	10.42	11.97	0.261	0.252	0.253
0	CC078	M	Y	30.75	14.86	16.52	0.245	0.245	0.245
1	CC078	F	Y	19.22	14.45	8.55	0.25	0.282	0.279
1	CC078	F	Y	15.19	24.81	9.96	0.244	0.274	0.247
1	CC078	M	Y	45.49	17.04	22.41	0.25	0.241	0.237
2	CC078	F	Y	43.09	13.72	20.35	0.244	0.235	0.192
2	CC078	M	Y	40.61	21.93	23.04	0.22	0.269	0.23
2	CC005	M	N	30.54	5.52	20.35	0.144	0.162	0.215
2	CC005	M	Y	7.22	14.59	6.09	0.182	0.208	0.166
2	CC015	F	N	32.77	23.4	18.44	0.122	0.157	0.108
2	CC015	M	N	22.79	18.43	13.55	0.308	0.356	0.343
2	CC015	M	Y	14.64	17.95	9.8	0.293	0.33	0.291
2	CC015	M	Y	40.41	18.53	24.29	0.179	0.22	0.168
2	CC017	F	N	34.72	11.05	18.29	0.275	0.336	0.179
2	CC017	M	N	53.01	23.72	22.25	0.14	0.199	0.13
2	CC017	F	Y	34.63	19.05	18.13	0.241	0.274	0.331
2	CC023	F	N	32.65	13.9	18.41	0.279	0.305	0.343

Trial#	Strain	Sex	Inf?	StepAngleCV_LH	StepAngleVar_LF	StepAngleVar_LH	Stride_LF	Stride_LH	Stride_RF

Table S3. Raw DigiGait measurement data. Far left column indicates time point at which data was measured: T0 = pre-infection, T1 = 21dpi, and T3 = 89dpi. DigiGait parameters listed across the top indicate which limb is associated with the data, where appropriate: FL for left fore limb, FR for right fore limb, HL for left hind limb, and HR for right hind limb.

Table S3

Trial#	Strain	Sex	Inf?	Stride_RH	StrideFrequency_LF	StrideFrequency_LH	StrideFrequency_RF
1	CC002	F	N	0.237	4.7	6.2	4.5
1	CC025	F	N	0.208	4.9	4.2	4.7
1	CC012XCC032	F	N	0.301	3.5	3	3.4
2	CC012XCC032	F	N	0.378	3.2	2.5	2.5
1	CC012XCC032	M	N	0.322	3.9	3.2	3.4
2	CC012XCC032	M	N	0.295	2.9	3.5	3
1	CC013xCC041	F	N	0.193	4.1	6.8	5.2
2	CC013XCC041	F	N	0.339	3.1	3	2.9
1	CC013xCC041	M	N	0.071	11	7	7.1
2	CC013XCC041	M	N	0.302	3.9	3.4	4
1	CC032XCC013	F	N	0.117	8.5	5.3	11.3
2	CC032XCC013	F	N	0.324	4.2	4.1	4
1	CC041XCC012	F	N	0.187	5.5	6	3.6
1	CC041XCC012	F	N	0.169	8.6	8.5	7.8
2	CC041XCC012	F	N	0.233	4.8	3.2	6.1
1	CC032XCC013	M	N	0.134	7.1	6.2	6.7
2	CC032XCC013	M	N	0.146	8	3.3	9.4
1	CC041XCC012	M	N	0.216	3.9	3.5	5.7
2	CC041XCC012	M	N	0.36	3.2	2.7	3.2
0	CC012	F	N	0.252	5.2	4.1	4.8
2	CC012	F	N	0.38	2.4	2.4	3.2
0	CC012	M	N	0.233	4.9	5.9	4.2
2	CC012	M	N	0.341	5.8	3.4	6.1
0	CC057	F	N	0.187	3.6	3.5	4.3
1	CC057	F	N	0.294	3.4	3.5	3.5
0	CC057	M	N	0.256	4.8	3.5	3.3
1	CC057	M	N	0.351	2.9	2.9	2.8
0	CC078	F	N	0.281	3.7	3.6	3.5
1	CC078	F	N	0.284	3.5	3.5	4.1
2	CC078	F	N	0.186	6.3	4.7	7.5
0	CC078	M	N	0.247	4.2	4.2	4.2
2	CC078	M	N	0.253	4.2	3.8	4.6
0	CC002	F	N	0.102	4.7	7.9	6
1	CC002	F	N	0.237	4.7	6.2	4.5
0	CC002	M	N	0.108	7.5	6	5.6
1	CC002	M	N	0.249	7.2	6	4.6
2	CC002	M	N	0.283	2.9	2.6	3.1
0	CC006	F	N	0.287	3.5	3.6	3.4
1	CC006	F	N	0.311	5.3	4.2	4
2	CC006	F	N	0.156	7.4	5.4	4.6
0	CC006	M	N	0.095	5.5	6.1	4.7
0	CC023	F	N	0.252	4	5.2	4.1
1	CC023	F	N	0.236	5.1	4.2	5.9
2	CC023	F	N	0.3	3	2.9	5
0	CC023	M	N	0.254	3.5	3.6	3.5
1	CC023	M	N	0.284	3.9	3.5	3.5
0	CC027	F	N	0.136	5.2	6.4	8.9

Trial#	Strain	Sex	Inf?	Stride_RH	StrideFrequency_LF	StrideFrequency_LH	StrideFrequency_RF
1	CC027	F	N	0.243	4.3	5	4.8
2	CC027	F	N	0.263	3.5	3	3.2
0	CC027	M	N	0.181	5.2	10.2	5.3
1	CC027	M	N	0.124	7.7	6.9	6.5
1	CC027	M	N	0.172	3.7	5.1	5.1
2	CC027	M	N	0.247	3.4	3.6	4.3
0	CC005	F	N	0.254	5.2	4.1	3.9
1	CC005	F	N	0.207	5.3	4.5	4.6
2	CC005	F	N	0.099	8.8	10.9	8.7
0	CC011	F	N	0.174	7.1	6.7	7.1
1	CC011	F	N	0.281	4.8	3.2	5.5
2	CC011	F	N	0.329	3.4	3.1	3.4
1	CC017	F	N	0.217	5.7	4.1	4.5
0	CC005	M	N	0.278	3.6	3.6	3.7
1	CC005	M	N	0.271	5.6	7.1	4.9
2	CC005	M	N	0.212	6.3	5.9	5.9
0	CC011	M	N	0.22	4.6	4.3	4.6
1	CC011	M	N	0.271	3.8	3.9	3.8
2	CC011	M	N	0.203	4.8	4.4	5.9
0	CC017	M	N	0.143	5.2	8.3	6.9
1	CC017	M	N	0.21	6.3	3.7	2.9
1	CC006	F	N	0.112	9.7	12.1	11.3
0	CC037	F	N	0.268	3.8	3.5	4.3
0	CC051	F	N	0.287	5.3	3.3	5
1	CC051	F	N	0.223	6.8	5.9	4
1	CC006	M	N	0.319	4	3.4	3.6
0	CC037	M	N	0.287	4.5	3.5	3.6
1	CC037	M	N	0.247	3.9	3.7	3.4
0	CC005	F	N	0.243	4.9	4	4.7
1	CC005	F	N	0.374	3.2	2.8	3.1
2	CC005	F	N	0.352	2.9	2.9	5.6
0	CC011	F	N	0.238	4.2	4.3	4.2
1	CC011	F	N	0.278	3.7	3.6	3.7
2	CC011	F	N	0.284	3.3	3.5	3.5
0	CC011	M	N	0.272	3.8	3.8	3.6
1	CC011	M	N	0.307	3.1	3.4	3.3
2	CC011	M	N	0.307	3.4	3.4	3.3
1	CC037	M	N	0.288	3.3	3.4	5.2
2	CC037	M	N	0.337	4	3.6	5.3
1	CC051	M	N	0.302	3.4	3.5	3.2
2	CC051	M	N	0.311	3.8	3.4	3.9
0	CC027	F	N	0.357	2.9	2.9	2.8
1	CC027	F	N	0.217	3.6	4.9	4.4
2	CC027	F	N	0.269	4.5	4	4
0	CC015	M	N	0.265	3.9	3.7	3.9
1	CC015	M	N	0.203	4.3	3.6	4.6
2	CC015	M	N	0.337	3	3	3.5
0	CC027	M	N	0.3	3.9	3.9	3.4

Trial#	Strain	Sex	Inf?	Stride_RH	StrideFrequency_LF	StrideFrequency_LH	StrideFrequency_RF
1	CC027	M	N	0.265	3.5	3.1	2.9
2	CC027	M	N	0.22	4.4	5.4	3.7
0	CC015	F	N	3.6	3.6	3.5	3.5
1	CC015	F	N	0.119	8.3	7.8	8.3
0	CC017	F	N	0.273	4.1	3.6	3.9
0	CC023	F	N	0.236	4	4.3	4.2
1	CC023	F	N	0.3	3.3	3.5	3.9
0	CC005	M	N	0.242	3.7	4.6	3.9
1	CC005	M	N	0.358	3	2.9	2.8
0	CC015	M	N	0.295	3.4	3.5	3.4
1	CC015	M	N	0.114	4.7	3.4	7.2
0	CC017	M	N	0.267	3.8	4.1	4.2
1	CC017	M	N	0.135	8	9	8.9
0	CC023	M	N	0.323	3.1	3.2	3.1
1	CC023	M	N	0.312	3.9	3.4	3.2
0	CC051	M	N	0.289	3.5	3.1	3.4
0	CC002	F	Y	0.134	7.2	6.5	7.1
0	CC002	M	Y	0.262	5.1	2.7	5
1	CC002	M	Y	0.208	4.5	9.6	4.5
1	CC002	F	Y	0.102	9.3	6.9	7.4
1	CC002	F	Y	0.161	8.6	5.6	6.9
1	CC002	M	Y	0.208	4.5	9.6	4.5
1	CC002	M	Y	0.118	6.2	6.7	6.6
1	CC002	F	Y	0.102	9.3	6.9	7.4
1	CC002	F	Y	0.1	5.7	11	7.5
2	CC002	M	Y	0.148	5.1	3.7	7
2	CC002	F	Y	0.346	3.3	3.2	3.2
2	CC002	M	Y	0.185	5.3	3.4	7
0	CC005	F	Y	0.268	4.4	3.8	4.5
0	CC005	M	Y	0.277	5	3.7	4.2
0	CC005	M	Y	0.264	4.2	3.7	4.2
1	CC005	F	Y	0.24	7.3	3.9	5.9
1	CC005	M	Y	0.116	3.7	5.5	6.7
1	CC005	F	Y	0.355	2.8	2.9	2.9
1	CC005	M	Y	0.226	5.5	6.5	3.5
2	CC005	F	Y	0.168	7	6.6	6.8
2	CC005	M	Y	0.29	4.8	3.3	3.6
2	CC005	M	Y	0.169	4	4.3	5.9
2	CC005	M	Y	0.29	4.8	3.3	3.6
2	CC005	F	Y	0.265	3.2	2.6	2.7
0	CC006	F	Y	0.241	5.3	4.2	5.3
0	CC006	M	Y	0.111	5.3	6.3	7.9
0	CC006	F	Y	0.209	5.2	4.9	5.4
1	CC006	F	Y	0.148	7.7	7.8	7.5
1	CC006	M	Y	0.112	9.3	10.9	8.5
1	CC006	F	Y	0.322	3	3.1	3
1	CC006	M	Y	0.355	3.4	2.9	3
2	CC006	F	Y	0.201	5.1	5.1	4.8

Trial#	Strain	Sex	Inf?	Stride_RH	StrideFrequency_LF	StrideFrequency_LH	StrideFrequency_RF
2	CC006	M	Y	0.156	8	8.5	5.3
0	CC011	F	Y	0.247	7.4	5.5	8.8
0	CC011	M	Y	0.177	5.1	5.4	5.9
0	CC011	F	Y	0.216	5.3	4.7	4.7
0	CC011	F	Y	0.133	4.8	6.5	5.5
1	CC011	F	Y	0.265	4.2	3.6	4.2
1	CC011	M	Y	0.272	3.8	3.5	3.6
1	CC011	F	Y	0.289	3.4	3.4	3.5
1	CC011	M	Y	0.278	4	3.9	4.2
2	CC011	F	Y	0.288	3.7	3.6	3.7
2	CC011	M	Y	0.345	4.9	4	6.8
2	CC011	F	Y	0.313	3.1	3.2	3.1
2	CC011	M	Y	0.343	3	2.9	2.9
0	CC012	M	Y	0.295	3	3.1	3
0	CC012	M	Y	0.239	3	4.2	4.8
0	CC012	M	Y	0.263	3.9	3.9	3.8
0	CC012	M	Y	0.264	3.9	3.7	3.8
0	CC012	M	Y	0.293	2.6	2.7	3.1
0	CC012	F	Y	0.269	5.6	4.2	5.1
0	CC012	F	Y	0.284	3.6	3.4	3.6
0	CC012	F	Y	0.238	6.2	4.9	3.8
0	CC012	F	Y	0.201	5.1	5.5	6.1
0	CC012	F	Y	0.121	8.4	6.2	7.4
2	CC012	M	Y	0.332	3	3	3.4
2	CC012	M	Y	0.381	2.7	2.7	2.6
2	CC012	M	Y	0.351	3.1	3.1	3
2	CC012	M	Y	0.417	2.4	2.4	2.2
2	CC012	M	Y	0.366	2.9	2.7	3
2	CC012	F	Y	0.35	3.4	2.7	2.6
2	CC012	F	Y	0.262	4.3	4	3.8
2	CC012	F	Y	0.201	5.2	3.6	5.3
2	CC012	F	Y	0.37	3.1	2.5	4.3
2	CC012	F	Y	0.294	4.5	3.3	3.9
1	CC012XCC032	F	Y	0.103	7.1	7.7	7
1	CC012xCC032	F	Y	0.092	10.8	10	8.1
1	CC012XCC032	M	Y	0.325	4	3.3	3.9
1	CC012xCC032	M	Y	0.28	4.6	4	4.1
1	CC012XCC032	M	Y	0.118	8.9	6.1	7.5
1	CC012xCC032	M	Y	0.111	6.8	7.1	8.8
2	CC012XCC032	F	Y	0.393	3.7	3	4.5
2	CC012XCC032	F	Y	0.426	2.7	2.7	2.2
2	CC012XCC032	M	Y	0.239	4.9	2.8	5.9
2	CC012XCC032	M	Y	0.365	5.2	3.2	4.3
2	CC012XCC032	M	Y	0.31	3.3	3.3	3.3
2	CC012XCC032	M	Y	0.414	3.4	3.3	3.7
1	CC013xCC041	F	Y	0.141	6.2	5.9	4.3
1	CC013xCC041	F	Y	0.088	13	14.3	9.5
1	CC013xCC041	F	Y	0.155	8	7	7.8

Trial#	Strain	Sex	Inf?	Stride_RH	StrideFrequency_LF	StrideFrequency_LH	StrideFrequency_RF
1	CC013xCC041	M	Y	0.204	6.1	5.3	6
1	CC013xCC041	M	Y	0.12	4	3.9	7.4
2	CC013XCC041	F	Y	0.296	3.4	3.4	3.5
2	CC013XCC041	F	Y	0.401	4.2	3.7	4
2	CC013XCC041	F	Y	0.353	3.4	3	3
2	CC013XCC041	M	Y	0.228	5.9	3.8	4.8
2	CC013XCC041	M	Y	0.098	9.8	7.5	10.1
0	CC015	F	Y	0.243	4.2	4.8	4.4
0	CC015	M	Y	0.287	3.4	3.6	3.6
0	CC015	M	Y	0.219	4	4.1	4.3
1	CC015	F	Y	0.255	3.6	3.7	3.5
1	CC015	M	Y	0.13	8.2	9.1	7.1
1	CC015	M	Y	0.126	6.9	9.4	5.9
1	CC015	M	Y	0.201	5.1	4.1	6
2	CC015	F	Y	0.271	4.1	3.7	3.7
0	CC017	F	Y	0.131	6.6	6.4	7.5
0	CC017	M	Y	0.196	4.7	7.3	4.5
0	CC017	F	Y	0.237	5.3	3.9	5.7
0	CC017	M	Y	0.245	4.7	3.5	6.7
1	CC017	M	Y	0.233	5.4	4.8	5.4
1	CC017	F	Y	0.172	6.6	5.6	5
0	CC023	M	Y	0.29	3.7	3.6	3.5
0	CC023	F	Y	0.136	4.5	3.5	4.2
0	CC023	F	Y	0.152	6	7	8.1
0	CC023	M	Y	0.24	9	4.6	8.4
1	CC023	M	Y	0.208	10.1	6.6	9.3
1	CC023	F	Y	0.243	7.3	5.7	8.2
1	CC023	M	Y	0.196	5.9	3.7	7.5
1	CC023	M	Y	0.285	3.7	4.1	4.1
2	CC023	M	Y	0.351	2.8	3.2	3
2	CC023	F	Y	0.331	3.6	3.3	4.8
2	CC023	M	Y	0.234	6.7	3	5.2
0	CC025	M	Y	0.208	5.1	4.8	4.8
0	CC025	F	Y	0.246	4.8	4.2	4.2
0	CC025	M	Y	0.307	3.6	3.8	4.2
1	CC025	M	Y	0.211	5	4.5	4.8
1	CC025	F	Y	0.199	5.5	5.9	5.3
1	CC025	F	Y	0.205	4.8	4.9	5.3
1	CC025	F	Y	0.234	4.2	3.5	4.4
2	CC025	F	Y	0.328	3.2	3	2.9
2	CC025	M	Y	0.391	2.7	2.6	2.6
0	CC027	F	Y	0.228	4.3	4.7	5.3
0	CC027	M	Y	0.36	5.6	3.7	6.5
1	CC027	M	Y	0.14	6.2	5.4	5.1
1	CC027	M	Y	0.14	6.2	5.4	5.1
1	CC027	F	Y	0.096	8.1	7.4	8.3
1	CC027	F	Y	0.162	4.3	3.9	5.5
1	CC027	M	Y	0.433	3.4	2.4	2.2

Trial#	Strain	Sex	Inf?	Stride_RH	StrideFrequency_LF	StrideFrequency_LH	StrideFrequency_RF
1	CC027	M	Y	0.323	3.1	3.1	3
2	CC027	F	Y	0.331	3.6	3.3	4.8
2	CC027	M	Y	0.363	4	4.9	2.9
2	CC027	F	Y	0.2	5.6	4.4	6.8
2	CC027	M	Y	0.363	3.3	2.8	2.8
1	CC032XCC013	M	Y	0.235	4.2	4	5.4
1	CC032XCC013	M	Y	0.309	3.6	3.3	3.5
1	CC032XCC013	M	Y	0.163	9.4	7	7.2
1	CC032XCC013	F	Y	0.168	7.9	6.3	5.1
1	CC032XCC013	F	Y	0.205	6.5	4.3	5.7
1	CC032XCC013	F	Y	0.283	4.5	3.4	3.7
1	CC032XCC013	F	Y	0.285	3	3	3.7
1	CC032XCC013	F	Y	0.275	4.1	4.6	3.2
2	CC032XCC013	M	Y	0.261	4.1	4	4.4
2	CC032XCC013	M	Y	0.171	4.9	4.4	6.9
2	CC032XCC013	M	Y	0.219	6.9	3.8	7
2	CC032XCC013	M	Y	0.225	3.7	3.1	6.9
2	CC032XCC013	F	Y	0.306	3.3	3.2	3.4
2	CC032XCC013	F	Y	0.297	4.6	3.5	3.9
2	CC032XCC013	F	Y	0.308	3.7	3.6	5
2	CC032XCC013	F	Y	0.35	3.4	3	3.3
0	CC037	F	Y	0.299	5.5	4.3	5.1
0	CC037	M	Y	0.251	7.4	5.1	7.1
0	CC037	M	Y	0.3	7.1	3.2	5
0	CC037	M	Y	0.158	5.2	3.7	6
1	CC037	F	Y	0.326	3.8	3	3.2
1	CC037	M	Y	0.299	3.6	3.4	3.3
1	CC037	M	Y	0.342	4	2.9	4.2
1	CC037	M	Y	0.266	2.9	2.9	3.3
2	CC037	M	Y	0.302	4.1	3.1	4.9
2	CC037	M	Y	0.385	2.6	2.6	2.6
1	CC041XCC012	M	Y	0.303	3.9	4.7	4.1
1	CC041XCC012	M	Y	0.276	5	3.6	3.7
1	CC041XCC012	M	Y	0.159	4	5.5	5
1	CC041XCC012	M	Y	0.199	6.6	4.8	4.2
1	CC041XCC012	M	Y	0.322	3.5	3.2	3.6
1	CC041XCC012	F	Y	0.305	4.3	2.8	4
1	CC041XCC012	F	Y	0.27	3.3	3.3	5.3
1	CC041XCC012	F	Y	0.289	3.2	3	5
1	CC041XCC012	F	Y	0.325	4.3	3.1	3.7
1	CC041XCC012	F	Y	0.278	3.4	3.3	4.3
1	CC041XCC012	F	Y	0.294	3.6	3.3	4.2
1	CC041XCC012	F	Y	0.31	3.1	3.6	3.5
1	CC041XCC012	F	Y	0.269	3.4	3.2	4.9
2	CC041XCC012	M	Y	0.297	3.2	3.3	3.1
2	CC041XCC012	M	Y	0.466	2.3	2.2	2.8
2	CC041XCC012	F	Y	0.401	2.5	2.6	2.9
2	CC041XCC012	M	Y	0.297	5.2	4.4	3.9

Trial#	Strain	Sex	Inf?	Stride_RH	StrideFrequency_LF	StrideFrequency_LH	StrideFrequency_RF
2	CC041XCC012	M	Y	0.342	2.9	2.9	2.9
2	CC041XCC012	M	Y	0.332	3.8	3.5	3.5
2	CC041XCC012	F	Y	0.424	2.4	2.4	2.3
2	CC041XCC012	F	Y	0.333	3.3	3.1	3
2	CC041XCC012	F	Y	0.335	2.8	2.7	3
2	CC041XCC012	F	Y	0.341	3	3	2.8
2	CC041XCC012	F	Y	0.37	3.2	2.7	3
2	CC041XCC012	F	Y	0.357	2.8	2.7	2.7
2	CC041XCC012	F	Y	0.355	2.9	2.8	3.3
0	CC051	F	Y	0.277	4.1	3.7	4.2
0	CC051	F	Y	0.25	5.2	3.8	5.4
0	CC051	F	Y	0.274	4.1	4	3.9
0	CC051	M	Y	0.226	4.3	4.4	5
1	CC051	F	Y	0.294	4	3.4	3.3
1	CC051	F	Y	0.283	3.7	3.6	4.3
1	CC051	F	Y	0.278	3.9	3	3.5
0	CC057	F	Y	0.258	4	3.9	3.9
0	CC057	F	Y	0.219	5.1	4.7	4.2
0	CC057	F	Y	0.148	5.8	5.8	6.1
0	CC057	M	Y	0.184	8.9	5.5	4.3
0	CC057	M	Y	0.098	4.9	9.9	6.2
0	CC057	M	Y	0.126	6.5	6.5	8.7
1	CC057	F	Y	0.256	4.2	4.3	4.2
1	CC057	F	Y	0.174	6.4	5.8	5.9
1	CC057	F	Y	0.138	7.2	7.7	8.9
1	CC057	F	Y	0.171	6.1	6.4	6.4
1	CC057	M	Y	0.333	3.8	3.4	4
1	CC057	M	Y	0.267	3.8	3.9	3.9
2	CC057	F	Y	0.171	6.1	6.4	6.4
0	CC078	F	Y	0.202	4.2	3.8	4.2
0	CC078	F	Y	0.227	4.1	5	4.1
0	CC078	F	Y	0.26	3.9	3.9	4
0	CC078	M	Y	0.25	4.1	4.2	4.2
1	CC078	F	Y	0.282	4	3.6	3.7
1	CC078	F	Y	0.245	4.2	3.7	4.1
1	CC078	M	Y	0.257	4.1	4	4.2
2	CC078	F	Y	0.208	4.1	4.4	5.3
2	CC078	M	Y	0.263	4.6	3.8	4.5
2	CC005	M	N	0.139	7.2	6.4	4.8
2	CC005	M	Y	0.249	5.7	5	6.3
2	CC015	F	N	0.161	8.7	6.6	9.7
2	CC015	M	N	0.342	3.3	2.8	2.9
2	CC015	M	Y	0.293	3.5	3.1	3.4
2	CC015	M	Y	0.186	5.8	4.7	6.2
2	CC017	F	N	0.301	3.6	3	5.8
2	CC017	M	N	0.23	7.3	5.2	8
2	CC017	F	Y	0.29	4.3	3.7	3.1
2	CC023	F	N	0.32	3.6	3.3	3

Trial#	Strain	Sex	Inf?	Stride_RH	StrideFrequency_LF	StrideFrequency_LH	StrideFrequency_RF

Table S3. Raw DigiGait measurement data. Far left column indicates time point at which data was measured: T0 = pre-infection, T1 = 21dpi, and T3 = 89dpi. DigiGait parameters listed across the top indicate which limb is associated with the data, where appropriate: FL for left fore limb, FR for right fore limb, HL for left hind limb, and HR for right hind limb.

Table S3

Trial#	Strain	Sex	Inf?	StrideFrequency_RH	StrideLength_LF	StrideLength_LH	StrideLength_RF	StrideLength_RH
1	CC002	F	N	4.3	3.3	2.5	3.4	3.5
1	CC025	F	N	4.9	3.1	3.6	3.3	3.1
1	CC012XCC032	F	N	3.4	4.3	5.2	4.5	4.5
2	CC012XCC032	F	N	2.6	4.8	5.9	5.9	5.7
1	CC012XCC032	M	N	3.3	3.9	4.7	4.5	4.8
2	CC012XCC032	M	N	3.6	5.1	4.3	5	4.4
1	CC013xCC041	F	N	5.3	3.7	2.3	2.9	2.9
2	CC013XCC041	F	N	3	5	5.1	5	5.1
1	CC013xCC041	M	N	15	1.4	2.1	2.1	1.1
2	CC013XCC041	M	N	3.4	3.8	4.2	3.8	4.5
1	CC032XCC013	F	N	8.9	1.8	2.9	1.4	1.8
2	CC032XCC013	F	N	3.1	3.7	3.7	3.8	4.9
1	CC041XCC012	F	N	5.5	2.8	2.6	4.2	2.8
1	CC041XCC012	F	N	6	1.8	1.8	2	2.5
2	CC041XCC012	F	N	4.3	3.2	4.8	2.5	3.5
1	CC032XCC013	M	N	7.7	2.2	2.5	2.3	2
2	CC032XCC013	M	N	7	2	4.6	1.6	2.2
1	CC041XCC012	M	N	4.8	3.9	4.4	2.7	3.2
2	CC041XCC012	M	N	2.8	4.7	5.4	4.7	5.4
0	CC012	F	N	4.1	2.9	3.5	3.2	3.8
2	CC012	F	N	2.5	6.4	6.5	4.7	5.7
0	CC012	M	N	4.3	3.2	2.7	3.6	3.5
2	CC012	M	N	3	2.6	4.5	2.5	5.1
0	CC057	F	N	5.5	4.1	4.4	3.5	2.8
1	CC057	F	N	3.5	4.5	4.3	4.4	4.4
0	CC057	M	N	4	3.1	4.4	4.7	3.8
1	CC057	M	N	2.8	5.2	5.3	5.6	5.3
0	CC078	F	N	3.7	4.2	4.1	4.1	4.2
1	CC078	F	N	3.7	4.3	4.3	3.7	4.3
2	CC078	F	N	5.5	2.4	3.3	2.1	2.8
0	CC078	M	N	3.9	3.6	3.6	3.6	3.7
2	CC078	M	N	4	3.7	3.9	3.4	3.8
0	CC002	F	N	10.4	3.3	2	2.6	1.5
1	CC002	F	N	4.3	3.3	2.5	3.4	3.5
0	CC002	M	N	9.8	2.1	2.6	2.8	1.6
1	CC002	M	N	4.1	2.2	2.5	3.4	3.7
2	CC002	M	N	3.6	5.3	6.1	4.7	4.2
0	CC006	F	N	3.4	4.4	4.4	4.4	4.3
1	CC006	F	N	3.3	2.9	3.7	3.8	4.7
2	CC006	F	N	6.7	2.1	2.9	3.2	2.3
0	CC006	M	N	11	2.8	2.5	3.3	1.4
0	CC023	F	N	4	3.8	2.9	3.8	3.8
1	CC023	F	N	4.3	3	3.7	2.6	3.5
2	CC023	F	N	3.4	4.8	5.2	3.1	4.5
0	CC023	M	N	4.1	4.2	4.3	4.4	3.8
1	CC023	M	N	3.6	4	4.2	4.3	4.3
0	CC027	F	N	7.7	3	2.4	1.8	2

Trial#	Strain	Sex	Inf?	StrideFrequency_RH	StrideLength_LF	StrideLength_LH	StrideLength_RF	StrideLength_RH
1	CC027	F	N	4.2	3.6	3.1	3.2	3.7
2	CC027	F	N	3.9	4.4	5.1	4.7	3.9
0	CC027	M	N	5.7	2.9	1.6	2.9	2.7
1	CC027	M	N	8.4	2	2.3	2.4	1.9
1	CC027	M	N	5.9	4.1	3	2.9	2.6
2	CC027	M	N	4.1	4.3	3.9	3.6	3.7
0	CC005	F	N	4.1	2.9	3.8	3.8	3.8
1	CC005	F	N	5	2.9	3.3	3.4	3.1
2	CC005	F	N	10.7	1.8	1.5	1.8	1.5
0	CC011	F	N	5.8	2.2	2.3	2.1	2.6
1	CC011	F	N	3.6	3.2	4.7	2.8	4.2
2	CC011	F	N	3.1	4.5	5	4.5	4.9
1	CC017	F	N	4.8	2.7	3.7	3.4	3.3
0	CC005	M	N	3.8	4.3	4.2	4	4.2
1	CC005	M	N	3.8	2.7	2.2	3.1	4.1
2	CC005	M	N	4.9	2.5	2.6	2.6	3.2
0	CC011	M	N	4.8	3.3	3.6	3.3	3.3
1	CC011	M	N	3.6	4	3.9	3.9	4.1
2	CC011	M	N	5.1	3.2	3.5	2.6	3.1
0	CC017	M	N	7.2	2.9	1.9	2.2	2.1
1	CC017	M	N	4.7	2.5	4	5.3	3.2
1	CC006	F	N	9.3	1.6	1.3	1.4	1.7
0	CC037	F	N	3.8	6.7	7.2	5.9	6.7
0	CC051	F	N	3.6	4.9	7.7	5.1	7.2
1	CC051	F	N	4.6	2.3	2.6	3.7	3.3
1	CC006	M	N	3.1	3.9	4.5	4.1	4.8
0	CC037	M	N	3.5	3.4	4.3	4.2	4.3
1	CC037	M	N	4.2	3.8	4.1	4.2	3.7
0	CC005	F	N	4.1	3.1	3.8	3.3	3.6
1	CC005	F	N	2.8	4.7	5.5	4.8	5.6
2	CC005	F	N	2.8	5.1	5	2.8	5.3
0	CC011	F	N	4.3	3.7	3.7	3.6	3.6
1	CC011	F	N	3.7	4.1	4.3	4	4.2
2	CC011	F	N	3.6	4.4	4.3	4.3	4.3
0	CC011	M	N	3.7	4	4	4.1	4.1
1	CC011	M	N	3.3	4.7	4.6	4.6	4.6
2	CC011	M	N	3.3	4.4	4.4	4.5	4.6
1	CC037	M	N	3.5	4.5	4.5	3	4.3
2	CC037	M	N	3	3.8	4.3	2.9	5.1
1	CC051	M	N	3.4	4.5	4.4	4.5	4.5
2	CC051	M	N	3.2	3.9	4.4	4	4.7
0	CC027	F	N	2.8	5.2	5.4	5.3	5.4
1	CC027	F	N	4.7	4.3	3.1	3.5	3.3
2	CC027	F	N	3.8	3.4	3.8	3.8	4
0	CC015	M	N	3.9	3.8	4	3.9	4
1	CC015	M	N	5	3.5	4.1	3.4	3
2	CC015	M	N	3	5.1	5	4.4	5.1
0	CC027	M	N	3.2	3.9	3.9	4.4	4.5

Trial#	Strain	Sex	Inf?	StrideFrequency_RH	StrideLength_LF	StrideLength_LH	StrideLength_RF	StrideLength_RH
1	CC027	M	N	3.9	4.3	4.9	5.1	4
2	CC027	M	N	4.7	3.4	2.9	4.1	3.3
0	CC015	F	N	4.2	4.2	4.4	4.4	28.94
1	CC015	F	N	8.8	1.9	2	1.9	1.8
0	CC017	F	N	3.6	3.7	4.2	3.9	4.1
0	CC023	F	N	4.3	3.8	3.5	3.6	3.5
1	CC023	F	N	3.4	4.6	4.3	3.9	4.5
0	CC005	M	N	4.1	4.1	3.4	4	3.6
1	CC005	M	N	3	5.1	5.5	5.2	5.4
0	CC015	M	N	3.5	4.5	4.4	4.4	4.4
1	CC015	M	N	9.1	3.2	4.5	2.2	1.7
0	CC017	M	N	3.8	4	3.7	3.5	4
1	CC017	M	N	7.7	2	1.7	1.8	2
0	CC023	M	N	3.1	4.8	4.8	4.9	4.8
1	CC023	M	N	3.3	3.9	4.5	4.7	4.7
0	CC051	M	N	3.5	4.4	5	4.6	4.3
0	CC002	F	Y	7.7	2.2	2.4	2.2	2
0	CC002	M	Y	3.9	3	5.6	3	3.9
1	CC002	M	Y	5.2	3.6	1.8	3.3	3.1
1	CC002	F	Y	10.2	0.8	1.1	1	0.7
1	CC002	F	Y	6.4	1.8	2.8	2.3	2.4
1	CC002	M	Y	5.2	3.6	1.8	3.3	3.1
1	CC002	M	Y	8.9	2.5	2.3	2.4	1.8
1	CC002	F	Y	10.2	0.8	1.1	1	0.7
1	CC002	F	Y	10.6	2.4	1.3	1.8	1.3
2	CC002	M	Y	6.8	2.6	3.6	1.9	1.9
2	CC002	F	Y	2.9	4.6	4.8	4.7	5.2
2	CC002	M	Y	5.5	2.9	4.6	2.2	2.8
0	CC005	F	Y	3.7	3.4	4	3.4	4
0	CC005	M	Y	3.7	3.1	4.2	3.6	4.2
0	CC005	M	Y	3.9	3.7	4	3.7	4
1	CC005	F	Y	4.2	2.1	4	2.6	3.6
1	CC005	M	Y	9.3	4.1	2.8	2.3	1.7
1	CC005	F	Y	2.9	5.3	5.2	5.1	5.3
1	CC005	M	Y	4.6	2.7	2.4	4.2	3.4
2	CC005	F	Y	6.2	2.2	2.4	2.3	2.5
2	CC005	M	Y	3.5	3.2	4.7	4.2	4.4
2	CC005	M	Y	6.1	3.7	3.6	2.6	2.5
2	CC005	M	Y	3.5	3.2	4.7	4.2	4.4
2	CC005	F	Y	3.9	4.6	6	5.4	4
0	CC006	F	Y	4.2	3	3.7	2.9	3.6
0	CC006	M	Y	9.5	2.9	2.5	1.9	1.7
0	CC006	F	Y	4.9	3	3.1	2.9	3.1
1	CC006	F	Y	7	2	2	2	2.2
1	CC006	M	Y	9.4	1.7	1.5	1.8	1.7
1	CC006	F	Y	3.1	5	5	5	4.8
1	CC006	M	Y	2.9	4.5	5.2	4.9	5.3
2	CC006	F	Y	5.2	3	3	3.1	3

Trial#	Strain	Sex	Inf?	StrideFrequency_RH	StrideLength_LF	StrideLength_LH	StrideLength_RF	StrideLength_RH
2	CC006	M	Y	6.7	2	1.8	2.9	2.3
0	CC011	F	Y	4.3	2.1	2.8	1.8	3.7
0	CC011	M	Y	5.7	3	2.8	2.6	2.7
0	CC011	F	Y	4.8	2.9	3.2	3.2	3.2
0	CC011	F	Y	7.7	3.2	2.3	3	2
1	CC011	F	Y	3.9	3.6	4.1	3.6	4
1	CC011	M	Y	3.8	4.1	4.5	4.3	4.1
1	CC011	F	Y	3.5	4.4	4.4	4.3	4.3
1	CC011	M	Y	3.7	3.6	3.9	3.7	4.2
2	CC011	F	Y	3.5	4.2	4.3	4.1	4.3
2	CC011	M	Y	3	3.2	3.9	2.3	5.2
2	CC011	F	Y	3.3	4.9	4.6	4.8	4.7
2	CC011	M	Y	2.9	4.9	5.3	5.2	5.1
0	CC012	M	Y	3.5	5	5	5.1	4.4
0	CC012	M	Y	4.3	5	3.6	3.1	3.6
0	CC012	M	Y	3.9	3.9	4	4	3.9
0	CC012	M	Y	3.8	3.9	4.1	4	4
0	CC012	M	Y	3.4	5.7	5.7	4.9	4.4
0	CC012	F	Y	3.8	2.7	3.6	3	4
0	CC012	F	Y	3.7	4.2	4.4	4.2	4.3
0	CC012	F	Y	4.3	2.5	3.1	4.1	3.6
0	CC012	F	Y	5.1	3	2.8	2.5	3
0	CC012	F	Y	8.1	1.8	2.3	2	1.8
2	CC012	M	Y	3.1	4.9	5.3	4.4	5
2	CC012	M	Y	2.7	5.5	5.7	5.8	5.7
2	CC012	M	Y	2.9	5	5	5.1	5.3
2	CC012	M	Y	2.4	5	5	5.2	5
2	CC012	M	Y	2.8	5.2	5.6	4.9	5.5
2	CC012	F	Y	2.9	4.4	5.6	5.7	5.3
2	CC012	F	Y	3.8	3.5	3.8	4	3.9
2	CC012	F	Y	5.1	3	4.2	2.9	3
2	CC012	F	Y	2.6	4.8	6.3	3.6	5.6
2	CC012	F	Y	3.5	3.7	4.6	4	4.4
1	CC012XCC032	F	Y	10.3	2.2	2	2.2	1.5
1	CC012xCC032	F	Y	11.5	1.5	1.6	1.9	1.4
1	CC012XCC032	M	Y	3.1	3.8	4.7	3.9	4.9
1	CC012xCC032	M	Y	3.7	3.3	3.8	3.7	4.2
1	CC012XCC032	M	Y	8.9	1.7	2.4	2.1	1.8
1	CC012xCC032	M	Y	9.5	2.3	2.2	1.8	1.7
2	CC012XCC032	F	Y	2.6	4	5	3.3	5.9
2	CC012XCC032	F	Y	2.3	5.6	5.5	6.6	6.4
2	CC012XCC032	M	Y	4.3	3.1	5.2	2.6	3.6
2	CC012XCC032	M	Y	2.7	2.9	4.7	3.6	5.5
2	CC012XCC032	M	Y	3.3	4.5	4.6	4.7	4.7
2	CC012XCC032	M	Y	2.4	4.3	4.8	4.1	6.2
1	CC013xCC041	F	Y	7.4	2.5	2.6	3.5	2.1
1	CC013xCC041	F	Y	12.2	1.2	1.1	1.7	1.3
1	CC013xCC041	F	Y	6.7	1.9	2.2	2	2.3

Trial#	Strain	Sex	Inf?	StrideFrequency_RH	StrideLength_LF	StrideLength_LH	StrideLength_RF	StrideLength_RH
1	CC013xCC041	M	Y	4.9	2.5	2.8	2.5	3.1
1	CC013xCC041	M	Y	8.7	3.9	3.8	2.1	1.8
2	CC013XCC041	F	Y	3.5	4.4	4.4	4.3	4.4
2	CC013XCC041	F	Y	2.5	3.6	4.1	3.9	6
2	CC013XCC041	F	Y	3	4.3	4.9	5.1	5.3
2	CC013XCC041	M	Y	4.4	2.6	4.1	3.2	3.4
2	CC013XCC041	M	Y	10.7	1.6	2.1	1.6	1.5
0	CC015	F	Y	4.2	3.6	3.2	3.5	3.6
0	CC015	M	Y	3.6	4.3	4.3	4.3	4.3
0	CC015	M	Y	4.7	3.8	3.7	3.5	3.3
1	CC015	F	Y	4	4.2	4.2	4.2	3.8
1	CC015	M	Y	7.9	1.9	1.7	2.2	1.9
1	CC015	M	Y	8.3	2.3	1.7	2.6	1.9
1	CC015	M	Y	5.1	3	3.7	2.6	3
2	CC015	F	Y	3.7	3.7	4.1	4	4.1
0	CC017	F	Y	8	2.4	2.4	2.1	2
0	CC017	M	Y	5.3	3.2	2.1	3.3	2.9
0	CC017	F	Y	4.3	2.9	4	2.7	3.6
0	CC017	M	Y	4.1	3.3	4.3	2.3	3.7
1	CC017	M	Y	4.4	2.8	3.1	2.8	3.5
1	CC017	F	Y	6	2.3	2.8	3	2.6
0	CC023	M	Y	3.6	4.1	4.2	4.4	4.4
0	CC023	F	Y	7.5	3.4	4.5	3.7	2
0	CC023	F	Y	6.9	2.5	2.2	1.9	2.3
0	CC023	M	Y	4.2	1.7	3.5	1.9	3.6
1	CC023	M	Y	5	1.6	2.4	1.7	3.1
1	CC023	F	Y	4.2	2.1	2.7	1.9	3.6
1	CC023	M	Y	5.2	2.6	4.1	2.1	2.9
1	CC023	M	Y	3.5	3.9	3.8	3.5	4.3
2	CC023	M	Y	2.9	5.3	4.4	5.2	5.3
2	CC023	F	Y	3	4.1	4.6	3.2	5
2	CC023	M	Y	4.4	2.3	5.2	2.9	3.5
0	CC025	M	Y	4.9	3.1	3.2	3.2	3.1
0	CC025	F	Y	4.2	3.2	3.6	3.8	3.7
0	CC025	M	Y	3.3	4.1	4	3.4	4.6
1	CC025	M	Y	4.9	3.1	3.4	3.2	3.2
1	CC025	F	Y	5.1	2.8	2.7	2.9	3
1	CC025	F	Y	5	3.2	3.1	2.9	3.1
1	CC025	F	Y	4.4	3.7	4.4	3.4	3.5
2	CC025	F	Y	3	4.6	5.1	5.2	4.9
2	CC025	M	Y	2.6	5.7	5.9	5.8	5.9
0	CC027	F	Y	4.5	3.5	3.3	2.9	3.4
0	CC027	M	Y	2.7	2.7	3.9	2.4	5.4
1	CC027	M	Y	7.4	2.7	2.9	3	2.1
1	CC027	M	Y	7.4	2.7	2.9	3	2.1
1	CC027	F	Y	11.1	1.9	2.1	1.9	1.4
1	CC027	F	Y	6.3	3.6	3.9	2.8	2.4
1	CC027	M	Y	2.5	4.4	6.3	6.2	6.5

Trial#	Strain	Sex	Inf?	StrideFrequency_RH	StrideLength_LF	StrideLength_LH	StrideLength_RF	StrideLength_RH
1	CC027	M	Y	3.2	4.9	5	4.9	4.8
2	CC027	F	Y	3	4.1	4.6	3.2	5
2	CC027	M	Y	2.8	3.8	3.1	5.2	5.4
2	CC027	F	Y	5.2	2.9	3.5	2.3	3
2	CC027	M	Y	2.8	4.5	5.4	5.5	5.4
1	CC032XCC013	M	Y	4.3	3.6	3.8	2.9	3.5
1	CC032XCC013	M	Y	3.3	4.2	4.6	4.4	4.6
1	CC032XCC013	M	Y	6.3	1.7	2.2	2.1	2.4
1	CC032XCC013	F	Y	6.2	1.9	2.4	3	2.5
1	CC032XCC013	F	Y	5	2.4	3.4	2.7	3.1
1	CC032XCC013	F	Y	3.6	3.3	4.4	4.1	4.2
1	CC032XCC013	F	Y	3.5	5	5.1	4.1	4.3
1	CC032XCC013	F	Y	3.7	3.7	3.3	4.5	4.1
2	CC032XCC013	M	Y	3.8	3.6	3.8	3.3	3.9
2	CC032XCC013	M	Y	6	3	3.5	2.2	2.6
2	CC032XCC013	M	Y	4.5	2.2	4.1	2.2	3.3
2	CC032XCC013	M	Y	4.5	4.1	4.8	2.2	3.4
2	CC032XCC013	F	Y	3.3	4.6	4.7	4.5	4.6
2	CC032XCC013	F	Y	3.4	3.3	4.3	3.9	4.5
2	CC032XCC013	F	Y	3.2	4.1	4.2	3	4.6
2	CC032XCC013	F	Y	2.8	4.5	5.1	4.4	5.2
0	CC037	F	Y	3.4	2.8	3.6	3	4.5
0	CC037	M	Y	4.1	2.1	3	2.2	3.8
0	CC037	M	Y	3.3	2.2	4.6	3.1	4.5
0	CC037	M	Y	7.1	3.4	4.1	2.6	2.4
1	CC037	F	Y	3.1	4	5.1	4.7	4.9
1	CC037	M	Y	3.4	4.2	4.6	4.7	4.5
1	CC037	M	Y	3	3.8	5.2	3.7	5.1
1	CC037	M	Y	3.8	5.1	5.2	4.6	4
2	CC037	M	Y	3.2	3.7	4.7	3.2	4.5
2	CC037	M	Y	2.6	5.8	5.8	5.6	5.8
1	CC041XCC012	M	Y	3.4	3.9	3.2	3.7	4.6
1	CC041XCC012	M	Y	3.7	3.1	4.3	4	4.1
1	CC041XCC012	M	Y	6.4	3.7	2.8	3	2.4
1	CC041XCC012	M	Y	5	2.4	3.2	3.6	3
1	CC041XCC012	M	Y	3.1	4.3	4.8	4.2	4.8
1	CC041XCC012	F	Y	3.3	3.6	5.3	3.7	4.6
1	CC041XCC012	F	Y	3.8	4.6	4.6	2.9	4
1	CC041XCC012	F	Y	3.6	4.7	5.2	3.1	4.3
1	CC041XCC012	F	Y	3.2	3.7	5	4.1	4.9
1	CC041XCC012	F	Y	3.7	4.4	4.7	3.4	4.2
1	CC041XCC012	F	Y	3.5	4.2	4.6	3.5	4.4
1	CC041XCC012	F	Y	3.3	4.8	4.3	4.4	4.6
1	CC041XCC012	F	Y	3.8	4.4	4.7	3.2	4
2	CC041XCC012	M	Y	3.4	4.8	4.7	4.8	4.5
2	CC041XCC012	M	Y	2.2	6.7	6.9	5.4	7
2	CC041XCC012	F	Y	2.5	5.8	5.8	5.1	6
2	CC041XCC012	M	Y	3.4	3	3.4	3.8	4.5

Trial#	Strain	Sex	Inf?	StrideFrequency_RH	StrideLength_LF	StrideLength_LH	StrideLength_RF	StrideLength_RH
2	CC041XCC012	M	Y	3	5.2	5.2	5.2	5.1
2	CC041XCC012	M	Y	3.1	3.9	4.4	4.4	5
2	CC041XCC012	F	Y	2.4	6.1	6.3	6.4	6.4
2	CC041XCC012	F	Y	3	4.4	5	5	5
2	CC041XCC012	F	Y	3	5.3	5.5	4.9	5
2	CC041XCC012	F	Y	3	5.1	5.1	5.2	5.1
2	CC041XCC012	F	Y	2.7	4.8	5.7	4.9	5.6
2	CC041XCC012	F	Y	2.8	5.3	5.6	5.5	5.4
2	CC041XCC012	F	Y	2.9	5.2	5.3	4.8	5.3
0	CC051	F	Y	3.7	3.7	4.2	3.6	4.2
0	CC051	F	Y	4.1	3	4	2.9	3.7
0	CC051	F	Y	3.7	3.7	3.8	3.8	4.1
0	CC051	M	Y	4.4	3.4	3.5	3.1	3.4
1	CC051	F	Y	3.4	3.8	4.5	4.5	4.4
1	CC051	F	Y	3.6	4.2	4.3	3.5	4.2
1	CC051	F	Y	3.6	3.9	5	4.3	4.2
0	CC057	F	Y	3.9	3.9	3.9	4	3.9
0	CC057	F	Y	4.6	3	3.3	3.6	3.3
0	CC057	F	Y	6.9	2.6	2.7	2.5	2.2
0	CC057	M	Y	5.6	1.8	2.8	3.6	2.8
0	CC057	M	Y	10.8	3.1	1.6	2.5	1.5
0	CC057	M	Y	8.3	2.4	2.4	1.8	1.9
1	CC057	F	Y	4.1	3.6	3.6	3.7	3.8
1	CC057	F	Y	5.9	2.4	2.6	2.6	2.6
1	CC057	F	Y	7.4	2.2	2	1.8	2.1
1	CC057	F	Y	6.1	2.5	2.4	2.4	2.6
1	CC057	M	Y	3.1	4	4.3	3.9	5
1	CC057	M	Y	3.7	4	4	3.8	4
2	CC057	F	Y	6.1	2.5	2.4	2.4	2.6
0	CC078	F	Y	5.1	3.7	3.8	3.6	3
0	CC078	F	Y	4.5	3.8	3.1	3.7	3.4
0	CC078	F	Y	4	3.9	3.8	3.8	3.9
0	CC078	M	Y	4	3.7	3.7	3.7	3.8
1	CC078	F	Y	3.6	3.7	4.2	4.2	4.2
1	CC078	F	Y	4.2	3.7	4.1	3.7	3.7
1	CC078	M	Y	4	3.8	3.6	3.6	3.8
2	CC078	F	Y	4.9	3.7	3.5	2.9	3.1
2	CC078	M	Y	3.9	3.3	4	3.4	3.9
2	CC005	M	N	7.4	2.2	2.4	3.2	2.1
2	CC005	M	Y	4.1	2.7	3.1	2.5	3.7
2	CC015	F	N	6.4	1.8	2.4	1.6	2.4
2	CC015	M	N	2.9	4.6	5.3	5.1	5.1
2	CC015	M	Y	3.5	4.4	5	4.4	4.4
2	CC015	M	Y	5.5	2.7	3.3	2.5	2.8
2	CC017	F	N	3.4	4.1	5	2.7	4.5
2	CC017	M	N	4.4	2.1	3	2	3.5
2	CC017	F	Y	3.5	3.6	4.1	5	4.3
2	CC023	F	N	3.1	4.2	4.6	5.1	4.8

Trial#	Strain	Sex	Inf?	StrideFrequency_RH	StrideLength_LF	StrideLength_LH	StrideLength_RF	StrideLength_RH

Table S3. Raw DigiGait measurement data. Far left column indicates time point at which data was measured: T0 = pre-infection, T1 = 21dpi, and T3 = 89dpi. DigiGait parameters listed across the top indicate which limb is associated with the data, where appropriate: FL for left fore limb, FR for right fore limb, HL for left hind limb, and HR for right hind limb.

Table S3

Trial#	Strain	Sex	Inf?	StrideLengthCV_LF	StrideLengthCV_LH	StrideLengthCV_RF	StrideLengthCV_RH	Swing_LF
1	CC002	F	N	57.85	52.11	41.02	40.39	0.065
1	CC025	F	N	25.41	35.97	27.5	39.43	0.065
1	CC012XCC032	F	N	33.19	13.67	48.64	32.81	0.098
2	CC012XCC032	F	N	38.65	10.93	3.35	8.86	0.113
1	CC012XCC032	M	N	33.18	5.28	31.08	7.11	0.075
2	CC012XCC032	M	N	16.79	17.23	10.76	19.98	0.112
1	CC013xCC041	F	N	38.72	67.38	52.31	51.16	0.073
2	CC013XCC041	F	N	11.63	7.83	22.36	15.43	0.077
1	CC013xCC041	M	N	45.14	54.8	52.37	41.18	0.048
2	CC013XCC041	M	N	30.45	17.22	38.26	20.84	0.078
1	CC032XCC013	F	N	36.96	42.32	48.43	63.06	0.029
2	CC032XCC013	F	N	46.23	41.18	40.48	26.86	0.07
1	CC041XCC012	F	N	60.61	63.61	59.66	57.83	0.043
1	CC041XCC012	F	N	57.49	70.35	70.43	79.95	0.033
2	CC041XCC012	F	N	53.62	14.3	66.44	48.67	0.081
1	CC032XCC013	M	N	61.92	46.71	51.3	59.97	0.032
2	CC032XCC013	M	N	40.21	35.41	48.64	53.68	0.067
1	CC041XCC012	M	N	38.09	27.97	55.07	46.63	0.09
2	CC041XCC012	M	N	31.48	17.61	45.73	9.34	0.087
0	CC012	F	N	45.18	39.33	48.33	35.63	0.067
2	CC012	F	N	29.67	10.94	37.97	41.71	0.135
0	CC012	M	N	52.34	53.67	55.28	45.49	0.092
2	CC012	M	N	76.96	25.58	57.86	26.36	0.047
0	CC057	F	N	33.81	9.76	38.42	62.77	0.081
1	CC057	F	N	33.15	10.15	31.93	20.52	0.083
0	CC057	M	N	55.83	24.04	15.7	39.86	0.052
1	CC057	M	N	13.97	8.03	16.31	7.81	0.107
0	CC078	F	N	9.73	5.51	10.32	20.08	0.099
1	CC078	F	N	28.82	17.37	43.19	10.92	0.097
2	CC078	F	N	63.11	47.56	57.36	47.23	0.058
0	CC078	M	N	20	14.94	18.72	18.68	0.1
2	CC078	M	N	38.6	24.14	50.83	36.19	0.097
0	CC002	F	N	53.66	56.54	56.91	60.89	0.065
1	CC002	F	N	57.85	52.11	41.02	40.39	0.065
0	CC002	M	N	53.45	51.48	78.77	74.31	0.052
1	CC002	M	N	54.22	69.38	66.13	40.51	0.039
2	CC002	M	N	32.72	10.99	42.11	49.85	0.121
0	CC006	F	N	15.91	14.19	17.25	14.2	0.116
1	CC006	F	N	53.34	55.86	38.1	21.63	0.065
2	CC006	F	N	51.17	38.53	48.56	63.07	0.043
0	CC006	M	N	51.96	58.52	49.13	53.27	0.047
0	CC023	F	N	43.67	62.81	46.74	25.86	0.081
1	CC023	F	N	63.72	54.75	65.06	44.96	0.07
2	CC023	F	N	51.9	49.56	70.09	41.85	0.116
0	CC023	M	N	19.75	13.26	13.09	40.3	0.096
1	CC023	M	N	29.34	16.46	17.38	17.65	0.098
0	CC027	F	N	55.03	70.72	48.04	79.84	0.117

Trial#	Strain	Sex	Inf?	StrideLengthCV_LF	StrideLengthCV_LH	StrideLengthCV_RF	StrideLengthCV_RH	Swing_LF
1	CC027	F	N	68.07	62.23	71.32	56.24	0.071
2	CC027	F	N	32.79	45.44	41.3	49.14	0.101
0	CC027	M	N	39.43	49.97	47.54	92.09	0.094
1	CC027	M	N	27.57	61.57	64.32	59.04	0.054
1	CC027	M	N	48.73	59.26	112.07	56.43	0.048
2	CC027	M	N	21.9	10.64	14.77	19.36	0.105
0	CC005	F	N	48.02	11.19	20.04	9.76	0.07
1	CC005	F	N	49.53	34.54	59.02	30.52	0.063
2	CC005	F	N	52.17	50.59	86.58	57.9	0.039
0	CC011	F	N	38.35	53.81	47.52	33.1	0.062
1	CC011	F	N	48.06	23.65	64.96	33.01	0.088
2	CC011	F	N	44.86	14.06	36.29	14.28	0.1
1	CC017	F	N	62.35	42.03	54.7	45.41	0.05
0	CC005	M	N	10.98	7.64	23.04	7	0.095
1	CC005	M	N	57.86	72.19	66.99	41.34	0.06
2	CC005	M	N	65.5	71.77	64.61	69.75	0.049
0	CC011	M	N	8.49	18.13	17.11	30.21	0.077
1	CC011	M	N	18.2	13.96	26.78	16.5	0.104
2	CC011	M	N	65.55	58.85	71.95	66.74	0.066
0	CC017	M	N	70.68	69.43	66.55	58.96	0.084
1	CC017	M	N	29	24.7	41.47	64.18	0.064
1	CC006	F	N	54.46	49.64	49.56	52.57	0.045
0	CC037	F	N	41.18	29.93	31.61	17.66	0.11
0	CC051	F	N	52.83	21.19	36.85	26.84	0.071
1	CC051	F	N	44.97	45.06	43.02	38.31	0.055
1	CC006	M	N	53.14	29.64	34.47	23.8	0.097
0	CC037	M	N	52.78	25.73	34.39	30.33	0.092
1	CC037	M	N	37.03	30.75	26.13	39.12	0.095
0	CC005	F	N	49.81	31.17	41.53	31.56	0.073
1	CC005	F	N	13.51	39.11	41.61	55.57	0.077
2	CC005	F	N	11.22	45.68	31.14	8.26	0.123
0	CC011	F	N	13.52	21	19.7	17.74	0.077
1	CC011	F	N	22.67	13.88	18.9	27.59	0.093
2	CC011	F	N	30.68	19.62	24.09	15.13	0.069
0	CC011	M	N	35.41	21.47	25.3	11.77	0.097
1	CC011	M	N	29.1	11.63	11.52	3.69	0.116
2	CC011	M	N	23.41	18.9	32.42	5.94	0.111
1	CC037	M	N	36.87	22.83	44.55	19.23	0.089
2	CC037	M	N	35.51	28.93	46.7	22.27	0.109
1	CC051	M	N	17.14	9.77	24.8	15.57	0.105
2	CC051	M	N	41.73	16.49	57.77	25.75	0.098
0	CC027	F	N	13.63	9.03	27.4	9.72	0.15
1	CC027	F	N	19.72	47.16	34.27	44.44	0.098
2	CC027	F	N	67.23	47.95	54.42	54.76	0.066
0	CC015	M	N	31.08	16.48	13.69	16.76	0.065
1	CC015	M	N	44.78	26.58	52.86	37.71	0.108
2	CC015	M	N	13.71	18.16	32.45	14.55	0.106
0	CC027	M	N	63.16	34.23	21.77	8.43	0.081

Trial#	Strain	Sex	Inf?	StrideLengthCV_LF	StrideLengthCV_LH	StrideLengthCV_RF	StrideLengthCV_RH	Swing_LF
1	CC027	M	N	42.18	8.97	15.54	41.82	0.119
2	CC027	M	N	47.11	59.11	11.41	36.37	0.088
0	CC015	F	N	17.6	20.62	18.66	0.068	0.08
1	CC015	F	N	68.23	66.13	60.05	58.04	0.034
0	CC017	F	N	43.97	50.4	30.08	16.79	0.099
0	CC023	F	N	26.21	15.9	39.9	18.49	0.089
1	CC023	F	N	22.8	17.98	38.49	12.2	0.102
0	CC005	M	N	21.4	46.43	17.23	42.18	0.093
1	CC005	M	N	26.11	31.65	17.54	21.73	0.098
0	CC015	M	N	23.31	26.55	28.57	23.62	0.102
1	CC015	M	N	50.95	8.05	66.19	48.95	0.059
0	CC017	M	N	34.37	35.09	42.46	23.28	0.074
1	CC017	M	N	46.1	66.05	54.6	59.51	0.041
0	CC023	M	N	13.29	11.69	8.36	8.02	0.127
1	CC023	M	N	47.28	29.07	29.82	29.28	0.1
0	CC051	M	N	15.26	21.38	15.42	16.76	0.092
0	CC002	F	Y	59.46	58.34	53.83	69.59	0.061
0	CC002	M	Y	49.81	43.25	42.26	55.43	0.066
1	CC002	M	Y	35.42	51.11	47.13	85.8	0.094
1	CC002	F	Y	50.76	34.43	66.59	77.08	0.039
1	CC002	F	Y	54.09	54.64	60.44	59.53	0.04
1	CC002	M	Y	35.42	51.11	47.13	85.8	0.094
1	CC002	M	Y	75.93	64.83	63.89	53.08	0.042
1	CC002	F	Y	50.76	34.43	66.59	77.08	0.039
1	CC002	F	Y	37.25	56.25	44.06	47.91	0.048
2	CC002	M	Y	59.13	19.83	46.84	31.82	0.071
2	CC002	F	Y	25.23	26.08	22.13	13.96	0.103
2	CC002	M	Y	54.44	31.09	63.3	68.31	0.05
0	CC005	F	Y	30.38	9.26	30.7	19.24	0.096
0	CC005	M	Y	33.06	12.22	31.29	11.87	0.07
0	CC005	M	Y	27.54	17.53	34.9	17.67	0.065
1	CC005	F	Y	27.53	57.76	49.25	33.32	0.054
1	CC005	M	Y	49.77	40.02	65.85	84.77	0.075
1	CC005	F	Y	17.75	7.41	28.15	14.84	0.127
1	CC005	M	Y	60.91	74.78	32.03	35.12	0.062
2	CC005	F	Y	72.32	48.28	76.7	59.21	0.062
2	CC005	M	Y	46.48	21.78	37.86	22.87	0.068
2	CC005	M	Y	49.89	38.12	56.29	78.4	0.09
2	CC005	M	Y	46.48	21.78	37.86	22.87	0.068
2	CC005	F	Y	46.36	25.95	20.89	41	0.099
0	CC006	F	Y	39.79	34.81	51.05	36.99	0.07
0	CC006	M	Y	44.93	48.73	63.16	63.88	0.059
0	CC006	F	Y	56.28	57.85	60.22	44.43	0.068
1	CC006	F	Y	54.63	53.88	49.83	78.64	0.048
1	CC006	M	Y	66.68	75.39	56.85	75.99	0.024
1	CC006	F	Y	19.19	11.68	15.88	8.9	0.118
1	CC006	M	Y	49.68	11.1	23.45	6.08	0.11
2	CC006	F	Y	55.34	57.73	73.1	62.07	0.08

Trial#	Strain	Sex	Inf?	StrideLengthCV_LF	StrideLengthCV_LH	StrideLengthCV_RF	StrideLengthCV_RH	Swing_LF
2	CC006	M	Y	69.98	59.68	43.79	61.05	0.035
0	CC011	F	Y	50.19	47.95	67.18	7.62	0.068
0	CC011	M	Y	45.29	44.99	55.16	42.07	0.06
0	CC011	F	Y	21.34	19.11	18.28	17.85	0.074
0	CC011	F	Y	30.89	67.59	34.19	52.66	0.068
1	CC011	F	Y	36.12	17.89	26.55	12.34	0.078
1	CC011	M	Y	16.31	32.13	21.32	14.21	0.076
1	CC011	F	Y	17.93	22.11	33.61	25.58	0.087
1	CC011	M	Y	20.27	10.68	25.8	19.45	0.087
2	CC011	F	Y	14.46	18.04	20.04	19.06	0.087
2	CC011	M	Y	71.15	46.14	73.53	27.66	0.05
2	CC011	F	Y	11.95	16.56	36.11	15.49	0.085
2	CC011	M	Y	21.96	8.59	16.85	4.18	0.122
0	CC012	M	Y	9.5	9.06	14.84	23.19	0.122
0	CC012	M	Y	20.99	46.53	59.65	55.9	0.109
0	CC012	M	Y	9.38	8.95	17.5	14.25	0.09
0	CC012	M	Y	42.64	31	41.77	45.58	0.089
0	CC012	M	Y	22.66	15.54	35.6	47.29	0.119
0	CC012	F	Y	42.45	46.01	42.84	21.9	0.069
0	CC012	F	Y	28.42	27.64	39.36	26.26	0.087
0	CC012	F	Y	47.15	56.92	42.98	38.65	0.044
0	CC012	F	Y	56.72	66.96	71.27	61.31	0.086
0	CC012	F	Y	26.06	19.81	38.25	45.96	0.044
2	CC012	M	Y	11.1	8.76	34.58	23.86	0.108
2	CC012	M	Y	19.3	12.26	8.66	11.24	0.115
2	CC012	M	Y	11.8	10.2	31.53	9.9	0.12
2	CC012	M	Y	12.79	17.52	36.98	26.58	0.12
2	CC012	M	Y	16.1	20.7	16.55	14.25	0.129
2	CC012	F	Y	29.93	29.78	29.36	18.94	0.101
2	CC012	F	Y	34.39	23.69	27.64	21.7	0.092
2	CC012	F	Y	61.67	39.03	62.85	51.58	0.066
2	CC012	F	Y	47.78	15.21	42.03	11.74	0.11
2	CC012	F	Y	26.9	26.92	44.52	29.84	0.072
1	CC012XCC032	F	Y	65.36	70.23	58.91	62.62	0.032
1	CC012xCC032	F	Y	56.5	57.09	51.04	52.66	0.029
1	CC012XCC032	M	Y	50.3	22.36	38.21	15.8	0.076
1	CC012xCC032	M	Y	59.35	59.05	53.78	47.12	0.086
1	CC012XCC032	M	Y	44.71	36.51	40.84	53.39	0.049
1	CC012xCC032	M	Y	95.33	54.65	47.61	70.24	0.033
2	CC012XCC032	F	Y	46.21	27.67	45.43	3.98	0.073
2	CC012XCC032	F	Y	9.64	6.21	44.98	37.25	0.109
2	CC012XCC032	M	Y	50.31	11.87	65.93	47.02	0.067
2	CC012XCC032	M	Y	61.49	30.24	44.87	11.93	0.085
2	CC012XCC032	M	Y	20.12	9.47	16.56	25.37	0.098
2	CC012XCC032	M	Y	41.4	23.14	25.33	39.37	0.089
1	CC013xCC041	F	Y	51.25	46.8	29.8	54.06	0.051
1	CC013xCC041	F	Y	53.29	51.23	51.93	49.45	0.025
1	CC013xCC041	F	Y	62.86	57.74	50.99	56.31	0.041

Trial#	Strain	Sex	Inf?	StrideLengthCV_LF	StrideLengthCV_LH	StrideLengthCV_RF	StrideLengthCV_RH	Swing_LF
1	CC013xCC041	M	Y	37.52	54.2	44	32.64	0.045
1	CC013xCC041	M	Y	58.83	31.49	63.14	65.48	0.071
2	CC013XCC041	F	Y	19.33	6.42	17.29	11.03	0.09
2	CC013XCC041	F	Y	37.47	28.91	45.66	63.3	0.069
2	CC013XCC041	F	Y	22.64	16.85	11.75	8.99	0.098
2	CC013XCC041	M	Y	38.54	38.01	33.37	29.56	0.047
2	CC013XCC041	M	Y	57.87	80.02	48.95	45.11	0.03
0	CC015	F	Y	30.71	43.7	9.26	15.89	0.079
0	CC015	M	Y	15.99	7.77	17.36	5.94	0.095
0	CC015	M	Y	15.29	12.96	25.89	24.58	0.078
1	CC015	F	Y	20.66	11.46	11.75	34.38	0.088
1	CC015	M	Y	67.66	60.44	69.56	70.35	0.034
1	CC015	M	Y	53.59	43.78	58.19	69.39	0.036
1	CC015	M	Y	47.28	41.77	71.38	60.1	0.063
2	CC015	F	Y	33.99	12.87	17.41	5.95	0.07
0	CC017	F	Y	14.74	33.76	46.06	52.4	0.067
0	CC017	M	Y	17.99	50.85	15.62	33.34	0.065
0	CC017	F	Y	57.17	18.21	52.45	50.45	0.065
0	CC017	M	Y	39.16	29.72	75.71	24.65	0.078
1	CC017	M	Y	46.21	74.32	67.77	79.46	0.077
1	CC017	F	Y	45.29	60.25	63.56	62.12	0.055
0	CC023	M	Y	8.57	5.95	7.26	9.19	0.114
0	CC023	F	Y	58.63	10.7	53.38	74.43	0.086
0	CC023	F	Y	47.79	58.61	56.22	58.02	0.066
0	CC023	M	Y	47.1	19.39	74.96	18.61	0.074
1	CC023	M	Y	52.71	60.11	58.92	76.33	0.031
1	CC023	F	Y	91.82	52.17	61.79	83.51	0.056
1	CC023	M	Y	57.82	42.73	72.55	50.9	0.045
1	CC023	M	Y	37.03	7.33	39.82	33.28	0.086
2	CC023	M	Y	16.72	40.71	14.21	6.82	0.064
2	CC023	F	Y	54.99	38.8	52.64	57.19	0.078
2	CC023	M	Y	59.56	24.64	60.15	67.67	0.059
0	CC025	M	Y	31.89	39.15	28.18	25.76	0.067
0	CC025	F	Y	35.09	40.72	23.22	22.69	0.084
0	CC025	M	Y	26.59	7.29	36.52	21.6	0.084
1	CC025	M	Y	32.96	41.21	31.17	25.91	0.067
1	CC025	F	Y	33.45	41.41	28.49	39.44	0.09
1	CC025	F	Y	51.89	30.46	24.17	22.6	0.078
1	CC025	F	Y	36.02	15.61	38.61	35.43	0.083
2	CC025	F	Y	35.39	28.58	15.8	20.37	0.09
2	CC025	M	Y	18.25	10.12	14.02	12.41	0.128
0	CC027	F	Y	13.81	39.69	22.92	21.1	0.079
0	CC027	M	Y	62.4	26.14	65.37	34.32	0.068
1	CC027	M	Y	126.58	48.61	53.51	47.83	0.018
1	CC027	M	Y	126.58	48.61	53.51	47.83	0.018
1	CC027	F	Y	52.06	55.47	52.53	47.95	0.06
1	CC027	F	Y	35.45	44.26	47.68	58.79	0.096
1	CC027	M	Y	9.5	6.41	10.2	9.73	0.08

Trial#	Strain	Sex	Inf?	StrideLengthCV_LF	StrideLengthCV_LH	StrideLengthCV_RF	StrideLengthCV_RH	Swing_LF
1	CC027	M	Y	10.72	13.65	24.9	15.18	0.094
2	CC027	F	Y	54.99	38.8	52.64	57.19	0.078
2	CC027	M	Y	51.88	55.11	23.6	23.08	0.091
2	CC027	F	Y	47.25	36.12	53.59	50.42	0.059
2	CC027	M	Y	51.12	14.78	14.28	8.77	0.107
1	CC032XCC013	M	Y	54.27	41.32	61.54	58.19	0.076
1	CC032XCC013	M	Y	37.21	15.1	23.54	21.56	0.077
1	CC032XCC013	M	Y	47.25	31.46	53.19	34.5	0.05
1	CC032XCC013	F	Y	38.02	51.43	43.96	62.83	0.046
1	CC032XCC013	F	Y	57.77	28.3	65.29	38.65	0.044
1	CC032XCC013	F	Y	47.01	30.97	16.9	11.66	0.068
1	CC032XCC013	F	Y	36.66	20.97	18.42	12.87	0.086
1	CC032XCC013	F	Y	19.25	28.43	18.19	13.51	0.076
2	CC032XCC013	M	Y	21.26	10.95	43.81	23.55	0.061
2	CC032XCC013	M	Y	59.08	50.26	56.96	32.84	0.068
2	CC032XCC013	M	Y	76.36	46.1	80.55	42.84	0.075
2	CC032XCC013	M	Y	29.2	10.51	62.3	49.7	0.088
2	CC032XCC013	F	Y	18.95	15	22.28	10.39	0.089
2	CC032XCC013	F	Y	33.91	15.01	31.35	11.82	0.071
2	CC032XCC013	F	Y	20.81	24.81	39.81	12.27	0.069
2	CC032XCC013	F	Y	26.71	7.76	21.67	23.71	0.095
0	CC037	F	Y	48.14	52.35	66.27	36.93	0.066
0	CC037	M	Y	51.27	48.03	42.97	48.43	0.061
0	CC037	M	Y	71.17	5.81	32.69	6.28	0.051
0	CC037	M	Y	28.28	61.02	37.51	38.75	0.096
1	CC037	F	Y	45.88	53.75	30.84	34.62	0.085
1	CC037	M	Y	28.73	14.36	21.58	9.82	0.098
1	CC037	M	Y	36.4	20.15	44.84	29.54	0.077
1	CC037	M	Y	20.74	11.73	14.74	52.11	0.114
2	CC037	M	Y	44.43	9.28	57.63	21.99	0.075
2	CC037	M	Y	13.43	20.58	18.22	7.09	0.13
1	CC041XCC012	M	Y	43.69	53.2	49.58	46.61	0.061
1	CC041XCC012	M	Y	65.46	5.94	19.31	14.38	0.084
1	CC041XCC012	M	Y	27.37	50.26	59.94	71.64	0.062
1	CC041XCC012	M	Y	51.45	44.79	59.68	55.3	0.04
1	CC041XCC012	M	Y	22.99	9.28	45.45	19.85	0.085
1	CC041XCC012	F	Y	37.25	19.09	66.17	29.64	0.065
1	CC041XCC012	F	Y	27.64	10.1	52.12	41.16	0.101
1	CC041XCC012	F	Y	26.02	12.12	38.43	34.6	0.089
1	CC041XCC012	F	Y	59.39	16.5	38.31	17.74	0.068
1	CC041XCC012	F	Y	27.84	25.69	60.83	41.87	0.075
1	CC041XCC012	F	Y	38.02	28.1	44.49	28.44	0.093
1	CC041XCC012	F	Y	16.43	11.45	12.52	21.61	0.126
1	CC041XCC012	F	Y	7.27	54.36	82.1	53.28	0.064
2	CC041XCC012	M	Y	25.13	10.23	8.87	31.39	0.089
2	CC041XCC012	M	Y	12.24	22.89	31.87	18.2	0.159
2	CC041XCC012	F	Y	9.83	9.87	34.59	8.37	0.144
2	CC041XCC012	M	Y	45.01	48.08	25.49	8.72	0.047

Trial#	Strain	Sex	Inf?	StrideLengthCV_LF	StrideLengthCV_LH	StrideLengthCV_RF	StrideLengthCV_RH	Swing_LF
2	CC041XCC012	M	Y	7.71	12.69	18.74	24.18	0.113
2	CC041XCC012	M	Y	23.88	29.71	43.74	18.59	0.077
2	CC041XCC012	F	Y	11.9	13.34	15.62	11.15	0.126
2	CC041XCC012	F	Y	39.41	33.25	39.23	40.27	0.08
2	CC041XCC012	F	Y	19.22	9.18	33.07	13.44	0.105
2	CC041XCC012	F	Y	18.96	13.96	18.01	12.74	0.109
2	CC041XCC012	F	Y	15.76	23.46	33.66	22.38	0.096
2	CC041XCC012	F	Y	22.5	6.97	23.88	10.79	0.101
2	CC041XCC012	F	Y	19.65	18.17	31.31	19.52	0.12
0	CC051	F	Y	31.43	21.95	34.73	28.11	0.084
0	CC051	F	Y	45.9	44.4	64.99	41.16	0.062
0	CC051	F	Y	38.66	37.74	39.72	30.46	0.087
0	CC051	M	Y	36.89	11.89	20.07	18.64	0.077
1	CC051	F	Y	27.47	17.69	19.12	6.44	0.086
1	CC051	F	Y	15.9	17.16	41.55	12.2	0.078
1	CC051	F	Y	40.45	13.36	27.98	21.29	0.088
0	CC057	F	Y	28.46	17.23	25.97	12.36	0.075
0	CC057	F	Y	42.15	38.22	38.61	44.27	0.068
0	CC057	F	Y	58.77	38.69	59.09	46.04	0.046
0	CC057	M	Y	50.42	48.54	46.7	52.18	0.03
0	CC057	M	Y	62.97	46.86	57.65	57.24	0.047
0	CC057	M	Y	57.41	64.4	55.64	47.81	0.039
1	CC057	F	Y	27.66	7.49	9.88	5.04	0.053
1	CC057	F	Y	45.4	39.11	45.35	61.66	0.049
1	CC057	F	Y	64.66	64.57	60.94	59.09	0.039
1	CC057	F	Y	64.05	67.81	68.36	63.07	0.042
1	CC057	M	Y	60.04	22.3	53.75	8.64	0.076
1	CC057	M	Y	10.56	24.09	14.88	23.08	0.061
2	CC057	F	Y	64.05	67.81	68.36	63.07	0.042
0	CC078	F	Y	10.21	5.33	27.29	41.93	0.099
0	CC078	F	Y	16.95	30.58	19.13	35.68	0.086
0	CC078	F	Y	13.5	8.4	9.57	9.78	0.093
0	CC078	M	Y	33.1	15	20.34	12.69	0.078
1	CC078	F	Y	26.92	8.64	8.91	7.13	0.085
1	CC078	F	Y	39.85	11.07	34.78	36.23	0.066
1	CC078	M	Y	25.74	16.44	28.72	21.1	0.093
2	CC078	F	Y	46.07	57.69	52.58	68.05	0.079
2	CC078	M	Y	42.16	34.22	32.76	28.01	0.084
2	CC005	M	N	77.66	59.7	51.85	46.58	0.053
2	CC005	M	Y	65.46	73.43	78.84	63.36	0.092
2	CC015	F	N	45.75	69.91	57.06	46.31	0.037
2	CC015	M	N	29.28	18	26.29	10.45	0.1
2	CC015	M	Y	30.13	20.77	42.83	14.44	0.074
2	CC015	M	Y	47.83	35.27	45.72	49.93	0.043
2	CC017	F	N	34.92	14.7	66	43.7	0.084
2	CC017	M	N	50.56	45.15	54.5	44.78	0.064
2	CC017	F	Y	78.19	60.05	52.98	76.4	0.06
2	CC023	F	N	31.23	31.66	23.96	25.17	0.09

Trial#	Strain	Sex	Inf?	StrideLengthCV_LF	StrideLengthCV_LH	StrideLengthCV_RF	StrideLengthCV_RH	Swing_LF

Table S3. Raw DigiGait measurement data. Far left column indicates time point at which data was measured: T0 = pre-infection, T1 = 21dpi, and T3 = 89dpi. DigiGait parameters listed across the top indicate which limb is associated with the data, where appropriate: FL for left fore limb, FR for right fore limb, HL for left hind limb, and HR for right hind limb.

Table S3

Trial#	Strain	Sex	Inf?	Swing_LH	Swing_RF	Swing_RH	SwingDurationCV_LF	SwingDurationCV_LH
1	CC002	F	N	0.047	0.081	0.062	50.79	67.31
1	CC025	F	N	0.054	0.066	0.053	25.77	31.99
1	CC012XCC032	F	N	0.096	0.073	0.091	45.11	28.86
2	CC012XCC032	F	N	0.132	0.134	0.1	45.65	4.91
1	CC012XCC032	M	N	0.05	0.076	0.055	33.68	9.51
2	CC012XCC032	M	N	0.051	0.113	0.045	11.07	19.57
1	CC013xCC041	F	N	0.052	0.06	0.049	37.22	90.03
2	CC013XCC041	F	N	0.087	0.082	0.075	11.78	31.3
1	CC013xCC041	M	N	0.049	0.047	0.025	66.31	73.43
2	CC013XCC041	M	N	0.067	0.077	0.072	13.35	22.1
1	CC032XCC013	F	N	0.062	0.031	0.028	111.08	66.23
2	CC032XCC013	F	N	0.054	0.085	0.067	58.16	41.3
1	CC041XCC012	F	N	0.057	0.072	0.059	55.36	73.57
1	CC041XCC012	F	N	0.039	0.045	0.053	65.75	68.45
2	CC041XCC012	F	N	0.075	0.08	0.065	42.05	29.38
1	CC032XCC013	M	N	0.057	0.043	0.05	111.16	56.49
2	CC032XCC013	M	N	0.067	0.045	0.06	35.87	19.58
1	CC041XCC012	M	N	0.084	0.062	0.063	37.7	33.13
2	CC041XCC012	M	N	0.092	0.091	0.12	46.6	44.37
0	CC012	F	N	0.074	0.065	0.071	60.7	51.98
2	CC012	F	N	0.123	0.101	0.133	21.12	40.03
0	CC012	M	N	0.052	0.101	0.062	62.39	72.79
2	CC012	M	N	0.068	0.049	0.059	69.61	56.92
0	CC057	F	N	0.076	0.07	0.052	33.74	22.51
1	CC057	F	N	0.077	0.081	0.077	26.94	7.45
0	CC057	M	N	0.077	0.075	0.061	57.76	43.28
1	CC057	M	N	0.1	0.092	0.092	15.47	15.73
0	CC078	F	N	0.075	0.094	0.078	12.07	12.62
1	CC078	F	N	0.092	0.087	0.084	9.82	9.12
2	CC078	F	N	0.043	0.055	0.068	68.47	42.24
0	CC078	M	N	0.093	0.091	0.087	10.11	9.61
2	CC078	M	N	0.079	0.083	0.081	35.14	38.6
0	CC002	F	N	0.043	0.056	0.037	67.49	59.14
1	CC002	F	N	0.047	0.081	0.062	50.79	67.31
0	CC002	M	N	0.049	0.056	0.042	74.54	62.9
1	CC002	M	N	0.042	0.03	0.073	56.04	68.04
2	CC002	M	N	0.095	0.108	0.067	30	25.88
0	CC006	F	N	0.086	0.109	0.095	22.25	11.42
1	CC006	F	N	0.068	0.073	0.094	84.45	42.38
2	CC006	F	N	0.052	0.068	0.057	89.78	47.07
0	CC006	M	N	0.039	0.067	0.044	70.77	79.55
0	CC023	F	N	0.058	0.085	0.066	37.27	71.5
1	CC023	F	N	0.08	0.083	0.083	72.11	52.6
2	CC023	F	N	0.096	0.074	0.105	22.06	29.49
0	CC023	M	N	0.075	0.104	0.086	31.17	20.8
1	CC023	M	N	0.075	0.093	0.081	39.02	21.37
0	CC027	F	N	0.111	0.056	0.076	87.62	89.36

Trial#	Strain	Sex	Inf?	Swing_LH	Swing_RF	Swing_RH	SwingDurationCV_LF	SwingDurationCV_LH
1	CC027	F	N	0.057	0.057	0.076	72.29	70.13
2	CC027	F	N	0.094	0.09	0.079	27.79	7.17
0	CC027	M	N	0.059	0.068	0.106	45.84	77.02
1	CC027	M	N	0.04	0.087	0.033	79.74	62.93
1	CC027	M	N	0.028	0.054	0.043	76.38	80.77
2	CC027	M	N	0.092	0.093	0.093	10.14	15.87
0	CC005	F	N	0.068	0.099	0.066	51.6	13.23
1	CC005	F	N	0.055	0.07	0.049	40.04	33.61
2	CC005	F	N	0.041	0.025	0.038	69.57	73.37
0	CC011	F	N	0.045	0.055	0.04	58.08	53.36
1	CC011	F	N	0.062	0.081	0.059	49.8	39.44
2	CC011	F	N	0.088	0.105	0.08	32.94	17.39
1	CC017	F	N	0.062	0.057	0.045	71.66	44.22
0	CC005	M	N	0.074	0.091	0.073	18.22	21.91
1	CC005	M	N	0.051	0.074	0.066	69.22	70.07
2	CC005	M	N	0.04	0.065	0.032	73.3	74.96
0	CC011	M	N	0.062	0.07	0.063	35.53	13.22
1	CC011	M	N	0.071	0.073	0.075	29.88	13.87
2	CC011	M	N	0.051	0.056	0.041	56.49	59.48
0	CC017	M	N	0.069	0.057	0.083	123.05	95.2
1	CC017	M	N	0.07	0.081	0.065	42.92	35.06
1	CC006	F	N	0.056	0.045	0.052	81.88	72.86
0	CC037	F	N	0.079	0.09	0.107	43.95	24.81
0	CC051	F	N	0.082	0.078	0.066	53.63	37.58
1	CC051	F	N	0.044	0.073	0.051	81.69	64.88
1	CC006	M	N	0.105	0.108	0.103	52.7	11.38
0	CC037	M	N	0.085	0.106	0.084	45.92	23.8
1	CC037	M	N	0.093	0.084	0.055	13.36	25.82
0	CC005	F	N	0.059	0.066	0.068	36.72	41.4
1	CC005	F	N	0.085	0.08	0.096	30.44	15.27
2	CC005	F	N	0.117	0.101	0.178	34.44	23.23
0	CC011	F	N	0.06	0.069	0.061	5.53	10.41
1	CC011	F	N	0.069	0.108	0.069	25.22	18.25
2	CC011	F	N	0.066	0.072	0.06	31.89	22.24
0	CC011	M	N	0.078	0.097	0.074	27.09	15.73
1	CC011	M	N	0.089	0.122	0.091	18.26	9.82
2	CC011	M	N	0.076	0.111	0.095	12.98	8.12
1	CC037	M	N	0.07	0.068	0.065	14.35	31.31
2	CC037	M	N	0.061	0.104	0.06	54.63	17.76
1	CC051	M	N	0.077	0.098	0.07	8.61	35.44
2	CC051	M	N	0.104	0.074	0.071	32.23	51.41
0	CC027	F	N	0.082	0.115	0.111	28.49	62.65
1	CC027	F	N	0.061	0.091	0.088	13.55	49.83
2	CC027	F	N	0.059	0.072	0.116	64.75	51.61
0	CC015	M	N	0.089	0.083	0.083	33.09	16.04
1	CC015	M	N	0.136	0.069	0.117	40.9	85.36
2	CC015	M	N	0.106	0.093	0.136	28.05	27.13
0	CC027	M	N	0.071	0.083	0.095	30.94	58.67

Trial#	Strain	Sex	Inf?	Swing_LH	Swing_RF	Swing_RH	SwingDurationCV_LF	SwingDurationCV_LH
1	CC027	M	N	0.092	0.121	0.079	43.21	13.88
2	CC027	M	N	0.073	0.099	0.062	43.42	59.63
0	CC015	F	N	0.103	0.064	32.27	42.85	39.75
1	CC015	F	N	0.044	0.036	0.043	89.73	100.57
0	CC017	F	N	0.1	0.087	0.106	34.64	33.72
0	CC023	F	N	0.065	0.073	0.061	65.9	27.03
1	CC023	F	N	0.08	0.084	0.101	35.01	29.54
0	CC005	M	N	0.067	0.096	0.118	23.42	49.17
1	CC005	M	N	0.083	0.081	0.194	14.88	30.14
0	CC015	M	N	0.074	0.095	0.074	8.91	13.28
1	CC015	M	N	0.079	0.039	0.042	48.5	20.84
0	CC017	M	N	0.047	0.07	0.062	30.89	37.95
1	CC017	M	N	0.037	0.038	0.037	70.78	79.58
0	CC023	M	N	0.093	0.118	0.093	15.55	10.71
1	CC023	M	N	0.089	0.103	0.075	46.18	48.79
0	CC051	M	N	0.078	0.107	0.073	26.38	27.15
0	CC002	F	Y	0.031	0.048	0.038	68.51	59.3
0	CC002	M	Y	0.088	0.057	0.082	67.14	41.99
1	CC002	M	Y	0.058	0.076	0.176	42.84	102.93
1	CC002	F	Y	0.082	0.039	0.047	69.57	103.29
1	CC002	F	Y	0.05	0.046	0.049	78.64	57.41
1	CC002	M	Y	0.058	0.076	0.176	42.84	102.93
1	CC002	M	Y	0.042	0.041	0.035	49.45	71.18
1	CC002	F	Y	0.082	0.039	0.047	69.57	103.29
1	CC002	F	Y	0.052	0.043	0.051	60.18	82.07
2	CC002	M	Y	0.097	0.054	0.041	75.9	27.94
2	CC002	F	Y	0.082	0.092	0.089	28.51	19.23
2	CC002	M	Y	0.078	0.04	0.05	78.15	41.43
0	CC005	F	Y	0.065	0.095	0.071	38.07	14.49
0	CC005	M	Y	0.077	0.086	0.067	42.96	21.75
0	CC005	M	Y	0.065	0.08	0.061	32.96	24.16
1	CC005	F	Y	0.066	0.066	0.078	58.9	61.76
1	CC005	M	Y	0.067	0.037	0.086	54.89	77.76
1	CC005	F	Y	0.121	0.105	0.191	11.74	23.4
1	CC005	M	Y	0.049	0.102	0.06	56.26	74.31
2	CC005	F	Y	0.036	0.049	0.032	53.01	49.13
2	CC005	M	Y	0.07	0.072	0.081	51.58	19.56
2	CC005	M	Y	0.049	0.061	0.095	58.13	47.31
2	CC005	M	Y	0.07	0.072	0.081	51.58	19.56
2	CC005	F	Y	0.086	0.084	0.139	46.25	51.38
0	CC006	F	Y	0.071	0.07	0.064	47.15	34.82
0	CC006	M	Y	0.069	0.056	0.081	66.2	62.5
0	CC006	F	Y	0.054	0.075	0.047	63.4	65.03
1	CC006	F	Y	0.035	0.058	0.035	70.53	76.1
1	CC006	M	Y	0.029	0.038	0.027	63.42	74.66
1	CC006	F	Y	0.104	0.134	0.097	11.44	14.7
1	CC006	M	Y	0.108	0.103	0.093	41.11	19.7
2	CC006	F	Y	0.074	0.069	0.058	57.75	55.17

Trial#	Strain	Sex	Inf?	Swing_LH	Swing_RF	Swing_RH	SwingDurationCV_LF	SwingDurationCV_LH
2	CC006	M	Y	0.039	0.048	0.059	68.89	71.98
0	CC011	F	Y	0.039	0.05	0.051	64.64	47.49
0	CC011	M	Y	0.044	0.053	0.047	38.81	75.92
0	CC011	F	Y	0.07	0.076	0.074	29.08	11.12
0	CC011	F	Y	0.119	0.085	0.079	39.62	86
1	CC011	F	Y	0.072	0.077	0.076	40.59	9.08
1	CC011	M	Y	0.073	0.061	0.072	31.15	11.03
1	CC011	F	Y	0.088	0.081	0.11	14.69	43.54
1	CC011	M	Y	0.072	0.07	0.076	33.99	12.22
2	CC011	F	Y	0.08	0.092	0.081	13.33	13.12
2	CC011	M	Y	0.053	0.037	0.066	52.95	64.02
2	CC011	F	Y	0.067	0.083	0.083	21.47	15.52
2	CC011	M	Y	0.103	0.123	0.113	18.72	31.1
0	CC012	M	Y	0.096	0.124	0.097	14.78	16.94
0	CC012	M	Y	0.088	0.075	0.08	12.73	50.22
0	CC012	M	Y	0.084	0.101	0.082	37.49	14.97
0	CC012	M	Y	0.093	0.105	0.075	48.13	40.98
0	CC012	M	Y	0.125	0.11	0.083	18.6	17
0	CC012	F	Y	0.066	0.089	0.063	48.53	41.77
0	CC012	F	Y	0.077	0.094	0.081	15.49	13.23
0	CC012	F	Y	0.075	0.09	0.068	67.52	62.29
0	CC012	F	Y	0.073	0.068	0.085	70.7	81.74
0	CC012	F	Y	0.07	0.05	0.052	73.74	46.14
2	CC012	M	Y	0.098	0.108	0.101	21.85	18.35
2	CC012	M	Y	0.138	0.147	0.13	14.97	19.76
2	CC012	M	Y	0.103	0.124	0.116	11.42	16.13
2	CC012	M	Y	0.115	0.14	0.094	11.81	35.55
2	CC012	M	Y	0.114	0.115	0.12	15.63	8.81
2	CC012	F	Y	0.088	0.149	0.084	47.01	21.19
2	CC012	F	Y	0.086	0.082	0.097	35.36	21.55
2	CC012	F	Y	0.084	0.067	0.047	77	37.44
2	CC012	F	Y	0.125	0.064	0.123	62.93	8.63
2	CC012	F	Y	0.087	0.071	0.077	40.57	26.23
1	CC012XCC032	F	Y	0.047	0.039	0.04	71.28	74.68
1	CC012xCC032	F	Y	0.042	0.043	0.043	62.72	90.1
1	CC012XCC032	M	Y	0.082	0.08	0.095	53.49	25.9
1	CC012xCC032	M	Y	0.082	0.088	0.086	62.87	60.92
1	CC012XCC032	M	Y	0.05	0.039	0.05	60.66	42.21
1	CC012xCC032	M	Y	0.039	0.021	0.03	27.15	84.01
2	CC012XCC032	F	Y	0.102	0.082	0.109	58.46	31.95
2	CC012XCC032	F	Y	0.113	0.112	0.124	10.44	12.86
2	CC012XCC032	M	Y	0.089	0.073	0.068	66.97	22.08
2	CC012XCC032	M	Y	0.104	0.082	0.137	68.93	37.29
2	CC012XCC032	M	Y	0.071	0.125	0.075	24.13	14.64
2	CC012XCC032	M	Y	0.118	0.086	0.091	44.53	14.11
1	CC013xCC041	F	Y	0.059	0.07	0.042	51.43	61.82
1	CC013xCC041	F	Y	0.026	0.035	0.034	75.57	71.15
1	CC013xCC041	F	Y	0.033	0.044	0.033	62.11	74.86

Trial#	Strain	Sex	Inf?	Swing_LH	Swing_RF	Swing_RH	SwingDurationCV_LF	SwingDurationCV_LH
1	CC013xCC041	M	Y	0.046	0.037	0.054	48.81	44.08
1	CC013xCC041	M	Y	0.056	0.043	0.034	46.25	40.38
2	CC013XCC041	F	Y	0.082	0.09	0.076	18.66	9.52
2	CC013XCC041	F	Y	0.068	0.06	0.071	43.19	22.57
2	CC013XCC041	F	Y	0.084	0.101	0.083	36.36	12.39
2	CC013XCC041	M	Y	0.053	0.052	0.052	39.41	18.65
2	CC013XCC041	M	Y	0.032	0.028	0.034	60.48	53.01
0	CC015	F	Y	0.068	0.07	0.066	32.01	63.52
0	CC015	M	Y	0.066	0.085	0.073	7.47	12.19
0	CC015	M	Y	0.06	0.073	0.056	23.19	16.67
1	CC015	F	Y	0.089	0.089	0.099	25.34	37.12
1	CC015	M	Y	0.033	0.036	0.05	58.78	67.21
1	CC015	M	Y	0.036	0.054	0.035	72.65	56.28
1	CC015	M	Y	0.058	0.051	0.046	51.96	55.18
2	CC015	F	Y	0.073	0.064	0.077	29.04	11.23
0	CC017	F	Y	0.06	0.055	0.048	43.48	33.15
0	CC017	M	Y	0.047	0.09	0.045	42.94	59.34
0	CC017	F	Y	0.058	0.053	0.05	65.93	31.88
0	CC017	M	Y	0.076	0.064	0.082	62.24	54.05
1	CC017	M	Y	0.052	0.079	0.058	76.87	48.53
1	CC017	F	Y	0.072	0.045	0.049	66.19	89.36
0	CC023	M	Y	0.081	0.113	0.106	17.61	21.64
0	CC023	F	Y	0.085	0.088	0.054	48.98	8.18
0	CC023	F	Y	0.056	0.051	0.065	57.66	73.72
0	CC023	M	Y	0.039	0.044	0.093	51.28	34.23
1	CC023	M	Y	0.027	0.041	0.03	62.53	62.22
1	CC023	F	Y	0.05	0.057	0.033	71.2	97.34
1	CC023	M	Y	0.049	0.043	0.063	71.73	61.67
1	CC023	M	Y	0.076	0.077	0.088	30.51	37.55
2	CC023	M	Y	0.137	0.084	0.119	15.75	32.49
2	CC023	F	Y	0.074	0.074	0.076	59.03	51.65
2	CC023	M	Y	0.119	0.062	0.057	67.59	19.33
0	CC025	M	Y	0.048	0.064	0.067	32.17	29.08
0	CC025	F	Y	0.046	0.078	0.07	53.64	57.73
0	CC025	M	Y	0.064	0.073	0.122	24.05	32.22
1	CC025	M	Y	0.05	0.058	0.068	32.6	24.7
1	CC025	F	Y	0.056	0.094	0.069	31.44	37.42
1	CC025	F	Y	0.057	0.066	0.064	110.84	26.06
1	CC025	F	Y	0.091	0.083	0.085	39.51	21.87
2	CC025	F	Y	0.098	0.102	0.118	41.94	27.72
2	CC025	M	Y	0.094	0.115	0.113	24.1	6.05
0	CC027	F	Y	0.059	0.075	0.09	18.86	37.08
0	CC027	M	Y	0.076	0.066	0.124	65.98	39.08
1	CC027	M	Y	0.057	0.052	0.034	33.33	111.65
1	CC027	M	Y	0.057	0.052	0.034	33.33	111.65
1	CC027	F	Y	0.058	0.056	0.033	71.79	79.32
1	CC027	F	Y	0.077	0.079	0.083	52.31	48.08
1	CC027	M	Y	0.079	0.093	0.077	38.27	24.11

Trial#	Strain	Sex	Inf?	Swing_LH	Swing_RF	Swing_RH	SwingDurationCV_LF	SwingDurationCV_LH
1	CC027	M	Y	0.08	0.099	0.086	8.93	6.2
2	CC027	F	Y	0.074	0.074	0.076	59.03	51.65
2	CC027	M	Y	0.063	0.113	0.121	42.86	66.81
2	CC027	F	Y	0.071	0.052	0.078	57.78	40.91
2	CC027	M	Y	0.09	0.12	0.085	42.75	21.91
1	CC032XCC013	M	Y	0.07	0.058	0.061	42.57	16.75
1	CC032XCC013	M	Y	0.058	0.086	0.098	32.07	39.4
1	CC032XCC013	M	Y	0.056	0.06	0.076	77.45	55.46
1	CC032XCC013	F	Y	0.053	0.046	0.048	82.33	95.27
1	CC032XCC013	F	Y	0.088	0.07	0.073	67.17	21.91
1	CC032XCC013	F	Y	0.084	0.081	0.097	67.53	40.28
1	CC032XCC013	F	Y	0.096	0.087	0.099	41.56	18.49
1	CC032XCC013	F	Y	0.052	0.129	0.073	40.78	41.97
2	CC032XCC013	M	Y	0.059	0.06	0.06	21.38	21.72
2	CC032XCC013	M	Y	0.074	0.063	0.041	57.93	52.45
2	CC032XCC013	M	Y	0.071	0.033	0.073	53.33	41.37
2	CC032XCC013	M	Y	0.113	0.066	0.057	42.24	30.83
2	CC032XCC013	F	Y	0.068	0.104	0.088	23.62	17.87
2	CC032XCC013	F	Y	0.074	0.076	0.08	45.15	37.15
2	CC032XCC013	F	Y	0.076	0.08	0.098	63.71	34.86
2	CC032XCC013	F	Y	0.073	0.101	0.11	46.04	10.07
0	CC037	F	Y	0.06	0.069	0.07	60.1	56.76
0	CC037	M	Y	0.071	0.057	0.052	70.06	54.45
0	CC037	M	Y	0.1	0.078	0.16	79.87	14.72
0	CC037	M	Y	0.167	0.068	0.101	8.15	84.61
1	CC037	F	Y	0.083	0.099	0.085	42.96	28.39
1	CC037	M	Y	0.115	0.121	0.101	35.82	31.5
1	CC037	M	Y	0.103	0.079	0.112	38.8	30.23
1	CC037	M	Y	0.096	0.11	0.079	39.34	28.71
2	CC037	M	Y	0.075	0.067	0.085	41.25	21.47
2	CC037	M	Y	0.117	0.13	0.17	15.4	19.69
1	CC041XCC012	M	Y	0.042	0.096	0.069	61.25	65.96
1	CC041XCC012	M	Y	0.064	0.068	0.065	68.88	3.25
1	CC041XCC012	M	Y	0.064	0.054	0.043	30.33	81.9
1	CC041XCC012	M	Y	0.058	0.062	0.063	48.31	49.57
1	CC041XCC012	M	Y	0.082	0.091	0.077	33.79	22.13
1	CC041XCC012	F	Y	0.083	0.079	0.082	49.91	19.72
1	CC041XCC012	F	Y	0.081	0.063	0.07	15.68	7.04
1	CC041XCC012	F	Y	0.096	0.058	0.062	25.9	15.34
1	CC041XCC012	F	Y	0.073	0.071	0.09	47.37	11.18
1	CC041XCC012	F	Y	0.086	0.08	0.083	35.19	41.92
1	CC041XCC012	F	Y	0.086	0.081	0.097	50.24	46.66
1	CC041XCC012	F	Y	0.076	0.082	0.083	30.77	9.45
1	CC041XCC012	F	Y	0.067	0.072	0.059	22.17	46.97
2	CC041XCC012	M	Y	0.078	0.091	0.083	27.65	19.17
2	CC041XCC012	M	Y	0.128	0.125	0.115	21.05	11.55
2	CC041XCC012	F	Y	0.108	0.125	0.152	9.45	10.66
2	CC041XCC012	M	Y	0.104	0.074	0.063	36.89	51.45

Trial#	Strain	Sex	Inf?	Swing_LH	Swing_RF	Swing_RH	SwingDurationCV_LF	SwingDurationCV_LH
2	CC041XCC012	M	Y	0.105	0.093	0.079	12.86	9.77
2	CC041XCC012	M	Y	0.15	0.089	0.092	53.23	35.9
2	CC041XCC012	F	Y	0.117	0.131	0.098	29.97	20.79
2	CC041XCC012	F	Y	0.067	0.091	0.083	47.46	54.65
2	CC041XCC012	F	Y	0.093	0.092	0.095	32.47	25.1
2	CC041XCC012	F	Y	0.109	0.112	0.106	17.55	8.88
2	CC041XCC012	F	Y	0.09	0.094	0.08	21.96	19.72
2	CC041XCC012	F	Y	0.083	0.089	0.071	23.69	15.75
2	CC041XCC012	F	Y	0.089	0.1	0.093	35	17.23
0	CC051	F	Y	0.067	0.078	0.07	38.46	19.24
0	CC051	F	Y	0.06	0.084	0.055	39.14	25.57
0	CC051	F	Y	0.064	0.078	0.073	46.49	43.03
0	CC051	M	Y	0.058	0.058	0.056	25.86	12.29
1	CC051	F	Y	0.08	0.103	0.068	25.44	11.13
1	CC051	F	Y	0.072	0.077	0.071	25.35	21.38
1	CC051	F	Y	0.076	0.091	0.063	43.45	15.49
0	CC057	F	Y	0.068	0.073	0.066	23.96	9.51
0	CC057	F	Y	0.066	0.067	0.069	40.61	39.11
0	CC057	F	Y	0.04	0.037	0.041	53.19	59.44
0	CC057	M	Y	0.04	0.078	0.039	73.44	55.09
0	CC057	M	Y	0.042	0.037	0.035	44.58	79.04
0	CC057	M	Y	0.042	0.038	0.03	56.31	71.17
1	CC057	F	Y	0.077	0.062	0.062	20.34	17.74
1	CC057	F	Y	0.039	0.038	0.057	56.05	75.06
1	CC057	F	Y	0.026	0.022	0.054	75.85	69.71
1	CC057	F	Y	0.047	0.052	0.05	61.85	70.64
1	CC057	M	Y	0.093	0.089	0.086	55.37	46.59
1	CC057	M	Y	0.07	0.053	0.069	26.76	16.82
2	CC057	F	Y	0.047	0.052	0.05	61.85	70.64
0	CC078	F	Y	0.089	0.087	0.064	8.45	12.79
0	CC078	F	Y	0.063	0.089	0.073	9.79	55.7
0	CC078	F	Y	0.076	0.092	0.084	10.77	9.84
0	CC078	M	Y	0.095	0.08	0.077	19.09	28.82
1	CC078	F	Y	0.073	0.066	0.088	31.58	10.48
1	CC078	F	Y	0.071	0.081	0.106	30.74	10.19
1	CC078	M	Y	0.095	0.075	0.081	16.22	21.95
2	CC078	F	Y	0.059	0.056	0.067	22.51	31.57
2	CC078	M	Y	0.091	0.066	0.074	70.41	22.26
2	CC005	M	N	0.032	0.032	0.04	66.88	60.72
2	CC005	M	Y	0.071	0.07	0.09	89.94	62.82
2	CC015	F	N	0.055	0.043	0.047	68.57	72.41
2	CC015	M	N	0.097	0.106	0.097	39.21	7.95
2	CC015	M	Y	0.072	0.098	0.072	33.16	15.93
2	CC015	M	Y	0.051	0.051	0.045	51.96	32.17
2	CC017	F	N	0.097	0.066	0.083	47.22	28.04
2	CC017	M	N	0.066	0.064	0.084	76.85	58.15
2	CC017	F	Y	0.098	0.093	0.095	41.9	47.07
2	CC023	F	N	0.094	0.112	0.129	36.1	49.28

Trial#	Strain	Sex	Inf?	Swing_LH	Swing_RF	Swing_RH	SwingDurationCV_LF	SwingDurationCV_LH

Table S3. Raw DigiGait measurement data. Far left column indicates time point at which data was measured: T0 = pre-infection, T1 = 21dpi, and T3 = 89dpi. DigiGait parameters listed across the top indicate which limb is associated with the data, where appropriate: FL for left fore limb, FR for right fore limb, HL for left hind limb, and HR for right hind limb.

Table S3

Trial#	Strain	Sex	Inf?	SwingDurationCV_RF	SwingDurationCV_RH	SWVar_LF	SWVar_LH	Tau-Propulsion_LH
1	CC002	F	N	54.46	41.52	0.91	0.91	0.3634
1	CC025	F	N	21.89	31.71	0.73	0.6	0.1871
1	CC012XCC032	F	N	60.12	41.28	0.93	0.43	0.4275
2	CC012XCC032	F	N	6.71	8.35	0.71	0.29	9.03E-02
1	CC012XCC032	M	N	31.71	21.76	0.34	0.26	0.1378
2	CC012XCC032	M	N	5.56	33.58	0.25	0.3	9.65E-02
1	CC013xCC041	F	N	52.56	76.14	0.6	0.6	3.14E-02
2	CC013XCC041	F	N	22.73	13.04	0.41	0.19	0.1262
1	CC013xCC041	M	N	70.62	79.58	1.04	0.86	0.1658
2	CC013XCC041	M	N	43.78	14.04	0.37	0.53	0.4777
1	CC032XCC013	F	N	52.72	99.21	0.79	1.09	0.1097
2	CC032XCC013	F	N	59.66	36.79	0.41	0.51	0.1663
1	CC041XCC012	F	N	77.13	59.57	0.54	0.56	0.6883
1	CC041XCC012	F	N	74.93	79.35	0.93	0.63	8.91E-02
2	CC041XCC012	F	N	92.31	47.23	0.71	0.39	0.1033
1	CC032XCC013	M	N	70.59	70.27	0.37	0.86	-Inf
2	CC032XCC013	M	N	54.69	68.99	0.93	0.49	0.3694
1	CC041XCC012	M	N	64.56	53.6	0.79	0.37	0.1255
2	CC041XCC012	M	N	41.04	16.71	0.63	0.35	0.4709
0	CC012	F	N	64.89	53.97	0.76	0.39	0.1601
2	CC012	F	N	48.05	37.6	0.61	0.19	0.1416
0	CC012	M	N	60.48	53.98	0.09	0.34	5.84E-02
2	CC012	M	N	83.65	8.91	0.87	0.35	0.4109
0	CC057	F	N	27.88	57.7	0.36	0.33	0.1011
1	CC057	F	N	28.73	19.78	0.93	0.23	0.2302
0	CC057	M	N	17.99	39.17	0.4	0.47	0.1253
1	CC057	M	N	11.48	12.49	0.41	0.17	0.1741
0	CC078	F	N	15.98	8.98	0.25	0.09	0.1279
1	CC078	F	N	33.04	15.44	0.38	0.12	0.1453
2	CC078	F	N	70.75	77	0.64	0.59	0.122
0	CC078	M	N	9.76	12.43	0.23	0.2	7.44E-02
2	CC078	M	N	52.67	40.62	0.43	0.28	0.1048
0	CC002	F	N	66.79	76.2	0.73	0.96	-5.65E-02
1	CC002	F	N	54.46	41.52	0.91	0.91	0.3634
0	CC002	M	N	46.7	84.3	0.59	0.65	0.1507
1	CC002	M	N	92.09	37.32	0.55	0.41	0.4164
2	CC002	M	N	37.06	61.36	0.13	0.25	9.47E-02
0	CC006	F	N	14.9	19.54	0.15	0.14	0.1291
1	CC006	F	N	46.6	40.62	0.5	0.45	0.1364
2	CC006	F	N	56.92	67.49	0.76	0.81	0.2719
0	CC006	M	N	58.67	78.86	0.57	0.7	-6.30E-03
0	CC023	F	N	47.07	71.37	0.75	1	0.263
1	CC023	F	N	82.04	64.86	0.84	0.81	0.8288
2	CC023	F	N	66.82	30.43	0.62	0.93	0.2176
0	CC023	M	N	10.81	52.07	0.14	0.77	0.2299
1	CC023	M	N	22.98	20.55	0.6	0.31	0.1041
0	CC027	F	N	78.54	145.16	1.4	1.46	5.55E-02

Trial#	Strain	Sex	Inf?	SwingDurationCV_RF	SwingDurationCV_RH	SWVar_LF	SWVar_LH	Tau-Propulsion_LH
1	CC027	F	N	69.42	73.91	0.84	0.66	4.30E-03
2	CC027	F	N	23.61	52.05	0.99	0.71	0.2246
0	CC027	M	N	72.69	83.2	1.01	1.07	-Inf
1	CC027	M	N	107.43	78.49	0.94	0.84	0.4526
1	CC027	M	N	67.35	68.7	0.65	1.21	1.7152
2	CC027	M	N	25.52	13.5	0.62	0.75	0.1551
0	CC005	F	N	16.82	19.02	0.83	0.23	9.83E-02
1	CC005	F	N	36.49	48.67	0.9	0.86	0.7768
2	CC005	F	N	54.72	85.29	0.76	0.89	-1.86E-02
0	CC011	F	N	50.19	37.5	0.3	0.26	0.6713
1	CC011	F	N	73.31	43.12	0.59	0.41	0.1218
2	CC011	F	N	32.05	15.79	0.56	0.32	0.1972
1	CC017	F	N	46.67	49.04	1.3	0.98	0.2767
0	CC005	M	N	27.87	8.37	0.61	0.21	0.1388
1	CC005	M	N	68.71	79.78	0.51	0.85	0.1697
2	CC005	M	N	85.19	53.09	0.59	0.77	0.1306
0	CC011	M	N	9.88	12.81	0.12	0.08	0.181
1	CC011	M	N	19.93	10.53	0.41	0.22	0.2661
2	CC011	M	N	71.63	67.07	0.58	0.64	0.7719
0	CC017	M	N	74.37	95.51	0.8	0.86	-Inf
1	CC017	M	N	41.65	62.54	1.03	1.16	0.1073
1	CC006	F	N	93.25	59.39	0.67	0.79	-7.40E-03
0	CC037	F	N	37.11	17.71	0.65	0.55	0.174
0	CC051	F	N	49.09	20.01	0.72	0.21	0.241
1	CC051	F	N	36.78	38.25	0.56	0.39	0.2658
1	CC006	M	N	49.81	46.18	0.56	0.69	4.85E-02
0	CC037	M	N	27.56	25.89	0.85	0.5	0.1312
1	CC037	M	N	35.85	37.68	0.71	0.78	0.1402
0	CC005	F	N	43.84	42.25	0.37	0.53	0.1111
1	CC005	F	N	31.76	27.18	0.7	0.19	0.1705
2	CC005	F	N	54.98	22	0.49	0.4	0.1293
0	CC011	F	N	18.68	14.11	0.32	0.51	0.284
1	CC011	F	N	22.26	21.19	0.18	0.21	0.3556
2	CC011	F	N	35.72	17.22	0.29	0.1	0.1755
0	CC011	M	N	15.87	29.62	0.73	0.48	0.1182
1	CC011	M	N	14.44	26.92	0.72	0.33	0.103
2	CC011	M	N	30.59	18.4	0.74	0.71	0.1677
1	CC037	M	N	48.7	41.31	0.77	0.35	0.1402
2	CC037	M	N	50.79	48.38	0.57	0.53	0.2437
1	CC051	M	N	27.06	27.39	0.46	0.39	9.16E-02
2	CC051	M	N	65.84	22.57	0.73	0.53	1.3748
0	CC027	F	N	19.41	17.68	0.41	0.65	0.3874
1	CC027	F	N	7.9	63.82	0.28	0.34	8.04E-02
2	CC027	F	N	49.42	89.69	0.88	0.77	0.1921
0	CC015	M	N	32.53	33.73	1.05	0.57	0.4457
1	CC015	M	N	56.98	137.81	0.91	0.77	0.1553
2	CC015	M	N	40.58	21.6	0.39	0.33	6.96E-02
0	CC027	M	N	19.59	14.09	1.04	1.02	9.76E-02

Trial#	Strain	Sex	Inf?	SwingDurationCV_RF	SwingDurationCV_RH	SWVar_LF	SWVar_LH	Tau-Propulsion_LH
1	CC027	M	N	32.12	48.48	0.54	0.79	0.1017
2	CC027	M	N	16.29	59.26	0.52	0.57	0.1212
0	CC015	F	N	33.53	0.32	0.14	0.0779	4.86E-02
1	CC015	F	N	71.79	62.28	0.99	0.86	-6.74E-02
0	CC017	F	N	38.61	51.83	0.45	0.23	0.2517
0	CC023	F	N	49.04	20.7	0.74	0.35	8.87E-02
1	CC023	F	N	38.08	23.65	0.35	0.21	8.94E-02
0	CC005	M	N	31.47	58.69	1.03	1.23	8.84E-02
1	CC005	M	N	27.85	33.15	0.46	0.16	0.1118
0	CC015	M	N	27.03	17.79	0.35	0.16	0.2576
1	CC015	M	N	61.77	73.76	0.77	0.24	9.62E-02
0	CC017	M	N	36.65	32.1	0.88	0.8	0.1959
1	CC017	M	N	108.31	71.69	1	0.7	1.7438
0	CC023	M	N	9.1	15.34	0.3	0.11	7.31E-02
1	CC023	M	N	39.57	54.8	0.69	0.37	-0.2292
0	CC051	M	N	23.57	33.63	0.39	0.36	0.1912
0	CC002	F	Y	68.15	66.17	0.67	0.54	-Inf
0	CC002	M	Y	63.34	52.51	0.73	0.52	0.2515
1	CC002	M	Y	62	100.02	0.32	0.59	7.42E-02
1	CC002	F	Y	50.85	133.51	0.51	0.65	3.65E-02
1	CC002	F	Y	78.89	69.52	0.99	0.9	-0.1372
1	CC002	M	Y	62	100.02	0.32	0.59	7.42E-02
1	CC002	M	Y	74.98	67	0.99	0.99	2.94E-02
1	CC002	F	Y	50.85	133.51	0.51	0.65	3.65E-02
1	CC002	F	Y	47.75	77.16	0.7	0.59	8.64E-02
2	CC002	M	Y	79.95	79.2	0.47	0.26	0.1301
2	CC002	F	Y	32.13	22.47	0.76	0.88	0.1822
2	CC002	M	Y	82.04	60.12	0.78	0.66	2.44E-02
0	CC005	F	Y	38.53	19.42	0.65	0.42	0.1069
0	CC005	M	Y	31.77	24.31	0.65	0.39	0.1302
0	CC005	M	Y	36.93	25.41	0.62	0.3	0.6187
1	CC005	F	Y	61.32	62.64	0.91	0.43	0.2966
1	CC005	M	Y	41.74	116.15	0.62	0.5	0.1263
1	CC005	F	Y	22.23	23.39	0.33	0.22	8.41E-02
1	CC005	M	Y	4.62	45.19	0.56	0.95	6.14E-02
2	CC005	F	Y	70.85	58.31	0.48	0.48	0.1267
2	CC005	M	Y	30.1	25	0.74	0.76	0.185
2	CC005	M	Y	76.43	143.43	0.67	0.66	-0.196
2	CC005	M	Y	30.1	25	0.74	0.76	0.185
2	CC005	F	Y	51.87	64.21	0.8	1.3	0.1123
0	CC006	F	Y	44.79	46.61	0.63	0.24	0.3824
0	CC006	M	Y	82.42	90.26	0.56	0.61	-0.1354
0	CC006	F	Y	67.95	54.35	1.1	1.24	0.1227
1	CC006	F	Y	76.35	61.74	0.71	0.6	-0.2244
1	CC006	M	Y	67.39	51.65	0.82	0.64	-0.1768
1	CC006	F	Y	13.59	17.46	0.67	0.33	0.1127
1	CC006	M	Y	23.79	28.79	0.74	0.34	0.1224
2	CC006	F	Y	60.86	68.09	0.56	0.65	0.1086

Trial#	Strain	Sex	Inf?	SwingDurationCV_RF	SwingDurationCV_RH	SWVar_LF	SWVar_LH	Tau-Propulsion_LH
2	CC006	M	Y	72.38	88.03	0.71	0.71	6.61E-02
0	CC011	F	Y	67.3	40.75	1.26	0.14	0.2338
0	CC011	M	Y	86.6	89.56	0.46	0.23	0.2449
0	CC011	F	Y	12.56	16	0.2	0.13	0.212
0	CC011	F	Y	38.41	83.96	0.27	0.37	4.47E-02
1	CC011	F	Y	23.9	9.5	0.19	0.16	0.1853
1	CC011	M	Y	17.82	16.72	0.27	0.2	0.2265
1	CC011	F	Y	17.01	15.91	0.33	0.25	0.1058
1	CC011	M	Y	47.77	17.63	0.45	0.19	0.3107
2	CC011	F	Y	15.63	10.91	0.56	0.13	0.1953
2	CC011	M	Y	83.82	28.96	0.62	0.2	-0.4282
2	CC011	F	Y	24.74	30.3	0.23	0.15	0.1629
2	CC011	M	Y	25.54	12.64	0.32	0.51	0.124
0	CC012	M	Y	17.43	36.82	0.65	0.43	9.92E-02
0	CC012	M	Y	46.51	52.26	0.5	1	9.43E-02
0	CC012	M	Y	8.18	19.16	0.77	0.34	7.54E-02
0	CC012	M	Y	77.24	43.85	0.48	0.62	2.55E-02
0	CC012	M	Y	42.09	59.36	0.39	0.35	0.1451
0	CC012	F	Y	50.16	27.6	0.48	0.43	0.1394
0	CC012	F	Y	23.74	22.56	0.57	0.22	0.2832
0	CC012	F	Y	41.11	58.21	0.55	0.65	0.1205
0	CC012	F	Y	74.26	67.98	0.61	0.53	0.2054
0	CC012	F	Y	56.98	75.38	0.62	0.76	5.65E-02
2	CC012	M	Y	49.7	32.85	0.33	0.22	0.3633
2	CC012	M	Y	12.07	5.87	0.36	0.35	0.1854
2	CC012	M	Y	12.17	16.95	0.57	0.24	0.2291
2	CC012	M	Y	22.46	29.16	0.83	0.26	0.3963
2	CC012	M	Y	34.01	15.12	0.65	0.54	0.2434
2	CC012	F	Y	62.64	17.65	0.55	0.37	0.1965
2	CC012	F	Y	24.85	12.23	0.25	0.1	0.414
2	CC012	F	Y	73.74	66.24	0.75	0.27	0.1106
2	CC012	F	Y	63.73	2.6	0.41	0.19	0.4789
2	CC012	F	Y	32.41	21.6	0.51	0.36	0.8165
1	CC012XCC032	F	Y	75.43	79.76	1.05	0.86	3.91E-02
1	CC012xCC032	F	Y	78.44	86.01	0.66	0.82	7.55E-02
1	CC012XCC032	M	Y	50.19	23.03	0.55	0.37	0.3584
1	CC012xCC032	M	Y	60.45	56.75	0.53	0.43	0.233
1	CC012XCC032	M	Y	72.01	45.91	0.8	0.98	-2.30E-02
1	CC012xCC032	M	Y	54.32	70.13	0.78	0.99	4.62E-02
2	CC012XCC032	F	Y	59.27	7.59	0.88	0.11	0.1121
2	CC012XCC032	F	Y	11.98	22.12	0.42	0.18	0.154
2	CC012XCC032	M	Y	58	42.69	0.66	0.38	0.1317
2	CC012XCC032	M	Y	61.78	15.27	0.73	0.16	0.1027
2	CC012XCC032	M	Y	30.92	24.75	0.4	0.32	0.1146
2	CC012XCC032	M	Y	47.39	23.06	0.83	0.19	0.1836
1	CC013xCC041	F	Y	22.96	69.92	0.69	0.59	-5.761
1	CC013xCC041	F	Y	61.9	69.66	0.7	0.82	5.93E-02
1	CC013xCC041	F	Y	66.46	66.09	0.61	0.47	0.1728

Trial#	Strain	Sex	Inf?	SwingDurationCV_RF	SwingDurationCV_RH	SWVar_LF	SWVar_LH	Tau-Propulsion_LH
1	CC013xCC041	M	Y	54.87	41.88	0.65	0.47	0.8822
1	CC013xCC041	M	Y	72.6	58.67	0.75	0.34	0.214
2	CC013XCC041	F	Y	16.13	10.06	0.21	0.17	0.1326
2	CC013XCC041	F	Y	48.77	44.67	0.23	0.18	0.208
2	CC013XCC041	F	Y	8.05	6.67	0.18	0.2	0.2447
2	CC013XCC041	M	Y	30.42	27.13	0.34	0.17	-0.4451
2	CC013XCC041	M	Y	69.28	78.44	0.83	1.17	5.42E-02
0	CC015	F	Y	31.1	25.41	0.52	0.6	7.76E-02
0	CC015	M	Y	11.76	9.51	0.32	0.29	0.1231
0	CC015	M	Y	16.25	33.02	0.3	0.18	0.1049
1	CC015	F	Y	39.58	39.5	1.03	1.3	-1.7486
1	CC015	M	Y	82.92	70.57	0.79	0.32	7.10E-02
1	CC015	M	Y	70.78	66.2	0.8	0.68	-5.19E-02
1	CC015	M	Y	64.31	57.11	0.83	0.72	0.3268
2	CC015	F	Y	10.39	8.81	0.63	0.15	9.98E-02
0	CC017	F	Y	62.2	42.94	0.79	0.8	6.73E-02
0	CC017	M	Y	55.41	33.52	0.61	0.73	0.1296
0	CC017	F	Y	48.33	38.14	1.21	0.75	0.2037
0	CC017	M	Y	101.28	68.41	1.03	0.78	6.57E-02
1	CC017	M	Y	92.9	61.64	1.3	0.92	0.1621
1	CC017	F	Y	65.38	68.01	0.91	0.74	0.1558
0	CC023	M	Y	29.3	17.68	0.52	0.61	0.2372
0	CC023	F	Y	51.32	100.43	0.71	1.01	0.1805
0	CC023	F	Y	66.14	71.59	0.4	0.46	6.38E-02
0	CC023	M	Y	72.48	8.71	1.13	1.1	0.1088
1	CC023	M	Y	77.79	52.25	0.51	0.66	-9.73E-02
1	CC023	F	Y	85.77	81.19	0.66	0.47	9.33E-02
1	CC023	M	Y	50.29	36.71	0.96	0.79	0.5774
1	CC023	M	Y	50.44	10.17	0.62	0.15	6.87E-02
2	CC023	M	Y	19.39	12.78	0.19	0.18	6.99E-02
2	CC023	F	Y	66.38	28.26	0.79	0.42	0.1498
2	CC023	M	Y	57.01	52.54	0.84	0.34	0.712
0	CC025	M	Y	24.76	27.71	0.58	0.47	0.1912
0	CC025	F	Y	47.64	39.02	0.7	0.61	0.1038
0	CC025	M	Y	9.37	46.39	0.44	0.1	0.2
1	CC025	M	Y	24.96	21.74	0.49	0.47	0.2602
1	CC025	F	Y	41.05	53.46	0.68	0.82	-0.3189
1	CC025	F	Y	25.3	16.93	0.58	0.46	0.1049
1	CC025	F	Y	41.26	59.59	0.72	0.59	9.96E-02
2	CC025	F	Y	38.37	16.54	0.77	0.76	0.6116
2	CC025	M	Y	12.52	21.24	0.62	1.06	2.74E-02
0	CC027	F	Y	47.22	28.93	1.66	0.45	9.99E-02
0	CC027	M	Y	59.02	24.92	0.63	1.28	0.2694
1	CC027	M	Y	63.55	8.7	1.41	1.47	9.99E-02
1	CC027	M	Y	63.55	8.7	1.41	1.47	9.99E-02
1	CC027	F	Y	54.03	53.23	0.98	0.86	0.1396
1	CC027	F	Y	55.63	97.39	0.67	0.4	0.1149
1	CC027	M	Y	7.37	13.32	0.38	1.16	0.1797

Trial#	Strain	Sex	Inf?	SwingDurationCV_RF	SwingDurationCV_RH	SWVar_LF	SWVar_LH	Tau-Propulsion_LH
1	CC027	M	Y	9.8	13.54	0.16	0.14	0.167
2	CC027	F	Y	66.38	28.26	0.79	0.42	0.1498
2	CC027	M	Y	13.27	14.69	0.56	0.57	0.2105
2	CC027	F	Y	63.81	54.72	0.98	1.11	-1.70E-03
2	CC027	M	Y	11.59	19.73	0.39	0.29	0.1039
1	CC032XCC013	M	Y	51.67	51.17	0.88	0.36	4.5891
1	CC032XCC013	M	Y	45.33	33.47	0.54	0.45	0.1828
1	CC032XCC013	M	Y	53.76	62.5	0.82	0.46	6.55E-02
1	CC032XCC013	F	Y	104.69	77.07	0.78	0.8	0.1067
1	CC032XCC013	F	Y	55.19	25.9	0.64	0.19	9.30E-02
1	CC032XCC013	F	Y	46.66	19.95	0.42	0.44	0.1214
1	CC032XCC013	F	Y	43.43	20.18	0.34	0.35	0.1179
1	CC032XCC013	F	Y	44.76	59.11	0.32	0.16	0.1241
2	CC032XCC013	M	Y	7.75	27.31	0.09	0.15	0.2259
2	CC032XCC013	M	Y	50.65	57.04	0.74	0.19	0.1997
2	CC032XCC013	M	Y	69.87	57.43	0.94	0.9	0.2639
2	CC032XCC013	M	Y	72.84	62.45	0.5	0.41	0.1063
2	CC032XCC013	F	Y	34.79	22.56	0.2	0.17	0.6159
2	CC032XCC013	F	Y	41.59	21.66	0.53	0.23	0.1768
2	CC032XCC013	F	Y	39.77	14.2	0.66	0.19	0.1077
2	CC032XCC013	F	Y	48.67	17.9	0.49	0.19	0.2974
0	CC037	F	Y	63.37	42.52	0.64	0.28	0.1807
0	CC037	M	Y	66.71	49.06	0.86	0.8	7.13E-02
0	CC037	M	Y	47.42	7.83	0.55	0.1	7.70E-02
0	CC037	M	Y	45.59	64.65	0.36	0.85	0.1376
1	CC037	F	Y	17.93	24.11	0.46	0.45	5.76E-02
1	CC037	M	Y	22.38	36.44	0.98	0.57	0.1057
1	CC037	M	Y	48.94	31.14	0.83	0.34	0.1307
1	CC037	M	Y	56.69	60.09	0.57	0.37	0.1032
2	CC037	M	Y	45.13	66.21	0.49	0.2	9.17E-02
2	CC037	M	Y	28.93	20.73	0.23	0.11	7.40E-02
1	CC041XCC012	M	Y	71.71	62.9	0.66	0.45	0.2542
1	CC041XCC012	M	Y	17.86	6.45	0.51	0.13	0.1529
1	CC041XCC012	M	Y	69.3	84.42	0.16	0.41	0.6128
1	CC041XCC012	M	Y	51.23	65.57	0.55	0.34	0.1261
1	CC041XCC012	M	Y	49.66	15.14	0.24	0.26	0.1866
1	CC041XCC012	F	Y	44.92	46.57	0.73	0.41	0.1646
1	CC041XCC012	F	Y	59.9	38.19	0.47	0.21	0.1646
1	CC041XCC012	F	Y	46.38	41.91	0.14	0.23	0.114
1	CC041XCC012	F	Y	25.17	12.68	0.28	0.4	0.1566
1	CC041XCC012	F	Y	69.3	53.64	0.45	0.86	0.1208
1	CC041XCC012	F	Y	56.61	23.07	0.66	0.17	0.1664
1	CC041XCC012	F	Y	19.93	6.67	0.37	0.29	0.2022
1	CC041XCC012	F	Y	70.91	61.14	0.23	0.81	9.99E-02
2	CC041XCC012	M	Y	32.02	40.32	0.2	0.19	0.1569
2	CC041XCC012	M	Y	48.11	11.66	0.15	0.21	0.1514
2	CC041XCC012	F	Y	22.61	12.82	0.45	0.16	0.1205
2	CC041XCC012	M	Y	43.59	18.94	0.62	0.71	-1.0501

Trial#	Strain	Sex	Inf?	SwingDurationCV_RF	SwingDurationCV_RH	SWVar_LF	SWVar_LH	Tau-Propulsion_LH
2	CC041XCC012	M	Y	15.85	10.32	0.39	0.25	0.1314
2	CC041XCC012	M	Y	24.71	16.01	0.41	0.22	-0.1301
2	CC041XCC012	F	Y	14.55	13.08	0.33	0.21	0.15
2	CC041XCC012	F	Y	31.55	41.56	0.31	0.64	0.881
2	CC041XCC012	F	Y	32.54	14	0.31	0.23	0.1886
2	CC041XCC012	F	Y	20.62	17.65	0.44	0.11	0.121
2	CC041XCC012	F	Y	33.46	16.42	0.71	0.56	0.411
2	CC041XCC012	F	Y	13.5	9.86	0.25	0.29	0.1736
2	CC041XCC012	F	Y	33.68	17.35	0.75	0.3	0.1362
0	CC051	F	Y	36.79	33.25	0.83	0.95	0.2034
0	CC051	F	Y	64.73	31.76	0.52	0.38	0.1735
0	CC051	F	Y	38.98	34.1	0.52	0.35	0.1694
0	CC051	M	Y	37.15	28.82	0.34	0.13	0.1949
1	CC051	F	Y	10.89	19.23	0.92	0.69	0.1668
1	CC051	F	Y	33.96	14.04	0.53	0.55	-8.3882
1	CC051	F	Y	30.87	17	1.41	0.54	0.143
0	CC057	F	Y	18.86	13.39	0.54	0.24	7.40E-02
0	CC057	F	Y	27.5	34.59	0.76	0.29	2.22E-02
0	CC057	F	Y	81.39	54.84	0.82	0.33	0.1626
0	CC057	M	Y	29.57	65.85	0.67	0.66	0.1315
0	CC057	M	Y	45.09	64.01	0.8	0.68	0.1131
0	CC057	M	Y	70.87	68.5	0.98	0.25	-0.1016
1	CC057	F	Y	32.94	6.7	0.83	0.26	8.14E-02
1	CC057	F	Y	65.52	65.77	0.58	0.5	0.2814
1	CC057	F	Y	75.12	56.56	0.88	0.94	0.8639
1	CC057	F	Y	70.8	70.42	1.11	0.52	9.55E-02
1	CC057	M	Y	44.93	7.28	0.75	0.11	0.1636
1	CC057	M	Y	33.32	11.11	0.45	0.41	0.5734
2	CC057	F	Y	70.8	70.42	1.11	0.52	9.55E-02
0	CC078	F	Y	7.6	46.87	0.19	0.18	0.1184
0	CC078	F	Y	21.65	27.62	0.29	0.43	7.04E-02
0	CC078	F	Y	14.49	9.43	0.3	0.1	8.77E-02
0	CC078	M	Y	16.6	8.09	0.32	0.09	0.1152
1	CC078	F	Y	13.07	15.67	0.15	0.15	7.94E-02
1	CC078	F	Y	33.12	35.72	0.3	0.13	8.54E-02
1	CC078	M	Y	15.84	12.23	0.38	0.19	7.65E-02
2	CC078	F	Y	44.39	52.57	0.34	0.48	0.1438
2	CC078	M	Y	38.62	37.76	0.49	0.52	-0.1149
2	CC005	M	N	61.69	86.93	0.6	1	-0.0588
2	CC005	M	Y	77.92	53.86	0.77	0.97	0.1797
2	CC015	F	N	79.79	56.03	0.86	0.63	-0.0059
2	CC015	M	N	19.13	8.66	0.14	0.08	0.2085
2	CC015	M	Y	54.79	34.7	0.23	0.26	0.2365
2	CC015	M	Y	61.85	47.21	0.76	0.82	-0.163
2	CC017	F	N	71.28	41.01	0.59	0.13	0.1197
2	CC017	M	N	62.89	35.7	0.47	0.31	0.0909
2	CC017	F	Y	95.12	67.39	0.78	0.53	-1.4964
2	CC023	F	N	27.12	33.84	0.39	0.24	0.1098

Trial#	Strain	Sex	Inf?	SwingDurationCV_RF	SwingDurationCV_RH	SWVar_LF	SWVar_LH	Tau-Propulsion_LH

Table S3. Raw DigiGait measurement data. Far left column indicates time point at which data was measured: T0 = pre-infection, T1 = 21dpi, and T3 = 89dpi. DigiGait parameters listed across the top indicate which limb is associated with the data, where appropriate: FL for left fore limb, FR for right fore limb, HL for left hind limb, and HR for right hind limb.

Table S3

Trial#	Strain	Sex	Inf?	Tau-Propulsion_RH
1	CC002	F	N	0.2674
1	CC025	F	N	9.01E-02
1	CC012XCC032	F	N	0.1192
2	CC012XCC032	F	N	0.1364
1	CC012XCC032	M	N	-0.957
2	CC012XCC032	M	N	0.1094
1	CC013xCC041	F	N	0.273
2	CC013XCC041	F	N	0.2794
1	CC013xCC041	M	N	4.61E-02
2	CC013XCC041	M	N	0.5681
1	CC032XCC013	F	N	11.5267
2	CC032XCC013	F	N	0.1218
1	CC041XCC012	F	N	6.97E-02
1	CC041XCC012	F	N	0.2305
2	CC041XCC012	F	N	0.1466
1	CC032XCC013	M	N	8.93E-02
2	CC032XCC013	M	N	0.1391
1	CC041XCC012	M	N	0.2263
2	CC041XCC012	M	N	0.127
0	CC012	F	N	0.1091
2	CC012	F	N	0.1263
0	CC012	M	N	0.1161
2	CC012	M	N	0.1338
0	CC057	F	N	7.18E-02
1	CC057	F	N	0.1925
0	CC057	M	N	8.95E-02
1	CC057	M	N	0.1641
0	CC078	F	N	0.1308
1	CC078	F	N	0.1369
2	CC078	F	N	13.3789
0	CC078	M	N	0.1008
2	CC078	M	N	0.2044
0	CC002	F	N	5.71E-02
1	CC002	F	N	0.2674
0	CC002	M	N	4.58E-02
1	CC002	M	N	0.4089
2	CC002	M	N	0.1011
0	CC006	F	N	8.54E-02
1	CC006	F	N	0.2111
2	CC006	F	N	0.1793
0	CC006	M	N	3.77E-02
0	CC023	F	N	-3.70E-03
1	CC023	F	N	-3.2984
2	CC023	F	N	0.1241
0	CC023	M	N	0.2056
1	CC023	M	N	0.1349
0	CC027	F	N	-Inf

Trial#	Strain	Sex	Inf?	Tau-Propulsion_RH
1	CC027	F	N	-0.71
2	CC027	F	N	9.63E-02
0	CC027	M	N	4.78E-02
1	CC027	M	N	4.94E-02
1	CC027	M	N	0.1553
2	CC027	M	N	0.1592
0	CC005	F	N	8.85E-02
1	CC005	F	N	1.9012
2	CC005	F	N	9.44E-02
0	CC011	F	N	0.2172
1	CC011	F	N	0.4435
2	CC011	F	N	0.173
1	CC017	F	N	0.1339
0	CC005	M	N	0.1225
1	CC005	M	N	-5.00E-03
2	CC005	M	N	0.2598
0	CC011	M	N	0.1348
1	CC011	M	N	0.3573
2	CC011	M	N	1.4513
0	CC017	M	N	8.35E-02
1	CC017	M	N	0.1552
1	CC006	F	N	1.77E-02
0	CC037	F	N	7.58E-02
0	CC051	F	N	0.3496
1	CC051	F	N	0.3767
1	CC006	M	N	0.1081
0	CC037	M	N	0.103
1	CC037	M	N	0.1131
0	CC005	F	N	9.24E-02
1	CC005	F	N	0.1528
2	CC005	F	N	9.81E-02
0	CC011	F	N	0.1285
1	CC011	F	N	0.1212
2	CC011	F	N	0.1807
0	CC011	M	N	6.88E-02
1	CC011	M	N	9.13E-02
2	CC011	M	N	0.1889
1	CC037	M	N	0.1316
2	CC037	M	N	0.1323
1	CC051	M	N	0.1803
2	CC051	M	N	0.3651
0	CC027	F	N	-0.1388
1	CC027	F	N	0.1122
2	CC027	F	N	0.1092
0	CC015	M	N	8.07E-02
1	CC015	M	N	1.3704
2	CC015	M	N	0.5758
0	CC027	M	N	0.1029

Trial#	Strain	Sex	Inf?	Tau-Propulsion_RH
1	CC027	M	N	8.11E-02
2	CC027	M	N	5.08E-02
0	CC015	F	N	NA
1	CC015	F	N	0.1202
0	CC017	F	N	4.68E-02
0	CC023	F	N	1.0238
1	CC023	F	N	8.52E-02
0	CC005	M	N	9.71E-02
1	CC005	M	N	0.1051
0	CC015	M	N	9.68E-02
1	CC015	M	N	0.1513
0	CC017	M	N	0.125
1	CC017	M	N	0.2442
0	CC023	M	N	8.02E-02
1	CC023	M	N	0.2188
0	CC051	M	N	0.1074
0	CC002	F	Y	-0.4204
0	CC002	M	Y	2.1891
1	CC002	M	Y	0.1275
1	CC002	F	Y	-3.556
1	CC002	F	Y	0.2478
1	CC002	M	Y	0.1275
1	CC002	M	Y	4.45E-02
1	CC002	F	Y	-3.556
1	CC002	F	Y	0.2214
2	CC002	M	Y	30.8496
2	CC002	F	Y	0.1612
2	CC002	M	Y	0.1343
0	CC005	F	Y	0.1164
0	CC005	M	Y	0.1125
0	CC005	M	Y	0.6003
1	CC005	F	Y	9.70E-02
1	CC005	M	Y	0.1057
1	CC005	F	Y	8.40E-02
1	CC005	M	Y	0.1014
2	CC005	F	Y	0.1201
2	CC005	M	Y	0.2933
2	CC005	M	Y	-0.1371
2	CC005	M	Y	0.2933
2	CC005	F	Y	0.503
0	CC006	F	Y	0.26
0	CC006	M	Y	-Inf
0	CC006	F	Y	0.195
1	CC006	F	Y	0.1041
1	CC006	M	Y	0.229
1	CC006	F	Y	0.1233
1	CC006	M	Y	0.1202
2	CC006	F	Y	0.2168

Trial#	Strain	Sex	Inf?	Tau-Propulsion_RH
2	CC006	M	Y	5.31E-02
0	CC011	F	Y	0.1715
0	CC011	M	Y	0.2043
0	CC011	F	Y	7.66E-02
0	CC011	F	Y	-7.55E-02
1	CC011	F	Y	0.1693
1	CC011	M	Y	0.179
1	CC011	F	Y	8.11E-02
1	CC011	M	Y	0.1316
2	CC011	F	Y	0.1713
2	CC011	M	Y	0.3876
2	CC011	F	Y	0.1851
2	CC011	M	Y	9.53E-02
0	CC012	M	Y	0.1527
0	CC012	M	Y	0.1232
0	CC012	M	Y	9.36E-02
0	CC012	M	Y	0.1326
0	CC012	M	Y	-0.4231
0	CC012	F	Y	0.1553
0	CC012	F	Y	0.2965
0	CC012	F	Y	0.2253
0	CC012	F	Y	0.3604
0	CC012	F	Y	0.217
2	CC012	M	Y	0.3966
2	CC012	M	Y	0.2091
2	CC012	M	Y	0.17
2	CC012	M	Y	0.2377
2	CC012	M	Y	0.5799
2	CC012	F	Y	0.1369
2	CC012	F	Y	0.299
2	CC012	F	Y	0.5213
2	CC012	F	Y	0.2418
2	CC012	F	Y	-0.1733
1	CC012XCC032	F	Y	-3.246
1	CC012xCC032	F	Y	2.58E-02
1	CC012XCC032	M	Y	0.2394
1	CC012xCC032	M	Y	0.1424
1	CC012XCC032	M	Y	4.77E-02
1	CC012xCC032	M	Y	8.68E-02
2	CC012XCC032	F	Y	9.88E-02
2	CC012XCC032	F	Y	0.1223
2	CC012XCC032	M	Y	0.2653
2	CC012XCC032	M	Y	7.63E-02
2	CC012XCC032	M	Y	0.7069
2	CC012XCC032	M	Y	-2.20E-03
1	CC013xCC041	F	Y	0.4049
1	CC013xCC041	F	Y	8.76E-02
1	CC013xCC041	F	Y	0.2381

Trial#	Strain	Sex	Inf?	Tau-Propulsion_RH
1	CC013xCC041	M	Y	0.2078
1	CC013xCC041	M	Y	5.94E-02
2	CC013XCC041	F	Y	0.7871
2	CC013XCC041	F	Y	-0.1025
2	CC013XCC041	F	Y	0.159
2	CC013XCC041	M	Y	-0.4292
2	CC013XCC041	M	Y	-7.00E-04
0	CC015	F	Y	0.1384
0	CC015	M	Y	0.1102
0	CC015	M	Y	0.1601
1	CC015	F	Y	0.1094
1	CC015	M	Y	9.69E-02
1	CC015	M	Y	7.43E-02
1	CC015	M	Y	0.2899
2	CC015	F	Y	0.1265
0	CC017	F	Y	0.1094
0	CC017	M	Y	-0.4989
0	CC017	F	Y	0.288
0	CC017	M	Y	-5.2868
1	CC017	M	Y	0.3818
1	CC017	F	Y	0.7857
0	CC023	M	Y	0.3157
0	CC023	F	Y	8.54E-02
0	CC023	F	Y	0.1655
0	CC023	M	Y	0.1203
1	CC023	M	Y	-0.1212
1	CC023	F	Y	0.5406
1	CC023	M	Y	0.1933
1	CC023	M	Y	0.1833
2	CC023	M	Y	7.46E-02
2	CC023	F	Y	0.1126
2	CC023	M	Y	0.1412
0	CC025	M	Y	-4.3164
0	CC025	F	Y	0.2029
0	CC025	M	Y	0.1157
1	CC025	M	Y	0.1375
1	CC025	F	Y	0.2377
1	CC025	F	Y	0.1965
1	CC025	F	Y	7.92E-02
2	CC025	F	Y	0.6111
2	CC025	M	Y	0.1472
0	CC027	F	Y	7.81E-02
0	CC027	M	Y	1.45E-02
1	CC027	M	Y	7.03E-02
1	CC027	M	Y	7.03E-02
1	CC027	F	Y	0.1782
1	CC027	F	Y	-1.51E-02
1	CC027	M	Y	10.2744

Trial#	Strain	Sex	Inf?	Tau-Propulsion_RH
1	CC027	M	Y	9.42E-02
2	CC027	F	Y	0.1126
2	CC027	M	Y	0.1118
2	CC027	F	Y	0.1437
2	CC027	M	Y	0.114
1	CC032XCC013	M	Y	0.139
1	CC032XCC013	M	Y	0.1382
1	CC032XCC013	M	Y	0.2126
1	CC032XCC013	F	Y	0.235
1	CC032XCC013	F	Y	9.14E-02
1	CC032XCC013	F	Y	0.1317
1	CC032XCC013	F	Y	0.133
1	CC032XCC013	F	Y	7.96E-02
2	CC032XCC013	M	Y	0.3346
2	CC032XCC013	M	Y	0.1313
2	CC032XCC013	M	Y	0.1448
2	CC032XCC013	M	Y	8.70E-02
2	CC032XCC013	F	Y	0.1528
2	CC032XCC013	F	Y	9.87E-02
2	CC032XCC013	F	Y	0.1021
2	CC032XCC013	F	Y	0.2427
0	CC037	F	Y	0.1883
0	CC037	M	Y	0.1541
0	CC037	M	Y	8.92E-02
0	CC037	M	Y	6.76E-02
1	CC037	F	Y	0.1193
1	CC037	M	Y	6.99E-02
1	CC037	M	Y	0.1297
1	CC037	M	Y	0.3887
2	CC037	M	Y	0.1305
2	CC037	M	Y	8.62E-02
1	CC041XCC012	M	Y	0.1978
1	CC041XCC012	M	Y	0.2838
1	CC041XCC012	M	Y	0.1278
1	CC041XCC012	M	Y	5.51E-02
1	CC041XCC012	M	Y	0.6778
1	CC041XCC012	F	Y	0.1152
1	CC041XCC012	F	Y	0.1879
1	CC041XCC012	F	Y	5.94E-02
1	CC041XCC012	F	Y	0.4774
1	CC041XCC012	F	Y	0.1098
1	CC041XCC012	F	Y	9.51E-02
1	CC041XCC012	F	Y	0.2362
1	CC041XCC012	F	Y	0.1257
2	CC041XCC012	M	Y	0.1465
2	CC041XCC012	M	Y	0.2336
2	CC041XCC012	F	Y	0.1026
2	CC041XCC012	M	Y	9.73E-02

Trial#	Strain	Sex	Inf?	Tau-Propulsion_RH
2	CC041XCC012	M	Y	0.1519
2	CC041XCC012	M	Y	0.1891
2	CC041XCC012	F	Y	0.1986
2	CC041XCC012	F	Y	0.2019
2	CC041XCC012	F	Y	0.1628
2	CC041XCC012	F	Y	1.2576
2	CC041XCC012	F	Y	0.3071
2	CC041XCC012	F	Y	0.1024
2	CC041XCC012	F	Y	0.2478
0	CC051	F	Y	0.1351
0	CC051	F	Y	0.2076
0	CC051	F	Y	NA
0	CC051	M	Y	0.1374
1	CC051	F	Y	9.84E-02
1	CC051	F	Y	0.1494
1	CC051	F	Y	0.1696
0	CC057	F	Y	8.18E-02
0	CC057	F	Y	8.90E-03
0	CC057	F	Y	4.00E-04
0	CC057	M	Y	0.1244
0	CC057	M	Y	0.3008
0	CC057	M	Y	0.1745
1	CC057	F	Y	0.1427
1	CC057	F	Y	0.1775
1	CC057	F	Y	-0.1211
1	CC057	F	Y	6.69E-02
1	CC057	M	Y	0.1768
1	CC057	M	Y	0.1632
2	CC057	F	Y	6.69E-02
0	CC078	F	Y	7.85E-02
0	CC078	F	Y	7.43E-02
0	CC078	F	Y	9.20E-02
0	CC078	M	Y	6.68E-02
1	CC078	F	Y	7.46E-02
1	CC078	F	Y	8.61E-02
1	CC078	M	Y	0.1872
2	CC078	F	Y	0.1781
2	CC078	M	Y	0.1576
2	CC005	M	N	-Inf
2	CC005	M	Y	8.77E-02
2	CC015	F	N	0.137
2	CC015	M	N	0.1269
2	CC015	M	Y	0.1279
2	CC015	M	Y	0.1501
2	CC017	F	N	0.1956
2	CC017	M	N	7.04E-02
2	CC017	F	Y	6.91E-02
2	CC023	F	N	0.1103

Trial#	Strain	Sex	Inf?	Tau-Propulsion_RH

Table S3. Raw DigiGait measurement data. Far left column indicates time point at which data was measured: T0 = pre-infection, T1 = 21dpi, and T3 = 89dpi. DigiGait parameters listed across the top indicate which limb is associated with the data, where appropriate: FL for left fore limb, FR for right fore limb, HL for left hind limb, and HR for right hind limb.

Table S4.

CC or RIX strain	Clinical progression profile / *cumulative score*				
	All		Females		Males
CC002	remitting	*-0.837*	remitting	*-0.738*	remitting
CC005	most progressive	*0.564*	moderately progressive	*0.250*	most progressive
CC006	non-progressive	*-0.002*	non-progressive	*-0.049*	non-progressive
CC011	remitting	*-0.375*	non-progressive	*-0.226*	remitting
CC012	moderately progressive	*0.193*	moderately progressive	*0.143*	moderately progressive
CC012xCC032	moderately progressive	*0.114*	moderately progressive	*0.050*	moderately progressive
CC013xCC041	moderately progressive	*0.123*	moderately progressive	*0.150*	moderately progressive
CC015	non-progressive	*-0.045*	non-progressive	*-0.080*	non-progressive
CC017	moderately progressive	*0.087*	non-progressive	*-0.130*	moderately progressive
CC023	most progressive	*0.527*	most progressive	*0.711*	moderately progressive
CC025	remitting	*-0.315*	remitting	*-0.726*	moderately progressive
CC027	non-progressive	*0.041*	moderately progressive	*0.073*	non-progressive
CC032xCC013	non-progressive	*-0.089*	non-progressive	*-0.046*	non-progressive
CC037	moderately progressive	*0.214*	most progressive	*0.407*	non-progressive
CC041xCC012	moderately progressive	*0.211*	moderately progressive	*0.133*	moderately progressive
CC051	non-progressive	*-0.063*	non-progressive	*-0.043*	non-progressive
CC057	moderately progressive	*0.235*	non-progressive	*-0.005*	most progressive
CC078	moderately progressive	*0.269*	most progressive	*0.519*	non-progressive

Table S4. Summaries of qualitative phenotypes for all strains.
Statistically significant ANOVA values for qualitative phenotypes are shown in bold italics.

Table S4.

CC or RIX strain		Delayed righting reflex	Paresis: Pr(>|z|)				Paralysis: Pr(>|z|)		
			Left fore	*Right fore*	*Left hind*	*Right hind*	*Left fore*	*Right fore*	*Left hind*
CC002	*-0.936*	3dpi	***0***	***0***	***0***	***0.001***	***0***	***0***	***0***
CC005	*0.878*	3dpi	***0***	***0***	***0***	***0***	***0***	***0***	***0***
CC006	*0.045*	4dpi	***0***	***0***	***0***	***0***	***0***	***0***	***0***
CC011	*-0.524*	2dpi	***0***	***0***	***0***	***0***	***0***	***0***	***0***
CC012	*0.243*	2dpi	***0***	***0***	***0***	***0***	***0***	***0***	***0***
CC012xCC032	*0.179*	3dpi	0.108	0.108	0.346	0.649	0.113	0.113	0.157
CC013xCC041	*0.096*	2dpi	0.168	0.157	0.457	0.751	0.175	0.175	0.187
CC015	*-0.009*	2dpi	***0.004***	***0.004***	***0***	***0***	***0.004***	***0.004***	***0.004***
CC017	*0.304*	2dpi	***0.001***	***0.001***	***0***	***0***	***0.001***	***0.001***	***0.001***
CC023	*0.343*	2dpi	***0***	***0***	***0***	***0***	***0***	***0***	***0***
CC025	*0.096*	2dpi	***0.001***	***0.001***	***0.031***	0.091	***0.001***	***0.001***	***0.001***
CC027	*0.009*	2dpi	***0***	***0***	***0.004***	***0***	***0***	***0***	***0***
CC032xCC013	*-0.132*	2dpi	***0.001***	***0.001***	0.496	***0.033***	***0.001***	***0.001***	***0.001***
CC037	*0.021*	2dpi	***0***	***0***	***0***	***0***	***0***	***0***	***0***
CC041xCC012	*0.290*	2dpi	0.061	0.056	***0***	***0.001***	0.056	0.056	0.054
CC051	*-0.083*	8dpi	***0***	***0***	***0.006***	0.07	***0***	***0***	***0***
CC057	*0.475*	3dpi	***0.023***	***0.023***	***0***	***0***	0.065	0.063	0.064
CC078	*0.019*	3dpi	***0.02***	***0.02***	***0***	***0***	0.067	0.144	0.312

Table S4.

CC or RIX strain		Seizures	Clonus	Encephalitis	Hunch	Ruffledness
	Right hind	*count*	*Pr(>|z|)*	*Pr(>|z|)*	*Pr(>|z|)*	*Pr(>|z|)*
CC002	***0***	4	0.387	***0.001***	***0***	0.273
CC005	***0***	0	***0***	***0.004***	***0***	***0***
CC006	***0***	0	0.769	***0***	0.074	***0.008***
CC011	***0***	2	***0***	***0***	***0.031***	***0***
CC012	***0***	0	***0***	***0***	0.97	***0***
CC012xCC032	0.162	0	***0.008***	0.123	0.995	0.454
CC013xCC041	0.193	0	0.983	0.189	0.996	0.134
CC015	***0.004***	0	***0***	***0.004***	0.998	0.992
CC017	***0.002***	0	0.6	0.167	***0.008***	***0.022***
CC023	***0***	2	0.548	***0***	***0***	***0***
CC025	***0.002***	2	***0***	***0.001***	***0***	***0***
CC027	***0***	0	0.113	***0***	0.996	0.981
CC032xCC013	***0.001***	1	0.169	***0.002***	***0.041***	0.359
CC037	***0***	0	0.939	***0***	0.997	0.665
CC041xCC012	0.065	2	0.475	0.095	0.657	0.458
CC051	***0***	0	***0***	***0***	0.351	***0.026***
CC057	0.131	0	***0***	0.066	***0.018***	0.226
CC078	0.267	0	***0***	0.222	***0.001***	***0***

Table S5

Parameter	Category
#Steps_LF	temporal
#Steps_LH	temporal
#Steps_RF	temporal
#Steps_RH	temporal
%BrakeStance_LF	temporal
%BrakeStance_LH	temporal
%BrakeStance_RF	temporal
%BrakeStance_RH	temporal
%BrakeStride_LF	kinetic
%BrakeStride_LH	kinetic
%BrakeStride_RF	kinetic
%BrakeStride_RH	kinetic
%PropelStance_LF	temporal
%PropelStance_LH	temporal
%PropelStance_RF	temporal
%PropelStance_RH	temporal
%PropelStride_LF	kinetic
%PropelStride_LH	kinetic
%PropelStride_RF	kinetic
%PropelStride_RH	kinetic
%SharedStance_LH	temporal
%SharedStance_RH	temporal
%StanceStride_LF	kinetic
%StanceStride_LH	kinetic
%StanceStride_RF	kinetic
%StanceStride_RH	kinetic
%SwingStride_LF	kinetic
%SwingStride_LH	kinetic
%SwingStride_RF	kinetic
%SwingStride_RH	kinetic
AbsolutePawAngle_LF	spatial
AbsolutePawAngle_LH	spatial
AbsolutePawAngle_RF	spatial
AbsolutePawAngle_RH	spatial
AtaxiaCoefficient_LF	coordination
AtaxiaCoefficient_LH	coordination
AtaxiaCoefficient_RF	coordination
AtaxiaCoefficient_RH	coordination
AxisDistance_LF	spatial
AxisDistance_LH	spatial
AxisDistance_RF	spatial

Parameter	Category
AxisDistance_RH	spatial
Brake_LF	kinetic
Brake_LH	kinetic
Brake_RF	kinetic
Brake_RH	kinetic
GaitSymmetry_LF	coordination
GaitSymmetry_LH	coordination
GaitSymmetry_RF	coordination
GaitSymmetry_RH	coordination
HindLimbSharedStanceTime_LH	temporal
MAXdA/dT_LF	kinetic
MAXdA/dT_LH	kinetic
MAXdA/dT_RF	kinetic
MAXdA/dT_RH	kinetic
MidlineDistance_LF	kinetic
MidlineDistance_LH	kinetic
MidlineDistance_RF	kinetic
MidlineDistance_RH	kinetic
MINdA/dT_LF	kinetic
MINdA/dT_LH	kinetic
MINdA/dT_RF	kinetic
MINdA/dT_RH	kinetic
OverlapDistance_LF	coordination
OverlapDistance_LH	coordination
OverlapDistance_RF	coordination
OverlapDistance_RH	coordination
PawAngle_LF	spatial
PawAngle_LH	spatial
PawAngle_RF	spatial
PawAngle_RH	spatial
PawAngleVariability_LF	coordination
PawAngleVariability_LH	coordination
PawAngleVariability_RF	coordination
PawAngleVariability_RH	coordination
PawAreaatPeakStanceinsq.cm_LF	kinetic
PawAreaatPeakStanceinsq.cm_LH	kinetic
PawAreaatPeakStanceinsq.cm_RF	kinetic
PawAreaatPeakStanceinsq.cm_RH	kinetic
PawAreaVariabilityatPeakStanceinsq.cm_LF	kinetic
PawAreaVariabilityatPeakStanceinsq.cm_LH	kinetic
PawAreaVariabilityatPeakStanceinsq.cm_RF	kinetic
PawAreaVariabilityatPeakStanceinsq.cm_RH	kinetic

Parameter	Category
PawDrag_LH	kinetic
PawDrag_RH	kinetic
PawPlacementPositioning[PPP]_LF	coordination
PawPlacementPositioning[PPP]_LH	coordination
PawPlacementPositioning[PPP]_RF	coordination
PawPlacementPositioning[PPP]_RH	coordination
Propel_LF	kinetic
Propel_LH	kinetic
Propel_RF	kinetic
Propel_RH	kinetic
SLVar_LF	temporal
SLVar_LH	temporal
SLVar_RF	temporal
SLVar_RH	temporal
Stance_LF	temporal
Stance_LH	temporal
Stance_RF	temporal
Stance_RH	temporal
Stance/Swing_LF	kinetic
Stance/Swing_LH	kinetic
Stance/Swing_RF	kinetic
Stance/Swing_RH	kinetic
StanceFactor_LF	temporal
StanceFactor_LH	temporal
StanceWidth_LF	spatial
StanceWidth_LH	spatial
StanceWidthCV_LF	spatial
StanceWidthCV_LH	spatial
StepAngle_LF	spatial
StepAngle_LH	spatial
StepAngleCV_LF	spatial
StepAngleCV_LH	spatial
StepAngleVar_LF	spatial
StepAngleVar_LH	spatial
Stride_LF	temporal
Stride_LH	temporal
Stride_RF	temporal
Stride_RH	temporal
StrideFrequency_LF	temporal
StrideFrequency_LH	temporal
StrideFrequency_RF	temporal
StrideFrequency_RH	temporal

Parameter	Category
StrideLength_LF	spatial
StrideLength_LH	spatial
StrideLength_RF	spatial
StrideLength_RH	spatial
StrideLengthCV_LF	spatial
StrideLengthCV_LH	spatial
StrideLengthCV_RF	spatial
StrideLengthCV_RH	spatial
Swing_LF	kinetic
Swing_LH	kinetic
Swing_RF	kinetic
Swing_RH	kinetic
SwingDurationCV_LF	temporal
SwingDurationCV_LH	temporal
SwingDurationCV_RF	temporal
SwingDurationCV_RH	temporal
SWVar_LF	spatial
SWVar_LH	spatial
Tau-Propulsion_LH	kinetic
Tau-Propulsion_RH	kinetic

Table S5. Categories (coordination, kinetic, spatial, or temporal) were assigned for each DigiGait parameter as described in the text. LF = left fore limb, LH = left hind limb, RF = right fore limb, RH = right hind limb.

Table S6

	CC002	CC005	CC006	CC011	CC012	CC012XCC032	CC013XCC041
nSteps_LF	-0.03	-0.49	0.86	-1.23	0.61	1.87	-0.12
nSteps_LH	-0.87	-0.82	1.07	-0.71	0.62	2.48	-0.78
nSteps_RF	0.84	-0.21	1.49	-1.17	0.2	1.68	-0.54
nSteps_RH	-0.19	-0.37	0.85	-0.88	1.18	2.48	-0.62
pBrakeStance_LF	-0.89	-0.24	-2.05	0.11	-0.76	0.8	0.53
pBrakeStance_LH	1.22	-0.35	-0.73	0.18	0.72	1.8	-0.24
pBrakeStance_RF	0.5	-0.01	-1.05	1.3	-1.32	-0.5	-1.16
pBrakeStance_RH	1.64	-0.12	-0.58	-0.29	0.03	2.84	0
pBrakeStride_LF	-0.99	-0.47	-1.63	0.32	-0.7	0.8	0.77
pBrakeStride_LH	0.8	-0.21	-0.6	0.01	0.59	1.64	0.04
pBrakeStride_RF	0.33	-0.2	-1.07	1.38	-1.32	-0.67	-0.97
pBrakeStride_RH	1.15	-0.27	-0.42	-0.51	-0.09	2.67	0.14
pPropelStance_LF	0.89	0.24	2.05	-0.11	0.76	-0.8	-0.53
pPropelStance_LH	-1.22	0.35	0.73	-0.18	-0.72	-1.8	0.24
pPropelStance_RF	-0.5	0.01	1.05	-1.3	1.32	0.5	1.16
pPropelStance_RH	-1.64	0.12	0.58	0.29	-0.03	-2.84	0
pPropelStride_LF	0.61	-0.13	2.23	0.22	0.82	-0.73	-0.13
pPropelStride_LH	-1.19	0.44	0.51	-0.28	-0.89	-2.35	0.75
pPropelStride_RF	-0.79	-0.16	0.73	-0.78	1.02	0.24	1.49
pPropelStride_RH	-1.69	-0.07	0.75	-0.02	-0.26	-3.02	0.08
pSharedStance_LH	-1.59	-0.46	0.34	0.06	-0.9	-1.25	1
pSharedStance_RH	0.13	1.05	-0.49	-0.07	0.32	-0.58	1.72
pStanceStride_LF	-1.01	-1.04	-0.09	0.85	-0.21	0.49	1.21
pStanceStride_LH	-0.95	0.45	-0.01	-0.5	-0.72	-1.75	1.37
pStanceStride_RF	-0.46	-0.43	-0.54	1	-0.56	-0.61	0.51
pStanceStride_RH	-1.27	-0.42	0.52	-0.57	-0.5	-1.65	0.15
pSwingStride_LF	1.01	1.04	0.09	-0.85	0.21	-0.49	-1.21
pSwingStride_LH	0.95	-0.45	0.01	0.5	0.72	1.75	-1.37
pSwingStride_RF	0.46	0.43	0.54	-1	0.56	0.61	-0.51

	CC002	CC005	CC006	CC011	CC012	CC012XCC032	CC013XCC041
pSwingStride_RH	1.44	0.38	-0.81	0.56	0.48	1.93	-0.34
AbsolutePawAngle_LF	-0.65	-0.62	-0.21	1.24	-1.8	-0.54	-0.72
AbsolutePawAngle_LH	1.21	0.19	1.15	0.88	0.53	-1.25	-0.48
AbsolutePawAngle_RF	0.55	-0.17	0.2	1.36	-1.46	0.44	-1.43
AbsolutePawAngle_RH	0.37	0.12	-0.4	0.73	-1.14	-0.9	-0.91
AtaxiaCoefficient_LF	-0.31	-0.49	0.33	-0.33	-1.66	1.59	0.96
AtaxiaCoefficient_LH	-0.47	-0.76	0.36	0.13	-0.44	2.15	0.35
AtaxiaCoefficient_RF	-0.33	-0.27	1.58	-0.43	-0.79	1.32	0.46
AtaxiaCoefficient_RH	0.56	-0.46	0.84	-0.6	-0.44	1.56	0.18
AxisDistance_LF	-0.53	1.25	0.09	0.48	0.05	0.05	0.64
AxisDistance_LH	-0.27	0.13	0.15	0.61	0.58	0.54	0.84
AxisDistance_RF	-0.51	0.81	-0.45	0.08	-0.58	0.58	0.9
AxisDistance_RH	-0.58	0.25	-0.25	-0.01	-0.2	-0.12	-0.7
Brake_LF	-1.6	-0.22	-1.19	0.59	-0.35	-0.66	-0.47
Brake_LH	0.83	0.19	-0.41	0.1	0.51	0.52	0.15
Brake_RF	-0.71	0.36	-0.8	1.02	0.06	-1.91	-1.07
Brake_RH	0.44	0.2	0.06	0	0.2	1.1	0.12
GaitSymmetry_LF	1.34	0.19	-0.06	0.01	-1.7	0.45	0.83
GaitSymmetry_LH	1.33	0.19	-0.06	0.01	-1.69	0.45	0.83
GaitSymmetry_RF	1.33	0.19	-0.06	0.01	-1.69	0.45	0.83
GaitSymmetry_RH	1.31	0.14	-0.12	-0.05	-1.79	0.4	0.79
HindLimbSharedStanceTime_LH	0.22	0.25	0.24	0.24	0.23	0.14	0.27
MAXdAratiodT_LF	-0.9	1.17	-0.94	-0.78	2.34	0.17	0.21
MAXdAratiodT_LH	-0.89	1.1	-1.34	-0.51	1.89	-0.32	1.17
MAXdAratiodT_RF	-0.27	0.89	-1.32	-1.24	0.7	0.24	1.57
MAXdAratiodT_RH	-0.68	1.49	-1.08	-0.51	1.09	-0.62	0.3
MidlineDistance_LF	1.15	-1.51	-0.85	0.63	-0.19	1.02	-0.57
MidlineDistance_LH	-1.14	0.01	0.2	0.58	1.06	-0.7	-1.76
MidlineDistance_RF	0.26	-0.97	-0.46	1.16	-1.37	0.81	-0.17
MidlineDistance_RH	-0.51	-0.98	-0.34	0.11	0.15	-0.65	-0.93

	CC002	CC005	CC006	CC011	CC012	CC012XCC032	CC013XCC041
MINdAratiodT_LF	0.23	-0.44	1.37	0.26	0.25	-1.18	-1.87
MINdAratiodT_LH	1.37	-0.76	1.18	-0.13	-1.29	-1.33	-1.18
MINdAratiodT_RF	-0.96	-1.06	1.06	1.19	1.14	-1.13	-0.69
MINdAratiodT_RH	0.66	-1.14	2.69	1.52	0.27	-0.9	-1.57
OverlapDistance_LF	-0.35	-0.02	-0.01	-0.19	-0.33	-0.88	-0.93
OverlapDistance_LH	-0.33	0	0.01	-0.17	-0.31	-0.87	-0.91
OverlapDistance_RF	0.68	0.29	0.07	-0.73	0.51	-0.66	0.39
OverlapDistance_RH	0.65	0.27	0.04	-0.76	0.48	-0.69	0.36
PawAngle_LF	-0.05	0.49	0.8	-0.63	2.18	0.97	0.74
PawAngle_LH	1.64	0.37	-1.33	-1.11	-0.19	0.86	0.35
PawAngle_RF	0.12	1.11	-0.05	0.63	-1.88	0.58	-1.78
PawAngle_RH	0.26	0.6	-0.57	0.63	-1.04	-1.06	-1.1
PawAngleVariability_LF	1.14	-0.61	0.75	0.12	-0.4	0.99	-0.2
PawAngleVariability_LH	0.13	-0.76	-0.19	0.25	-0.29	2.31	-1.81
PawAngleVariability_RF	0.94	-0.15	0.86	0.4	-0.29	1.83	-1.04
PawAngleVariability_RH	1.92	-0.81	-0.54	-0.05	-0.62	1.59	-0.08
PawAreaatPeakStanceinsq.cm_LF	-1.11	1.26	-1.42	-0.7	1.5	0.18	0.48
PawAreaatPeakStanceinsq.cm_LH	-0.84	1.24	-1.21	-0.64	1.67	-0.03	1.45
PawAreaatPeakStanceinsq.cm_RF	-0.49	0.87	-1.57	-0.86	0.72	-0.2	1.23
PawAreaatPeakStanceinsq.cm_RH	-0.65	1.37	-0.9	-0.39	0.86	-0.41	0.6
PawAreaVariabilityatPeakStanceinsq.cm_LF	-1.01	0.4	0.53	-0.88	1.53	0.9	0.34
PawAreaVariabilityatPeakStanceinsq.cm_LH	-0.96	0.51	-1.41	-0.25	-0.19	1.83	1.37
PawAreaVariabilityatPeakStanceinsq.cm_RF	-0.9	0.24	-1.89	-0.66	-0.47	1.31	2.45
PawAreaVariabilityatPeakStanceinsq.cm_RH	-0.41	-0.07	-0.25	-0.2	-0.15	0.03	-0.38
PawDrag_LH	1.49	-0.49	0.91	0.24	-1.65	-0.96	-0.85
PawDrag_RH	-0.5	-0.63	0.71	0.96	-2.11	0.79	0.11
PawPlacementPositioning[PPP]_LF	-0.75	1.99	-0.91	-0.6	0.17	-0.27	0.34
PawPlacementPositioning[PPP]_LH	-0.75	1.99	-0.91	-0.59	0.17	-0.26	0.35
PawPlacementPositioning[PPP]_RF	-0.46	0.02	0.56	0.18	-0.63	-0.69	-1.43
PawPlacementPositioning[PPP]_RH	-0.46	0	0.54	0.17	-0.64	-0.69	-1.42

	CC002	CC005	CC006	CC011	CC012	CC012XCC032	CC013XCC041
Propel_LF	-0.02	0.08	1.02	0.01	1.18	-1.54	-0.74
Propel_LH	-0.19	0.9	0.11	-0.28	0.12	-3.18	0.18
Propel_RF	-0.77	0.14	-0.07	-0.28	1.5	-1.22	0.25
Propel_RH	-0.5	0.2	0.4	0.29	0	-1.51	0.03
SLVar_LF	-0.67	0.82	0.57	-0.3	-2.07	0.21	0.91
SLVar_LH	0.42	-0.93	0.25	0.48	-0.22	1.75	0.89
SLVar_RF	-2.32	0.21	0.82	0.48	-0.86	1.94	-0.81
SLVar_RH	0.19	-0.36	0.62	-0.18	-1.54	1.08	0.55
Stance_LF	-0.81	-0.04	0.23	0.3	0.77	-1.57	-0.83
Stance_LH	0.36	0.88	-0.17	-0.17	0.41	-2.37	0.23
Stance_RF	-0.85	0.27	-0.46	0.36	0.95	-1.76	-0.42
Stance_RH	-0.02	0.23	0.28	0.21	0.13	-0.29	0.12
StanceratioSwing_LF	-0.99	-0.96	-0.07	0.84	-0.53	0.93	0.5
StanceratioSwing_LH	-0.12	0.36	-0.3	-0.03	-0.54	-2.43	1.43
StanceratioSwing_RF	-1.8	-1	-0.43	1.16	-0.48	-0.33	0.78
StanceratioSwing_RH	-0.4	-0.18	1.11	-0.52	-0.8	-2.43	0.22
StanceFactor_LF	0.47	-0.81	2.1	0.03	-0.32	0.9	0.38
StanceFactor_LH	1.44	1.92	0.22	-0.12	0.59	0.42	0.42
StanceWidth_LF	0.51	-0.96	-0.03	-0.26	0.05	0.66	-0.12
StanceWidth_LH	0.12	0.42	-0.12	-0.03	0.08	0.23	-0.04
StanceWidthCV_LF	0.39	0.14	0.72	-0.09	-0.42	0.86	-0.49
StanceWidthCV_LH	-0.02	-0.51	0.68	-0.67	0.79	1.68	0.47
StepAngle_LF	-0.08	1.01	1.09	0.27	-0.09	-1.02	-0.72
StepAngle_LH	-0.74	0.68	0.61	0.09	-0.3	1.16	0.88
StepAngleCV_LF	-0.28	-0.62	-1.04	-1.12	-0.4	2.01	1.82
StepAngleCV_LH	0.71	-0.21	0.53	0.3	0.31	-1.9	1.4
StepAngleVar_LF	-0.53	-0.13	-0.82	-0.96	-0.55	2.42	1.81
StepAngleVar_LH	0.51	0.02	1.22	0.83	0.41	-1.02	2.46
Stride_LF	-0.6	0.22	0.26	0.07	0.85	-1.71	-0.77
Stride_LH	0.56	0.82	-0.14	-0.08	0.74	-2.02	-0.13

	CC002	CC005	CC006	CC011	CC012	CC012XCC032	CC013XCC041
Stride_RF	-0.83	0.38	-0.45	0.11	1.18	-1.76	-0.49
Stride_RH	0.08	0.22	0.17	0.26	0.18	-0.21	0.07
StrideFrequency_LF	0.5	-0.36	-0.27	-0.02	-0.68	1.81	0.62
StrideFrequency_LH	-0.18	-1.14	0.16	0	-0.69	2.12	0.51
StrideFrequency_RF	0.91	-0.29	0.51	0.05	-0.7	1.41	0.8
StrideFrequency_RH	0.18	-0.23	-0.09	0.1	0.22	2.33	-0.22
StrideLength_LF	-0.72	0.31	0.38	0.19	0.91	-1.53	-0.64
StrideLength_LH	0.41	0.99	0.07	0.12	0.75	-1.71	0.06
StrideLength_RF	-0.92	0.47	-0.34	0.24	1.19	-1.62	-0.33
StrideLength_RH	-0.06	0.27	0.19	0.34	0.15	-0.49	0
StrideLengthCV_LF	0.02	-0.06	0.13	-0.25	-2.15	1.43	0.99
StrideLengthCV_LH	-0.31	-0.98	0.54	0.38	-0.59	2.03	0.45
StrideLengthCV_RF	-0.5	-0.24	1.12	0.22	-1.25	1.82	-0.16
StrideLengthCV_RH	0.57	-0.14	0.74	-0.2	-0.75	1.49	0.56
Swing_LF	-0.15	0.68	0.31	-0.36	0.89	-1.73	-0.55
Swing_LH	0.79	0.36	-0.02	0.19	1.2	-0.38	-0.91
Swing_RF	-0.68	0.58	-0.34	-0.53	1.55	-1.51	-0.58
Swing_RH	0.24	0.23	0.23	0.24	0.24	0.24	0.23
SwingDurationCV_LF	0.55	0.1	-0.63	-0.01	-1.07	1.01	0.95
SwingDurationCV_LH	0.33	-0.16	0.59	0.79	-1.61	1.73	-0.89
SwingDurationCV_RF	0.25	-0.41	0.27	0.36	-1.63	1.57	-0.24
SwingDurationCV_RH	1.19	0.12	0.4	0.63	-0.32	1.27	0.18
SWVar_LF	0.39	-0.21	1.16	-0.46	-0.46	1.02	-0.65
SWVar_LH	-0.31	-0.16	0.03	-0.94	0.73	1.26	-0.5

Table S6. Post-normalization DigiGait data is listed for all DigiGait parameters and strains. LF = left fore limb, LH = left hind limb, RF = right fore limb, RH = right hind limb.

Table S6

	CC015	CC017	CC023	CC025	CC027	CC032XCC013	CC037
nSteps_LF	-2.33	0.33	-0.02	0.61	-0.43	-0.71	1.04
nSteps_LH	-1.6	0.14	-0.37	0.34	-0.87	-0.54	0.42
nSteps_RF	-2.14	-0.31	0.13	-0.12	-0.17	-0.87	0.78
nSteps_RH	-1.94	0.19	-0.45	-0.37	-0.48	-0.84	0.21
pBrakeStance_LF	0.92	0.83	0.02	-2.07	0.51	0.08	0.44
pBrakeStance_LH	0.45	-1.1	-1.28	-2.06	-0.78	0.67	-0.02
pBrakeStance_RF	0.9	1.58	0.62	-1.58	0.93	-0.52	-1.33
pBrakeStance_RH	0.71	-1.13	0.51	-0.59	-1.24	-0.49	-0.88
pBrakeStride_LF	0.88	0.66	-0.2	-2.34	0.61	-0.26	0.64
pBrakeStride_LH	0.78	-0.97	-1.16	-2.54	-0.47	0.63	-0.19
pBrakeStride_RF	1.05	1.19	0.34	-2.21	0.58	-0.42	-0.71
pBrakeStride_RH	1.12	-0.82	0.78	-1.1	-0.78	-0.42	-1.52
pPropelStance_LF	-0.92	-0.83	-0.03	2.07	-0.51	-0.08	-0.44
pPropelStance_LH	-0.45	1.1	1.28	2.06	0.78	-0.67	0.02
pPropelStance_RF	-0.9	-1.58	-0.62	1.58	-0.93	0.52	1.33
pPropelStance_RH	-0.71	1.13	-0.51	0.59	1.24	0.49	0.88
pPropelStride_LF	-1.23	-1.19	-0.5	1.19	0.05	-0.05	0.04
pPropelStride_LH	0.15	0.88	1.51	1.17	1.24	-0.45	-0.37
pPropelStride_RF	-0.45	-1.89	-0.96	0.4	-1	0.47	1.84
pPropelStride_RH	-0.01	1.14	0.07	-0.16	1.24	0.51	0.31
pSharedStance_LH	0.52	0.63	1.82	-1.06	1.68	0.58	-1.11
pSharedStance_RH	-0.22	-0.01	1.7	-1.8	1.78	-1.09	-0.88
pStanceStride_LF	0.21	-0.36	-1.03	-2.67	1.16	-0.55	1.17
pStanceStride_LH	1.71	0.11	0.94	-1.73	1.49	0.14	-0.95
pStanceStride_RF	0.3	-0.63	-0.67	-2.53	-0.38	0.03	1.3
pStanceStride_RH	2.47	0.67	0.71	-1.28	0.83	0.2	-1.03
pSwingStride_LF	-0.21	0.36	1.03	2.67	-1.16	0.55	-1.17
pSwingStride_LH	-1.71	-0.11	-0.94	1.73	-1.49	-0.14	0.95
pSwingStride_RF	-0.3	0.63	0.67	2.53	0.38	-0.03	-1.3

	CC015	CC017	CC023	CC025	CC027	CC032XCC013	CC037
pSwingStride_RH	-0.5	-1	-1.05	1.46	-1.2	-0.41	1.15
AbsolutePawAngle_LF	-0.09	1.44	1.3	-1.41	0.99	-0.45	-0.2
AbsolutePawAngle_LH	-0.3	-0.75	-0.71	-0.67	1.13	0.4	-1.09
AbsolutePawAngle_RF	-0.77	-0.41	0.72	0.94	0.81	-0.78	0.74
AbsolutePawAngle_RH	0.87	-0.85	-0.37	1.13	0.85	0.66	0.84
AtaxiaCoefficient_LF	-1.18	-0.27	1.33	1.59	-0.29	-0.51	-0.63
AtaxiaCoefficient_LH	-1.14	0.52	0.15	-0.97	-0.79	-1.3	0.26
AtaxiaCoefficient_RF	-1.45	-0.17	1.35	-0.04	-0.12	-0.94	0.66
AtaxiaCoefficient_RH	-0.22	1.36	1.5	-1.12	-1.5	-1.67	0.38
AxisDistance_LF	0.36	0.11	0.51	-3.52	0.77	-0.56	-0.03
AxisDistance_LH	-1.74	0.56	0.46	-3.25	0.07	0.42	-0.11
AxisDistance_RF	0.29	0.37	-0.63	-3.07	-0.34	1.15	0.35
AxisDistance_RH	1	-0.06	-0.28	-2.84	0.44	2.47	-0.09
Brake_LF	1.28	0.46	-1.4	-1.64	0.47	1.76	0.5
Brake_LH	0.93	-1.04	-1.38	-2.17	-0.05	0.87	0.09
Brake_RF	0.82	0.9	-1	-1.12	0.28	1.12	-0.22
Brake_RH	-3.71	-0.13	0.38	-0.07	0.13	0.69	-0.42
GaitSymmetry_LF	-0.84	-0.54	1.93	-0.37	1.71	-1.07	0.64
GaitSymmetry_LH	-0.84	-0.54	1.93	-0.36	1.71	-1.06	0.64
GaitSymmetry_RF	-0.84	-0.54	1.93	-0.36	1.71	-1.06	0.64
GaitSymmetry_RH	0.13	-0.61	1.91	-0.43	1.69	-1.15	0.59
HindLimbSharedStanceTime_LH	-4	0.24	0.27	0.19	0.3	0.28	0.21
MAXdAratiodT_LF	-0.15	0.19	-0.34	0.74	-0.83	-0.38	-0.56
MAXdAratiodT_LH	-0.46	0.3	-1.39	0.19	0.2	1.26	-1.06
MAXdAratiodT_RF	0.14	0.66	-0.7	0.6	-0.32	0.5	-0.43
MAXdAratiodT_RH	0.49	0.7	-1.6	-0.49	0.13	1.35	-0.07
MidlineDistance_LF	-1.56	1.27	-1.75	1.32	0.56	-0.63	0.96
MidlineDistance_LH	1.11	-0.15	-2.04	1.08	0.04	1.41	-0.24
MidlineDistance_RF	-1.29	1.35	-1.04	1.59	-0.08	-1.62	0.19
MidlineDistance_RH	2.45	-0.44	-2.04	1.34	0.11	0.34	0.16

	CC015	CC017	CC023	CC025	CC027	CC032XCC013	CC037
MINdAratiodT_LF	0.61	-0.19	-0.85	-0.96	-0.13	2.1	0.03
MINdAratiodT_LH	2.12	0.07	0.89	-0.21	-0.96	1.01	-0.57
MINdAratiodT_RF	0.59	-0.98	-1.72	-0.19	-0.69	1.4	1.18
MINdAratiodT_RH	0.2	-0.37	-0.59	0	-0.08	0.48	0.18
OverlapDistance_LF	0.48	-1.01	0.63	-0.38	-0.11	3.53	-0.02
OverlapDistance_LH	0.15	-0.99	0.66	-0.36	-0.08	3.57	0
OverlapDistance_RF	-0.15	-2.64	1.48	1.11	0.19	-1.02	0.81
OverlapDistance_RH	0.31	-2.66	1.45	1.08	0.16	-1.05	0.78
PawAngle_LF	0.59	-1.55	-1.1	-1.32	-0.39	-0.03	0.45
PawAngle_LH	0.05	0.41	0.43	0.47	-1.41	-0.64	0.87
PawAngle_RF	-0.32	-0.09	0.32	0.44	0.17	-0.46	0.71
PawAngle_RH	1.09	-0.84	-0.35	0.99	1.16	0.57	0.73
PawAngleVariability_LF	-0.55	0.31	0.16	0.36	1.03	-2.89	-0.36
PawAngleVariability_LH	-1.39	0.48	0.72	0.9	-0.37	-1.39	1.01
PawAngleVariability_RF	-1.29	0.39	0.65	0.89	0.04	-1.93	-0.76
PawAngleVariability_RH	-0.52	0.39	0.88	1.96	-0.73	-1.74	0.05
PawAreaatPeakStanceinsq.cm_LF	0.33	0.56	-1.14	0.72	-0.72	0.04	-0.38
PawAreaatPeakStanceinsq.cm_LH	-0.36	-0.01	-1.44	-0.33	0.32	1.18	-0.83
PawAreaatPeakStanceinsq.cm_RF	0.3	1.12	-0.83	0.47	-0.41	0.42	-0.4
PawAreaatPeakStanceinsq.cm_RH	0.78	0.42	-1.71	-0.75	0.07	1.21	-0.25
PawAreaVariabilityatPeakStanceinsq.cm_LF	-1.5	0.83	-0.64	1.78	1.07	-1.74	-0.06
PawAreaVariabilityatPeakStanceinsq.cm_LH	-1.44	1.31	-0.34	0.05	0.31	-0.61	-0.38
PawAreaVariabilityatPeakStanceinsq.cm_RF	-0.51	1.38	0.45	0.3	-0.42	0.05	-0.48
PawAreaVariabilityatPeakStanceinsq.cm_RH	3.95	-0.07	-0.37	0.18	-0.32	-0.23	-0.24
PawDrag_LH	0.51	-0.67	0.82	0.45	0.69	-1.49	1.4
PawDrag_RH	-1.41	-0.43	1.01	1.25	0.73	-1.15	1.52
PawPlacementPositioning[PPP]_LF	1.41	-1.15	0.32	-0.54	-0.05	-0.12	0.85
PawPlacementPositioning[PPP]_LH	1.36	-1.15	0.32	-0.54	-0.05	-0.12	0.86
PawPlacementPositioning[PPP]_RF	1.16	0.11	2.02	0.9	-1.07	1.33	-1
PawPlacementPositioning[PPP]_RH	1.34	0.1	1.98	0.87	-1.06	1.3	-1

	CC015	CC017	CC023	CC025	CC027	CC032XCC013	CC037
Propel_LF	-0.07	-0.62	-1.11	0.75	-0.37	1.06	0.02
Propel_LH	0.15	0.21	0.48	0.77	1.36	0.73	0.07
Propel_RF	0.43	-0.68	-1.41	0.27	-0.55	1.44	1.08
Propel_RH	-3.18	0.46	-0.08	0.25	1	0.93	0.43
SLVar_LF	0.07	0.03	0.59	1.92	0.14	0.58	-2
SLVar_LH	-0.77	1.21	-0.29	-1.32	-0.31	-1.33	-0.61
SLVar_RF	-0.57	0.96	0.36	0.49	-0.61	1.03	-0.11
SLVar_RH	0.05	1.74	2.28	-0.65	-0.67	-0.61	-0.13
Stance_LF	0.6	-0.28	-1.58	-0.23	-0.06	1.73	0.27
Stance_LH	0.71	-0.48	-0.46	-0.72	1.11	1.17	0.11
Stance_RF	0.7	0.04	-1.39	-0.42	-0.19	1.49	0.56
Stance_RH	-3.86	0.25	0.15	0.17	0.58	0.7	0.15
StanceratioSwing_LF	0.16	-0.41	-0.46	-2.29	1.91	-1.37	0.87
StanceratioSwing_LH	1.38	-0.08	1.33	-1.95	0.5	0.18	-0.54
StanceratioSwing_RF	0.2	-0.29	-0.54	-2	-0.35	0.26	0.82
StanceratioSwing_RH	1.25	0.36	1.61	-1.31	0.68	-0.03	-0.93
StanceFactor_LF	-0.82	-0.86	0.22	0.64	0.75	-0.47	-1.45
StanceFactor_LH	-1.38	-0.48	0.49	-0.52	0.5	-2.42	0.42
StanceWidth_LF	-1.14	0.69	-1.86	-1.14	-0.02	0.73	1.06
StanceWidth_LH	-3.85	0.16	-0.02	0.35	0.63	0.84	0.47
StanceWidthCV_LF	0.2	0.26	2.48	0.05	0.29	-1.03	-1.24
StanceWidthCV_LH	-0.43	0.96	1.01	0.07	-1.14	-2.65	-1.02
StepAngle_LF	0.93	-1.52	1.84	-1.04	1.03	-0.14	-0.86
StepAngle_LH	-0.41	-0.45	1.39	0.58	-0.17	-2.09	1.41
StepAngleCV_LF	-0.72	0.8	-0.78	0.9	-0.99	0.17	-0.04
StepAngleCV_LH	0.52	0.71	-0.97	-0.53	-0.34	1.22	-2.39
StepAngleVar_LF	-0.4	0.72	-0.07	0.86	-1.09	0.17	-0.72
StepAngleVar_LH	0.95	-0.33	-0.69	-1.13	-0.91	-0.14	-1.68
Stride_LF	0.54	-0.24	-1.55	0.38	-0.36	1.79	-0.09
Stride_LH	0.14	-0.55	-0.91	-0.18	0.84	1.27	0.54

	CC015	CC017	CC023	CC025	CC027	CC032XCC013	CC037
Stride_RF	0.73	0.17	-1.48	0.07	-0.19	1.64	0.31
Stride_RH	-3.85	0.16	-0.03	0.3	0.52	0.83	0.31
StrideFrequency_LF	-0.9	0.17	1.67	-0.23	0.37	-2	0.5
StrideFrequency_LH	-0.33	0.09	0.88	0.44	-1.03	-1.06	-0.23
StrideFrequency_RF	-1.15	-0.22	1.57	-0.01	0.25	-2.33	-0.05
StrideFrequency_RH	-0.97	0.16	0.81	-0.15	-0.17	-2.46	0.01
StrideLength_LF	0.68	-0.12	-1.4	0.57	-0.24	1.91	-0.99
StrideLength_LH	0.31	-0.31	-0.63	0.09	0.94	1.37	-0.58
StrideLength_RF	0.8	0.29	-1.33	0.09	-0.05	1.72	-0.32
StrideLength_RH	-3.33	0.17	-0.18	0.47	0.83	1.38	-0.18
StrideLengthCV_LF	-0.65	-0.13	1.64	1.15	0.37	-0.8	-0.41
StrideLengthCV_LH	-0.95	0.91	0.33	-0.69	-0.73	-1.4	0.13
StrideLengthCV_RF	-1.22	0.32	1.46	0.13	-0.19	-0.57	0.33
StrideLengthCV_RH	-0.32	1.19	2.01	-0.72	-1.08	-1.84	0.08
Swing_LF	0.36	-0.13	-1.27	1.47	-0.88	1.67	-0.77
Swing_LH	-1.3	-0.47	-1.59	1.12	-0.19	0.95	1.33
Swing_RF	0.69	0.47	-1.44	1.25	-0.13	1.78	-0.34
Swing_RH	-4.01	0.23	0.22	0.25	0.23	0.25	0.25
SwingDurationCV_LF	-1.05	0.01	0.85	2.12	-0.08	-1.89	0.27
SwingDurationCV_LH	-0.96	0.48	1.05	-0.27	-0.36	-0.69	0.92
SwingDurationCV_RF	-1.02	1	1.31	0.63	-0.08	-0.79	0.37
SwingDurationCV_RH	-0.78	0.24	0.72	0.87	-2.11	-2.12	0.7
SWVar_LF	-0.14	0.82	1.14	-0.68	0.66	-0.56	-0.97
SWVar_LH	0.97	0.95	0.72	0.21	-0.36	-2.78	-1.25

Table S6. Post-normalization DigiGait data is listed for all DigiGait parameters and strains. LF = left fore limb, LH = left hind limb, RF = right fore limb, RH = right hind limb.

Table S6

	CC041XCC012	CC051	CC057	CC078
nSteps_LF	-1.1	-0.4	1.2	0.33
nSteps_LH	-0.7	0.22	1.33	0.64
nSteps_RF	-1.26	0.08	1.31	0.3
nSteps_RH	-0.74	0.26	1.17	0.56
pBrakeStance_LF	-0.64	-0.05	1.84	0.63
pBrakeStance_LH	0.06	1.07	-0.64	1.06
pBrakeStance_RF	0.47	0.75	0.81	-0.37
pBrakeStance_RH	-0.46	0.29	-0.64	0.41
pBrakeStride_LF	-0.47	-0.02	1.78	0.61
pBrakeStride_LH	0.1	1.18	-0.64	1.03
pBrakeStride_RF	0.86	0.87	0.77	0.21
pBrakeStride_RH	-0.2	0.39	-0.74	0.62
pPropelStance_LF	0.64	0.05	-1.84	-0.63
pPropelStance_LH	-0.06	-1.07	0.64	-1.06
pPropelStance_RF	-0.47	-0.75	-0.81	0.37
pPropelStance_RH	0.46	-0.29	0.64	-0.41
pPropelStride_LF	0.93	0.29	-2.18	-0.23
pPropelStride_LH	-0.02	-0.74	0.6	-0.97
pPropelStride_RF	0.17	-0.54	-0.92	1.12
pPropelStride_RH	0.98	-0.12	0.45	-0.18
pSharedStance_LH	0.39	-1	0.29	0.06
pSharedStance_RH	-0.81	-0.15	-0.53	-0.07
pStanceStride_LF	0.34	0.34	0.38	0.8
pStanceStride_LH	0.09	0.42	0.1	-0.21
pStanceStride_RF	1.42	0.57	-0.02	1.69
pStanceStride_RH	1.01	0.1	-0.16	0.23
pSwingStride_LF	-0.34	-0.34	-0.38	-0.8
pSwingStride_LH	-0.09	-0.42	-0.1	0.21
pSwingStride_RF	-1.42	-0.57	0.02	-1.69

	CC041XCC012	CC051	CC057	CC078
pSwingStride_RH	-1.43	-0.28	0.05	-0.45
AbsolutePawAngle_LF	-0.87	0.95	1.37	0.28
AbsolutePawAngle_LH	-1.8	1.87	0.27	-0.58
AbsolutePawAngle_RF	-2.19	0.67	1.06	-0.28
AbsolutePawAngle_RH	-2.61	0.54	-0.06	1.14
AtaxiaCoefficient_LF	-1.44	-0.24	1.22	0.33
AtaxiaCoefficient_LH	-0.79	-0.35	2.1	1
AtaxiaCoefficient_RF	-1.83	0.22	1.52	-0.73
AtaxiaCoefficient_RH	-1.18	-0.33	0.49	0.63
AxisDistance_LF	-0.56	0.68	0.25	-0.04
AxisDistance_LH	0.62	-0.07	0.67	-0.21
AxisDistance_RF	-0.16	1.54	0.25	-0.56
AxisDistance_RH	0.37	0.6	0.18	-0.18
Brake_LF	0.94	0.35	0.4	0.79
Brake_LH	0.64	1.26	-1.88	0.83
Brake_RF	1.91	0.56	-0.73	0.55
Brake_RH	0.59	0.34	-0.44	0.52
GaitSymmetry_LF	-0.95	-0.81	-0.04	-0.71
GaitSymmetry_LH	-0.97	-0.81	-0.04	-0.71
GaitSymmetry_RF	-0.97	-0.81	-0.04	-0.71
GaitSymmetry_RH	-1.05	-0.89	-0.1	-0.78
HindLimbSharedStanceTime_LH	0.28	0.23	0.19	0.24
MAXdAratiodT_LF	2.01	-0.3	-1.29	-0.36
MAXdAratiodT_LH	1.37	-0.22	-0.91	-0.37
MAXdAratiodT_RF	1.88	-1.02	-1.86	-0.02
MAXdAratiodT_RH	1.85	-0.18	-1.21	-0.96
MidlineDistance_LF	-0.13	-0.1	0.6	-0.23
MidlineDistance_LH	0.23	0.99	-0.95	0.25
MidlineDistance_RF	0.35	1.21	0.55	-0.46
MidlineDistance_RH	0.88	0.61	-0.77	0.51

	CC041XCC012	CC051	CC057	CC078
MINdAratiodT_LF	0.41	1.48	-1.01	-0.11
MINdAratiodT_LH	0.64	-0.08	-0.26	-0.51
MINdAratiodT_RF	0.92	0.29	-0.51	0.15
MINdAratiodT_RH	-0.73	-0.12	0.43	-0.91
OverlapDistance_LF	-0.6	0.45	-0.45	0.18
OverlapDistance_LH	-0.63	0.47	-0.43	0.21
OverlapDistance_RF	-0.24	1.2	-1.18	-0.08
OverlapDistance_RH	-0.27	1.17	-1.21	-0.11
PawAngle_LF	0.92	-0.76	-1.41	0.08
PawAngle_LH	1.52	-1.98	-0.53	0.24
PawAngle_RF	-1.83	1.89	0.51	-0.07
PawAngle_RH	-2.45	0.46	-0.08	1
PawAngleVariability_LF	-1.56	0.61	0.36	0.74
PawAngleVariability_LH	-0.38	-0.37	0.74	0.45
PawAngleVariability_RF	-1.17	0.11	1.21	-0.7
PawAngleVariability_RH	-0.58	-0.44	-0.18	-0.51
PawAreaatPeakStanceinsq.cm_LF	2.19	-0.39	-1.12	-0.27
PawAreaatPeakStanceinsq.cm_LH	1.39	-0.17	-1.21	-0.17
PawAreaatPeakStanceinsq.cm_RF	2.23	-0.89	-1.48	-0.23
PawAreaatPeakStanceinsq.cm_RH	1.96	0.01	-1.51	-0.71
PawAreaVariabilityatPeakStanceinsq.cm_LF	-0.33	-0.67	-0.49	-0.06
PawAreaVariabilityatPeakStanceinsq.cm_LH	0.55	-1.47	1.24	-0.1
PawAreaVariabilityatPeakStanceinsq.cm_RF	-0.91	-0.11	0.59	-0.41
PawAreaVariabilityatPeakStanceinsq.cm_RH	-0.19	-0.35	-0.44	-0.5
PawDrag_LH	-0.76	-0.36	1.38	-0.65
PawDrag_RH	-0.23	-0.69	0.47	-0.38
PawPlacementPositioning[PPP]_LF	-2.19	0.54	-0.24	1.22
PawPlacementPositioning[PPP]_LH	-2.21	0.54	-0.24	1.23
PawPlacementPositioning[PPP]_RF	1.1	-1.27	-0.74	-0.08
PawPlacementPositioning[PPP]_RH	1.07	-1.26	-0.74	-0.09

	CC041XCC012	CC051	CC057	CC078
Propel_LF	1.59	0.93	-2.19	0.02
Propel_LH	0.83	-0.51	-1.19	-0.53
Propel_RF	1.25	-0.29	-1.97	0.87
Propel_RH	1.28	0.15	-0.2	0.05
SLVar_LF	-1.12	-0.52	0.19	0.64
SLVar_LH	-0.12	-1.49	1.73	0.64
SLVar_RF	-0.88	-0.83	0.95	-0.24
SLVar_RH	-0.61	-1.47	-0.62	0.34
Stance_LF	1.73	0.91	-1.56	0.4
Stance_LH	1.11	0.37	-2.18	0.06
Stance_RF	1.79	0.13	-1.62	0.82
Stance_RH	0.84	0.24	-0.14	0.25
StanceratioSwing_LF	0.24	0.33	0.62	0.7
StanceratioSwing_LH	0.36	0.41	0.32	-0.29
StanceratioSwing_RF	1.24	0.67	0.41	1.69
StanceratioSwing_RH	1.15	0.12	-0.11	0.21
StanceFactor_LF	-1.56	1.38	0.55	-1.11
StanceFactor_LH	-1.03	0.28	-0.61	-0.13
StanceWidth_LF	0.3	2.5	-0.11	-0.86
StanceWidth_LH	-0.13	0.61	0.01	0.28
StanceWidthCV_LF	-2.14	-0.69	1.02	-0.3
StanceWidthCV_LH	-0.37	0.39	0.6	0.15
StepAngle_LF	-0.84	-0.44	-0.86	1.43
StepAngle_LH	-1.3	-1.55	-0.26	0.47
StepAngleCV_LF	0.14	-1.25	1.21	0.18
StepAngleCV_LH	1	0.07	-0.3	-0.14
StepAngleVar_LF	-0.13	-1.46	0.46	0.44
StepAngleVar_LH	0.4	-0.68	-0.44	0.23
Stride_LF	1.65	0.91	-1.56	0.21
Stride_LH	1.23	0.22	-2.45	0.1

	CC041XCC012	CC051	CC057	CC078
Stride_RF	1.68	-0.02	-1.58	0.52
Stride_RH	0.78	0.22	-0.22	0.21
StrideFrequency_LF	-1.15	-1.05	1.34	-0.34
StrideFrequency_LH	-1.19	-0.55	2.31	-0.13
StrideFrequency_RF	-1.34	-0.04	1.14	-0.51
StrideFrequency_RH	-1.05	-0.06	1.58	0
StrideLength_LF	1.75	-0.03	-1.4	0.36
StrideLength_LH	1.35	-1.41	-2.17	0.34
StrideLength_RF	1.73	-0.79	-1.44	0.59
StrideLength_RH	1.34	-0.61	-0.53	0.26
StrideLengthCV_LF	-1.68	-0.46	0.91	-0.04
StrideLengthCV_LH	-0.78	-0.82	2.15	0.31
StrideLengthCV_RF	-1.9	-0.1	1.45	-0.72
StrideLengthCV_RH	-1.36	-0.62	0.17	0.22
Swing_LF	1.28	0.74	-1.36	-0.21
Swing_LH	1.02	-0.19	-2.07	0.16
Swing_RF	1.16	-0.37	-1.25	-0.31
Swing_RH	0.24	0.23	0.22	0.23
SwingDurationCV_LF	-0.78	-1.37	0.85	0.16
SwingDurationCV_LH	-1	-1.52	1.55	0.03
SwingDurationCV_RF	-1.98	-0.23	1.39	-0.77
SwingDurationCV_RH	-1.23	-0.31	0.57	-0.02
SWVar_LF	-2.53	0.5	1.57	-0.6
SWVar_LH	-0.58	0.84	0.84	0.32

Table S6. Post-normalization DigiGait data is listed for all DigiGait parameters and strains. LF = left fore limb, LH = left hind limb, RF = right fore limb, RH = right hind limb.

Table S7

strain	sequence	baseMean	log2FoldChange	lfcSE	stat	pvalue	padj	threshold	H2 haplotype
CC002	AAA47930	110.2772	-1.522750882	25.437	-0.059864	0.952264	0.998574	0	*b*
CC005	AAA47930	110.2772	21.90465089	23.24061	0.942516	0.345928	0.999981	0	*z*
CC006	AAA47930	110.2772	23.98200402	23.12826	1.036913	0.299776	0.999982	0	*het*
CC011	AAA47930	110.2772	22.5519973	23.12921	0.975044	0.329539	0.91203	0	*g7*
CC015	AAA47930	110.2772	32.3817353	23.64777	1.369336	0.170894	0.87209	0	*WSB/EiJ x a*
CC017	AAA47930	110.2772	27.55962531	23.71757	1.161992	0.245239	0.999954	0	*CAST/EiJ*
CC023	AAA47930	110.2772	20.95029657	24.72297	0.847402	0.396771	0.999855	0	*b*
CC025	AAA47930	110.2772	-1.24269009	24.48641	-0.05075	0.959525	0.999785	0	*PWK/PhJ*
CC027	AAA47930	110.2772	24.72110375	23.71842	1.042274	0.297285	0.999947	0	*het*
CC032xCC013	AAA47930	110.2772	-3.59503499	23.6652	-0.151912	0.879256	0.95573	0	*b x a*
CC037	AAA47930	110.2772	21.52961081	22.97444	0.937112	0.348701	0.999351	0	*b*
CC041xCC012	AAA47930	110.2772	20.84597717	22.83799	0.912776	0.36136	0.763196	0	*b x WSB/EiJ*
CC051	AAA47930	110.2772	-2.665830301	23.05183	-0.115645	0.907934	0.999877	0	*b x a*
sexM_effect	AAA47930	110.2772	14.85360648	24.3668	0.609584	0.542138	0.999998	0	
tissueS_effect	AAA47930	110.2772	11.00627929	24.85006	0.442908	0.657833	0.808695	0	

Table S7. Levels of TMEV RNA were measured in infected and uninfected mice for all strains listed here,
using the sequence for TMEV polyprotein AAA47930. Log2FoldChange values were calculated for infected vs uninfected mice.
lfcSE = log fold change standard error.

File S1. Graphs and statistical data by strain, sex, and infection status for each of the following phenotypes: Clonus, Encephalitis, Hunch, Paralysis, Paresis (labeled as Weakness), Ruffledness, and Seizure. Labels indicate which limb is associated with the data, where appropriate: FL for left fore limb, FR for right fore limb, HL for left hind limb, and HR for right hind limb.

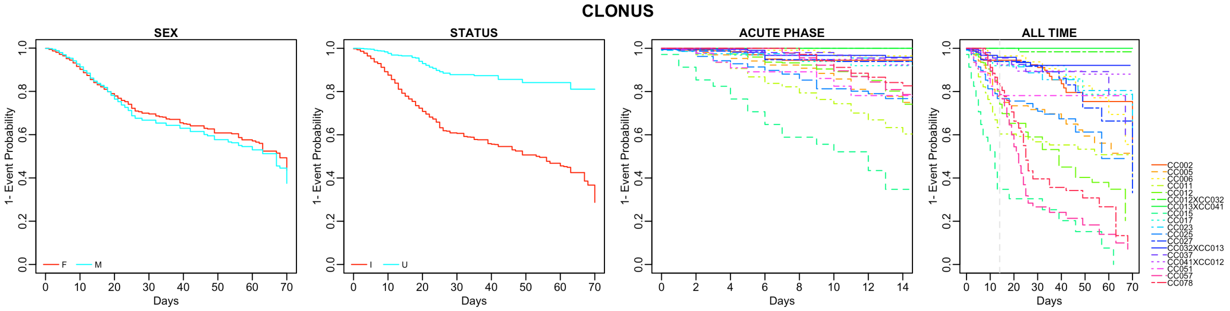


```
Call: survfit(formula = Surv(Date.dpi, as.numeric(Clonus)) ~ Sex, data = aData)
```

Sex=F

time	n.risk	n.event	survival	std.err	lower 95% CI	upper 95% CI
0	2190	2	0.999	0.000645	0.998	1.000
1	2084	11	0.994	0.001711	0.990	0.997
2	1993	14	0.987	0.002518	0.982	0.992
3	1883	12	0.981	0.003088	0.975	0.987
4	1778	17	0.971	0.003805	0.964	0.979
5	1678	12	0.964	0.004273	0.956	0.973
6	1583	21	0.951	0.005047	0.942	0.961
7	1480	11	0.944	0.005441	0.934	0.955
8	1383	19	0.931	0.006126	0.919	0.943
9	1272	22	0.915	0.006916	0.902	0.929
10	1154	17	0.902	0.007548	0.887	0.917
11	1055	18	0.886	0.008245	0.870	0.903
12	965	14	0.874	0.008812	0.856	0.891
13	879	19	0.855	0.009628	0.836	0.874
14	799	13	0.841	0.010214	0.821	0.861
15	736	9	0.830	0.010649	0.810	0.852
16	696	8	0.821	0.011048	0.800	0.843
17	667	9	0.810	0.011500	0.788	0.833
18	645	9	0.799	0.011940	0.775	0.822
19	617	7	0.789	0.012286	0.766	0.814
20	597	8	0.779	0.012678	0.754	0.804
21	580	8	0.768	0.013060	0.743	0.794
22	540	7	0.758	0.013422	0.732	0.785
23	523	4	0.752	0.013629	0.726	0.780
24	498	8	0.740	0.014064	0.713	0.768
25	481	12	0.722	0.014689	0.694	0.751
26	457	8	0.709	0.015096	0.680	0.739
27	441	2	0.706	0.015198	0.677	0.736
28	422	4	0.699	0.015418	0.670	0.730
30	387	2	0.696	0.015548	0.666	0.727
32	376	5	0.686	0.015883	0.656	0.718
33	366	2	0.683	0.016016	0.652	0.715
34	357	1	0.681	0.016085	0.650	0.713
35	335	4	0.673	0.016398	0.641	0.706
36	310	1	0.670	0.016488	0.639	0.704
39	301	7	0.655	0.017126	0.622	0.689
40	291	2	0.650	0.017301	0.617	0.685
41	282	1	0.648	0.017393	0.615	0.683
42	263	3	0.641	0.017710	0.607	0.676
46	224	4	0.629	0.018295	0.594	0.666
47	214	1	0.626	0.018444	0.591	0.664

```
   49    205       6    0.608 0.019363        0.571        0.647
   54    158       1    0.604 0.019619        0.567        0.644
   56    137       4    0.586 0.020935        0.547        0.629
   57    113       2    0.576 0.021813        0.535        0.620
   60    111       1    0.571 0.022226        0.529        0.616
   61    103       1    0.565 0.022690        0.523        0.612
   62     99       1    0.560 0.023169        0.516        0.607
   63     94       6    0.524 0.025876        0.476        0.577
   67     56       1    0.515 0.027053        0.464        0.570
   68     48       2    0.493 0.029873        0.438        0.555
   70     17       2    0.435 0.046688        0.353        0.537

                Sex=M
 time n.risk n.event survival  std.err lower 95% CI upper 95% CI
    0   2010       4    0.998 0.000994        0.996        1.000
    1   1919       1    0.997 0.001121        0.995        1.000
    2   1838       6    0.994 0.001735        0.991        0.998
    3   1734      11    0.988 0.002562        0.983        0.993
    4   1633      14    0.979 0.003396        0.973        0.986
    5   1540      16    0.969 0.004207        0.961        0.978
    6   1454      18    0.957 0.005016        0.947        0.967
    7   1356       6    0.953 0.005284        0.943        0.963
    8   1270      16    0.941 0.006010        0.929        0.953
    9   1169      18    0.927 0.006819        0.913        0.940
   10   1058      15    0.913 0.007519        0.899        0.928
   11    964      19    0.895 0.008429        0.879        0.912
   12    883      11    0.884 0.008970        0.867        0.902
   13    809      21    0.861 0.010039        0.842        0.881
   14    730      12    0.847 0.010673        0.826        0.868
   15    669       8    0.837 0.011130        0.815        0.859
   16    631       7    0.828 0.011547        0.805        0.851
   17    607       7    0.818 0.011964        0.795        0.842
   18    587      14    0.799 0.012765        0.774        0.824
   19    558      12    0.781 0.013419        0.756        0.808
   20    539      12    0.764 0.014029        0.737        0.792
   21    525       8    0.752 0.014406        0.725        0.781
   22    487      10    0.737 0.014916        0.708        0.767
   23    473       7    0.726 0.015254        0.697        0.757
   24    452       9    0.712 0.015693        0.682        0.743
   25    438      15    0.687 0.016369        0.656        0.720
   26    414       7    0.676 0.016671        0.644        0.709
   28    384       5    0.667 0.016912        0.635        0.701
   32    349       7    0.653 0.017312        0.620        0.688
   35    313       5    0.643 0.017653        0.609        0.679
   39    286       6    0.630 0.018122        0.595        0.666
   42    252       6    0.615 0.018695        0.579        0.652
   46    219       5    0.601 0.019292        0.564        0.640
   47    208       2    0.595 0.019534        0.558        0.634
   49    199       6    0.577 0.020271        0.538        0.618
   53    163       3    0.566 0.020804        0.527        0.608
   54    153       1    0.562 0.020994        0.523        0.605
   56    134       3    0.550 0.021747        0.509        0.594
   57    110       1    0.545 0.022116        0.503        0.590
   60    108       3    0.530 0.023164        0.486        0.577
   63     90       3    0.512 0.024533        0.466        0.563
   67     54       6    0.455 0.030907        0.398        0.520
   68     46       1    0.445 0.031780        0.387        0.512
   70     19       3    0.375 0.045869        0.295        0.477

Call: survfit(formula = Surv(Date.dpi, as.numeric(Clonus)) ~ Status,
```

```
   data = aData)

                Status=I
 time n.risk n.event survival  std.err lower 95% CI upper 95% CI
    0   2809       6    0.998 0.000871        0.996        1.000
    1   2667      12    0.993 0.001557        0.990        0.996
    2   2560      19    0.986 0.002287        0.982        0.990
    3   2420      21    0.977 0.002931        0.972        0.983
    4   2287      30    0.965 0.003712        0.957        0.972
    5   2165      27    0.953 0.004328        0.944        0.961
    6   2054      34    0.937 0.005031        0.927        0.947
    7   1926      15    0.930 0.005332        0.919        0.940
    8   1811      34    0.912 0.006014        0.900        0.924
    9   1669      37    0.892 0.006737        0.879        0.905
   10   1511      27    0.876 0.007281        0.862        0.890
   11   1380      32    0.856 0.007949        0.840        0.871
   12   1267      25    0.839 0.008479        0.822        0.856
   13   1165      39    0.811 0.009311        0.793        0.829
   14   1057      24    0.792 0.009828        0.773        0.812
   15    973      17    0.778 0.010214        0.759        0.799
   16    913      15    0.766 0.010566        0.745        0.787
   17    879      13    0.754 0.010867        0.733        0.776
   18    849      22    0.735 0.011356        0.713        0.757
   19    802      13    0.723 0.011643        0.700        0.746
   20    775      16    0.708 0.011985        0.685        0.732
   21    756      13    0.696 0.012245        0.672        0.720
   22    700      14    0.682 0.012552        0.658        0.707
   23    681       9    0.673 0.012741        0.648        0.698
   24    654      14    0.658 0.013037        0.633        0.684
   25    635      25    0.632 0.013515        0.607        0.660
   26    598      13    0.619 0.013749        0.592        0.646
   27    575       2    0.617 0.013785        0.590        0.644
   28    551       7    0.609 0.013924        0.582        0.637
   30    519       2    0.606 0.013969        0.580        0.634
   32    512      12    0.592 0.014231        0.565        0.621
   33    493       2    0.590 0.014274        0.562        0.618
   34    483       1    0.589 0.014297        0.561        0.617
   35    455       8    0.578 0.014506        0.550        0.607
   36    429       1    0.577 0.014535        0.549        0.606
   39    420      13    0.559 0.014905        0.531        0.589
   40    401       2    0.556 0.014960        0.528        0.586
   41    391       1    0.555 0.014989        0.526        0.585
   42    364       6    0.546 0.015200        0.517        0.576
   46    318       9    0.530 0.015617        0.500        0.562
   47    299       3    0.525 0.015760        0.495        0.557
   49    288      10    0.507 0.016232        0.476        0.540
   53    235       3    0.500 0.016449        0.469        0.534
   54    217       2    0.496 0.016617        0.464        0.529
   56    189       7    0.477 0.017390        0.444        0.513
   57    158       3    0.468 0.017829        0.435        0.504
   60    154       4    0.456 0.018374        0.421        0.493
   61    139       1    0.453 0.018532        0.418        0.491
   62    134       1    0.449 0.018699        0.414        0.488
   63    129       7    0.425 0.019826        0.388        0.466
   67     76       7    0.386 0.022863        0.344        0.433
   68     62       3    0.367 0.024164        0.323        0.418
   70     23       5    0.287 0.036808        0.224        0.369

                Status=U
 time n.risk n.event survival  std.err lower 95% CI upper 95% CI
```

```
    2   1271       1    0.999 0.000786        0.998        1.000
    3   1197       2    0.998 0.001417        0.995        1.000
    4   1124       1    0.997 0.001671        0.993        1.000
    5   1053       1    0.996 0.001919        0.992        0.999
    6    983       5    0.991 0.002958        0.985        0.996
    7    910       2    0.988 0.003328        0.982        0.995
    8    842       1    0.987 0.003525        0.980        0.994
    9    772       3    0.983 0.004149        0.975        0.992
   10    701       5    0.976 0.005171        0.966        0.987
   11    639       5    0.969 0.006157        0.957        0.981
   13    523       1    0.967 0.006418        0.954        0.980
   14    472       1    0.965 0.006723        0.952        0.978
   17    395       3    0.958 0.007892        0.942        0.973
   18    383       1    0.955 0.008258        0.939        0.971
   19    373       6    0.940 0.010233        0.920        0.960
   20    361       4    0.929 0.011367        0.907        0.952
   21    349       3    0.921 0.012169        0.898        0.945
   22    327       3    0.913 0.012999        0.888        0.939
   23    315       2    0.907 0.013548        0.881        0.934
   24    296       3    0.898 0.014412        0.870        0.927
   25    284       2    0.892 0.014988        0.863        0.921
   26    273       2    0.885 0.015574        0.855        0.916
   28    255       2    0.878 0.016207        0.847        0.910
   35    193       1    0.874 0.016749        0.841        0.907
   42    151       3    0.856 0.019181        0.819        0.895
   49    116       2    0.841 0.021503        0.800        0.885
   63     55       2    0.811 0.029672        0.755        0.871

Call: survfit(formula = Surv(Date.dpi, as.numeric(Clonus)) ~ Strain,
    data = aData)

                Strain=CC002
 time n.risk n.event survival std.err lower 95% CI upper 95% CI
    1    274       2    0.993 0.00514        0.983        1.000
    3    242       1    0.989 0.00656        0.976        1.000
    4    226       1    0.984 0.00785        0.969        1.000
    5    210       3    0.970 0.01117        0.949        0.992
    6    194       4    0.950 0.01476        0.922        0.980
    7    178       1    0.945 0.01561        0.915        0.976
   23     91       1    0.934 0.01857        0.899        0.972
   27     87       1    0.924 0.02124        0.883        0.966
   30     74       1    0.911 0.02434        0.865        0.960
   33     70       1    0.898 0.02726        0.846        0.953
   34     66       1    0.885 0.03005        0.828        0.945
   35     62       1    0.870 0.03278        0.808        0.937
   36     54       1    0.854 0.03591        0.787        0.928
   40     47       1    0.836 0.03948        0.762        0.917
   41     43       1    0.817 0.04309        0.736        0.906
   42     39       1    0.796 0.04679        0.709        0.893
   49     19       1    0.754 0.06022        0.645        0.882
   70      4       1    0.565 0.16933        0.314        1.000

                Strain=CC005
 time n.risk n.event survival std.err lower 95% CI upper 95% CI
    1    206       2    0.990 0.00683        0.977        1.000
    2    198       2    0.980 0.00976        0.961        1.000
    3    186       2    0.970 0.01217        0.946        0.994
    4    174       3    0.953 0.01532        0.923        0.984
    5    162       2    0.941 0.01724        0.908        0.976
    6    150       3    0.922 0.02003        0.884        0.963
```

8	126	2	0.908	0.02223	0.865	0.952
9	114	3	0.884	0.02557	0.835	0.935
10	102	3	0.858	0.02889	0.803	0.916
11	90	5	0.810	0.03426	0.746	0.880
12	78	2	0.789	0.03639	0.721	0.864
13	66	1	0.778	0.03775	0.707	0.855
14	56	2	0.750	0.04120	0.673	0.835
19	48	1	0.734	0.04320	0.654	0.824
26	42	1	0.717	0.04557	0.633	0.812
33	36	1	0.697	0.04846	0.608	0.798
40	30	1	0.674	0.05211	0.579	0.784
42	28	1	0.649	0.05552	0.549	0.768
47	24	1	0.622	0.05944	0.516	0.751
49	22	1	0.594	0.06311	0.482	0.732
54	18	1	0.561	0.06769	0.443	0.711
61	12	1	0.514	0.07651	0.384	0.688

Strain=CC006

time	n.risk	n.event	survival	std.err	lower 95% CI	upper 95% CI
3	218	2	0.991	0.00646	0.978	1.000
9	123	1	0.983	0.01027	0.963	1.000
10	109	2	0.965	0.01616	0.934	0.997
25	35	1	0.937	0.03138	0.878	1.000
32	30	1	0.906	0.04317	0.825	0.995
39	25	1	0.870	0.05457	0.769	0.984
46	20	1	0.826	0.06696	0.705	0.968
53	15	1	0.771	0.08208	0.626	0.950
60	10	1	0.694	0.10397	0.517	0.931
67	5	1	0.555	0.14944	0.328	0.941
70	3	1	0.370	0.18100	0.142	0.965

Strain=CC011

time	n.risk	n.event	survival	std.err	lower 95% CI	upper 95% CI
1	240	3	0.988	0.00717	0.974	1.000
2	230	4	0.970	0.01105	0.949	0.992
3	216	8	0.934	0.01639	0.903	0.967
4	202	6	0.907	0.01943	0.869	0.946
5	187	8	0.868	0.02293	0.824	0.914
6	173	6	0.838	0.02522	0.790	0.889
7	159	3	0.822	0.02634	0.772	0.875
8	145	5	0.794	0.02832	0.740	0.851
9	131	5	0.763	0.03030	0.706	0.825
10	117	3	0.744	0.03156	0.684	0.808
11	103	6	0.700	0.03432	0.636	0.771
12	89	4	0.669	0.03621	0.602	0.744
13	75	4	0.633	0.03842	0.562	0.713
14	64	3	0.604	0.04026	0.530	0.688
21	53	1	0.592	0.04108	0.517	0.678
26	49	1	0.580	0.04198	0.503	0.669
28	46	1	0.567	0.04292	0.489	0.658
35	39	1	0.553	0.04422	0.473	0.647
47	28	1	0.533	0.04684	0.449	0.633
54	21	1	0.508	0.05103	0.417	0.618

Strain=CC012

time	n.risk	n.event	survival	std.err	lower 95% CI	upper 95% CI
2	420	1	0.998	0.00238	0.993	1.000
4	372	9	0.973	0.00828	0.957	0.990
5	348	5	0.959	0.01025	0.940	0.980
6	324	8	0.936	0.01298	0.911	0.962

```
    7    300       1    0.933 0.01331        0.907        0.959
    8    276       3    0.923 0.01439        0.895        0.951
    9    252       5    0.904 0.01627        0.873        0.937
   10    228       3    0.892 0.01744        0.859        0.927
   11    204       5    0.870 0.01957        0.833        0.910
   12    180       4    0.851 0.02139        0.810        0.894
   13    156       9    0.802 0.02567        0.753        0.854
   14    132      10    0.741 0.03006        0.685        0.803
   15    120       7    0.698 0.03245        0.637        0.765
   18    108       7    0.653 0.03456        0.588        0.724
   25     96       6    0.612 0.03619        0.545        0.687
   26     83       3    0.590 0.03707        0.522        0.667
   32     71       7    0.532 0.03940        0.460        0.615
   39     59       9    0.451 0.04164        0.376        0.540
   46     47       5    0.403 0.04237        0.328        0.495
   53     35       2    0.380 0.04296        0.304        0.474
   60     24       2    0.348 0.04483        0.270        0.448
   67     12       5    0.203 0.05601        0.118        0.349

                Strain=CC012XCC032 
        time        n.risk      n.event     survival      std.err lower 95% CI upper 
95% CI 
      22.000        62.000        1.000        0.984        0.016        0.953 
1.000 

                Strain=CC013XCC041 
     time n.risk n.event survival std.err lower 95% CI upper 95% CI

                Strain=CC015 
 time n.risk n.event survival std.err lower 95% CI upper 95% CI
    0     35       1    0.971  0.0282       0.9178        1.000
    1     33       2    0.913  0.0482       0.8227        1.000
    2     31       2    0.854  0.0605       0.7430        0.981
    3     29       1    0.824  0.0652       0.7059        0.962
    4     28       2    0.765  0.0726       0.6355        0.922
    5     26       2    0.706  0.0780       0.5690        0.877
    6     24       2    0.648  0.0819       0.5054        0.830
    7     22       2    0.589  0.0844       0.4446        0.780
    9     18       1    0.556  0.0858       0.4109        0.752
   10     16       1    0.521  0.0872       0.3756        0.723
   12     12       2    0.434  0.0918       0.2871        0.657
   13     10       2    0.348  0.0917       0.2072        0.583
   18      8       1    0.304  0.0899       0.1703        0.543
   32      6       1    0.253  0.0881       0.1282        0.501
   39      5       1    0.203  0.0838       0.0902        0.456
   46      4       1    0.152  0.0767       0.0566        0.408
   57      2       1    0.076  0.0660       0.0139        0.417
   62      1       1    0.000     NaN           NA           NA

                Strain=CC017 
 time n.risk n.event survival std.err lower 95% CI upper 95% CI
    2    105       1    0.990 0.00948        0.972        1.000
    4     90       2    0.968 0.01796        0.934        1.000
    8     66       2    0.939 0.02685        0.888        0.993
   11     48       1    0.920 0.03265        0.858        0.986
   42     15       1    0.858 0.06661        0.737        0.999
   49     12       1    0.787 0.09174        0.626        0.989

                Strain=CC023 
 time n.risk n.event survival std.err lower 95% CI upper 95% CI
```

time	n.risk	n.event	survival	std.err	lower 95% CI	upper 95% CI
0	209	2	0.990	0.00673	0.977	1.000
1	201	1	0.986	0.00831	0.969	1.000
2	189	2	0.975	0.01102	0.954	0.997
8	119	1	0.967	0.01364	0.941	0.994
11	83	1	0.955	0.01776	0.921	0.991
13	59	1	0.939	0.02372	0.894	0.987
18	38	1	0.914	0.03359	0.851	0.983
25	33	1	0.887	0.04249	0.807	0.974
32	28	1	0.855	0.05143	0.760	0.962
47	17	1	0.805	0.06873	0.681	0.951
70	4	1	0.603	0.18168	0.335	1.000

Strain=CC025

time	n.risk	n.event	survival	std.err	lower 95% CI	upper 95% CI
0	173	1	0.994	0.00576	0.983	1.000
1	164	1	0.988	0.00833	0.972	1.000
2	154	4	0.962	0.01504	0.933	0.992
3	144	3	0.942	0.01866	0.907	0.980
4	135	2	0.928	0.02083	0.889	0.970
5	125	2	0.914	0.02299	0.870	0.960
6	115	2	0.898	0.02519	0.850	0.948
7	105	2	0.881	0.02746	0.828	0.936
8	95	3	0.853	0.03093	0.794	0.916
9	85	4	0.813	0.03539	0.746	0.885
11	73	1	0.802	0.03662	0.733	0.877
12	71	1	0.790	0.03780	0.720	0.868
13	69	2	0.767	0.04002	0.693	0.850
18	67	1	0.756	0.04103	0.680	0.841
25	66	1	0.744	0.04198	0.667	0.831
28	65	2	0.722	0.04370	0.641	0.812
30	57	1	0.709	0.04473	0.626	0.802
32	53	1	0.696	0.04584	0.611	0.791
39	32	1	0.674	0.04929	0.584	0.778
46	11	1	0.613	0.07361	0.484	0.775
57	5	1	0.490	0.12440	0.298	0.806

Strain=CC027

time	n.risk	n.event	survival	std.err	lower 95% CI	upper 95% CI
0	246	1	0.996	0.00406	0.988	1.000
2	223	2	0.987	0.00746	0.972	1.000
4	198	1	0.982	0.00894	0.965	1.000
6	170	6	0.947	0.01635	0.916	0.980
10	115	1	0.939	0.01817	0.904	0.975
25	42	1	0.917	0.02833	0.863	0.974
32	36	1	0.891	0.03727	0.821	0.967
39	30	1	0.862	0.04638	0.775	0.957
42	28	1	0.831	0.05398	0.731	0.944
46	24	1	0.796	0.06184	0.684	0.927
49	22	2	0.724	0.07444	0.592	0.885
57	12	1	0.663	0.08940	0.510	0.864
70	4	2	0.332	0.17179	0.120	0.915

Strain=CC032XCC013

time	n.risk	n.event	survival	std.err	lower 95% CI	upper 95% CI
0	262	1	0.996	0.00381	0.989	1.000
5	179	1	0.991	0.00672	0.978	1.000
6	169	4	0.967	0.01331	0.941	0.994
13	109	1	0.958	0.01587	0.928	0.990
21	80	2	0.934	0.02279	0.891	0.980
27	70	1	0.921	0.02608	0.871	0.974

Strain=CC037

time	n.risk	n.event	survival	std.err	lower 95% CI	upper 95% CI
3	168	1	0.994	0.00593	0.982	1.000
5	144	2	0.980	0.01132	0.958	1.000
10	84	1	0.969	0.01612	0.937	1.000
11	72	1	0.955	0.02076	0.915	0.997
19	32	1	0.925	0.03560	0.858	0.998
25	28	1	0.892	0.04724	0.804	0.990
60	8	1	0.781	0.11222	0.589	1.000
67	4	1	0.586	0.18882	0.311	1.000

Strain=CC041XCC012

time	n.risk	n.event	survival	std.err	lower 95% CI	upper 95% CI
4	237	2	0.992	0.00594	0.980	1.000
5	225	1	0.987	0.00737	0.973	1.000
6	214	4	0.969	0.01166	0.946	0.992
8	193	4	0.949	0.01513	0.919	0.979
13	149	4	0.923	0.01935	0.886	0.962
21	128	4	0.894	0.02352	0.849	0.942
49	64	1	0.880	0.02698	0.829	0.935

Strain=CC051

time	n.risk	n.event	survival	std.err	lower 95% CI	upper 95% CI
1	138	1	0.993	0.00722	0.979	1.000
2	128	2	0.977	0.01300	0.952	1.000
3	118	5	0.936	0.02198	0.894	0.980
4	108	3	0.910	0.02600	0.860	0.962
5	98	2	0.891	0.02859	0.837	0.949
9	58	2	0.861	0.03490	0.795	0.932
10	48	2	0.825	0.04165	0.747	0.910
11	38	2	0.781	0.04949	0.690	0.885

Strain=CC057

time	n.risk	n.event	survival	std.err	lower 95% CI	upper 95% CI
7	364	8	0.9780	0.00768	0.9631	0.993
8	354	8	0.9559	0.01077	0.9350	0.977
9	336	12	0.9218	0.01420	0.8944	0.950
10	318	9	0.8957	0.01624	0.8644	0.928
11	300	9	0.8688	0.01806	0.8341	0.905
12	282	8	0.8442	0.01953	0.8067	0.883
13	264	11	0.8090	0.02141	0.7681	0.852
14	248	7	0.7862	0.02248	0.7433	0.831
15	232	6	0.7658	0.02338	0.7214	0.813
16	216	10	0.7304	0.02484	0.6833	0.781
17	200	13	0.6829	0.02648	0.6329	0.737
18	184	10	0.6458	0.02752	0.5940	0.702
19	168	12	0.5997	0.02860	0.5462	0.658
20	152	14	0.5444	0.02953	0.4895	0.605
21	136	8	0.5124	0.02988	0.4571	0.574
22	120	13	0.4569	0.03035	0.4011	0.520
23	104	8	0.4217	0.03046	0.3661	0.486
24	88	11	0.3690	0.03052	0.3138	0.434
25	72	10	0.3178	0.03028	0.2636	0.383
26	56	6	0.2837	0.03006	0.2305	0.349
28	49	3	0.2664	0.02984	0.2138	0.332
35	42	4	0.2410	0.02957	0.1895	0.307
42	35	4	0.2134	0.02922	0.1632	0.279
49	28	4	0.1830	0.02875	0.1345	0.249
56	21	5	0.1394	0.02773	0.0944	0.206

```
   63     14       4   0.0996 0.02599       0.0597        0.166
   68      7       2   0.0711 0.02517       0.0355        0.142

                Strain=CC078
 time n.risk n.event survival std.err lower 95% CI upper 95% CI
    8    245       7    0.971  0.0106       0.9508        0.993
    9    231       7    0.942  0.0151       0.9129        0.972
   10    217       7    0.912  0.0184       0.8762        0.948
   11    203       6    0.885  0.0209       0.8446        0.927
   12    190       4    0.866  0.0225       0.8231        0.911
   13    178       5    0.842  0.0243       0.7954        0.891
   14    166       3    0.826  0.0254       0.7782        0.878
   15    154       4    0.805  0.0269       0.7540        0.860
   16    142       5    0.777  0.0288       0.7222        0.835
   17    130       3    0.759  0.0299       0.7023        0.820
   18    119       3    0.740  0.0312       0.6810        0.803
   19    109       5    0.706  0.0332       0.6435        0.774
   20     99       6    0.663  0.0355       0.5969        0.736
   21     89       1    0.655  0.0359       0.5888        0.730
   22     79       3    0.631  0.0373       0.5616        0.708
   23     69       2    0.612  0.0384       0.5415        0.692
   24     59       6    0.550  0.0421       0.4735        0.639
   25     49       6    0.483  0.0450       0.4021        0.579
   26     39       4    0.433  0.0467       0.3507        0.535
   28     35       3    0.396  0.0474       0.3133        0.501
   35     30       3    0.356  0.0478       0.2740        0.464
   42     25       1    0.342  0.0480       0.2599        0.450
   49     20       2    0.308  0.0489       0.2256        0.420
   56     15       2    0.267  0.0503       0.1845        0.386
   63     10       5    0.133  0.0491       0.0649        0.275
   68      5       1    0.107  0.0460       0.0459        0.248

Call:
coxph(formula = Surv(Date.dpi, as.numeric(Clonus)) ~ Sex, data = aData)

  n= 4200, number of events= 755 

        coef exp(coef) se(coef)     z Pr(>|z|)
SexM 0.04307   1.04402  0.07281 0.592    0.554

     exp(coef) exp(-coef) lower .95 upper .95
SexM     1.044     0.9578    0.9052     1.204

Concordance= 0.494  (se = 0.011 )
Likelihood ratio test= 0.35  on 1 df,   p=0.6
Wald test            = 0.35  on 1 df,   p=0.6
Score (logrank) test = 0.35  on 1 df,   p=0.6

Call:
coxph(formula = Surv(Date.dpi, as.numeric(Clonus)) ~ Status, 
    data = aData)

  n= 4200, number of events= 755 

           coef exp(coef) se(coef)      z Pr(>|z|)    
StatusU -1.5589    0.2104   0.1280 -12.18   <2e-16 ***
---
Signif. codes:  0 ‚Äò***‚Äô 0.001 ‚Äò**‚Äô 0.01 ‚Äò*‚Äô 0.05 ‚Äò.‚Äô 0.1 ‚Äò ‚Äô 1

        exp(coef) exp(-coef) lower .95 upper .95
```

```
StatusU    0.2104      4.753    0.1637    0.2704

Concordance= 0.622  (se = 0.006 )
Likelihood ratio test= 225.1  on 1 df,   p=<2e-16
Wald test            = 148.3  on 1 df,   p=<2e-16
Score (logrank) test = 180.9  on 1 df,   p=<2e-16

Call:
coxph(formula = Surv(Date.dpi, as.numeric(Clonus)) ~ Strain,
    data = Z)

  n= 4396, number of events= 774

                       coef  exp(coef)   se(coef)      z Pr(>|z|)
StrainCC078        1.673e+00  5.331e+00  2.500e-01  6.693 2.19e-11 ***
StrainCC057        2.034e+00  7.643e+00  2.397e-01  8.484  < 2e-16 ***
StrainCC012        1.563e+00  4.775e+00  2.480e-01  6.303 2.92e-10 ***
StrainCC041XCC012 -2.295e-01  7.950e-01  3.210e-01 -0.715  0.47475
StrainCC002        2.659e-01  1.305e+00  3.074e-01  0.865  0.38714
StrainCC025        1.169e+00  3.219e+00  2.824e-01  4.140 3.47e-05 ***
StrainCC012XCC032 -2.743e+00  6.439e-02  1.026e+00 -2.673  0.00752 **
StrainCC013XCC041 -1.578e+01  1.401e-07  7.389e+02 -0.021  0.98296
StrainCC032XCC013 -5.385e-01  5.836e-01  3.912e-01 -1.377  0.16866
StrainCC015        2.715e+00  1.511e+01  3.022e-01  8.984  < 2e-16 ***
StrainCC023        2.169e-01  1.242e+00  3.607e-01  0.601  0.54770
StrainCC027        5.023e-01  1.652e+00  3.173e-01  1.583  0.11348
StrainCC005        1.219e+00  3.385e+00  2.785e-01  4.378 1.20e-05 ***
StrainCC011        1.673e+00  5.329e+00  2.582e-01  6.481 9.14e-11 ***
StrainCC051        1.354e+00  3.874e+00  3.254e-01  4.162 3.15e-05 ***
StrainCC037        3.108e-02  1.032e+00  4.051e-01  0.077  0.93886
StrainCC006        1.059e-01  1.112e+00  3.606e-01  0.294  0.76911
StrainCC017        2.216e-01  1.248e+00  4.224e-01  0.525  0.59980
---
Signif. codes:  0 ,Äò***,Äô 0.001 ,Äò**,Äô 0.01 ,Äò*,Äô 0.05 ,Äò.,Äô 0.1 ,Äò ,Äô 1

                  exp(coef) exp(-coef) lower .95 upper .95
StrainCC078       5.331e+00  1.876e-01  3.265533    8.7019
StrainCC057       7.643e+00  1.308e-01  4.777419   12.2266
StrainCC012       4.775e+00  2.094e-01  2.936548    7.7642
StrainCC041XCC012 7.950e-01  1.258e+00  0.423719    1.4915
StrainCC002       1.305e+00  7.666e-01  0.714155    2.3830
StrainCC025       3.219e+00  3.107e-01  1.850622    5.5976
StrainCC012XCC032 6.439e-02  1.553e+01  0.008616    0.4812
StrainCC013XCC041 1.401e-07  7.140e+06  0.000000       Inf
StrainCC032XCC013 5.836e-01  1.713e+00  0.271131    1.2564
StrainCC015       1.511e+01  6.619e-02  8.354992   27.3196
StrainCC023       1.242e+00  8.050e-01  0.612541    2.5191
StrainCC027       1.652e+00  6.052e-01  0.887177    3.0778
StrainCC005       3.385e+00  2.954e-01  1.960842    5.8429
StrainCC011       5.329e+00  1.876e-01  3.212840    8.8395
StrainCC051       3.874e+00  2.581e-01  2.047538    7.3310
StrainCC037       1.032e+00  9.694e-01  0.466274    2.2822
StrainCC006       1.112e+00  8.996e-01  0.548285    2.2539
StrainCC017       1.248e+00  8.012e-01  0.545413    2.8560

Concordance= 0.707  (se = 0.009 )
Likelihood ratio test= 637.3  on 18 df,   p=<2e-16
Wald test            = 405.8  on 18 df,   p=<2e-16
Score (logrank) test = 650.1  on 18 df,   p=<2e-16
```

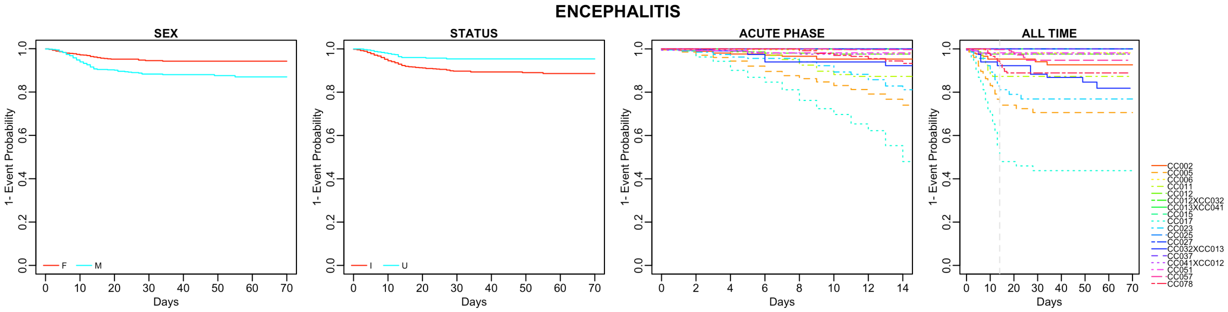


```
Call: survfit(formula = Surv(Date.dpi, as.numeric(Encephalitis)) ~
    Sex, data = aData)
```

Sex=F

time	n.risk	n.event	survival	std.err	lower 95% CI	upper 95% CI
0	2190	2	0.999	0.000645	0.998	1.000
1	2084	5	0.997	0.001249	0.994	0.999
2	1993	8	0.993	0.001882	0.989	0.996
3	1883	7	0.989	0.002335	0.984	0.994
4	1778	5	0.986	0.002639	0.981	0.991
5	1678	6	0.983	0.002997	0.977	0.989
6	1583	5	0.980	0.003293	0.973	0.986
7	1480	2	0.978	0.003419	0.972	0.985
8	1383	2	0.977	0.003558	0.970	0.984
9	1272	4	0.974	0.003864	0.966	0.981
10	1154	3	0.971	0.004121	0.963	0.979
11	1055	1	0.970	0.004219	0.962	0.979
12	965	2	0.968	0.004443	0.960	0.977
13	879	4	0.964	0.004939	0.954	0.974
14	799	2	0.961	0.005213	0.951	0.972
15	736	2	0.959	0.005516	0.948	0.970
16	696	2	0.956	0.005835	0.945	0.968
17	667	1	0.955	0.005999	0.943	0.967
18	645	1	0.953	0.006170	0.941	0.965
19	617	1	0.952	0.006350	0.939	0.964
27	441	2	0.947	0.007017	0.934	0.961
29	395	1	0.945	0.007397	0.931	0.960
34	357	1	0.942	0.007836	0.927	0.958

Sex=M

time	n.risk	n.event	survival	std.err	lower 95% CI	upper 95% CI
0	2010	3	0.999	0.000861	0.997	1.000
1	1919	1	0.998	0.001006	0.996	1.000
2	1838	3	0.996	0.001375	0.994	0.999
3	1734	3	0.995	0.001695	0.991	0.998
4	1633	11	0.988	0.002624	0.983	0.993
5	1540	9	0.982	0.003239	0.976	0.989
6	1454	13	0.973	0.004023	0.966	0.981
7	1356	8	0.968	0.004482	0.959	0.976
8	1270	13	0.958	0.005211	0.948	0.968
9	1169	12	0.948	0.005879	0.936	0.959
10	1058	10	0.939	0.006471	0.926	0.952
11	964	7	0.932	0.006918	0.919	0.946
12	883	6	0.926	0.007338	0.912	0.940
13	809	9	0.915	0.008019	0.900	0.931
14	730	7	0.907	0.008602	0.890	0.924
15	669	2	0.904	0.008787	0.887	0.921
18	587	1	0.902	0.008906	0.885	0.920
20	539	1	0.901	0.009045	0.883	0.919

```
   21    525       3    0.896 0.009469        0.877        0.914
   23    473       1    0.894 0.009637        0.875        0.913
   24    452       1    0.892 0.009816        0.873        0.911
   25    438       1    0.890 0.010003        0.870        0.910
   27    398       1    0.887 0.010224        0.868        0.908
   28    384       2    0.883 0.010681        0.862        0.904
   34    330       1    0.880 0.010978        0.859        0.902
   49    199       1    0.876 0.011780        0.853        0.899
   55    148       1    0.870 0.013103        0.845        0.896

Call: survfit(formula = Surv(Date.dpi, as.numeric(Encephalitis)) ~
    Status, data = aData)

                Status=I
 time n.risk n.event survival  std.err lower 95% CI upper 95% CI
    0   2809       5    0.998 0.000795        0.997        1.000
    1   2667       6    0.996 0.001212        0.994        0.998
    2   2560      10    0.992 0.001722        0.989        0.995
    3   2420       7    0.989 0.002030        0.985        0.993
    4   2287      14    0.983 0.002583        0.978        0.988
    5   2165      12    0.978 0.003010        0.972        0.984
    6   2054      14    0.971 0.003477        0.964        0.978
    7   1926       8    0.967 0.003743        0.960        0.974
    8   1811      13    0.960 0.004182        0.952        0.968
    9   1669      14    0.952 0.004668        0.943        0.961
   10   1511      11    0.945 0.005081        0.935        0.955
   11   1380       6    0.941 0.005328        0.931        0.951
   12   1267       7    0.936 0.005650        0.925        0.947
   13   1165       8    0.929 0.006050        0.918        0.941
   14   1057       7    0.923 0.006442        0.911        0.936
   15    973       4    0.919 0.006689        0.906        0.933
   16    913       2    0.917 0.006824        0.904        0.931
   17    879       1    0.916 0.006896        0.903        0.930
   18    849       2    0.914 0.007047        0.900        0.928
   19    802       1    0.913 0.007129        0.899        0.927
   20    775       1    0.912 0.007217        0.898        0.926
   21    756       2    0.909 0.007397        0.895        0.924
   23    681       1    0.908 0.007505        0.894        0.923
   24    654       1    0.907 0.007621        0.892        0.922
   25    635       1    0.905 0.007742        0.890        0.921
   27    575       2    0.902 0.008029        0.887        0.918
   28    551       2    0.899 0.008327        0.883        0.915
   29    525       1    0.897 0.008485        0.881        0.914
   34    483       2    0.893 0.008847        0.876        0.911
   49    288       1    0.890 0.009345        0.872        0.909
   55    212       1    0.886 0.010201        0.866        0.906

                Status=U
 time n.risk n.event survival  std.err lower 95% CI upper 95% CI
    2   1271       1    0.999 0.000786        0.998        1.000
    3   1197       3    0.997 0.001643        0.993        1.000
    4   1124       2    0.995 0.002064        0.991        0.999
    5   1053       3    0.992 0.002628        0.987        0.997
    6    983       4    0.988 0.003303        0.982        0.995
    7    910       2    0.986 0.003635        0.979        0.993
    8    842       2    0.984 0.003986        0.976        0.991
    9    772       2    0.981 0.004364        0.972        0.990
   10    701       2    0.978 0.004779        0.969        0.988
   11    639       2    0.975 0.005232        0.965        0.985
   12    581       1    0.973 0.005485        0.963        0.984
```

```
   13    523       5    0.964 0.006832        0.951        0.978
   14    472       2    0.960 0.007388        0.946        0.975
   21    349       1    0.957 0.007863        0.942        0.973
   27    264       1    0.954 0.008629        0.937        0.971

Call: survfit(formula = Surv(Date.dpi, as.numeric(Encephalitis)) ~ 
    Strain, data = aData)

                Strain=CC002 
 time n.risk n.event survival std.err lower 95% CI upper 95% CI
    1    274       2    0.993 0.00514        0.983        1.000
    2    258       1    0.989 0.00640        0.976        1.000
    3    242       2    0.981 0.00857        0.964        0.998
    4    226       1    0.976 0.00957        0.958        0.995
    6    194       1    0.971 0.01076        0.950        0.993
    7    178       1    0.966 0.01200        0.943        0.990
    9    148       2    0.953 0.01498        0.924        0.983
   29     78       1    0.941 0.01913        0.904        0.979
   34     66       1    0.926 0.02356        0.881        0.974

                Strain=CC005 
 time n.risk n.event survival std.err lower 95% CI upper 95% CI
    0    212       1    0.995 0.00471        0.986        1.000
    1    206       2    0.986 0.00824        0.970        1.000
    2    198       3    0.971 0.01179        0.948        0.994
    3    186       2    0.960 0.01379        0.934        0.988
    4    174       3    0.944 0.01653        0.912        0.977
    5    162       4    0.920 0.01981        0.882        0.960
    6    150       4    0.896 0.02277        0.852        0.942
    7    138       3    0.876 0.02489        0.829        0.927
    8    126       2    0.862 0.02637        0.812        0.916
    9    114       2    0.847 0.02799        0.794        0.904
   10    102       2    0.831 0.02981        0.774        0.891
   11     90       2    0.812 0.03188        0.752        0.877
   12     78       2    0.791 0.03429        0.727        0.862
   13     66       2    0.767 0.03721        0.698        0.844
   14     56       2    0.740 0.04062        0.665        0.824
   21     46       1    0.724 0.04280        0.645        0.813
   28     40       1    0.706 0.04540        0.622        0.801

                Strain=CC006 
 time n.risk n.event survival std.err lower 95% CI upper 95% CI
    0    262       1    0.996 0.00381        0.989            1
   13     67       1    0.981 0.01523        0.952            1

                Strain=CC011 
 time n.risk n.event survival std.err lower 95% CI upper 95% CI
    2    230       1    0.996 0.00434        0.987        1.000
    3    216       1    0.991 0.00631        0.979        1.000
    4    202       1    0.986 0.00796        0.971        1.000
    5    187       1    0.981 0.00950        0.962        1.000
    6    173       2    0.970 0.01232        0.946        0.994
    7    159       3    0.951 0.01599        0.920        0.983
    8    145       4    0.925 0.02023        0.886        0.965
    9    131       4    0.897 0.02404        0.851        0.945
   10    117       2    0.881 0.02596        0.832        0.934
   11    103       1    0.873 0.02708        0.821        0.928

                Strain=CC012 
 time n.risk n.event survival std.err lower 95% CI upper 95% CI
```

time	n.risk	n.event	survival	std.err	lower 95% CI	upper 95% CI
4	372	4	0.989	0.00535	0.979	1.000
5	348	1	0.986	0.00604	0.975	0.998
6	324	2	0.980	0.00738	0.966	0.995
10	228	1	0.976	0.00851	0.959	0.993

Strain=CC012XCC032

time n.risk n.event survival std.err lower 95% CI upper 95% CI

Strain=CC013XCC041

time n.risk n.event survival std.err lower 95% CI upper 95% CI

Strain=CC015

time n.risk n.event survival std.err lower 95% CI upper 95% CI

Strain=CC017

time	n.risk	n.event	survival	std.err	lower 95% CI	upper 95% CI
1	109	1	0.991	0.00913	0.973	1.000
2	105	3	0.963	0.01839	0.927	0.999
3	97	2	0.943	0.02274	0.899	0.988
4	90	4	0.901	0.02986	0.844	0.961
5	84	3	0.869	0.03408	0.804	0.938
6	78	2	0.846	0.03667	0.777	0.921
7	72	3	0.811	0.04040	0.736	0.894
8	66	4	0.762	0.04481	0.679	0.855
9	60	3	0.724	0.04766	0.636	0.824
10	54	2	0.697	0.04952	0.606	0.801
11	48	3	0.653	0.05243	0.558	0.765
12	42	2	0.622	0.05435	0.524	0.739
13	36	4	0.553	0.05828	0.450	0.680
14	30	4	0.479	0.06107	0.373	0.615
21	24	1	0.459	0.06171	0.353	0.598
28	21	1	0.438	0.06253	0.331	0.579

Strain=CC023

time	n.risk	n.event	survival	std.err	lower 95% CI	upper 95% CI
0	209	1	0.995	0.00477	0.986	1.000
1	201	1	0.990	0.00685	0.977	1.000
2	189	1	0.985	0.00859	0.968	1.000
3	177	2	0.974	0.01155	0.952	0.997
4	167	2	0.962	0.01405	0.935	0.990
5	155	1	0.956	0.01527	0.927	0.986
8	119	2	0.940	0.01877	0.904	0.977
9	107	2	0.922	0.02215	0.880	0.967
10	95	3	0.893	0.02709	0.842	0.948
11	83	1	0.882	0.02883	0.828	0.941
12	71	2	0.858	0.03294	0.795	0.925
13	59	2	0.829	0.03770	0.758	0.906
14	48	1	0.811	0.04067	0.735	0.895
18	38	1	0.790	0.04486	0.707	0.883
23	37	1	0.769	0.04846	0.679	0.870

Strain=CC025

time n.risk n.event survival std.err lower 95% CI upper 95% CI

Strain=CC027

time n.risk n.event survival std.err lower 95% CI upper 95% CI

Strain=CC032XCC013

time	n.risk	n.event	survival	std.err	lower 95% CI	upper 95% CI
2	229	1	0.996	0.00436	0.987	1.000

```
    3    209       1    0.991 0.00643        0.978        1.000
    5    179       3    0.974 0.01142        0.952        0.997
    6    169       6    0.940 0.01771        0.906        0.975
   13    109       2    0.922 0.02117        0.882        0.965
   27     70       3    0.883 0.03015        0.826        0.944
   34     60       1    0.868 0.03305        0.806        0.935
   49     40       1    0.846 0.03870        0.774        0.926
   55     30       1    0.818 0.04657        0.732        0.915

                Strain=CC037 
     time n.risk n.event survival std.err lower 95% CI upper 95% CI

                Strain=CC041XCC012 
 time n.risk n.event survival std.err lower 95% CI upper 95% CI
    0    325       2    0.994 0.00434        0.985        1.000
    4    237       1    0.990 0.00601        0.978        1.000
    5    225       1    0.985 0.00742        0.971        1.000
    6    214       1    0.981 0.00870        0.964        0.998
    8    193       1    0.976 0.01003        0.956        0.995

                Strain=CC051 
 time n.risk n.event survival std.err lower 95% CI upper 95% CI
    2    128       1    0.992 0.00778        0.977            1
    5     98       1    0.982 0.01268        0.958            1

                Strain=CC057 
 time n.risk n.event survival std.err lower 95% CI upper 95% CI
   10    318       1    0.997 0.00314        0.991        1.000
   18    184       1    0.991 0.00624        0.979        1.000
   19    168       1    0.986 0.00855        0.969        1.000
   20    152       1    0.979 0.01067        0.958        1.000
   21    136       1    0.972 0.01279        0.947        0.997
   24     88       1    0.961 0.01675        0.929        0.994
   25     72       1    0.947 0.02118        0.907        0.990

                Strain=CC078 
 time n.risk n.event survival std.err lower 95% CI upper 95% CI
    8    245       2    0.992 0.00575        0.981        1.000
    9    231       3    0.979 0.00932        0.961        0.997
   10    217       2    0.970 0.01120        0.948        0.992
   11    203       1    0.965 0.01212        0.942        0.989
   12    190       2    0.955 0.01396        0.928        0.983
   13    178       2    0.944 0.01573        0.914        0.976
   14    166       2    0.933 0.01748        0.899        0.968
   15    154       4    0.909 0.02081        0.869        0.950
   16    142       2    0.896 0.02239        0.853        0.941
   17    130       1    0.889 0.02326        0.845        0.936

Call:
coxph(formula = Surv(Date.dpi, as.numeric(Encephalitis)) ~ Sex, 
    data = aData)

  n= 4200, number of events= 200 

       coef exp(coef) se(coef)     z Pr(>|z|)    
SexM 0.7291    2.0732   0.1488 4.902 9.51e-07 ***
---
Signif. codes:  0 ‚Äò***‚Äô 0.001 ‚Äò**‚Äô 0.01 ‚Äò*‚Äô 0.05 ‚Äò.‚Äô 0.1 ‚Äò ‚Äô 1

     exp(coef) exp(-coef) lower .95 upper .95
```

```
SexM     2.073     0.4823     1.549     2.775

Concordance= 0.574  (se = 0.019 )
Likelihood ratio test= 25.38  on 1 df,   p=5e-07
Wald test            = 24.03  on 1 df,   p=1e-06
Score (logrank) test = 25.11  on 1 df,   p=5e-07

Call:
coxph(formula = Surv(Date.dpi, as.numeric(Encephalitis)) ~ Status,
    data = aData)

  n= 4200, number of events= 200

           coef exp(coef) se(coef)      z Pr(>|z|)
StatusU -0.8690    0.4194   0.1905 -4.561 5.09e-06 ***
---
Signif. codes:  0 ‚Äò***‚Äô 0.001 ‚Äò**‚Äô 0.01 ‚Äò*‚Äô 0.05 ‚Äò.‚Äô 0.1 ‚Äò ‚Äô 1

        exp(coef) exp(-coef) lower .95 upper .95
StatusU    0.4194      2.385    0.2887    0.6092

Concordance= 0.581  (se = 0.014 )
Likelihood ratio test= 24.94  on 1 df,   p=6e-07
Wald test            = 20.8  on 1 df,   p=5e-06
Score (logrank) test = 22.15  on 1 df,   p=3e-06

Call:
coxph(formula = Surv(Date.dpi, as.numeric(Encephalitis)) ~ Strain,
    data = Z)

  n= 4196, number of events= 4196
   (200 observations deleted due to missingness)

                     coef exp(coef) se(coef)     z Pr(>|z|)
StrainCC078       0.11814   1.12540  0.09674 1.221 0.222014
StrainCC057       0.16231   1.17623  0.08820 1.840 0.065741 .
StrainCC012       0.48815   1.62929  0.08558 5.704 1.17e-08 ***
StrainCC041XCC012 0.15238   1.16460  0.09122 1.670 0.094823 .
StrainCC002       0.31492   1.37015  0.09389 3.354 0.000796 ***
StrainCC025       0.34675   1.41446  0.10439 3.322 0.000894 ***
StrainCC012XCC032 0.15503   1.16769  0.10052 1.542 0.123000
StrainCC013XCC041 0.13834   1.14837  0.10538 1.313 0.189266
StrainCC032XCC013 0.29800   1.34716  0.09641 3.091 0.001996 **
StrainCC015       0.53130   1.70114  0.18364 2.893 0.003814 **
StrainCC023       0.47021   1.60033  0.10280 4.574 4.78e-06 ***
StrainCC027       0.48192   1.61918  0.09615 5.012 5.38e-07 ***
StrainCC005       0.30041   1.35041  0.10457 2.873 0.004070 **
StrainCC011       0.37016   1.44796  0.09790 3.781 0.000156 ***
StrainCC051       0.83798   2.31169  0.11026 7.600 2.96e-14 ***
StrainCC037       0.64810   1.91190  0.10062 6.441 1.18e-10 ***
StrainCC006       0.60498   1.83121  0.09503 6.366 1.94e-10 ***
StrainCC017       0.19191   1.21156  0.13900 1.381 0.167389
---
Signif. codes:  0 ‚Äò***‚Äô 0.001 ‚Äò**‚Äô 0.01 ‚Äò*‚Äô 0.05 ‚Äò.‚Äô 0.1 ‚Äò ‚Äô 1

                  exp(coef) exp(-coef) lower .95 upper .95
StrainCC078           1.125     0.8886    0.9310     1.360
StrainCC057           1.176     0.8502    0.9895     1.398
StrainCC012           1.629     0.6138    1.3777     1.927
StrainCC041XCC012     1.165     0.8587    0.9739     1.393
```

StrainCC002	1.370	0.7298	1.1399	1.647
StrainCC025	1.414	0.7070	1.1528	1.736
StrainCC012XCC032	1.168	0.8564	0.9589	1.422
StrainCC013XCC041	1.148	0.8708	0.9341	1.412
StrainCC032XCC013	1.347	0.7423	1.1152	1.627
StrainCC015	1.701	0.5878	1.1869	2.438
StrainCC023	1.600	0.6249	1.3083	1.958
StrainCC027	1.619	0.6176	1.3411	1.955
StrainCC005	1.350	0.7405	1.1002	1.658
StrainCC011	1.448	0.6906	1.1952	1.754
StrainCC051	2.312	0.4326	1.8624	2.869
StrainCC037	1.912	0.5230	1.5697	2.329
StrainCC006	1.831	0.5461	1.5200	2.206
StrainCC017	1.212	0.8254	0.9226	1.591

```
Concordance= 0.58  (se = 0.005 )
Likelihood ratio test= 158.7  on 18 df,   p=<2e-16
Wald test            = 163.6  on 18 df,   p=<2e-16
Score (logrank) test = 166.5  on 18 df,   p=<2e-16
```

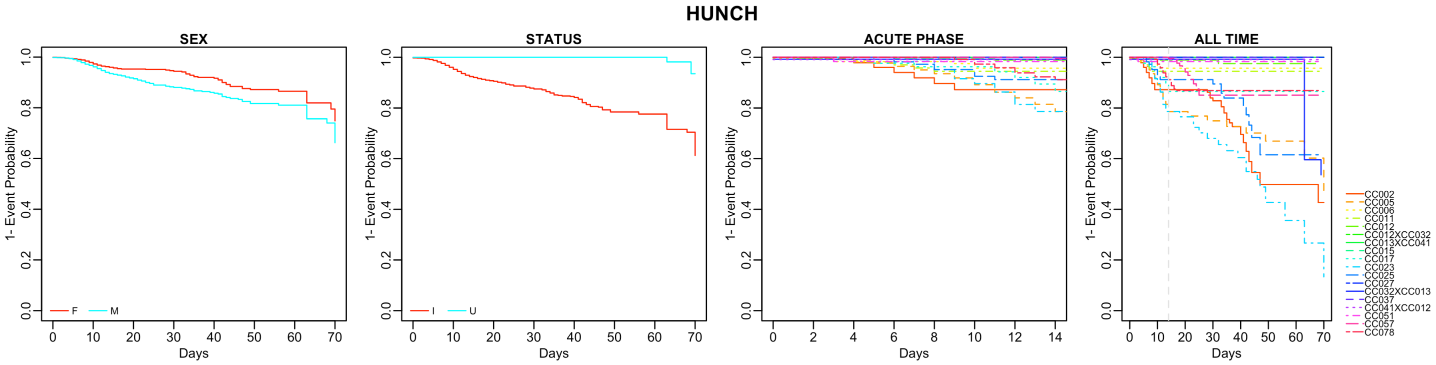

Call: survfit(formula = Surv(Date.dpi, as.numeric(Hunch)) ~ Sex, data = aData)

7 observations deleted due to missingness

Sex=F

time	n.risk	n.event	survival	std.err	lower 95% CI	upper 95% CI
0	2183	2	0.999	0.000648	0.998	1.000
1	2077	1	0.999	0.000806	0.997	1.000
2	1986	1	0.998	0.000950	0.996	1.000
3	1876	5	0.995	0.001520	0.992	0.998
4	1773	2	0.994	0.001713	0.991	0.998
5	1673	2	0.993	0.001906	0.989	0.997
6	1578	4	0.991	0.002279	0.986	0.995
7	1475	3	0.989	0.002554	0.984	0.994
8	1378	6	0.984	0.003089	0.978	0.990
9	1268	7	0.979	0.003692	0.972	0.986
10	1150	7	0.973	0.004302	0.965	0.981
11	1051	5	0.968	0.004753	0.959	0.978
12	961	4	0.964	0.005143	0.954	0.974
13	875	2	0.962	0.005362	0.952	0.973
14	796	2	0.960	0.005615	0.949	0.971
15	733	2	0.957	0.005897	0.946	0.969
16	693	2	0.954	0.006195	0.942	0.966
17	664	1	0.953	0.006350	0.940	0.965
23	520	1	0.951	0.006597	0.938	0.964
28	419	1	0.949	0.006960	0.935	0.962
29	392	1	0.946	0.007351	0.932	0.961
30	384	1	0.944	0.007734	0.929	0.959
32	373	1	0.941	0.008117	0.926	0.957
33	363	3	0.934	0.009209	0.916	0.952
34	354	2	0.928	0.009883	0.909	0.948
35	332	2	0.923	0.010585	0.902	0.944
36	307	1	0.920	0.010969	0.898	0.941
40	290	1	0.916	0.011380	0.894	0.939
41	282	2	0.910	0.012192	0.886	0.934
42	263	3	0.900	0.013446	0.874	0.926
43	234	2	0.892	0.014388	0.864	0.921
44	229	2	0.884	0.015280	0.855	0.915
47	214	2	0.876	0.016216	0.845	0.908
49	205	1	0.872	0.016690	0.839	0.905
56	137	1	0.865	0.017740	0.831	0.901
63	94	5	0.819	0.026138	0.770	0.872
69	34	1	0.795	0.034742	0.730	0.866
70	17	1	0.748	0.055928	0.646	0.866

Sex=M

time	n.risk	n.event	survival	std.err	lower 95% CI	upper 95% CI
0	2010	4	0.998	0.000994	0.996	1.000
1	1919	1	0.997	0.001121	0.995	1.000

```
    3   1734       2    0.996 0.001384        0.994        0.999
    4   1633       4    0.994 0.001842        0.990        0.998
    5   1540       6    0.990 0.002420        0.985        0.995
    6   1454       8    0.985 0.003079        0.979        0.991
    7   1356       9    0.978 0.003751        0.971        0.985
    8   1270       8    0.972 0.004313        0.963        0.980
    9   1169       6    0.967 0.004748        0.958        0.976
   10   1058       6    0.961 0.005222        0.951        0.972
   11    964       7    0.954 0.005813        0.943        0.966
   12    883       7    0.947 0.006432        0.934        0.960
   13    809       6    0.940 0.006994        0.926        0.954
   14    730       4    0.935 0.007414        0.920        0.949
   15    669       2    0.932 0.007651        0.917        0.947
   16    631       2    0.929 0.007907        0.914        0.945
   17    607       2    0.926 0.008171        0.910        0.942
   18    587       3    0.921 0.008574        0.904        0.938
   19    558       2    0.918 0.008856        0.901        0.935
   20    539       2    0.914 0.009144        0.897        0.933
   21    525       3    0.909 0.009577        0.891        0.928
   22    487       2    0.905 0.009895        0.886        0.925
   23    473       3    0.900 0.010373        0.880        0.920
   24    452       2    0.896 0.010702        0.875        0.917
   25    438       3    0.890 0.011200        0.868        0.912
   28    384       2    0.885 0.011611        0.863        0.908
   29    361       1    0.883 0.011834        0.860        0.906
   30    356       1    0.880 0.012058        0.857        0.904
   32    349       1    0.878 0.012284        0.854        0.902
   33    338       1    0.875 0.012519        0.851        0.900
   34    330       1    0.872 0.012759        0.848        0.898
   35    313       2    0.867 0.013272        0.841        0.893
   37    289       1    0.864 0.013561        0.838        0.891
   39    286       1    0.861 0.013846        0.834        0.888
   40    275       1    0.858 0.014145        0.830        0.886
   41    267       1    0.854 0.014452        0.826        0.883
   42    252       3    0.844 0.015427        0.814        0.875
   43    225       1    0.840 0.015808        0.810        0.872
   44    222       1    0.837 0.016184        0.806        0.869
   46    219       1    0.833 0.016554        0.801        0.866
   47    208       2    0.825 0.017337        0.792        0.860
   49    199       2    0.817 0.018126        0.782        0.853
   56    134       1    0.810 0.018988        0.774        0.849
   63     90       6    0.756 0.027716        0.704        0.813
   68     46       1    0.740 0.031617        0.681        0.805
   70     19       2    0.662 0.059283        0.556        0.789

Call: survfit(formula = Surv(Date.dpi, as.numeric(Hunch)) ~ Status,
    data = aData)

7 observations deleted due to missingness
                Status=I
 time n.risk n.event survival  std.err lower 95% CI upper 95% CI
    0   2804       6    0.998 0.000873        0.996        1.000
    1   2662       2    0.997 0.001020        0.995        0.999
    2   2555       1    0.997 0.001092        0.995        0.999
    3   2415       7    0.994 0.001541        0.991        0.997
    4   2282       6    0.991 0.001870        0.988        0.995
    5   2160       8    0.988 0.002269        0.983        0.992
    6   2049      12    0.982 0.002804        0.976        0.987
    7   1921      12    0.976 0.003298        0.969        0.982
    8   1806      14    0.968 0.003842        0.961        0.976
```

```
     9   1665      13    0.961 0.004347        0.952        0.969
    10   1507      13    0.952 0.004879        0.943        0.962
    11   1376      12    0.944 0.005393        0.933        0.955
    12   1263      11    0.936 0.005889        0.924        0.947
    13   1161       8    0.929 0.006274        0.917        0.942
    14   1054       6    0.924 0.006599        0.911        0.937
    15    970       4    0.920 0.006841        0.907        0.934
    16    910       4    0.916 0.007104        0.902        0.930
    17    876       3    0.913 0.007307        0.899        0.927
    18    846       3    0.910 0.007516        0.895        0.925
    19    799       2    0.907 0.007668        0.893        0.923
    20    772       2    0.905 0.007826        0.890        0.921
    21    753       3    0.901 0.008067        0.886        0.917
    22    697       2    0.899 0.008249        0.883        0.915
    23    678       4    0.894 0.008616        0.877        0.911
    24    651       2    0.891 0.008805        0.874        0.908
    25    632       3    0.887 0.009096        0.869        0.905
    28    548       3    0.882 0.009468        0.863        0.901
    29    522       2    0.878 0.009728        0.860        0.898
    30    516       2    0.875 0.009984        0.856        0.895
    32    509       2    0.872 0.010236        0.852        0.892
    33    490       4    0.864 0.010753        0.844        0.886
    34    480       3    0.859 0.011129        0.838        0.881
    35    452       4    0.851 0.011662        0.829        0.875
    36    426       1    0.849 0.011805        0.827        0.873
    37    422       1    0.847 0.011947        0.824        0.871
    39    419       1    0.845 0.012088        0.822        0.869
    40    400       2    0.841 0.012392        0.817        0.866
    41    391       3    0.835 0.012845        0.810        0.860
    42    364       6    0.821 0.013807        0.794        0.848
    43    328       3    0.813 0.014345        0.786        0.842
    44    323       3    0.806 0.014860        0.777        0.836
    46    318       1    0.803 0.015028        0.774        0.833
    47    299       4    0.793 0.015759        0.762        0.824
    49    288       3    0.784 0.016299        0.753        0.817
    56    189       2    0.776 0.017151        0.743        0.810
    63    129      10    0.716 0.024170        0.670        0.765
    68     62       1    0.704 0.026395        0.654        0.758
    70     23       3    0.612 0.054528        0.514        0.729

                Status=U
 time n.risk n.event survival std.err lower 95% CI upper 95% CI
   63     55       1    0.982  0.0180        0.947            1
   69     21       1    0.935  0.0487        0.844            1

Call: survfit(formula = Surv(Date.dpi, as.numeric(Hunch)) ~ Strain,
    data = aData)

7 observations deleted due to missingness
                Strain=CC002
 time n.risk n.event survival std.err lower 95% CI upper 95% CI
    3    240       2    0.992 0.00587        0.980        1.000
    4    226       3    0.979 0.00951        0.960        0.997
    5    210       4    0.960 0.01313        0.934        0.986
    6    194       4    0.940 0.01616        0.909        0.972
    7    178       4    0.919 0.01894        0.883        0.957
    8    162       4    0.896 0.02160        0.855        0.940
    9    148       4    0.872 0.02418        0.826        0.921
   28     85       1    0.862 0.02598        0.812        0.914
   29     78       2    0.840 0.02964        0.784        0.900
```

30	74	1	0.828	0.03134	0.769	0.892
33	70	2	0.805	0.03462	0.740	0.875
34	66	2	0.780	0.03762	0.710	0.858
35	62	2	0.755	0.04040	0.680	0.839
36	54	1	0.741	0.04200	0.663	0.828
37	50	1	0.726	0.04370	0.646	0.817
40	47	2	0.695	0.04699	0.609	0.794
41	43	2	0.663	0.05006	0.572	0.769
42	39	2	0.629	0.05295	0.533	0.742
43	31	2	0.588	0.05678	0.487	0.711
44	27	2	0.545	0.06037	0.439	0.677
47	23	2	0.497	0.06374	0.387	0.640
68	7	1	0.426	0.08552	0.288	0.632

Strain=CC005

time	n.risk	n.event	survival	std.err	lower 95% CI	upper 95% CI
1	206	1	0.995	0.00484	0.986	1.000
4	174	1	0.989	0.00746	0.975	1.000
5	162	2	0.977	0.01131	0.955	1.000
6	150	2	0.964	0.01444	0.936	0.993
7	138	2	0.950	0.01728	0.917	0.985
8	126	2	0.935	0.02003	0.897	0.975
9	114	2	0.919	0.02279	0.875	0.964
10	102	3	0.892	0.02694	0.840	0.946
11	90	3	0.862	0.03103	0.803	0.925
12	78	2	0.840	0.03394	0.776	0.909
13	66	2	0.814	0.03738	0.744	0.891
14	56	2	0.785	0.04132	0.708	0.871
21	46	1	0.768	0.04380	0.687	0.859
28	40	1	0.749	0.04673	0.663	0.846
35	34	1	0.727	0.05028	0.635	0.833
42	28	1	0.701	0.05478	0.602	0.817
49	22	1	0.669	0.06086	0.560	0.800
63	10	1	0.602	0.08385	0.458	0.791
70	4	1	0.452	0.14477	0.241	0.847

Strain=CC006

time	n.risk	n.event	survival	std.err	lower 95% CI	upper 95% CI
0	262	1	0.996	0.00381	0.989	1.000
2	234	1	0.992	0.00570	0.981	1.000
3	218	2	0.983	0.00854	0.966	1.000
9	123	1	0.975	0.01162	0.952	0.998
10	109	2	0.957	0.01695	0.924	0.991

Strain=CC011

time	n.risk	n.event	survival	std.err	lower 95% CI	upper 95% CI
1	240	1	0.996	0.00416	0.988	1.000
3	216	1	0.991	0.00619	0.979	1.000
4	202	2	0.981	0.00923	0.963	1.000
6	173	2	0.970	0.01212	0.947	0.994
7	159	2	0.958	0.01472	0.929	0.987
8	145	2	0.945	0.01723	0.911	0.979

Strain=CC012

time	n.risk	n.event	survival	std.err	lower 95% CI	upper 95% CI
6	324	2	0.994	0.00435	0.985	1
11	204	1	0.989	0.00651	0.976	1
32	71	1	0.975	0.01525	0.946	1

Strain=CC012XCC032

time	n.risk	n.event	survival	std.err	lower 95% CI	upper 95% CI

Strain=CC013XCC041

time	n.risk	n.event	survival	std.err	lower 95% CI	upper 95% CI

Strain=CC015

time	n.risk	n.event	survival	std.err	lower 95% CI	upper 95% CI

Strain=CC017

time	n.risk	n.event	survival	std.err	lower 95% CI	upper 95% CI
5	84	2	0.976	0.0166	0.944	1.000
7	72	1	0.963	0.0212	0.922	1.000
11	48	1	0.943	0.0287	0.888	1.000
12	42	1	0.920	0.0358	0.853	0.993
13	36	1	0.895	0.0429	0.814	0.983
14	30	1	0.865	0.0508	0.771	0.970

Strain=CC023

time	n.risk	n.event	survival	std.err	lower 95% CI	upper 95% CI
7	130	2	0.985	0.0108	0.9637	1.000
8	118	4	0.951	0.0194	0.9139	0.990
9	106	4	0.915	0.0257	0.8664	0.967
10	94	2	0.896	0.0286	0.8415	0.954
11	82	3	0.863	0.0332	0.8004	0.931
12	70	4	0.814	0.0394	0.7400	0.895
13	58	2	0.786	0.0428	0.7062	0.874
18	38	1	0.765	0.0464	0.6793	0.862
23	37	2	0.724	0.0523	0.6281	0.834
25	33	1	0.702	0.0551	0.6016	0.819
28	32	1	0.680	0.0576	0.5758	0.803
32	28	1	0.656	0.0604	0.5472	0.785
35	27	1	0.631	0.0629	0.5193	0.767
39	23	1	0.604	0.0659	0.4876	0.748
42	22	2	0.549	0.0704	0.4269	0.706
46	18	1	0.518	0.0728	0.3937	0.683
47	17	1	0.488	0.0746	0.3616	0.658
49	16	2	0.427	0.0767	0.3002	0.607
56	12	2	0.356	0.0787	0.2306	0.549
63	8	2	0.267	0.0803	0.1479	0.481
70	4	2	0.133	0.0779	0.0425	0.419

Strain=CC025

time	n.risk	n.event	survival	std.err	lower 95% CI	upper 95% CI
6	112	2	0.982	0.0125	0.958	1.000
7	102	1	0.973	0.0157	0.942	1.000
8	92	2	0.951	0.0213	0.911	0.994
10	72	2	0.925	0.0277	0.872	0.981
11	70	1	0.912	0.0303	0.854	0.973
30	54	1	0.895	0.0341	0.830	0.964
33	49	2	0.858	0.0414	0.781	0.943
34	45	1	0.839	0.0446	0.756	0.931
41	27	1	0.808	0.0527	0.711	0.918
42	23	1	0.773	0.0610	0.662	0.902
43	19	1	0.732	0.0701	0.607	0.883
44	15	1	0.684	0.0806	0.542	0.861
47	10	1	0.615	0.0973	0.451	0.839

Strain=CC027

time	n.risk	n.event	survival	std.err	lower 95% CI	upper 95% CI

```
                Strain=CC032XCC013
 time n.risk n.event survival std.err lower 95% CI upper 95% CI
    0    262       2    0.992 0.00538        0.982        1.000
   63     20       8    0.595 0.10876        0.416        0.852
   69     10       1    0.536 0.11301        0.354        0.810

                Strain=CC037
     time n.risk n.event survival std.err lower 95% CI upper 95% CI

                Strain=CC041XCC012
        time        n.risk      n.event     survival      std.err lower 95% CI upper
95% CI
      0.0000     325.0000       3.0000       0.9908       0.0053       0.9804
1.0000

                Strain=CC051
        time        n.risk      n.event     survival      std.err lower 95% CI upper
95% CI
      3.0000     118.0000       2.0000       0.9831       0.0119       0.9600
1.0000

                Strain=CC057
 time n.risk n.event survival std.err lower 95% CI upper 95% CI
   14    248       1    0.996 0.00402        0.988        1.000
   16    216       2    0.987 0.00762        0.972        1.000
   17    200       2    0.977 0.01025        0.957        0.997
   18    184       2    0.966 0.01259        0.942        0.991
   19    168       2    0.955 0.01484        0.926        0.984
   20    152       2    0.942 0.01710        0.909        0.976
   21    136       2    0.928 0.01945        0.891        0.967
   22    120       2    0.913 0.02199        0.871        0.957
   23    104       2    0.895 0.02482        0.848        0.945
   24     88       2    0.875 0.02812        0.822        0.932
   25     72       2    0.851 0.03217        0.790        0.916

                Strain=CC078
 time n.risk n.event survival std.err lower 95% CI upper 95% CI
    9    231       2    0.991  0.0061        0.979        1.000
   10    217       4    0.973  0.0109        0.952        0.995
   11    203       3    0.959  0.0135        0.933        0.986
   12    190       4    0.939  0.0166        0.907        0.972
   13    178       3    0.923  0.0186        0.887        0.960
   14    166       2    0.912  0.0200        0.873        0.952
   15    154       4    0.888  0.0227        0.844        0.934
   16    142       2    0.875  0.0241        0.829        0.924
   17    130       1    0.869  0.0248        0.821        0.919

Call:
coxph(formula = Surv(Date.dpi, as.numeric(Hunch)) ~ Sex, data = aData)

  n= 4193, number of events= 229
   (7 observations deleted due to missingness)

       coef exp(coef) se(coef)     z Pr(>|z|)
SexM 0.4719    1.6031   0.1348 3.501 0.000464 ***
---
Signif. codes:  0 ‚Äò***‚Äô 0.001 ‚Äò**‚Äô 0.01 ‚Äò*‚Äô 0.05 ‚Äò.‚Äô 0.1 ‚Äò ‚Äô 1

     exp(coef) exp(-coef) lower .95 upper .95
SexM     1.603     0.6238     1.231     2.088
```

```
Concordance= 0.561  (se = 0.019 )
Likelihood ratio test= 12.53  on 1 df,   p=4e-04
Wald test            = 12.26  on 1 df,   p=5e-04
Score (logrank) test = 12.48  on 1 df,   p=4e-04

Call:
coxph(formula = Surv(Date.dpi, as.numeric(Hunch)) ~ Status, data = aData)

  n= 4193, number of events= 229
   (7 observations deleted due to missingness)

            coef exp(coef) se(coef)      z Pr(>|z|)
StatusU -3.95334   0.01919  0.71023 -5.566  2.6e-08 ***
---
Signif. codes:  0 ,Äò***,Äô 0.001 ,Äò**,Äô 0.01 ,Äò*,Äô 0.05 ,Äò.,Äô 0.1 ,Äò ,Äô 1

        exp(coef) exp(-coef) lower .95 upper .95
StatusU   0.01919      52.11   0.00477    0.0772

Concordance= 0.66  (se = 0.004 )
Likelihood ratio test= 153.1  on 1 df,   p=<2e-16
Wald test            = 30.98  on 1 df,   p=3e-08
Score (logrank) test = 99.34  on 1 df,   p=<2e-16

Call:
coxph(formula = Surv(Date.dpi, as.numeric(Hunch)) ~ Strain, data = Z)

  n= 4389, number of events= 232
   (7 observations deleted due to missingness)

                       coef  exp(coef)   se(coef)      z Pr(>|z|)
StrainCC078        2.024e+00  7.567e+00  6.117e-01  3.309 0.000938 ***
StrainCC057        1.466e+00  4.330e+00  6.179e-01  2.372 0.017694 *
StrainCC012       -2.827e-02  9.721e-01  7.642e-01 -0.037 0.970489
StrainCC041XCC012 -3.630e-01  6.956e-01  8.175e-01 -0.444 0.657057
StrainCC002        2.807e+00  1.657e+01  5.949e-01  4.719 2.37e-06 ***
StrainCC025        2.271e+00  9.687e+00  6.264e-01  3.625 0.000289 ***
StrainCC012XCC032 -1.697e+01  4.276e-08  3.005e+03 -0.006 0.995495
StrainCC013XCC041 -1.697e+01  4.278e-08  3.249e+03 -0.005 0.995834
StrainCC032XCC013  1.332e+00  3.788e+00  6.523e-01  2.042 0.041175 *
StrainCC015       -1.697e+01  4.253e-08  8.929e+03 -0.002 0.998483
StrainCC023        3.133e+00  2.295e+01  5.992e-01  5.229 1.70e-07 ***
StrainCC027       -1.697e+01  4.270e-08  3.252e+03 -0.005 0.995836
StrainCC005        2.703e+00  1.492e+01  6.057e-01  4.462 8.13e-06 ***
StrainCC011        1.426e+00  4.161e+00  6.592e-01  2.163 0.030566 *
StrainCC051        8.523e-01  2.345e+00  9.138e-01  0.933 0.350937
StrainCC037       -1.698e+01  4.234e-08  3.973e+03 -0.004 0.996591
StrainCC006        1.233e+00  3.432e+00  6.910e-01  1.784 0.074357 .
StrainCC017        1.835e+00  6.267e+00  6.913e-01  2.655 0.007934 **
---
Signif. codes:  0 ,Äò***,Äô 0.001 ,Äò**,Äô 0.01 ,Äò*,Äô 0.05 ,Äò.,Äô 0.1 ,Äò ,Äô 1

                  exp(coef) exp(-coef) lower .95 upper .95
StrainCC078       7.567e+00  1.322e-01    2.2816    25.094
StrainCC057       4.330e+00  2.309e-01    1.2899    14.535
StrainCC012       9.721e-01  1.029e+00    0.2174     4.347
StrainCC041XCC012 6.956e-01  1.438e+00    0.1401     3.453
StrainCC002       1.657e+01  6.036e-02    5.1628    53.168
StrainCC025       9.687e+00  1.032e-01    2.8378    33.066
```

```
StrainCC012XCC032 4.276e-08  2.339e+07    0.0000       Inf
StrainCC013XCC041 4.278e-08  2.337e+07    0.0000       Inf
StrainCC032XCC013 3.788e+00  2.640e-01    1.0548    13.603
StrainCC015       4.253e-08  2.351e+07    0.0000       Inf
StrainCC023       2.295e+01  4.358e-02    7.0916    74.261
StrainCC027       4.270e-08  2.342e+07    0.0000       Inf
StrainCC005       1.492e+01  6.704e-02    4.5511    48.893
StrainCC011       4.161e+00  2.403e-01    1.1430    15.146
StrainCC051       2.345e+00  4.264e-01    0.3912    14.060
StrainCC037       4.234e-08  2.362e+07    0.0000       Inf
StrainCC006       3.432e+00  2.914e-01    0.8858    13.294
StrainCC017       6.267e+00  1.596e-01    1.6167    24.292

Concordance= 0.777  (se = 0.017 )
Likelihood ratio test= 299  on 18 df,   p=<2e-16
Wald test            = 152.1  on 18 df,   p=<2e-16
Score (logrank) test = 333.6  on 18 df,   p=<2e-16
```

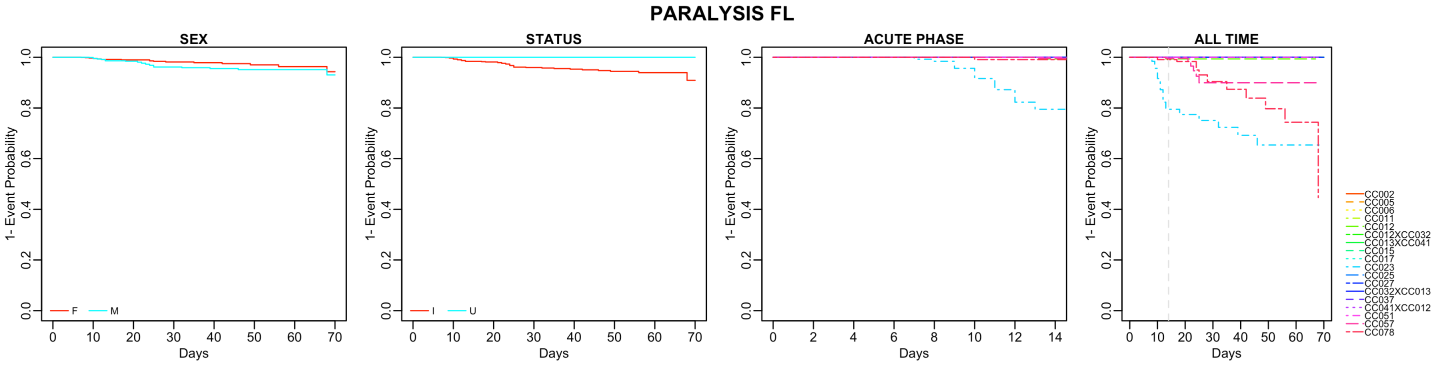


```
Call: survfit(formula = Surv(Date.dpi, as.numeric(Paralysis_FL)) ~
    Sex, data = aData)

                Sex=F
 time n.risk n.event survival  std.err lower 95% CI upper 95% CI
    7   1480       1    0.999 0.000675        0.998        1.000
    8   1383       1    0.999 0.000989        0.997        1.000
    9   1272       2    0.997 0.001485        0.994        1.000
   10   1154       2    0.995 0.001920        0.992        0.999
   11   1055       2    0.993 0.002335        0.989        0.998
   12    965       2    0.991 0.002746        0.986        0.997
   17    667       1    0.990 0.003119        0.984        0.996
   24    498       2    0.986 0.004185        0.978        0.994
   25    481       1    0.984 0.004652        0.975        0.993
   28    422       1    0.982 0.005192        0.971        0.992
   35    335       1    0.979 0.005946        0.967        0.990
   42    263       1    0.975 0.006991        0.961        0.989
   49    205       1    0.970 0.008421        0.954        0.987
   56    137       1    0.963 0.010939        0.942        0.985
   68     48       1    0.943 0.022558        0.900        0.988

                Sex=M
 time n.risk n.event survival  std.err lower 95% CI upper 95% CI
    9   1169       1    0.999 0.000855        0.997        1.000
   10   1058       4    0.995 0.002069        0.991        0.999
   11    964       2    0.993 0.002528        0.988        0.998
   12    883       2    0.991 0.002981        0.985        0.997
   13    809       4    0.986 0.003843        0.979        0.994
   18    587       1    0.984 0.004188        0.976        0.993
   21    525       2    0.981 0.004941        0.971        0.990
   22    487       2    0.977 0.005682        0.966        0.988
   23    473       2    0.973 0.006365        0.960        0.985
   24    452       2    0.968 0.007026        0.955        0.982
   25    438       3    0.962 0.007953        0.946        0.977
   32    349       1    0.959 0.008394        0.943        0.975
   39    286       1    0.956 0.009010        0.938        0.973
   46    219       1    0.951 0.009969        0.932        0.971
   68     46       1    0.930 0.022658        0.887        0.976
```

```
Call: survfit(formula = Surv(Date.dpi, as.numeric(Paralysis_FL)) ~
    Status, data = aData)

                Status=I
 time n.risk n.event survival  std.err lower 95% CI upper 95% CI
    7   1926       1    0.999 0.000519        0.998        1.000
    8   1811       1    0.999 0.000757        0.997        1.000
    9   1669       3    0.997 0.001282        0.995        1.000
```

```
   10   1511       6    0.993 0.002058        0.989        0.997
   11   1380       4    0.990 0.002505        0.985        0.995
   12   1267       4    0.987 0.002945        0.981        0.993
   13   1165       4    0.984 0.003387        0.977        0.990
   17    879       1    0.983 0.003564        0.976        0.990
   18    849       1    0.982 0.003743        0.974        0.989
   21    756       2    0.979 0.004159        0.971        0.987
   22    700       2    0.976 0.004593        0.967        0.985
   23    681       2    0.973 0.005007        0.963        0.983
   24    654       4    0.967 0.005794        0.956        0.979
   25    635       4    0.961 0.006509        0.949        0.974
   28    551       1    0.959 0.006727        0.946        0.973
   32    512       1    0.958 0.006970        0.944        0.971
   35    455       1    0.955 0.007266        0.941        0.970
   39    420       1    0.953 0.007596        0.938        0.968
   42    364       1    0.951 0.008014        0.935        0.966
   46    318       1    0.948 0.008528        0.931        0.964
   49    288       1    0.944 0.009111        0.927        0.962
   56    189       1    0.939 0.010342        0.919        0.960
   68     62       2    0.909 0.023333        0.864        0.956

                Status=U
     time n.risk n.event survival std.err lower 95% CI upper 95% CI

Call: survfit(formula = Surv(Date.dpi, as.numeric(Paralysis_FL)) ~ 
    Strain, data = aData)

                Strain=CC002
     time n.risk n.event survival std.err lower 95% CI upper 95% CI

                Strain=CC005
     time n.risk n.event survival std.err lower 95% CI upper 95% CI

                Strain=CC006
     time n.risk n.event survival std.err lower 95% CI upper 95% CI

                Strain=CC011
     time n.risk n.event survival std.err lower 95% CI upper 95% CI

                Strain=CC012
        time        n.risk      n.event     survival      std.err lower 95% CI upper 
95% CI 
    1.30e+01     1.56e+02     1.00e+00     9.94e-01     6.39e-03     9.81e-01 
1.00e+00 

                Strain=CC012XCC032
     time n.risk n.event survival std.err lower 95% CI upper 95% CI

                Strain=CC013XCC041
     time n.risk n.event survival std.err lower 95% CI upper 95% CI

                Strain=CC015
     time n.risk n.event survival std.err lower 95% CI upper 95% CI

                Strain=CC017
     time n.risk n.event survival std.err lower 95% CI upper 95% CI

                Strain=CC023
 time n.risk n.event survival std.err lower 95% CI upper 95% CI
    7    131       1    0.992  0.0076        0.978        1.000
```

```
    8    119       1    0.984  0.0112        0.962        1.000
    9    107       3    0.956  0.0191        0.920        0.995
   10     95       4    0.916  0.0269        0.865        0.970
   11     83       4    0.872  0.0335        0.809        0.940
   12     71       4    0.823  0.0396        0.749        0.904
   13     59       2    0.795  0.0429        0.715        0.884
   18     38       1    0.774  0.0466        0.688        0.871
   25     33       1    0.751  0.0507        0.658        0.857
   32     28       1    0.724  0.0555        0.623        0.841
   39     23       1    0.692  0.0614        0.582        0.824
   46     18       1    0.654  0.0690        0.532        0.804

                Strain=CC025
     time n.risk n.event survival std.err lower 95% CI upper 95% CI

                Strain=CC027
     time n.risk n.event survival std.err lower 95% CI upper 95% CI

                Strain=CC032XCC013
     time n.risk n.event survival std.err lower 95% CI upper 95% CI

                Strain=CC037
     time n.risk n.event survival std.err lower 95% CI upper 95% CI

                Strain=CC041XCC012
     time n.risk n.event survival std.err lower 95% CI upper 95% CI

                Strain=CC051
     time n.risk n.event survival std.err lower 95% CI upper 95% CI

                Strain=CC057
 time n.risk n.event survival std.err lower 95% CI upper 95% CI
   13    264       1    0.996 0.00378        0.989        1.000
   21    136       2    0.982 0.01094        0.960        1.000
   22    120       2    0.965 0.01572        0.935        0.997
   23    104       2    0.947 0.02017        0.908        0.987
   24     88       2    0.925 0.02479        0.878        0.975
   25     72       2    0.899 0.03003        0.842        0.960

                Strain=CC078
 time n.risk n.event survival std.err lower 95% CI upper 95% CI
   10    217       2    0.991 0.00649        0.978        1.000
   17    130       1    0.983 0.00995        0.964        1.000
   24     59       2    0.950 0.02508        0.902        1.000
   25     49       1    0.930 0.03117        0.871        0.994
   28     35       1    0.904 0.04004        0.829        0.986
   35     30       1    0.874 0.04874        0.783        0.975
   42     25       1    0.839 0.05798        0.733        0.960
   49     20       1    0.797 0.06860        0.673        0.943
   56     15       1    0.744 0.08205        0.599        0.923
   68      5       2    0.446 0.17022        0.211        0.942

Call:
coxph(formula = Surv(Date.dpi, as.numeric(Paralysis_FL)) ~ Sex,
    data = aData)

  n= 4200, number of events= 49

       coef exp(coef) se(coef)     z Pr(>|z|)
SexM 0.4547    1.5756   0.2907 1.564    0.118
```

```
     exp(coef) exp(-coef) lower .95 upper .95
SexM     1.576     0.6347    0.8913     2.785

Concordance= 0.549  (se = 0.04 )
Likelihood ratio test= 2.49  on 1 df,   p=0.1
Wald test            = 2.45  on 1 df,   p=0.1
Score (logrank) test = 2.49  on 1 df,   p=0.1

Call:
coxph(formula = Surv(Date.dpi, as.numeric(Paralysis_FL)) ~ Status,
    data = aData)

  n= 4200, number of events= 49

              coef  exp(coef)   se(coef)      z Pr(>|z|)
StatusU -1.862e+01  8.223e-09  2.336e+03 -0.008    0.994

        exp(coef) exp(-coef) lower .95 upper .95
StatusU 8.223e-09  121611293         0       Inf

Concordance= 0.657  (se = 0.005 )
Likelihood ratio test= 36.72  on 1 df,   p=1e-09
Wald test            = 0  on 1 df,   p=1
Score (logrank) test = 22.28  on 1 df,   p=2e-06

Call:
coxph(formula = Surv(Date.dpi, as.numeric(Paralysis_FL)) ~ Strain,
    data = Z)

  n= 4347, number of events= 4347
   (49 observations deleted due to missingness)

                     coef exp(coef) se(coef)     z Pr(>|z|)
StrainCC078       0.17602   1.19246  0.09605 1.832 0.066877 .
StrainCC057       0.16286   1.17688  0.08836 1.843 0.065292 .
StrainCC012       0.50028   1.64918  0.08539 5.859 4.66e-09 ***
StrainCC041XCC012 0.17398   1.19003  0.09088 1.914 0.055572 .
StrainCC002       0.34007   1.40504  0.09304 3.655 0.000257 ***
StrainCC025       0.34946   1.41830  0.10438 3.348 0.000815 ***
StrainCC012XCC032 0.15922   1.17260  0.10050 1.584 0.113110
StrainCC013XCC041 0.14280   1.15350  0.10536 1.355 0.175293
StrainCC032XCC013 0.31431   1.36932  0.09480 3.315 0.000915 ***
StrainCC015       0.53277   1.70364  0.18364 2.901 0.003717 **
StrainCC023       0.52455   1.68969  0.10296 5.094 3.50e-07 ***
StrainCC027       0.48508   1.62430  0.09613 5.046 4.51e-07 ***
StrainCC005       0.40524   1.49966  0.09949 4.073 4.64e-05 ***
StrainCC011       0.41940   1.52105  0.09605 4.367 1.26e-05 ***
StrainCC051       0.84805   2.33510  0.10981 7.723 1.14e-14 ***
StrainCC037       0.64886   1.91336  0.10060 6.450 1.12e-10 ***
StrainCC006       0.61021   1.84083  0.09486 6.433 1.25e-10 ***
StrainCC017       0.40895   1.50524  0.11853 3.450 0.000560 ***
---
Signif. codes:  0 ‚Äò***‚Äô 0.001 ‚Äò**‚Äô 0.01 ‚Äò*‚Äô 0.05 ‚Äò.‚Äô 0.1 ‚Äò ‚Äô 1

                  exp(coef) exp(-coef) lower .95 upper .95
StrainCC078           1.192     0.8386    0.9878     1.439
StrainCC057           1.177     0.8497    0.9897     1.399
StrainCC012           1.649     0.6064    1.3950     1.950
StrainCC041XCC012     1.190     0.8403    0.9959     1.422
```

StrainCC002	1.405	0.7117	1.1708	1.686
StrainCC025	1.418	0.7051	1.1559	1.740
StrainCC012XCC032	1.173	0.8528	0.9630	1.428
StrainCC013XCC041	1.154	0.8669	0.9383	1.418
StrainCC032XCC013	1.369	0.7303	1.1371	1.649
StrainCC015	1.704	0.5870	1.1887	2.442
StrainCC023	1.690	0.5918	1.3809	2.068
StrainCC027	1.624	0.6156	1.3454	1.961
StrainCC005	1.500	0.6668	1.2340	1.823
StrainCC011	1.521	0.6574	1.2600	1.836
StrainCC051	2.335	0.4282	1.8829	2.896
StrainCC037	1.913	0.5226	1.5710	2.330
StrainCC006	1.841	0.5432	1.5285	2.217
StrainCC017	1.505	0.6643	1.1932	1.899

```
Concordance= 0.58  (se = 0.005 )
Likelihood ratio test= 159.2  on 18 df,   p=<2e-16
Wald test            = 162.1  on 18 df,   p=<2e-16
Score (logrank) test = 164.8  on 18 df,   p=<2e-16
```

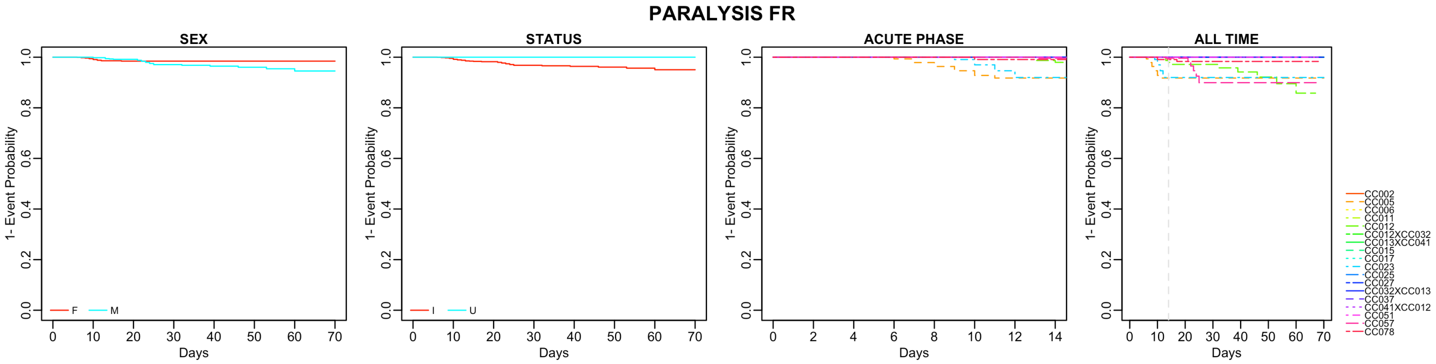


```
Call: survfit(formula = Surv(Date.dpi, as.numeric(Paralysis_FR)) ~
    Sex, data = aData)

                Sex=F
 time n.risk n.event survival  std.err lower 95% CI upper 95% CI
    6   1583       1    0.999 0.000632        0.998        1.000
    7   1480       2    0.998 0.001144        0.996        1.000
    8   1383       2    0.997 0.001531        0.994        1.000
    9   1272       3    0.994 0.002042        0.990        0.998
   10   1154       4    0.991 0.002665        0.986        0.996
   11   1055       3    0.988 0.003114        0.982        0.994
   12    965       2    0.986 0.003428        0.979        0.993
   17    667       1    0.984 0.003728        0.977        0.992

                Sex=M
 time n.risk n.event survival std.err lower 95% CI upper 95% CI
   10   1058       2    0.998 0.00134        0.995        1.000
   13    809       3    0.994 0.00251        0.989        0.999
   14    730       1    0.993 0.00286        0.987        0.999
   15    669       1    0.992 0.00321        0.985        0.998
   21    525       2    0.988 0.00417        0.980        0.996
   22    487       2    0.984 0.00504        0.974        0.994
   23    473       2    0.980 0.00581        0.968        0.991
   24    452       2    0.975 0.00655        0.962        0.988
   25    438       2    0.971 0.00723        0.957        0.985
   32    349       1    0.968 0.00773        0.953        0.983
   39    286       1    0.965 0.00841        0.948        0.981
   46    219       1    0.960 0.00946        0.942        0.979
   53    163       1    0.954 0.01108        0.933        0.976
   60    108       1    0.945 0.01407        0.918        0.973

Call: survfit(formula = Surv(Date.dpi, as.numeric(Paralysis_FR)) ~
    Status, data = aData)

                Status=I
 time n.risk n.event survival  std.err lower 95% CI upper 95% CI
    6   2054       1    1.000 0.000487        0.999        1.000
    7   1926       2    0.998 0.000880        0.997        1.000
    8   1811       2    0.997 0.001175        0.995        1.000
    9   1669       3    0.996 0.001563        0.993        0.999
   10   1511       6    0.992 0.002240        0.987        0.996
   11   1380       3    0.989 0.002558        0.984        0.994
   12   1267       2    0.988 0.002782        0.982        0.993
   13   1165       3    0.985 0.003139        0.979        0.992
   14   1057       1    0.984 0.003271        0.978        0.991
   15    973       1    0.983 0.003421        0.977        0.990
   17    879       1    0.982 0.003595        0.975        0.989
   21    756       2    0.980 0.004028        0.972        0.988
```

```
   22    700       2    0.977 0.004477        0.968        0.986
   23    681       2    0.974 0.004902        0.964        0.984
   24    654       2    0.971 0.005320        0.961        0.982
   25    635       2    0.968 0.005726        0.957        0.979
   32    512       1    0.966 0.006019        0.954        0.978
   39    420       1    0.964 0.006429        0.951        0.976
   46    318       1    0.961 0.007087        0.947        0.975
   53    235       1    0.957 0.008151        0.941        0.973
   60    154       1    0.950 0.010195        0.931        0.971

                Status=U
     time n.risk n.event survival std.err lower 95% CI upper 95% CI

Call: survfit(formula = Surv(Date.dpi, as.numeric(Paralysis_FR)) ~ 
    Strain, data = aData)

                Strain=CC002
     time n.risk n.event survival std.err lower 95% CI upper 95% CI

                Strain=CC005
 time n.risk n.event survival std.err lower 95% CI upper 95% CI
    6    150       1    0.993 0.00664        0.980        1.000
    7    138       2    0.979 0.01204        0.956        1.000
    8    126       2    0.963 0.01610        0.932        0.995
    9    114       2    0.946 0.01976        0.909        0.986
   10    102       2    0.928 0.02333        0.883        0.975
   11     90       1    0.918 0.02525        0.869        0.968

                Strain=CC006
     time n.risk n.event survival std.err lower 95% CI upper 95% CI

                Strain=CC011
     time n.risk n.event survival std.err lower 95% CI upper 95% CI

                Strain=CC012
 time n.risk n.event survival std.err lower 95% CI upper 95% CI
   13    156       2    0.987 0.00901        0.970        1.000
   14    132       1    0.980 0.01164        0.957        1.000
   15    120       1    0.972 0.01412        0.944        1.000
   32     71       1    0.958 0.01945        0.920        0.997
   39     59       1    0.942 0.02499        0.894        0.992
   46     47       1    0.922 0.03148        0.862        0.985
   53     35       1    0.895 0.04011        0.820        0.977
   60     24       1    0.858 0.05302        0.760        0.968

                Strain=CC012XCC032
     time n.risk n.event survival std.err lower 95% CI upper 95% CI

                Strain=CC013XCC041
     time n.risk n.event survival std.err lower 95% CI upper 95% CI

                Strain=CC015
     time n.risk n.event survival std.err lower 95% CI upper 95% CI

                Strain=CC017
     time n.risk n.event survival std.err lower 95% CI upper 95% CI

                Strain=CC023
 time n.risk n.event survival std.err lower 95% CI upper 95% CI
    9    107       1    0.991  0.0093        0.973        1.000
```

```
   10     95       2    0.970  0.0172        0.937        1.000
   11     83       2    0.946  0.0234        0.902        0.993
   12     71       2    0.920  0.0294        0.864        0.979

                Strain=CC025
     time n.risk n.event survival std.err lower 95% CI upper 95% CI

                Strain=CC027
     time n.risk n.event survival std.err lower 95% CI upper 95% CI

                Strain=CC032XCC013
     time n.risk n.event survival std.err lower 95% CI upper 95% CI

                Strain=CC037
     time n.risk n.event survival std.err lower 95% CI upper 95% CI

                Strain=CC041XCC012
     time n.risk n.event survival std.err lower 95% CI upper 95% CI

                Strain=CC051
     time n.risk n.event survival std.err lower 95% CI upper 95% CI

                Strain=CC057
 time n.risk n.event survival std.err lower 95% CI upper 95% CI
   13    264       1    0.996 0.00378        0.989        1.000
   21    136       2    0.982 0.01094        0.960        1.000
   22    120       2    0.965 0.01572        0.935        0.997
   23    104       2    0.947 0.02017        0.908        0.987
   24     88       2    0.925 0.02479        0.878        0.975
   25     72       2    0.899 0.03003        0.842        0.960

                Strain=CC078
 time n.risk n.event survival std.err lower 95% CI upper 95% CI
   10    217       2    0.991 0.00649        0.978            1
   17    130       1    0.983 0.00995        0.964            1

Call:
coxph(formula = Surv(Date.dpi, as.numeric(Paralysis_FR)) ~ Sex,
    data = aData)

  n= 4200, number of events= 40

       coef exp(coef) se(coef)     z Pr(>|z|)
SexM 0.2850    1.3298   0.3178 0.897     0.37

     exp(coef) exp(-coef) lower .95 upper .95
SexM      1.33      0.752    0.7132     2.479

Concordance= 0.448  (se = 0.039 )
Likelihood ratio test= 0.81  on 1 df,   p=0.4
Wald test            = 0.8  on 1 df,   p=0.4
Score (logrank) test = 0.81  on 1 df,   p=0.4

Call:
coxph(formula = Surv(Date.dpi, as.numeric(Paralysis_FR)) ~ Status,
    data = aData)

  n= 4200, number of events= 40

              coef  exp(coef)   se(coef)      z Pr(>|z|)
```

```
StatusU -1.861e+01  8.235e-09  2.587e+03 -0.007    0.994

        exp(coef) exp(-coef) lower .95 upper .95
StatusU 8.235e-09  121433475         0       Inf

Concordance= 0.658  (se = 0.005 )
Likelihood ratio test= 29.92  on 1 df,   p=5e-08
Wald test            = 0  on 1 df,   p=1
Score (logrank) test = 18.14  on 1 df,   p=2e-05

Call:
coxph(formula = Surv(Date.dpi, as.numeric(Paralysis_FR)) ~ Strain,
    data = Z)

  n= 4356, number of events= 4356
   (40 observations deleted due to missingness)

                     coef exp(coef) se(coef)     z Pr(>|z|)
StrainCC078       0.13924   1.14939  0.09519 1.463 0.143561
StrainCC057       0.16416   1.17841  0.08836 1.858 0.063175 .
StrainCC012       0.52150   1.68455  0.08562 6.091 1.12e-09 ***
StrainCC041XCC012 0.17360   1.18958  0.09088 1.910 0.056097 .
StrainCC002       0.33999   1.40493  0.09304 3.654 0.000258 ***
StrainCC025       0.34932   1.41810  0.10438 3.346 0.000819 ***
StrainCC012XCC032 0.15925   1.17263  0.10050 1.585 0.113045
StrainCC013XCC041 0.14282   1.15352  0.10536 1.356 0.175246
StrainCC032XCC013 0.31420   1.36916  0.09480 3.314 0.000919 ***
StrainCC015       0.53336   1.70465  0.18364 2.904 0.003679 **
StrainCC023       0.50818   1.66226  0.10068 5.048 4.47e-07 ***
StrainCC027       0.48548   1.62495  0.09613 5.050 4.41e-07 ***
StrainCC005       0.38400   1.46815  0.10065 3.815 0.000136 ***
StrainCC011       0.41972   1.52153  0.09605 4.370 1.24e-05 ***
StrainCC051       0.84936   2.33815  0.10981 7.735 1.04e-14 ***
StrainCC037       0.64995   1.91544  0.10060 6.461 1.04e-10 ***
StrainCC006       0.61093   1.84215  0.09486 6.441 1.19e-10 ***
StrainCC017       0.40905   1.50539  0.11853 3.451 0.000559 ***
---
Signif. codes:  0 ‚Äò***‚Äô 0.001 ‚Äò**‚Äô 0.01 ‚Äò*‚Äô 0.05 ‚Äò.‚Äô 0.1 ‚Äò ‚Äô 1

                  exp(coef) exp(-coef) lower .95 upper .95
StrainCC078           1.149     0.8700    0.9538     1.385
StrainCC057           1.178     0.8486    0.9910     1.401
StrainCC012           1.685     0.5936    1.4243     1.992
StrainCC041XCC012     1.190     0.8406    0.9955     1.422
StrainCC002           1.405     0.7118    1.1707     1.686
StrainCC025           1.418     0.7052    1.1557     1.740
StrainCC012XCC032     1.173     0.8528    0.9630     1.428
StrainCC013XCC041     1.154     0.8669    0.9383     1.418
StrainCC032XCC013     1.369     0.7304    1.1370     1.649
StrainCC015           1.705     0.5866    1.1894     2.443
StrainCC023           1.662     0.6016    1.3646     2.025
StrainCC027           1.625     0.6154    1.3459     1.962
StrainCC005           1.468     0.6811    1.2053     1.788
StrainCC011           1.522     0.6572    1.2604     1.837
StrainCC051           2.338     0.4277    1.8854     2.900
StrainCC037           1.915     0.5221    1.5727     2.333
StrainCC006           1.842     0.5428    1.5296     2.219
StrainCC017           1.505     0.6643    1.1933     1.899

Concordance= 0.583  (se = 0.005 )
```

```
Likelihood ratio test= 165.2  on 18 df,   p=<2e-16
Wald test            = 168.2  on 18 df,   p=<2e-16
Score (logrank) test = 171.2  on 18 df,   p=<2e-16
```

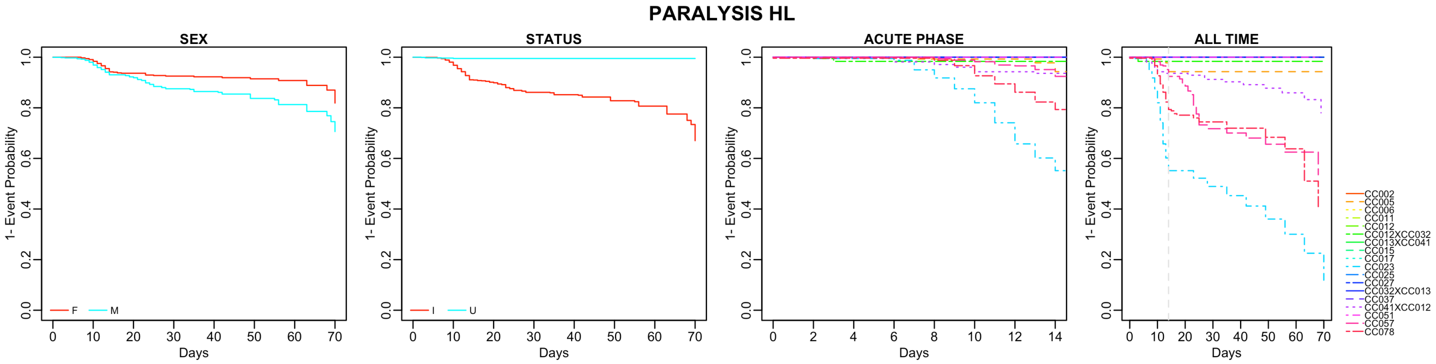


```
Call: survfit(formula = Surv(Date.dpi, as.numeric(Paralysis_HL)) ~
    Sex, data = aData)

                Sex=F
 time n.risk n.event survival std.err lower 95% CI upper 95% CI
    7   1480       4    0.997 0.00135        0.995        1.000
    8   1383       4    0.994 0.00197        0.991        0.998
    9   1272       5    0.991 0.00263        0.985        0.996
   10   1154       8    0.984 0.00356        0.977        0.991
   11   1055       9    0.975 0.00449        0.966        0.984
   12    965       9    0.966 0.00538        0.956        0.977
   13    879      10    0.955 0.00634        0.943        0.968
   14    799      11    0.942 0.00739        0.928        0.957
   15    736       1    0.941 0.00749        0.926        0.956
   16    696       2    0.938 0.00771        0.923        0.953
   17    667       1    0.937 0.00782        0.921        0.952
   23    523       4    0.929 0.00855        0.913        0.946
   25    481       1    0.928 0.00874        0.911        0.945
   28    422       1    0.925 0.00899        0.908        0.943
   35    335       1    0.923 0.00938        0.904        0.941
   42    263       1    0.919 0.00998        0.900        0.939
   49    205       1    0.915 0.01089        0.893        0.936
   56    137       1    0.908 0.01270        0.883        0.933
   63     94       2    0.889 0.01836        0.853        0.925
   68     48       1    0.870 0.02566        0.821        0.922
   70     17       1    0.819 0.05522        0.718        0.935

                Sex=M
 time n.risk n.event survival  std.err lower 95% CI upper 95% CI
    0   2010       1    1.000 0.000497        0.999        1.000
    2   1838       2    0.998 0.000915        0.997        1.000
    3   1734       2    0.997 0.001224        0.995        1.000
    6   1454       5    0.994 0.001957        0.990        0.998
    7   1356       2    0.992 0.002212        0.988        0.997
    8   1270       6    0.988 0.002914        0.982        0.993
    9   1169       9    0.980 0.003839        0.973        0.988
   10   1058      12    0.969 0.004958        0.959        0.979
   11    964      10    0.959 0.005838        0.948        0.970
   12    883       7    0.951 0.006460        0.939        0.964
   13    809       9    0.941 0.007288        0.927        0.955
   14    730       8    0.930 0.008068        0.915        0.946
   18    587       2    0.927 0.008346        0.911        0.944
   19    558       3    0.922 0.008784        0.905        0.940
   20    539       2    0.919 0.009078        0.901        0.937
   21    525       4    0.912 0.009660        0.893        0.931
   22    487       2    0.908 0.009977        0.889        0.928
   23    473       5    0.898 0.010755        0.878        0.920
   24    452       3    0.893 0.011222        0.871        0.915
```

```
   25    438        4    0.884 0.011836        0.861        0.908
   27    398        2    0.880 0.012187        0.856        0.904
   28    384        2    0.875 0.012547        0.851        0.900
   34    330        1    0.873 0.012786        0.848        0.898
   35    313        3    0.864 0.013545        0.838        0.891
   41    267        1    0.861 0.013875        0.834        0.889
   42    252        2    0.854 0.014583        0.826        0.883
   49    199        4    0.837 0.016626        0.805        0.870
   55    148        1    0.831 0.017449        0.798        0.866
   56    134        3    0.813 0.020097        0.774        0.853
   63     90        3    0.786 0.024778        0.739        0.836
   68     46        1    0.769 0.029546        0.713        0.829
   69     33        1    0.745 0.036701        0.677        0.821
   70     19        1    0.706 0.051641        0.612        0.815

Call: survfit(formula = Surv(Date.dpi, as.numeric(Paralysis_HL)) ~ 
    Status, data = aData)

                Status=I 
 time n.risk n.event survival  std.err lower 95% CI upper 95% CI
    0   2809       1    1.000 0.000356        0.999        1.000
    2   2560       1    0.999 0.000528        0.998        1.000
    6   2054       5    0.997 0.001208        0.994        0.999
    7   1926       5    0.994 0.001669        0.991        0.998
    8   1811      10    0.989 0.002399        0.984        0.993
    9   1669      14    0.980 0.003245        0.974        0.987
   10   1511      19    0.968 0.004262        0.960        0.977
   11   1380      19    0.955 0.005186        0.945        0.965
   12   1267      16    0.943 0.005932        0.931        0.954
   13   1165      19    0.927 0.006804        0.914        0.941
   14   1057      19    0.911 0.007681        0.896        0.926
   15    973       1    0.910 0.007730        0.895        0.925
   16    913       2    0.908 0.007841        0.893        0.923
   17    879       1    0.907 0.007899        0.891        0.922
   18    849       2    0.905 0.008024        0.889        0.920
   19    802       3    0.901 0.008228        0.885        0.917
   20    775       2    0.899 0.008370        0.883        0.915
   21    756       4    0.894 0.008657        0.877        0.911
   22    700       2    0.892 0.008818        0.874        0.909
   23    681       9    0.880 0.009536        0.861        0.899
   24    654       3    0.876 0.009773        0.857        0.895
   25    635       5    0.869 0.010171        0.849        0.889
   27    575       2    0.866 0.010358        0.846        0.886
   28    551       3    0.861 0.010653        0.840        0.882
   34    483       1    0.859 0.010779        0.838        0.881
   35    455       4    0.852 0.011327        0.830        0.874
   41    391       1    0.850 0.011506        0.827        0.872
   42    364       3    0.843 0.012100        0.819        0.867
   49    288       5    0.828 0.013544        0.802        0.855
   55    212       1    0.824 0.014032        0.797        0.852
   56    189       4    0.807 0.016220        0.775        0.839
   63    129       5    0.775 0.020760        0.736        0.817
   68     62       2    0.750 0.026577        0.700        0.804
   69     46       1    0.734 0.030598        0.676        0.797
   70     23       2    0.670 0.051386        0.577        0.779

                Status=U 
 time n.risk n.event survival  std.err lower 95% CI upper 95% CI
    2   1271       1    0.999 0.000786        0.998            1
    3   1197       2    0.998 0.001417        0.995            1
```

```
    7    910       1    0.996 0.001790        0.993            1
   10    701       1    0.995 0.002283        0.991            1

Call: survfit(formula = Surv(Date.dpi, as.numeric(Paralysis_HL)) ~ 
    Strain, data = aData)

                Strain=CC002 
     time n.risk n.event survival std.err lower 95% CI upper 95% CI

                Strain=CC005 
 time n.risk n.event survival std.err lower 95% CI upper 95% CI
    7    138       1    0.993 0.00722        0.979            1
   13     66       1    0.978 0.01653        0.946            1
   14     56       2    0.943 0.02902        0.888            1

                Strain=CC006 
     time n.risk n.event survival std.err lower 95% CI upper 95% CI

                Strain=CC011 
     time n.risk n.event survival std.err lower 95% CI upper 95% CI

                Strain=CC012 
     time n.risk n.event survival std.err lower 95% CI upper 95% CI

                Strain=CC012XCC032 
 time n.risk n.event survival std.err lower 95% CI upper 95% CI
    2    188       1    0.995 0.00530        0.984            1
    3    177       2    0.983 0.00948        0.965            1

                Strain=CC013XCC041 
     time n.risk n.event survival std.err lower 95% CI upper 95% CI

                Strain=CC015 
     time n.risk n.event survival std.err lower 95% CI upper 95% CI

                Strain=CC017 
     time n.risk n.event survival std.err lower 95% CI upper 95% CI

                Strain=CC023 
 time n.risk n.event survival std.err lower 95% CI upper 95% CI
    2    189       1    0.995 0.00528       0.9844        1.000
    6    143       1    0.988 0.00869       0.9709        1.000
    7    131       5    0.950 0.01853       0.9144        0.987
    8    119       4    0.918 0.02381       0.8726        0.966
    9    107       5    0.875 0.02943       0.8194        0.935
   10     95       6    0.820 0.03517       0.7538        0.892
   11     83       8    0.741 0.04142       0.6640        0.827
   12     71       8    0.657 0.04609       0.5730        0.754
   13     59       5    0.602 0.04845       0.5139        0.705
   14     48       4    0.552 0.05048       0.4610        0.660
   23     37       2    0.522 0.05197       0.4292        0.634
   28     32       2    0.489 0.05359       0.3946        0.606
   35     27       2    0.453 0.05541       0.3563        0.576
   42     22       2    0.412 0.05752       0.3131        0.541
   49     16       2    0.360 0.06076       0.2589        0.501
   56     12       2    0.300 0.06376       0.1980        0.455
   63      8       2    0.225 0.06633       0.1264        0.401
   70      4       2    0.113 0.06534       0.0361        0.351

                Strain=CC025 
```

time	n.risk	n.event	survival	std.err	lower 95% CI	upper 95% CI

Strain=CC027

time	n.risk	n.event	survival	std.err	lower 95% CI	upper 95% CI

Strain=CC032XCC013

time	n.risk	n.event	survival	std.err	lower 95% CI	upper 95% CI

Strain=CC037

time	n.risk	n.event	survival	std.err	lower 95% CI	upper 95% CI

Strain=CC041XCC012

time	n.risk	n.event	survival	std.err	lower 95% CI	upper 95% CI
6	214	4	0.981	0.00926	0.963	1.000
8	193	2	0.971	0.01162	0.949	0.994
9	182	2	0.960	0.01373	0.934	0.988
10	160	3	0.942	0.01696	0.910	0.976
13	149	1	0.936	0.01798	0.902	0.972
21	128	1	0.929	0.01927	0.892	0.967
27	112	2	0.912	0.02221	0.870	0.957
34	96	1	0.903	0.02393	0.857	0.951
41	80	1	0.891	0.02615	0.842	0.944
49	64	1	0.878	0.02922	0.822	0.937
55	48	1	0.859	0.03385	0.795	0.928
63	32	1	0.832	0.04212	0.754	0.919
69	16	1	0.780	0.06400	0.664	0.916

Strain=CC051

time	n.risk	n.event	survival	std.err	lower 95% CI	upper 95% CI

Strain=CC057

time	n.risk	n.event	survival	std.err	lower 95% CI	upper 95% CI
8	354	2	0.994	0.00398	0.987	1.000
9	336	2	0.988	0.00575	0.977	1.000
10	318	2	0.982	0.00720	0.968	0.996
11	300	4	0.969	0.00963	0.950	0.988
12	282	1	0.966	0.01019	0.946	0.986
13	264	4	0.951	0.01239	0.927	0.976
14	248	7	0.924	0.01565	0.894	0.955
18	184	2	0.914	0.01702	0.881	0.948
19	168	3	0.898	0.01915	0.861	0.936
20	152	2	0.886	0.02064	0.846	0.927
21	136	3	0.866	0.02306	0.822	0.913
22	120	2	0.852	0.02484	0.805	0.902
23	104	6	0.803	0.03045	0.745	0.865
24	88	3	0.776	0.03326	0.713	0.844
25	72	4	0.732	0.03775	0.662	0.810
28	49	1	0.717	0.03983	0.644	0.800
35	42	1	0.700	0.04239	0.622	0.789
42	35	1	0.680	0.04565	0.597	0.776
49	28	1	0.656	0.05007	0.565	0.762
56	21	1	0.625	0.05660	0.523	0.746
68	7	1	0.536	0.09583	0.377	0.761

Strain=CC078

time	n.risk	n.event	survival	std.err	lower 95% CI	upper 95% CI
0	258	1	0.996	0.00387	0.989	1.000
8	245	2	0.988	0.00689	0.975	1.000
9	231	5	0.967	0.01162	0.944	0.990
10	217	9	0.927	0.01718	0.893	0.961

```
   11    203       7    0.895 0.02039        0.855        0.935
   12    190       7    0.862 0.02314        0.817        0.908
   13    178       8    0.823 0.02583        0.774        0.875
   14    166       6    0.793 0.02761        0.741        0.849
   15    154       1    0.788 0.02790        0.735        0.845
   16    142       2    0.777 0.02859        0.723        0.835
   17    130       1    0.771 0.02899        0.716        0.830
   23     69       1    0.760 0.03065        0.702        0.822
   25     49       1    0.744 0.03372        0.681        0.813
   35     30       1    0.719 0.04071        0.644        0.804
   49     20       1    0.683 0.05220        0.588        0.794
   56     15       1    0.638 0.06566        0.521        0.780
   63     10       2    0.510 0.09628        0.353        0.739
   68      5       1    0.408 0.11944        0.230        0.724

Call:
coxph(formula = Surv(Date.dpi, as.numeric(Paralysis_HL)) ~ Sex,
    data = aData)

  n= 4200, number of events= 201

       coef exp(coef) se(coef)     z Pr(>|z|)
SexM 0.5378    1.7122   0.1448 3.715 0.000203 ***
---
Signif. codes:  0 ‚Äò***‚Äô 0.001 ‚Äò**‚Äô 0.01 ‚Äò*‚Äô 0.05 ‚Äò.‚Äô 0.1 ‚Äò ‚Äô 1

     exp(coef) exp(-coef) lower .95 upper .95
SexM     1.712      0.584     1.289     2.274

Concordance= 0.562  (se = 0.019 )
Likelihood ratio test= 14.2  on 1 df,   p=2e-04
Wald test            = 13.8  on 1 df,   p=2e-04
Score (logrank) test = 14.14  on 1 df,   p=2e-04

Call:
coxph(formula = Surv(Date.dpi, as.numeric(Paralysis_HL)) ~ Status,
    data = aData)

  n= 4200, number of events= 201

            coef exp(coef) se(coef)      z Pr(>|z|)
StatusU -2.89042   0.05555  0.45289 -6.382 1.75e-10 ***
---
Signif. codes:  0 ‚Äò***‚Äô 0.001 ‚Äò**‚Äô 0.01 ‚Äò*‚Äô 0.05 ‚Äò.‚Äô 0.1 ‚Äò ‚Äô 1

        exp(coef) exp(-coef) lower .95 upper .95
StatusU   0.05555         18   0.02287     0.135

Concordance= 0.635  (se = 0.011 )
Likelihood ratio test= 112.8  on 1 df,   p=<2e-16
Wald test            = 40.73  on 1 df,   p=2e-10
Score (logrank) test = 78.27  on 1 df,   p=<2e-16

Call:
coxph(formula = Surv(Date.dpi, as.numeric(Paralysis_HL)) ~ Strain,
    data = Z)

  n= 4195, number of events= 4195
   (201 observations deleted due to missingness)
```

```
                     coef exp(coef) se(coef)     z Pr(>|z|)    
StrainCC078       0.10173   1.10708  0.10056 1.012 0.311716    
StrainCC057       0.16686   1.18159  0.09025 1.849 0.064470 .  
StrainCC012       0.49322   1.63759  0.08537 5.777 7.58e-09 ***
StrainCC041XCC012 0.17720   1.19387  0.09207 1.925 0.054279 .  
StrainCC002       0.33443   1.39715  0.09305 3.594 0.000326 ***
StrainCC025       0.34483   1.41175  0.10439 3.303 0.000955 ***
StrainCC012XCC032 0.14271   1.15339  0.10088 1.415 0.157176    
StrainCC013XCC041 0.13896   1.14908  0.10538 1.319 0.187262    
StrainCC032XCC013 0.30848   1.36135  0.09482 3.253 0.001141 ** 
StrainCC015       0.52407   1.68889  0.18364 2.854 0.004320 ** 
StrainCC023       0.70442   2.02267  0.10972 6.420 1.36e-10 ***
StrainCC027       0.47649   1.61041  0.09614 4.956 7.19e-07 ***
StrainCC005       0.39464   1.48385  0.09996 3.948 7.89e-05 ***
StrainCC011       0.41091   1.50819  0.09606 4.278 1.89e-05 ***
StrainCC051       0.83414   2.30282  0.10982 7.595 3.07e-14 ***
StrainCC037       0.63916   1.89488  0.10061 6.353 2.12e-10 ***
StrainCC006       0.60083   1.82363  0.09487 6.333 2.40e-10 ***
StrainCC017       0.40074   1.49293  0.11855 3.380 0.000724 ***
---
Signif. codes:  0 ,Äò***,Äô 0.001 ,Äò**,Äô 0.01 ,Äò*,Äô 0.05 ,Äò.,Äô 0.1 ,Äò ,Äô 1

                  exp(coef) exp(-coef) lower .95 upper .95
StrainCC078           1.107     0.9033    0.9090     1.348
StrainCC057           1.182     0.8463    0.9900     1.410
StrainCC012           1.638     0.6107    1.3853     1.936
StrainCC041XCC012     1.194     0.8376    0.9967     1.430
StrainCC002           1.397     0.7157    1.1642     1.677
StrainCC025           1.412     0.7083    1.1505     1.732
StrainCC012XCC032     1.153     0.8670    0.9465     1.406
StrainCC013XCC041     1.149     0.8703    0.9347     1.413
StrainCC032XCC013     1.361     0.7346    1.1305     1.639
StrainCC015           1.689     0.5921    1.1784     2.421
StrainCC023           2.023     0.4944    1.6313     2.508
StrainCC027           1.610     0.6210    1.3338     1.944
StrainCC005           1.484     0.6739    1.2198     1.805
StrainCC011           1.508     0.6630    1.2494     1.821
StrainCC051           2.303     0.4342    1.8569     2.856
StrainCC037           1.895     0.5277    1.5557     2.308
StrainCC006           1.824     0.5484    1.5142     2.196
StrainCC017           1.493     0.6698    1.1834     1.883

Concordance= 0.584  (se = 0.005 )
Likelihood ratio test= 168.2  on 18 df,   p=<2e-16
Wald test            = 171.3  on 18 df,   p=<2e-16
Score (logrank) test = 174.4  on 18 df,   p=<2e-16
```

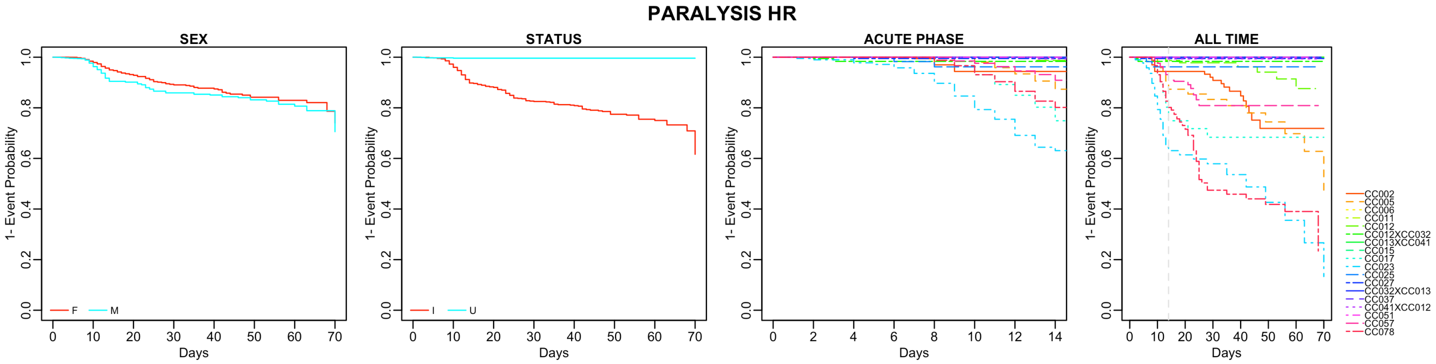


```
Call: survfit(formula = Surv(Date.dpi, as.numeric(Paralysis_HR)) ~
    Sex, data = aData)

                Sex=F
 time n.risk n.event survival  std.err lower 95% CI upper 95% CI
    4   1778       1    0.999 0.000562        0.998        1.000
    6   1583       3    0.998 0.001228        0.995        1.000
    7   1480       3    0.996 0.001692        0.992        0.999
    8   1383       7    0.990 0.002538        0.986        0.995
    9   1272       8    0.984 0.003344        0.978        0.991
   10   1154       6    0.979 0.003925        0.971        0.987
   11   1055       6    0.974 0.004514        0.965        0.982
   12    965      10    0.963 0.005479        0.953        0.974
   13    879       6    0.957 0.006064        0.945        0.969
   14    799       6    0.950 0.006691        0.937        0.963
   15    736       2    0.947 0.006917        0.934        0.961
   16    696       4    0.942 0.007393        0.927        0.956
   17    667       3    0.937 0.007754        0.922        0.953
   18    645       2    0.935 0.007998        0.919        0.950
   19    617       2    0.932 0.008254        0.915        0.948
   20    597       2    0.928 0.008516        0.912        0.945
   21    580       3    0.924 0.008912        0.906        0.941
   23    523       4    0.917 0.009518        0.898        0.935
   24    498       3    0.911 0.009980        0.892        0.931
   25    481       4    0.903 0.010591        0.883        0.924
   26    457       1    0.901 0.010751        0.881        0.923
   27    441       1    0.899 0.010919        0.878        0.921
   28    422       2    0.895 0.011276        0.873        0.918
   29    395       1    0.893 0.011473        0.871        0.916
   30    387       1    0.891 0.011673        0.868        0.914
   33    366       1    0.888 0.011892        0.865        0.912
   34    357       1    0.886 0.012116        0.862        0.910
   35    335       2    0.880 0.012607        0.856        0.905
   36    310       1    0.878 0.012883        0.853        0.903
   40    291       1    0.875 0.013187        0.849        0.901
   41    282       1    0.871 0.013500        0.845        0.898
   42    263       3    0.861 0.014514        0.833        0.890
   43    234       1    0.858 0.014912        0.829        0.888
   44    229       1    0.854 0.015310        0.825        0.885
   47    214       1    0.850 0.015750        0.820        0.881
   49    205       2    0.842 0.016652        0.810        0.875
   56    137       2    0.829 0.018538        0.794        0.867
   63     94       1    0.821 0.020333        0.782        0.861
   68     48       2    0.786 0.030658        0.729        0.849
   70     17       1    0.740 0.053356        0.643        0.853

                Sex=M
 time n.risk n.event survival  std.err lower 95% CI upper 95% CI
```

```
    1   1919       1    0.999 0.000521        0.998        1.000
    2   1838       2    0.998 0.000928        0.997        1.000
    3   1734       2    0.997 0.001234        0.995        1.000
    4   1633       2    0.996 0.001504        0.993        0.999
    5   1540       1    0.995 0.001637        0.992        0.999
    6   1454       1    0.995 0.001773        0.991        0.998
    7   1356       1    0.994 0.001917        0.990        0.998
    8   1270       7    0.988 0.002811        0.983        0.994
    9   1169      14    0.977 0.004195        0.968        0.985
   10   1058      15    0.963 0.005450        0.952        0.974
   11    964      12    0.951 0.006387        0.938        0.963
   12    883      13    0.937 0.007379        0.922        0.951
   13    809      17    0.917 0.008632        0.900        0.934
   14    730      10    0.905 0.009383        0.886        0.923
   18    587       2    0.901 0.009601        0.883        0.920
   21    525       4    0.895 0.010123        0.875        0.915
   22    487       2    0.891 0.010410        0.871        0.912
   23    473       6    0.880 0.011254        0.858        0.902
   24    452       3    0.874 0.011673        0.851        0.897
   25    438       4    0.866 0.012229        0.842        0.890
   28    384       3    0.859 0.012742        0.834        0.884
   35    313       2    0.854 0.013239        0.828        0.880
   39    286       1    0.851 0.013525        0.824        0.878
   42    252       2    0.844 0.014235        0.816        0.872
   46    219       1    0.840 0.014682        0.812        0.869
   49    199       2    0.832 0.015701        0.801        0.863
   53    163       1    0.826 0.016413        0.795        0.859
   56    134       2    0.814 0.018339        0.779        0.851
   60    108       1    0.807 0.019658        0.769        0.846
   63     90       2    0.789 0.022946        0.745        0.835
   70     19       2    0.706 0.059198        0.599        0.832

Call: survfit(formula = Surv(Date.dpi, as.numeric(Paralysis_HR)) ~ 
    Status, data = aData)

                Status=I 
 time n.risk n.event survival  std.err lower 95% CI upper 95% CI
    1   2667       1    1.000 0.000375        0.999        1.000
    2   2560       1    0.999 0.000541        0.998        1.000
    4   2287       3    0.998 0.000930        0.996        1.000
    5   2165       1    0.997 0.001037        0.995        0.999
    6   2054       4    0.996 0.001419        0.993        0.998
    7   1926       4    0.993 0.001752        0.990        0.997
    8   1811      14    0.986 0.002684        0.981        0.991
    9   1669      22    0.973 0.003820        0.965        0.980
   10   1511      20    0.960 0.004731        0.951        0.969
   11   1380      18    0.947 0.005514        0.937        0.958
   12   1267      23    0.930 0.006476        0.918        0.943
   13   1165      23    0.912 0.007394        0.897        0.926
   14   1057      16    0.898 0.008047        0.882        0.914
   15    973       2    0.896 0.008135        0.880        0.912
   16    913       4    0.892 0.008333        0.876        0.909
   17    879       3    0.889 0.008488        0.873        0.906
   18    849       4    0.885 0.008703        0.868        0.902
   19    802       2    0.883 0.008820        0.866        0.900
   20    775       2    0.881 0.008943        0.863        0.898
   21    756       7    0.872 0.009376        0.854        0.891
   22    700       2    0.870 0.009514        0.851        0.889
   23    681      10    0.857 0.010195        0.837        0.877
   24    654       6    0.849 0.010595        0.829        0.870
```

```
   25    635       8    0.839 0.011117        0.817        0.861
   26    598       1    0.837 0.011186        0.816        0.859
   27    575       1    0.836 0.011261        0.814        0.858
   28    551       5    0.828 0.011658        0.806        0.851
   29    525       1    0.827 0.011742        0.804        0.850
   30    519       1    0.825 0.011827        0.802        0.848
   33    493       1    0.823 0.011921        0.800        0.847
   34    483       1    0.822 0.012017        0.798        0.845
   35    455       4    0.814 0.012443        0.790        0.839
   36    429       1    0.812 0.012558        0.788        0.837
   39    420       1    0.811 0.012676        0.786        0.836
   40    401       1    0.808 0.012804        0.784        0.834
   41    391       1    0.806 0.012937        0.781        0.832
   42    364       5    0.795 0.013675        0.769        0.823
   43    328       1    0.793 0.013847        0.766        0.821
   44    323       1    0.790 0.014020        0.763        0.818
   46    318       1    0.788 0.014194        0.761        0.816
   47    299       1    0.785 0.014390        0.758        0.814
   49    288       4    0.774 0.015188        0.745        0.805
   53    235       1    0.771 0.015477        0.741        0.802
   56    189       4    0.755 0.017166        0.722        0.789
   60    154       1    0.750 0.017741        0.716        0.785
   63    129       3    0.732 0.019982        0.694        0.773
   68     62       2    0.709 0.025379        0.661        0.760
   70     23       3    0.616 0.054450        0.518        0.733

                Status=U
 time n.risk n.event survival  std.err lower 95% CI upper 95% CI
    2   1271       1    0.999 0.000786        0.998            1
    3   1197       2    0.998 0.001417        0.995            1
   10    701       1    0.996 0.002006        0.992            1

Call: survfit(formula = Surv(Date.dpi, as.numeric(Paralysis_HR)) ~
    Strain, data = aData)

                Strain=CC002
 time n.risk n.event survival std.err lower 95% CI upper 95% CI
    7    178       1    0.994  0.0056        0.983        1.000
    8    162       4    0.970  0.0133        0.944        0.996
    9    148       4    0.944  0.0183        0.908        0.980
   27     87       1    0.933  0.0211        0.892        0.975
   29     78       1    0.921  0.0239        0.875        0.969
   30     74       1    0.908  0.0267        0.858        0.962
   33     70       1    0.895  0.0293        0.840        0.955
   34     66       1    0.882  0.0318        0.822        0.946
   36     54       1    0.865  0.0352        0.799        0.937
   40     47       1    0.847  0.0389        0.774        0.927
   41     43       1    0.827  0.0427        0.748        0.916
   42     39       1    0.806  0.0466        0.720        0.903
   43     31       1    0.780  0.0518        0.685        0.889
   44     27       1    0.751  0.0574        0.647        0.873
   47     23       1    0.719  0.0635        0.604        0.855

                Strain=CC005
 time n.risk n.event survival std.err lower 95% CI upper 95% CI
   10    102       2    0.980  0.0137        0.954        1.000
   11     90       2    0.959  0.0203        0.920        0.999
   12     78       2    0.934  0.0262        0.884        0.987
   13     66       2    0.906  0.0321        0.845        0.971
   14     56       2    0.873  0.0383        0.801        0.952
```

```
   21     46       1    0.854  0.0419        0.776        0.941
   28     40       1    0.833  0.0460        0.748        0.928
   35     34       1    0.809  0.0507        0.715        0.914
   42     28       1    0.780  0.0565        0.676        0.899
   49     22       1    0.744  0.0641        0.629        0.881
   56     16       1    0.698  0.0751        0.565        0.862
   63     10       1    0.628  0.0946        0.467        0.844
   70      4       1    0.471  0.1534        0.249        0.892

                Strain=CC006
     time n.risk n.event survival std.err lower 95% CI upper 95% CI

                Strain=CC011
     time n.risk n.event survival std.err lower 95% CI upper 95% CI

                Strain=CC012
 time n.risk n.event survival std.err lower 95% CI upper 95% CI
   13    156       2    0.987 0.00901        0.970        1.000
   18    108       1    0.978 0.01274        0.953        1.000
   39     59       1    0.961 0.02067        0.922        1.000
   46     47       1    0.941 0.02861        0.887        0.999
   53     35       1    0.914 0.03840        0.842        0.993
   60     24       1    0.876 0.05239        0.779        0.985

                Strain=CC012XCC032
 time n.risk n.event survival std.err lower 95% CI upper 95% CI
    2    188       1    0.995 0.00530        0.984            1
    3    177       2    0.983 0.00948        0.965            1

                Strain=CC013XCC041
     time n.risk n.event survival std.err lower 95% CI upper 95% CI

                Strain=CC015
     time n.risk n.event survival std.err lower 95% CI upper 95% CI

                Strain=CC017
 time n.risk n.event survival std.err lower 95% CI upper 95% CI
    9     60       2    0.967  0.0232        0.922        1.000
   10     54       2    0.931  0.0334        0.868        0.999
   11     48       2    0.892  0.0418        0.814        0.978
   12     42       2    0.850  0.0494        0.758        0.952
   13     36       2    0.802  0.0568        0.698        0.922
   14     30       2    0.749  0.0644        0.633        0.886
   21     24       1    0.718  0.0689        0.595        0.866
   28     21       1    0.684  0.0736        0.553        0.844

                Strain=CC023
 time n.risk n.event survival std.err lower 95% CI upper 95% CI
    1    201       1    0.995 0.00496        0.985        1.000
    2    189       1    0.990 0.00721        0.976        1.000
    4    167       2    0.978 0.01096        0.957        1.000
    5    155       1    0.972 0.01257        0.947        0.997
    6    143       2    0.958 0.01564        0.928        0.989
    7    131       3    0.936 0.01976        0.898        0.976
    8    119       5    0.897 0.02559        0.848        0.948
    9    107       6    0.846 0.03132        0.787        0.910
   10     95       6    0.793 0.03616        0.725        0.867
   11     83       4    0.755 0.03914        0.682        0.836
   12     71       6    0.691 0.04364        0.611        0.782
   13     59       4    0.644 0.04655        0.559        0.742
```

```
   14     48       1    0.631 0.04747        0.544        0.731
   18     38       1    0.614 0.04904        0.525        0.718
   23     37       1    0.598 0.05044        0.506        0.705
   28     32       1    0.579 0.05221        0.485        0.691
   35     27       2    0.536 0.05646        0.436        0.659
   42     22       2    0.487 0.06094        0.381        0.623
   49     16       2    0.426 0.06683        0.314        0.580
   56     12       2    0.355 0.07215        0.239        0.529
   63      8       2    0.266 0.07672        0.152        0.469
   70      4       2    0.133 0.07687        0.043        0.413

                Strain=CC025
 time n.risk n.event survival std.err lower 95% CI upper 95% CI
    6    115       2    0.983  0.0122        0.959        1.000
    8     95       2    0.962  0.0188        0.926        0.999

                Strain=CC027
        time       n.risk      n.event     survival      std.err lower 95% CI upper
95% CI
    4.00e+00     1.98e+02     1.00e+00     9.95e-01     5.04e-03     9.85e-01
1.00e+00

                Strain=CC032XCC013
     time n.risk n.event survival std.err lower 95% CI upper 95% CI

                Strain=CC037
     time n.risk n.event survival std.err lower 95% CI upper 95% CI

                Strain=CC041XCC012
        time       n.risk      n.event     survival      std.err lower 95% CI upper
95% CI
    1.30e+01     1.49e+02     1.00e+00     9.93e-01     6.69e-03     9.80e-01
1.00e+00

                Strain=CC051
     time n.risk n.event survival std.err lower 95% CI upper 95% CI

                Strain=CC057
 time n.risk n.event survival std.err lower 95% CI upper 95% CI
    9    336       5    0.985 0.00661        0.972        0.998
   10    318       3    0.976 0.00845        0.959        0.993
   11    300       4    0.963 0.01054        0.942        0.984
   12    282       5    0.946 0.01283        0.921        0.971
   13    264       4    0.931 0.01450        0.903        0.960
   14    248       6    0.909 0.01681        0.877        0.942
   16    216       1    0.905 0.01725        0.871        0.939
   21    136       2    0.891 0.01940        0.854        0.930
   22    120       2    0.877 0.02173        0.835        0.920
   23    104       3    0.851 0.02554        0.803        0.903
   24     88       2    0.832 0.02839        0.778        0.889
   25     72       2    0.809 0.03196        0.749        0.874

                Strain=CC078
 time n.risk n.event survival std.err lower 95% CI upper 95% CI
    8    245       3    0.988 0.00703        0.974        1.000
    9    231       5    0.966 0.01169        0.944        0.990
   10    217       8    0.931 0.01672        0.899        0.964
   11    203       6    0.903 0.01964        0.866        0.943
   12    190       8    0.865 0.02296        0.821        0.911
   13    178       8    0.826 0.02572        0.777        0.878
```

```
   14    166       5    0.801 0.02724        0.750        0.857
   15    154       2    0.791 0.02787        0.738        0.848
   16    142       3    0.774 0.02890        0.720        0.833
   17    130       3    0.756 0.03002        0.700        0.818
   18    119       2    0.744 0.03083        0.686        0.807
   19    109       2    0.730 0.03174        0.670        0.795
   20     99       2    0.715 0.03277        0.654        0.783
   21     89       3    0.691 0.03449        0.627        0.762
   23     69       6    0.631 0.03926        0.559        0.713
   24     59       4    0.588 0.04203        0.511        0.677
   25     49       6    0.516 0.04604        0.434        0.615
   26     39       1    0.503 0.04672        0.419        0.603
   28     35       2    0.474 0.04827        0.389        0.579
   35     30       1    0.458 0.04918        0.372        0.566
   42     25       1    0.440 0.05052        0.351        0.551
   49     20       1    0.418 0.05257        0.327        0.535
   56     15       1    0.390 0.05597        0.295        0.517
   68      5       2    0.234 0.09186        0.109        0.505

Call:
coxph(formula = Surv(Date.dpi, as.numeric(Paralysis_HR)) ~ Sex,
    data = aData)

  n= 4200, number of events= 250

       coef exp(coef) se(coef)     z Pr(>|z|)
SexM 0.2917    1.3387   0.1272 2.293   0.0218 *
---
Signif. codes:  0 ‚Äò***‚Äô 0.001 ‚Äò**‚Äô 0.01 ‚Äò*‚Äô 0.05 ‚Äò.‚Äô 0.1 ‚Äò ‚Äô 1

     exp(coef) exp(-coef) lower .95 upper .95
SexM     1.339      0.747     1.043     1.718

Concordance= 0.556  (se = 0.017 )
Likelihood ratio test= 5.29  on 1 df,   p=0.02
Wald test            = 5.26  on 1 df,   p=0.02
Score (logrank) test = 5.3  on 1 df,   p=0.02

Call:
coxph(formula = Surv(Date.dpi, as.numeric(Paralysis_HR)) ~ Status,
    data = aData)

  n= 4200, number of events= 250

            coef exp(coef) se(coef)      z Pr(>|z|)
StatusU -3.33914   0.03547  0.50406 -6.624 3.48e-11 ***
---
Signif. codes:  0 ‚Äò***‚Äô 0.001 ‚Äò**‚Äô 0.01 ‚Äò*‚Äô 0.05 ‚Äò.‚Äô 0.1 ‚Äò ‚Äô 1

        exp(coef) exp(-coef) lower .95 upper .95
StatusU   0.03547      28.19   0.01321   0.09525

Concordance= 0.643  (se = 0.009 )
Likelihood ratio test= 153.8  on 1 df,   p=<2e-16
Wald test            = 43.88  on 1 df,   p=3e-11
Score (logrank) test = 103.2  on 1 df,   p=<2e-16

Call:
coxph(formula = Surv(Date.dpi, as.numeric(Paralysis_HR)) ~ Strain,
    data = Z)
```

```
  n= 4146, number of events= 4146 
   (250 observations deleted due to missingness)

                     coef exp(coef) se(coef)     z Pr(>|z|)    
StrainCC078       0.11604   1.12304  0.10449 1.110 0.266791    
StrainCC057       0.13511   1.14466  0.08956 1.509 0.131407    
StrainCC012       0.50893   1.66352  0.08558 5.947 2.73e-09 ***
StrainCC041XCC012 0.16796   1.18289  0.09096 1.847 0.064796 .  
StrainCC002       0.35979   1.43303  0.09459 3.804 0.000143 ***
StrainCC025       0.33100   1.39236  0.10504 3.151 0.001626 ** 
StrainCC012XCC032 0.14103   1.15146  0.10088 1.398 0.162120    
StrainCC013XCC041 0.13717   1.14702  0.10538 1.302 0.193038    
StrainCC032XCC013 0.30759   1.36015  0.09482 3.244 0.001179 ** 
StrainCC015       0.52269   1.68656  0.18364 2.846 0.004424 ** 
StrainCC023       0.65612   1.92729  0.10846 6.049 1.45e-09 ***
StrainCC027       0.47096   1.60154  0.09623 4.894 9.88e-07 ***
StrainCC005       0.46746   1.59593  0.10168 4.597 4.28e-06 ***
StrainCC011       0.40886   1.50510  0.09607 4.256 2.08e-05 ***
StrainCC051       0.83035   2.29412  0.10983 7.560 4.02e-14 ***
StrainCC037       0.63640   1.88967  0.10062 6.325 2.53e-10 ***
StrainCC006       0.59762   1.81779  0.09488 6.299 3.00e-10 ***
StrainCC017       0.39073   1.47806  0.12379 3.156 0.001597 ** 
---
Signif. codes:  0 ,Äò***,Äô 0.001 ,Äò**,Äô 0.01 ,Äò*,Äô 0.05 ,Äò.,Äô 0.1 ,Äò ,Äô 1

                  exp(coef) exp(-coef) lower .95 upper .95
StrainCC078           1.123     0.8904    0.9151     1.378
StrainCC057           1.145     0.8736    0.9604     1.364
StrainCC012           1.664     0.6011    1.4066     1.967
StrainCC041XCC012     1.183     0.8454    0.9897     1.414
StrainCC002           1.433     0.6978    1.1905     1.725
StrainCC025           1.392     0.7182    1.1333     1.711
StrainCC012XCC032     1.151     0.8685    0.9449     1.403
StrainCC013XCC041     1.147     0.8718    0.9330     1.410
StrainCC032XCC013     1.360     0.7352    1.1295     1.638
StrainCC015           1.687     0.5929    1.1768     2.417
StrainCC023           1.927     0.5189    1.5582     2.384
StrainCC027           1.602     0.6244    1.3262     1.934
StrainCC005           1.596     0.6266    1.3076     1.948
StrainCC011           1.505     0.6644    1.2468     1.817
StrainCC051           2.294     0.4359    1.8498     2.845
StrainCC037           1.890     0.5292    1.5515     2.302
StrainCC006           1.818     0.5501    1.5093     2.189
StrainCC017           1.478     0.6766    1.1596     1.884

Concordance= 0.585  (se = 0.005 )
Likelihood ratio test= 171.3  on 18 df,   p=<2e-16
Wald test            = 173.3  on 18 df,   p=<2e-16
Score (logrank) test = 176.4  on 18 df,   p=<2e-16
```

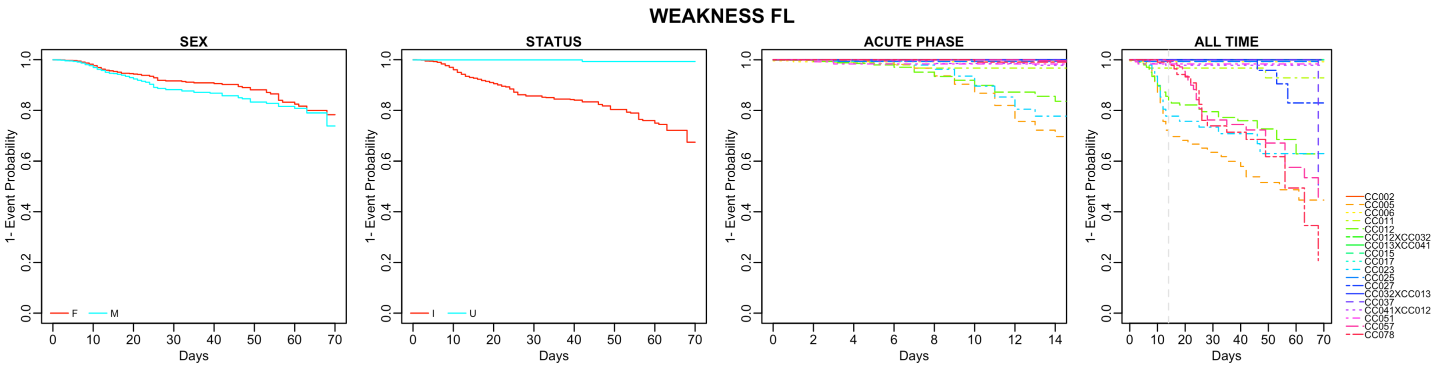


```
Call: survfit(formula = Surv(Date.dpi, as.numeric(Weakness_FL)) ~ Sex,
    data = aData)
```

Sex=F

time	n.risk	n.event	survival	std.err	lower 95% CI	upper 95% CI
0	2190	1	1.000	0.000457	0.999	1.000
2	1993	1	0.999	0.000678	0.998	1.000
3	1883	4	0.997	0.001257	0.994	0.999
5	1678	1	0.996	0.001390	0.994	0.999
6	1583	3	0.994	0.001764	0.991	0.998
7	1480	6	0.990	0.002405	0.986	0.995
8	1383	5	0.987	0.002880	0.981	0.992
9	1272	6	0.982	0.003437	0.975	0.989
10	1154	7	0.976	0.004088	0.968	0.984
11	1055	8	0.969	0.004822	0.959	0.978
12	965	6	0.963	0.005383	0.952	0.973
13	879	4	0.958	0.005787	0.947	0.970
14	799	2	0.956	0.006016	0.944	0.968
15	736	2	0.953	0.006274	0.941	0.966
16	696	2	0.951	0.006548	0.938	0.964
17	667	3	0.946	0.006969	0.933	0.960
19	617	1	0.945	0.007124	0.931	0.959
20	597	1	0.943	0.007286	0.929	0.958
21	580	1	0.942	0.007453	0.927	0.956
22	540	2	0.938	0.007822	0.923	0.954
24	498	2	0.934	0.008232	0.918	0.951
25	481	3	0.929	0.008842	0.911	0.946
26	457	5	0.918	0.009843	0.899	0.938
28	422	1	0.916	0.010058	0.897	0.936
32	376	1	0.914	0.010322	0.894	0.934
33	366	1	0.911	0.010591	0.891	0.932
35	335	1	0.909	0.010904	0.887	0.930
40	291	1	0.905	0.011304	0.884	0.928
42	263	1	0.902	0.011774	0.879	0.925
46	224	2	0.894	0.012973	0.869	0.920
47	214	1	0.890	0.013568	0.864	0.917
49	205	2	0.881	0.014759	0.853	0.911
53	167	2	0.871	0.016360	0.839	0.903
54	158	1	0.865	0.017159	0.832	0.899
56	137	4	0.840	0.020792	0.800	0.882
57	113	1	0.832	0.021896	0.791	0.876
60	111	1	0.825	0.022947	0.781	0.871
61	103	1	0.817	0.024081	0.771	0.865
63	94	2	0.799	0.026520	0.749	0.853
68	48	1	0.783	0.030756	0.725	0.845

Sex=M

time	n.risk	n.event	survival	std.err	lower 95% CI	upper 95% CI
0	2010	1	1.000	0.000497	0.999	1.000

```
    1   1919       1    0.999 0.000720        0.998        1.000
    2   1838       2    0.998 0.001052        0.996        1.000
    3   1734       4    0.996 0.001557        0.993        0.999
    4   1633       1    0.995 0.001671        0.992        0.998
    5   1540       3    0.993 0.002008        0.989        0.997
    6   1454       3    0.991 0.002326        0.986        0.996
    7   1356       6    0.987 0.002925        0.981        0.992
    8   1270       8    0.980 0.003639        0.973        0.988
    9   1169       5    0.976 0.004078        0.968        0.984
   10   1058       8    0.969 0.004811        0.959        0.978
   11    964       7    0.962 0.005461        0.951        0.973
   12    883       4    0.957 0.005855        0.946        0.969
   13    809       6    0.950 0.006490        0.938        0.963
   14    730       3    0.946 0.006843        0.933        0.960
   15    669       1    0.945 0.006978        0.931        0.959
   16    631       2    0.942 0.007270        0.928        0.956
   17    607       2    0.939 0.007570        0.924        0.954
   18    587       3    0.934 0.008022        0.919        0.950
   19    558       3    0.929 0.008487        0.913        0.946
   20    539       3    0.924 0.008950        0.907        0.942
   21    525       3    0.919 0.009403        0.900        0.937
   22    487       2    0.915 0.009736        0.896        0.934
   23    473       2    0.911 0.010071        0.891        0.931
   24    452       4    0.903 0.010759        0.882        0.924
   25    438       6    0.891 0.011737        0.868        0.914
   26    414       2    0.886 0.012068        0.863        0.910
   28    384       2    0.882 0.012439        0.858        0.906
   32    349       2    0.877 0.012870        0.852        0.902
   35    313       2    0.871 0.013383        0.845        0.898
   39    286       1    0.868 0.013679        0.842        0.895
   42    252       3    0.858 0.014760        0.829        0.887
   46    219       2    0.850 0.015629        0.820        0.881
   47    208       1    0.846 0.016079        0.815        0.878
   49    199       3    0.833 0.017441        0.799        0.868
   53    163       1    0.828 0.018067        0.793        0.864
   56    134       2    0.815 0.019797        0.778        0.855
   60    108       1    0.808 0.021004        0.768        0.850
   63     90       2    0.790 0.024071        0.744        0.839
   68     46       3    0.738 0.036515        0.670        0.814

Call: survfit(formula = Surv(Date.dpi, as.numeric(Weakness_FL)) ~ Status,
    data = aData)

                Status=I
 time n.risk n.event survival  std.err lower 95% CI upper 95% CI
    0   2809       1    1.000 0.000356        0.999        1.000
    1   2667       1    0.999 0.000517        0.998        1.000
    2   2560       3    0.998 0.000850        0.996        1.000
    3   2420       8    0.995 0.001440        0.992        0.998
    4   2287       1    0.994 0.001504        0.991        0.997
    5   2165       4    0.993 0.001759        0.989        0.996
    6   2054       6    0.990 0.002115        0.985        0.994
    7   1926      12    0.983 0.002751        0.978        0.989
    8   1811      13    0.976 0.003356        0.970        0.983
    9   1669      11    0.970 0.003855        0.962        0.978
   10   1511      15    0.960 0.004548        0.951        0.969
   11   1380      15    0.950 0.005237        0.940        0.960
   12   1267      10    0.942 0.005707        0.931        0.954
   13   1165      10    0.934 0.006205        0.922        0.947
   14   1057       5    0.930 0.006482        0.917        0.943
```

```
   15    973        3    0.927 0.006670        0.914        0.940
   16    913        4    0.923 0.006943        0.909        0.937
   17    879        5    0.918 0.007290        0.904        0.932
   18    849        3    0.914 0.007501        0.900        0.929
   19    802        4    0.910 0.007803        0.895        0.925
   20    775        4    0.905 0.008108        0.889        0.921
   21    756        4    0.900 0.008411        0.884        0.917
   22    700        4    0.895 0.008748        0.878        0.913
   23    681        2    0.893 0.008917        0.875        0.910
   24    654        6    0.884 0.009442        0.866        0.903
   25    635        9    0.872 0.010191        0.852        0.892
   26    598        7    0.862 0.010777        0.841        0.883
   28    551        3    0.857 0.011053        0.836        0.879
   32    512        3    0.852 0.011362        0.830        0.875
   33    493        1    0.850 0.011470        0.828        0.873
   35    455        3    0.845 0.011842        0.822        0.868
   39    420        1    0.843 0.011984        0.819        0.866
   40    401        1    0.841 0.012137        0.817        0.865
   42    364        3    0.834 0.012678        0.809        0.859
   46    318        4    0.823 0.013560        0.797        0.850
   47    299        2    0.818 0.014017        0.791        0.846
   49    288        5    0.803 0.015143        0.774        0.834
   53    235        3    0.793 0.016066        0.762        0.825
   54    217        1    0.790 0.016402        0.758        0.822
   56    189        6    0.764 0.018804        0.728        0.802
   57    158        1    0.760 0.019298        0.723        0.798
   60    154        2    0.750 0.020269        0.711        0.791
   61    139        1    0.744 0.020828        0.705        0.786
   63    129        4    0.721 0.023160        0.677        0.768
   68     62        4    0.675 0.031239        0.616        0.739

                Status=U
  time n.risk n.event survival  std.err lower 95% CI upper 95% CI
     0   1391       1    0.999 0.000719        0.998            1
    42    151       1    0.993 0.006634        0.980            1

Call: survfit(formula = Surv(Date.dpi, as.numeric(Weakness_FL)) ~ Strain,
    data = aData)

                Strain=CC002
     time n.risk n.event survival std.err lower 95% CI upper 95% CI

                Strain=CC005
  time n.risk n.event survival std.err lower 95% CI upper 95% CI
     3    186       1    0.995 0.00536       0.984        1.000
     6    150       2    0.981 0.01071       0.961        1.000
     7    138       2    0.967 0.01453       0.939        0.996
     8    126       4    0.936 0.02064       0.897        0.978
     9    114       4    0.904 0.02564       0.855        0.955
    10    102       4    0.868 0.03014       0.811        0.929
    11     90       5    0.820 0.03535       0.753        0.892
    12     78       6    0.757 0.04095       0.681        0.842
    13     66       3    0.722 0.04364       0.642        0.813
    14     56       2    0.697 0.04573       0.613        0.792
    19     48       1    0.682 0.04703       0.596        0.781
    21     46       1    0.667 0.04829       0.579        0.769
    26     42       1    0.651 0.04968       0.561        0.756
    28     40       1    0.635 0.05104       0.543        0.743
    33     36       1    0.617 0.05258       0.523        0.730
    35     34       1    0.599 0.05408       0.502        0.715
```

```
   40     30       1    0.579 0.05585        0.480        0.700
   42     28       2    0.538 0.05903        0.434        0.667
   47     24       1    0.516 0.06068        0.409        0.649
   54     18       1    0.487 0.06371        0.377        0.629
   61     12       1    0.446 0.07014        0.328        0.607

                Strain=CC006 
 time n.risk n.event survival std.err lower 95% CI upper 95% CI
    0    262       1    0.996 0.00381        0.989            1
    1    250       1    0.992 0.00550        0.981            1

                Strain=CC011 
 time n.risk n.event survival std.err lower 95% CI upper 95% CI
    3    216       2    0.991 0.00652        0.978        1.000
    5    187       1    0.985 0.00836        0.969        1.000
    6    173       1    0.980 0.01007        0.960        1.000
    7    159       2    0.967 0.01318        0.942        0.994
   49     25       1    0.929 0.03997        0.854        1.000

                Strain=CC012 
 time n.risk n.event survival std.err lower 95% CI upper 95% CI
    0    468       1    0.998 0.00213        0.994        1.000
    2    420       2    0.993 0.00397        0.985        1.000
    3    396       2    0.988 0.00530        0.978        0.999
    4    372       1    0.985 0.00592        0.974        0.997
    5    348       2    0.980 0.00711        0.966        0.994
    6    324       3    0.971 0.00876        0.954        0.988
    7    300       6    0.951 0.01163        0.929        0.974
    8    276       5    0.934 0.01374        0.908        0.961
    9    252       4    0.919 0.01539        0.890        0.950
   10    228       5    0.899 0.01750        0.865        0.934
   11    204       6    0.873 0.02004        0.834        0.913
   13    156       3    0.856 0.02187        0.814        0.900
   14    132       3    0.836 0.02408        0.791        0.885
   15    120       1    0.829 0.02487        0.782        0.880
   18    108       1    0.822 0.02580        0.773        0.874
   25     96       2    0.805 0.02796        0.752        0.861
   26     83       1    0.795 0.02925        0.740        0.854
   32     71       2    0.773 0.03243        0.712        0.839
   39     59       1    0.759 0.03442        0.695        0.830
   46     47       2    0.727 0.03983        0.653        0.810
   53     35       2    0.686 0.04716        0.599        0.785
   60     24       2    0.628 0.05801        0.524        0.753

                Strain=CC012XCC032 
     time n.risk n.event survival std.err lower 95% CI upper 95% CI

                Strain=CC013XCC041 
     time n.risk n.event survival std.err lower 95% CI upper 95% CI

                Strain=CC015 
     time n.risk n.event survival std.err lower 95% CI upper 95% CI

                Strain=CC017 
     time n.risk n.event survival std.err lower 95% CI upper 95% CI

                Strain=CC023 
 time n.risk n.event survival std.err lower 95% CI upper 95% CI
    3    177       1    0.994 0.00563        0.983        1.000
    7    131       2    0.979 0.01201        0.956        1.000
```

```
    8    119       2    0.963 0.01651        0.931        0.996
    9    107       3    0.936 0.02222        0.893        0.980
   10     95       4    0.896 0.02872        0.842        0.954
   11     83       4    0.853 0.03451        0.788        0.924
   12     71       4    0.805 0.04007        0.730        0.888
   13     59       2    0.778 0.04311        0.698        0.867
   18     38       1    0.757 0.04658        0.671        0.854
   25     33       1    0.734 0.05051        0.642        0.840
   32     28       1    0.708 0.05509        0.608        0.825
   46     18       1    0.669 0.06457        0.553        0.808
   47     17       1    0.629 0.07176        0.503        0.787

                Strain=CC025
        time        n.risk      n.event     survival      std.err lower 95% CI upper
95% CI
    3.00e+00     1.44e+02     1.00e+00     9.93e-01     6.92e-03     9.80e-01
1.00e+00

                Strain=CC027
 time n.risk n.event survival std.err lower 95% CI upper 95% CI
   46     24       1    0.958  0.0408        0.882            1
   53     18       1    0.905  0.0645        0.787            1
   57     12       1    0.830  0.0933        0.665            1

                Strain=CC032XCC013
     time n.risk n.event survival std.err lower 95% CI upper 95% CI

                Strain=CC037
        time        n.risk      n.event     survival      std.err lower 95% CI upper
95% CI
      68.000        2.000        1.000        0.500        0.354        0.125
1.000

                Strain=CC041XCC012
 time n.risk n.event survival std.err lower 95% CI upper 95% CI
    5    225       1    0.996 0.00443        0.987            1
    8    193       2    0.985 0.00848        0.969            1
   13    149       1    0.979 0.01070        0.958            1

                Strain=CC051
 time n.risk n.event survival std.err lower 95% CI upper 95% CI
    2    128       1    0.992 0.00778        0.977            1
    3    118       1    0.984 0.01139        0.962            1

                Strain=CC057
 time n.risk n.event survival std.err lower 95% CI upper 95% CI
   13    264       1    0.996 0.00378        0.989        1.000
   16    216       2    0.987 0.00750        0.972        1.000
   17    200       2    0.977 0.01016        0.957        0.997
   18    184       1    0.972 0.01141        0.950        0.994
   19    168       3    0.954 0.01497        0.926        0.984
   20    152       3    0.936 0.01820        0.901        0.972
   21    136       3    0.915 0.02135        0.874        0.958
   22    120       2    0.900 0.02356        0.855        0.947
   23    104       2    0.882 0.02609        0.833        0.935
   24     88       4    0.842 0.03169        0.782        0.907
   25     72       3    0.807 0.03627        0.739        0.882
   26     56       2    0.778 0.04030        0.703        0.862
   28     49       1    0.763 0.04249        0.684        0.851
   35     42       1    0.744 0.04519        0.661        0.838
```

```
   42     35       1    0.723 0.04865        0.634        0.825
   49     28       2    0.671 0.05727        0.568        0.794
   56     21       3    0.576 0.07098        0.452        0.733
   63     14       1    0.534 0.07690        0.403        0.709
   68      7       1    0.458 0.09664        0.303        0.693

                Strain=CC078
 time n.risk n.event survival std.err lower 95% CI upper 95% CI
   10    217       2    0.991 0.00649        0.978        1.000
   15    154       2    0.978 0.01108        0.956        1.000
   16    142       2    0.964 0.01459        0.936        0.993
   17    130       3    0.942 0.01909        0.905        0.980
   20     99       1    0.932 0.02113        0.892        0.975
   22     79       2    0.909 0.02638        0.859        0.962
   24     59       2    0.878 0.03328        0.815        0.946
   25     49       3    0.824 0.04336        0.743        0.914
   26     39       3    0.761 0.05328        0.663        0.873
   28     35       1    0.739 0.05602        0.637        0.857
   35     30       1    0.714 0.05932        0.607        0.841
   42     25       1    0.686 0.06346        0.572        0.822
   49     20       2    0.617 0.07334        0.489        0.779
   56     15       3    0.494 0.08664        0.350        0.696
   63     10       3    0.346 0.09381        0.203        0.588
   68      5       2    0.207 0.09436        0.085        0.506

Call:
coxph(formula = Surv(Date.dpi, as.numeric(Weakness_FL)) ~ Sex,
    data = aData)

  n= 4200, number of events= 220

       coef exp(coef) se(coef)     z Pr(>|z|)
SexM 0.2623    1.2999   0.1354 1.937   0.0527 .
---
Signif. codes:  0 ,Äò***,Äô 0.001 ,Äò**,Äô 0.01 ,Äò*,Äô 0.05 ,Äò.,Äô 0.1 ,Äò ,Äô 1

     exp(coef) exp(-coef) lower .95 upper .95
SexM       1.3     0.7693    0.9969     1.695

Concordance= 0.537  (se = 0.02 )
Likelihood ratio test= 3.77  on 1 df,   p=0.05
Wald test            = 3.75  on 1 df,   p=0.05
Score (logrank) test = 3.77  on 1 df,   p=0.05

Call:
coxph(formula = Surv(Date.dpi, as.numeric(Weakness_FL)) ~ Status,
    data = aData)

  n= 4200, number of events= 220

            coef exp(coef) se(coef)      z Pr(>|z|)
StatusU -3.90928   0.02005  0.71035 -5.503 3.73e-08 ***
---
Signif. codes:  0 ,Äò***,Äô 0.001 ,Äò**,Äô 0.01 ,Äò*,Äô 0.05 ,Äò.,Äô 0.1 ,Äò ,Äô 1

        exp(coef) exp(-coef) lower .95 upper .95
StatusU   0.02005      49.86  0.004984    0.0807

Concordance= 0.653  (se = 0.007 )
Likelihood ratio test= 146  on 1 df,   p=<2e-16
```

```
Wald test            = 30.29  on 1 df,   p=4e-08
Score (logrank) test = 94.87  on 1 df,   p=<2e-16

Call:
coxph(formula = Surv(Date.dpi, as.numeric(Weakness_FL)) ~ Strain, 
    data = Z)

  n= 4176, number of events= 4176 
   (220 observations deleted due to missingness)

                     coef exp(coef) se(coef)     z Pr(>|z|)    
StrainCC078       0.22740   1.25533  0.09797 2.321 0.020275 *  
StrainCC057       0.20414   1.22647  0.08956 2.279 0.022639 *  
StrainCC012       0.50642   1.65934  0.08709 5.815 6.07e-09 ***
StrainCC041XCC012 0.17057   1.18597  0.09110 1.872 0.061161 .  
StrainCC002       0.34009   1.40508  0.09305 3.655 0.000257 ***
StrainCC025       0.34405   1.41065  0.10455 3.291 0.000999 ***
StrainCC012XCC032 0.16167   1.17547  0.10050 1.609 0.107683    
StrainCC013XCC041 0.14516   1.15622  0.10536 1.378 0.168285    
StrainCC032XCC013 0.31639   1.37217  0.09481 3.337 0.000846 ***
StrainCC015       0.53086   1.70039  0.18364 2.891 0.003843 ** 
StrainCC023       0.51687   1.67678  0.10340 4.999 5.77e-07 ***
StrainCC027       0.50971   1.66481  0.09642 5.286 1.25e-07 ***
StrainCC005       0.42832   1.53468  0.10582 4.048 5.18e-05 ***
StrainCC011       0.40966   1.50630  0.09667 4.238 2.26e-05 ***
StrainCC051       0.83373   2.30190  0.11026 7.562 3.98e-14 ***
StrainCC037       0.66866   1.95161  0.10073 6.638 3.18e-11 ***
StrainCC006       0.60122   1.82435  0.09502 6.327 2.50e-10 ***
StrainCC017       0.40918   1.50558  0.11854 3.452 0.000557 ***
---
Signif. codes:  0 ,Äò***,Äô 0.001 ,Äò**,Äô 0.01 ,Äò*,Äô 0.05 ,Äò.,Äô 0.1 ,Äò ,Äô 1

                  exp(coef) exp(-coef) lower .95 upper .95
StrainCC078           1.255     0.7966    1.0360     1.521
StrainCC057           1.226     0.8153    1.0290     1.462
StrainCC012           1.659     0.6026    1.3990     1.968
StrainCC041XCC012     1.186     0.8432    0.9920     1.418
StrainCC002           1.405     0.7117    1.1708     1.686
StrainCC025           1.411     0.7089    1.1493     1.731
StrainCC012XCC032     1.175     0.8507    0.9653     1.431
StrainCC013XCC041     1.156     0.8649    0.9405     1.421
StrainCC032XCC013     1.372     0.7288    1.1395     1.652
StrainCC015           1.700     0.5881    1.1864     2.437
StrainCC023           1.677     0.5964    1.3692     2.053
StrainCC027           1.665     0.6007    1.3781     2.011
StrainCC005           1.535     0.6516    1.2472     1.888
StrainCC011           1.506     0.6639    1.2463     1.821
StrainCC051           2.302     0.4344    1.8545     2.857
StrainCC037           1.952     0.5124    1.6020     2.378
StrainCC006           1.824     0.5481    1.5143     2.198
StrainCC017           1.506     0.6642    1.1934     1.899

Concordance= 0.577  (se = 0.005 )
Likelihood ratio test= 148.2  on 18 df,   p=<2e-16
Wald test            = 150.6  on 18 df,   p=<2e-16
Score (logrank) test = 153.1  on 18 df,   p=<2e-16
```

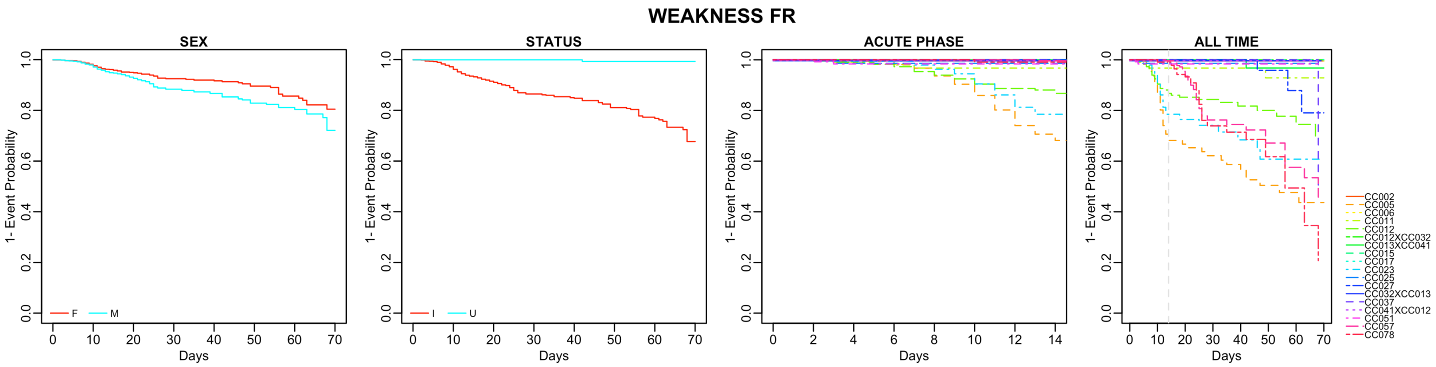


```
Call: survfit(formula = Surv(Date.dpi, as.numeric(Weakness_FR)) ~ Sex,
    data = aData)
```

Sex=F

time	n.risk	n.event	survival	std.err	lower 95% CI	upper 95% CI
0	2190	2	0.999	0.000645	0.998	1.000
2	1993	1	0.999	0.000817	0.997	1.000
3	1883	5	0.996	0.001437	0.993	0.999
5	1678	1	0.995	0.001554	0.992	0.998
6	1583	3	0.993	0.001895	0.990	0.997
7	1480	6	0.989	0.002501	0.985	0.994
8	1383	4	0.987	0.002874	0.981	0.992
9	1272	6	0.982	0.003431	0.975	0.989
10	1154	7	0.976	0.004083	0.968	0.984
11	1055	6	0.970	0.004646	0.961	0.980
12	965	6	0.964	0.005229	0.954	0.975
13	879	2	0.962	0.005443	0.952	0.973
14	799	1	0.961	0.005568	0.950	0.972
15	736	2	0.958	0.005851	0.947	0.970
16	696	2	0.956	0.006149	0.944	0.968
17	667	3	0.951	0.006604	0.938	0.964
19	617	1	0.950	0.006770	0.937	0.963
20	597	1	0.948	0.006943	0.935	0.962
21	580	1	0.947	0.007121	0.933	0.961
22	540	2	0.943	0.007514	0.928	0.958
24	498	2	0.939	0.007947	0.924	0.955
25	481	2	0.935	0.008380	0.919	0.952
26	457	4	0.927	0.009252	0.909	0.945
28	422	1	0.925	0.009488	0.907	0.944
33	366	1	0.922	0.009793	0.903	0.942
35	335	1	0.920	0.010143	0.900	0.940
40	291	1	0.917	0.010589	0.896	0.938
42	263	1	0.913	0.011108	0.892	0.935
46	224	1	0.909	0.011782	0.886	0.932
47	214	1	0.905	0.012469	0.881	0.929
49	205	2	0.896	0.013822	0.869	0.923
54	158	1	0.890	0.014852	0.862	0.920
56	137	4	0.864	0.019283	0.827	0.903
57	113	1	0.857	0.020573	0.817	0.898
61	103	1	0.848	0.021990	0.806	0.892
62	99	1	0.840	0.023378	0.795	0.887
63	94	2	0.822	0.026071	0.772	0.875
68	48	1	0.805	0.030639	0.747	0.867

Sex=M

time	n.risk	n.event	survival	std.err	lower 95% CI	upper 95% CI
0	2010	1	1.000	0.000497	0.999	1.000
2	1838	2	0.998	0.000915	0.997	1.000
3	1734	4	0.996	0.001469	0.993	0.999

```
    4   1633       2    0.995 0.001701        0.992        0.998
    5   1540       2    0.994 0.001929        0.990        0.997
    6   1454       3    0.992 0.002259        0.987        0.996
    7   1356       6    0.987 0.002873        0.982        0.993
    8   1270       6    0.982 0.003433        0.976        0.989
    9   1169       4    0.979 0.003810        0.972        0.987
   10   1058       9    0.971 0.004681        0.962        0.980
   11    964       8    0.963 0.005440        0.952        0.973
   12    883       4    0.958 0.005837        0.947        0.970
   13    809       5    0.952 0.006373        0.940        0.965
   14    730       3    0.949 0.006736        0.935        0.962
   15    669       1    0.947 0.006873        0.934        0.961
   16    631       2    0.944 0.007172        0.930        0.958
   17    607       2    0.941 0.007478        0.926        0.956
   18    587       3    0.936 0.007939        0.921        0.952
   19    558       3    0.931 0.008411        0.915        0.948
   20    539       3    0.926 0.008880        0.909        0.944
   21    525       3    0.921 0.009340        0.903        0.939
   22    487       2    0.917 0.009677        0.898        0.936
   23    473       2    0.913 0.010017        0.894        0.933
   24    452       4    0.905 0.010712        0.884        0.926
   25    438       6    0.893 0.011700        0.870        0.916
   26    414       2    0.888 0.012034        0.865        0.912
   28    384       2    0.884 0.012408        0.860        0.908
   32    349       2    0.879 0.012843        0.854        0.904
   35    313       2    0.873 0.013361        0.847        0.900
   39    286       2    0.867 0.013947        0.840        0.895
   42    252       4    0.853 0.015329        0.824        0.884
   46    219       2    0.845 0.016149        0.814        0.878
   47    208       1    0.841 0.016574        0.809        0.874
   49    199       3    0.829 0.017869        0.794        0.864
   53    163       1    0.823 0.018468        0.788        0.860
   56    134       2    0.811 0.020134        0.773        0.852
   60    108       1    0.804 0.021302        0.763        0.847
   63     90       2    0.786 0.024285        0.740        0.835
   67     54       1    0.771 0.027856        0.719        0.828
   68     46       3    0.721 0.038294        0.650        0.800

Call: survfit(formula = Surv(Date.dpi, as.numeric(Weakness_FR)) ~ Status,
    data = aData)

                Status=I
 time n.risk n.event survival  std.err lower 95% CI upper 95% CI
    0   2809       2    0.999 0.000503        0.998        1.000
    2   2560       3    0.998 0.000842        0.996        1.000
    3   2420       9    0.994 0.001493        0.991        0.997
    4   2287       2    0.994 0.001613        0.990        0.997
    5   2165       3    0.992 0.001796        0.989        0.996
    6   2054       6    0.989 0.002146        0.985        0.993
    7   1926      12    0.983 0.002774        0.978        0.989
    8   1811      10    0.978 0.003246        0.971        0.984
    9   1669      10    0.972 0.003718        0.965        0.979
   10   1511      16    0.962 0.004481        0.953        0.970
   11   1380      14    0.952 0.005138        0.942        0.962
   12   1267      10    0.944 0.005620        0.933        0.955
   13   1165       7    0.939 0.005982        0.927        0.950
   14   1057       4    0.935 0.006217        0.923        0.947
   15    973       3    0.932 0.006417        0.920        0.945
   16    913       4    0.928 0.006706        0.915        0.941
   17    879       5    0.923 0.007071        0.909        0.937
```

```
   18    849        3    0.920 0.007292        0.905        0.934
   19    802        4    0.915 0.007608        0.900        0.930
   20    775        4    0.910 0.007927        0.895        0.926
   21    756        4    0.905 0.008242        0.889        0.922
   22    700        4    0.900 0.008591        0.884        0.917
   23    681        2    0.898 0.008767        0.881        0.915
   24    654        6    0.889 0.009309        0.871        0.908
   25    635        8    0.878 0.009999        0.859        0.898
   26    598        6    0.869 0.010526        0.849        0.890
   28    551        3    0.865 0.010818        0.844        0.886
   32    512        2    0.861 0.011036        0.840        0.883
   33    493        1    0.859 0.011151        0.838        0.882
   35    455        3    0.854 0.011547        0.831        0.877
   39    420        2    0.850 0.011845        0.827        0.873
   40    401        1    0.848 0.012003        0.824        0.871
   42    364        4    0.838 0.012743        0.814        0.864
   46    318        3    0.830 0.013416        0.805        0.857
   47    299        2    0.825 0.013889        0.798        0.853
   49    288        5    0.811 0.015052        0.782        0.841
   53    235        1    0.807 0.015378        0.777        0.838
   54    217        1    0.803 0.015751        0.773        0.835
   56    189        6    0.778 0.018372        0.743        0.815
   57    158        1    0.773 0.018904        0.737        0.811
   60    154        1    0.768 0.019436        0.731        0.807
   61    139        1    0.762 0.020066        0.724        0.803
   62    134        1    0.757 0.020707        0.717        0.798
   63    129        4    0.733 0.023151        0.689        0.780
   67     76        1    0.724 0.024775        0.677        0.774
   68     62        4    0.677 0.032355        0.616        0.743

                Status=U
 time n.risk n.event survival  std.err lower 95% CI upper 95% CI
    0   1391       1    0.999 0.000719        0.998            1
   42    151       1    0.993 0.006634        0.980            1

Call: survfit(formula = Surv(Date.dpi, as.numeric(Weakness_FR)) ~ Strain,
    data = aData)

                Strain=CC002
       time        n.risk      n.event     survival      std.err lower 95% CI upper
95% CI
    4.00e+00     2.26e+02     1.00e+00     9.96e-01     4.41e-03     9.87e-01
1.00e+00

                Strain=CC005
 time n.risk n.event survival std.err lower 95% CI upper 95% CI
    3    186       1    0.995 0.00536        0.984        1.000
    6    150       2    0.981 0.01071        0.961        1.000
    7    138       2    0.967 0.01453        0.939        0.996
    8    126       4    0.936 0.02064        0.897        0.978
    9    114       4    0.904 0.02564        0.855        0.955
   10    102       5    0.859 0.03110        0.800        0.922
   11     90       6    0.802 0.03679        0.733        0.877
   12     78       6    0.740 0.04170        0.663        0.827
   13     66       3    0.707 0.04410        0.625        0.799
   14     56       2    0.681 0.04599        0.597        0.778
   19     48       1    0.667 0.04717        0.581        0.766
   21     46       1    0.653 0.04833        0.565        0.755
   26     42       1    0.637 0.04961        0.547        0.742
   28     40       1    0.621 0.05086        0.529        0.729
```

```
   33     36       1    0.604 0.05230        0.510        0.716
   35     34       1    0.586 0.05369        0.490        0.701
   40     30       1    0.567 0.05534        0.468        0.686
   42     28       2    0.526 0.05832        0.423        0.654
   47     24       1    0.504 0.05987        0.400        0.636
   54     18       1    0.476 0.06276        0.368        0.617
   61     12       1    0.437 0.06895        0.320        0.595

                Strain=CC006 
        time        n.risk      n.event     survival      std.err lower 95% CI upper 
95% CI 
    0.00e+00     2.62e+02     1.00e+00     9.96e-01     3.81e-03     9.89e-01 
1.00e+00 

                Strain=CC011 
 time n.risk n.event survival std.err lower 95% CI upper 95% CI
    3    216       2    0.991 0.00652        0.978        1.000
    5    187       1    0.985 0.00836        0.969        1.000
    6    173       1    0.980 0.01007        0.960        1.000
    7    159       2    0.967 0.01318        0.942        0.994
   49     25       1    0.929 0.03997        0.854        1.000

                Strain=CC012 
 time n.risk n.event survival std.err lower 95% CI upper 95% CI
    2    420       2    0.995 0.00336        0.989        1.000
    3    396       2    0.990 0.00487        0.981        1.000
    4    372       1    0.988 0.00554        0.977        0.998
    5    348       2    0.982 0.00681        0.969        0.995
    6    324       3    0.973 0.00853        0.956        0.990
    7    300       6    0.953 0.01148        0.931        0.976
    8    276       4    0.940 0.01323        0.914        0.966
    9    252       4    0.925 0.01497        0.896        0.954
   10    228       5    0.904 0.01717        0.871        0.939
   11    204       4    0.887 0.01899        0.850        0.925
   13    156       1    0.881 0.01970        0.843        0.920
   14    132       2    0.868 0.02154        0.826        0.911
   15    120       1    0.860 0.02254        0.817        0.906
   18    108       1    0.852 0.02370        0.807        0.900
   25     96       1    0.843 0.02506        0.796        0.894
   32     71       1    0.832 0.02738        0.780        0.887
   39     59       1    0.818 0.03033        0.760        0.879
   46     47       1    0.800 0.03431        0.736        0.870
   53     35       1    0.777 0.04023        0.702        0.860
   60     24       1    0.745 0.04992        0.653        0.849
   67     12       1    0.683 0.07500        0.551        0.847

                Strain=CC012XCC032 
     time n.risk n.event survival std.err lower 95% CI upper 95% CI

                Strain=CC013XCC041 
        time        n.risk      n.event     survival      std.err lower 95% CI upper 
95% CI 
     42.0000      31.0000       1.0000       0.9677       0.0317       0.9075 
1.0000 

                Strain=CC015 
     time n.risk n.event survival std.err lower 95% CI upper 95% CI

                Strain=CC017 
     time n.risk n.event survival std.err lower 95% CI upper 95% CI
```

```
                Strain=CC023 
 time n.risk n.event survival std.err lower 95% CI upper 95% CI
    3    177       1    0.994 0.00563        0.983        1.000
    7    131       2    0.979 0.01201        0.956        1.000
    8    119       2    0.963 0.01651        0.931        0.996
    9    107       2    0.945 0.02053        0.905        0.986
   10     95       4    0.905 0.02767        0.852        0.961
   11     83       4    0.861 0.03385        0.797        0.930
   12     71       4    0.813 0.03970        0.739        0.894
   13     59       2    0.785 0.04287        0.706        0.874
   18     38       1    0.765 0.04646        0.679        0.861
   25     33       1    0.741 0.05050        0.649        0.847
   32     28       1    0.715 0.05520        0.615        0.832
   39     23       1    0.684 0.06093        0.574        0.814
   46     18       1    0.646 0.06837        0.525        0.795
   47     17       1    0.608 0.07416        0.479        0.772

                Strain=CC025 
        time       n.risk      n.event     survival      std.err lower 95% CI upper 
95% CI 
    3.00e+00     1.44e+02     2.00e+00     9.86e-01     9.75e-03     9.67e-01 
1.00e+00 

                Strain=CC027 
 time n.risk n.event survival std.err lower 95% CI upper 95% CI
   46     24       1    0.958  0.0408        0.882            1
   57     12       1    0.878  0.0851        0.727            1
   62     10       1    0.791  0.1132        0.597            1

                Strain=CC032XCC013 
        time       n.risk      n.event     survival      std.err lower 95% CI upper 
95% CI 
    0.00e+00     2.62e+02     1.00e+00     9.96e-01     3.81e-03     9.89e-01 
1.00e+00 

                Strain=CC037 
        time       n.risk      n.event     survival      std.err lower 95% CI upper 
95% CI 
      68.000        2.000        1.000        0.500        0.354        0.125 
1.000 

                Strain=CC041XCC012 
        time       n.risk      n.event     survival      std.err lower 95% CI upper 
95% CI 
    0.00e+00     3.25e+02     1.00e+00     9.97e-01     3.07e-03     9.91e-01 
1.00e+00 

                Strain=CC051 
 time n.risk n.event survival std.err lower 95% CI upper 95% CI
    2    128       1    0.992 0.00778        0.977            1
    3    118       1    0.984 0.01139        0.962            1

                Strain=CC057 
 time n.risk n.event survival std.err lower 95% CI upper 95% CI
   13    264       1    0.996 0.00378        0.989        1.000
   16    216       2    0.987 0.00750        0.972        1.000
   17    200       2    0.977 0.01016        0.957        0.997
   18    184       1    0.972 0.01141        0.950        0.994
   19    168       3    0.954 0.01497        0.926        0.984
```

```
   20    152       3    0.936 0.01820        0.901        0.972
   21    136       3    0.915 0.02135        0.874        0.958
   22    120       2    0.900 0.02356        0.855        0.947
   23    104       2    0.882 0.02609        0.833        0.935
   24     88       4    0.842 0.03169        0.782        0.907
   25     72       3    0.807 0.03627        0.739        0.882
   26     56       2    0.778 0.04030        0.703        0.862
   28     49       1    0.763 0.04249        0.684        0.851
   35     42       1    0.744 0.04519        0.661        0.838
   42     35       1    0.723 0.04865        0.634        0.825
   49     28       2    0.671 0.05727        0.568        0.794
   56     21       3    0.576 0.07098        0.452        0.733
   63     14       1    0.534 0.07690        0.403        0.709
   68      7       1    0.458 0.09664        0.303        0.693

                Strain=CC078
 time n.risk n.event survival std.err lower 95% CI upper 95% CI
   10    217       2    0.991 0.00649        0.978        1.000
   15    154       2    0.978 0.01108        0.956        1.000
   16    142       2    0.964 0.01459        0.936        0.993
   17    130       3    0.942 0.01909        0.905        0.980
   20     99       1    0.932 0.02113        0.892        0.975
   22     79       2    0.909 0.02638        0.859        0.962
   24     59       2    0.878 0.03328        0.815        0.946
   25     49       3    0.824 0.04336        0.743        0.914
   26     39       3    0.761 0.05328        0.663        0.873
   28     35       1    0.739 0.05602        0.637        0.857
   35     30       1    0.714 0.05932        0.607        0.841
   42     25       1    0.686 0.06346        0.572        0.822
   49     20       2    0.617 0.07334        0.489        0.779
   56     15       3    0.494 0.08664        0.350        0.696
   63     10       3    0.346 0.09381        0.203        0.588
   68      5       2    0.207 0.09436        0.085        0.506

Call:
coxph(formula = Surv(Date.dpi, as.numeric(Weakness_FR)) ~ Sex,
    data = aData)

  n= 4200, number of events= 210

       coef exp(coef) se(coef)     z Pr(>|z|)
SexM 0.3680    1.4448   0.1395 2.639  0.00832 **
---
Signif. codes:  0 ‚Äò***‚Äô 0.001 ‚Äò**‚Äô 0.01 ‚Äò*‚Äô 0.05 ‚Äò.‚Äô 0.1 ‚Äò ‚Äô 1

     exp(coef) exp(-coef) lower .95 upper .95
SexM     1.445     0.6921     1.099     1.899

Concordance= 0.538  (se = 0.02 )
Likelihood ratio test= 7.05  on 1 df,   p=0.008
Wald test            = 6.96  on 1 df,   p=0.008
Score (logrank) test = 7.04  on 1 df,   p=0.008

Call:
coxph(formula = Surv(Date.dpi, as.numeric(Weakness_FR)) ~ Status,
    data = aData)

  n= 4200, number of events= 210

            coef exp(coef) se(coef)      z Pr(>|z|)
```

```
StatusU -3.86372   0.02099  0.71051 -5.438 5.39e-08 ***
---
Signif. codes:  0 ‚Äò***‚Äô 0.001 ‚Äò**‚Äô 0.01 ‚Äò*‚Äô 0.05 ‚Äò.‚Äô 0.1 ‚Äò ‚Äô 1

        exp(coef) exp(-coef) lower .95 upper .95
StatusU   0.02099      47.64  0.005215   0.08449

Concordance= 0.653  (se = 0.007 )
Likelihood ratio test= 138.9  on 1 df,   p=<2e-16
Wald test            = 29.57  on 1 df,   p=5e-08
Score (logrank) test = 90.43  on 1 df,   p=<2e-16

Call:
coxph(formula = Surv(Date.dpi, as.numeric(Weakness_FR)) ~ Strain,
    data = Z)

  n= 4186, number of events= 4186
   (210 observations deleted due to missingness)

                    coef exp(coef) se(coef)     z Pr(>|z|)
StrainCC078       0.22763   1.25562  0.09797 2.324 0.020148 *
StrainCC057       0.20425   1.22660  0.08956 2.281 0.022567 *
StrainCC012       0.49637   1.64275  0.08668 5.727 1.02e-08 ***
StrainCC041XCC012 0.17366   1.18965  0.09094 1.910 0.056181 .
StrainCC002       0.33748   1.40141  0.09311 3.624 0.000290 ***
StrainCC025       0.33930   1.40396  0.10471 3.240 0.001194 **
StrainCC012XCC032 0.16131   1.17505  0.10050 1.605 0.108481
StrainCC013XCC041 0.14943   1.16118  0.10554 1.416 0.156805
StrainCC032XCC013 0.31225   1.36650  0.09489 3.291 0.000999 ***
StrainCC015       0.53097   1.70059  0.18364 2.891 0.003836 **
StrainCC023       0.52644   1.69289  0.10342 5.090 3.57e-07 ***
StrainCC027       0.51249   1.66945  0.09641 5.316 1.06e-07 ***
StrainCC005       0.42553   1.53039  0.10617 4.008 6.12e-05 ***
StrainCC011       0.40973   1.50641  0.09667 4.238 2.25e-05 ***
StrainCC051       0.83453   2.30374  0.11026 7.569 3.76e-14 ***
StrainCC037       0.66904   1.95237  0.10073 6.642 3.10e-11 ***
StrainCC006       0.60516   1.83155  0.09494 6.374 1.84e-10 ***
StrainCC017       0.40936   1.50586  0.11854 3.453 0.000554 ***
---
Signif. codes:  0 ‚Äò***‚Äô 0.001 ‚Äò**‚Äô 0.01 ‚Äò*‚Äô 0.05 ‚Äò.‚Äô 0.1 ‚Äò ‚Äô 1

                  exp(coef) exp(-coef) lower .95 upper .95
StrainCC078           1.256     0.7964    1.0363     1.521
StrainCC057           1.227     0.8153    1.0291     1.462
StrainCC012           1.643     0.6087    1.3861     1.947
StrainCC041XCC012     1.190     0.8406    0.9954     1.422
StrainCC002           1.401     0.7136    1.1676     1.682
StrainCC025           1.404     0.7123    1.1435     1.724
StrainCC012XCC032     1.175     0.8510    0.9650     1.431
StrainCC013XCC041     1.161     0.8612    0.9442     1.428
StrainCC032XCC013     1.366     0.7318    1.1346     1.646
StrainCC015           1.701     0.5880    1.1865     2.437
StrainCC023           1.693     0.5907    1.3823     2.073
StrainCC027           1.669     0.5990    1.3820     2.017
StrainCC005           1.530     0.6534    1.2429     1.884
StrainCC011           1.506     0.6638    1.2464     1.821
StrainCC051           2.304     0.4341    1.8560     2.859
StrainCC037           1.952     0.5122    1.6026     2.379
StrainCC006           1.832     0.5460    1.5205     2.206
StrainCC017           1.506     0.6641    1.1937     1.900
```

```
Concordance= 0.577  (se = 0.005 )
Likelihood ratio test= 148.1  on 18 df,   p=<2e-16
Wald test            = 150.6  on 18 df,   p=<2e-16
Score (logrank) test = 153.1  on 18 df,   p=<2e-16
```

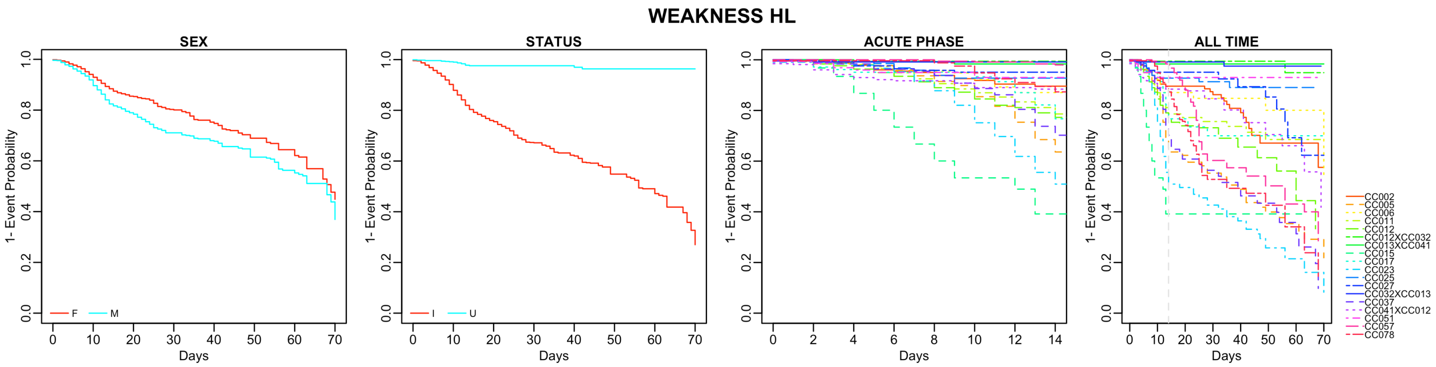


```
Call: survfit(formula = Surv(Date.dpi, as.numeric(Weakness_HL)) ~ Sex,
    data = aData)
```

Sex=F

time	n.risk	n.event	survival	std.err	lower 95% CI	upper 95% CI
0	2190	4	0.998	0.000912	0.996	1.000
1	2084	3	0.997	0.001232	0.994	0.999
2	1993	6	0.994	0.001733	0.990	0.997
3	1883	8	0.990	0.002280	0.985	0.994
4	1778	13	0.982	0.003020	0.976	0.988
5	1678	9	0.977	0.003477	0.970	0.984
6	1583	10	0.971	0.003965	0.963	0.979
7	1480	12	0.963	0.004538	0.954	0.972
8	1383	17	0.951	0.005313	0.941	0.962
9	1272	14	0.941	0.005946	0.929	0.952
10	1154	14	0.929	0.006610	0.916	0.942
11	1055	13	0.918	0.007251	0.904	0.932
12	965	13	0.905	0.007923	0.890	0.921
13	879	12	0.893	0.008581	0.876	0.910
14	799	8	0.884	0.009059	0.867	0.902
15	736	8	0.875	0.009576	0.856	0.893
16	696	5	0.868	0.009911	0.849	0.888
17	667	3	0.864	0.010120	0.845	0.884
18	645	3	0.860	0.010335	0.840	0.881
19	617	4	0.855	0.010638	0.834	0.876
20	597	1	0.853	0.010716	0.833	0.875
21	580	3	0.849	0.010959	0.828	0.871
22	540	2	0.846	0.011142	0.824	0.868
23	523	1	0.844	0.011237	0.822	0.866
24	498	3	0.839	0.011547	0.817	0.862
25	481	7	0.827	0.012266	0.803	0.851
26	457	7	0.814	0.012979	0.789	0.840
27	441	2	0.810	0.013180	0.785	0.837
28	422	3	0.805	0.013500	0.779	0.832
29	395	1	0.803	0.013618	0.776	0.830
30	387	1	0.801	0.013740	0.774	0.828
32	376	3	0.794	0.014117	0.767	0.822
33	366	2	0.790	0.014369	0.762	0.819
34	357	6	0.777	0.015115	0.748	0.807
35	335	6	0.763	0.015876	0.732	0.794
36	310	1	0.760	0.016014	0.729	0.792
39	301	3	0.753	0.016441	0.721	0.786
40	291	2	0.747	0.016730	0.715	0.781
41	282	3	0.740	0.017170	0.707	0.774
42	263	5	0.725	0.017958	0.691	0.762
43	234	1	0.722	0.018147	0.688	0.759
44	229	1	0.719	0.018340	0.684	0.756
46	224	3	0.710	0.018919	0.673	0.748
47	214	2	0.703	0.019314	0.666	0.742

49	205	4	0.689	0.020118	0.651	0.730
53	167	4	0.673	0.021262	0.632	0.716
54	158	1	0.668	0.021550	0.628	0.712
56	137	5	0.644	0.023363	0.600	0.692
60	111	4	0.621	0.025239	0.573	0.672
61	103	1	0.615	0.025704	0.566	0.667
63	94	7	0.569	0.029036	0.515	0.629
67	56	4	0.528	0.033324	0.467	0.598
68	48	2	0.506	0.035386	0.442	0.581
69	34	2	0.477	0.039073	0.406	0.560
70	17	1	0.449	0.045740	0.367	0.548

Sex=M

time	n.risk	n.event	survival	std.err	lower 95% CI	upper 95% CI
0	2010	8	0.996	0.00140	0.993	0.999
1	1919	2	0.995	0.00158	0.992	0.998
2	1838	14	0.987	0.00256	0.982	0.992
3	1734	16	0.978	0.00340	0.972	0.985
4	1633	10	0.972	0.00387	0.965	0.980
5	1540	16	0.962	0.00458	0.953	0.971
6	1454	13	0.954	0.00512	0.944	0.964
7	1356	14	0.944	0.00571	0.933	0.955
8	1270	13	0.934	0.00625	0.922	0.946
9	1169	19	0.919	0.00705	0.905	0.933
10	1058	26	0.896	0.00815	0.880	0.912
11	964	18	0.880	0.00890	0.862	0.897
12	883	17	0.863	0.00963	0.844	0.882
13	809	21	0.840	0.01055	0.820	0.861
14	730	14	0.824	0.01119	0.803	0.846
15	669	6	0.817	0.01149	0.795	0.840
16	631	4	0.812	0.01170	0.789	0.835
17	607	5	0.805	0.01198	0.782	0.829
18	587	7	0.795	0.01238	0.771	0.820
19	558	4	0.790	0.01261	0.765	0.815
20	539	4	0.784	0.01286	0.759	0.809
21	525	8	0.772	0.01333	0.746	0.798
22	487	5	0.764	0.01366	0.738	0.791
23	473	6	0.754	0.01405	0.727	0.782
24	452	6	0.744	0.01444	0.716	0.773
25	438	8	0.731	0.01496	0.702	0.760
26	414	3	0.725	0.01516	0.696	0.756
27	398	3	0.720	0.01537	0.690	0.751
28	384	5	0.710	0.01573	0.680	0.742
32	349	4	0.702	0.01607	0.672	0.735
33	338	1	0.700	0.01616	0.669	0.733
34	330	1	0.698	0.01624	0.667	0.731
35	313	4	0.689	0.01664	0.657	0.723
36	292	1	0.687	0.01675	0.655	0.720
39	286	3	0.680	0.01708	0.647	0.714
40	275	1	0.677	0.01720	0.644	0.712
41	267	3	0.670	0.01756	0.636	0.705
42	252	5	0.656	0.01818	0.622	0.693
46	219	2	0.650	0.01851	0.615	0.688
47	208	1	0.647	0.01868	0.612	0.685
49	199	10	0.615	0.02038	0.576	0.656
53	163	1	0.611	0.02060	0.572	0.653
54	153	1	0.607	0.02085	0.567	0.649
55	148	3	0.595	0.02160	0.554	0.638
56	134	6	0.568	0.02321	0.524	0.615
57	110	1	0.563	0.02356	0.518	0.611

```
   60    108       2    0.552 0.02425        0.507        0.602
   61     99       1    0.547 0.02464        0.501        0.597
   62     94       1    0.541 0.02505        0.494        0.592
   63     90       5    0.511 0.02703        0.461        0.567
   68     46       4    0.466 0.03255        0.407        0.535
   69     33       2    0.438 0.03620        0.373        0.515
   70     19       3    0.369 0.04768        0.286        0.475

Call: survfit(formula = Surv(Date.dpi, as.numeric(Weakness_HL)) ~ Status, 
    data = aData)

                Status=I 
 time n.risk n.event survival std.err lower 95% CI upper 95% CI
    0   2809      11    0.996 0.00118        0.994        0.998
    1   2667       3    0.995 0.00134        0.992        0.998
    2   2560      20    0.987 0.00218        0.983        0.991
    3   2420      22    0.978 0.00288        0.973        0.984
    4   2287      23    0.968 0.00351        0.962        0.975
    5   2165      25    0.957 0.00412        0.949        0.965
    6   2054      23    0.946 0.00464        0.937        0.956
    7   1926      24    0.935 0.00517        0.925        0.945
    8   1811      29    0.920 0.00579        0.908        0.931
    9   1669      32    0.902 0.00646        0.890        0.915
   10   1511      39    0.879 0.00729        0.865        0.893
   11   1380      29    0.860 0.00790        0.845        0.876
   12   1267      28    0.841 0.00851        0.825        0.858
   13   1165      30    0.820 0.00916        0.802        0.838
   14   1057      21    0.803 0.00964        0.785        0.822
   15    973      14    0.792 0.00999        0.772        0.812
   16    913       9    0.784 0.01022        0.764        0.804
   17    879       8    0.777 0.01044        0.757        0.798
   18    849      10    0.768 0.01071        0.747        0.789
   19    802       8    0.760 0.01094        0.739        0.782
   20    775       5    0.755 0.01108        0.734        0.777
   21    756      11    0.744 0.01141        0.722        0.767
   22    700       7    0.737 0.01163        0.714        0.760
   23    681       7    0.729 0.01186        0.706        0.753
   24    654       9    0.719 0.01216        0.696        0.743
   25    635      15    0.702 0.01264        0.678        0.727
   26    598      10    0.690 0.01296        0.665        0.716
   27    575       5    0.684 0.01312        0.659        0.711
   28    551       8    0.674 0.01340        0.649        0.701
   29    525       1    0.673 0.01343        0.647        0.700
   30    519       1    0.672 0.01347        0.646        0.699
   32    512       7    0.663 0.01372        0.636        0.690
   33    493       3    0.659 0.01384        0.632        0.686
   34    483       7    0.649 0.01410        0.622        0.677
   35    455      10    0.635 0.01449        0.607        0.664
   36    429       2    0.632 0.01458        0.604        0.661
   39    420       6    0.623 0.01483        0.594        0.653
   40    401       2    0.620 0.01491        0.591        0.650
   41    391       6    0.610 0.01518        0.581        0.641
   42    364       9    0.595 0.01562        0.565        0.627
   43    328       1    0.593 0.01567        0.563        0.625
   44    323       1    0.591 0.01573        0.561        0.623
   46    318       5    0.582 0.01603        0.552        0.614
   47    299       3    0.576 0.01622        0.545        0.609
   49    288      14    0.548 0.01707        0.516        0.583
   53    235       5    0.537 0.01749        0.503        0.572
   54    217       2    0.532 0.01767        0.498        0.568
```

```
   55    212       3    0.524 0.01795        0.490        0.561
   56    189      11    0.494 0.01912        0.458        0.533
   57    158       1    0.491 0.01925        0.454        0.530
   60    154       6    0.471 0.02002        0.434        0.512
   61    139       2    0.465 0.02030        0.427        0.506
   62    134       1    0.461 0.02044        0.423        0.503
   63    129      12    0.418 0.02197        0.377        0.464
   67     76       4    0.396 0.02341        0.353        0.445
   68     62       6    0.358 0.02586        0.311        0.412
   69     46       4    0.327 0.02790        0.276        0.386
   70     23       4    0.270 0.03462        0.210        0.347

                Status=U 
 time n.risk n.event survival  std.err lower 95% CI upper 95% CI
    0   1391       1    0.999 0.000719        0.998        1.000
    1   1336       2    0.998 0.001278        0.995        1.000
    3   1197       2    0.996 0.001736        0.993        1.000
    7    910       2    0.994 0.002322        0.989        0.998
    8    842       1    0.993 0.002602        0.988        0.998
    9    772       1    0.991 0.002899        0.986        0.997
   10    701       1    0.990 0.003222        0.984        0.996
   11    639       2    0.987 0.003886        0.979        0.995
   12    581       2    0.984 0.004555        0.975        0.993
   13    523       3    0.978 0.005573        0.967        0.989
   14    472       1    0.976 0.005934        0.964        0.988
   40    165       1    0.970 0.008340        0.954        0.986
   42    151       1    0.964 0.010470        0.943        0.984

Call: survfit(formula = Surv(Date.dpi, as.numeric(Weakness_HL)) ~ Strain, 
    data = aData)

                Strain=CC002 
 time n.risk n.event survival std.err lower 95% CI upper 95% CI
    3    242       3    0.988 0.00711        0.974        1.000
    4    226       2    0.979 0.00936        0.961        0.997
    5    210       4    0.960 0.01302        0.935        0.986
    6    194       1    0.955 0.01386        0.928        0.983
    7    178       1    0.950 0.01479        0.921        0.979
    8    162       2    0.938 0.01677        0.906        0.972
    9    148       2    0.925 0.01879        0.889        0.963
   10    134       1    0.919 0.01987        0.880        0.958
   11    126       2    0.904 0.02207        0.862        0.948
   13    110       1    0.896 0.02335        0.851        0.943
   27     87       1    0.885 0.02525        0.837        0.936
   29     78       1    0.874 0.02736        0.822        0.929
   30     74       1    0.862 0.02943        0.807        0.922
   33     70       1    0.850 0.03148        0.790        0.914
   34     66       1    0.837 0.03354        0.774        0.906
   35     62       1    0.824 0.03561        0.757        0.896
   36     54       1    0.808 0.03808        0.737        0.887
   40     47       1    0.791 0.04097        0.715        0.876
   41     43       1    0.773 0.04395        0.691        0.864
   42     39       1    0.753 0.04708        0.666        0.851
   43     31       1    0.729 0.05145        0.635        0.837
   44     27       1    0.702 0.05618        0.600        0.821
   47     23       1    0.671 0.06146        0.561        0.803
   68      7       1    0.575 0.10323        0.405        0.818

                Strain=CC005 
 time n.risk n.event survival std.err lower 95% CI upper 95% CI
```

time	n.risk	n.event	survival	std.err	lower 95% CI	upper 95% CI
3	186	1	0.995	0.00536	0.984	1.000
4	174	2	0.983	0.00963	0.965	1.000
5	162	1	0.977	0.01132	0.955	1.000
6	150	3	0.958	0.01574	0.927	0.989
7	138	3	0.937	0.01945	0.899	0.976
8	126	3	0.914	0.02286	0.871	0.960
9	114	2	0.898	0.02512	0.851	0.949
10	102	5	0.854	0.03065	0.796	0.917
11	90	4	0.816	0.03467	0.751	0.887
12	78	6	0.754	0.04039	0.678	0.837
13	66	6	0.685	0.04538	0.602	0.780
14	56	4	0.636	0.04828	0.548	0.738
19	48	1	0.623	0.04906	0.534	0.727
21	46	2	0.596	0.05053	0.505	0.704
26	42	1	0.582	0.05128	0.489	0.691
28	40	2	0.553	0.05268	0.458	0.666
33	36	1	0.537	0.05340	0.442	0.653
35	34	2	0.506	0.05474	0.409	0.625
40	30	1	0.489	0.05545	0.391	0.610
42	28	3	0.436	0.05716	0.338	0.564
47	24	1	0.418	0.05760	0.319	0.548
49	22	1	0.399	0.05803	0.300	0.531
54	18	1	0.377	0.05889	0.278	0.512
56	16	1	0.353	0.05974	0.254	0.492
61	12	1	0.324	0.06160	0.223	0.470
63	10	1	0.292	0.06339	0.190	0.446
70	4	1	0.219	0.07903	0.108	0.444

Strain=CC006

time	n.risk	n.event	survival	std.err	lower 95% CI	upper 95% CI
1	250	2	0.992	0.00563	0.981	1.000
2	234	2	0.984	0.00818	0.968	1.000
4	202	1	0.979	0.00947	0.960	0.997
5	186	1	0.973	0.01079	0.952	0.995
6	170	2	0.962	0.01336	0.936	0.988
7	154	1	0.956	0.01466	0.927	0.985
9	123	1	0.948	0.01647	0.916	0.981
10	109	3	0.922	0.02184	0.880	0.966
11	95	3	0.893	0.02685	0.842	0.947
12	81	2	0.871	0.03038	0.813	0.932
23	38	1	0.848	0.03723	0.778	0.924
49	18	1	0.801	0.05772	0.695	0.922
70	3	1	0.534	0.22129	0.237	1.000

Strain=CC011

time	n.risk	n.event	survival	std.err	lower 95% CI	upper 95% CI
3	216	3	0.986	0.00796	0.971	1.000
4	202	1	0.981	0.00930	0.963	1.000
5	187	3	0.965	0.01285	0.941	0.991
6	173	3	0.949	0.01585	0.918	0.980
7	159	4	0.925	0.01943	0.888	0.964
8	145	3	0.906	0.02195	0.864	0.950
9	131	3	0.885	0.02449	0.838	0.934
10	117	2	0.870	0.02631	0.820	0.923
11	103	2	0.853	0.02838	0.799	0.910
12	89	2	0.834	0.03081	0.776	0.896
13	75	2	0.812	0.03376	0.748	0.881
14	64	2	0.786	0.03716	0.717	0.863
19	56	1	0.772	0.03906	0.699	0.853
26	49	1	0.756	0.04132	0.680	0.842

time	n.risk	n.event	survival	std.err	lower 95% CI	upper 95% CI
35	39	1	0.737	0.04458	0.655	0.830
42	32	1	0.714	0.04878	0.625	0.816
49	25	1	0.685	0.05455	0.586	0.801

Strain=CC012

time	n.risk	n.event	survival	std.err	lower 95% CI	upper 95% CI
0	468	2	0.996	0.00302	0.990	1.000
2	420	5	0.984	0.00605	0.972	0.996
3	396	3	0.976	0.00738	0.962	0.991
4	372	5	0.963	0.00933	0.945	0.982
5	348	5	0.949	0.01106	0.928	0.971
6	324	5	0.935	0.01268	0.910	0.960
7	300	7	0.913	0.01482	0.884	0.943
8	276	7	0.890	0.01684	0.857	0.923
9	252	5	0.872	0.01826	0.837	0.909
10	228	7	0.845	0.02031	0.807	0.886
11	204	6	0.821	0.02210	0.778	0.865
12	180	2	0.811	0.02278	0.768	0.857
13	156	4	0.791	0.02446	0.744	0.840
14	132	3	0.773	0.02601	0.723	0.825
15	120	3	0.753	0.02765	0.701	0.810
18	108	2	0.739	0.02884	0.685	0.798
25	96	1	0.732	0.02955	0.676	0.792
32	71	4	0.690	0.03433	0.626	0.761
39	59	3	0.655	0.03810	0.585	0.734
46	47	3	0.614	0.04264	0.535	0.703
53	35	3	0.561	0.04861	0.473	0.665
60	24	5	0.444	0.06036	0.340	0.580
67	12	3	0.333	0.07163	0.218	0.508

Strain=CC012XCC032

time	n.risk	n.event	survival	std.err	lower 95% CI	upper 95% CI
3	177	1	0.994	0.00563	0.983	1
56	22	1	0.949	0.04448	0.866	1

Strain=CC013XCC041

time	n.risk	n.event	survival	std.err	lower 95% CI	upper 95% CI
4	140	1	0.993	0.00712	0.979	1
9	107	1	0.984	0.01162	0.961	1

Strain=CC015

time	n.risk	n.event	survival	std.err	lower 95% CI	upper 95% CI
2	31	1	0.968	0.0317	0.908	1.000
3	29	1	0.934	0.0449	0.850	1.000
4	28	2	0.868	0.0617	0.755	0.997
5	26	2	0.801	0.0728	0.670	0.957
6	24	2	0.734	0.0806	0.592	0.910
7	22	2	0.667	0.0860	0.518	0.859
8	20	2	0.601	0.0894	0.449	0.804
9	18	2	0.534	0.0911	0.382	0.746
12	12	1	0.489	0.0937	0.336	0.712
13	10	2	0.392	0.0972	0.241	0.637

Strain=CC017

time	n.risk	n.event	survival	std.err	lower 95% CI	upper 95% CI
2	105	3	0.971	0.0163	0.940	1.000
3	97	1	0.961	0.0189	0.925	0.999
4	90	1	0.951	0.0215	0.909	0.994
10	54	1	0.933	0.0274	0.881	0.988
11	48	1	0.914	0.0330	0.851	0.981

time	n.risk	n.event	survival	std.err	lower 95% CI	upper 95% CI
12	42	2	0.870	0.0435	0.789	0.960
13	36	2	0.822	0.0528	0.725	0.932
14	30	2	0.767	0.0619	0.655	0.898
21	24	1	0.735	0.0671	0.615	0.879
28	21	1	0.700	0.0724	0.572	0.857

Strain=CC023

time	n.risk	n.event	survival	std.err	lower 95% CI	upper 95% CI
0	209	2	0.9904	0.00673	0.9773	1.000
3	177	1	0.9848	0.00872	0.9679	1.000
4	167	1	0.9789	0.01047	0.9586	1.000
5	155	2	0.9663	0.01362	0.9400	0.993
6	143	2	0.9528	0.01645	0.9211	0.986
7	131	5	0.9164	0.02246	0.8734	0.962
8	119	5	0.8779	0.02733	0.8259	0.933
9	107	7	0.8205	0.03306	0.7582	0.888
10	95	8	0.7514	0.03825	0.6800	0.830
11	83	6	0.6971	0.04142	0.6204	0.783
12	71	8	0.6185	0.04511	0.5361	0.714
13	59	6	0.5556	0.04727	0.4703	0.656
14	48	4	0.5093	0.04867	0.4223	0.614
18	38	1	0.4959	0.04920	0.4083	0.602
23	37	2	0.4691	0.05006	0.3806	0.578
25	33	1	0.4549	0.05052	0.3659	0.566
28	32	2	0.4265	0.05121	0.3370	0.540
32	28	1	0.4112	0.05159	0.3216	0.526
35	27	2	0.3808	0.05208	0.2912	0.498
39	23	1	0.3642	0.05238	0.2748	0.483
42	22	2	0.3311	0.05259	0.2425	0.452
46	18	1	0.3127	0.05279	0.2246	0.435
47	17	1	0.2943	0.05279	0.2071	0.418
49	16	2	0.2575	0.05221	0.1731	0.383
56	12	2	0.2146	0.05158	0.1340	0.344
63	8	2	0.1610	0.05075	0.0868	0.299
70	4	2	0.0805	0.04757	0.0253	0.256

Strain=CC025

time	n.risk	n.event	survival	std.err	lower 95% CI	upper 95% CI
3	144	1	0.993	0.00692	0.980	1.000
5	125	1	0.985	0.01048	0.965	1.000
6	115	2	0.968	0.01582	0.937	0.999
7	105	1	0.959	0.01815	0.924	0.995
8	95	2	0.939	0.02270	0.895	0.984
9	85	1	0.928	0.02497	0.880	0.978
25	66	1	0.913	0.02827	0.860	0.971
36	40	1	0.891	0.03562	0.824	0.963

Strain=CC027

time	n.risk	n.event	survival	std.err	lower 95% CI	upper 95% CI
1	236	1	0.996	0.00423	0.988	1.000
3	209	1	0.991	0.00635	0.979	1.000
4	198	3	0.976	0.01063	0.955	0.997
6	170	2	0.965	0.01325	0.939	0.991
7	156	1	0.958	0.01454	0.930	0.987
9	129	1	0.951	0.01621	0.920	0.983
32	36	1	0.924	0.03044	0.867	0.986
39	30	1	0.894	0.04224	0.815	0.980
49	22	1	0.853	0.05657	0.749	0.971
53	18	1	0.806	0.07054	0.679	0.956
56	16	1	0.755	0.08216	0.610	0.935

57	12	1	0.692	0.09646	0.527	0.910
62	10	1	0.623	0.10886	0.442	0.878

Strain=CC032XCC013

time	n.risk	n.event	survival	std.err	lower 95% CI	upper 95% CI
1	240	1	0.996	0.00416	0.988	1
2	229	1	0.991	0.00600	0.980	1
34	60	1	0.975	0.01742	0.941	1

Strain=CC037

time	n.risk	n.event	survival	std.err	lower 95% CI	upper 95% CI
0	202	1	0.9950	0.00494	0.9854	1.000
2	180	1	0.9895	0.00738	0.9752	1.000
3	168	1	0.9836	0.00940	0.9654	1.000
5	144	3	0.9631	0.01489	0.9344	0.993
7	120	1	0.9551	0.01679	0.9228	0.989
8	108	2	0.9374	0.02062	0.8979	0.979
9	96	3	0.9081	0.02600	0.8586	0.961
10	84	2	0.8865	0.02954	0.8305	0.946
11	72	2	0.8619	0.03346	0.7987	0.930
12	60	4	0.8044	0.04178	0.7266	0.891
13	48	4	0.7374	0.04997	0.6457	0.842
14	42	2	0.7023	0.05340	0.6050	0.815
15	38	3	0.6468	0.05799	0.5426	0.771
18	34	1	0.6278	0.05932	0.5217	0.756
19	32	1	0.6082	0.06063	0.5003	0.739
25	28	1	0.5865	0.06223	0.4763	0.722
26	26	1	0.5639	0.06379	0.4518	0.704
32	24	1	0.5404	0.06532	0.4264	0.685
33	22	1	0.5159	0.06681	0.4002	0.665
39	20	1	0.4901	0.06827	0.3730	0.644
40	18	1	0.4628	0.06969	0.3445	0.622
46	16	1	0.4339	0.07109	0.3147	0.598
53	12	1	0.3977	0.07379	0.2765	0.572
54	10	1	0.3580	0.07638	0.2356	0.544
60	8	1	0.3132	0.07886	0.1912	0.513
61	6	1	0.2610	0.08118	0.1419	0.480
67	4	1	0.1958	0.08307	0.0852	0.450
68	2	1	0.0979	0.08072	0.0194	0.493

Strain=CC041XCC012

time	n.risk	n.event	survival	std.err	lower 95% CI	upper 95% CI
0	325	5	0.985	0.00683	0.971	0.998
1	293	1	0.981	0.00759	0.966	0.996
2	281	6	0.960	0.01126	0.938	0.983
3	259	5	0.942	0.01376	0.915	0.969
4	237	3	0.930	0.01521	0.901	0.960
5	225	2	0.922	0.01616	0.890	0.954
6	214	1	0.917	0.01665	0.885	0.950
9	182	2	0.907	0.01792	0.873	0.943
10	160	3	0.890	0.02010	0.852	0.930
13	149	1	0.884	0.02083	0.844	0.926
21	128	1	0.877	0.02179	0.836	0.921
27	112	4	0.846	0.02604	0.796	0.899
34	96	5	0.802	0.03126	0.743	0.866
41	80	5	0.752	0.03647	0.684	0.827
49	64	4	0.705	0.04106	0.629	0.790
55	48	3	0.661	0.04570	0.577	0.757
63	32	5	0.558	0.05732	0.456	0.682
69	16	4	0.418	0.07410	0.295	0.592

Strain=CC051

time	n.risk	n.event	survival	std.err	lower 95% CI	upper 95% CI
0	146	1	0.993	0.00683	0.980	1.000
2	128	1	0.985	0.01028	0.965	1.000
3	118	2	0.969	0.01546	0.939	0.999
4	108	1	0.960	0.01773	0.926	0.995
5	98	1	0.950	0.02007	0.911	0.990
10	48	1	0.930	0.02775	0.877	0.986

Strain=CC057

time	n.risk	n.event	survival	std.err	lower 95% CI	upper 95% CI
8	354	2	0.994	0.00398	0.987	1.000
10	318	1	0.991	0.00505	0.981	1.000
13	264	2	0.984	0.00729	0.970	0.998
14	248	1	0.980	0.00827	0.964	0.996
15	232	3	0.967	0.01093	0.946	0.989
16	216	4	0.949	0.01392	0.922	0.977
17	200	3	0.935	0.01595	0.904	0.967
18	184	3	0.920	0.01796	0.885	0.956
19	168	4	0.898	0.02060	0.858	0.939
20	152	3	0.880	0.02259	0.837	0.925
21	136	4	0.854	0.02536	0.806	0.905
22	120	3	0.833	0.02756	0.781	0.889
23	104	2	0.817	0.02927	0.761	0.876
24	88	6	0.761	0.03501	0.696	0.833
25	72	8	0.677	0.04199	0.599	0.764
26	56	4	0.628	0.04541	0.545	0.724
28	49	2	0.603	0.04704	0.517	0.702
35	42	2	0.574	0.04898	0.485	0.678
42	35	2	0.541	0.05138	0.449	0.652
49	28	2	0.502	0.05449	0.406	0.621
56	21	3	0.431	0.06045	0.327	0.567
63	14	1	0.400	0.06348	0.293	0.546
68	7	2	0.286	0.08197	0.163	0.501

Strain=CC078

time	n.risk	n.event	survival	std.err	lower 95% CI	upper 95% CI
0	258	1	0.996	0.00387	0.9886	1.000
8	245	2	0.988	0.00689	0.9746	1.000
9	231	3	0.975	0.01002	0.9557	0.995
10	217	6	0.948	0.01459	0.9200	0.977
11	203	5	0.925	0.01757	0.8910	0.960
12	190	3	0.910	0.01921	0.8734	0.949
13	178	3	0.895	0.02083	0.8550	0.937
14	166	4	0.873	0.02295	0.8295	0.919
15	154	5	0.845	0.02547	0.7965	0.896
16	142	5	0.815	0.02783	0.7625	0.872
17	130	5	0.784	0.03009	0.7271	0.845
18	119	3	0.764	0.03142	0.7050	0.828
19	109	1	0.757	0.03190	0.6971	0.822
20	99	2	0.742	0.03304	0.6798	0.809
21	89	3	0.717	0.03494	0.6515	0.789
22	79	4	0.681	0.03759	0.6107	0.758
23	69	2	0.661	0.03900	0.5886	0.742
24	59	3	0.627	0.04156	0.5508	0.714
25	49	3	0.589	0.04454	0.5077	0.683
26	39	3	0.543	0.04818	0.4568	0.647
28	35	1	0.528	0.04924	0.4398	0.634
35	30	2	0.493	0.05187	0.4009	0.606

```
   42     25       1    0.473 0.05341       0.3791        0.590
   49     20       2    0.426 0.05760       0.3266        0.555
   56     15       3    0.341 0.06369       0.2361        0.491
   63     10       3    0.238 0.06651       0.1380        0.412
   68      5       2    0.143 0.06574       0.0581        0.352

Call:
coxph(formula = Surv(Date.dpi, as.numeric(Weakness_HL)) ~ Sex,
    data = aData)

  n= 4200, number of events= 644

       coef exp(coef) se(coef)     z Pr(>|z|)
SexM 0.3237    1.3823   0.0794 4.077 4.56e-05 ***
---
Signif. codes:  0 ‚Äò***‚Äô 0.001 ‚Äò**‚Äô 0.01 ‚Äò*‚Äô 0.05 ‚Äò.‚Äô 0.1 ‚Äò ‚Äô 1

     exp(coef) exp(-coef) lower .95 upper .95
SexM     1.382     0.7234     1.183     1.615

Concordance= 0.55  (se = 0.011 )
Likelihood ratio test= 16.77  on 1 df,   p=4e-05
Wald test            = 16.62  on 1 df,   p=5e-05
Score (logrank) test = 16.77  on 1 df,   p=4e-05

Call:
coxph(formula = Surv(Date.dpi, as.numeric(Weakness_HL)) ~ Status,
    data = aData)

  n= 4200, number of events= 644

           coef exp(coef) se(coef)      z Pr(>|z|)
StatusU -2.6692    0.0693   0.2272 -11.75   <2e-16 ***
---
Signif. codes:  0 ‚Äò***‚Äô 0.001 ‚Äò**‚Äô 0.01 ‚Äò*‚Äô 0.05 ‚Äò.‚Äô 0.1 ‚Äò ‚Äô 1

        exp(coef) exp(-coef) lower .95 upper .95
StatusU    0.0693      14.43    0.0444    0.1082

Concordance= 0.641  (se = 0.006 )
Likelihood ratio test= 342  on 1 df,   p=<2e-16
Wald test            = 138.1  on 1 df,   p=<2e-16
Score (logrank) test = 242.1  on 1 df,   p=<2e-16

Call:
coxph(formula = Surv(Date.dpi, as.numeric(Weakness_HL)) ~ Strain,
    data = Z)

  n= 4396, number of events= 649

                      coef exp(coef) se(coef)      z Pr(>|z|)
StrainCC078         2.6766   14.5349   0.4613  5.802 6.55e-09 ***
StrainCC057         2.1081    8.2329   0.4639  4.544 5.52e-06 ***
StrainCC012         2.6120   13.6258   0.4594  5.685 1.31e-08 ***
StrainCC041XCC012  2.1280    8.3977   0.4662  4.565 5.01e-06 ***
StrainCC002         1.8788    6.5458   0.4802  3.913 9.12e-05 ***
StrainCC025         1.1818    3.2603   0.5478  2.157 0.030981 *
StrainCC012XCC032 -0.7895    0.4541   0.8370 -0.943 0.345525
StrainCC013XCC041 -0.6220    0.5369   0.8370 -0.743 0.457457
StrainCC032XCC013 -0.4975    0.6080   0.7307 -0.681 0.495938
```

```
StrainCC015        3.5580   35.0928   0.5091  6.989 2.77e-12 ***
StrainCC023        3.2941   26.9539   0.4618  7.134 9.76e-13 ***
StrainCC027        1.4772    4.3807   0.5129  2.880 0.003973 **
StrainCC005        2.8643   17.5368   0.4661  6.145 7.99e-10 ***
StrainCC011        2.1779    8.8276   0.4787  4.550 5.37e-06 ***
StrainCC051        1.6006    4.9560   0.5862  2.731 0.006321 **
StrainCC037        2.8847   17.8983   0.4724  6.106 1.02e-09 ***
StrainCC006        1.8338    6.2574   0.4982  3.681 0.000232 ***
StrainCC017        2.1018    8.1805   0.5171  4.064 4.81e-05 ***
---
Signif. codes:  0 ,Äò***,Äô 0.001 ,Äò**,Äô 0.01 ,Äò*,Äô 0.05 ,Äò.,Äô 0.1 ,Äò ,Äô 1

                  exp(coef) exp(-coef) lower .95 upper .95
StrainCC078         14.5349    0.06880   5.88489    35.899
StrainCC057          8.2329    0.12146   3.31622    20.439
StrainCC012         13.6258    0.07339   5.53717    33.530
StrainCC041XCC012    8.3977    0.11908   3.36771    20.940
StrainCC002          6.5458    0.15277   2.55417    16.775
StrainCC025          3.2603    0.30672   1.11417     9.540
StrainCC012XCC032    0.4541    2.20239   0.08803     2.342
StrainCC013XCC041    0.5369    1.86257   0.10409     2.769
StrainCC032XCC013    0.6080    1.64467   0.14519     2.546
StrainCC015         35.0928    0.02850  12.93878    95.179
StrainCC023         26.9539    0.03710  10.90355    66.631
StrainCC027          4.3807    0.22827   1.60322    11.970
StrainCC005         17.5368    0.05702   7.03400    43.722
StrainCC011          8.8276    0.11328   3.45448    22.558
StrainCC051          4.9560    0.20178   1.57104    15.634
StrainCC037         17.8983    0.05587   7.09041    45.181
StrainCC006          6.2574    0.15981   2.35698    16.612
StrainCC017          8.1805    0.12224   2.96906    22.539

Concordance= 0.699  (se = 0.012 )
Likelihood ratio test= 427.8  on 18 df,   p=<2e-16
Wald test            = 270.8  on 18 df,   p=<2e-16
Score (logrank) test = 416.8  on 18 df,   p=<2e-16
```

WEAKNESS HR

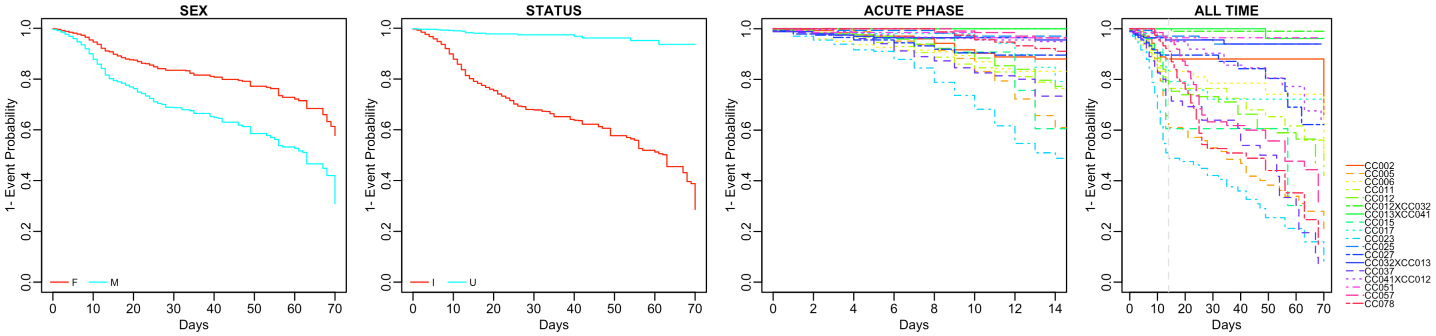


```
Call: survfit(formula = Surv(Date.dpi, as.numeric(Weakness_HR)) ~ Sex,
    data = aData)

                Sex=F
 time n.risk n.event survival  std.err lower 95% CI upper 95% CI
    0   2190       4    0.998 0.000912        0.996        1.000
    1   2084       5    0.996 0.001405        0.993        0.999
    2   1993      10    0.991 0.002106        0.987        0.995
    3   1883       6    0.988 0.002463        0.983        0.992
    4   1778       6    0.984 0.002805        0.979        0.990
    5   1678       6    0.981 0.003142        0.975        0.987
    6   1583       6    0.977 0.003477        0.970        0.984
    7   1480       7    0.972 0.003874        0.965        0.980
    8   1383      11    0.965 0.004491        0.956        0.974
    9   1272      14    0.954 0.005262        0.944        0.964
   10   1154      10    0.946 0.005830        0.934        0.957
   11   1055      10    0.937 0.006427        0.924        0.950
   12    965      16    0.921 0.007401        0.907        0.936
   13    879      10    0.911 0.008025        0.895        0.927
   14    799       3    0.907 0.008234        0.891        0.924
   15    736       8    0.898 0.008852        0.880        0.915
   16    696       5    0.891 0.009247        0.873        0.909
   17    667       5    0.884 0.009648        0.866        0.904
   18    645       4    0.879 0.009970        0.860        0.899
   19    617       2    0.876 0.010139        0.856        0.896
   20    597       1    0.875 0.010228        0.855        0.895
   21    580       4    0.869 0.010593        0.848        0.890
   22    540       4    0.862 0.010992        0.841        0.884
   23    523       1    0.861 0.011094        0.839        0.883
   24    498       3    0.855 0.011424        0.833        0.878
   25    481       4    0.848 0.011869        0.825        0.872
   26    457       4    0.841 0.012332        0.817        0.865
   28    422       3    0.835 0.012718        0.810        0.860
   33    366       1    0.833 0.012886        0.808        0.858
   34    357       4    0.823 0.013560        0.797        0.850
   35    335       3    0.816 0.014091        0.789        0.844
   39    301       2    0.810 0.014509        0.782        0.839
   40    291       1    0.808 0.014724        0.779        0.837
   42    263       3    0.798 0.015487        0.769        0.829
   46    224       1    0.795 0.015823        0.764        0.826
   47    214       1    0.791 0.016179        0.760        0.823
   49    205       5    0.772 0.017938        0.737        0.808
   53    167       1    0.767 0.018417        0.732        0.804
   54    158       1    0.762 0.018930        0.726        0.800
   56    137       5    0.735 0.021951        0.693        0.779
   57    113       1    0.728 0.022698        0.685        0.774
   60    111       1    0.721 0.023422        0.677        0.769
   61    103       1    0.714 0.024220        0.669        0.764
   63     94       4    0.684 0.027550        0.632        0.740
```

67	56	2	0.660	0.031520	0.601	0.724
68	48	2	0.632	0.035699	0.566	0.706
69	34	1	0.614	0.039193	0.541	0.695
70	17	1	0.577	0.050859	0.486	0.686

Sex=M

time	n.risk	n.event	survival	std.err	lower 95% CI	upper 95% CI
0	2010	10	0.995	0.00157	0.992	0.998
1	1919	8	0.991	0.00214	0.987	0.995
2	1838	11	0.985	0.00278	0.980	0.990
3	1734	15	0.976	0.00352	0.970	0.983
4	1633	14	0.968	0.00414	0.960	0.976
5	1540	14	0.959	0.00472	0.950	0.969
6	1454	19	0.947	0.00547	0.936	0.957
7	1356	17	0.935	0.00611	0.923	0.947
8	1270	21	0.919	0.00688	0.906	0.933
9	1169	25	0.900	0.00777	0.885	0.915
10	1058	26	0.878	0.00871	0.861	0.895
11	964	21	0.859	0.00946	0.840	0.877
12	883	17	0.842	0.01010	0.822	0.862
13	809	25	0.816	0.01104	0.795	0.838
14	730	13	0.801	0.01156	0.779	0.824
15	669	6	0.794	0.01182	0.771	0.818
16	631	4	0.789	0.01201	0.766	0.813
17	607	4	0.784	0.01221	0.760	0.808
18	587	6	0.776	0.01252	0.752	0.801
19	558	5	0.769	0.01279	0.744	0.795
20	539	5	0.762	0.01306	0.737	0.788
21	525	9	0.749	0.01354	0.723	0.776
22	487	4	0.743	0.01377	0.716	0.770
23	473	6	0.733	0.01413	0.706	0.761
24	452	6	0.724	0.01449	0.696	0.752
25	438	8	0.710	0.01496	0.682	0.740
26	414	4	0.703	0.01520	0.674	0.734
27	398	2	0.700	0.01533	0.671	0.731
28	384	6	0.689	0.01573	0.659	0.721
30	356	1	0.687	0.01580	0.657	0.719
32	349	4	0.679	0.01610	0.648	0.711
34	330	2	0.675	0.01627	0.644	0.708
35	313	5	0.664	0.01671	0.632	0.698
39	286	5	0.653	0.01720	0.620	0.687
40	275	2	0.648	0.01740	0.615	0.683
41	267	1	0.645	0.01750	0.612	0.681
42	252	6	0.630	0.01818	0.595	0.667
46	219	4	0.619	0.01873	0.583	0.656
47	208	2	0.613	0.01902	0.576	0.651
49	199	9	0.585	0.02028	0.547	0.626
53	163	2	0.578	0.02066	0.539	0.620
54	153	2	0.570	0.02106	0.530	0.613
55	148	2	0.563	0.02147	0.522	0.606
56	134	6	0.537	0.02284	0.494	0.584
57	110	1	0.532	0.02315	0.489	0.580
60	108	1	0.528	0.02345	0.483	0.576
61	99	2	0.517	0.02416	0.472	0.566
62	94	1	0.511	0.02452	0.465	0.562
63	90	8	0.466	0.02710	0.416	0.522
67	54	2	0.449	0.02871	0.396	0.509
68	46	3	0.419	0.03142	0.362	0.486
70	19	5	0.309	0.04828	0.228	0.420

```
Call: survfit(formula = Surv(Date.dpi, as.numeric(Weakness_HR)) ~ Status,
    data = aData)

                Status=I
 time n.risk n.event survival std.err lower 95% CI upper 95% CI
    0   2809      11    0.996 0.00118        0.994        0.998
    1   2667      11    0.992 0.00170        0.989        0.995
    2   2560      20    0.984 0.00242        0.980        0.989
    3   2420      21    0.976 0.00303        0.970        0.982
    4   2287      20    0.967 0.00355        0.960        0.974
    5   2165      20    0.958 0.00404        0.950        0.966
    6   2054      23    0.947 0.00458        0.939        0.956
    7   1926      23    0.936 0.00509        0.926        0.946
    8   1811      32    0.920 0.00578        0.908        0.931
    9   1669      38    0.899 0.00657        0.886        0.912
   10   1511      35    0.878 0.00730        0.864        0.892
   11   1380      31    0.858 0.00795        0.843        0.874
   12   1267      32    0.836 0.00862        0.820        0.854
   13   1165      32    0.814 0.00929        0.795        0.832
   14   1057      16    0.801 0.00965        0.782        0.820
   15    973      13    0.790 0.00997        0.771        0.810
   16    913       9    0.783 0.01020        0.763        0.803
   17    879       9    0.775 0.01044        0.754        0.795
   18    849       9    0.766 0.01068        0.746        0.788
   19    802       7    0.760 0.01089        0.739        0.781
   20    775       6    0.754 0.01106        0.733        0.776
   21    756      13    0.741 0.01144        0.719        0.764
   22    700       8    0.732 0.01170        0.710        0.756
   23    681       7    0.725 0.01192        0.702        0.749
   24    654       9    0.715 0.01221        0.691        0.739
   25    635      12    0.701 0.01259        0.677        0.727
   26    598       7    0.693 0.01281        0.669        0.719
   27    575       2    0.691 0.01288        0.666        0.717
   28    551       9    0.680 0.01321        0.654        0.706
   30    519       1    0.678 0.01325        0.653        0.705
   32    512       4    0.673 0.01341        0.647        0.700
   33    493       1    0.672 0.01345        0.646        0.698
   34    483       6    0.663 0.01371        0.637        0.691
   35    455       8    0.652 0.01407        0.625        0.680
   39    420       7    0.641 0.01442        0.613        0.670
   40    401       2    0.638 0.01453        0.610        0.667
   41    391       1    0.636 0.01458        0.608        0.665
   42    364       8    0.622 0.01508        0.593        0.652
   46    318       5    0.612 0.01546        0.583        0.643
   47    299       3    0.606 0.01571        0.576        0.638
   49    288      14    0.577 0.01680        0.545        0.610
   53    235       3    0.569 0.01712        0.537        0.604
   54    217       2    0.564 0.01735        0.531        0.599
   55    212       2    0.559 0.01759        0.525        0.594
   56    189      11    0.526 0.01911        0.490        0.565
   57    158       2    0.519 0.01944        0.483        0.559
   60    154       2    0.513 0.01976        0.475        0.553
   61    139       2    0.505 0.02015        0.467        0.546
   62    134       1    0.502 0.02035        0.463        0.543
   63    129      12    0.455 0.02248        0.413        0.501
   67     76       4    0.431 0.02427        0.386        0.481
   68     62       5    0.396 0.02683        0.347        0.452
   69     46       1    0.388 0.02760        0.337        0.446
   70     23       6    0.286 0.04093        0.216        0.379
```

```
              Status=U
 time n.risk n.event survival std.err lower 95% CI upper 95% CI
    0   1391       3    0.998 0.00124        0.995        1.000
    1   1336       2    0.996 0.00163        0.993        1.000
    2   1271       1    0.996 0.00181        0.992        0.999
    6    983       2    0.994 0.00230        0.989        0.998
    7    910       1    0.992 0.00255        0.987        0.997
    9    772       1    0.991 0.00285        0.986        0.997
   10    701       1    0.990 0.00318        0.984        0.996
   12    581       1    0.988 0.00360        0.981        0.995
   13    523       3    0.982 0.00484        0.973        0.992
   15    432       1    0.980 0.00534        0.970        0.991
   18    383       1    0.978 0.00591        0.966        0.989
   26    273       1    0.974 0.00688        0.961        0.988
   40    165       1    0.968 0.00903        0.951        0.986
   42    151       1    0.962 0.01101        0.940        0.983
   54     94       1    0.951 0.01491        0.923        0.981
   61     63       1    0.936 0.02097        0.896        0.978

Call: survfit(formula = Surv(Date.dpi, as.numeric(Weakness_HR)) ~ Strain,
    data = aData)

              Strain=CC002
 time n.risk n.event survival std.err lower 95% CI upper 95% CI
    0    284       1    0.996 0.00351        0.990        1.000
    2    258       1    0.993 0.00521        0.982        1.000
    3    242       2    0.984 0.00775        0.969        1.000
    4    226       2    0.976 0.00983        0.957        0.995
    5    210       1    0.971 0.01082        0.950        0.993
    7    178       2    0.960 0.01317        0.935        0.986
    8    162       3    0.942 0.01645        0.911        0.975
    9    148       4    0.917 0.02034        0.878        0.958
   10    134       2    0.903 0.02222        0.861        0.948
   11    126       2    0.889 0.02407        0.843        0.937
   13    110       1    0.881 0.02517        0.833        0.932
   70      4       1    0.661 0.19163        0.374        1.000

              Strain=CC005
 time n.risk n.event survival std.err lower 95% CI upper 95% CI
    3    186       1    0.995 0.00536        0.984        1.000
    4    174       1    0.989 0.00780        0.974        1.000
    5    162       3    0.971 0.01298        0.945        0.996
    6    150       3    0.951 0.01688        0.919        0.985
    7    138       2    0.937 0.01924        0.900        0.976
    8    126       3    0.915 0.02269        0.872        0.961
    9    114       4    0.883 0.02698        0.832        0.937
   10    102       6    0.831 0.03268        0.769        0.898
   11     90       4    0.794 0.03607        0.726        0.868
   12     78       7    0.723 0.04170        0.646        0.809
   13     66       6    0.657 0.04573        0.573        0.753
   14     56       4    0.610 0.04811        0.523        0.712
   19     48       1    0.597 0.04876        0.509        0.701
   21     46       2    0.571 0.04998        0.481        0.678
   26     42       1    0.558 0.05061        0.467        0.666
   28     40       2    0.530 0.05178        0.438        0.642
   33     36       1    0.515 0.05239        0.422        0.629
   35     34       2    0.485 0.05351        0.391        0.602
   40     30       1    0.469 0.05412        0.374        0.588
   42     28       3    0.419 0.05555        0.323        0.543
   47     24       1    0.401 0.05590        0.305        0.527
```

49	22	1	0.383	0.05626	0.287	0.511
54	18	1	0.362	0.05701	0.265	0.493
56	16	1	0.339	0.05775	0.243	0.473
61	12	1	0.311	0.05945	0.214	0.452
63	10	1	0.280	0.06109	0.182	0.429
70	4	1	0.210	0.07594	0.103	0.426

Strain=CC006

time	n.risk	n.event	survival	std.err	lower 95% CI	upper 95% CI
0	262	2	0.992	0.00538	0.982	1.000
1	250	3	0.980	0.00866	0.964	0.998
2	234	5	0.960	0.01256	0.935	0.984
3	218	2	0.951	0.01390	0.924	0.978
4	202	3	0.937	0.01590	0.906	0.968
5	186	2	0.927	0.01725	0.893	0.961
6	170	2	0.916	0.01869	0.880	0.953
8	138	1	0.909	0.01970	0.871	0.948
9	123	2	0.894	0.02198	0.852	0.938
10	109	3	0.870	0.02556	0.821	0.921
11	95	3	0.842	0.02926	0.787	0.901
12	81	1	0.832	0.03069	0.774	0.894
23	38	1	0.810	0.03687	0.741	0.885
28	33	1	0.785	0.04315	0.705	0.875
49	18	1	0.742	0.05881	0.635	0.866
70	3	1	0.494	0.20563	0.219	1.000

Strain=CC011

time	n.risk	n.event	survival	std.err	lower 95% CI	upper 95% CI
3	216	4	0.981	0.00917	0.964	1.000
4	202	3	0.967	0.01231	0.943	0.991
5	187	4	0.946	0.01580	0.916	0.978
6	173	3	0.930	0.01815	0.895	0.966
7	159	4	0.906	0.02113	0.866	0.949
8	145	3	0.888	0.02330	0.843	0.935
9	131	4	0.861	0.02623	0.811	0.914
10	117	2	0.846	0.02777	0.793	0.902
11	103	2	0.829	0.02956	0.773	0.889
12	89	2	0.811	0.03170	0.751	0.875
13	75	2	0.789	0.03434	0.725	0.859
14	64	2	0.765	0.03744	0.695	0.842
35	39	2	0.725	0.04462	0.643	0.818
42	32	2	0.680	0.05208	0.585	0.790
49	25	1	0.653	0.05666	0.551	0.774
56	18	1	0.617	0.06407	0.503	0.756
63	11	1	0.560	0.07905	0.425	0.739
70	4	1	0.420	0.13505	0.224	0.789

Strain=CC012

time	n.risk	n.event	survival	std.err	lower 95% CI	upper 95% CI
0	468	2	0.996	0.00302	0.990	1.000
1	444	1	0.993	0.00375	0.986	1.000
2	420	4	0.984	0.00600	0.972	0.996
3	396	2	0.979	0.00692	0.966	0.993
4	372	1	0.976	0.00739	0.962	0.991
5	348	2	0.971	0.00834	0.955	0.987
6	324	4	0.959	0.01017	0.939	0.979
7	300	6	0.940	0.01262	0.915	0.965
8	276	5	0.923	0.01451	0.895	0.952
9	252	5	0.904	0.01637	0.873	0.937
10	228	5	0.884	0.01825	0.849	0.921

11	204	7	0.854	0.02092	0.814	0.896
12	180	3	0.840	0.02213	0.798	0.884
13	156	8	0.797	0.02571	0.748	0.849
14	132	4	0.773	0.02762	0.720	0.829
15	120	3	0.753	0.02909	0.698	0.813
18	108	2	0.739	0.03018	0.683	0.801
25	96	1	0.732	0.03083	0.674	0.795
32	71	2	0.711	0.03323	0.649	0.779
39	59	4	0.663	0.03875	0.591	0.743
46	47	4	0.606	0.04455	0.525	0.700
53	35	1	0.589	0.04652	0.505	0.688
60	24	1	0.565	0.05065	0.474	0.673
67	12	2	0.470	0.07397	0.346	0.640

Strain=CC012XCC032

time	n.risk	n.event	survival	std.err	lower 95% CI	upper 95% CI
1.50e+01	1.02e+02	1.00e+00	9.90e-01	9.76e-03	9.71e-01	1.00e+00

Strain=CC013XCC041

time	n.risk	n.event	survival	std.err	lower 95% CI	upper 95% CI
49.0000	26.0000	1.0000	0.9615	0.0377	0.8904	1.0000

Strain=CC015

time	n.risk	n.event	survival	std.err	lower 95% CI	upper 95% CI
5	26	1	0.962	0.0377	0.8904	1.000
9	18	1	0.908	0.0630	0.7927	1.000
12	12	2	0.757	0.1109	0.5678	1.000
13	10	2	0.605	0.1305	0.3968	0.924
57	2	1	0.303	0.2238	0.0711	1.000

Strain=CC017

time	n.risk	n.event	survival	std.err	lower 95% CI	upper 95% CI
2	105	2	0.981	0.0133	0.955	1.000
10	54	1	0.963	0.0223	0.920	1.000
11	48	1	0.943	0.0295	0.887	1.000
12	42	2	0.898	0.0418	0.820	0.984
13	36	2	0.848	0.0523	0.751	0.957
14	30	2	0.791	0.0622	0.678	0.923
21	24	1	0.758	0.0678	0.637	0.904
28	21	1	0.722	0.0736	0.592	0.882

Strain=CC023

time	n.risk	n.event	survival	std.err	lower 95% CI	upper 95% CI
0	209	2	0.9904	0.00673	0.9773	1.000
1	201	4	0.9707	0.01178	0.9479	0.994
2	189	3	0.9553	0.01457	0.9272	0.984
3	177	3	0.9391	0.01706	0.9063	0.973
4	167	4	0.9166	0.02002	0.8782	0.957
5	155	1	0.9107	0.02074	0.8709	0.952
6	143	5	0.8789	0.02442	0.8323	0.928
7	131	5	0.8453	0.02772	0.7927	0.901
8	119	8	0.7885	0.03233	0.7276	0.854
9	107	7	0.7369	0.03561	0.6703	0.810
10	95	7	0.6826	0.03845	0.6113	0.762
11	83	8	0.6168	0.04118	0.5412	0.703
12	71	8	0.5473	0.04326	0.4688	0.639

13	59	4	0.5102	0.04412	0.4307	0.604
14	48	2	0.4890	0.04477	0.4086	0.585
18	38	1	0.4761	0.04540	0.3949	0.574
23	37	1	0.4632	0.04596	0.3813	0.563
25	33	1	0.4492	0.04667	0.3664	0.551
28	32	2	0.4211	0.04779	0.3371	0.526
32	28	1	0.4061	0.04839	0.3215	0.513
35	27	2	0.3760	0.04926	0.2908	0.486
39	23	1	0.3596	0.04975	0.2742	0.472
42	22	2	0.3269	0.05032	0.2418	0.442
46	18	1	0.3088	0.05069	0.2238	0.426
47	17	1	0.2906	0.05086	0.2062	0.410
49	16	2	0.2543	0.05058	0.1722	0.376
56	12	2	0.2119	0.05025	0.1331	0.337
63	8	2	0.1589	0.04973	0.0861	0.293
70	4	2	0.0795	0.04687	0.0250	0.252

Strain=CC025

time	n.risk	n.event	survival	std.err	lower 95% CI	upper 95% CI
3	144	1	0.993	0.00692	0.980	1.000
8	95	1	0.983	0.01245	0.959	1.000
9	85	1	0.971	0.01684	0.939	1.000
25	66	1	0.956	0.02209	0.914	1.000
30	57	1	0.940	0.02734	0.887	0.995

Strain=CC027

time	n.risk	n.event	survival	std.err	lower 95% CI	upper 95% CI
0	246	2	0.992	0.00573	0.981	1.000
1	236	3	0.979	0.00918	0.961	0.997
2	223	1	0.975	0.01013	0.955	0.995
3	209	2	0.966	0.01199	0.942	0.989
4	198	2	0.956	0.01371	0.929	0.983
6	170	2	0.945	0.01569	0.914	0.976
7	156	2	0.932	0.01767	0.898	0.968
8	142	2	0.919	0.01971	0.881	0.959
9	129	2	0.905	0.02183	0.863	0.949
11	101	1	0.896	0.02338	0.851	0.943
32	36	1	0.871	0.03345	0.808	0.939
39	30	1	0.842	0.04314	0.762	0.931
49	22	1	0.804	0.05563	0.702	0.921
56	16	1	0.754	0.07132	0.626	0.907
57	12	1	0.691	0.08882	0.537	0.889
62	10	1	0.622	0.10337	0.449	0.861

Strain=CC032XCC013

time	n.risk	n.event	survival	std.err	lower 95% CI	upper 95% CI
0	262	3	0.989	0.00657	0.976	1.000
2	229	2	0.980	0.00891	0.963	0.998
3	209	1	0.975	0.01003	0.956	0.995
4	189	1	0.970	0.01122	0.948	0.992
5	179	1	0.965	0.01240	0.941	0.989
13	109	1	0.956	0.01512	0.927	0.986
34	60	1	0.940	0.02169	0.898	0.983

Strain=CC037

time	n.risk	n.event	survival	std.err	lower 95% CI	upper 95% CI
0	202	2	0.9901	0.00697	0.97654	1.000
1	192	1	0.9849	0.00863	0.96817	1.000
2	180	1	0.9795	0.01017	0.95974	1.000
3	168	1	0.9736	0.01166	0.95105	0.997

4	156	2	0.9612	0.01447	0.93321	0.990
5	144	3	0.9411	0.01821	0.90611	0.978
6	132	4	0.9126	0.02256	0.86945	0.958
7	120	3	0.8898	0.02556	0.84109	0.941
8	108	2	0.8733	0.02761	0.82085	0.929
9	96	3	0.8460	0.03092	0.78755	0.909
10	84	2	0.8259	0.03330	0.76313	0.894
11	72	1	0.8144	0.03476	0.74906	0.885
12	60	1	0.8008	0.03673	0.73199	0.876
13	48	4	0.7341	0.04642	0.64854	0.831
15	38	1	0.7148	0.04905	0.62483	0.818
19	32	1	0.6924	0.05236	0.59707	0.803
26	26	2	0.6392	0.06038	0.53116	0.769
39	20	1	0.6072	0.06527	0.49187	0.750
40	18	2	0.5398	0.07341	0.41345	0.705
47	14	1	0.5012	0.07763	0.36997	0.679
53	12	2	0.4177	0.08422	0.28132	0.620
54	10	2	0.3341	0.08562	0.20221	0.552
60	8	1	0.2924	0.08449	0.16594	0.515
61	6	2	0.1949	0.07962	0.08753	0.434
67	4	2	0.0975	0.06292	0.02749	0.345
68	2	1	0.0487	0.04666	0.00746	0.318

Strain=CC041XCC012

time	n.risk	n.event	survival	std.err	lower 95% CI	upper 95% CI
2	281	1	0.996	0.00355	0.990	1.000
3	259	2	0.989	0.00647	0.976	1.000
4	237	1	0.985	0.00767	0.970	1.000
5	225	2	0.976	0.00978	0.957	0.995
6	214	2	0.967	0.01162	0.944	0.990
8	193	1	0.962	0.01260	0.937	0.987
10	160	1	0.956	0.01388	0.929	0.983
13	149	1	0.949	0.01519	0.920	0.980
21	128	4	0.920	0.02073	0.880	0.961
27	112	2	0.903	0.02339	0.858	0.950
34	96	5	0.856	0.03018	0.799	0.917
41	80	1	0.845	0.03165	0.786	0.910
49	64	3	0.806	0.03753	0.736	0.883
55	48	2	0.772	0.04283	0.693	0.861
63	32	4	0.676	0.05867	0.570	0.801
69	16	1	0.633	0.06854	0.512	0.783

Strain=CC051

time	n.risk	n.event	survival	std.err	lower 95% CI	upper 95% CI
1	138	1	0.993	0.00722	0.979	1
2	128	1	0.985	0.01054	0.965	1
10	48	1	0.964	0.02278	0.921	1

Strain=CC057

time	n.risk	n.event	survival	std.err	lower 95% CI	upper 95% CI
9	336	3	0.991	0.00513	0.981	1.000
10	318	2	0.985	0.00673	0.972	0.998
12	282	4	0.971	0.00960	0.952	0.990
13	264	2	0.964	0.01084	0.942	0.985
15	232	2	0.955	0.01224	0.932	0.979
16	216	4	0.938	0.01487	0.909	0.967
17	200	4	0.919	0.01727	0.886	0.953
18	184	5	0.894	0.02009	0.855	0.934
19	168	4	0.873	0.02225	0.830	0.917
20	152	3	0.855	0.02393	0.810	0.904

```
   21    136       4    0.830 0.02633        0.780        0.883
   22    120       4    0.802 0.02886        0.748        0.861
   23    104       3    0.779 0.03097        0.721        0.842
   24     88       5    0.735 0.03497        0.670        0.807
   25     72       5    0.684 0.03929        0.611        0.766
   26     56       2    0.660 0.04151        0.583        0.746
   28     49       2    0.633 0.04397        0.552        0.725
   35     42       1    0.618 0.04543        0.535        0.713
   42     35       1    0.600 0.04743        0.514        0.700
   49     28       2    0.557 0.05284        0.463        0.671
   56     21       3    0.478 0.06214        0.370        0.616
   63     14       1    0.443 0.06640        0.331        0.595
   68      7       2    0.317 0.08934        0.182        0.551

                Strain=CC078 
 time n.risk n.event survival std.err lower 95% CI upper 95% CI
    8    245       3    0.988 0.00703       0.9741        1.000
    9    231       3    0.975 0.01011       0.9553        0.995
   10    217       4    0.957 0.01333       0.9312        0.983
   11    203       2    0.948 0.01477       0.9190        0.977
   12    190       3    0.933 0.01688       0.9001        0.966
   13    178       2    0.922 0.01824       0.8870        0.959
   14    166       2    0.911 0.01964       0.8733        0.950
   15    154       7    0.870 0.02419       0.8234        0.918
   16    142       5    0.839 0.02694       0.7878        0.893
   17    130       5    0.807 0.02952       0.7509        0.867
   18    119       2    0.793 0.03054       0.7355        0.855
   19    109       1    0.786 0.03111       0.7272        0.849
   20     99       3    0.762 0.03307       0.6999        0.830
   21     89       2    0.745 0.03447       0.6803        0.816
   22     79       4    0.707 0.03753       0.6373        0.785
   23     69       2    0.687 0.03914       0.6141        0.768
   24     59       4    0.640 0.04285       0.5614        0.730
   25     49       4    0.588 0.04665       0.5032        0.687
   26     39       3    0.543 0.04983       0.4533        0.650
   28     35       1    0.527 0.05076       0.4365        0.637
   35     30       1    0.510 0.05202       0.4172        0.622
   42     25       1    0.489 0.05379       0.3944        0.607
   49     20       2    0.440 0.05848       0.3394        0.571
   56     15       3    0.352 0.06524       0.2450        0.506
   63     10       3    0.247 0.06849       0.1430        0.425
   68      5       2    0.148 0.06787       0.0602        0.364

Call:
coxph(formula = Surv(Date.dpi, as.numeric(Weakness_HR)) ~ Sex, 
    data = aData)

  n= 4200, number of events= 620 

        coef exp(coef) se(coef)     z Pr(>|z|)    
SexM 0.73119   2.07755  0.08457 8.646   <2e-16 ***
---
Signif. codes:  0 ‚Äò***‚Äô 0.001 ‚Äò**‚Äô 0.01 ‚Äò*‚Äô 0.05 ‚Äò.‚Äô 0.1 ‚Äò ‚Äô 1

     exp(coef) exp(-coef) lower .95 upper .95
SexM     2.078     0.4813      1.76     2.452

Concordance= 0.593  (se = 0.011 )
Likelihood ratio test= 79.04  on 1 df,   p=<2e-16
Wald test            = 74.75  on 1 df,   p=<2e-16
```

```
Score (logrank) test = 78.14  on 1 df,   p=<2e-16

Call:
coxph(formula = Surv(Date.dpi, as.numeric(Weakness_HR)) ~ Status, 
    data = aData)

  n= 4200, number of events= 620 

            coef exp(coef) se(coef)      z Pr(>|z|)    
StatusU -2.53682   0.07912  0.21710 -11.69   <2e-16 ***
---
Signif. codes:  0 ‚Äò***‚Äô 0.001 ‚Äò**‚Äô 0.01 ‚Äò*‚Äô 0.05 ‚Äò.‚Äô 0.1 ‚Äò ‚Äô 1

        exp(coef) exp(-coef) lower .95 upper .95
StatusU   0.07912      12.64    0.0517    0.1211

Concordance= 0.641  (se = 0.006 )
Likelihood ratio test= 316.9  on 1 df,   p=<2e-16
Wald test            = 136.5  on 1 df,   p=<2e-16
Score (logrank) test = 227.3  on 1 df,   p=<2e-16

Call:
coxph(formula = Surv(Date.dpi, as.numeric(Weakness_HR)) ~ Strain, 
    data = Z)

  n= 4396, number of events= 622 

                     coef exp(coef) se(coef)      z Pr(>|z|)    
StrainCC078        3.4938   32.9114   0.7168  4.874 1.09e-06 ***
StrainCC057        3.0177   20.4436   0.7176  4.205 2.61e-05 ***
StrainCC012        3.3605   28.8038   0.7162  4.692 2.71e-06 ***
StrainCC041XCC012  2.4773   11.9086   0.7286  3.400 0.000674 ***
StrainCC002        2.3983   11.0042   0.7387  3.247 0.001168 ** 
StrainCC025        1.4145    4.1143   0.8367  1.690 0.090934 .  
StrainCC012XCC032 -0.5567    0.5731   1.2250 -0.454 0.649482    
StrainCC013XCC041 -0.3880    0.6784   1.2250 -0.317 0.751428    
StrainCC032XCC013  1.6502    5.2080   0.7750  2.129 0.033221 *  
StrainCC015        3.5785   35.8185   0.8020  4.462 8.12e-06 ***
StrainCC023        4.3644   78.6029   0.7151  6.103 1.04e-09 ***
StrainCC027        2.8403   17.1214   0.7352  3.863 0.000112 ***
StrainCC005        3.8526   47.1132   0.7185  5.362 8.23e-08 ***
StrainCC011        3.3097   27.3766   0.7238  4.573 4.81e-06 ***
StrainCC051        1.6569    5.2431   0.9133  1.814 0.069643 .  
StrainCC037        3.8816   48.5025   0.7220  5.376 7.61e-08 ***
StrainCC006        3.1995   24.5209   0.7286  4.391 1.13e-05 ***
StrainCC017        2.7987   16.4227   0.7643  3.662 0.000250 ***
---
Signif. codes:  0 ‚Äò***‚Äô 0.001 ‚Äò**‚Äô 0.01 ‚Äò*‚Äô 0.05 ‚Äò.‚Äô 0.1 ‚Äò ‚Äô 1

                  exp(coef) exp(-coef) lower .95 upper .95
StrainCC078         32.9114    0.03038   8.07603   134.120
StrainCC057         20.4436    0.04892   5.00845    83.447
StrainCC012         28.8038    0.03472   7.07596   117.250
StrainCC041XCC012   11.9086    0.08397   2.85518    49.669
StrainCC002         11.0042    0.09087   2.58679    46.812
StrainCC025          4.1143    0.24305   0.79814    21.209
StrainCC012XCC032    0.5731    1.74495   0.05194     6.323
StrainCC013XCC041    0.6784    1.47407   0.06148     7.485
StrainCC032XCC013    5.2080    0.19201   1.14032    23.786
StrainCC015         35.8185    0.02792   7.43767   172.495
```

```
StrainCC023         78.6029    0.01272  19.35099   319.282
StrainCC027         17.1214    0.05841   4.05246    72.337
StrainCC005         47.1132    0.02123  11.52300   192.628
StrainCC011         27.3766    0.03653   6.62704   113.094
StrainCC051          5.2431    0.19073   0.87537    31.404
StrainCC037         48.5025    0.02062  11.78085   199.688
StrainCC006         24.5209    0.04078   5.87956   102.265
StrainCC017         16.4227    0.06089   3.67204    73.448

Concordance= 0.713  (se = 0.012 )
Likelihood ratio test= 465.6  on 18 df,   p=<2e-16
Wald test            = 295.6  on 18 df,   p=<2e-16
Score (logrank) test = 476.2  on 18 df,   p=<2e-16
```

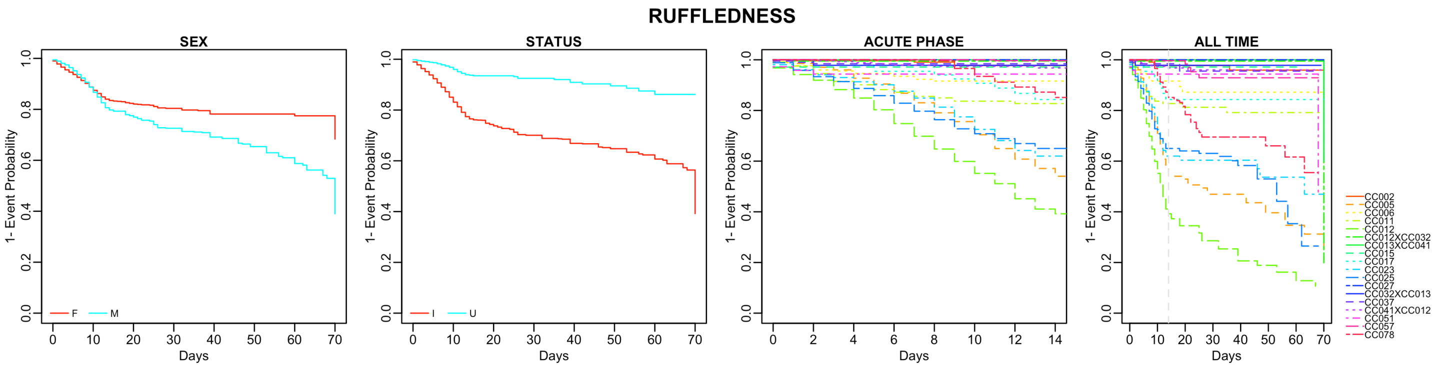


```
Call: survfit(formula = Surv(Date.dpi, as.numeric(Ruffledness)) ~ Sex,
    data = aData)

1 observation deleted due to missingness
                Sex=F
 time n.risk n.event survival std.err lower 95% CI upper 95% CI
    0   2189      22    0.990 0.00213        0.986        0.994
    1   2083      25    0.978 0.00316        0.972        0.984
    2   1992      25    0.966 0.00396        0.958        0.974
    3   1882      21    0.955 0.00456        0.946        0.964
    4   1777      17    0.946 0.00503        0.936        0.956
    5   1677      16    0.937 0.00546        0.926        0.948
    6   1582      19    0.926 0.00598        0.914        0.937
    7   1479      20    0.913 0.00652        0.900        0.926
    8   1382      17    0.902 0.00699        0.888        0.916
    9   1271      20    0.888 0.00756        0.873        0.903
   10   1153      17    0.875 0.00809        0.859        0.891
   11   1054      15    0.862 0.00859        0.845        0.879
   12    964      15    0.849 0.00913        0.831        0.867
   13    878       9    0.840 0.00948        0.822        0.859
   14    799       5    0.835 0.00971        0.816        0.854
   15    736       2    0.832 0.00982        0.813        0.852
   16    696       1    0.831 0.00987        0.812        0.851
   17    667       1    0.830 0.00994        0.811        0.850
   18    645       3    0.826 0.01014        0.807        0.846
   19    617       2    0.824 0.01028        0.804        0.844
   20    597       2    0.821 0.01043        0.801        0.841
   21    580       1    0.819 0.01051        0.799        0.840
   22    540       1    0.818 0.01060        0.797        0.839
   24    498       1    0.816 0.01070        0.795        0.837
   25    481       3    0.811 0.01103        0.790        0.833
   26    457       3    0.806 0.01138        0.784        0.828
   28    422       1    0.804 0.01151        0.782        0.827
   32    376       3    0.797 0.01200        0.774        0.821
   36    310       1    0.795 0.01223        0.771        0.819
   39    301       5    0.782 0.01338        0.756        0.808
   60    111       1    0.775 0.01500        0.746        0.805
   70     17       2    0.683 0.06196        0.572        0.816

                Sex=M
 time n.risk n.event survival std.err lower 95% CI upper 95% CI
    0   2010      11    0.995 0.00165        0.991        0.998
    1   1919      10    0.989 0.00231        0.985        0.994
    2   1838      16    0.981 0.00314        0.975        0.987
    3   1734      11    0.975 0.00364        0.967        0.982
    4   1633      18    0.964 0.00439        0.955        0.972
    5   1540      20    0.951 0.00515        0.941        0.961
    6   1454      22    0.937 0.00592        0.925        0.949
    7   1356      20    0.923 0.00659        0.910        0.936
```

```
    8   1270      23    0.906 0.00733        0.892        0.921
    9   1169      25    0.887 0.00813        0.871        0.903
   10   1058      24    0.867 0.00893        0.850        0.884
   11    964      21    0.848 0.00964        0.829        0.867
   12    883      23    0.826 0.01043        0.806        0.847
   13    809      19    0.806 0.01109        0.785        0.828
   14    730       8    0.798 0.01140        0.776        0.820
   15    669       4    0.793 0.01158        0.770        0.816
   18    587      10    0.779 0.01215        0.756        0.804
   19    558       3    0.775 0.01232        0.751        0.800
   20    539       4    0.769 0.01256        0.745        0.794
   21    525       4    0.764 0.01280        0.739        0.789
   22    487       4    0.757 0.01307        0.732        0.783
   24    452       2    0.754 0.01323        0.728        0.780
   25    438       8    0.740 0.01385        0.713        0.768
   26    414       7    0.728 0.01440        0.700        0.756
   28    384       1    0.726 0.01449        0.698        0.755
   32    349       6    0.713 0.01511        0.684        0.744
   35    313       1    0.711 0.01523        0.682        0.741
   37    289       1    0.709 0.01538        0.679        0.739
   39    286       7    0.691 0.01634        0.660        0.724
   42    252       2    0.686 0.01666        0.654        0.719
   46    219       6    0.667 0.01788        0.633        0.703
   47    208       1    0.664 0.01808        0.629        0.700
   49    199       3    0.654 0.01871        0.618        0.691
   53    163       6    0.630 0.02044        0.591        0.671
   56    134       3    0.616 0.02154        0.575        0.659
   57    110       1    0.610 0.02206        0.568        0.655
   60    108       4    0.587 0.02396        0.542        0.636
   62     94       1    0.581 0.02451        0.535        0.631
   63     90       3    0.562 0.02612        0.513        0.615
   67     54       2    0.541 0.02900        0.487        0.601
   68     46       1    0.529 0.03066        0.472        0.593
   70     19       5    0.390 0.05804        0.291        0.522

Call: survfit(formula = Surv(Date.dpi, as.numeric(Ruffledness)) ~ Status,
    data = aData)

1 observation deleted due to missingness
                Status=I
 time n.risk n.event survival std.err lower 95% CI upper 95% CI
    0   2808      30    0.989 0.00194        0.986        0.993
    1   2666      31    0.978 0.00281        0.972        0.983
    2   2559      38    0.963 0.00362        0.956        0.970
    3   2419      30    0.951 0.00418        0.943        0.960
    4   2286      32    0.938 0.00474        0.929        0.947
    5   2164      34    0.923 0.00530        0.913        0.934
    6   2053      37    0.907 0.00587        0.895        0.918
    7   1925      36    0.890 0.00640        0.877        0.902
    8   1810      36    0.872 0.00692        0.859        0.886
    9   1668      41    0.851 0.00752        0.836        0.865
   10   1510      35    0.831 0.00805        0.815        0.847
   11   1379      30    0.813 0.00852        0.796        0.830
   12   1266      34    0.791 0.00908        0.773        0.809
   13   1164      25    0.774 0.00950        0.756        0.793
   14   1057      12    0.765 0.00972        0.746        0.784
   15    973       5    0.761 0.00983        0.742        0.781
   16    913       1    0.760 0.00985        0.741        0.780
   17    879       1    0.760 0.00988        0.740        0.779
   18    849      13    0.748 0.01024        0.728        0.768
```

```
   19    802        5    0.743 0.01039        0.723        0.764
   20    775        6    0.737 0.01057        0.717        0.759
   21    756        5    0.733 0.01072        0.712        0.754
   22    700        5    0.727 0.01090        0.706        0.749
   24    654        3    0.724 0.01102        0.703        0.746
   25    635       10    0.713 0.01142        0.691        0.735
   26    598        8    0.703 0.01175        0.680        0.727
   28    551        2    0.701 0.01185        0.678        0.724
   32    512        9    0.688 0.01233        0.664        0.713
   36    429        1    0.687 0.01241        0.663        0.711
   37    424        1    0.685 0.01248        0.661        0.710
   39    420       10    0.669 0.01321        0.643        0.695
   42    364        1    0.667 0.01330        0.641        0.693
   46    318        6    0.654 0.01400        0.627        0.682
   47    299        1    0.652 0.01413        0.625        0.680
   49    288        2    0.648 0.01439        0.620        0.676
   53    235        5    0.634 0.01534        0.604        0.665
   56    189        2    0.627 0.01590        0.597        0.659
   57    158        1    0.623 0.01629        0.592        0.656
   60    154        4    0.607 0.01776        0.573        0.643
   62    134        1    0.602 0.01820        0.568        0.639
   63    129        3    0.588 0.01949        0.551        0.628
   67     76        2    0.573 0.02183        0.532        0.617
   68     62        1    0.564 0.02336        0.520        0.611
   70     23        7    0.392 0.05647        0.296        0.520

                Status=U 
 time n.risk n.event survival std.err lower 95% CI upper 95% CI
    0   1391        3    0.998 0.00124        0.995        1.000
    1   1336        4    0.995 0.00194        0.991        0.999
    2   1271        3    0.993 0.00236        0.988        0.997
    3   1197        2    0.991 0.00263        0.986        0.996
    4   1124        3    0.988 0.00304        0.982        0.994
    5   1053        2    0.986 0.00331        0.980        0.993
    6    983        4    0.982 0.00386        0.975        0.990
    7    910        4    0.978 0.00440        0.969        0.987
    8    842        4    0.973 0.00496        0.964        0.983
    9    772        4    0.968 0.00553        0.958        0.979
   10    701        6    0.960 0.00644        0.947        0.973
   11    639        6    0.951 0.00736        0.937        0.966
   12    581        4    0.944 0.00800        0.929        0.960
   13    523        3    0.939 0.00854        0.922        0.956
   14    472        1    0.937 0.00875        0.920        0.954
   15    432        1    0.935 0.00900        0.917        0.953
   25    284        1    0.932 0.00955        0.913        0.950
   26    273        2    0.925 0.01063        0.904        0.946
   35    193        1    0.920 0.01160        0.898        0.943
   39    167        2    0.909 0.01384        0.882        0.936
   42    151        1    0.903 0.01500        0.874        0.933
   49    116        1    0.895 0.01677        0.863        0.929
   53     95        1    0.886 0.01905        0.849        0.924
   56     82        1    0.875 0.02167        0.833        0.918
   60     65        1    0.861 0.02517        0.814        0.912

Call: survfit(formula = Surv(Date.dpi, as.numeric(Ruffledness)) ~ Strain, 
    data = aData)

1 observation deleted due to missingness 
                Strain=CC002 
 time n.risk n.event survival std.err lower 95% CI upper 95% CI
```

1	274	1	0.996	0.00364	0.989	1
7	178	1	0.991	0.00665	0.978	1
9	148	2	0.977	0.01147	0.955	1
36	54	1	0.959	0.02117	0.919	1

Strain=CC005

time	n.risk	n.event	survival	std.err	lower 95% CI	upper 95% CI
0	212	1	0.995	0.00471	0.986	1.000
1	206	2	0.986	0.00824	0.970	1.000
2	198	3	0.971	0.01179	0.948	0.994
3	186	2	0.960	0.01379	0.934	0.988
4	174	6	0.927	0.01880	0.891	0.965
5	162	4	0.904	0.02154	0.863	0.947
6	150	6	0.868	0.02524	0.820	0.919
7	138	6	0.830	0.02846	0.776	0.888
8	126	6	0.791	0.03135	0.732	0.855
9	114	5	0.756	0.03359	0.693	0.825
10	102	7	0.704	0.03657	0.636	0.780
11	90	7	0.649	0.03915	0.577	0.731
12	78	5	0.608	0.04083	0.533	0.693
13	66	4	0.571	0.04230	0.494	0.660
14	56	3	0.540	0.04357	0.461	0.633
19	48	1	0.529	0.04409	0.449	0.623
21	46	2	0.506	0.04507	0.425	0.603
26	42	1	0.494	0.04558	0.412	0.592
28	40	2	0.469	0.04653	0.386	0.570
42	28	2	0.436	0.04888	0.350	0.543
49	22	2	0.396	0.05184	0.307	0.512
56	16	2	0.347	0.05596	0.253	0.476
63	10	1	0.312	0.06015	0.214	0.455
70	4	1	0.234	0.08123	0.119	0.462

Strain=CC006

time	n.risk	n.event	survival	std.err	lower 95% CI	upper 95% CI
0	262	3	0.989	0.00657	0.976	1.000
1	250	3	0.977	0.00941	0.958	0.995
2	234	4	0.960	0.01241	0.936	0.985
3	218	1	0.956	0.01311	0.930	0.982
4	202	2	0.946	0.01459	0.918	0.975
6	170	2	0.935	0.01640	0.903	0.968
7	154	2	0.923	0.01830	0.888	0.959
8	138	1	0.916	0.01935	0.879	0.955
18	42	1	0.894	0.02866	0.840	0.952
19	40	1	0.872	0.03561	0.805	0.945

Strain=CC011

time	n.risk	n.event	survival	std.err	lower 95% CI	upper 95% CI
0	247	2	0.992	0.00570	0.981	1.000
1	240	4	0.975	0.00993	0.956	0.995
2	230	7	0.946	0.01466	0.917	0.975
3	216	7	0.915	0.01819	0.880	0.951
4	202	3	0.901	0.01954	0.864	0.941
5	187	4	0.882	0.02137	0.841	0.925
6	173	2	0.872	0.02230	0.829	0.917
7	159	3	0.856	0.02382	0.810	0.904
8	145	1	0.850	0.02438	0.803	0.899
9	131	2	0.837	0.02567	0.788	0.889
12	89	1	0.827	0.02705	0.776	0.882
19	56	1	0.812	0.03033	0.755	0.874
35	39	1	0.792	0.03601	0.724	0.865

Strain=CC012

time	n.risk	n.event	survival	std.err	lower 95% CI	upper 95% CI
0	468	15	0.968	0.00814	0.9521	0.984
1	444	12	0.942	0.01087	0.9207	0.963
2	420	10	0.919	0.01272	0.8948	0.945
3	396	16	0.882	0.01522	0.8529	0.913
4	372	14	0.849	0.01704	0.8163	0.883
5	348	19	0.803	0.01914	0.7660	0.841
6	324	22	0.748	0.02108	0.7080	0.791
7	300	20	0.698	0.02243	0.6557	0.744
8	276	20	0.648	0.02348	0.6033	0.695
9	252	19	0.599	0.02424	0.5532	0.648
10	228	18	0.552	0.02476	0.5051	0.602
11	204	15	0.511	0.02505	0.4642	0.563
12	180	21	0.451	0.02528	0.4045	0.504
13	156	14	0.411	0.02523	0.3643	0.463
14	132	6	0.392	0.02520	0.3458	0.445
15	120	6	0.373	0.02518	0.3264	0.425
18	108	8	0.345	0.02514	0.2991	0.398
25	96	8	0.316	0.02501	0.2708	0.369
26	83	8	0.286	0.02482	0.2410	0.339
32	71	8	0.254	0.02449	0.2098	0.306
39	59	11	0.206	0.02371	0.1647	0.258
46	47	4	0.189	0.02326	0.1482	0.240
53	35	5	0.162	0.02285	0.1226	0.213
60	24	5	0.128	0.02252	0.0907	0.181
67	12	2	0.107	0.02328	0.0696	0.164

Strain=CC012XCC032

time	n.risk	n.event	survival	std.err	lower 95% CI	upper 95% CI
1	194	1	0.995	0.00514	0.9848	1
70	5	4	0.199	0.17797	0.0345	1

Strain=CC013XCC041

time	n.risk	n.event	survival	std.err	lower 95% CI	upper 95% CI
70.000	5.000	2.000	0.600	0.219	0.293	1.000

Strain=CC015

time	n.risk	n.event	survival	std.err	lower 95% CI	upper 95% CI
0.0000	35.0000	1.0000	0.9714	0.0282	0.9178	1.0000

Strain=CC017

time	n.risk	n.event	survival	std.err	lower 95% CI	upper 95% CI
4	90	2	0.978	0.0155	0.948	1.000
5	84	2	0.954	0.0222	0.912	0.999
8	66	1	0.940	0.0262	0.890	0.993
9	60	1	0.924	0.0301	0.867	0.985
10	54	1	0.907	0.0340	0.843	0.976
11	48	1	0.888	0.0382	0.817	0.967
12	42	1	0.867	0.0428	0.787	0.955
13	36	1	0.843	0.0479	0.754	0.942

Strain=CC023

time	n.risk	n.event	survival	std.err	lower 95% CI	upper 95% CI
0	208	4	0.981	0.00952	0.962	1.000

```
    1    200       4    0.961 0.01347        0.935        0.988
    2    188       4    0.941 0.01661        0.909        0.974
    3    176       2    0.930 0.01806        0.895        0.966
    4    166       3    0.913 0.02018        0.875        0.954
    5    154       2    0.901 0.02159        0.860        0.945
    6    142       4    0.876 0.02443        0.829        0.925
    7    130       4    0.849 0.02714        0.797        0.904
    8    118       5    0.813 0.03039        0.756        0.875
    9    106       5    0.775 0.03345        0.712        0.843
   10     94       6    0.725 0.03690        0.656        0.801
   11     82       5    0.681 0.03960        0.608        0.763
   12     70       4    0.642 0.04184        0.565        0.730
   13     58       2    0.620 0.04323        0.541        0.711
   18     38       1    0.604 0.04507        0.521        0.699
   46     18       1    0.570 0.05361        0.474        0.685
   47     17       1    0.537 0.06003        0.431        0.668
   63      8       1    0.469 0.08183        0.334        0.661

                Strain=CC025
 time n.risk n.event survival std.err lower 95% CI upper 95% CI
    0    173       2    0.988 0.00813        0.973        1.000
    1    164       5    0.958 0.01543        0.929        0.989
    2    154       4    0.933 0.01941        0.896        0.972
    3    144       3    0.914 0.02202        0.872        0.958
    4    135       4    0.887 0.02519        0.839        0.938
    5    125       4    0.859 0.02809        0.805        0.915
    6    115       4    0.829 0.03083        0.770        0.891
    7    105       4    0.797 0.03345        0.734        0.865
    8     95       4    0.764 0.03601        0.696        0.837
    9     85       4    0.728 0.03854        0.656        0.807
   10     75       2    0.708 0.03988        0.634        0.791
   11     73       2    0.689 0.04108        0.613        0.774
   12     71       2    0.669 0.04215        0.592        0.757
   13     69       2    0.650 0.04310        0.571        0.740
   18     67       1    0.640 0.04354        0.560        0.732
   25     66       1    0.631 0.04394        0.550        0.723
   32     53       1    0.619 0.04470        0.537        0.713
   37     36       1    0.601 0.04664        0.517        0.700
   39     32       1    0.583 0.04882        0.494        0.687
   46     11       1    0.530 0.06724        0.413        0.679
   53      6       1    0.441 0.09816        0.285        0.683
   57      5       1    0.353 0.11137        0.190        0.655
   62      4       1    0.265 0.11324        0.115        0.612

                Strain=CC027
     time n.risk n.event survival std.err lower 95% CI upper 95% CI

                Strain=CC032XCC013
        time        n.risk      n.event     survival      std.err lower 95% CI upper
95% CI
    2.00e+00     2.29e+02     5.00e+00     9.78e-01     9.66e-03     9.59e-01
9.97e-01

                Strain=CC037
 time n.risk n.event survival std.err lower 95% CI upper 95% CI
    0    202       1    0.995 0.00494        0.985            1
    1    192       1    0.990 0.00713        0.976            1
    2    180       1    0.984 0.00896        0.967            1
   18     34       1    0.955 0.02982        0.899            1
```

```
                Strain=CC041XCC012 
 time n.risk n.event survival std.err lower 95% CI upper 95% CI
    2    281       1    0.996 0.00355        0.990        1.000
    3    259       1    0.993 0.00522        0.982        1.000
    4    237       1    0.988 0.00667        0.975        1.000
    5    225       1    0.984 0.00796        0.969        1.000
    6    214       1    0.979 0.00915        0.962        0.998
    8    193       1    0.974 0.01042        0.954        0.995
   13    149       1    0.968 0.01223        0.944        0.992
   21    128       1    0.960 0.01428        0.933        0.989

                Strain=CC051 
 time n.risk n.event survival std.err lower 95% CI upper 95% CI
    0    146       4    0.973  0.0135        0.946        0.999
    1    138       2    0.959  0.0166        0.927        0.992
    2    128       2    0.944  0.0194        0.906        0.982
   68      2       1    0.472  0.3337        0.118        1.000

                Strain=CC057 
 time n.risk n.event survival std.err lower 95% CI upper 95% CI
   11    300       1    0.997 0.00333        0.990        1.000
   20    152       2    0.984 0.00978        0.965        1.000
   21    136       2    0.969 0.01400        0.942        0.997
   22    120       2    0.953 0.01782        0.919        0.989
   24     88       1    0.942 0.02065        0.902        0.983
   25     72       1    0.929 0.02416        0.883        0.978

                Strain=CC078 
 time n.risk n.event survival std.err lower 95% CI upper 95% CI
    8    245       1    0.996 0.00407        0.988        1.000
    9    231       7    0.966 0.01191        0.943        0.989
   10    217       7    0.935 0.01634        0.903        0.967
   11    203       5    0.912 0.01890        0.875        0.949
   12    190       4    0.892 0.02080        0.853        0.934
   13    178       4    0.872 0.02262        0.829        0.918
   14    166       4    0.851 0.02439        0.805        0.900
   16    142       1    0.845 0.02495        0.798        0.896
   17    130       1    0.839 0.02559        0.790        0.890
   18    119       1    0.832 0.02633        0.782        0.885
   19    109       2    0.816 0.02797        0.763        0.873
   20     99       4    0.784 0.03133        0.724        0.847
   22     79       3    0.754 0.03453        0.689        0.825
   24     59       2    0.728 0.03779        0.658        0.806
   25     49       1    0.713 0.03983        0.639        0.796
   26     39       1    0.695 0.04281        0.616        0.784
   49     20       1    0.660 0.05292        0.564        0.773
   56     15       1    0.616 0.06518        0.501        0.758
   63     10       1    0.555 0.08282        0.414        0.743

Call:
coxph(formula = Surv(Date.dpi, as.numeric(Ruffledness)) ~ Sex, 
    data = aData)

  n= 4199, number of events= 667 
   (1 observation deleted due to missingness)

        coef exp(coef) se(coef)     z Pr(>|z|)    
SexM 0.30901   1.36208  0.07794 3.965 7.34e-05 ***
---
Signif. codes:  0 ‚Äò***‚Äô 0.001 ‚Äò**‚Äô 0.01 ‚Äò*‚Äô 0.05 ‚Äò.‚Äô 0.1 ‚Äò ‚Äô 1
```

```
     exp(coef) exp(-coef) lower .95 upper .95
SexM     1.362     0.7342     1.169     1.587

Concordance= 0.508  (se = 0.011 )
Likelihood ratio test= 15.84  on 1 df,   p=7e-05
Wald test            = 15.72  on 1 df,   p=7e-05
Score (logrank) test = 15.85  on 1 df,   p=7e-05

Call:
coxph(formula = Surv(Date.dpi, as.numeric(Ruffledness)) ~ Status,
    data = aData)

  n= 4199, number of events= 667
   (1 observation deleted due to missingness)

            coef exp(coef) se(coef)      z Pr(>|z|)
StatusU -1.4776    0.2282   0.1306 -11.31   <2e-16 ***
---
Signif. codes:  0 ‚Äò***‚Äô 0.001 ‚Äò**‚Äô 0.01 ‚Äò*‚Äô 0.05 ‚Äò.‚Äô 0.1 ‚Äò ‚Äô 1

        exp(coef) exp(-coef) lower .95 upper .95
StatusU    0.2282      4.382    0.1767    0.2947

Concordance= 0.616  (se = 0.007 )
Likelihood ratio test= 187.8  on 1 df,   p=<2e-16
Wald test            = 128  on 1 df,   p=<2e-16
Score (logrank) test = 153.1  on 1 df,   p=<2e-16

Call:
coxph(formula = Surv(Date.dpi, as.numeric(Ruffledness)) ~ Strain,
    data = Z)

  n= 4395, number of events= 676
   (1 observation deleted due to missingness)

                      coef  exp(coef)   se(coef)      z Pr(>|z|)
StrainCC078       1.555e+00  4.737e+00  3.618e-01  4.299 1.71e-05 ***
StrainCC057      -5.714e-01  5.647e-01  4.716e-01 -1.212  0.22565
StrainCC012       3.135e+00  2.300e+01  3.386e-01  9.261  < 2e-16 ***
StrainCC041XCC012 -3.608e-01  6.971e-01  4.862e-01 -0.742  0.45807
StrainCC002      -6.122e-01  5.421e-01  5.580e-01 -1.097  0.27253
StrainCC025       2.311e+00  1.009e+01  3.597e-01  6.426 1.31e-10 ***
StrainCC012XCC032 -4.177e-01  6.586e-01  5.580e-01 -0.749  0.45414
StrainCC013XCC041 -1.173e+00  3.095e-01  7.820e-01 -1.500  0.13367
StrainCC032XCC013 -5.117e-01  5.995e-01  5.580e-01 -0.917  0.35913
StrainCC015       1.049e-02  1.011e+00  1.054e+00  0.010  0.99206
StrainCC023       2.287e+00  9.848e+00  3.590e-01  6.371 1.87e-10 ***
StrainCC027      -1.481e+01  3.704e-07  6.113e+02 -0.024  0.98067
StrainCC005       2.526e+00  1.250e+01  3.520e-01  7.177 7.14e-13 ***
StrainCC011       1.616e+00  5.031e+00  3.713e-01  4.351 1.35e-05 ***
StrainCC051       1.049e+00  2.855e+00  4.720e-01  2.222  0.02627 *
StrainCC037      -2.607e-01  7.705e-01  6.012e-01 -0.434  0.66457
StrainCC006       1.062e+00  2.893e+00  4.019e-01  2.643  0.00821 **
StrainCC017       1.051e+00  2.861e+00  4.602e-01  2.285  0.02233 *
---
Signif. codes:  0 ‚Äò***‚Äô 0.001 ‚Äò**‚Äô 0.01 ‚Äò*‚Äô 0.05 ‚Äò.‚Äô 0.1 ‚Äò ‚Äô 1

                  exp(coef) exp(-coef) lower .95 upper .95
StrainCC078       4.737e+00  2.111e-01   2.33114     9.627
```

StrainCC057	5.647e-01	1.771e+00	0.22409	1.423
StrainCC012	2.300e+01	4.348e-02	11.84412	44.654
StrainCC041XCC012	6.971e-01	1.434e+00	0.26880	1.808
StrainCC002	5.421e-01	1.845e+00	0.18162	1.618
StrainCC025	1.009e+01	9.911e-02	4.98540	20.419
StrainCC012XCC032	6.586e-01	1.518e+00	0.22059	1.966
StrainCC013XCC041	3.095e-01	3.231e+00	0.06684	1.433
StrainCC032XCC013	5.995e-01	1.668e+00	0.20081	1.790
StrainCC015	1.011e+00	9.896e-01	0.12800	7.978
StrainCC023	9.848e+00	1.015e-01	4.87271	19.903
StrainCC027	3.704e-07	2.700e+06	0.00000	Inf
StrainCC005	1.250e+01	7.997e-02	6.27259	24.926
StrainCC011	5.031e+00	1.988e-01	2.42999	10.415
StrainCC051	2.855e+00	3.503e-01	1.13180	7.200
StrainCC037	7.705e-01	1.298e+00	0.23715	2.503
StrainCC006	2.893e+00	3.456e-01	1.31600	6.361
StrainCC017	2.861e+00	3.495e-01	1.16116	7.051

```
Concordance= 0.831  (se = 0.007 )
Likelihood ratio test= 1083  on 18 df,   p=<2e-16
Wald test            = 673  on 18 df,   p=<2e-16
Score (logrank) test = 1396  on 18 df,   p=<2e-16
```

SEIZURE

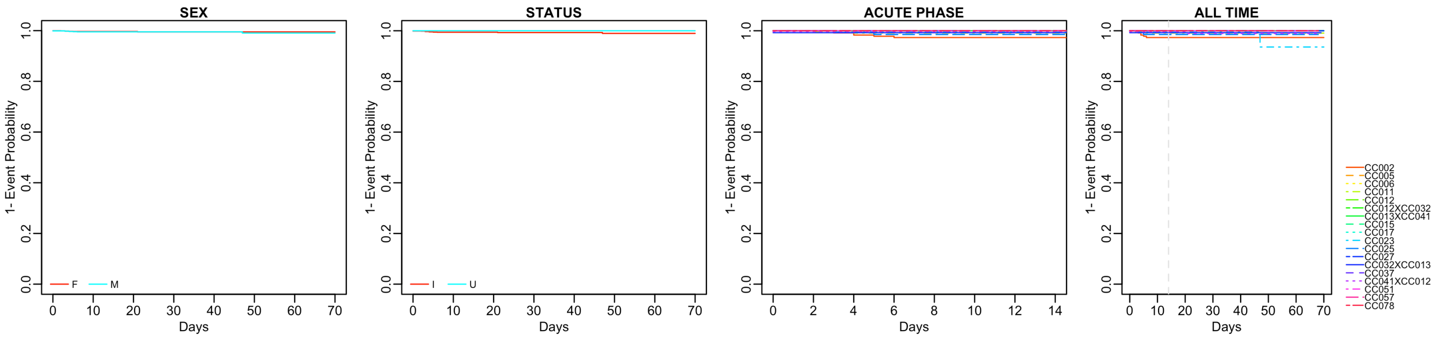


```
Call: survfit(formula = Surv(Date.dpi, as.numeric(Seizure)) ~ Sex,
    data = aData)

                Sex=F
 time n.risk n.event survival  std.err lower 95% CI upper 95% CI
    0   2190       1    1.000 0.000457        0.999        1.000
    2   1993       1    0.999 0.000678        0.998        1.000
    3   1883       2    0.998 0.001010        0.996        1.000
    4   1778       1    0.997 0.001155        0.995        1.000
    5   1678       1    0.997 0.001299        0.994        0.999
   21    580       1    0.995 0.002152        0.991        0.999

                Sex=M
 time n.risk n.event survival  std.err lower 95% CI upper 95% CI
    0   2010       2    0.999 0.000703        0.998        1.000
    3   1734       2    0.998 0.001075        0.996        1.000
    4   1633       2    0.997 0.001378        0.994        0.999
    5   1540       1    0.996 0.001522        0.993        0.999
    6   1454       1    0.995 0.001668        0.992        0.999
   47    208       1    0.991 0.005054        0.981        1.000

Call: survfit(formula = Surv(Date.dpi, as.numeric(Seizure)) ~ Status,
    data = aData)

                Status=I
 time n.risk n.event survival  std.err lower 95% CI upper 95% CI
    0   2809       3    0.999 0.000616        0.998        1.000
    2   2560       1    0.999 0.000729        0.997        1.000
    3   2420       4    0.997 0.001100        0.995        0.999
    4   2287       3    0.996 0.001333        0.993        0.998
    5   2165       2    0.995 0.001482        0.992        0.998
    6   2054       1    0.994 0.001558        0.991        0.997
   21    756       1    0.993 0.002037        0.989        0.997
   47    299       1    0.990 0.003887        0.982        0.997

                Status=U
     time n.risk n.event survival std.err lower 95% CI upper 95% CI

Call: survfit(formula = Surv(Date.dpi, as.numeric(Seizure)) ~ Strain,
    data = aData)

                Strain=CC002
 time n.risk n.event survival std.err lower 95% CI upper 95% CI
    3    242       2    0.992 0.00582        0.980        1.000
    4    226       2    0.983 0.00845        0.967        1.000
    5    210       1    0.978 0.00962        0.960        0.997
    6    194       1    0.973 0.01081        0.952        0.995

                Strain=CC005
```

```
     time n.risk n.event survival std.err lower 95% CI upper 95% CI

                Strain=CC006
     time n.risk n.event survival std.err lower 95% CI upper 95% CI

                Strain=CC011
 time n.risk n.event survival std.err lower 95% CI upper 95% CI
    2    230       1    0.996 0.00434        0.987            1
    4    202       1    0.991 0.00654        0.978            1

                Strain=CC012
     time n.risk n.event survival std.err lower 95% CI upper 95% CI

                Strain=CC012XCC032
     time n.risk n.event survival std.err lower 95% CI upper 95% CI

                Strain=CC013XCC041
     time n.risk n.event survival std.err lower 95% CI upper 95% CI

                Strain=CC015
     time n.risk n.event survival std.err lower 95% CI upper 95% CI

                Strain=CC017
     time n.risk n.event survival std.err lower 95% CI upper 95% CI

                Strain=CC023
 time n.risk n.event survival std.err lower 95% CI upper 95% CI
    3    177       1    0.994 0.00563        0.983            1
   47     17       1    0.936 0.05699        0.831            1

                Strain=CC025
 time n.risk n.event survival std.err lower 95% CI upper 95% CI
    3    144       1    0.993 0.00692        0.980            1
    5    125       1    0.985 0.01048        0.965            1

                Strain=CC027
     time n.risk n.event survival std.err lower 95% CI upper 95% CI

                Strain=CC032XCC013
        time       n.risk      n.event     survival      std.err lower 95% CI upper
95% CI
    0.00e+00     2.62e+02     2.00e+00     9.92e-01     5.38e-03     9.82e-01
1.00e+00

                Strain=CC037
     time n.risk n.event survival std.err lower 95% CI upper 95% CI

                Strain=CC041XCC012
 time n.risk n.event survival std.err lower 95% CI upper 95% CI
    0    325       1    0.997 0.00307        0.991            1
   21    128       1    0.989 0.00834        0.973            1

                Strain=CC051
     time n.risk n.event survival std.err lower 95% CI upper 95% CI

                Strain=CC057
     time n.risk n.event survival std.err lower 95% CI upper 95% CI

                Strain=CC078
     time n.risk n.event survival std.err lower 95% CI upper 95% CI
```

```
Call:
coxph(formula = Surv(Date.dpi, as.numeric(Seizure)) ~ Sex, data = aData)

  n= 4200, number of events= 16

       coef exp(coef) se(coef)     z Pr(>|z|)
SexM 0.3332    1.3954   0.5040 0.661    0.509

     exp(coef) exp(-coef) lower .95 upper .95
SexM     1.395     0.7167    0.5197     3.747

Concordance= 0.54  (se = 0.065 )
Likelihood ratio test= 0.44  on 1 df,   p=0.5
Wald test            = 0.44  on 1 df,   p=0.5
Score (logrank) test = 0.44  on 1 df,   p=0.5

Call:
coxph(formula = Surv(Date.dpi, as.numeric(Seizure)) ~ Status,
    data = aData)

  n= 4200, number of events= 16

              coef  exp(coef)   se(coef)      z Pr(>|z|)
StatusU -1.865e+01  7.935e-09  4.029e+03 -0.005    0.996

        exp(coef) exp(-coef) lower .95 upper .95
StatusU 7.935e-09   1.26e+08         0       Inf

Concordance= 0.665  (se = 0.004 )
Likelihood ratio test= 12.65  on 1 df,   p=4e-04
Wald test            = 0  on 1 df,   p=1
Score (logrank) test = 7.76  on 1 df,   p=0.005

Call:
coxph(formula = Surv(Date.dpi, as.numeric(Seizure)) ~ Strain,
    data = Z)

  n= 4380, number of events= 4380
   (16 observations deleted due to missingness)

                     coef exp(coef) se(coef)     z Pr(>|z|)
StrainCC078       0.14488   1.15590  0.09496 1.526 0.127064
StrainCC057       0.16177   1.17559  0.08793 1.840 0.065791 .
StrainCC012       0.50378   1.65497  0.08536 5.902 3.59e-09 ***
StrainCC041XCC012 0.17147   1.18705  0.09099 1.885 0.059484 .
StrainCC002       0.32510   1.38417  0.09345 3.479 0.000503 ***
StrainCC025       0.34130   1.40677  0.10471 3.260 0.001116 **
StrainCC012XCC032 0.16048   1.17407  0.10050 1.597 0.110293
StrainCC013XCC041 0.14414   1.15505  0.10536 1.368 0.171282
StrainCC032XCC013 0.30802   1.36072  0.09496 3.244 0.001180 **
StrainCC015       0.53642   1.70988  0.18364 2.921 0.003488 **
StrainCC023       0.52175   1.68498  0.10010 5.213 1.86e-07 ***
StrainCC027       0.48865   1.63011  0.09613 5.083 3.71e-07 ***
StrainCC005       0.40825   1.50418  0.09949 4.103 4.07e-05 ***
StrainCC011       0.41579   1.51557  0.09622 4.321 1.55e-05 ***
StrainCC051       0.85491   2.35116  0.10981 7.785 6.94e-15 ***
StrainCC037       0.65390   1.92302  0.10060 6.500 8.03e-11 ***
StrainCC006       0.61482   1.84932  0.09485 6.482 9.07e-11 ***
StrainCC017       0.41187   1.50963  0.11853 3.475 0.000511 ***
```

```
---
Signif. codes:  0 ‚Äò***‚Äô 0.001 ‚Äò**‚Äô 0.01 ‚Äò*‚Äô 0.05 ‚Äò.‚Äô 0.1 ‚Äò ‚Äô 1

                  exp(coef) exp(-coef) lower .95 upper .95
StrainCC078           1.156     0.8651    0.9596     1.392
StrainCC057           1.176     0.8506    0.9895     1.397
StrainCC012           1.655     0.6042    1.4000     1.956
StrainCC041XCC012     1.187     0.8424    0.9932     1.419
StrainCC002           1.384     0.7225    1.1525     1.662
StrainCC025           1.407     0.7108    1.1458     1.727
StrainCC012XCC032     1.174     0.8517    0.9642     1.430
StrainCC013XCC041     1.155     0.8658    0.9395     1.420
StrainCC032XCC013     1.361     0.7349    1.1296     1.639
StrainCC015           1.710     0.5848    1.1930     2.451
StrainCC023           1.685     0.5935    1.3848     2.050
StrainCC027           1.630     0.6135    1.3502     1.968
StrainCC005           1.504     0.6648    1.2377     1.828
StrainCC011           1.516     0.6598    1.2551     1.830
StrainCC051           2.351     0.4253    1.8959     2.916
StrainCC037           1.923     0.5200    1.5789     2.342
StrainCC006           1.849     0.5407    1.5356     2.227
StrainCC017           1.510     0.6624    1.1967     1.904

Concordance= 0.583  (se = 0.005 )
Likelihood ratio test= 166.5  on 18 df,   p=<2e-16
Wald test            = 169.9  on 18 df,   p=<2e-16
Score (logrank) test = 172.8  on 18 df,   p=<2e-16
```

File S2. Statistics for all DigiGait parameters evaluated. Labels indicate which limb is associated with the data, where appropriate: FL for left fore limb, FR for right fore limb, HL for left hind limb, and HR for right hind limb.

```
Response SwingDurationCV_RF :

Call:
lm(formula = SwingDurationCV_RF ~ clusterID)

Residuals:
CC041XCC012       CC012 CC032XCC013       CC017       CC023
CC012XCC032       CC057
  -0.512021   -0.164674    0.676695   -0.315628   -0.009632
0.254359    0.070901

Coefficients:
            Estimate Std. Error t value Pr(>|t|)
(Intercept)  -1.4654     0.2472  -5.928 0.001949 **
clusterID2    2.7815     0.3270   8.505 0.000369 ***
---
Signif. codes:  0 ë***í 0.001 ë**í 0.01 ë*í 0.05 ë.í 0.1 ë í 1

Residual standard error: 0.4282 on 5 degrees of freedom
Multiple R-squared:  0.9354,     Adjusted R-squared:  0.9224
F-statistic: 72.34 on 1 and 5 DF,  p-value: 0.0003694


Response Propel_RF :

Call:
lm(formula = Propel_RF ~ clusterID)

Residuals:
CC041XCC012       CC012 CC032XCC013       CC017       CC023
CC012XCC032       CC057
   -0.14632     0.10126     0.04506     0.64087    -0.08862
0.09587    -0.64812

Coefficients:
            Estimate Std. Error t value Pr(>|t|)
(Intercept)   1.3960     0.2424   5.759 0.002217 **
clusterID2   -2.7133     0.3207  -8.461 0.000379 ***
---
Signif. codes:  0 ë***í 0.001 ë**í 0.01 ë*í 0.05 ë.í 0.1 ë í 1

Residual standard error: 0.4199 on 5 degrees of freedom
Multiple R-squared:  0.9347,     Adjusted R-squared:  0.9217
F-statistic: 71.58 on 1 and 5 DF,  p-value: 0.0003788
```

```
Response MINdAratiodT_RF :

Call:
lm(formula = MINdAratiodT_RF ~ clusterID)

Residuals:
CC041XCC012       CC012 CC032XCC013       CC017       CC023
CC012XCC032       CC057
   -0.23201    -0.01674     0.24875     0.10101    -0.63409
-0.04312     0.57620

Coefficients:
            Estimate Std. Error t value Pr(>|t|)
(Intercept)   1.1550     0.2397   4.818 0.004808 **
clusterID2   -2.2391     0.3171  -7.060 0.000881 ***
---
Signif. codes:  0 ë***í 0.001 ë**í 0.01 ë*í 0.05 ë.í 0.1 ë í 1

Residual standard error: 0.4152 on 5 degrees of freedom
Multiple R-squared:  0.9088,      Adjusted R-squared:  0.8906
F-statistic: 49.85 on 1 and 5 DF,  p-value: 0.0008812


Response Propel_LF :

Call:
lm(formula = Propel_LF ~ clusterID)

Residuals:
CC041XCC012       CC012 CC032XCC013       CC017       CC023
CC012XCC032       CC057
     0.3157     -0.0959     -0.2198      0.7421      0.2588
-0.1771     -0.8238

Coefficients:
            Estimate Std. Error t value Pr(>|t|)
(Intercept)   1.2760     0.3146   4.055  0.00977 **
clusterID2   -2.6416     0.4162  -6.347  0.00143 **
---
Signif. codes:  0 ë***í 0.001 ë**í 0.01 ë*í 0.05 ë.í 0.1 ë í 1

Residual standard error: 0.545 on 5 degrees of freedom
Multiple R-squared:  0.8896,      Adjusted R-squared:  0.8675
F-statistic: 40.28 on 1 and 5 DF,  p-value: 0.001434


Response SwingDurationCV_LH :

Call:
```

```
lm(formula = SwingDurationCV_LH ~ clusterID)

Residuals:
CC041XCC012       CC012 CC032XCC013       CC017       CC023
CC012XCC032       CC057
     0.1039     -0.5152      0.4114     -0.7224     -0.1505
0.5262      0.3467

Coefficients:
            Estimate Std. Error t value Pr(>|t|)
(Intercept)  -1.0993     0.3041  -3.615  0.01530 *
clusterID2    2.3040     0.4023   5.728  0.00227 **
---
Signif. codes:  0 ë***í 0.001 ë**í 0.01 ë*í 0.05 ë.í 0.1 ë í 1

Residual standard error: 0.5267 on 5 degrees of freedom
Multiple R-squared:  0.8677,     Adjusted R-squared:  0.8413
F-statistic: 32.81 on 1 and 5 DF,  p-value: 0.00227


Response Stance_LF :

Call:
lm(formula = Stance_LF ~ clusterID)

Residuals:
CC041XCC012       CC012 CC032XCC013       CC017       CC023
CC012XCC032       CC057
     0.3219     -0.6413      0.3194      0.9650     -0.3319
-0.3222     -0.3109

Coefficients:
            Estimate Std. Error t value Pr(>|t|)
(Intercept)   1.4131     0.3520   4.014  0.01018 *
clusterID2   -2.6572     0.4657  -5.706  0.00231 **
---
Signif. codes:  0 ë***í 0.001 ë**í 0.01 ë*í 0.05 ë.í 0.1 ë í 1

Residual standard error: 0.6097 on 5 degrees of freedom
Multiple R-squared:  0.8669,     Adjusted R-squared:  0.8403
F-statistic: 32.56 on 1 and 5 DF,  p-value: 0.002308


Response Stride_LF :

Call:
lm(formula = Stride_LF ~ clusterID)

Residuals:
CC041XCC012       CC012 CC032XCC013       CC017       CC023
```

```
CC012XCC032       CC057
     0.2215     -0.5824      0.3609      1.0299     -0.2869
-0.4475     -0.2955

Coefficients:
            Estimate Std. Error t value Pr(>|t|)
(Intercept)   1.4302     0.3605   3.967  0.01066 *
clusterID2   -2.6952     0.4769  -5.652  0.00241 **
---
Signif. codes:  0 ë***í 0.001 ë**í 0.01 ë*í 0.05 ë.í 0.1 ë í 1

Residual standard error: 0.6244 on 5 degrees of freedom
Multiple R-squared:  0.8647,     Adjusted R-squared:  0.8376
F-statistic: 31.94 on 1 and 5 DF,  p-value: 0.002408


Response StrideLength_LF :

Call:
lm(formula = StrideLength_LF ~ clusterID)

Residuals:
CC041XCC012       CC012 CC032XCC013       CC017       CC023
CC012XCC032       CC057
     0.2261     -0.6146      0.3884      0.9890     -0.2891
-0.4165     -0.2834

Coefficients:
            Estimate Std. Error t value Pr(>|t|)
(Intercept)   1.5257     0.3555   4.292  0.00777 **
clusterID2   -2.6383     0.4702  -5.611  0.00249 **
---
Signif. codes:  0 ë***í 0.001 ë**í 0.01 ë*í 0.05 ë.í 0.1 ë í 1

Residual standard error: 0.6157 on 5 degrees of freedom
Multiple R-squared:  0.8629,     Adjusted R-squared:  0.8355
F-statistic: 31.48 on 1 and 5 DF,  p-value: 0.002487


Response AtaxiaCoefficient_RH :

Call:
lm(formula = AtaxiaCoefficient_RH ~ clusterID)

Residuals:
CC041XCC012       CC012 CC032XCC013       CC017       CC023
CC012XCC032       CC057
   -0.08177     0.65688    -0.57512     0.13158     0.27123
0.33306    -0.73587
```

```
Coefficients:
            Estimate Std. Error t value Pr(>|t|)
(Intercept)  -1.0953     0.3175  -3.450  0.01824 *
clusterID2    2.3256     0.4200   5.537  0.00264 **
---
Signif. codes:  0 ë***í 0.001 ë**í 0.01 ë*í 0.05 ë.í 0.1 ë í 1

Residual standard error: 0.55 on 5 degrees of freedom
Multiple R-squared:  0.8598,      Adjusted R-squared:  0.8317
F-statistic: 30.66 on 1 and 5 DF,  p-value: 0.002637


Response Swing_LF :

Call:
lm(formula = Swing_LF ~ clusterID)

Residuals:
CC041XCC012       CC012 CC032XCC013       CC017       CC023
CC012XCC032       CC057
   0.001213   -0.392672    0.391458    0.994592   -0.147284
-0.608247   -0.239061

Coefficients:
            Estimate Std. Error t value Pr(>|t|)
(Intercept)   1.2833     0.3411   3.762   0.0131 *
clusterID2   -2.4075     0.4513  -5.335   0.0031 **
---
Signif. codes:  0 ë***í 0.001 ë**í 0.01 ë*í 0.05 ë.í 0.1 ë í 1

Residual standard error: 0.5908 on 5 degrees of freedom
Multiple R-squared:  0.8506,      Adjusted R-squared:  0.8207
F-statistic: 28.46 on 1 and 5 DF,  p-value: 0.003102


Response StrideLengthCV_RF :

Call:
lm(formula = StrideLengthCV_RF ~ clusterID)

Residuals:
CC041XCC012       CC012 CC032XCC013       CC017       CC023
CC012XCC032       CC057
  -0.661524   -0.008283    0.669808   -0.940475    0.192459
0.558876    0.189140

Coefficients:
            Estimate Std. Error t value Pr(>|t|)
(Intercept)  -1.2430     0.3791  -3.279   0.0220 *
clusterID2    2.5080     0.5015   5.001   0.0041 **
```

```
---
Signif. codes:  0 ë***í 0.001 ë**í 0.01 ë*í 0.05 ë.í 0.1 ë í 1

Residual standard error: 0.6567 on 5 degrees of freedom
Multiple R-squared:  0.8334,	Adjusted R-squared:    0.8 
F-statistic: 25.01 on 1 and 5 DF,  p-value: 0.004103


Response SwingDurationCV_LF :

Call:
lm(formula = SwingDurationCV_LF ~ clusterID)

Residuals:
CC041XCC012       CC012 CC032XCC013       CC017       CC023 
CC012XCC032       CC057 
     0.4665      0.1794     -0.6460     -0.6746      0.1738 
0.3292      0.1716 

Coefficients:
            Estimate Std. Error t value Pr(>|t|)   
(Intercept)  -1.2452     0.2933  -4.246  0.00812 **
clusterID2    1.9253     0.3880   4.962  0.00424 **
---
Signif. codes:  0 ë***í 0.001 ë**í 0.01 ë*í 0.05 ë.í 0.1 ë í 1

Residual standard error: 0.508 on 5 degrees of freedom
Multiple R-squared:  0.8312,	Adjusted R-squared:  0.7975 
F-statistic: 24.62 on 1 and 5 DF,  p-value: 0.00424


Response Stride_RF :

Call:
lm(formula = Stride_RF ~ clusterID)

Residuals:
CC041XCC012       CC012 CC032XCC013       CC017       CC023 
CC012XCC032       CC057 
     0.1807     -0.3205      0.1398      1.3342     -0.3209 
-0.5970     -0.4163 

Coefficients:
            Estimate Std. Error t value Pr(>|t|)   
(Intercept)   1.5042     0.4137   3.636  0.01497 * 
clusterID2   -2.6679     0.5473  -4.874  0.00458 **
---
Signif. codes:  0 ë***í 0.001 ë**í 0.01 ë*í 0.05 ë.í 0.1 ë í 1

Residual standard error: 0.7166 on 5 degrees of freedom
```

```
Multiple R-squared:  0.8261,	Adjusted R-squared:  0.7914 
F-statistic: 23.76 on 1 and 5 DF,  p-value: 0.004575


Response Stance_RF :

Call:
lm(formula = Stance_RF ~ clusterID)

Residuals:
CC041XCC012       CC012 CC032XCC013       CC017       CC023 
CC012XCC032       CC057 
    0.37638    -0.45779     0.08141     1.22423    -0.20981 
-0.57905    -0.43538 

Coefficients:
            Estimate Std. Error t value Pr(>|t|)  
(Intercept)   1.4090     0.4021   3.504  0.01721 * 
clusterID2   -2.5905     0.5320  -4.870  0.00459 **
---
Signif. codes:  0 ë***í 0.001 ë**í 0.01 ë*í 0.05 ë.í 0.1 ë í 1

Residual standard error: 0.6965 on 5 degrees of freedom
Multiple R-squared:  0.8259,	Adjusted R-squared:  0.791 
F-statistic: 23.71 on 1 and 5 DF,  p-value: 0.004594


Response StrideLengthCV_RH :

Call:
lm(formula = StrideLengthCV_RH ~ clusterID)

Residuals:
CC041XCC012       CC012 CC032XCC013       CC017       CC023 
CC012XCC032       CC057 
   -0.04345     0.56756    -0.52412    -0.02348     0.79287 
0.27408    -1.04347 

Coefficients:
            Estimate Std. Error t value Pr(>|t|)  
(Intercept)  -1.3196     0.3993  -3.305  0.02137 * 
clusterID2    2.5360     0.5283   4.801  0.00488 **
---
Signif. codes:  0 ë***í 0.001 ë**í 0.01 ë*í 0.05 ë.í 0.1 ë í 1

Residual standard error: 0.6916 on 5 degrees of freedom
Multiple R-squared:  0.8217,	Adjusted R-squared:  0.7861 
F-statistic: 23.05 on 1 and 5 DF,  p-value: 0.00488
```

```
Response StrideLength_RF :

Call:
lm(formula = StrideLength_RF ~ clusterID)

Residuals:
CC041XCC012       CC012 CC032XCC013       CC017       CC023
CC012XCC032       CC057
     0.1844     -0.3546      0.1702      1.3146     -0.3043
-0.5932     -0.4170

Coefficients:
            Estimate Std. Error t value Pr(>|t|)
(Intercept)   1.5494     0.4111   3.769  0.01304 *
clusterID2   -2.5726     0.5439  -4.730  0.00519 **
---
Signif. codes:  0 ë***í 0.001 ë**í 0.01 ë*í 0.05 ë.í 0.1 ë í 1

Residual standard error: 0.7121 on 5 degrees of freedom
Multiple R-squared:  0.8174,      Adjusted R-squared:  0.7808
F-statistic: 22.38 on 1 and 5 DF,  p-value: 0.005195


Response Stride_LH :

Call:
lm(formula = Stride_LH ~ clusterID)

Residuals:
CC041XCC012       CC012 CC032XCC013       CC017       CC023
CC012XCC032       CC057
     0.1519     -0.3381      0.1862      0.9294      0.5716
-0.5338     -0.9672

Coefficients:
            Estimate Std. Error t value Pr(>|t|)
(Intercept)   1.0811     0.4150   2.605  0.04794 *
clusterID2   -2.5632     0.5489  -4.669  0.00549 **
---
Signif. codes:  0 ë***í 0.001 ë**í 0.01 ë*í 0.05 ë.í 0.1 ë í 1

Residual standard error: 0.7187 on 5 degrees of freedom
Multiple R-squared:  0.8135,      Adjusted R-squared:  0.7762
F-statistic:  21.8 on 1 and 5 DF,  p-value: 0.005485


Response StrideFrequency_LF :

Call:
lm(formula = StrideFrequency_LF ~ clusterID)
```

```
Residuals:
CC041XCC012       CC012 CC032XCC013       CC017       CC023
CC012XCC032       CC057
     0.1238      0.5932     -0.7171     -1.0814      0.4237
0.5640      0.0937

Coefficients:
            Estimate Std. Error t value Pr(>|t|)
(Intercept)  -1.2781     0.4129  -3.095  0.02699 *
clusterID2    2.5280     0.5462   4.628  0.00569 **
---
Signif. codes:  0 ë***í 0.001 ë**í 0.01 ë*í 0.05 ë.í 0.1 ë í 1

Residual standard error: 0.7152 on 5 degrees of freedom
Multiple R-squared:  0.8107,      Adjusted R-squared:  0.7729
F-statistic: 21.42 on 1 and 5 DF,  p-value: 0.005694


Response MAXdAratiodT_LH :

Call:
lm(formula = MAXdAratiodT_LH ~ clusterID)

Residuals:
CC041XCC012       CC012 CC032XCC013       CC017       CC023
CC012XCC032       CC057
    -0.1369      0.3840     -0.2471      0.8849     -0.8068
0.2561     -0.3342

Coefficients:
            Estimate Std. Error t value Pr(>|t|)
(Intercept)   1.5042     0.3501   4.297  0.00774 **
clusterID2   -2.0845     0.4631  -4.501  0.00639 **
---
Signif. codes:  0 ë***í 0.001 ë**í 0.01 ë*í 0.05 ë.í 0.1 ë í 1

Residual standard error: 0.6064 on 5 degrees of freedom
Multiple R-squared:  0.802,       Adjusted R-squared:  0.7624
F-statistic: 20.26 on 1 and 5 DF,  p-value: 0.006395


Response PawAreaatPeakStanceinsq.cm_LH :

Call:
lm(formula = PawAreaatPeakStanceinsq.cm_LH ~ clusterID)

Residuals:
CC041XCC012       CC012 CC032XCC013       CC017       CC023
CC012XCC032       CC057
```

```
   -0.01997     0.25327    -0.23329     0.66269    -0.76808 
0.64282    -0.53744 

Coefficients:
            Estimate Std. Error t value Pr(>|t|)   
(Intercept)   1.4132     0.3512   4.024  0.01008 * 
clusterID2   -2.0895     0.4646  -4.497  0.00641 **
---
Signif. codes:  0 ë***í 0.001 ë**í 0.01 ë*í 0.05 ë.í 0.1 ë í 1

Residual standard error: 0.6083 on 5 degrees of freedom
Multiple R-squared:  0.8018,	Adjusted R-squared:  0.7622 
F-statistic: 20.23 on 1 and 5 DF,  p-value: 0.006415


Response Swing_LH :

Call:
lm(formula = Swing_LH ~ clusterID)

Residuals:
CC041XCC012       CC012 CC032XCC013       CC017       CC023 
CC012XCC032       CC057 
   -0.03321     0.13843    -0.10523     0.66047    -0.45955 
0.74427    -0.94519 

Coefficients:
            Estimate Std. Error t value Pr(>|t|)   
(Intercept)   1.0580     0.3765   2.810  0.03754 * 
clusterID2   -2.1859     0.4980  -4.389  0.00709 **
---
Signif. codes:  0 ë***í 0.001 ë**í 0.01 ë*í 0.05 ë.í 0.1 ë í 1

Residual standard error: 0.6521 on 5 degrees of freedom
Multiple R-squared:  0.7939,	Adjusted R-squared:  0.7527 
F-statistic: 19.26 on 1 and 5 DF,  p-value: 0.007094


Response StrideLengthCV_LF :

Call:
lm(formula = StrideLengthCV_LF ~ clusterID)

Residuals:
CC041XCC012       CC012 CC032XCC013       CC017       CC023 
CC012XCC032       CC057 
   -0.13956    -0.60526     0.74482    -1.09193     0.67520 
0.47194    -0.05521 

Coefficients:
```

```
            Estimate Std. Error t value Pr(>|t|)
(Intercept)  -1.5422     0.4332  -3.560  0.01621 *
clusterID2    2.5044     0.5730   4.371  0.00722 **
---
Signif. codes:  0 ë***í 0.001 ë**í 0.01 ë*í 0.05 ë.í 0.1 ë í 1

Residual standard error: 0.7503 on 5 degrees of freedom
Multiple R-squared:  0.7925,	Adjusted R-squared:  0.7511
F-statistic:  19.1 on 1 and 5 DF,  p-value: 0.007219


Response StrideLength_LH :

Call:
lm(formula = StrideLength_LH ~ clusterID)

Residuals:
CC041XCC012       CC012 CC032XCC013       CC017       CC023
CC012XCC032       CC057
     0.1896     -0.4055      0.2159      0.8940      0.5772
-0.5016     -0.9696

Coefficients:
            Estimate Std. Error t value Pr(>|t|)
(Intercept)   1.1588     0.4140   2.799  0.03804 *
clusterID2   -2.3625     0.5477  -4.313  0.00762 **
---
Signif. codes:  0 ë***í 0.001 ë**í 0.01 ë*í 0.05 ë.í 0.1 ë í 1

Residual standard error: 0.7171 on 5 degrees of freedom
Multiple R-squared:  0.7882,	Adjusted R-squared:  0.7458
F-statistic: 18.61 on 1 and 5 DF,  p-value: 0.007618


Response Swing_RF :

Call:
lm(formula = Swing_RF ~ clusterID)

Residuals:
CC041XCC012       CC012 CC032XCC013       CC017       CC023
CC012XCC032       CC057
   -0.33516     0.05563     0.27952     1.40223    -0.51047
-0.57614    -0.31562

Coefficients:
            Estimate Std. Error t value Pr(>|t|)
(Intercept)   1.4982     0.4360   3.436  0.01852 *
clusterID2   -2.4310     0.5768  -4.215  0.00837 **
---
```

```
Signif. codes:  0 ë***í 0.001 ë**í 0.01 ë*í 0.05 ë.í 0.1 ë í 1

Residual standard error: 0.7552 on 5 degrees of freedom
Multiple R-squared:  0.7803,	Adjusted R-squared:  0.7364 
F-statistic: 17.76 on 1 and 5 DF,  p-value: 0.008372


Response StrideLengthCV_LH :

Call:
lm(formula = StrideLengthCV_LH ~ clusterID)

Residuals:
CC041XCC012       CC012 CC032XCC013       CC017       CC023 
CC012XCC032       CC057 
     0.1460      0.3284     -0.4744     -0.4436     -1.0237 
0.6749      0.7924 

Coefficients:
            Estimate Std. Error t value Pr(>|t|)   
(Intercept)  -0.9214     0.4229  -2.179  0.08122 . 
clusterID2    2.2791     0.5594   4.074  0.00959 **
---
Signif. codes:  0 ë***í 0.001 ë**í 0.01 ë*í 0.05 ë.í 0.1 ë í 1

Residual standard error: 0.7324 on 5 degrees of freedom
Multiple R-squared:  0.7685,	Adjusted R-squared:  0.7222 
F-statistic:  16.6 on 1 and 5 DF,  p-value: 0.009595


Response AtaxiaCoefficient_RF :

Call:
lm(formula = AtaxiaCoefficient_RF ~ clusterID)

Residuals:
CC041XCC012       CC012 CC032XCC013       CC017       CC023 
CC012XCC032       CC057 
    -0.6449      0.4005      0.2444     -1.1753      0.3477 
0.3146      0.5129 

Coefficients:
            Estimate Std. Error t value Pr(>|t|)   
(Intercept)  -1.1882     0.4083  -2.910  0.03338 * 
clusterID2    2.1933     0.5401   4.061  0.00972 **
---
Signif. codes:  0 ë***í 0.001 ë**í 0.01 ë*í 0.05 ë.í 0.1 ë í 1

Residual standard error: 0.7071 on 5 degrees of freedom
Multiple R-squared:  0.7674,	Adjusted R-squared:  0.7208 
```

```
F-statistic: 16.49 on 1 and 5 DF,  p-value: 0.00972


Response PawAngle_RF :

Call:
lm(formula = PawAngle_RF ~ clusterID)

Residuals:
CC041XCC012       CC012 CC032XCC013       CC017       CC023 
CC012XCC032       CC057 
   -0.43869    -0.48958     0.92826    -0.41812    -0.01256 
0.24746     0.18321 

Coefficients:
            Estimate Std. Error t value Pr(>|t|)   
(Intercept)  -1.3882     0.3229  -4.300  0.00772 **
clusterID2    1.7165     0.4271   4.019  0.01013 * 
---
Signif. codes:  0 ë***í 0.001 ë**í 0.01 ë*í 0.05 ë.í 0.1 ë í 1

Residual standard error: 0.5592 on 5 degrees of freedom
Multiple R-squared:  0.7636,      Adjusted R-squared:  0.7163 
F-statistic: 16.15 on 1 and 5 DF,  p-value: 0.01013


Response PawAngleVariability_RF :

Call:
lm(formula = PawAngleVariability_RF ~ clusterID)

Residuals:
CC041XCC012       CC012 CC032XCC013       CC017       CC023 
CC012XCC032       CC057 
   -0.03829     0.84255    -0.80426    -0.63000    -0.37246 
0.80825     0.19420 

Coefficients:
            Estimate Std. Error t value Pr(>|t|)  
(Intercept)  -1.1298     0.4151  -2.722   0.0417 *
clusterID2    2.1486     0.5491   3.913   0.0113 *
---
Signif. codes:  0 ë***í 0.001 ë**í 0.01 ë*í 0.05 ë.í 0.1 ë í 1

Residual standard error: 0.719 on 5 degrees of freedom
Multiple R-squared:  0.7538,      Adjusted R-squared:  0.7046 
F-statistic: 15.31 on 1 and 5 DF,  p-value: 0.01127


Response SWVar_LF :
```

```
Call:
lm(formula = SWVar_LF ~ clusterID)

Residuals:
CC041XCC012       CC012 CC032XCC013       CC017       CC023
CC012XCC032       CC057
 -1.3473730   0.7241927   0.6231803  -0.3145675  -0.0001624
-0.1138419   0.4285718

Coefficients:
            Estimate Std. Error t value Pr(>|t|)
(Intercept)   -1.181      0.449  -2.629   0.0466 *
clusterID2     2.317      0.594   3.901   0.0114 *
---
Signif. codes:  0 ë***í 0.001 ë**í 0.01 ë*í 0.05 ë.í 0.1 ë í 1

Residual standard error: 0.7777 on 5 degrees of freedom
Multiple R-squared:  0.7527,      Adjusted R-squared:  0.7033
F-statistic: 15.22 on 1 and 5 DF,  p-value: 0.01139


Response StrideFrequency_RF :

Call:
lm(formula = StrideFrequency_RF ~ clusterID)

Residuals:
CC041XCC012       CC012 CC032XCC013       CC017       CC023
CC012XCC032       CC057
     0.1171      0.7589     -0.8760     -1.1970      0.5981
0.4308      0.1681

Coefficients:
            Estimate Std. Error t value Pr(>|t|)
(Intercept)  -1.4586     0.4734  -3.081   0.0274 *
clusterID2    2.4332     0.6262   3.886   0.0116 *
---
Signif. codes:  0 ë***í 0.001 ë**í 0.01 ë*í 0.05 ë.í 0.1 ë í 1

Residual standard error: 0.8199 on 5 degrees of freedom
Multiple R-squared:  0.7512,      Adjusted R-squared:  0.7015
F-statistic:  15.1 on 1 and 5 DF,  p-value: 0.01158


Response AbsolutePawAngle_RF :

Call:
lm(formula = AbsolutePawAngle_RF ~ clusterID)
```

```
Residuals:
CC041XCC012       CC012 CC032XCC013       CC017       CC023
CC012XCC032       CC057
   -0.70873     0.01343     0.69530    -0.85847     0.26599
-0.01543     0.60790

Coefficients:
            Estimate Std. Error t value Pr(>|t|)
(Intercept)  -1.4778     0.3798  -3.891   0.0115 *
clusterID2    1.9287     0.5024   3.839   0.0121 *
---
Signif. codes:  0 ë***í 0.001 ë**í 0.01 ë*í 0.05 ë.í 0.1 ë í 1

Residual standard error: 0.6578 on 5 degrees of freedom
Multiple R-squared:  0.7467,      Adjusted R-squared:  0.696
F-statistic: 14.74 on 1 and 5 DF,  p-value: 0.01214


Response SwingDurationCV_RH :

Call:
lm(formula = SwingDurationCV_RH ~ clusterID)

Residuals:
CC041XCC012       CC012 CC032XCC013       CC017       CC023
CC012XCC032       CC057
  -0.007678    0.904353   -0.896675   -0.455507    0.017804
0.567198   -0.129494

Coefficients:
            Estimate Std. Error t value Pr(>|t|)
(Intercept)  -1.2203     0.3802  -3.210   0.0237 *
clusterID2    1.9187     0.5030   3.815   0.0124 *
---
Signif. codes:  0 ë***í 0.001 ë**í 0.01 ë*í 0.05 ë.í 0.1 ë í 1

Residual standard error: 0.6585 on 5 degrees of freedom
Multiple R-squared:  0.7443,      Adjusted R-squared:  0.6931
F-statistic: 14.55 on 1 and 5 DF,  p-value: 0.01244


Response PawAreaVariabilityatPeakStanceinsq.cm_RF :

Call:
lm(formula = PawAreaVariabilityatPeakStanceinsq.cm_RF ~ clusterID)

Residuals:
CC041XCC012       CC012 CC032XCC013       CC017       CC023
CC012XCC032       CC057
   -0.46676    -0.02527     0.49204     0.44372    -0.48386
```

```
0.37683    -0.33670

Coefficients:
            Estimate Std. Error t value Pr(>|t|)
(Intercept)  -0.4460     0.2765  -1.613   0.1677
clusterID2    1.3773     0.3658   3.765   0.0131 *
---
Signif. codes:  0 ë***í 0.001 ë**í 0.01 ë*í 0.05 ë.í 0.1 ë í 1

Residual standard error: 0.479 on 5 degrees of freedom
Multiple R-squared:  0.7392,	Adjusted R-squared:  0.6871
F-statistic: 14.18 on 1 and 5 DF,  p-value: 0.01309


Response AtaxiaCoefficient_LF :

Call:
lm(formula = AtaxiaCoefficient_LF ~ clusterID)

Residuals:
CC041XCC012       CC012 CC032XCC013       CC017       CC023
CC012XCC032       CC057
    -0.2357     -0.4582      0.6939     -1.2386      0.3595
0.6250      0.2541

Coefficients:
            Estimate Std. Error t value Pr(>|t|)
(Intercept)  -1.2056     0.4371  -2.758   0.0399 *
clusterID2    2.1725     0.5782   3.757   0.0132 *
---
Signif. codes:  0 ë***í 0.001 ë**í 0.01 ë*í 0.05 ë.í 0.1 ë í 1

Residual standard error: 0.757 on 5 degrees of freedom
Multiple R-squared:  0.7385,	Adjusted R-squared:  0.6862
F-statistic: 14.12 on 1 and 5 DF,  p-value: 0.01319


Response StrideFrequency_LH :

Call:
lm(formula = StrideFrequency_LH ~ clusterID)

Residuals:
CC041XCC012       CC012 CC032XCC013       CC017       CC023
CC012XCC032       CC057
   -0.20914     0.29117    -0.08203    -1.25800    -0.47704
0.77219     0.96285

Coefficients:
            Estimate Std. Error t value Pr(>|t|)
```

```
(Intercept)  -0.9780     0.4809  -2.034   0.0976 .
clusterID2    2.3301     0.6361   3.663   0.0146 *
---
Signif. codes:  0 ë***í 0.001 ë**í 0.01 ë*í 0.05 ë.í 0.1 ë í 1

Residual standard error: 0.8329 on 5 degrees of freedom
Multiple R-squared:  0.7285,      Adjusted R-squared:  0.6742
F-statistic: 13.42 on 1 and 5 DF,  p-value: 0.01455


Response StepAngleVar_LH :

Call:
lm(formula = StepAngleVar_LH ~ clusterID)

Residuals:
CC041XCC012       CC012 CC032XCC013       CC017       CC023
CC012XCC032       CC057
    0.17646     0.18864    -0.36509     0.29065    -0.07059
-0.40211     0.18205

Coefficients:
            Estimate Std. Error t value Pr(>|t|)
(Intercept)   0.2240     0.1797   1.247   0.2677
clusterID2   -0.8456     0.2377  -3.557   0.0163 *
---
Signif. codes:  0 ë***í 0.001 ë**í 0.01 ë*í 0.05 ë.í 0.1 ë í 1

Residual standard error: 0.3112 on 5 degrees of freedom
Multiple R-squared:  0.7168,      Adjusted R-squared:  0.6601
F-statistic: 12.65 on 1 and 5 DF,  p-value: 0.01626


Response Stance_LH :

Call:
lm(formula = Stance_LH ~ clusterID)

Residuals:
CC041XCC012       CC012 CC032XCC013       CC017       CC023
CC012XCC032       CC057
     0.2083     -0.4829      0.2746      0.8926      0.9133
-0.9950     -0.8108

Coefficients:
            Estimate Std. Error t value Pr(>|t|)
(Intercept)   0.8979     0.4919   1.825   0.1276
clusterID2   -2.2683     0.6508  -3.485   0.0176 *
---
Signif. codes:  0 ë***í 0.001 ë**í 0.01 ë*í 0.05 ë.í 0.1 ë í 1
```

```
Residual standard error: 0.8521 on 5 degrees of freedom
Multiple R-squared:  0.7084,     Adjusted R-squared:  0.6501 
F-statistic: 12.15 on 1 and 5 DF,  p-value: 0.01756


Response MAXdAratiodT_RH :

Call:
lm(formula = MAXdAratiodT_RH ~ clusterID)

Residuals:
CC041XCC012       CC012 CC032XCC013       CC017       CC023 
CC012XCC032       CC057 
    0.42061    -0.34064    -0.07997     1.38208    -0.92157 
0.06263    -0.52314 

Coefficients:
            Estimate Std. Error t value Pr(>|t|)  
(Intercept)   1.4292     0.4716   3.030   0.0291 *
clusterID2   -2.1121     0.6239  -3.385   0.0196 *
---
Signif. codes:  0 ë***í 0.001 ë**í 0.01 ë*í 0.05 ë.í 0.1 ë í 1

Residual standard error: 0.8169 on 5 degrees of freedom
Multiple R-squared:  0.6962,     Adjusted R-squared:  0.6355 
F-statistic: 11.46 on 1 and 5 DF,  p-value: 0.01956


Response StanceWidthCV_LF :

Call:
lm(formula = StanceWidthCV_LF ~ clusterID)

Residuals:
CC041XCC012       CC012 CC032XCC013       CC017       CC023 
CC012XCC032       CC057 
    -0.9444      0.7787      0.1657     -0.8956      1.3269 
-0.2971     -0.1342 

Coefficients:
            Estimate Std. Error t value Pr(>|t|)  
(Intercept)  -1.1961     0.5288  -2.262   0.0732 .
clusterID2    2.3508     0.6996   3.360   0.0201 *
---
Signif. codes:  0 ë***í 0.001 ë**í 0.01 ë*í 0.05 ë.í 0.1 ë í 1

Residual standard error: 0.9159 on 5 degrees of freedom
Multiple R-squared:  0.6931,     Adjusted R-squared:  0.6317 
F-statistic: 11.29 on 1 and 5 DF,  p-value: 0.0201
```

```
Response MidlineDistance_LH :

Call:
lm(formula = MidlineDistance_LH ~ clusterID)

Residuals:
CC041XCC012       CC012 CC032XCC013       CC017       CC023
CC012XCC032       CC057
   -0.67074     0.16207     0.50866     0.81244    -1.08401
0.25946     0.01211

Coefficients:
            Estimate Std. Error t value Pr(>|t|)
(Intercept)   0.8998     0.4193   2.146   0.0847 .
clusterID2   -1.8581     0.5547  -3.350   0.0203 *
---
Signif. codes:  0 ë***í 0.001 ë**í 0.01 ë*í 0.05 ë.í 0.1 ë í 1

Residual standard error: 0.7263 on 5 degrees of freedom
Multiple R-squared:  0.6917,     Adjusted R-squared:  0.6301
F-statistic: 11.22 on 1 and 5 DF,  p-value: 0.02034


Response pPropelStride_LF :

Call:
lm(formula = pPropelStride_LF ~ clusterID)

Residuals:
CC041XCC012       CC012 CC032XCC013       CC017       CC023
CC012XCC032       CC057
    0.36336     0.25278    -0.61614    -0.03962     0.65117
0.42400    -1.03555

Coefficients:
            Estimate Std. Error t value Pr(>|t|)
(Intercept)   0.5656     0.3876   1.459   0.2043
clusterID2   -1.7149     0.5127  -3.345   0.0204 *
---
Signif. codes:  0 ë***í 0.001 ë**í 0.01 ë*í 0.05 ë.í 0.1 ë í 1

Residual standard error: 0.6713 on 5 degrees of freedom
Multiple R-squared:  0.6911,     Adjusted R-squared:  0.6293
F-statistic: 11.19 on 1 and 5 DF,  p-value: 0.02045


Response PawAreaatPeakStanceinsq.cm_RH :
```

```
Call:
lm(formula = PawAreaatPeakStanceinsq.cm_RH ~ clusterID)

Residuals:
CC041XCC012       CC012 CC032XCC013       CC017       CC023 
CC012XCC032       CC057 
     0.6185     -0.4827     -0.1358      1.2216     -0.9064 
0.3961     -0.7114 

Coefficients:
            Estimate Std. Error t value Pr(>|t|)  
(Intercept)   1.3458     0.4906   2.743   0.0407 *
clusterID2   -2.1487     0.6491  -3.310   0.0212 *
---
Signif. codes:  0 ë***í 0.001 ë**í 0.01 ë*í 0.05 ë.í 0.1 ë í 1

Residual standard error: 0.8498 on 5 degrees of freedom
Multiple R-squared:  0.6867,	Adjusted R-squared:  0.624 
F-statistic: 10.96 on 1 and 5 DF,  p-value: 0.02123


Response Stride_RH :

Call:
lm(formula = Stride_RH ~ clusterID)

Residuals:
CC041XCC012       CC012 CC032XCC013       CC017       CC023 
CC012XCC032       CC057 
    0.18607    -0.41696     0.23089     0.23752     0.04413 
-0.13090    -0.15075 

Coefficients:
            Estimate Std. Error t value Pr(>|t|)  
(Intercept)   0.5945     0.1549   3.837   0.0122 *
clusterID2   -0.6686     0.2049  -3.262   0.0224 *
---
Signif. codes:  0 ë***í 0.001 ë**í 0.01 ë*í 0.05 ë.í 0.1 ë í 1

Residual standard error: 0.2683 on 5 degrees of freedom
Multiple R-squared:  0.6804,	Adjusted R-squared:  0.6164 
F-statistic: 10.64 on 1 and 5 DF,  p-value: 0.02239


Response PawAngleVariability_LF :

Call:
lm(formula = PawAngleVariability_LF ~ clusterID)

Residuals:
```

```
CC041XCC012       CC012 CC032XCC013       CC017       CC023
CC012XCC032       CC057
    0.05365     1.21676    -1.27041    -0.14359    -0.29511
0.53276    -0.09406

Coefficients:
            Estimate Std. Error t value Pr(>|t|)
(Intercept)  -1.6172     0.4829  -3.349   0.0204 *
clusterID2    2.0732     0.6388   3.245   0.0228 *
---
Signif. codes:  0 ë***í 0.001 ë**í 0.01 ë*í 0.05 ë.í 0.1 ë í 1

Residual standard error: 0.8364 on 5 degrees of freedom
Multiple R-squared:  0.6781,     Adjusted R-squared:  0.6137
F-statistic: 10.53 on 1 and 5 DF,  p-value: 0.02281


Response AtaxiaCoefficient_LH :

Call:
lm(formula = AtaxiaCoefficient_LH ~ clusterID)

Residuals:
CC041XCC012       CC012 CC032XCC013       CC017       CC023
CC012XCC032       CC057
    0.05376     0.39966    -0.45342    -0.70949    -1.08499
0.92395     0.87053

Coefficients:
            Estimate Std. Error t value Pr(>|t|)
(Intercept)  -0.8445     0.4940  -1.710   0.1480
clusterID2    2.0751     0.6535   3.175   0.0247 *
---
Signif. codes:  0 ë***í 0.001 ë**í 0.01 ë*í 0.05 ë.í 0.1 ë í 1

Residual standard error: 0.8556 on 5 degrees of freedom
Multiple R-squared:  0.6685,     Adjusted R-squared:  0.6022
F-statistic: 10.08 on 1 and 5 DF,  p-value: 0.02466


Response MINdAratiodT_LF :

Call:
lm(formula = MINdAratiodT_LF ~ clusterID)

Residuals:
CC041XCC012       CC012 CC032XCC013       CC017       CC023
CC012XCC032       CC057
   -0.50908    -0.66633     1.17540     0.62194    -0.04478
-0.37385    -0.20330
```

```
Coefficients:
            Estimate Std. Error t value Pr(>|t|)
(Intercept)   0.9201     0.4207   2.187   0.0804 .
clusterID2   -1.7292     0.5565  -3.107   0.0266 *
---
Signif. codes:  0 ë***í 0.001 ë**í 0.01 ë*í 0.05 ë.í 0.1 ë í 1

Residual standard error: 0.7286 on 5 degrees of freedom
Multiple R-squared:  0.6588,	Adjusted R-squared:  0.5906
F-statistic: 9.655 on 1 and 5 DF,  p-value: 0.02664


Response StrideLength_RH :

Call:
lm(formula = StrideLength_RH ~ clusterID)

Residuals:
CC041XCC012       CC012 CC032XCC013       CC017       CC023
CC012XCC032       CC057
    0.38400    -0.80683     0.42283     0.42881     0.07737
-0.23250    -0.27368

Coefficients:
            Estimate Std. Error t value Pr(>|t|)
(Intercept)   0.9524     0.2939   3.240   0.0229 *
clusterID2   -1.2074     0.3888  -3.105   0.0267 *
---
Signif. codes:  0 ë***í 0.001 ë**í 0.01 ë*í 0.05 ë.í 0.1 ë í 1

Residual standard error: 0.5091 on 5 degrees of freedom
Multiple R-squared:  0.6585,	Adjusted R-squared:  0.5902
F-statistic: 9.641 on 1 and 5 DF,  p-value: 0.0267


Response MidlineDistance_RH :

Call:
lm(formula = MidlineDistance_RH ~ clusterID)

Residuals:
CC041XCC012       CC012 CC032XCC013       CC017       CC023
CC012XCC032       CC057
     0.4236     -0.3071     -0.1165      0.5326     -1.0673
0.3274      0.2072

Coefficients:
            Estimate Std. Error t value Pr(>|t|)
(Intercept)   0.4559     0.3522   1.294   0.2521
```

```
clusterID2   -1.4323     0.4659  -3.075   0.0276 *
---
Signif. codes:  0 ë***í 0.001 ë**í 0.01 ë*í 0.05 ë.í 0.1 ë í 1

Residual standard error: 0.61 on 5 degrees of freedom
Multiple R-squared:  0.654,	Adjusted R-squared:  0.5849
F-statistic: 9.453 on 1 and 5 DF,  p-value: 0.02764


Response PawAngleVariability_LH :

Call:
lm(formula = PawAngleVariability_LH ~ clusterID)

Residuals:
CC041XCC012       CC012 CC032XCC013       CC017       CC023
CC012XCC032       CC057
     0.3098      0.3961     -0.7059     -0.5794     -0.3445
1.2449     -0.3210

Coefficients:
            Estimate Std. Error t value Pr(>|t|)
(Intercept)  -0.6886     0.4365  -1.577   0.1755
clusterID2    1.7501     0.5775   3.031   0.0291 *
---
Signif. codes:  0 ë***í 0.001 ë**í 0.01 ë*í 0.05 ë.í 0.1 ë í 1

Residual standard error: 0.7561 on 5 degrees of freedom
Multiple R-squared:  0.6475,	Adjusted R-squared:  0.577
F-statistic: 9.184 on 1 and 5 DF,  p-value: 0.02906


Response PawAngleVariability_RH :

Call:
lm(formula = PawAngleVariability_RH ~ clusterID)

Residuals:
CC041XCC012       CC012 CC032XCC013       CC017       CC023
CC012XCC032       CC057
     0.4037      0.3571     -0.7608     -0.2777      0.2066
0.9212     -0.8502

Coefficients:
            Estimate Std. Error t value Pr(>|t|)
(Intercept)  -0.9797     0.4132  -2.371   0.0639 .
clusterID2    1.6512     0.5466   3.021   0.0294 *
---
Signif. codes:  0 ë***í 0.001 ë**í 0.01 ë*í 0.05 ë.í 0.1 ë í 1
```

```
Residual standard error: 0.7156 on 5 degrees of freedom
Multiple R-squared:  0.6461,	Adjusted R-squared:  0.5753
F-statistic: 9.127 on 1 and 5 DF,  p-value: 0.02938


Response AbsolutePawAngle_LF :

Call:
lm(formula = AbsolutePawAngle_LF ~ clusterID)

Residuals:
CC041XCC012       CC012 CC032XCC013       CC017       CC023
CC012XCC032       CC057
     0.1749     -0.7630      0.5881      0.5452      0.4045
-1.4270      0.4773

Coefficients:
            Estimate Std. Error t value Pr(>|t|)
(Intercept)  -1.0402     0.4956  -2.099   0.0899 .
clusterID2    1.9312     0.6556   2.946   0.0320 *
---
Signif. codes:  0 ë***í 0.001 ë**í 0.01 ë*í 0.05 ë.í 0.1 ë í 1

Residual standard error: 0.8583 on 5 degrees of freedom
Multiple R-squared:  0.6345,	Adjusted R-squared:  0.5614
F-statistic: 8.679 on 1 and 5 DF,  p-value: 0.03204


Response AxisDistance_LF :

Call:
lm(formula = AxisDistance_LF ~ clusterID)

Residuals:
CC041XCC012       CC012 CC032XCC013       CC017       CC023
CC012XCC032       CC057
   -0.20410     0.40486    -0.20076    -0.12354     0.27785
-0.17530     0.02099

Coefficients:
            Estimate Std. Error t value Pr(>|t|)
(Intercept)  -0.3556     0.1570  -2.266   0.0728 .
clusterID2    0.5854     0.2076   2.820   0.0371 *
---
Signif. codes:  0 ë***í 0.001 ë**í 0.01 ë*í 0.05 ë.í 0.1 ë í 1

Residual standard error: 0.2718 on 5 degrees of freedom
Multiple R-squared:  0.6139,	Adjusted R-squared:  0.5367
F-statistic: 7.951 on 1 and 5 DF,  p-value: 0.03712
```

```
Response PawDrag_RH :

Call:
lm(formula = PawDrag_RH ~ clusterID)

Residuals:
CC041XCC012       CC012 CC032XCC013       CC017       CC023
CC012XCC032       CC057
   0.929971   -0.949520    0.019550   -0.892901    0.549054
0.335405    0.008441

Coefficients:
            Estimate Std. Error t value Pr(>|t|)
(Intercept)  -1.1647     0.4456  -2.614   0.0474 *
clusterID2    1.6228     0.5894   2.753   0.0402 *
---
Signif. codes:  0 ë***í 0.001 ë**í 0.01 ë*í 0.05 ë.í 0.1 ë í 1

Residual standard error: 0.7718 on 5 degrees of freedom
Multiple R-squared:  0.6025,      Adjusted R-squared:  0.523
F-statistic: 7.579 on 1 and 5 DF,  p-value: 0.04016


Response StrideFrequency_RH :

Call:
lm(formula = StrideFrequency_RH ~ clusterID)

Residuals:
CC041XCC012       CC012 CC032XCC013       CC017       CC023
CC012XCC032       CC057
    0.04515     1.31955    -1.36470    -1.05967    -0.41405
1.11198     0.36174

Coefficients:
            Estimate Std. Error t value Pr(>|t|)
(Intercept)  -1.0995     0.6464  -1.701   0.1497
clusterID2    2.3208     0.8551   2.714   0.0421 *
---
Signif. codes:  0 ë***í 0.001 ë**í 0.01 ë*í 0.05 ë.í 0.1 ë í 1

Residual standard error: 1.12 on 5 degrees of freedom
Multiple R-squared:  0.5957,      Adjusted R-squared:  0.5148
F-statistic: 7.366 on 1 and 5 DF,  p-value: 0.04207


Response pPropelStance_LF :

Call:
```

```
lm(formula = pPropelStance_LF ~ clusterID)

Residuals:
CC041XCC012       CC012 CC032XCC013       CC017       CC023
CC012XCC032       CC057
    0.20258     0.31584    -0.51842     0.03828     0.84807
0.07719    -0.96353

Coefficients:
            Estimate Std. Error t value Pr(>|t|)
(Intercept)   0.4416     0.3710   1.190   0.2874
clusterID2   -1.3147     0.4908  -2.679   0.0439 *
---
Signif. codes:  0 ë***í 0.001 ë**í 0.01 ë*í 0.05 ë.í 0.1 ë í 1

Residual standard error: 0.6426 on 5 degrees of freedom
Multiple R-squared:  0.5894,      Adjusted R-squared:  0.5072
F-statistic: 7.176 on 1 and 5 DF,  p-value: 0.04388


Response pBrakeStance_LF :

Call:
lm(formula = pBrakeStance_LF ~ clusterID)

Residuals:
CC041XCC012       CC012 CC032XCC013       CC017       CC023
CC012XCC032       CC057
   -0.20258    -0.31585     0.51843    -0.03828    -0.84808
-0.07719     0.96355

Coefficients:
            Estimate Std. Error t value Pr(>|t|)
(Intercept)  -0.4417     0.3710  -1.190   0.2873
clusterID2    1.3147     0.4908   2.679   0.0439 *
---
Signif. codes:  0 ë***í 0.001 ë**í 0.01 ë*í 0.05 ë.í 0.1 ë í 1

Residual standard error: 0.6426 on 5 degrees of freedom
Multiple R-squared:  0.5894,      Adjusted R-squared:  0.5072
F-statistic: 7.176 on 1 and 5 DF,  p-value: 0.04388


Response Brake_LH :

Call:
lm(formula = Brake_LH ~ clusterID)

Residuals:
CC041XCC012       CC012 CC032XCC013       CC017       CC023
```

```
CC012XCC032       CC057
   -0.03295    -0.16472     0.19767    -0.09897    -0.43292
1.46779    -0.93590

Coefficients:
            Estimate Std. Error t value Pr(>|t|)
(Intercept)   0.6748     0.4687    1.44   0.2095
clusterID2   -1.6181     0.6200   -2.61   0.0477 *
---
Signif. codes:  0 ë***í 0.001 ë**í 0.01 ë*í 0.05 ë.í 0.1 ë í 1

Residual standard error: 0.8118 on 5 degrees of freedom
Multiple R-squared:  0.5767,      Adjusted R-squared:  0.492
F-statistic: 6.811 on 1 and 5 DF,  p-value: 0.04768


Response SLVar_RH :

Call:
lm(formula = SLVar_RH ~ clusterID)

Residuals:
CC041XCC012       CC012 CC032XCC013       CC017       CC023
CC012XCC032       CC057
    0.30906    -0.61722     0.30816     0.62153     1.16298
-0.04294    -1.74156

Coefficients:
            Estimate Std. Error t value Pr(>|t|)
(Intercept)  -0.9229     0.5969  -1.546   0.1827
clusterID2    2.0445     0.7897   2.589   0.0489 *
---
Signif. codes:  0 ë***í 0.001 ë**í 0.01 ë*í 0.05 ë.í 0.1 ë í 1

Residual standard error: 1.034 on 5 degrees of freedom
Multiple R-squared:  0.5727,      Adjusted R-squared:  0.4873
F-statistic: 6.703 on 1 and 5 DF,  p-value: 0.0489


Response pPropelStride_RF :

Call:
lm(formula = pPropelStride_RF ~ clusterID)

Residuals:
CC041XCC012       CC012 CC032XCC013       CC017       CC023
CC012XCC032       CC057
   -0.38234     0.46817    -0.08583    -1.00832    -0.07730
1.12352    -0.03791
```

```
Coefficients:
            Estimate Std. Error t value Pr(>|t|)
(Intercept)   0.5548     0.4210   1.318   0.2447
clusterID2   -1.4365     0.5570  -2.579   0.0495 *
---
Signif. codes:  0 ë***í 0.001 ë**í 0.01 ë*í 0.05 ë.í 0.1 ë í 1

Residual standard error: 0.7293 on 5 degrees of freedom
Multiple R-squared:  0.5709,	Adjusted R-squared:  0.485
F-statistic: 6.651 on 1 and 5 DF,  p-value: 0.04949


Response GaitSymmetry_RH :

Call:
lm(formula = GaitSymmetry_RH ~ clusterID)

Residuals:
CC041XCC012       CC012 CC032XCC013       CC017       CC023
CC012XCC032       CC057
  0.2795994  -0.4616714   0.1820720  -1.0106397   1.5094941
-0.0001558  -0.4986986

Coefficients:
            Estimate Std. Error t value Pr(>|t|)
(Intercept)  -1.3278     0.5081  -2.613   0.0475 *
clusterID2    1.7305     0.6722   2.574   0.0498 *
---
Signif. codes:  0 ë***í 0.001 ë**í 0.01 ë*í 0.05 ë.í 0.1 ë í 1

Residual standard error: 0.8801 on 5 degrees of freedom
Multiple R-squared:   0.57,	Adjusted R-squared:  0.484
F-statistic: 6.627 on 1 and 5 DF,  p-value: 0.04977


Response GaitSymmetry_LH :

Call:
lm(formula = GaitSymmetry_LH ~ clusterID)

Residuals:
CC041XCC012       CC012 CC032XCC013       CC017       CC023
CC012XCC032       CC057
  0.2734979  -0.4515966   0.1780987  -0.9885851   1.4765533
-0.0001524  -0.4878158

Coefficients:
            Estimate Std. Error t value Pr(>|t|)
(Intercept)  -1.2418     0.4971  -2.498   0.0546 .
clusterID2    1.6928     0.6575   2.574   0.0498 *
```

```
---
Signif. codes:  0 ë***í 0.001 ë**í 0.01 ë*í 0.05 ë.í 0.1 ë í 1

Residual standard error: 0.8609 on 5 degrees of freedom
Multiple R-squared:   0.57,	Adjusted R-squared:  0.484 
F-statistic: 6.627 on 1 and 5 DF,  p-value: 0.04977


Response GaitSymmetry_RF :

Call:
lm(formula = GaitSymmetry_RF ~ clusterID)

Residuals:
CC041XCC012       CC012 CC032XCC013       CC017       CC023 
CC012XCC032       CC057 
  0.2734979  -0.4515966   0.1780987  -0.9885851   1.4765533 
-0.0001524  -0.4878158 

Coefficients:
            Estimate Std. Error t value Pr(>|t|)  
(Intercept)  -1.2418     0.4971  -2.498   0.0546 .
clusterID2    1.6928     0.6575   2.574   0.0498 *
---
Signif. codes:  0 ë***í 0.001 ë**í 0.01 ë*í 0.05 ë.í 0.1 ë í 1

Residual standard error: 0.8609 on 5 degrees of freedom
Multiple R-squared:   0.57,	Adjusted R-squared:  0.484 
F-statistic: 6.627 on 1 and 5 DF,  p-value: 0.04977


Response GaitSymmetry_LF :

Call:
lm(formula = GaitSymmetry_LF ~ clusterID)

Residuals:
CC041XCC012       CC012 CC032XCC013       CC017       CC023 
CC012XCC032       CC057 
  0.2875472  -0.4589872   0.1714400  -0.9897340   1.4782693 
-0.0001526  -0.4883827 

Coefficients:
            Estimate Std. Error t value Pr(>|t|)  
(Intercept)  -1.2376     0.4984  -2.483   0.0556 .
clusterID2    1.6879     0.6593   2.560   0.0506 .
---
Signif. codes:  0 ë***í 0.001 ë**í 0.01 ë*í 0.05 ë.í 0.1 ë í 1

Residual standard error: 0.8633 on 5 degrees of freedom
```

```
Multiple R-squared:  0.5672,	Adjusted R-squared:  0.4807 
F-statistic: 6.553 on 1 and 5 DF,  p-value: 0.05065


Response SLVar_LH :

Call:
lm(formula = SLVar_LH ~ clusterID)

Residuals:
CC041XCC012       CC012 CC032XCC013       CC017       CC023 
CC012XCC032       CC057 
     0.4410      0.3368     -0.7778      0.1067     -1.3894 
0.6516      0.6311 

Coefficients:
            Estimate Std. Error t value Pr(>|t|)  
(Intercept)  -0.5565     0.4951  -1.124   0.3121  
clusterID2    1.6568     0.6550   2.529   0.0526 .
---
Signif. codes:  0 ë***í 0.001 ë**í 0.01 ë*í 0.05 ë.í 0.1 ë í 1

Residual standard error: 0.8576 on 5 degrees of freedom
Multiple R-squared:  0.5613,	Adjusted R-squared:  0.4736 
F-statistic: 6.398 on 1 and 5 DF,  p-value: 0.05257


Response pBrakeStride_LF :

Call:
lm(formula = pBrakeStride_LF ~ clusterID)

Residuals:
CC041XCC012       CC012 CC032XCC013       CC017       CC023 
CC012XCC032       CC057 
   0.009031   -0.220625    0.211594   -0.101387   -0.964414 
0.042545    1.023257 

Coefficients:
            Estimate Std. Error t value Pr(>|t|)  
(Intercept)  -0.4752     0.3726  -1.275   0.2583  
clusterID2    1.2354     0.4929   2.506   0.0541 .
---
Signif. codes:  0 ë***í 0.001 ë**í 0.01 ë*í 0.05 ë.í 0.1 ë í 1

Residual standard error: 0.6454 on 5 degrees of freedom
Multiple R-squared:  0.5568,	Adjusted R-squared:  0.4681 
F-statistic: 6.281 on 1 and 5 DF,  p-value: 0.05408
```

```
Response Swing_RH :

Call:
lm(formula = Swing_RH ~ clusterID)

Residuals:
CC041XCC012       CC012 CC032XCC013       CC017       CC023
CC012XCC032       CC057
 -0.0056898  -0.0047621   0.0104519   0.0008166  -0.0100256
0.0125405  -0.0033315

Coefficients:
             Estimate Std. Error t value Pr(>|t|)
(Intercept)  0.243452   0.005378   45.27 9.93e-08 ***
clusterID2  -0.017500   0.007114   -2.46   0.0572 .
---
Signif. codes:  0 ë***í 0.001 ë**í 0.01 ë*í 0.05 ë.í 0.1 ë í 1

Residual standard error: 0.009315 on 5 degrees of freedom
Multiple R-squared:  0.5475,	Adjusted R-squared:  0.457
F-statistic: 6.051 on 1 and 5 DF,  p-value: 0.05724


Response Stance_RH :

Call:
lm(formula = Stance_RH ~ clusterID)

Residuals:
CC041XCC012       CC012 CC032XCC013       CC017       CC023
CC012XCC032       CC057
     0.2789     -0.4243      0.1454      0.2603      0.1598
-0.2849     -0.1352

Coefficients:
            Estimate Std. Error t value Pr(>|t|)
(Intercept)   0.5592     0.1773   3.154   0.0253 *
clusterID2   -0.5662     0.2346  -2.414   0.0606 .
---
Signif. codes:  0 ë***í 0.001 ë**í 0.01 ë*í 0.05 ë.í 0.1 ë í 1

Residual standard error: 0.3071 on 5 degrees of freedom
Multiple R-squared:  0.5382,	Adjusted R-squared:  0.4458
F-statistic: 5.827 on 1 and 5 DF,  p-value: 0.06057


Response StepAngle_LH :

Call:
lm(formula = StepAngle_LH ~ clusterID)
```

```
Residuals:
CC041XCC012       CC012 CC032XCC013       CC017       CC023
CC012XCC032       CC057
   -0.07174     0.93089    -0.85915    -0.91336     0.93303
0.70216    -0.72183

Coefficients:
            Estimate Std. Error t value Pr(>|t|)
(Intercept)  -1.2275     0.5372  -2.285   0.0711 .
clusterID2    1.6872     0.7106   2.374   0.0636 .
---
Signif. codes:  0 ë***í 0.001 ë**í 0.01 ë*í 0.05 ë.í 0.1 ë í 1

Residual standard error: 0.9304 on 5 degrees of freedom
Multiple R-squared:  0.5299,	Adjusted R-squared:  0.4359
F-statistic: 5.636 on 1 and 5 DF,  p-value: 0.06363


Response PawAreaatPeakStanceinsq.cm_LF :

Call:
lm(formula = PawAreaatPeakStanceinsq.cm_LF ~ clusterID)

Residuals:
CC041XCC012       CC012 CC032XCC013       CC017       CC023
CC012XCC032       CC057
     0.9447      0.2565     -1.2012      0.9366     -0.7632
0.5625     -0.7359

Coefficients:
            Estimate Std. Error t value Pr(>|t|)
(Intercept)   1.2431     0.5609   2.216   0.0775 .
clusterID2   -1.6245     0.7420  -2.189   0.0802 .
---
Signif. codes:  0 ë***í 0.001 ë**í 0.01 ë*í 0.05 ë.í 0.1 ë í 1

Residual standard error: 0.9715 on 5 degrees of freedom
Multiple R-squared:  0.4895,	Adjusted R-squared:  0.3873
F-statistic: 4.793 on 1 and 5 DF,  p-value: 0.08016


Response StepAngleCV_LH :

Call:
lm(formula = StepAngleCV_LH ~ clusterID)

Residuals:
CC041XCC012       CC012 CC032XCC013       CC017       CC023
CC012XCC032       CC057
```

```
     0.1563     -0.5356      0.3793      1.3225     -0.3568 
-1.2794      0.3138 

Coefficients:
            Estimate Std. Error t value Pr(>|t|)  
(Intercept)   0.8430     0.5207   1.619   0.1664  
clusterID2   -1.4595     0.6888  -2.119   0.0876 .
---
Signif. codes:  0 ë***í 0.001 ë**í 0.01 ë*í 0.05 ë.í 0.1 ë í 1

Residual standard error: 0.9019 on 5 degrees of freedom
Multiple R-squared:  0.4731,	Adjusted R-squared:  0.3677 
F-statistic: 4.489 on 1 and 5 DF,  p-value: 0.08764


Response SWVar_LH :

Call:
lm(formula = SWVar_LH ~ clusterID)

Residuals:
CC041XCC012       CC012 CC032XCC013       CC017       CC023 
CC012XCC032       CC057 
    0.29631     1.60625    -1.90256     0.01181    -0.21878 
0.31353    -0.10656 

Coefficients:
            Estimate Std. Error t value Pr(>|t|)  
(Intercept)  -0.8726     0.6555  -1.331   0.2406  
clusterID2    1.8152     0.8671   2.093   0.0905 .
---
Signif. codes:  0 ë***í 0.001 ë**í 0.01 ë*í 0.05 ë.í 0.1 ë í 1

Residual standard error: 1.135 on 5 degrees of freedom
Multiple R-squared:  0.4671,	Adjusted R-squared:  0.3605 
F-statistic: 4.382 on 1 and 5 DF,  p-value: 0.09052


Response Brake_RF :

Call:
lm(formula = Brake_RF ~ clusterID)

Residuals:
CC041XCC012       CC012 CC032XCC013       CC017       CC023 
CC012XCC032       CC057 
    0.88013    -0.97024     0.09011     1.58439    -0.31711 
-1.22314    -0.04415 

Coefficients:
```

```
            Estimate Std. Error t value Pr(>|t|)
(Intercept)   1.0269     0.6236   1.647   0.1605
clusterID2   -1.7131     0.8249  -2.077   0.0925 .
---
Signif. codes:  0 ë***í 0.001 ë**í 0.01 ë*í 0.05 ë.í 0.1 ë í 1

Residual standard error: 1.08 on 5 degrees of freedom
Multiple R-squared:  0.4631,      Adjusted R-squared:  0.3557
F-statistic: 4.312 on 1 and 5 DF,  p-value: 0.09246


Response PawAngle_LF :

Call:
lm(formula = PawAngle_LF ~ clusterID)

Residuals:
CC041XCC012       CC012 CC032XCC013       CC017       CC023
CC012XCC032       CC057
    -0.1047      1.1586     -1.0540     -0.7785     -0.3282
1.7422     -0.6355

Coefficients:
            Estimate Std. Error t value Pr(>|t|)
(Intercept)   1.0252     0.6642   1.544   0.1833
clusterID2   -1.7959     0.8786  -2.044   0.0964 .
---
Signif. codes:  0 ë***í 0.001 ë**í 0.01 ë*í 0.05 ë.í 0.1 ë í 1

Residual standard error: 1.15 on 5 degrees of freedom
Multiple R-squared:  0.4552,      Adjusted R-squared:  0.3463
F-statistic: 4.178 on 1 and 5 DF,  p-value: 0.09638


Response StanceWidthCV_LH :

Call:
lm(formula = StanceWidthCV_LH ~ clusterID)

Residuals:
CC041XCC012       CC012 CC032XCC013       CC017       CC023
CC012XCC032       CC057
    0.36913     1.53628    -1.90541    -0.10634    -0.05265
0.62001    -0.46101

Coefficients:
            Estimate Std. Error t value Pr(>|t|)
(Intercept)  -0.7421     0.6702  -1.107   0.3186
clusterID2    1.8062     0.8866   2.037   0.0972 .
---
```

```
Signif. codes:  0 ë***í 0.001 ë**í 0.01 ë*í 0.05 ë.í 0.1 ë í 1

Residual standard error: 1.161 on 5 degrees of freedom
Multiple R-squared:  0.4536,     Adjusted R-squared:  0.3443
F-statistic:  4.15 on 1 and 5 DF,  p-value: 0.09722


Response PawDrag_LH :

Call:
lm(formula = PawDrag_LH ~ clusterID)

Residuals:
CC041XCC012       CC012 CC032XCC013       CC017       CC023
CC012XCC032       CC057
     0.5412     -0.3511     -0.1902     -0.8122      0.6791
-1.1025      1.2357

Coefficients:
            Estimate Std. Error t value Pr(>|t|)
(Intercept)  -1.3025     0.5364  -2.428   0.0595 .
clusterID2    1.4445     0.7096   2.036   0.0974 .
---
Signif. codes:  0 ë***í 0.001 ë**í 0.01 ë*í 0.05 ë.í 0.1 ë í 1

Residual standard error: 0.9291 on 5 degrees of freedom
Multiple R-squared:  0.4532,     Adjusted R-squared:  0.3438
F-statistic: 4.144 on 1 and 5 DF,  p-value: 0.0974


Response nSteps_RF :

Call:
lm(formula = nSteps_RF ~ clusterID)

Residuals:
CC041XCC012       CC012 CC032XCC013       CC017       CC023
CC012XCC032       CC057
    -0.6180      0.8398     -0.2218     -1.0144     -0.5688
0.9757      0.6075

Coefficients:
            Estimate Std. Error t value Pr(>|t|)
(Intercept)  -0.6436     0.5040  -1.277   0.2577
clusterID2    1.3460     0.6667   2.019   0.0995 .
---
Signif. codes:  0 ë***í 0.001 ë**í 0.01 ë*í 0.05 ë.í 0.1 ë í 1

Residual standard error: 0.8729 on 5 degrees of freedom
Multiple R-squared:  0.4491,     Adjusted R-squared:  0.3389
```

```
F-statistic: 4.076 on 1 and 5 DF,  p-value: 0.09952


Response MAXdAratiodT_LF :

Call:
lm(formula = MAXdAratiodT_LF ~ clusterID)

Residuals:
CC041XCC012       CC012 CC032XCC013       CC017       CC023
CC012XCC032       CC057
    0.68780     1.01194    -1.69974     0.50715    -0.02371
0.49025    -0.97370

Coefficients:
            Estimate Std. Error t value Pr(>|t|)
(Intercept)   1.3238     0.6236   2.123   0.0872 .
clusterID2   -1.6437     0.8249  -1.993   0.1029
---
Signif. codes:  0 ë***í 0.001 ë**í 0.01 ë*í 0.05 ë.í 0.1 ë í 1

Residual standard error: 1.08 on 5 degrees of freedom
Multiple R-squared:  0.4426,      Adjusted R-squared:  0.3311
F-statistic: 3.971 on 1 and 5 DF,  p-value: 0.1029


Response SLVar_RF :

Call:
lm(formula = SLVar_RF ~ clusterID)

Residuals:
CC041XCC012       CC012 CC032XCC013       CC017       CC023
CC012XCC032       CC057
   -0.64508    -0.62608     1.27116    -0.09455    -0.69037
0.88814    -0.10322

Coefficients:
            Estimate Std. Error t value Pr(>|t|)
(Intercept)  -0.2380     0.4973  -0.479    0.652
clusterID2    1.2919     0.6578   1.964    0.107

Residual standard error: 0.8613 on 5 degrees of freedom
Multiple R-squared:  0.4355,      Adjusted R-squared:  0.3226
F-statistic: 3.857 on 1 and 5 DF,  p-value: 0.1067


Response MAXdAratiodT_RF :

Call:
```

```
lm(formula = MAXdAratiodT_RF ~ clusterID)

Residuals:
CC041XCC012       CC012 CC032XCC013       CC017       CC023
CC012XCC032       CC057
     0.8542     -0.3239     -0.5303      1.0758     -0.2898
0.6553     -1.4412

Coefficients:
            Estimate Std. Error t value Pr(>|t|)
(Intercept)   1.0262     0.5694   1.802    0.131
clusterID2   -1.4408     0.7533  -1.913    0.114

Residual standard error: 0.9863 on 5 degrees of freedom
Multiple R-squared:  0.4225,      Adjusted R-squared:  0.307
F-statistic: 3.658 on 1 and 5 DF,  p-value: 0.114


Response nSteps_LF :

Call:
lm(formula = nSteps_LF ~ clusterID)

Residuals:
CC041XCC012       CC012 CC032XCC013       CC017       CC023
CC012XCC032       CC057
    -0.7027      1.0123     -0.3096     -0.5126     -0.8708
1.0289      0.3546

Coefficients:
            Estimate Std. Error t value Pr(>|t|)
(Intercept)  -0.3974     0.5046  -0.788    0.467
clusterID2    1.2435     0.6676   1.863    0.122

Residual standard error: 0.8741 on 5 degrees of freedom
Multiple R-squared:  0.4097,      Adjusted R-squared:  0.2916
F-statistic:  3.47 on 1 and 5 DF,  p-value: 0.1215


Response PawAreaatPeakStanceinsq.cm_RF :

Call:
lm(formula = PawAreaatPeakStanceinsq.cm_RF ~ clusterID)

Residuals:
CC041XCC012       CC012 CC032XCC013       CC017       CC023
CC012XCC032       CC057
     1.1036     -0.4037     -0.7000      1.4711     -0.4785
0.1442     -1.1369
```

```
Coefficients:
            Estimate Std. Error t value Pr(>|t|)
(Intercept)   1.1218     0.6098   1.840    0.125
clusterID2   -1.4688     0.8067  -1.821    0.128

Residual standard error: 1.056 on 5 degrees of freedom
Multiple R-squared:  0.3987,	Adjusted R-squared:  0.2785 
F-statistic: 3.316 on 1 and 5 DF,  p-value: 0.1283


Response Propel_RH :

Call:
lm(formula = Propel_RH ~ clusterID)

Residuals:
CC041XCC012       CC012 CC032XCC013       CC017       CC023 
CC012XCC032       CC057 
     0.5427     -0.7341      0.1914      0.7943      0.2541 
-1.1810      0.1326 

Coefficients:
            Estimate Std. Error t value Pr(>|t|)
(Intercept)   0.7384     0.4456   1.657    0.158
clusterID2   -1.0709     0.5894  -1.817    0.129

Residual standard error: 0.7718 on 5 degrees of freedom
Multiple R-squared:  0.3977,	Adjusted R-squared:  0.2772 
F-statistic: 3.301 on 1 and 5 DF,  p-value: 0.1289


Response StanceFactor_LF :

Call:
lm(formula = StanceFactor_LF ~ clusterID)

Residuals:
CC041XCC012       CC012 CC032XCC013       CC017       CC023 
CC012XCC032       CC057 
   -0.77903     0.46452     0.31451    -1.06097     0.01857 
0.69616     0.34624 

Coefficients:
            Estimate Std. Error t value Pr(>|t|)
(Intercept)  -0.7827     0.4205  -1.861    0.122
clusterID2    0.9828     0.5562   1.767    0.137

Residual standard error: 0.7283 on 5 degrees of freedom
Multiple R-squared:  0.3844,	Adjusted R-squared:  0.2613 
F-statistic: 3.122 on 1 and 5 DF,  p-value: 0.1375
```

```
Response PawAreaVariabilityatPeakStanceinsq.cm_LH :

Call:
lm(formula = PawAreaVariabilityatPeakStanceinsq.cm_LH ~ clusterID)

Residuals:
CC041XCC012       CC012 CC032XCC013       CC017       CC023
CC012XCC032       CC057
     0.6337     -0.1084     -0.5253      0.3038     -1.3532
0.8183      0.2312

Coefficients:
            Estimate Std. Error t value Pr(>|t|)
(Intercept) -0.08305    0.47157  -0.176    0.867
clusterID2   1.09179    0.62383   1.750    0.140

Residual standard error: 0.8168 on 5 degrees of freedom
Multiple R-squared:  0.3799,      Adjusted R-squared:  0.2559
F-statistic: 3.063 on 1 and 5 DF,  p-value: 0.1405


Response SLVar_LF :

Call:
lm(formula = SLVar_LF ~ clusterID)

Residuals:
CC041XCC012       CC012 CC032XCC013       CC017       CC023
CC012XCC032       CC057
   -0.24941    -1.19755     1.44697    -0.22192     0.33583
-0.04791    -0.06599

Coefficients:
            Estimate Std. Error t value Pr(>|t|)
(Intercept)  -0.8679     0.5006  -1.734    0.143
clusterID2    1.1246     0.6622   1.698    0.150

Residual standard error: 0.867 on 5 degrees of freedom
Multiple R-squared:  0.3658,      Adjusted R-squared:  0.239
F-statistic: 2.884 on 1 and 5 DF,  p-value: 0.1502


Response HindLimbSharedStanceTime_LH :

Call:
lm(formula = HindLimbSharedStanceTime_LH ~ clusterID)

Residuals:
```

```
CC041XCC012       CC012 CC032XCC013       CC017       CC023
CC012XCC032       CC057
    0.01609    -0.03215     0.01606     0.02887     0.05956
-0.06774    -0.02070

Coefficients:
            Estimate Std. Error t value Pr(>|t|)
(Intercept)  0.26525    0.02702   9.818 0.000187 ***
clusterID2  -0.05820    0.03574  -1.628 0.164367
---
Signif. codes:  0 ë***í 0.001 ë**í 0.01 ë*í 0.05 ë.í 0.1 ë í 1

Residual standard error: 0.04679 on 5 degrees of freedom
Multiple R-squared:  0.3466,     Adjusted R-squared:  0.2159
F-statistic: 2.652 on 1 and 5 DF,  p-value: 0.1644


Response MidlineDistance_RF :

Call:
lm(formula = MidlineDistance_RF ~ clusterID)

Residuals:
CC041XCC012       CC012 CC032XCC013       CC017       CC023
CC012XCC032       CC057
     1.2282     -0.4887     -0.7396      0.9340     -1.4595
0.3957      0.1298

Coefficients:
            Estimate Std. Error t value Pr(>|t|)
(Intercept)  -0.8821     0.6039  -1.461    0.204
clusterID2    1.2988     0.7989   1.626    0.165

Residual standard error: 1.046 on 5 degrees of freedom
Multiple R-squared:  0.3458,     Adjusted R-squared:  0.215
F-statistic: 2.643 on 1 and 5 DF,  p-value: 0.1649


Response pBrakeStance_RF :

Call:
lm(formula = pBrakeStance_RF ~ clusterID)

Residuals:
CC041XCC012       CC012 CC032XCC013       CC017       CC023
CC012XCC032       CC057
   0.925533   -0.861107   -0.064426    0.950339   -0.005629
-1.128929    0.184218

Coefficients:
```

```
            Estimate Std. Error t value Pr(>|t|)
(Intercept)  -0.4558     0.5042  -0.904    0.407
clusterID2    1.0819     0.6670   1.622    0.166

Residual standard error: 0.8734 on 5 degrees of freedom
Multiple R-squared:  0.3447,	Adjusted R-squared:  0.2137 
F-statistic: 2.631 on 1 and 5 DF,  p-value: 0.1657


Response pPropelStance_RF :

Call:
lm(formula = pPropelStance_RF ~ clusterID)

Residuals:
CC041XCC012       CC012 CC032XCC013       CC017       CC023 
CC012XCC032       CC057 
  -0.925533    0.861107    0.064426   -0.950339    0.005629 
1.128929   -0.184218 

Coefficients:
            Estimate Std. Error t value Pr(>|t|)
(Intercept)   0.4558     0.5042   0.904    0.407
clusterID2   -1.0819     0.6670  -1.622    0.166

Residual standard error: 0.8734 on 5 degrees of freedom
Multiple R-squared:  0.3447,	Adjusted R-squared:  0.2137 
F-statistic: 2.631 on 1 and 5 DF,  p-value: 0.1657


Response StepAngleVar_LF :

Call:
lm(formula = StepAngleVar_LF ~ clusterID)

Residuals:
CC041XCC012       CC012 CC032XCC013       CC017       CC023 
CC012XCC032       CC057 
    0.04371    -0.38142     0.33771    -0.16457    -0.94717 
1.53654    -0.42480 

Coefficients:
            Estimate Std. Error t value Pr(>|t|)
(Intercept)  -0.1727     0.4985  -0.346    0.743
clusterID2    1.0541     0.6594   1.599    0.171

Residual standard error: 0.8634 on 5 degrees of freedom
Multiple R-squared:  0.3382,	Adjusted R-squared:  0.2059 
F-statistic: 2.555 on 1 and 5 DF,  p-value: 0.1708
```

```
Response pStanceStride_RF :

Call:
lm(formula = pStanceStride_RF ~ clusterID)

Residuals:
CC041XCC012       CC012 CC032XCC013       CC017       CC023
CC012XCC032       CC057
     1.1242     -0.8552     -0.2690     -0.1512     -0.1887
-0.1247      0.4646

Coefficients:
            Estimate Std. Error t value Pr(>|t|)
(Intercept)   0.2998     0.3964   0.756    0.484
clusterID2   -0.7823     0.5244  -1.492    0.196

Residual standard error: 0.6866 on 5 degrees of freedom
Multiple R-squared:  0.308,	Adjusted R-squared:  0.1696
F-statistic: 2.225 on 1 and 5 DF,  p-value: 0.196


Response pSwingStride_RF :

Call:
lm(formula = pSwingStride_RF ~ clusterID)

Residuals:
CC041XCC012       CC012 CC032XCC013       CC017       CC023
CC012XCC032       CC057
    -1.1242      0.8552      0.2690      0.1512      0.1887
0.1247     -0.4646

Coefficients:
            Estimate Std. Error t value Pr(>|t|)
(Intercept)  -0.2998     0.3964  -0.756    0.484
clusterID2    0.7823     0.5244   1.492    0.196

Residual standard error: 0.6866 on 5 degrees of freedom
Multiple R-squared:  0.308,	Adjusted R-squared:  0.1696
F-statistic: 2.225 on 1 and 5 DF,  p-value: 0.196


Response Brake_LF :

Call:
lm(formula = Brake_LF ~ clusterID)

Residuals:
CC041XCC012       CC012 CC032XCC013       CC017       CC023
```

```
CC012XCC032       CC057
     0.1624     -1.1357      0.9733      0.7620     -1.1006
-0.3624      0.7010

Coefficients:
            Estimate Std. Error t value Pr(>|t|)
(Intercept)   0.7822     0.5585   1.401    0.220
clusterID2   -1.0834     0.7388  -1.466    0.202

Residual standard error: 0.9673 on 5 degrees of freedom
Multiple R-squared:  0.3008,      Adjusted R-squared:  0.1609
F-statistic: 2.151 on 1 and 5 DF,  p-value: 0.2024


Response Propel_LH :

Call:
lm(formula = Propel_LH ~ clusterID)

Residuals:
CC041XCC012       CC012 CC032XCC013       CC017       CC023
CC012XCC032       CC057
     0.2690     -0.4385      0.1696      1.1279      1.4011
-2.2605     -0.2685

Coefficients:
            Estimate Std. Error t value Pr(>|t|)
(Intercept)   0.5571     0.7620   0.731    0.498
clusterID2   -1.4783     1.0081  -1.466    0.202

Residual standard error: 1.32 on 5 degrees of freedom
Multiple R-squared:  0.3007,      Adjusted R-squared:  0.1609
F-statistic:  2.15 on 1 and 5 DF,  p-value: 0.2024


Response AxisDistance_RH :

Call:
lm(formula = AxisDistance_RH ~ clusterID)

Residuals:
CC041XCC012       CC012 CC032XCC013       CC017       CC023
CC012XCC032       CC057
  -0.512849   -1.075835    1.588683    0.009437   -0.212731
-0.043444    0.246738

Coefficients:
            Estimate Std. Error t value Pr(>|t|)
(Intercept)   0.8779     0.5198   1.689    0.152
clusterID2   -0.9496     0.6876  -1.381    0.226
```

```
Residual standard error: 0.9003 on 5 degrees of freedom
Multiple R-squared:  0.2761,	Adjusted R-squared:  0.1313 
F-statistic: 1.907 on 1 and 5 DF,  p-value: 0.2258


Response nSteps_LH :

Call:
lm(formula = nSteps_LH ~ clusterID)

Residuals:
CC041XCC012       CC012 CC032XCC013       CC017       CC023 
CC012XCC032       CC057 
    -0.4903      0.8276     -0.3373     -0.7524     -1.2688 
1.5873      0.4339 

Coefficients:
            Estimate Std. Error t value Pr(>|t|)
(Intercept)  -0.2065     0.6284  -0.329    0.756
clusterID2    1.1015     0.8313   1.325    0.242

Residual standard error: 1.088 on 5 degrees of freedom
Multiple R-squared:  0.2599,	Adjusted R-squared:  0.1119 
F-statistic: 1.756 on 1 and 5 DF,  p-value: 0.2425


Response StanceratioSwing_LF :

Call:
lm(formula = StanceratioSwing_LF ~ clusterID)

Residuals:
CC041XCC012       CC012 CC032XCC013       CC017       CC023 
CC012XCC032       CC057 
    0.79819     0.02088    -0.81907    -0.58298    -0.62854 
0.76236     0.44916 

Coefficients:
            Estimate Std. Error t value Pr(>|t|)
(Intercept)  -0.5551     0.4341  -1.279    0.257
clusterID2    0.7235     0.5742   1.260    0.263

Residual standard error: 0.7518 on 5 degrees of freedom
Multiple R-squared:  0.241,	Adjusted R-squared:  0.08915 
F-statistic: 1.587 on 1 and 5 DF,  p-value: 0.2633


Response StepAngleCV_LF :
```

```
Call:
lm(formula = StepAngleCV_LF ~ clusterID)

Residuals:
CC041XCC012       CC012 CC032XCC013       CC017       CC023
CC012XCC032       CC057
    0.17101    -0.36736     0.19635    -0.01042    -1.59005
1.20183     0.39865

Coefficients:
            Estimate Std. Error t value Pr(>|t|)
(Intercept) -0.02823    0.53755  -0.053     0.96
clusterID2   0.84091    0.71112   1.183     0.29

Residual standard error: 0.9311 on 5 degrees of freedom
Multiple R-squared:  0.2186,	Adjusted R-squared:  0.06226
F-statistic: 1.398 on 1 and 5 DF,  p-value: 0.2902


Response StanceFactor_LH :

Call:
lm(formula = StanceFactor_LH ~ clusterID)

Residuals:
CC041XCC012       CC012 CC032XCC013       CC017       CC023
CC012XCC032       CC057
   -0.07584     1.54332    -1.46748    -0.43146     0.53200
0.46511    -0.56565

Coefficients:
            Estimate Std. Error t value Pr(>|t|)
(Intercept)  -0.9563     0.6081  -1.573    0.177
clusterID2    0.9106     0.8044   1.132    0.309

Residual standard error: 1.053 on 5 degrees of freedom
Multiple R-squared:  0.204,	Adjusted R-squared:  0.04479
F-statistic: 1.281 on 1 and 5 DF,  p-value: 0.309


Response StanceratioSwing_RF :

Call:
lm(formula = StanceratioSwing_RF ~ clusterID)

Residuals:
CC041XCC012       CC012 CC032XCC013       CC017       CC023
CC012XCC032       CC057
    0.89530    -0.81723    -0.07807    -0.10475    -0.34957
-0.14359     0.59791
```

```
Coefficients:
            Estimate Std. Error t value Pr(>|t|)
(Intercept)   0.3406     0.3639   0.936    0.392
clusterID2   -0.5289     0.4815  -1.098    0.322

Residual standard error: 0.6304 on 5 degrees of freedom
Multiple R-squared:  0.1944,      Adjusted R-squared:  0.0333
F-statistic: 1.207 on 1 and 5 DF,  p-value: 0.3221


Response OverlapDistance_LF :

Call:
lm(formula = OverlapDistance_LF ~ clusterID)

Residuals:
CC041XCC012       CC012 CC032XCC013       CC017       CC023
CC012XCC032       CC057
   -1.46802    -1.19579     2.66381    -0.58075     1.06212
-0.45631    -0.02507

Coefficients:
            Estimate Std. Error t value Pr(>|t|)
(Intercept)   0.8698     0.9076   0.958    0.382
clusterID2   -1.2981     1.2006  -1.081    0.329

Residual standard error: 1.572 on 5 degrees of freedom
Multiple R-squared:  0.1895,      Adjusted R-squared:  0.02739
F-statistic: 1.169 on 1 and 5 DF,  p-value: 0.329


Response OverlapDistance_LH :

Call:
lm(formula = OverlapDistance_LH ~ clusterID)

Residuals:
CC041XCC012       CC012 CC032XCC013       CC017       CC023
CC012XCC032       CC057
   -1.50684    -1.18588     2.69272    -0.58361     1.06735
-0.45855    -0.02519

Coefficients:
            Estimate Std. Error t value Pr(>|t|)
(Intercept)   0.8808     0.9172   0.960    0.381
clusterID2   -1.2887     1.2133  -1.062    0.337

Residual standard error: 1.589 on 5 degrees of freedom
Multiple R-squared:  0.1841,      Adjusted R-squared:  0.0209
```

```
F-statistic: 1.128 on 1 and 5 DF,  p-value: 0.3368


Response nSteps_RH :

Call:
lm(formula = nSteps_RH ~ clusterID)

Residuals:
CC041XCC012       CC012 CC032XCC013       CC017       CC023
CC012XCC032       CC057
    -0.6065      1.3138     -0.7072     -0.6555     -1.2992
1.6303      0.3244

Coefficients:
            Estimate Std. Error t value Pr(>|t|)
(Intercept)  -0.1347     0.7059  -0.191    0.856
clusterID2    0.9821     0.9338   1.052    0.341

Residual standard error: 1.223 on 5 degrees of freedom
Multiple R-squared:  0.1812,      Adjusted R-squared:  0.01738
F-statistic: 1.106 on 1 and 5 DF,  p-value: 0.3411


Response pBrakeStride_RF :

Call:
lm(formula = pBrakeStride_RF ~ clusterID)

Residuals:
CC041XCC012       CC012 CC032XCC013       CC017       CC023
CC012XCC032       CC057
    1.15392    -1.02619    -0.12773     0.78249    -0.06719
-1.07850     0.36321

Coefficients:
            Estimate Std. Error t value Pr(>|t|)
(Intercept)  -0.2945     0.5362  -0.549    0.606
clusterID2    0.6985     0.7093   0.985    0.370

Residual standard error: 0.9287 on 5 degrees of freedom
Multiple R-squared:  0.1624,      Adjusted R-squared:  -0.005067
F-statistic: 0.9698 on 1 and 5 DF,  p-value: 0.37


Response pSharedStance_RH :

Call:
lm(formula = pSharedStance_RH ~ clusterID)
```

```
Residuals:
CC041XCC012       CC012 CC032XCC013       CC017       CC023
CC012XCC032       CC057
    -0.2822      0.8478     -0.5657     -0.1542      1.5521
-0.7267     -0.6712

Coefficients:
            Estimate Std. Error t value Pr(>|t|)
(Intercept)  -0.5246     0.5495  -0.955    0.384
clusterID2    0.6695     0.7270   0.921    0.399

Residual standard error: 0.9518 on 5 degrees of freedom
Multiple R-squared:  0.145,	Adjusted R-squared:  -0.02596
F-statistic: 0.8482 on 1 and 5 DF,  p-value: 0.3993


Response pBrakeStride_LH :

Call:
lm(formula = pBrakeStride_LH ~ clusterID)

Residuals:
CC041XCC012       CC012 CC032XCC013       CC017       CC023
CC012XCC032       CC057
    -0.3399      0.1471      0.1929     -0.6910     -0.8816
1.9249     -0.3523

Coefficients:
            Estimate Std. Error t value Pr(>|t|)
(Intercept)   0.4381     0.5921   0.740    0.493
clusterID2   -0.7213     0.7833  -0.921    0.399

Residual standard error: 1.026 on 5 degrees of freedom
Multiple R-squared:  0.145,	Adjusted R-squared:  -0.02598
F-statistic: 0.8481 on 1 and 5 DF,  p-value: 0.3994


Response pBrakeStance_LH :

Call:
lm(formula = pBrakeStance_LH ~ clusterID)

Residuals:
CC041XCC012       CC012 CC032XCC013       CC017       CC023
CC012XCC032       CC057
    -0.4199      0.2339      0.1860     -0.7898     -0.9738
2.1020     -0.3384

Coefficients:
            Estimate Std. Error t value Pr(>|t|)
```

```
(Intercept)   0.4814     0.6517   0.739    0.493
clusterID2   -0.7873     0.8621  -0.913    0.403

Residual standard error: 1.129 on 5 degrees of freedom
Multiple R-squared:  0.143,	Adjusted R-squared:  -0.02846 
F-statistic: 0.834 on 1 and 5 DF,  p-value: 0.403


Response pPropelStance_LH :

Call:
lm(formula = pPropelStance_LH ~ clusterID)

Residuals:
CC041XCC012       CC012 CC032XCC013       CC017       CC023 
CC012XCC032       CC057 
     0.4199     -0.2339     -0.1860      0.7898      0.9738 
-2.1021      0.3384 

Coefficients:
            Estimate Std. Error t value Pr(>|t|)
(Intercept)  -0.4813     0.6517  -0.739    0.493
clusterID2    0.7873     0.8621   0.913    0.403

Residual standard error: 1.129 on 5 degrees of freedom
Multiple R-squared:  0.143,	Adjusted R-squared:  -0.02846 
F-statistic: 0.834 on 1 and 5 DF,  p-value: 0.403


Response MINdAratiodT_RH :

Call:
lm(formula = MINdAratiodT_RH ~ clusterID)

Residuals:
CC041XCC012       CC012 CC032XCC013       CC017       CC023 
CC012XCC032       CC057 
  -0.732380    0.260499    0.471880   -0.006897   -0.234674 
-0.544179    0.785750 

Coefficients:
             Estimate Std. Error t value Pr(>|t|)
(Intercept)  0.006719   0.345983   0.019    0.985
clusterID2  -0.366114   0.457693  -0.800    0.460

Residual standard error: 0.5993 on 5 degrees of freedom
Multiple R-squared:  0.1135,	Adjusted R-squared:  -0.06386 
F-statistic: 0.6399 on 1 and 5 DF,  p-value: 0.4601
```

```
Response pBrakeStride_RH :

Call:
lm(formula = pBrakeStride_RH ~ clusterID)

Residuals:
CC041XCC012       CC012 CC032XCC013       CC017       CC023
CC012XCC032       CC057
    0.03624     0.14749    -0.18373    -1.29400     0.30892
2.20009    -1.21501

Coefficients:
            Estimate Std. Error t value Pr(>|t|)
(Intercept)  -0.2386     0.7368  -0.324    0.759
clusterID2    0.7132     0.9747   0.732    0.497

Residual standard error: 1.276 on 5 degrees of freedom
Multiple R-squared:  0.09673,     Adjusted R-squared:  -0.08393
F-statistic: 0.5354 on 1 and 5 DF,  p-value: 0.4972


Response MidlineDistance_LF :

Call:
lm(formula = MidlineDistance_LF ~ clusterID)

Residuals:
CC041XCC012       CC012 CC032XCC013       CC017       CC023
CC012XCC032       CC057
     0.1884      0.1243     -0.3126      0.9853     -2.0342
0.7362      0.3128

Coefficients:
            Estimate Std. Error t value Pr(>|t|)
(Intercept)  -0.3134     0.6270  -0.500    0.638
clusterID2    0.6005     0.8295   0.724    0.502

Residual standard error: 1.086 on 5 degrees of freedom
Multiple R-squared:  0.09486,     Adjusted R-squared:  -0.08616
F-statistic: 0.524 on 1 and 5 DF,  p-value: 0.5016


Response StanceWidth_LF :

Call:
lm(formula = StanceWidth_LF ~ clusterID)

Residuals:
CC041XCC012       CC012 CC032XCC013       CC017       CC023
CC012XCC032       CC057
```

```
   -0.06094    -0.30741     0.36835     0.84643    -1.70849 
0.81866     0.04339 

Coefficients:
            Estimate Std. Error t value Pr(>|t|)
(Intercept)   0.3611     0.5502   0.656    0.541
clusterID2   -0.5151     0.7279  -0.708    0.511

Residual standard error: 0.953 on 5 degrees of freedom
Multiple R-squared:  0.09104,	Adjusted R-squared:  -0.09075 
F-statistic: 0.5008 on 1 and 5 DF,  p-value: 0.5108


Response pPropelStride_RH :

Call:
lm(formula = pPropelStride_RH ~ clusterID)

Residuals:
CC041XCC012       CC012 CC032XCC013       CC017       CC023 
CC012XCC032       CC057 
    0.56697    -0.66584     0.09887     1.47746     0.41351 
-2.67699     0.78602 

Coefficients:
            Estimate Std. Error t value Pr(>|t|)
(Intercept)   0.4089     0.8529   0.479    0.652
clusterID2   -0.7482     1.1283  -0.663    0.537

Residual standard error: 1.477 on 5 degrees of freedom
Multiple R-squared:  0.08083,	Adjusted R-squared:  -0.103 
F-statistic: 0.4397 on 1 and 5 DF,  p-value: 0.5366


Response pPropelStance_RH :

Call:
lm(formula = pPropelStance_RH ~ clusterID)

Residuals:
CC041XCC012       CC012 CC032XCC013       CC017       CC023 
CC012XCC032       CC057 
     0.1558     -0.3406      0.1847      1.5265     -0.1200 
-2.4431      1.0365 

Coefficients:
            Estimate Std. Error t value Pr(>|t|)
(Intercept)   0.3066     0.7984   0.384    0.717
clusterID2   -0.6996     1.0562  -0.662    0.537
```

```
Residual standard error: 1.383 on 5 degrees of freedom
Multiple R-squared:  0.08067,	Adjusted R-squared:  -0.1032
F-statistic: 0.4388 on 1 and 5 DF,  p-value: 0.537


Response pBrakeStance_RH :

Call:
lm(formula = pBrakeStance_RH ~ clusterID)

Residuals:
CC041XCC012       CC012 CC032XCC013       CC017       CC023
CC012XCC032       CC057
    -0.1558      0.3406     -0.1847     -1.5265      0.1200
2.4431     -1.0365

Coefficients:
            Estimate Std. Error t value Pr(>|t|)
(Intercept)  -0.3066     0.7984  -0.384    0.717
clusterID2    0.6996     1.0562   0.662    0.537

Residual standard error: 1.383 on 5 degrees of freedom
Multiple R-squared:  0.08067,	Adjusted R-squared:  -0.1032
F-statistic: 0.4388 on 1 and 5 DF,  p-value: 0.537


Response StanceWidth_LH :

Call:
lm(formula = StanceWidth_LH ~ clusterID)

Residuals:
CC041XCC012       CC012 CC032XCC013       CC017       CC023
CC012XCC032       CC057
   -0.39239    -0.18567     0.57806     0.07034    -0.11341
0.13064    -0.08757

Coefficients:
            Estimate Std. Error t value Pr(>|t|)
(Intercept)   0.2609     0.1941   1.344    0.237
clusterID2   -0.1664     0.2568  -0.648    0.545

Residual standard error: 0.3362 on 5 degrees of freedom
Multiple R-squared:  0.07751,	Adjusted R-squared:  -0.107
F-statistic: 0.4201 on 1 and 5 DF,  p-value: 0.5455


Response Brake_RH :

Call:
```

```
lm(formula = Brake_RH ~ clusterID)

Residuals:
CC041XCC012       CC012 CC032XCC013       CC017       CC023
CC012XCC032       CC057
     0.1008     -0.2951      0.1942     -0.3558      0.1532
0.8758     -0.6732

Coefficients:
            Estimate Std. Error t value Pr(>|t|)
(Intercept)   0.4908     0.3168   1.549    0.182
clusterID2   -0.2618     0.4191  -0.625    0.560

Residual standard error: 0.5487 on 5 degrees of freedom
Multiple R-squared:  0.07241,     Adjusted R-squared:  -0.1131
F-statistic: 0.3903 on 1 and 5 DF,  p-value: 0.5595


Response pPropelStride_LH :

Call:
lm(formula = pPropelStride_LH ~ clusterID)

Residuals:
CC041XCC012       CC012 CC032XCC013       CC017       CC023
CC012XCC032       CC057
   0.431204   -0.435935    0.004731    0.716222    1.352083
-2.510551    0.442247

Coefficients:
            Estimate Std. Error t value Pr(>|t|)
(Intercept)  -0.4525     0.7838  -0.577    0.589
clusterID2    0.6143     1.0369   0.592    0.579

Residual standard error: 1.358 on 5 degrees of freedom
Multiple R-squared:  0.06558,     Adjusted R-squared:  -0.1213
F-statistic: 0.3509 on 1 and 5 DF,  p-value: 0.5794


Response AbsolutePawAngle_RH :

Call:
lm(formula = AbsolutePawAngle_RH ~ clusterID)

Residuals:
CC041XCC012       CC012 CC032XCC013       CC017       CC023
CC012XCC032       CC057
    -1.5808     -0.1130      1.6938     -0.3059      0.1786
-0.3576      0.4849
```

```
Coefficients:
            Estimate Std. Error t value Pr(>|t|)
(Intercept)  -1.0313     0.6255  -1.649    0.160
clusterID2    0.4843     0.8275   0.585    0.584

Residual standard error: 1.083 on 5 degrees of freedom
Multiple R-squared:  0.06411,	Adjusted R-squared:  -0.1231 
F-statistic: 0.3425 on 1 and 5 DF,  p-value: 0.5838


Response PawPlacementPositioning[PPP]_LH :

Call:
lm(formula = `PawPlacementPositioning[PPP]_LH` ~ clusterID)

Residuals:
CC041XCC012       CC012 CC032XCC013       CC017       CC023 
CC012XCC032       CC057 
   -1.49260     0.89177     0.60083    -0.81503     0.65408 
0.06845     0.09250 

Coefficients:
            Estimate Std. Error t value Pr(>|t|)
(Intercept)  -0.7207     0.5471  -1.317    0.245
clusterID2    0.3891     0.7237   0.538    0.614

Residual standard error: 0.9476 on 5 degrees of freedom
Multiple R-squared:  0.05466,	Adjusted R-squared:  -0.1344 
F-statistic: 0.2891 on 1 and 5 DF,  p-value: 0.6139


Response PawPlacementPositioning[PPP]_LF :

Call:
lm(formula = `PawPlacementPositioning[PPP]_LF` ~ clusterID)

Residuals:
CC041XCC012       CC012 CC032XCC013       CC017       CC023 
CC012XCC032       CC057 
   -1.47393     0.88220     0.59173    -0.81373     0.65304 
0.06834     0.09235 

Coefficients:
            Estimate Std. Error t value Pr(>|t|)
(Intercept)  -0.7160     0.5418  -1.322    0.244
clusterID2    0.3804     0.7167   0.531    0.618

Residual standard error: 0.9384 on 5 degrees of freedom
Multiple R-squared:  0.05333,	Adjusted R-squared:  -0.136 
F-statistic: 0.2816 on 1 and 5 DF,  p-value: 0.6183
```

```
Response PawAngle_RH :

Call:
lm(formula = PawAngle_RH ~ clusterID)

Residuals:
CC041XCC012       CC012 CC032XCC013       CC017       CC023
CC012XCC032       CC057
    -1.4775     -0.0681      1.5456     -0.2575      0.2304
-0.4769      0.5040

Coefficients:
            Estimate Std. Error t value Pr(>|t|)
(Intercept)  -0.9760     0.5875  -1.661    0.158
clusterID2    0.3944     0.7772   0.508    0.633

Residual standard error: 1.018 on 5 degrees of freedom
Multiple R-squared:  0.04899,     Adjusted R-squared:  -0.1412
F-statistic: 0.2576 on 1 and 5 DF,  p-value: 0.6334


Response OverlapDistance_RH :

Call:
lm(formula = OverlapDistance_RH ~ clusterID)

Residuals:
CC041XCC012       CC012 CC032XCC013       CC017       CC023
CC012XCC032       CC057
   0.008319    0.760782   -0.769101   -1.885635    2.225880
0.090735   -0.430980

Coefficients:
            Estimate Std. Error t value Pr(>|t|)
(Intercept)  -0.2798     0.8114  -0.345    0.744
clusterID2   -0.4980     1.0733  -0.464    0.662

Residual standard error: 1.405 on 5 degrees of freedom
Multiple R-squared:  0.04127,     Adjusted R-squared:  -0.1505
F-statistic: 0.2153 on 1 and 5 DF,  p-value: 0.6622


Response OverlapDistance_RF :

Call:
lm(formula = OverlapDistance_RF ~ clusterID)

Residuals:
```

```
CC041XCC012       CC012 CC032XCC013       CC017       CC023
CC012XCC032       CC057
   0.008338    0.762582   -0.770920   -1.890096    2.231146
0.090950   -0.432000

Coefficients:
            Estimate Std. Error t value Pr(>|t|)
(Intercept)  -0.2533     0.8133  -0.311    0.768
clusterID2   -0.4992     1.0759  -0.464    0.662

Residual standard error: 1.409 on 5 degrees of freedom
Multiple R-squared:  0.04127,     Adjusted R-squared:  -0.1505
F-statistic: 0.2153 on 1 and 5 DF,  p-value: 0.6622


Response pStanceStride_RH :

Call:
lm(formula = pStanceStride_RH ~ clusterID)

Residuals:
CC041XCC012       CC012 CC032XCC013       CC017       CC023
CC012XCC032       CC057
    0.77367    -0.74067    -0.03300     0.77881     0.81960
-1.54483    -0.05358

Coefficients:
            Estimate Std. Error t value Pr(>|t|)
(Intercept)   0.2361     0.5666   0.417    0.694
clusterID2   -0.3454     0.7496  -0.461    0.664

Residual standard error: 0.9814 on 5 degrees of freedom
Multiple R-squared:  0.04074,     Adjusted R-squared:  -0.1511
F-statistic: 0.2124 on 1 and 5 DF,  p-value: 0.6643


Response pSwingStride_RH :

Call:
lm(formula = pSwingStride_RH ~ clusterID)

Residuals:
CC041XCC012       CC012 CC032XCC013       CC017       CC023
CC012XCC032       CC057
   -0.97465     0.93307     0.04157    -0.98112    -1.03251
1.94613     0.06750

Coefficients:
            Estimate Std. Error t value Pr(>|t|)
(Intercept)  -0.4511     0.7138  -0.632    0.555
```

```
clusterID2    0.4351     0.9443   0.461    0.664

Residual standard error: 1.236 on 5 degrees of freedom
Multiple R-squared:  0.04074,	Adjusted R-squared:  -0.1511 
F-statistic: 0.2124 on 1 and 5 DF,  p-value: 0.6643


Response PawPlacementPositioning[PPP]_RF :

Call:
lm(formula = `PawPlacementPositioning[PPP]_RF` ~ clusterID)

Residuals:
CC041XCC012       CC012 CC032XCC013       CC017       CC023 
CC012XCC032       CC057 
    0.49715    -1.23221     0.73505    -0.06008     1.84406 
-0.86502    -0.91896 

Coefficients:
            Estimate Std. Error t value Pr(>|t|)
(Intercept)   0.5979     0.6977   0.857    0.431
clusterID2   -0.4230     0.9230  -0.458    0.666

Residual standard error: 1.209 on 5 degrees of freedom
Multiple R-squared:  0.0403,	Adjusted R-squared:  -0.1516 
F-statistic:  0.21 on 1 and 5 DF,  p-value: 0.666


Response PawPlacementPositioning[PPP]_RH :

Call:
lm(formula = `PawPlacementPositioning[PPP]_RH` ~ clusterID)

Residuals:
CC041XCC012       CC012 CC032XCC013       CC017       CC023 
CC012XCC032       CC057 
    0.48960    -1.21349     0.72389    -0.05917     1.81606 
-0.85189    -0.90501 

Coefficients:
            Estimate Std. Error t value Pr(>|t|)
(Intercept)   0.5773     0.6871   0.840    0.439
clusterID2   -0.4165     0.9090  -0.458    0.666

Residual standard error: 1.19 on 5 degrees of freedom
Multiple R-squared:  0.0403,	Adjusted R-squared:  -0.1516 
F-statistic:  0.21 on 1 and 5 DF,  p-value: 0.666


Response AbsolutePawAngle_LH :
```

```
Call:
lm(formula = AbsolutePawAngle_LH ~ clusterID)

Residuals:
CC041XCC012       CC012 CC032XCC013       CC017       CC023
CC012XCC032       CC057
   -1.50530     0.81946     0.68585    -0.14423    -0.09976
-0.63852     0.88250

Coefficients:
            Estimate Std. Error t value Pr(>|t|)
(Intercept)  -0.2903     0.5553  -0.523    0.623
clusterID2   -0.3193     0.7346  -0.435    0.682

Residual standard error: 0.9618 on 5 degrees of freedom
Multiple R-squared:  0.03641,    Adjusted R-squared:  -0.1563
F-statistic: 0.1889 on 1 and 5 DF,  p-value: 0.6819


Response pSharedStance_LH :

Call:
lm(formula = pSharedStance_LH ~ clusterID)

Residuals:
CC041XCC012       CC012 CC032XCC013       CC017       CC023
CC012XCC032       CC057
    0.36653    -0.92717     0.56065     0.25893     1.45212
-1.62464    -0.08641

Coefficients:
            Estimate Std. Error t value Pr(>|t|)
(Intercept)  0.02373    0.63932   0.037    0.972
clusterID2   0.34876    0.84574   0.412    0.697

Residual standard error: 1.107 on 5 degrees of freedom
Multiple R-squared:  0.03289,    Adjusted R-squared:  -0.1605
F-statistic: 0.1701 on 1 and 5 DF,  p-value: 0.6971


Response PawAreaVariabilityatPeakStanceinsq.cm_LF :

Call:
lm(formula = PawAreaVariabilityatPeakStanceinsq.cm_LF ~ clusterID)

Residuals:
CC041XCC012       CC012 CC032XCC013       CC017       CC023
CC012XCC032       CC057
    -0.1452      1.7088     -1.5636      0.6784     -0.7889
```

```
0.7535     -0.6430

Coefficients:
            Estimate Std. Error t value Pr(>|t|)
(Intercept)  -0.1808     0.7047  -0.257    0.808
clusterID2    0.3300     0.9323   0.354    0.738

Residual standard error: 1.221 on 5 degrees of freedom
Multiple R-squared:  0.02445,     Adjusted R-squared:  -0.1707
F-statistic: 0.1253 on 1 and 5 DF,  p-value: 0.7378


Response MINdAratiodT_LH :

Call:
lm(formula = MINdAratiodT_LH ~ clusterID)

Residuals:
CC041XCC012       CC012 CC032XCC013       CC017       CC023
CC012XCC032       CC057
     0.5247     -1.4135      0.8889      0.2250      1.0459
-1.1698     -0.1011

Coefficients:
            Estimate Std. Error t value Pr(>|t|)
(Intercept)   0.1191     0.6103   0.195    0.853
clusterID2   -0.2782     0.8073  -0.345    0.744

Residual standard error: 1.057 on 5 degrees of freedom
Multiple R-squared:  0.0232,      Adjusted R-squared:  -0.1722
F-statistic: 0.1187 on 1 and 5 DF,  p-value: 0.7444


Response StanceratioSwing_RH :

Call:
lm(formula = StanceratioSwing_RH ~ clusterID)

Residuals:
CC041XCC012       CC012 CC032XCC013       CC017       CC023
CC012XCC032       CC057
    1.04282    -0.90585    -0.13696     0.49975     1.75371
-2.28296     0.02951

Coefficients:
            Estimate Std. Error t value Pr(>|t|)
(Intercept)   0.1083     0.8353   0.130    0.902
clusterID2   -0.2523     1.1049  -0.228    0.828

Residual standard error: 1.447 on 5 degrees of freedom
```

```
Multiple R-squared:  0.01032,	Adjusted R-squared:  -0.1876 
F-statistic: 0.05212 on 1 and 5 DF,  p-value: 0.8285


Response StanceratioSwing_LH :

Call:
lm(formula = StanceratioSwing_LH ~ clusterID)

Residuals:
CC041XCC012       CC012 CC032XCC013       CC017       CC023 
CC012XCC032       CC057 
     0.3605     -0.5421      0.1816      0.1375      1.5441 
-2.2162      0.5345 

Coefficients:
             Estimate Std. Error t value Pr(>|t|)
(Intercept) -0.002706   0.732895  -0.004    0.997
clusterID2  -0.210728   0.969529  -0.217    0.837

Residual standard error: 1.269 on 5 degrees of freedom
Multiple R-squared:  0.00936,	Adjusted R-squared:  -0.1888 
F-statistic: 0.04724 on 1 and 5 DF,  p-value: 0.8365


Response PawAreaVariabilityatPeakStanceinsq.cm_RH :

Call:
lm(formula = PawAreaVariabilityatPeakStanceinsq.cm_RH ~ clusterID)

Residuals:
CC041XCC012       CC012 CC032XCC013       CC017       CC023 
CC012XCC032       CC057 
 -0.0008619   0.0421900  -0.0413281   0.1413084  -0.1565640 
0.2429263  -0.2276707 

Coefficients:
            Estimate Std. Error t value Pr(>|t|)
(Intercept) -0.18936    0.10290  -1.840    0.125
clusterID2  -0.02465    0.13612  -0.181    0.863

Residual standard error: 0.1782 on 5 degrees of freedom
Multiple R-squared:  0.006516,	Adjusted R-squared:  -0.1922 
F-statistic: 0.03279 on 1 and 5 DF,  p-value: 0.8634


Response AxisDistance_LH :

Call:
lm(formula = AxisDistance_LH ~ clusterID)
```

```
Residuals:
CC041XCC012       CC012 CC032XCC013       CC017       CC023
CC012XCC032       CC057
   0.083321    0.036654   -0.119975    0.003563   -0.098200
-0.018966    0.113602

Coefficients:
            Estimate Std. Error t value Pr(>|t|)
(Intercept)  0.54134    0.05514   9.818 0.000187 ***
clusterID2   0.01286    0.07294   0.176 0.866959
---
Signif. codes:  0 ë***í 0.001 ë**í 0.01 ë*í 0.05 ë.í 0.1 ë í 1

Residual standard error: 0.0955 on 5 degrees of freedom
Multiple R-squared:  0.00618,     Adjusted R-squared:  -0.1926
F-statistic: 0.03109 on 1 and 5 DF,  p-value: 0.867


Response PawAngle_LH :

Call:
lm(formula = PawAngle_LH ~ clusterID)

Residuals:
CC041XCC012       CC012 CC032XCC013       CC017       CC023
CC012XCC032       CC057
     1.2896     -0.4186     -0.8709      0.1166      0.1362
0.5678     -0.8206

Coefficients:
            Estimate Std. Error t value Pr(>|t|)
(Intercept)  0.22830    0.49157   0.464    0.662
clusterID2   0.06156    0.65028   0.095    0.928

Residual standard error: 0.8514 on 5 degrees of freedom
Multiple R-squared:  0.001789,    Adjusted R-squared:  -0.1979
F-statistic: 0.008961 on 1 and 5 DF,  p-value: 0.9283


Response StepAngle_LF :

Call:
lm(formula = StepAngle_LF ~ clusterID)

Residuals:
CC041XCC012       CC012 CC032XCC013       CC017       CC023
CC012XCC032       CC057
    -0.4802      0.2648      0.2154     -1.1308      2.2308
-0.6275     -0.4724
```

```
Coefficients:
            Estimate Std. Error t value Pr(>|t|)
(Intercept) -0.35622    0.69375  -0.513    0.629
clusterID2  -0.03285    0.91774  -0.036    0.973

Residual standard error: 1.202 on 5 degrees of freedom
Multiple R-squared:  0.0002562,   Adjusted R-squared:  -0.1997
F-statistic: 0.001281 on 1 and 5 DF,  p-value: 0.9728


Response pStanceStride_LH :

Call:
lm(formula = pStanceStride_LH ~ clusterID)

Residuals:
CC041XCC012       CC012 CC032XCC013       CC017       CC023
CC012XCC032       CC057
     0.2575     -0.5582      0.3007      0.2571      1.0901
-1.5956      0.2484

Coefficients:
            Estimate Std. Error t value Pr(>|t|)
(Intercept) -0.16385    0.53731  -0.305    0.773
clusterID2   0.01319    0.71079   0.019    0.986

Residual standard error: 0.9306 on 5 degrees of freedom
Multiple R-squared:  6.886e-05,   Adjusted R-squared:  -0.1999
F-statistic: 0.0003443 on 1 and 5 DF,  p-value: 0.9859


Response pSwingStride_LH :

Call:
lm(formula = pSwingStride_LH ~ clusterID)

Residuals:
CC041XCC012       CC012 CC032XCC013       CC017       CC023
CC012XCC032       CC057
    -0.2575      0.5582     -0.3007     -0.2571     -1.0901
1.5956     -0.2484

Coefficients:
            Estimate Std. Error t value Pr(>|t|)
(Intercept)  0.16385    0.53731   0.305    0.773
clusterID2  -0.01319    0.71079  -0.019    0.986

Residual standard error: 0.9306 on 5 degrees of freedom
Multiple R-squared:  6.886e-05,   Adjusted R-squared:  -0.1999
```

```
F-statistic: 0.0003443 on 1 and 5 DF,  p-value: 0.9859


Response pSwingStride_LF :

Call:
lm(formula = pSwingStride_LF ~ clusterID)

Residuals:
CC041XCC012       CC012 CC032XCC013       CC017       CC023
CC012XCC032       CC057
   -0.48116     0.07213     0.40904     0.22776     0.90031
-0.61824    -0.50983

Coefficients:
             Estimate Std. Error t value Pr(>|t|)
(Intercept)  0.138633   0.356709   0.389    0.714
clusterID2  -0.008041   0.471882  -0.017    0.987

Residual standard error: 0.6178 on 5 degrees of freedom
Multiple R-squared:  5.807e-05,   Adjusted R-squared:  -0.1999
F-statistic: 0.0002904 on 1 and 5 DF,  p-value: 0.9871


Response pStanceStride_LF :

Call:
lm(formula = pStanceStride_LF ~ clusterID)

Residuals:
CC041XCC012       CC012 CC032XCC013       CC017       CC023
CC012XCC032       CC057
    0.48116    -0.07213    -0.40904    -0.22776    -0.90031
0.61824     0.50983

Coefficients:
             Estimate Std. Error t value Pr(>|t|)
(Intercept) -0.138633   0.356709  -0.389    0.714
clusterID2   0.008041   0.471882   0.017    0.987

Residual standard error: 0.6178 on 5 degrees of freedom
Multiple R-squared:  5.807e-05,   Adjusted R-squared:  -0.1999
F-statistic: 0.0002904 on 1 and 5 DF,  p-value: 0.9871


Response AxisDistance_RF :

Call:
lm(formula = AxisDistance_RF ~ clusterID)
```

```
Residuals:
CC041XCC012       CC012 CC032XCC013       CC017       CC023 
CC012XCC032       CC057 
    -0.2971     -0.7157      1.0128      0.2274     -0.7718 
0.4387      0.1057 

Coefficients:
            Estimate Std. Error t value Pr(>|t|)
(Intercept) 0.138861   0.406389   0.342    0.746
clusterID2  0.001443   0.537602   0.003    0.998

Residual standard error: 0.7039 on 5 degrees of freedom
Multiple R-squared:  1.442e-06,   Adjusted R-squared:   -0.2 
F-statistic: 7.208e-06 on 1 and 5 DF,  p-value: 0.998
```
